# Computationally Assisted Analysis of NMR Chemical Shifts as a Tool in Conformational Analysis

**DOI:** 10.1021/acs.orglett.4c01642

**Published:** 2024-06-18

**Authors:** Cristina Cuadrado, Francisco Cen-Pacheco, Antonio Hernández Daranas

**Affiliations:** † Instituto de Productos Naturales y Agrobiología del CSIC (IPNA-CSIC), La Laguna, 38206 Tenerife, Spain.; ‡ Faculty of Bioanalysis, Iturbide s/n, Veracruz University, 91700 Veracruz, Veracruz, México.

## Abstract

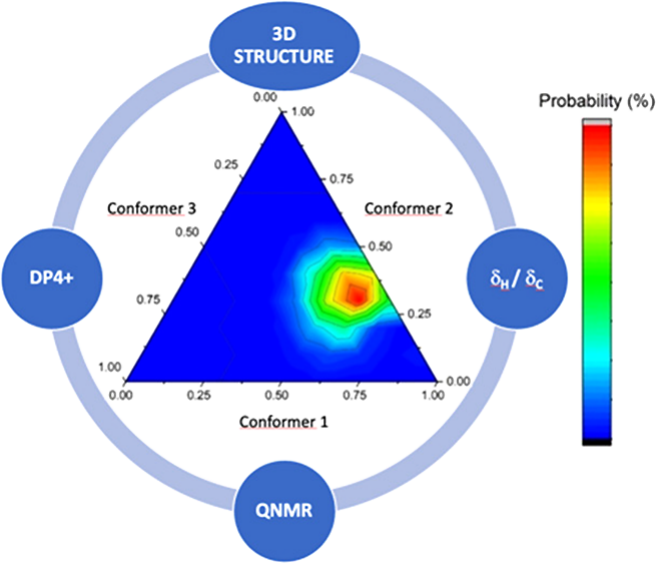

A key to understanding the properties of functional molecules is to determine their conformation in solution. A conformational analysis procedure that relies on quantum mechanical calculations and the widely used DP4+ probability was evaluated to decipher the structural information encoded in NMR chemical shifts. The results underscore the potential utility of using NMR chemical shifts in advancing conformational analysis studies of complex molecules in solution.

Determining molecular structures is a fundamental challenge in both synthetic and natural product chemistry, with natural products offering vast structural and chemical diversity that drives discoveries across various scientific fields, notably medicine, biology, and chemistry.^1,2^ The accurate depiction of molecular structures, particularly in three dimensions, offers crucial insights into drug–target interactions and structure–activity relationships.^3^ NMR spectroscopy stands as the foremost technique for unraveling the structures of small organic molecules, despite persistent challenges, especially concerning H-deficient or flexible molecules.^4,5^ Traditional approaches rely heavily on coupling constants and NOE data, with recent advancements introducing residual dipolar couplings (RDCs) and residual chemical shift anisotropy (RCSA) to enhance structural validation, although experimental measurement of these remains challenging.^6−11^ Chemical shifts, uniquely sensitive to molecular structure, provide valuable information, albeit they are difficult to interpret fully. Their interpretation could potentially offer detailed insights into the molecular environment and aid in determining compatible conformations.^12^ While chemical shifts are well-established indicators of local structure in proteins, their utility in small molecule conformational analysis is limited, due to challenges in correlating them with geometrical restraints.^13^ The increasing success of quantum chemistry methods has led to the development of computational approaches for calculating NMR properties, particularly utilizing chemical shifts to address complex stereochemical problems.^14^ The DP4 probability, based on Bayes’ theorem, has emerged as a prominent metric for selecting the most likely structure.^15^ This approach has been refined with improved versions such as DP4+ and J-DP4, incorporating higher levels of theory or geometrical information from coupling constants.^16−20^

This study aims to leverage NMR chemical shift values as an additional tool for analyzing the conformation of intricate molecules. To accomplish this, we utilized a computationally assisted approach based on DP4+ probability calculations to interpret the chemical shifts. While DP4-like probabilities have traditionally aided in solving stereochemical problems by selecting one structure from multiple candidate stereoisomers, our study suggests extending its application to choose the correct conformation for a structure with a defined configuration. Compounds **1**–**5** with different conformational behaviors in solution were examined to show the power of this approach (Figure 1).^21−24^

**Figure 1:**
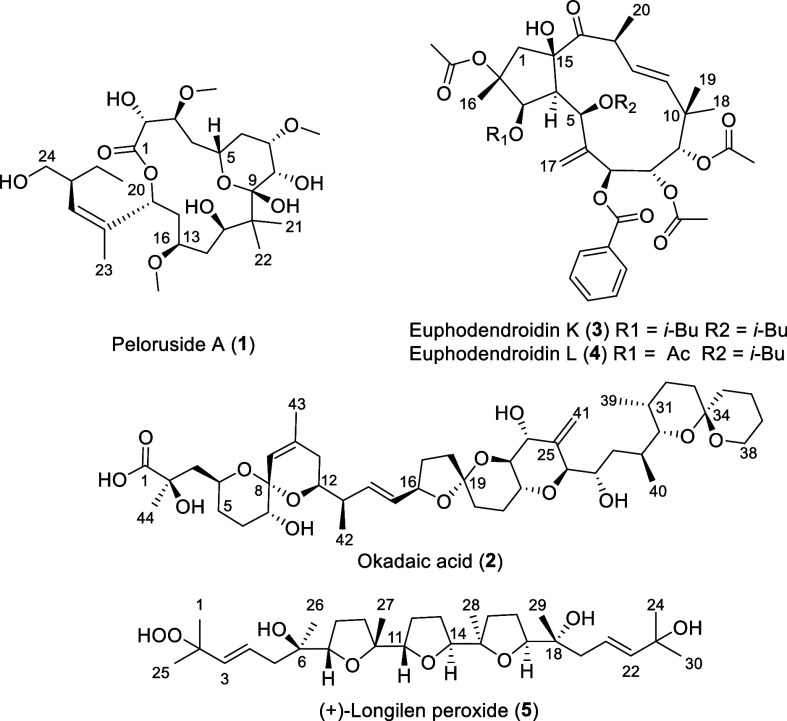
Structure of compounds **1**–**5**.

The primary aim was to assess the suitability of the previous hypothesis for medium- to large-sized molecules. Peloruside A (**1**), a natural microtubule-stabilizing agent, was selected due to its status as a complex 16-membered macrolide with limited conformational flexibility.^21^ Previous reports indicate that Peloruside A adopts a predominant conformation in CDCl_3_.^25^ Our approach begins with a rapid MMFF molecular mechanics conformational search. Reducing the number of structures for DFT calculations would be highly beneficial. However, balancing the energy cutoff and RMSD with the potential loss of significant conformations is delicate. As it can be assumed that very similar conformers would exhibit only minor differences in their chemical shifts, retaining these conformers would unnecessarily complicate the analysis. Based on previous reports, we collated structures within a 12 kJ/mol energy threshold, using a 1 Å RMSD cutoff.^20^ Using this value, the molecular backbone was fairly conserved, aligning with our primary objective of identifying the correct conformation. Subsequently, a structural optimization was conducted through DFT quantum mechanical calculations at the affordable B3LYP/6–31G* level of theory. Next, NMR chemical shifts for each conformation were calculated at the mPW1PW91/6–31+G** level of theory according to the DP4+ approach. Agreement between calculated and experimental NMR data was quantified using three different statistical parameters such as CMAE (corrected mean average error = Σn | δscaled - δexp |/n), CMaxErr (corrected maximum error = max | δscaled - δexp |), and the DP4+ probability. CMAE and CMaxErr for conformers 1–2, 1–5 and 1–9 gave the smallest errors (1–2, 1–5 and 1–9 refer to the second, fifth, and ninth conformations of the conformational search, and not to groups of conformers. See Supporting Information). Notably, conformers 1–2 and 1–5 exhibited the highest overall DP4+ probabilities, accounting for 16% and 84%, respectively. In contrast, the lowest MMFF energy conformation, 1–1, was discarded by DP4+. Figure 2 illustrates the clear differences in the macrocyclic moiety of the molecule between the 1–1 and 1–2. Specifically, the dihedral angles C9-C10-C11-C12 and C11-C12-C13-C14 exhibit significant differences, changing from ∼ 60° to ∼ 180° and from ∼ 160° to ∼ 75°, respectively. Other, less significant, geometrical changes were also observed along various other angles. On the other hand, close examination of 1–2 and 1–5 structures reveals they essentially represent a single conformation, (RMSD = 0.39 Å in Figure 2), that clearly matches with the previous reported conformation in CDCl_3_ derived from NOE and ^3^J_HH_ data analysis.^25^

**Figure 2:**
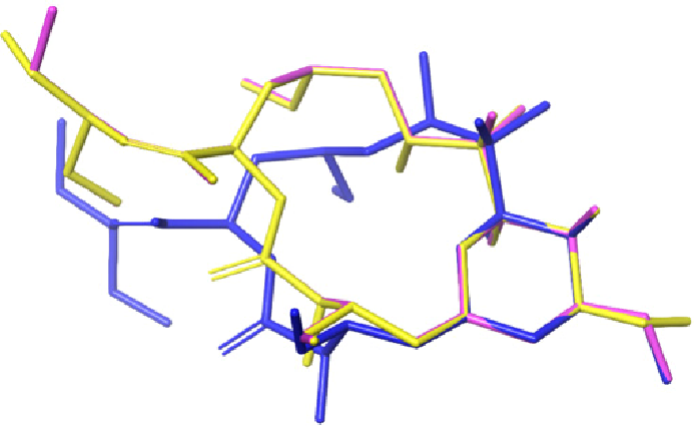
Structures of conformers 1–1 (blue), 1–2 (pink), and 1–5 (yellow) of peloruside A (1).

Okadaic acid (**2**) and its congeners are the primary toxins underlying diarrhetic shellfish poisoning (DSP).^22,26,27^ Its intricate structure, encompassing the 3D conformation in solution, was determined through NMR spectroscopic studies by incorporating long-range heteronuclear coupling constant measurements. Such structure closely aligns with crystallographic results.^28,29^ Notably, despite the molecule’s potential flexibility, an intramolecular hydrogen bond stabilizes its structure, rendering it an ideal candidate for our investigation into utilizing chemical shifts for conformational analysis. Conformers obtained following the same procedure used for **1** showed considerable variation, particularly in the flexible C26–C38 moiety, indicating that a bigger challenge was faced. According to calculated δ_H_ and δ_C_ NMR values, conformer 2–1 gave the best fitting for ^1^H-CMAE while ^13^C-CMAE gave a best match for conformer 2–2 (Figure 3). In accordance, H-DP4+ pointed to 2–1 with 100% probability, while C-DP4+ gave 96.67% probability for 2–2 and 0.27% for 2–1. As a result, the H-C DP4+ probability strongly pointed to conformer 2–1 (99.97% probability). In general, it has been shown that the use of both sets of data (^1^H and ^13^C), provides better results. The apparent ambiguity of the previous results can be explained by the similarity between the structures of conformers 2–1 and 2–2, and the resemblances of these with the solid-state structure of okadaic acid (2) (RMSD = 0.60 Å for 2–1 and RMSD = 0.99 Å for 2–2) (Figure 4). Therefore, we concluded that again the molecular conformation of **2** in solution could be successfully obtained using only NMR chemical shift data. Boltzmann populations derived from density functional theory (DFT) energies indicated a preference for 2–1 with 88.09%. Notably, 2–2 showed a population of 0%, despite its structural similarity, whereas the second most populated conformation, 2–12 (3.32%), exhibited greater structural divergence from the crystallographic structure.

**Figure 3:**
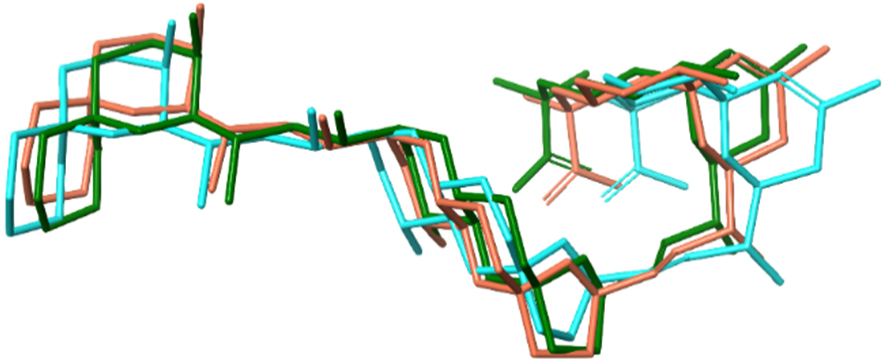
Crystallographic structure of okadaic acid (green) superimposed with conformers 2–1 (pink) and 2–2 (light blue).

Building on the success of studying single conformation molecules, we explored the viability of utilizing chemical shifts in analyzing complex molecules undergoing conformational exchange. Euphodendroidins K (**3**) and L (**4**) that belong to a series of macrocyclic diterpenoids of the jatrophane type were selected.^23^ Their NMR spectroscopic data at 227 K in CDCl_3_ revealed signal duplication due to a slow 1:1 equilibrium between the so-called *endo* and *exo* conformations (Figure 4). Both have been extensively characterized by using NMR and X-ray analysis. For compound **3**, 16 DFT optimized conformations were found and grouped into two homogeneous clusters (merge distance = 0.25 Å), containing 6 *exo* and 10 *endo* structures, respectively (Figure 4). The *exo* cluster comprised conformers 3–3, 3–5, 3–7, 3–8, 3–12, and 3–15. Using these candidate structures, the calculated chemical shifts were correlated with the experimental NMR data. According to our CMAE, CMaxErr, and DP4+ probability analysis, the *exo* and *endo* conformations could be clearly differentiated just by using δ_H_ and δ_C_ NMR data (see Tables S20 and S21). When compared with experimental NMR data of the *exo* conformation, 3–7 yielded 97% DP4+ probability, despite it accounting for only 1.78% of the Boltzmann distribution. For the *endo* conformation, conformers 3–4 and 3–6 yielded DP4+ probabilities of 40% and 58%, respectively, while accounting for 22% and 3% of the Boltzmann population. Thereby, statistical metrics associated with NMR chemical shift calculations allowed for precise differentiation between the two very similar *exo*- and *endo*-type arrangements of **3**. Moreover, the dihedral angle geometry at H4–C4–C5–H5, which is used as a good criterion to differentiate between the *exo* (^3^J_H4/H5_ < 2 Hz) and *endo* (^3^J_H4/H5_ > 8.5 Hz) conformations in the jatrophane backbone, confirmed that the calculated conformations for **3** were consistent with those previously described (RMSD = 0.84 Å for 3–4, RMSD = 0.79 Å for 3–6, and RMSD = 0.32 Å for 3–7) related to *endo* and *exo* conformation.^23^ Analogous outcomes were obtained by doing the same analysis for compound **4**. The euphodendroidins analysis demonstrated that computationally assisted interpretation of NMR chemical shifts can effectively differentiate between two similar conformations of the same molecule, highlighting the efficacy of this approach in conformational analysis.

**Figure 4:**
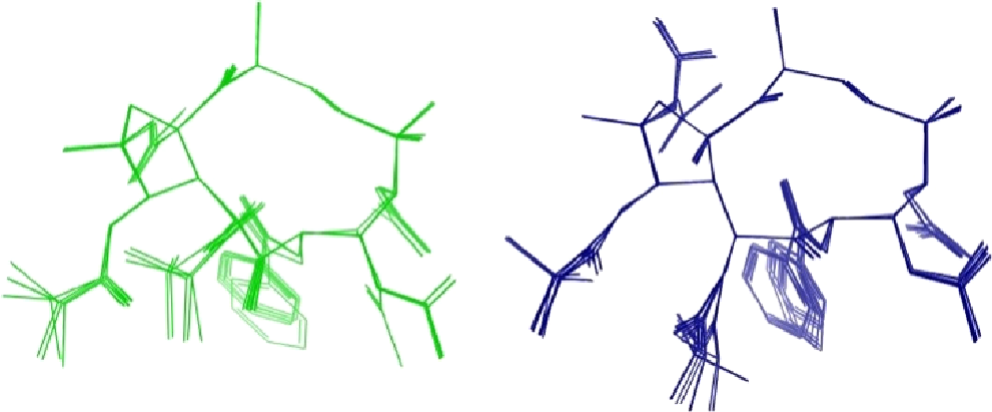
Calculated conformers for the euphodendroidin K (**3**): *exo* cluster (left) and *endo* cluster (right).

As a final case study, we challenged the analysis of a molecule with a complex behavior in solution.^30^ (+)-Longilene peroxide (**5**) belongs to a large group of polyether compounds derived from squalene, demonstrating diverse biological activities as protein phosphatase 2A inhibition, integrin antagonism, and cytotoxicity.^24^ The structure of its enantiomer was determined on the basis of X-ray crystallography and total synthesis.^31^ According to the crystallographic data available, **5** adopts a folded conformation, reminiscent of the letter C, stabilized by intramolecular hydrogen-bond interactions (see Figure 5).^32^ Nevertheless, the conformational analysis of **5** in solution by NMR proved to be highly challenging. This difficulty arises from its quasi-symmetric structure featuring isochronous chemical shifts at the pairwise positions on both sides of the middle oxolane ring, along with minor differences in the chemical shifts of the acyclic side chains C1–C7 and C18–C24 (see Table S8). Additionally, the presence of four nonprotonated stereogenic centers (C6, C10, C15, and C18) further complicates the analysis, introducing ambiguity that hampers the measurement and interpretation of ^3^J_HH_ and NOE data in conformational analysis. This makes it a definitive example to test the potential utility of computational-assisted interpretation of chemical shifts.

**Figure 5:**
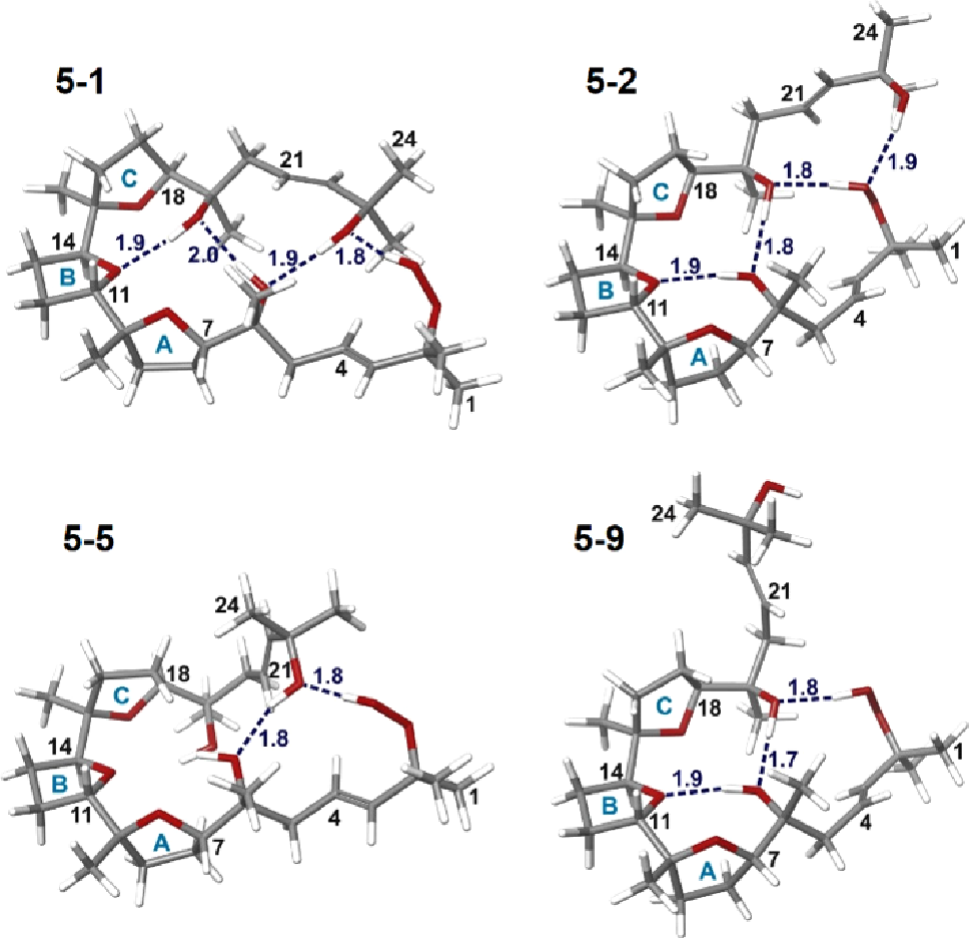
Selected conformers from clusters I–IV. The root-mean-square deviation (RMSD) of 5–1 versus the crystallographic structure is 0.0175.

The conformational search of **5** resulted in 9 conformers, using an atomic RMSD threshold of 1.0 Å, which can be grouped into four structural clusters. Cluster I (conf. 5–1, 5–4, and 5–6), II (conf. 5–2 and 5–3), III (conf. 5–5), and IV (conf. 5–7, 5–8, and 5–9). As in the previous cases, their NMR chemical shifts and coupling constants (^3^J_HH_) were computed using the mPW1PW91/6–31+G** level of theory, and DP4+ probabilities were calculated. ^1^H-DP4+ pointed to conformer 5–2 (86.8%), whereas ^13^C data pointed to conformer 5–1 (>99%). However, the overall DP4+ probability (using both ^1^H and ^13^C) clearly pointed to an answer concordant with the solid-state structure; this is conformer 5–1 as the selected conformation (>99.9%), showing an RMSD value of 0.0175 Å, compared to that for the crystallographic structure. Comparable results were obtained under the CMAE and CMaxErr analyses (Table S24). Despite this apparently good result, it is important to note that several discrepancies between the X-ray structure and the NMR data of **5** recorded in CDCl_3_ solution can be observed. First, the ^3^J_HH_ values observed for methylene protons at C5 and C20 (^3^J_HH_ = 7 Hz in all of them) are far from those predicted from the crystallographic structure, suggesting conformational flexibility at these positions. Additionally, the ROESY spectrum showed a pair of intriguing correlations between H18 and the olefinic protons H21 and H22 that cannot be explained by the crystallographic structure. Moreover, the expected corresponding correlations between H7 and H3 or H4 in the other “arm” of the molecule could not be found, suggesting conformational differences at both sides of the molecule. Thus, NMR experiments at varied temperatures (from 22 °C to 37 °C) were performed to investigate the possibility of hydrogen-bonding interactions, as expected from the crystallographic structure.^33^ Two general trends were observed: small temperature coefficients (Δδ/Δ*T* = 4.0 and 2.0 ppb/K) for C6 and C19 hydroxy protons, respectively, while the C23 hydroxy and the hydroperoxide hydrogens clearly displayed wider variation (Δδ/Δ*T* = 12.7 and 10.7 ppb/K). These data suggest that the C6 and C19 hydroxy hydrogens participate in stronger intramolecular hydrogen bonds than the C2 and C23 exchangeable protons.

Considering the aforementioned discrepancies, the possibility of a fast conformational equilibrium was explored. The impact of improperly calculating the energy landscape of flexible molecules, particularly those with a network of intramolecular hydrogen bonds, on DP4+ has recently been addressed. This was done by moving beyond traditional DFT energies and employing multiensemble strategies for the structural identification of different diastereoisomers.^34^ In this study, we further this approach by selecting appropriate conformations from computational calculations, not solely based on calculated energies, but also by utilizing their NMR chemical shifts as a criterion. NMR data interpretation was conducted by taking a representative structure for each conformational cluster and combining them with varying relative molar fraction ratios at steps of 0.1. This approach, which uses the DP4+ metric, revealed a more satisfactory fit for several combinations of conformers than for any individual conformation. In our opinion, the DP4+ analysis yielded more coherent results, indicating that the most probable situation (accounting for 82.5% of probability) fell within a relatively narrow band, of molar ratio combinations between 0.6:0.3:0.1:0 and 0.5:0.3:0:0.2 (of 5–1:5–2:5–5:5–9), as illustrated in Figure 6. The highest probabilities were associated with the 0.5:0.3:0.1:0.1 mixtures (17.1%) and 0.6:0.2:0:0.2 mixtures (12.2%). Interpreting these findings, our DP4+ analysis points to a limited range of combinations, with the highest probabilities observed in combinations predominantly populated by conformers 5–1 in equilibrium with conformers 5–2, along with minor proportions of conformers 5–5 and 5–9 (Figure 6). Likewise, for ^1^H data, the best CMAE (0.068 ppm) was obtained for 0.5:0.3:0:0.2 combination of the conformers 1:2:5:9, with a CMaxErr of 0.21 ppm. For ^13^C data, the best CMAE and CMaxErr values (1.17 and 2.61 ppm, respectively) were obtained for a 0.5:0.3:0.2:0 mixture. These results are better than those recorded for a single conformer (^13^C CMAE = 1.43 and 1.67 for the conformers 5–1 and 5–2; ^1^H CMAE = 0.10 and 0.11 for 5–1 and 5–2; ^13^C CMaxErr = 3.66 and 4.64 for 5–1 and 5–2; and ^1^H CMaxErr = 0.31 for the two conformers 5–1 and 5–2) (see Table S25).

**Figure 6:**
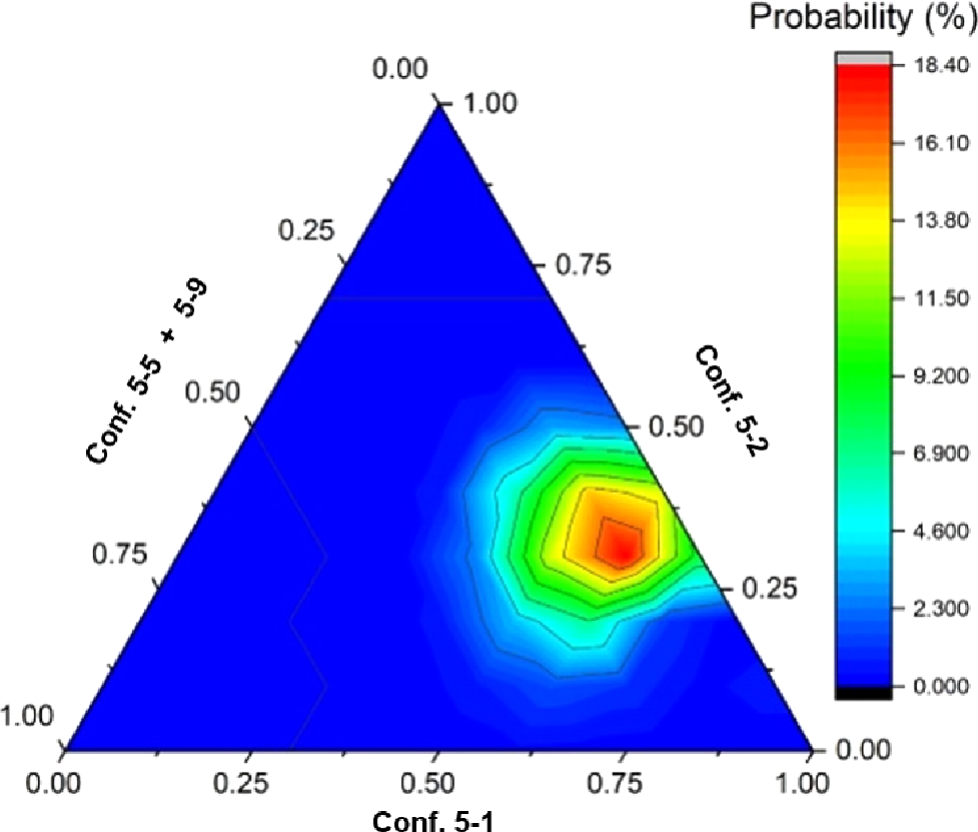
Ternary plot of conformers 5–1, 5–2, 5–5, and 5–9. The fraction of conformers 5–5 and 5–9 is represented as their sum on one of the axes, as they are minor components in the most likely combinations. The DP4+ probability for each mixture is color-coded. The sum of all probabilities accounts for 100%. The highest probability value in the graph is located at a ratio of 0.5:0.3:0.1:0.1.

To check the previous findings, we compared the suggested equilibrium based on the computationally assisted interpretation of NMR chemical shifts against the NOE and ^3^J_HH_ data available. It was satisfying to find that the experimentally measured values fit better with the equilibria suggested by the computational analysis than with the static crystallographic structure. Indeed, the lowest deviation between ^3^J_HH_ calculated vs ^3^J_HH_ experimental was obtained for a 0.6:0.3:0:0.1 mixture (RMSD = 0.07 Hz), far better than any found for a single conformer (RMSD = 0.65, 0.31, 0.81 and 0.59 Hz for the selected conformers 5–1, 5–2, 5–5, and 5–9, respectively). With regard to the NOE data, the inclusion of conformers 5–9 in the equilibrium, as suggested by the DP4+ analysis, explains the already mentioned dipolar correlations between H18 and the olefinic protons H21 and H22 (Figure 6). Additionally, the proposed equilibrium is compatible with the measured temperature coefficients of the hydroxy and hydroperoxide protons. According to these values, the C6 and C19 hydroxy protons are involved most of the time in hydrogen bonding, as opposed to the exchangeable protons at C2 and C23. This would be the case in an equilibrium where conformers 5–1 and 5–2 are preponderant and conformer 5–9 is marginal, as obtained in our analysis. Thus, the distant location of C23-OH, in conformer 5–9, could explain its slightly higher Δδ/Δ*T* value, compared to the hydroperoxide hydrogen.

In conclusion, this study demonstrates that utilizing NMR chemical shift data can significantly enhance conformational analysis. Allocating relative populations within an ensemble of conformers based on molecular mechanics or even DFT calculations often yields uncertain results, due to the limitations of accurately predicting energy distributions.^35^ Consequently, conformational analysis has been supplemented with geometrical restraints, such as coupling constants or NOE data from NMR experiments, which can be challenging to obtain and interpret. This proof-of-concept highlights the potential of incorporating chemical shift information decoded with quantum chemical calculations to provide a more robust framework for conformational analysis. However, it is important to acknowledge the limitations of this study. The method may not be fully applicable to very large structures or systems with intricate conformational equilibria. Additionally, the use of hydrogen-bonding solvents could introduce further complications that were not addressed in this study. Future research should aim to expand this approach to include more-complex systems.

## Data Availability

The underlying data for this study are available in the published article and its Supporting Information.

## Supporting Information Available

NMR experimental, computationally calculated data, Cartesian coordinates, and data analysis results (PDF)
