# Supplementary material for: Computationally Assisted Analysis of NMR Chemical Shifts as a Tool in Conformational Analysis
Source: Org Lett. 2024 Jun 18;26(31):6529–34. doi: 10.1021/acs.orglett.4c01642 (PMC11320654; doi:10.1021/acs.orglett.4c01642)
Supplement: Supplementary file 1 — ol4c01642_si_001.pdf [file ol4c01642_si_001.pdf]

Supporting information

Computationally Assisted Analysis of NMR Chemical Shifts as a Tool in Conformational Analysis.

Cristina Cuadrado[a], Francisco Cen-Pacheco[b,]∗, Antonio Hernández Daranas[a,]∗

[a] *Instituto de Productos Naturales y Agrobiología del CSIC (IPNA-CSIC), La Laguna, 38206, Tenerife, Spain*
[b] *Faculty of Bioanalysis, Iturbide s/n, Veracruz University, 91700, Veracruz, Veracruz, México*

∗ Corresponding author. Francisco Cen-Pacheco
E-mail address: fcen@uv.mx
∗ Corresponding author. Antonio Hernández Daranas
E-mail address: adaranas@ipna.csic.es

List of contents

1. Computational methods..3
2. Experimental data for compounds 1, 2, 3, 4 and 5..4
3. Isotropic magnetic shielding of compounds 1-5. 16
4. Combination of conformations for compound 5. 41
5. CMAE, Max Error and DP4+ values for compounds 1-5...87
6. Cartesian coordinates for compounds 1-5. 99

1. Computational methods.

Conformational searches were performed using the Macromodel software and the MMFF94 force field as included in the Schrodinger 2016-4 suite (Schrödinger Inc., San Diego, CA, USA). Solvation effects were simulated using the generalized Born/surface area (GBSA) solvation model with chloroform. Extended non-bonded cutoff distances (van der Waals cutoff of 8.0 Å and an electrostatic cutoff of 20.0 Å) were used. An energy cutoff of 12 kJ/mole and a geometric RMSD > 1Å from the lowest energy was chosen as a criterium to select contributing conformers. Analysis of the results was undertaken using the Maestro software. Selected conformers were used for full geometry optimization at the B3LYP/6-31G* level of theory in the gas phase. Frequency calculations were done at the same level to determine the nature of the stationary points found. All the B3LYP/6-31G* optimized geometries (after removing duplicates) were next subjected to NMR calculations. Magnetic shielding constants (σ) were computed using the GIAO method at the PCM/mPW1PW91/6-31+G** level of theory using the polarizable continuum model (PCM) with chloroform as solvent, as recommended for DP4+ calculations. Quantum mechanical calculations were carried out with the Gaussian software.[1] Scaled and unscaled chemical shifts were computed using TMS as reference according to the procedure described for DP4+ calculations. The DP4+ probability was calculated using an Excel spreadsheet available as part of the Supporting Information of the original DP4+ paper.[2] The scaled chemical shifts used to calculated the corrected mean average error (CMAE=$\Sigma_n|\delta_{scaled} - \delta_{exp}|/n$) and the corrected maximum error (CMaxErr=max$|\delta_{scaled} - \delta_{exp}|$) were computed as δ_s = (intercept - σ)/(-slope), resulting from a linear regression calculation on a plot of σ against δ_{exp}. The $^3J_{HH}$ values were computed calculating just the Fermi contact terms. This procedure has shown better results than those obtained considering all terms.[3] A correction factor of 0.912 was used to obtain values for comparison with experimental data.

(1) Frisch, M. J.; Trucks, G. W.; Schlegel, H. B.; Scuseria, G. E.; Robb, M. A.; Cheeseman, J. R.; Scalmani, G.; Barone, V.; Mennucci, B.; Petersson, G. A.; Nakatsuji, H.; Caricato, M.; Li, X.; Hratchian, H. P.; Izmaylov, A. F.; Bloino, J.; Zheng, G.; Sonnenberg, J. L.; Hada, M.; Ehara, M.; Toyota, K.; Fukuda, R.; Hasegawa, J.; Ishida, M.; Nakajima, T.; Honda, Y.; Kitao, O.; Nakai, H.; Vreven, T.; Montgomery, J. A., Jr., Peralta, J. E.; Ogliaro, F.; Bearpark, M.; Heyd, J. J.; Brothers, E.; Kudin, K. N.; Staroverov, V. N.; Kobayashi, R.; Normand, J.; Raghavachari, K.; Rendell, A.; Burant, J. C.; Iyengar, S. S.; Tomasi, J.; Cossi, M.; Rega, N.; Millam, J. M.; Klene, M.; Knox, J. E.; Cross, J. B.; Bakken, V.; Adamo, C.; Jaramillo, J.; Gomperts, R.; Stratmann, R. E.; Yazyev, O.; Austin, A. J.; Cammi, R.; Pomelli, C.; Ochterski, J. W.; Martin, R. L.; Morokuma, K.; Zakrzewski, V. G.; Voth, G. A.; Salvador, P.; Dannenberg, J. J.; Dapprich, S.; Daniels, A. D.; Farkas, O.; Foresman, J. B.; Ortiz, J. V.; Cioslowski, J.; Fox, D. J. Gaussian 09, Gaussian, Inc.: Wallingford, CT, 2009.

(2) N. Grimblat, M. M. Zanardi, A. M. Sarotti, J. Org. Chem. 2015, 80, 12526.

(3) Bally, T.; Rable, P. R. *J. Org. Chem.* **2011**, 76, 4818-4830.

2. Experimental data for compounds 1, 2, 3, 4 and 5.

Table S1. NMR data for peloruside (**1**) (500 MHz, $CDCl_3$).

**Atom**	δ_C	**Atom**	δ_H
**C10**	43.60	**H8**	4.02
**C9**	101.90	**H11**	4.89
**C8**	66.80	**H7**	3.82
**C11**	73.90	**H5**	4.25
**C7**	75.90	**H6a**	1.53
**C5**	63.50	**H6b**	1.78
**C6**	31.70	**H12a**	1.4
**C21**	15.80	**H12b**	2.07
**C22**	20.80	**H4a**	1.78
**C12**	33.90	**H4b**	2.13
**C4**	32.60	**H13**	3.99
**C13**	77.90	**H3**	4.22
**C3**	78.30	**H14a**	2.02
**C14**	35.70	**H14b**	2.15
**C2**	70.30	**H2**	4.53
**C15**	70.90	**H15**	5.68
**7-OCH3**	55.70	**H17**	6.75
**C1**	174.00	**H18**	5.05
**C16**	136.10	**H19a**	2.61
**13-OCH3**	59.10	**H19b**	1.17
**3-OCH3**	56.10	**H24a**	1.44
**C17**	131.10	**H24b**	3.36
**C23**	17.50	**$H_3$21**	3.64
**C18**	43.30	**$H_3$22**	1.08
**C19**	24.60	**7-OCH_3**	1.12
**C24**	66.90	**13-OCH_3**	3.31
**C20**	12.20	**3-OCH_3**	3.48
		**$H_3$23**	3.31
		**$H_3$20**	1.67
		**H8**	0.85

Table S2. Cartesian coordinates for Rx of the okadaic acid (**2**).

**Atom**	**X**	**Y**	**Z**
**C**	-2.37836	-1.712622	-2.914971
**C**	-2.216848	-2.930883	-2.019705
**C**	-1.988569	-2.541569	-0.573459
**C**	-3.169343	-1.814293	0.14668
**C**	-2.907208	-1.44556	1.528093

**C**	-4.129125	-0.791976	2.158519
**C**	-5.30189	-1.810725	2.018031
**C**	-5.59303	-2.191101	0.607877
**C**	-6.618007	-3.190522	0.366324
**C**	-7.657263	-3.050059	-0.467485
**C**	-7.812446	-1.77525	-1.282937
**C**	-6.467943	-0.994889	-1.355578
**C**	-6.631083	0.434344	-1.85735
**C**	-5.312407	1.114581	-1.849411
**C**	-5.024369	2.183296	-1.255317
**C**	-3.697167	2.920594	-1.158405
**C**	-3.622096	4.434258	-1.311366
**C**	-2.460128	4.897202	-0.377679
**C**	-1.973622	3.515913	0.259862
**C**	-1.577623	3.680998	1.672081
**C**	-1.088857	2.283992	2.251117
**C**	0.054951	1.794847	1.331213
**C**	-0.453445	1.730772	-0.076829
**C**	0.726765	1.218402	-0.964321
**C**	1.230527	-0.196673	-0.425317
**C**	1.558047	-0.108762	1.016715
**C**	2.885372	0.583338	1.46318
**C**	4.108118	-0.115066	0.798164
**C**	5.493011	0.512018	1.141441
**C**	6.602697	-0.369287	0.485301
**C**	6.614387	-1.835488	0.956816
**C**	7.803869	-2.510436	0.269977
**C**	9.145082	-1.767604	0.549243
**C**	9.055428	-0.309979	0.256851
**C**	10.20455	0.537033	0.778891
**C**	10.239848	1.948543	0.126345
**C**	10.115435	1.975722	-1.377526
**C**	8.962358	1.079623	-1.82611
**C**	6.721369	-2.03197	2.4976
**C**	5.577299	1.924086	0.59545
**C**	1.278791	-1.186041	-1.190579
**C**	-7.2512	0.512128	-3.219995
**C**	-8.708858	-4.113962	-0.681136
**C**	-0.97867	-3.665697	-2.623612
**O**	-3.535017	-1.688581	-3.623807
**O**	-1.596708	-0.782212	-3.050141

**O**	-3.269988	-3.813852	-2.214183
**O**	-4.335011	-2.722732	-0.004742
**O**	-6.507462	-1.256372	2.625854
**O**	-5.857115	-0.958901	-0.093251
**O**	-3.126594	2.649039	0.098483
**O**	-0.887857	2.999637	-0.564901
**O**	0.475697	0.52675	1.816517
**O**	0.35136	1.162404	-2.326562
**O**	3.055024	0.578438	2.815647
**O**	7.875435	0.275252	0.798619
**O**	9.026192	-0.250693	-1.19753
**HO**	-3.157302	-4.57844	-1.644168
**HO**	1.091632	0.845786	-2.849793
**HO**	3.882121	1.013555	3.036161
**HO**	-7.228414	-1.884324	2.539135

Table S3. NMR data for okadaic acid (**2**) (600 MHz, $CDCl_3$).

**Atom**	δ_C	**Atom**	δ_H
**C1**	176.7	**H22**	3.57
**C2**	77.2	**H30**	3.25
**C3**	42.6	**H4**	3.96
**C4**	69.3	**H12**	3.35
**C44**	27.3	**H16**	4.51
**C5**	31.6	**H23**	3.35
**C6**	32.8	**H26**	3.90
**C7**	71.5	**H3a**	1.62
**C8**	96.5	**H3b**	2.12
**C12**	71.5	**CH_3-2**	1.36
**C11**	33.2	**H5a**	1.31
**C10**	139.4	**H5b**	1.72
**C9**	121.5	**H6a**	1.79
**C13**	42.2	**H6b**	1.83
**C14**	136.3	**H7**	3.34
**C42**	15.9	**H11a**	1.87
**C15**	131.4	**H11b**	1.91
**C16**	79.1	**H9**	5.29
**C19**	105.7	**H13**	2.21
**C18**	37.3	**H14**	5.63
**C17**	30.6	**CH_3-13**	0.97
**C20**	26.4	**H15**	5.42
**C21**	27.1	**H18a**	1.80

**C22**	69.7	**H18b**	2.04
**C23**	76.5	**H17a**	1.54
**C26**	84.9	**H17b**	2.14
**C25**	144.7	**H20a**	1.32
**C24**	71.1	**H20b**	1.47
**C27**	64.7	**H21a**	1.72
**C28**	35.3	**H21b**	1.81
**C29**	31.1	**H24**	4.07
**C30**	75.0	**H27**	4.04
**C40**	16.2	**H28a**	0.95
**C34**	95.6	**H28b**	1.28
**C33**	30.3	**H29**	1.91
**C32**	26.6	**C$\underline{H_3}$-29**	1.01
**C31**	27.4	**H33a**	1.34
**C35**	25.4	**H33b**	1.52
**C36**	35.9	**H32a**	1.86
**C37**	18.7	**H32b**	1.96
**C38**	60.3	**H31**	1.75
**C39**	10.7	**H35a**	1.31
**C43**	23.1	**H35b**	1.48
**C41**	112.5	**H36a**	1.39
		**H36b**	1.61
		**H37a**	1.51
		**H37b**	1.84
		**H38a**	3.53
		**H38b**	3.62
		**C$\underline{H_3}$-31**	0.88
		**C$\underline{H_3}$-10**	1.73
		**H41a**	5.02
		**H41b**	5.39

Table S4. NMR data of the *exo*-conformation of euphodendroid K (**3**) (227 K, 600 MHz, $CDCl_3$).

**Atom**	δ_C	**Atom**	δ_H
**C1**	50.5	**H1a**	2.83
**C2**	87.6	**H1b**	2.03
**C3**	77.5	**H3**	5.71
**C4**	47.4	**H4**	3.43
**C5**	67.1	**H5**	5.27
**C6**	143.9	**H7**	5.65
**C7**	68.0	**H8**	5.02
**C8**	70.2	**H9**	4.93
**C9**	80.0	**H11**	6.00
**C10**	40.5	**H12**	5.46
**C11**	137.4	**H13**	3.85
**C12**	129.2	**H16**	1.50
**C13**	44.0	**H17a**	5.09

**C14**	212.0	**H17b**	4.86
**C15**	88.9	**H18**	0.86
**C16**	18.7	**H19**	1.26
**C17**	111.8	**H20**	1.23
**C18**	26.1	**H22**	2.32
**C19**	23.1	**H24**	2.58
**C20**	20.2	**H25**	1.11
**C21**	171.0	**H26**	1.09
**C22**	22.8	**H28**	2.46
**C23**	176.2	**H29**	1.08
**C24**	34.1	**H30**	1.01
**C25**	18.7	**H33**	8.03
**C26**	18.4	**H34**	7.44
**C27**	175.6	**H35**	7.58
**C28**	33.8	**H36**	7.44
**C29**	18.6	**H37**	8.03
**C30**	19.2	**H39**	2.07
**C31**	164.5	**H41**	1.47
**C32**	130.3	**H1a**	2.83
**C33**	128.8	**H1b**	2.03
**C34**	133.9	**H3**	5.71
**C35**	128.8	**H4**	3.43
**C36**	130.3	**H5**	5.27
**C37**	170.4	**H7**	5.65
**C38**	21.0	**H8**	5.02
**C39**	170.4	**H9**	4.93
**C40**	20.7	**H11**	6.00
		**H12**	5.46
		**H13**	3.85
		**H16**	1.50
		**H17a**	5.09
		**H17b**	4.86
		**H18**	0.86
		**H19**	1.26
		**H20**	1.23
		**H22**	2.32
		**H24**	2.58
		**H25**	1.11
		**H26**	1.09
		**H28**	2.46
		**H29**	1.08
		**H30**	1.01
		**H33**	8.03
		**H34**	7.44
		**H35**	7.58
		**H36**	7.44
		**H37**	8.03

		**H39**	2.07
		**H41**	1.47

Table S5. NMR data of the *endo*-conformation of euphodendroid K (**3**) (227 K, 600 MHz, $CDCl_3$).

**Atom**	δ_C	**Atom**	δ_H
**C1**	50.0	**H1a**	3.02
**C2**	87.9	**H1b**	2.07
**C3**	78.2	**H3**	5.58
**C4**	46.4	**H4**	3.83
**C5**	72.9	**H5**	5.79
**C6**	136.8	**H7**	6.39
**C7**	66.2	**H8**	5.04
**C8**	69.5	**H9**	4.84
**C9**	81.4	**H11**	5.83
**C10**	40.0	**H12**	5.46
**C11**	135.9	**H13**	3.68
**C12**	129.0	**H16**	1.50
**C13**	43.6	**H17a**	4.97
**C14**	213.6	**H17b**	5.43
**C15**	84.0	**H18**	0.83
**C16**	20.7	**H19**	1.21
**C17**	123.8	**H20**	1.29
**C18**	24.8	**H22**	2.25
**C19**	23.6	**H24**	2.46
**C20**	20.5	**H25**	1.16
**C21**	170.7	**H26**	1.14
**C22**	22.8	**H28**	1.37
**C23**	175.8	**H29**	0.53
**C24**	33.9	**H30**	0.54
**C25**	19.6	**H33**	8.03
**C26**	18.9	**H34**	7.40
**C27**	175.5	**H35**	7.54
**C28**	33.3	**H36**	7.40
**C29**	18.5	**H37**	8.03
**C30**	18.1	**H39**	2.14
**C31**	165.3	**H41**	1.28
**C32**	129.6	**H1a**	3.02
**C33**	128.7	**H1b**	2.07
**C34**	133.6	**H3**	5.58
**C35**	128.7	**H4**	3.83
**C36**	129.6	**H5**	5.79
**C37**	170.3	**H7**	6.39
**C38**	21.6	**H8**	5.04
**C39**	170.8	**H9**	4.84

**C40**	22.0	**H11**	5.83
		**H12**	5.46
		**H13**	3.68
		**H16**	1.50
		**H17a**	4.97
		**H17b**	5.43
		**H18**	0.83
		**H19**	1.21
		**H20**	1.29
		**H22**	2.25
		**H24**	2.46
		**H25**	1.16
		**H26**	1.14
		**H28**	1.37
		**H29**	0.53
		**H30**	0.54
		**H33**	8.03
		**H34**	7.40
		**H35**	7.54
		**H36**	7.40
		**H37**	8.03
		**H39**	2.14
		**H41**	1.28

Table S6. NMR data of the *exo*-conformation of euphodendroid L (**4**) (227 K, 600 MHz, $CDCl_3$).

**Atom**	δ_C	**Atom**	δ_H
**C1**	50.4	**H1a**	2.01
**C2**	87.3	**H1b**	2.80
**C3**	77.5	**H3**	5.69
**C4**	47.4	**H4**	3.36
**C5**	67.0	**H5**	5.18
**C6**	143.7	**H7**	5.65
**C7**	67.9	**H8**	4.97
**C8**	70.1	**H9**	4.91
**C9**	79.9	**H11**	5.96
**C10**	40.5	**H12**	5.43
**C11**	136.9	**H13**	3.81
**C12**	128.9	**H16**	1.50
**C13**	43.8	**H17a**	4.87
**C14**	211.9	**H17b**	5.08
**C15**	88.5	**H18**	0.83
**C16**	20.3	**H19**	1.22
**C17**	112.0	**H20**	1.22
**C18**	23.1	**H22**	2.24
**C19**	26.0	**H24**	2.08

**C20**	20.2	**H26**	2.44
**C21**	170.7	**H27**	1.10
**C22**	22.7	**H28**	1.07
**C23**	169.9	**H31**	8.01
**C24**	21.5	**H32**	7.39
**C25**	175.2	**H33**	7.52
**C26**	33.9	**H34**	7.39
**C27**	18.6	**H35**	8.01
**C28**	18.9	**H37**	2.27
**C29**	164.5	**H39**	1.94
**C31**	129.6		
**C32**	128.8		
**C33**	133.7		
**C34**	128.8		
**C35**	129.6		
**C36**	170.5		
**C37**	22.7		
**C38**	170.3		
**C39**	21.4		

Table S7. NMR data of the *endo*-conformation of euphodendroid L (**4**) (227 K, 600 MHz, $CDCl_3$).

**Atom**	δ_C	**Atom**	δ_H
**C1**	50.0	**H1a**	2.07
**C2**	84.2	**H1b**	2.99
**C3**	72.7	**H3**	5.54
**C4**	46.4	**H4**	3.82
**C5**	78.8	**H5**	5.83
**C6**	137.5	**H7**	6.37
**C7**	66.2	**H8**	5.02
**C8**	69.4	**H9**	4.82
**C9**	81.3	**H11**	5.80
**C10**	39.9	**H12**	5.43
**C11**	136.0	**H13**	3.66
**C12**	129.2	**H16**	1.50
**C13**	43.6	**H17a**	4.94
**C14**	213.4	**H17b**	5.39
**C15**	87.9	**H18**	0.84
**C16**	20.5	**H19**	1.18
**C17**	123.6	**H20**	1.27
**C18**	24.8	**H22**	2.10
**C19**	23.5	**H24**	2.05
**C20**	20.6	**H26**	1.36
**C21**	170.7	**H27**	0.53

**C22**	21.0	**H28**	0.49
**C23**	170.3	**H31**	7.55
**C24**	21.2	**H32**	7.42
**C25**	175.9	**H33**	7.42
**C26**	33.3	**H34**	7.42
**C27**	18.6	**H35**	7.55
**C28**	18.3	**H37**	2.05
**C29**	165.3	**H39**	1.43
**C31**	129.1		
**C32**	128.7		
**C33**	133.6		
**C34**	128.7		
**C35**	129.1		
**C36**	170.9		
**C37**	21.8		
**C38**	170.6		
**C39**	20.2		

Table S8. NMR data for (+)-longilene peroxide **5** (500 MHz, $CDCl_3$).

**C**	**δ_C**	**δ_H**	**C**	**δ_C**	**δ_H**
**1**	26.9	1.19	**18**	84.1	3.72
**2**	80.1		**19**	73.8	
**3**	137.0	5.43	**20**	40.8	1.88<br>2.20
**4**	125.8	5.81	**21**	122.2	5.75
**5**	41.3	1.78<br>2.20	**22**	141.2	5.61
**6**	73.9		**23**	70.0	
**7**	85.1	3.72	**24**	29.4	1.27
**8**	25.8	1.89<br>2.06	**25**	24.2	1.37
**9**	29.7	1.49<br>2.00	**26**	24.3	1.20
**10**	85.8		**27**	24.2	1.09
**11**	85.8	4.09	**28**	23.6	1.07
**12**	30.1	1.50<br>2.01	**29**	24.3	1.27
**13**	29.9	1.50<br>2.01	**30**	29.6	1.31
**14**	85.4	4.09	**-OOH**		10.57
**15**	85.4		**-OH-6**		5.24
**16**	29.3	1.46<br>2.03	**-OH-19**		5.03
**17**	25.2	1.89<br>2.03	**-OH-23**		3.29

Table S9. Distances calculated for select conformers of (+)-longilene peroxide (**5**) after energy optimization at the B3LYP/6-31G* level of theory and their corresponding dipolar correlation intensities.

Proton pair	ROE Intensity	Distance (Å) Conformer 5-1	Distance (Å) Conformer 5-2	Distance (Å) Conformer 5-5	Distance (Å) Conformer 5-9
H-11/-OH-6	Weak	4.0	3.2	3.3	3.2
H-11/-OH-19	Weak	2.9	5.0	4.8	5.0
H-14/-OH-6	Weak	5.3	3.2	5.0	3.2
H-14/-OH-19	Weak	3.4	4.6	3.0	4.6
H-18/H-21	Weak	4.7	4.3	4.5	3.9
H-18/H-22	Weak	4.9	5.3	4.2	3.7

Table S10. Chemical shifts of exchangeable protons at different temperatures for (+)-longilene peroxide (**5**). Calculated temperature coefficients are shown in bold.

Temp. (K)	(+)-Longilene peroxide (**5**) (ppm)			
	-OOH	-OH-6	-OH-19	-OH-23
295	10.67	5.30	5.07	3.39
300	10.61	5.28	5.06	3.33
305	10.56	5.26	5.05	3.26
310	10.51	5.24	5.04	3.20
**Δδ/ΔT (ppb/K)**	**10.7**	**4.0**	**2.0**	**12.7**

Figure S1. Chemical shifts (ppm) of hydroxyl and hydroperoxide hydrogens of (+)-longilene peroxide (**5**) as a function of temperature (K).

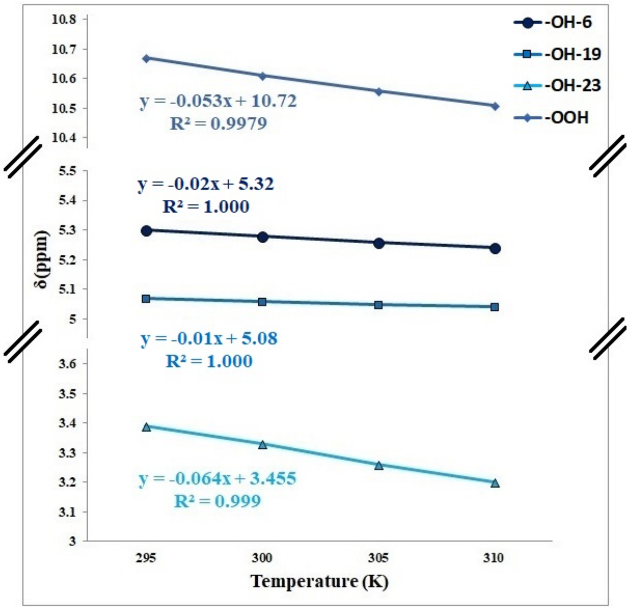

Figure S2. ^{1}H NMR spectra of (+)-longilene peroxide (**5**) recorded at different temperatures (295 – 310 K).

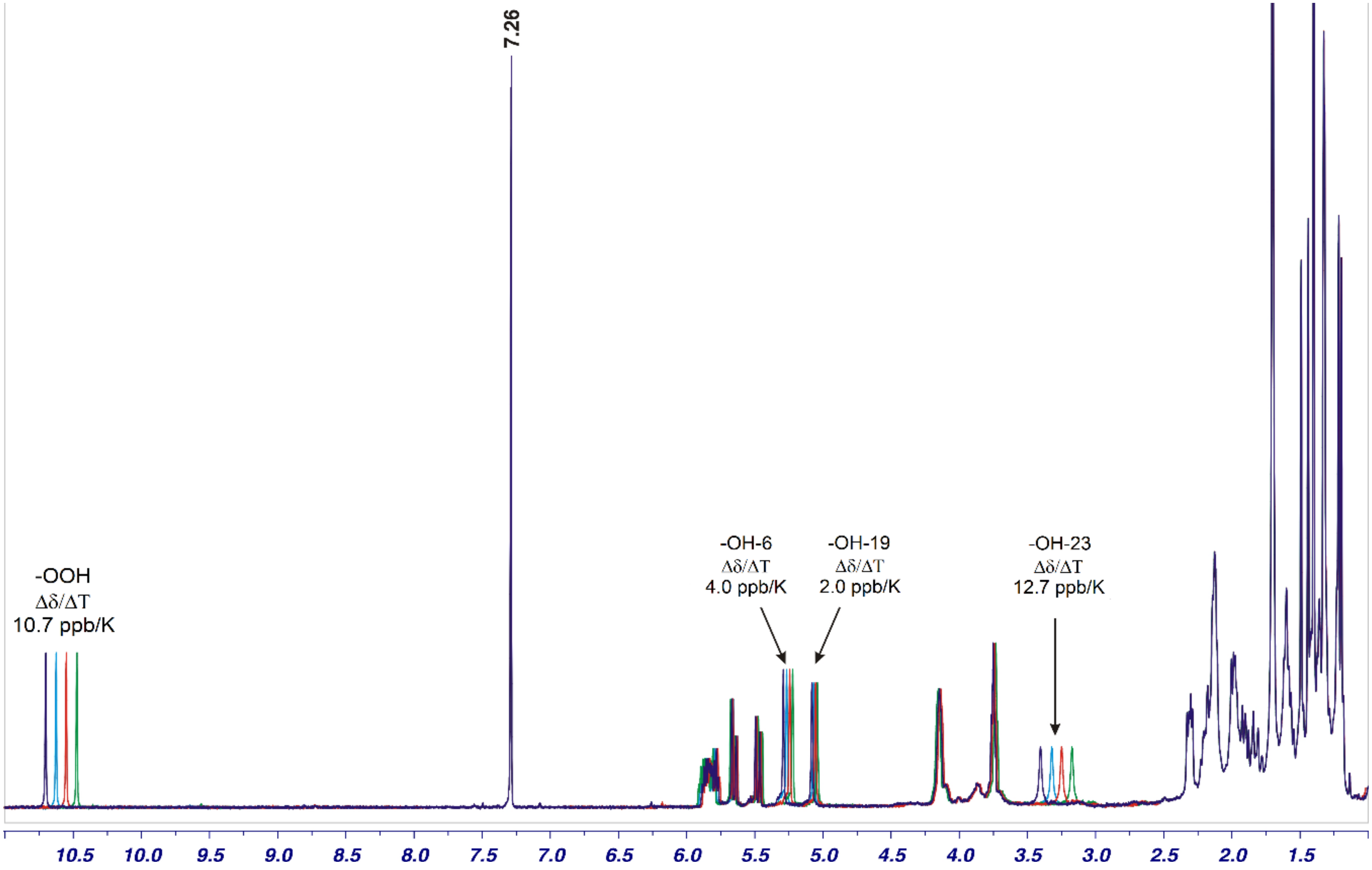

Figure S3. Selected region of the ROESY spectrum of (+)-longilene peroxide (**5**).

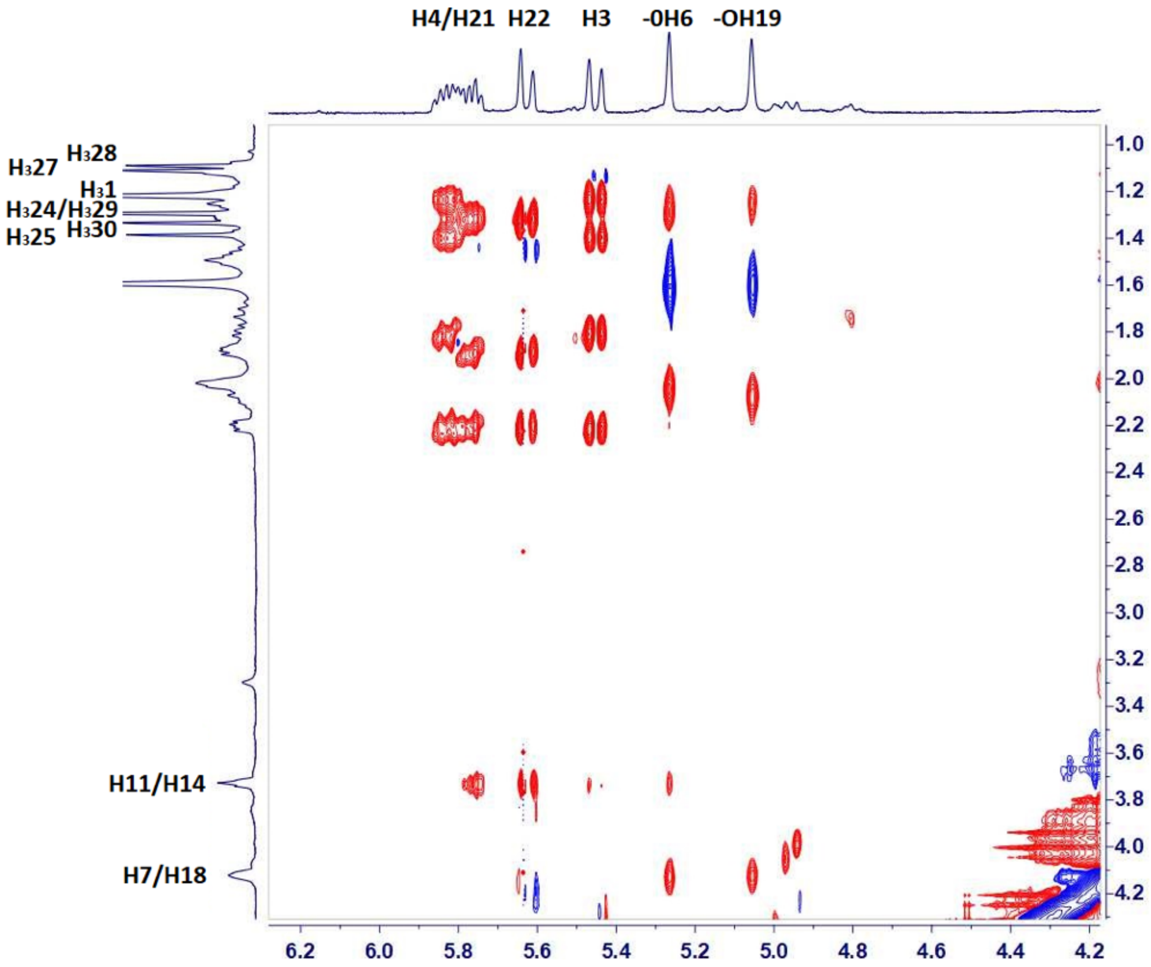

Table S11. Key $^{3}J_{\mathrm{H,H}}$ coupling constants calculated for (+)-longilene peroxide (**5**).

		$^{3}J_{\mathrm{H4,H5}}$	$^{3}J_{\mathrm{H4',H5}}$	$^{3}J_{\mathrm{H20,H21}}$	$^{3}J_{\mathrm{H20',H21}}$
mPW1PW91/6-31+G**	Conformer 5-1	9.7	4.2	9.8	4.4
PCM	Conformer 5-2	5.4	9.0	4.1	9.4
	Conformer 5-5	9.6	4.3	3.9	9.3
	Conformer 5-9	5.4	8.7	3.8	9.3
**Experimental**		**8.5**	**6.6**	**7**	**6.8**

3. Isotropic magnetic shielding of compounds 1-5.

Table S12. Isotropic magnetic shielding values computed for conformers 1-1 to 1-9 of peloruside (**1**) at the PCM/B3LYP/6-31+G** level of theory.

Atom (σ)	1-1	1-2	1-3	1-4	1-5	1-6	1-7	1-8	1-9
**C10**	145.8338	148.6123	146.8344	148.2428	148.8299	145.4695	146.4140	148.8039	149.5642
**C9**	92.8730	91.6672	92.3520	87.6011	92.0018	92.6806	92.4618	89.6575	92.5803
**C8**	123.0664	125.3237	123.6174	124.5539	130.4022	122.4230	131.2465	123.2399	132.0857
**C11**	121.2289	121.3143	122.3540	112.5854	120.0861	123.9017	117.7789	111.8522	122.7184
**C7**	119.1129	119.2666	119.3068	119.5545	117.7766	120.0553	118.9927	119.3417	118.5224
**C5**	129.3250	130.5366	129.4472	128.7435	129.7510	130.3005	128.1566	127.6319	129.9276
**C6**	164.0703	163.8625	164.4249	164.5367	161.8856	164.8110	163.7552	164.5713	161.5978
**C21**	179.4714	175.7083	179.2049	169.0406	174.8681	179.3545	172.0029	169.9082	175.5354
**C22**	174.3489	180.1608	174.5355	172.3187	179.7685	173.8510	171.3824	174.4093	179.2180
**C12**	148.2547	157.7228	151.8696	156.1938	157.6972	152.4392	152.7022	161.6099	157.1603
**C4**	161.0884	162.0069	160.8951	162.3865	162.4313	160.2734	163.2586	162.2140	156.2514
**C13**	117.9926	115.9692	117.4453	114.1193	115.9124	119.1505	119.0865	112.3277	115.7908
**C3**	117.0311	115.3325	117.0687	116.0097	115.3301	116.8845	118.3793	115.8542	114.8424
**C14**	160.9452	158.9543	161.1625	164.3851	158.8873	160.8285	160.9026	157.0291	159.4240
**C2**	123.8598	122.7094	123.4591	123.9491	122.7955	122.4218	117.2420	123.7121	124.7069
**C15**	120.8339	120.6029	120.8513	117.5408	120.8730	122.8500	120.0513	117.8443	118.6286
**7-OCH3**	141.1075	140.6846	141.2364	140.5919	140.9542	141.3088	141.0101	140.7678	141.4058
**C1**	22.0926	22.3126	22.8040	23.4223	22.1852	22.0539	23.9966	23.5419	19.4246
**C16**	56.3341	57.0272	60.2867	58.3879	59.4217	62.2392	57.1035	56.6301	59.9049
**13-OCH3**	138.8220	136.6885	138.9982	140.8293	136.7473	139.1073	139.8273	139.9537	136.2211
**3-OCH3**	140.1440	141.4686	139.8881	140.7781	141.4113	140.0114	140.4997	140.4403	137.3953
**C17**	68.3636	66.6015	66.4470	65.2496	65.6211	61.4862	66.8550	65.8331	63.1088
**C23**	176.3184	175.9432	176.8795	174.5597	176.6819	174.8186	175.8661	175.0870	173.9046
**C18**	149.4714	148.9731	151.8071	149.2257	150.1548	153.4307	149.4274	150.7619	149.0585
**C19**	167.5265	167.6206	164.3299	167.7512	169.0029	170.0264	168.0969	167.7458	166.4902
**C24**	124.6855	124.9587	129.7015	125.7891	128.2857	132.0494	126.3477	126.0882	126.1110
**C20**	182.6153	182.6191	182.1665	181.9707	182.2027	186.2808	182.0444	181.9596	181.4422
**H8**	27.5256	27.6781	27.6405	27.6330	27.3903	27.7685	27.3310	27.8530	27.3822
**H11**	28.0977	26.0447	27.7189	28.5432	26.2386	27.0963	28.4414	28.6701	26.0406
**H7**	27.8186	27.7236	27.8412	27.7418	27.6007	28.2812	27.7144	27.7183	27.7745
**H5**	27.2994	27.2088	27.3264	27.2023	27.2297	27.8338	27.2082	27.0290	27.2873
**H6a**	29.8091	29.7097	29.7696	29.7737	30.0573	29.7494	30.2648	29.7768	30.1725
**H6b**	29.8044	30.0491	29.8438	29.7810	29.9862	29.9287	29.7151	29.7639	30.1106
**H12a**	28.5782	30.3170	28.7103	29.7369	29.5751	29.0423	29.8196	29.4615	30.2721
**H12b**	30.6001	29.5860	30.1811	29.2653	30.3401	30.2399	29.6971	29.3412	29.6320
**H4a**	29.4299	29.3925	29.4288	29.4028	29.4433	29.3329	30.2243	29.3434	29.6869

**H4b**	29.9816	29.8482	29.9593	29.9545	29.8535	30.0249	29.0614	29.9177	29.7449
**H13**	27.7699	27.6574	27.7383	28.2279	27.6846	27.5213	27.6793	28.3944	27.6309
**H3**	27.1301	27.1226	27.1405	27.4275	27.1892	27.2572	27.4824	27.3554	27.4059
**H14a**	30.0452	29.4706	29.7716	28.4370	29.5788	29.5600	29.9228	28.2505	29.3780
**H14b**	29.2235	29.4238	29.3740	29.6310	29.4883	29.4454	29.4513	29.9875	29.4890
**H2**	27.2920	26.9655	27.3182	27.3672	27.0434	27.1891	27.5600	27.1973	26.7526
**H15**	25.6172	25.4115	26.2651	25.3953	25.4048	25.8865	25.6070	25.2475	25.3360
**H17**	26.1628	26.0612	26.0245	26.1409	25.9968	25.6055	26.2264	26.1169	26.0098
**H18**	29.0892	29.0289	29.5066	28.9605	29.1448	28.2575	29.0042	29.0213	29.1049
**H19a**	30.5042	30.4871	29.6230	30.4506	30.3986	30.0301	30.4933	30.4799	30.4089
**H19b**	30.2313	30.2937	30.2039	30.2420	30.4404	30.0101	30.2436	30.2835	30.2696
**H24a**	27.9435	27.8708	28.2152	28.0607	28.0730	27.7915	27.9992	28.0576	27.8944
**H24b**	28.0200	27.9786	27.0980	28.0665	27.7162	27.8029	28.0281	28.0593	28.1234
**$H_3$21**	30.5481	30.3752	30.4447	30.0501	30.4343	30.4066	30.2384	30.0955	30.3675
**$H_3$22**	30.2387	30.4793	30.3699	30.4640	30.3636	30.3633	30.8337	30.5707	30.4652
**7-OC$\underline{H_3}$**	28.0771	28.1033	28.0978	28.0874	28.1554	28.0424	28.0784	28.0543	28.1300
**13-OC$\underline{H_3}$**	28.0134	27.9247	28.1053	27.9859	27.9559	28.1246	28.0939	28.1704	27.9405
**3-OC$\underline{H_3}$**	27.9136	28.0047	28.1350	28.0282	28.3270	27.9429	28.2939	27.9840	28.0690
**$H_3$23**	29.8556	29.9016	29.8215	29.8842	29.9740	29.6703	29.8234	29.8556	29.6878
**$H_3$20**	30.6809	30.7103	30.6869	30.6637	30.6455	30.7463	30.7046	30.6804	30.6736

Table S13. Isotropic magnetic shielding values computed for conformers 2-1 to 2-48 of okadaic acid (**2**) at the PCM/B3LYP/6-31+G** level of theory.

Atom (σ)	2-1	2-2	2-3	2-4	2-5	2-6	2-7	2-8	2-9
**C1**	21.0632	22.5138	20.2418	19.5312	21.0569	22.9685	22.2978	21.0324	19.5295
**C2**	116.0400	116.6756	116.0763	115.5398	115.3803	116.3514	116.5754	115.6854	115.5406
**C3**	154.2186	153.8743	154.0894	154.0860	154.9633	153.0980	154.0737	154.1901	154.0872
**C4**	124.8462	124.7410	124.5159	123.6737	123.4922	124.6511	122.1108	124.2135	123.6728
**C44**	168.5716	167.4816	168.3520	168.4173	169.4798	168.2212	169.5086	167.7713	168.4200
**C5**	162.7248	162.7511	162.4075	162.1425	162.8704	162.8632	161.1274	162.7000	162.1429
**C6**	166.6015	165.8710	165.9715	166.5843	165.5615	165.6382	164.7496	166.0323	166.5845
**C7**	123.2352	125.8309	124.7290	122.6940	123.7807	124.0974	126.5876	123.4234	122.6923
**C8**	97.0184	96.0897	95.4046	94.1481	96.1151	97.7945	101.2213	95.2334	94.1509
**C12**	125.3112	122.9319	123.9895	121.5642	122.9653	122.5001	123.7681	118.7946	121.5659
**C11**	160.4150	161.5382	160.8072	161.8180	161.9899	161.4371	161.2410	161.7271	161.8188
**C10**	55.8394	53.7197	54.8710	54.1440	54.3520	55.7238	53.9129	53.9538	54.1430
**C9**	75.4358	74.2106	75.6667	76.3050	75.8992	74.3946	76.2607	75.5818	76.3010
**C13**	147.5780	149.6436	150.8926	151.0655	150.8606	149.7423	149.9615	154.4601	151.0659
**C14**	58.9789	59.5081	57.7718	56.9402	57.5846	60.4426	68.8678	66.3601	56.9421

**C42**	178.5124	180.5012	180.7075	179.8702	179.8613	180.4956	177.4416	183.1547	179.8711
**C15**	64.8266	64.5957	64.7523	67.9805	68.0702	65.2104	60.9291	69.3605	67.9798
**C16**	115.0804	113.2245	115.4494	113.7230	114.0634	114.0392	116.1942	116.9585	113.7279
**C19**	87.3712	89.3710	89.9718	88.9794	88.8036	88.4030	90.2627	87.7097	88.9814
**C18**	155.5264	157.7907	156.8676	154.4381	154.9993	157.7791	158.5083	154.0620	154.4379
**C17**	162.2715	164.4004	163.6374	160.0543	160.2799	165.0929	165.6258	162.1115	160.0527
**C20**	160.5834	161.3276	162.1491	159.0137	159.4659	162.2019	161.3618	158.7443	159.0148
**C21**	167.5180	167.3037	166.1182	167.0629	165.7930	165.6933	167.2546	166.3775	167.0623
**C22**	125.1011	125.8773	124.9072	125.4443	122.7786	122.8017	125.7605	126.0443	125.4481
**C23**	119.4826	118.1513	120.6630	118.1505	117.4589	118.0776	118.3184	117.3580	118.1496
**C26**	109.7160	109.6605	110.0750	110.7894	112.9018	113.3336	110.0950	109.5978	110.7881
**C25**	47.1224	52.6002	48.6069	53.4885	46.3135	47.0756	55.2656	51.6950	53.4886
**C24**	121.7888	124.1031	123.6941	123.4954	122.5752	123.6295	123.3406	123.4630	123.4981
**C27**	130.1139	131.0371	128.3898	130.5719	116.6965	116.4463	131.2468	131.9653	130.5701
**C28**	160.0720	160.2323	160.6992	161.3682	157.9152	157.3265	159.0835	157.2930	161.3692
**C29**	160.8597	161.6035	158.8550	159.4303	161.4287	162.3477	162.1336	161.0561	159.4310
**C30**	121.7172	121.0427	122.3672	122.2006	118.7290	120.6030	118.1697	121.2838	122.2004
**C40**	181.0440	180.2904	175.9472	177.0799	180.7589	180.0678	179.2569	179.7027	177.0808
**C34**	98.6508	97.7901	98.1240	97.4685	99.3583	97.5519	98.5964	98.8437	97.4669
**C33**	162.7937	162.8948	161.8869	163.1414	163.0883	162.7346	163.5403	162.7420	163.1406
**C32**	166.8040	166.2505	168.9278	168.0696	166.8763	166.3972	167.8451	168.5620	168.0689
**C31**	165.2485	164.7194	163.5116	164.9856	165.1861	165.3347	165.3637	164.2089	164.9868
**C35**	158.4298	158.3854	159.2309	158.6823	158.9235	158.4324	158.3544	159.5048	158.6830
**C36**	173.9379	173.5918	175.3577	174.1499	174.5146	173.2831	174.8173	174.1239	174.1501
**C37**	167.3062	166.8093	168.3301	168.6808	168.3664	168.3790	168.4743	168.3938	168.6807
**C38**	135.1144	134.7116	134.5953	134.3215	134.2652	134.2417	134.5450	134.3645	134.3224
**C39**	184.8612	185.0015	184.4562	184.1808	185.1919	185.2969	184.2730	185.3355	184.1816
**C43**	168.6581	172.1050	174.4576	167.8022	172.0025	170.5133	171.2720	169.4184	167.8015
**C41**	85.1578	85.4158	83.3559	82.9762	88.0755	89.5933	85.7023	82.7222	82.9767
**H22**	27.9735	27.9783	27.8923	27.9040	27.3045	27.3379	27.9213	27.8217	27.0907
**H30**	28.1122	28.0382	27.5911	27.5719	27.9892	28.0502	27.8490	28.0214	27.2609
**H4**	27.4022	27.2352	27.1118	27.2543	27.2543	27.3332	27.3221	27.2165	27.2102
**H12**	27.9799	28.0329	27.9824	27.9410	27.9156	27.8714	27.3668	27.9544	27.5798
**H16**	26.9070	26.6246	26.7262	26.4118	26.4361	26.6985	26.5978	25.8539	26.7485
**H23**	28.0985	28.0188	27.7589	28.0063	28.0310	28.0966	27.9705	28.0153	27.9513
**H26**	27.6844	27.6119	27.7355	27.9290	27.5875	27.5700	27.6644	27.7957	27.3731
**H3a**	29.4415	29.3379	29.3757	28.3082	27.9913	29.4036	29.4502	28.3908	29.3615
**H3b**	30.0248	30.0226	29.9992	29.9688	30.0176	30.0162	30.0317	29.9744	29.9112
**C$\underline{H}_3$-2**	30.2247	30.2287	30.2606	30.1498	30.0823	30.2249	30.2262	30.1176	30.1807
**H5a**	30.2260	30.1392	30.1800	30.1770	30.3122	30.2132	30.0856	30.2115	30.1409
**H5b**	29.7683	29.8661	29.9384	29.9940	29.9847	29.8764	30.3812	29.8709	29.7894

**H6a**	29.7752	29.7754	29.7281	29.8181	29.8294	29.8037	29.4730	29.7913	29.6309
**H6b**	29.8271	29.9875	30.0182	29.7908	29.9590	30.0124	29.7332	29.9053	29.8121
**H7**	28.1226	28.1074	28.1810	28.1818	28.2048	28.2214	28.0091	28.0819	27.9627
**H11a**	29.5979	29.7242	29.7353	29.5631	29.7496	29.6699	29.8412	29.7322	30.2218
**H11b**	29.6418	29.6507	29.4680	29.6339	29.6112	29.5979	29.3599	29.5880	29.3862
**H9**	25.8767	25.9418	25.9693	26.0094	25.9969	25.8554	25.6785	26.1038	26.5324
**H13**	29.5214	29.4426	29.1902	29.3346	29.2853	29.4852	29.2694	29.2079	29.5848
**H14**	25.3220	25.2540	25.3213	25.1942	25.2611	25.1908	25.7033	25.0134	25.2966
**C$\underline{H_3}$-13**	30.5507	30.6257	30.6285	30.6198	30.6919	30.5496	30.1015	30.6019	30.0864
**H15**	25.7895	25.7662	25.5260	25.6366	25.4787	25.8005	25.4305	25.5476	25.7439
**H18a**	29.4120	29.6280	29.6286	29.2158	29.4000	29.5671	29.7028	29.1795	29.8110
**H18b**	29.6671	29.5621	29.7534	29.5839	29.6213	29.6247	29.7806	29.4122	29.8647
**H17a**	29.5435	29.0079	29.3939	29.7643	29.6597	29.0581	29.2162	29.5092	29.1048
**H17b**	29.9412	29.8905	30.0230	30.0934	29.9964	29.8612	29.5974	30.1056	30.0446
**H20a**	29.7305	29.6128	29.7231	29.7415	29.7666	29.7222	29.6349	29.6357	29.8083
**H20b**	29.8051	29.8464	29.8139	29.6921	29.7394	29.8521	29.8994	29.6155	29.5816
**H21a**	29.7300	29.7663	29.8233	29.7873	29.9315	30.0531	29.6509	29.7162	29.8392
**H21b**	29.8222	29.8285	29.8651	29.8115	29.8766	29.8627	29.7925	29.8445	29.7030
**H24**	27.4640	27.2105	27.3367	27.0673	26.6152	26.5089	27.2011	27.0942	26.5874
**H27**	27.3228	27.3297	27.3972	27.3126	27.5692	27.5999	27.4696	27.2488	27.7440
**H28a**	30.4793	30.6600	29.8206	29.7664	30.6244	30.5349	30.8161	30.4548	29.9874
**H28b**	30.0780	30.1476	30.6472	30.5405	29.4185	29.2788	30.3668	30.0936	29.2856
**H29**	29.6849	29.4553	29.7067	29.8081	29.8016	29.9952	29.2447	29.2209	29.5968
**C$\underline{H_3}$-29**	30.4137	30.4444	30.1280	30.2353	30.5121	30.5100	30.5192	30.4404	30.5928
**H33a**	29.9884	29.9898	30.0387	30.0069	29.9258	29.8780	29.9452	30.0023	29.6422
**H33b**	30.3371	30.2814	30.4093	30.3066	30.3042	30.2712	30.2692	30.4288	30.3056
**H32a**	30.2661	30.3107	30.3162	30.3122	30.2736	30.2941	30.2779	30.2338	30.2099
**H32b**	29.5086	29.5301	29.4094	29.4078	29.5128	29.5854	29.4478	29.5105	29.7021
**H31**	29.8202	29.7948	30.1259	30.0728	29.8284	29.7336	29.8047	29.8636	29.6901
**H35a**	30.1270	30.0242	30.0697	30.0621	30.1149	29.9951	29.8885	30.1905	29.9729
**H35b**	30.2337	30.3068	30.2883	30.2987	30.2733	30.3222	30.3997	30.3911	30.3079
**H36a**	29.7421	29.7346	29.5060	29.6103	29.5349	29.6292	29.4878	29.5538	30.1671
**H36b**	30.0713	30.0763	30.1685	30.1385	30.0759	30.0603	30.1407	30.1166	29.6670
**H37a**	30.1227	30.1717	29.9782	29.9907	29.9784	29.9469	30.0115	29.9740	29.9376
**H37b**	30.2825	30.2698	30.1928	30.1842	30.1761	30.2748	30.1593	30.2159	30.3179
**H38a**	28.0498	28.0159	27.9843	28.0153	28.0459	28.0168	28.0754	27.9951	27.9498
**H38b**	27.9508	27.8729	27.5834	27.6728	27.8280	27.7706	27.7769	27.6645	27.7952
**C$\underline{H_3}$-31**	30.5952	30.5341	30.7931	30.7253	30.6101	30.5827	30.5652	30.5940	30.2904
**C$\underline{H_3}$-10**	29.8443	29.6038	29.5871	29.9024	29.8158	29.7515	29.7515	29.8151	29.9617
**H41a**	25.5258	25.7092	25.5263	25.7793	25.9407	25.9562	25.7939	25.4629	25.9491

**H41b**	26.0400	26.1073	26.1666	25.9139	26.1895	26.2697	26.1214	26.0127	26.2824

**Atom (σ)**	**2-10**	**2-11**	**2-12**	**2-13**	**2-14**	**2-15**	**2-16**	**2-17**	**2-18**
**C1**	23.6124	21.4306	22.0038	20.2204	21.4334	23.8057	24.6671	20.5592	22.1721
**C2**	115.9858	115.2664	115.6690	115.7708	115.2671	116.3436	116.8060	115.5715	116.5530
**C3**	153.5461	154.3613	153.9901	155.2806	154.3640	153.7748	153.5872	153.6901	153.4153
**C4**	125.0549	123.5962	124.4297	123.6037	123.5951	125.2061	126.1006	124.8369	123.7716
**C44**	169.1314	169.0223	168.5985	169.5679	169.0237	170.0064	167.6091	167.5227	169.1632
**C5**	165.5935	162.6529	164.3379	163.1183	162.6561	163.8156	162.6915	161.5951	163.3237
**C6**	163.5791	165.4154	166.0664	166.8919	165.4153	163.9017	166.2452	166.3681	163.9691
**C7**	124.4097	123.8952	122.8679	123.1125	123.8982	124.7153	122.9729	123.7134	125.3254
**C8**	98.5853	95.6232	96.3032	96.7342	95.6245	97.4360	94.8283	96.2401	99.8511
**C12**	121.4068	123.7051	119.7735	124.3335	123.6938	123.3982	118.2724	119.5313	123.6350
**C11**	162.7367	161.0368	161.6541	160.8687	161.0372	162.0841	159.6787	159.4232	160.2350
**C10**	58.3472	54.7931	54.7337	53.9517	54.7978	55.2469	54.8485	55.6081	51.9643
**C9**	78.8631	75.3224	75.2563	75.2149	75.3304	74.8747	75.2767	76.0553	75.0884
**C13**	150.1477	150.5137	152.9317	149.2641	150.5203	149.9837	153.7530	154.7056	150.6131
**C14**	69.5396	57.7139	68.4381	57.7484	57.7104	70.4911	67.8740	66.3175	68.9768
**C42**	176.0014	178.2125	184.4365	179.4227	178.2096	175.5302	178.9895	181.0234	177.4493
**C15**	63.6030	66.2750	68.0472	65.0315	66.2743	61.4541	65.4272	68.6585	61.5559
**C16**	117.2932	113.1768	118.4736	115.6668	113.1745	116.1840	115.7960	116.0556	116.0514
**C19**	88.2105	88.8251	90.7673	89.3257	88.8249	89.1760	87.8720	88.1325	89.8004
**C18**	159.4306	154.3550	156.7115	155.6059	154.3548	158.3148	158.7067	153.0251	158.2595
**C17**	162.7791	160.7130	161.1320	162.4404	160.7117	166.8198	165.2927	163.5635	166.0065
**C20**	161.9584	159.0213	160.8567	160.5121	159.0212	160.6530	160.8622	158.5916	161.0701
**C21**	166.6965	166.4195	166.7273	166.3643	166.4202	166.4498	166.7905	167.1259	167.4069
**C22**	123.3798	124.4195	124.5795	123.2002	124.4191	124.1379	125.8481	125.9713	126.0460
**C23**	118.8268	116.4711	122.1711	119.3732	116.4714	117.3129	117.4561	116.9796	118.3576
**C26**	111.4413	111.5445	110.1887	112.4912	111.5446	113.1854	109.4049	110.4211	109.5608
**C25**	45.8845	47.1381	48.0234	42.7812	47.1369	48.0784	52.9719	52.2647	53.7910
**C24**	120.7679	123.1780	123.2834	121.2571	123.1773	122.8472	123.2508	124.4140	123.4089
**C27**	114.3553	114.0667	131.0193	117.3335	114.0677	117.0401	131.0556	130.1977	129.3769
**C28**	155.9631	157.3128	158.8393	161.1515	157.3131	159.4715	158.8940	161.0906	159.8780
**C29**	153.0450	153.7778	161.3235	161.8650	153.7781	162.2865	161.3247	158.5579	157.2133
**C30**	119.2983	119.9780	119.5739	120.3488	119.9775	122.1486	120.9823	122.2343	125.3140
**C40**	177.2585	175.0045	178.8701	179.8898	175.0054	180.2905	180.6119	176.1786	179.5199
**C34**	96.2800	96.9331	98.3242	96.6827	96.9331	97.5660	97.5328	99.3068	101.2939
**C33**	163.5175	162.9881	162.1247	163.0945	162.9880	162.2378	163.0567	161.4386	164.3945
**C32**	165.0133	164.7666	168.8777	167.6152	164.7677	168.5131	167.7065	168.4228	167.1569
**C31**	162.0147	162.9130	164.8756	164.8557	162.9103	164.6293	164.8457	164.8051	166.2448
**C35**	160.3742	160.2947	159.5359	157.8964	160.2946	159.9984	157.9165	159.6694	157.5815

**C36**	173.9226	173.0767	174.0858	174.6156	173.0777	173.9153	173.2586	173.9230	174.9000
**C37**	169.2331	168.9751	168.9067	167.6319	168.9756	168.0842	166.8617	169.1055	168.8777
**C38**	133.5562	133.6955	134.3376	134.4453	133.6958	134.7098	134.6368	134.7733	134.5263
**C39**	180.8930	180.3020	183.8510	184.4443	180.3027	187.5245	185.5025	184.9640	182.9749
**C43**	172.0304	173.6359	170.2587	173.1294	173.6382	173.5124	169.7447	171.5241	169.4873
**C41**	90.5848	89.2480	85.0647	87.6332	89.2504	89.7543	85.1134	82.2123	83.8411
**H22**	27.2214	27.9863	27.2245	27.2214	27.4301	27.8761	27.9394	28.0694	27.1637
**H30**	27.4999	28.0190	27.9686	27.4997	27.8973	28.0963	27.7362	27.7658	27.4320
**H4**	27.2490	27.4822	27.2672	27.2494	27.2264	27.3756	27.4007	27.4917	27.5361
**H12**	27.9573	27.9089	27.9150	27.9565	27.5520	27.7627	27.9112	27.5350	28.0602
**H16**	26.5142	26.5198	26.8567	26.5139	26.6631	26.3519	25.9497	26.6012	27.0088
**H23**	28.1783	28.0164	28.0216	28.1781	28.1683	28.0573	28.0717	27.9890	28.3038
**H26**	27.5368	27.7865	27.4898	27.5367	27.5599	27.6200	27.8498	27.6850	27.5253
**H3a**	27.9996	29.3291	29.3004	27.9993	29.4601	29.4301	28.4721	29.4426	29.4015
**H3b**	29.9784	30.0630	30.0245	29.9784	30.0083	30.0930	30.0877	30.0363	30.0276
**C$\underline{H_3}$-2**	30.1373	30.2511	30.2642	30.1374	30.1347	30.2510	30.1929	30.2280	30.2617
**H5a**	30.3072	30.3234	30.2948	30.3074	30.0628	30.2382	30.3096	30.0530	30.2533
**H5b**	29.9731	29.6321	29.6668	29.9733	29.9281	30.0820	29.9498	30.3114	29.6704
**H6a**	29.8144	29.7774	29.7336	29.8142	29.7323	29.6699	29.6956	29.6247	29.8582
**H6b**	29.9965	29.8616	29.7544	29.9968	29.9233	29.8486	29.8185	29.7417	29.8672
**H7**	28.1672	28.1924	28.2172	28.1674	27.9996	28.0895	28.1525	28.0430	28.2002
**H11a**	29.8769	29.6543	29.6774	29.8770	30.0506	29.4624	29.6831	29.9370	29.6710
**H11b**	29.6396	29.4740	29.6300	29.6401	29.5396	29.5372	29.5591	28.8932	29.7002
**H9**	25.9600	25.8778	26.0779	25.9606	26.1406	25.9040	26.0042	25.5277	25.9784
**H13**	29.2824	29.1923	29.3880	29.2821	29.3617	29.0907	29.0949	29.3773	29.4712
**H14**	25.3003	24.5321	25.3511	25.2999	25.7570	24.8043	24.6787	25.7334	25.3112
**C$\underline{H_3}$-13**	30.7082	30.3939	30.6417	30.7085	30.2124	30.4064	30.5392	30.2076	30.4504
**H15**	25.5486	25.2252	25.6567	25.5482	25.5660	25.4175	25.3116	25.4708	25.7665
**H18a**	29.4469	29.5598	29.4191	29.4469	29.7830	29.7650	29.2621	29.7813	29.4887
**H18b**	29.6488	29.6710	29.7240	29.6487	29.9465	29.9186	29.4797	29.8574	29.7043
**H17a**	29.6974	29.3425	29.5772	29.6973	29.2663	29.1388	29.4289	29.2226	29.5048
**H17b**	30.0270	30.0752	30.0162	30.0269	29.5847	29.8636	30.1091	29.6538	30.0126
**H20a**	29.7496	29.7845	29.7879	29.7495	29.7106	29.6140	29.6715	29.6861	29.8960
**H20b**	29.8206	29.8479	29.9117	29.8206	29.9685	29.7511	29.5956	29.9137	29.9199
**H21a**	30.0132	29.7134	29.8855	30.0132	29.8641	29.6245	29.6839	29.6928	29.9936
**H21b**	29.8783	29.8312	29.7690	29.8783	29.8021	29.7074	29.8515	29.8361	29.8665
**H24**	26.5571	27.4109	27.3128	26.5567	26.7048	27.1761	27.1365	27.3231	26.8743
**H27**	27.8793	27.4496	27.5912	27.8793	27.4358	27.2525	27.3449	27.6430	27.7722
**H28a**	30.0479	30.5221	30.7173	30.0478	30.6553	30.5366	29.7692	29.8631	30.1297
**H28b**	29.3452	30.3314	29.4958	29.3452	29.4206	30.0970	30.5392	30.1442	29.3600
**H29**	29.4709	29.3314	29.4734	29.4707	29.9790	29.4842	29.7147	29.7844	29.4455

**$C\underline{H}_3$-29**	30.5528	30.5292	30.4166	30.5527	30.4121	30.4869	30.1461	30.2387	30.5792
**H33a**	29.7920	30.0103	29.8438	29.7919	30.0103	29.9174	30.1689	30.1191	29.8718
**H33b**	30.2304	30.3617	30.3241	30.2302	30.5577	30.2548	30.4539	30.2502	30.2954
**H32a**	30.2417	30.1950	30.2853	30.2416	30.1744	30.2514	30.2893	30.4598	30.2963
**H32b**	29.4205	29.4190	29.4341	29.4207	29.5002	29.4326	29.5359	29.4482	29.5206
**H31**	29.6074	29.9315	29.8631	29.6074	29.7561	29.8022	30.0008	30.1339	29.6184
**H35a**	29.9896	30.0651	29.9537	29.9895	30.0986	30.0256	30.1845	30.0000	30.1411
**H35b**	30.1194	30.4517	30.3516	30.1195	30.2164	30.5016	30.2687	30.2909	30.1391
**H36a**	30.1151	29.5280	29.6362	30.1152	29.6786	29.7339	29.4799	29.5424	30.0985
**H36b**	29.7836	30.1133	30.1102	29.7837	30.0806	30.0880	30.1731	30.0870	29.7841
**H37a**	29.9928	29.9402	30.0952	29.9928	29.8766	30.1769	29.8108	29.9823	29.9007
**H37b**	30.1900	30.2538	30.1914	30.1901	30.3048	30.2412	30.1640	30.2106	30.1967
**H38a**	27.9631	28.0549	28.0093	27.9632	27.9244	27.9409	27.9116	28.0071	28.0158
**H38b**	27.6967	27.6096	27.6959	27.6968	27.7283	27.7839	27.4262	27.6555	27.8905
**$C\underline{H}_3$-31**	30.3997	30.6294	30.6418	30.3997	30.5224	30.5774	30.7929	30.7669	30.4038
**$C\underline{H}_3$-10**	29.7288	29.8103	29.7106	29.7286	29.6416	29.7621	29.8737	29.8839	29.6944
**H41a**	26.0447	25.5067	25.5876	26.0447	25.8157	25.7226	25.6451	25.6810	25.8040
**H41b**	26.3043	26.1697	25.8240	26.3046	26.1349	26.0856	26.0448	25.8465	26.1358

**Atom (σ)**	**2-19**	**2-20**	**2-21**	**2-22**	**2-23**	**2-24**	**2-25**	**2-26**	**2-27**
**C1**	20.2345	21.8752	22.0271	22.5173	21.8940	22.9478	22.5032	22.7015	22.4751
**C2**	115.8671	116.7387	116.2188	116.7347	116.0036	116.3485	117.4694	115.7216	116.2841
**C3**	154.5148	153.8583	153.6313	153.2861	153.1413	153.5912	153.6096	152.8002	153.9581
**C4**	123.4440	125.7304	124.6177	125.4422	125.0331	123.9232	125.9577	124.0524	123.5914
**C44**	169.6248	169.2843	167.1822	168.4739	168.2141	168.4771	168.3796	168.3672	168.8971
**C5**	163.2748	163.4978	163.2688	164.4495	163.0721	160.4363	162.4744	162.7864	162.3556
**C6**	166.3561	165.0604	163.9668	166.6782	166.5279	167.1514	165.2156	165.5456	165.3749
**C7**	122.4689	126.0022	124.6761	124.1458	123.4359	125.6223	124.4272	123.8875	125.9199
**C8**	96.7031	98.4687	98.7789	98.1246	95.7959	99.1168	101.2483	97.2837	98.0717
**C12**	123.6110	124.1596	125.3198	123.1344	120.4219	123.5660	122.3305	120.1893	123.8861
**C11**	162.3064	160.0594	165.1043	159.9847	161.4238	160.8432	166.1486	160.4347	161.2764
**C10**	53.0248	55.8508	54.4658	56.3530	53.9427	52.0949	54.2839	53.7219	55.6098
**C9**	73.6957	73.9867	73.8768	75.8905	75.4833	75.8726	76.6163	74.6510	77.3027
**C13**	148.4046	148.4979	154.5296	148.4741	153.6762	150.8917	153.4408	154.6467	150.4428
**C14**	59.6727	64.8305	67.4713	65.5224	67.9801	64.1619	70.0993	69.8902	69.5086
**C42**	181.2411	179.1128	185.0442	178.4939	182.4593	177.1666	182.4312	181.1432	175.1564
**C15**	64.7754	66.5107	67.3720	64.9641	68.3282	63.1174	61.7936	65.3947	59.7666
**C16**	116.1956	115.5186	115.5038	114.8995	117.2007	114.8550	116.4805	114.9446	116.6331
**C19**	87.9436	89.6597	87.2130	89.3698	89.5475	88.4022	88.3497	88.1673	89.2395
**C18**	156.2814	157.5284	159.3408	158.0634	155.9895	157.1319	158.8558	158.9742	158.1670
**C17**	163.1907	168.8572	163.1461	168.8003	162.0954	163.2215	164.7589	165.1548	164.3506

**C20**	160.5984	160.6597	161.5591	160.2353	158.9959	161.5655	161.0757	161.7144	160.7374
**C21**	165.6049	167.0301	166.1681	168.2296	166.7719	166.9773	166.7316	165.5745	167.9679
**C22**	123.0629	122.7990	126.3732	125.2617	124.9316	124.2363	123.6413	123.1434	123.3795
**C23**	119.0686	117.4028	117.3176	118.1827	120.8100	117.5651	118.1056	117.7843	117.3108
**C26**	112.7578	111.9297	109.1683	110.1357	109.8658	110.2830	113.6434	113.1577	111.1067
**C25**	39.9611	47.8210	53.2548	53.6714	47.5300	48.5696	47.4290	47.4289	47.6041
**C24**	121.6850	122.9270	124.3564	123.4251	122.0845	123.4777	121.8224	123.7091	121.4325
**C27**	113.8098	115.7738	130.7118	129.5132	130.4613	113.8353	117.2593	117.4055	113.6524
**C28**	156.8833	158.0697	158.6761	160.7444	161.2247	158.4805	158.7360	158.6291	156.7516
**C29**	154.1269	160.2225	161.0758	156.9900	158.7031	152.9484	162.9777	162.3832	151.9600
**C30**	118.4792	122.0941	119.8389	121.8400	121.7593	119.4408	120.0424	120.6958	118.9527
**C40**	175.0651	179.2219	179.6098	176.9371	176.3370	177.2352	179.3168	180.7119	178.3227
**C34**	95.9871	99.1397	96.9565	98.7276	97.7264	97.0810	96.8016	97.0183	96.1671
**C33**	163.9613	161.8504	162.8212	162.6648	161.8796	163.9508	163.3098	163.2914	163.8892
**C32**	164.8428	169.4960	168.6857	167.0482	168.9361	164.5150	166.8662	165.9515	164.8337
**C31**	163.1607	165.4624	165.9358	164.8954	163.9420	163.7266	164.9923	165.3797	162.5789
**C35**	160.7219	161.0765	159.1529	157.8312	159.0801	159.2832	158.6887	158.2552	159.7202
**C36**	173.4250	171.7926	173.7698	174.2390	175.3828	173.5932	174.5438	173.6962	174.9076
**C37**	169.8443	168.9881	168.3234	168.8042	168.4673	169.9497	168.5905	168.4664	168.1113
**C38**	133.6561	132.9047	134.2186	134.4994	134.6197	133.1429	134.5473	134.2205	133.9401
**C39**	180.7332	185.1817	184.0834	182.4281	184.5031	181.2606	185.1594	185.3192	179.9362
**C43**	168.7218	173.8486	170.1803	173.8772	169.4052	171.1823	170.8429	169.4483	168.9704
**C41**	89.8115	89.7334	85.3429	84.9723	84.1194	90.6370	88.8558	89.7501	90.2805
**H22**	27.1917	27.8488	27.9993	27.8549	27.1700	27.3827	27.3680	27.1466	27.7541
**H30**	28.0262	28.1328	27.5974	27.5512	27.3953	27.9711	28.0404	27.1802	28.1808
**H4**	27.5863	27.0301	27.6728	27.5923	27.4434	27.0541	27.3897	27.2356	27.1354
**H12**	27.8304	27.4240	27.9135	28.0748	27.4192	27.8754	28.0421	27.5550	28.0734
**H16**	26.6881	26.6146	26.6434	26.4741	27.0017	26.5413	26.3728	26.5856	26.9461
**H23**	28.0669	28.0282	28.0266	27.9041	28.1043	28.0524	28.0625	28.0084	28.0681
**H26**	27.5534	27.7336	27.7794	27.7861	27.3198	27.3555	27.5122	27.4778	27.6958
**H3a**	29.3962	29.4503	29.3806	29.3557	29.4436	29.4342	29.4462	29.3560	29.0539
**H3b**	30.1026	30.0119	30.0848	30.0539	30.1370	30.0719	29.9717	29.9520	29.9745
**C$\underline{H_3}$-2**	30.2007	30.2743	30.2657	30.3005	30.1733	30.3017	30.2420	30.1783	30.2228
**H5a**	30.2950	29.8979	30.2411	30.3077	30.0791	29.8280	30.1823	30.0018	30.1461
**H5b**	30.1599	29.9190	30.1747	29.6988	30.3158	30.0916	29.9156	30.1145	29.6601
**H6a**	29.9842	29.7131	29.9217	29.7987	29.6206	29.7182	29.7369	29.5819	29.8534
**H6b**	30.0032	29.9460	30.1288	29.7304	29.8046	29.8111	29.9082	29.8449	29.8487
**H7**	28.3037	27.9114	28.2803	28.1303	28.0802	28.2778	28.0866	27.8707	28.1484
**H11a**	29.4207	30.0304	29.5347	29.5449	29.8619	29.8369	29.5344	30.1146	29.6662
**H11b**	29.4228	29.6586	29.4889	29.4685	28.8457	29.0357	29.4160	29.5392	29.5830
**H9**	25.3417	26.0628	25.8686	25.9718	25.7664	26.0536	26.0013	26.3871	26.1257

**H13**	29.4157	28.8375	29.3437	29.2909	29.2694	28.9694	29.2168	29.3401	29.1503
**H14**	25.6055	25.0077	25.4913	24.5976	25.3623	25.4508	24.7806	25.5649	24.5350
**C$\underline{H}_3$-13**	30.5592	30.4714	30.5523	30.5204	30.2351	30.3384	30.3530	30.1948	30.5611
**H15**	25.5882	25.7792	25.5031	25.3200	25.6326	25.1538	25.6950	25.4532	25.6052
**H18a**	29.7543	29.8902	29.7100	29.3220	29.7518	29.8604	29.8296	29.7512	29.4918
**H18b**	29.8187	29.8054	29.7558	29.5718	29.7752	29.9811	29.8791	29.8636	29.7596
**H17a**	29.2749	29.1149	29.3722	29.4800	29.2424	29.2341	29.2147	29.1738	28.9696
**H17b**	28.6984	29.9332	28.6296	30.1208	29.9359	29.5188	29.9313	29.5837	29.9910
**H20a**	29.6674	29.6746	29.6511	29.7111	29.7364	29.6770	29.7200	29.7517	29.7026
**H20b**	29.9616	29.0876	29.9880	29.7816	29.8031	29.7510	29.7435	29.9559	29.8091
**H21a**	29.7920	29.6813	29.6409	29.6455	29.7951	29.7527	29.8638	29.8480	29.6656
**H21b**	29.7608	29.7511	29.7999	29.7370	29.8122	29.6846	29.8107	29.7987	29.7340
**H24**	26.5130	27.2083	27.2854	27.4318	26.5601	26.6845	26.5188	26.5407	27.0676
**H27**	27.7456	27.2966	27.3707	27.2023	27.7518	27.5077	27.4784	27.6743	27.3892
**H28a**	30.6552	30.6569	29.8539	29.7623	30.1629	30.5627	30.5556	30.1222	30.6242
**H28b**	29.1865	30.3013	30.3267	30.6039	29.2027	29.2563	29.2762	29.1874	30.1237
**H29**	30.0923	29.4056	29.6463	29.7611	29.4075	30.2641	30.0532	29.5271	28.9614
**C$\underline{H}_3$-29**	30.4136	30.4456	30.2088	30.1668	30.5288	30.4195	30.5513	30.5573	30.4461
**H33a**	30.0876	29.8831	29.9829	30.0357	29.6473	29.9794	29.9148	29.8250	29.9449
**H33b**	30.4685	30.3289	30.3025	30.4004	30.1871	30.1658	30.1762	30.4128	30.3474
**H32a**	30.2260	30.2050	30.3308	30.2858	30.2261	30.2616	30.2988	30.3152	30.2285
**H32b**	29.5514	29.4604	29.3819	29.4004	29.7082	29.4562	29.5980	29.8132	29.5779
**H31**	29.6567	29.8541	29.9895	30.0631	29.5908	29.5573	29.7419	29.6342	29.7923
**H35a**	30.1547	30.0365	30.0288	30.0542	29.8981	29.7834	29.9541	30.1017	30.0957
**H35b**	30.1720	30.4538	30.2543	30.2776	30.3146	30.3038	30.3085	30.1885	30.3088
**H36a**	29.5081	29.5626	29.4678	29.5079	30.1425	29.5193	30.0862	30.1540	29.6215
**H36b**	29.9114	30.0837	30.1662	30.1611	29.6895	30.1529	29.6181	29.6991	30.0413
**H37a**	29.8106	30.0139	29.9110	29.9471	30.0144	29.9873	29.9810	29.9368	29.9700
**H37b**	30.2374	30.1839	30.0919	30.1845	30.2989	30.3622	30.2961	30.1962	30.2158
**H38a**	28.0358	27.9823	28.0366	27.9480	28.0695	28.0637	28.0484	27.8264	28.1078
**H38b**	27.5805	27.6492	27.5674	27.5878	27.9493	27.7627	27.8216	27.6898	27.8311
**C$\underline{H}_3$-31**	30.5865	30.6392	30.6922	30.8351	30.3441	30.4878	30.5851	30.4519	30.6690
**C$\underline{H}_3$-10**	29.6741	29.8710	29.7480	29.8250	29.7926	29.8578	29.6889	29.9271	29.6743
**H41a**	26.0638	25.8053	25.7633	25.6502	26.1384	25.8143	25.8868	26.0631	25.9505
**H41b**	26.3183	26.1429	25.9449	26.1884	26.2161	26.2778	26.2178	26.3064	26.1158

**Atom (σ)**	**2-28**	**2-29**	**2-30**	**2-31**	**2-32**	**2-33**	**2-34**	**2-35**	**2-36**
**C1**	20.4345	23.6854	20.8956	22.7636	20.1728	20.6329	22.7897	23.7226	21.4745
**C2**	115.7804	116.3339	119.3664	116.1040	116.6724	119.0554	115.9562	115.8934	115.7570
**C3**	153.4068	154.6869	148.7309	154.1001	153.7145	149.1997	152.8345	153.4113	153.0677

**C4**	124.3094	124.5807	124.5394	124.3809	125.5302	124.0246	125.1047	123.8876	123.7053
**C44**	168.1433	169.0732	169.2659	169.8561	168.8852	169.4396	167.1515	168.2910	167.9591
**C5**	163.2826	163.3372	162.5492	162.4126	164.9260	162.0935	162.5567	163.1339	162.0573
**C6**	165.6817	164.4600	166.0020	166.1618	165.7662	164.7451	166.0883	165.9465	167.9914
**C7**	123.9597	124.4045	124.3916	122.8815	124.0044	124.4481	123.3849	124.1918	123.1360
**C8**	94.8032	97.4511	97.4176	99.0583	96.8001	96.5416	95.0654	97.4139	95.1967
**C12**	122.2837	120.3452	120.7062	122.4757	120.7956	122.7935	119.6987	123.5380	124.7842
**C11**	160.8533	164.0212	160.1617	162.9970	161.9022	160.3875	159.9990	160.4758	164.9876
**C10**	56.6117	56.0535	56.3943	57.3567	56.4461	55.0191	54.4383	53.5865	53.5525
**C9**	75.4796	79.9911	73.5602	81.1752	77.1021	73.7267	74.4813	79.0629	77.2936
**C13**	155.3504	151.2768	151.9143	151.1012	149.6020	154.1338	153.6275	151.5105	155.1277
**C14**	52.7706	66.4611	67.4293	69.0206	65.1516	65.9071	69.8238	68.9599	67.9648
**C42**	181.2931	176.7334	182.1798	176.2497	176.6687	182.3955	181.1392	175.6842	185.3657
**C15**	71.9949	67.4031	68.5476	63.2196	65.0050	68.1852	64.8397	63.7306	65.3206
**C16**	115.0651	117.6136	114.3938	116.9272	117.6854	114.0733	115.2932	116.5476	115.0678
**C19**	88.5265	89.4719	87.2035	88.3654	88.1561	86.1611	88.6198	86.9770	87.7832
**C18**	156.7595	156.9438	158.5963	159.4638	155.5997	158.1575	159.4204	159.4346	159.5105
**C17**	164.3631	162.0071	163.5642	164.2936	165.3060	163.8929	165.1130	162.4909	163.5455
**C20**	161.3039	161.0935	161.6538	162.4372	160.4980	160.0769	161.9045	162.2337	162.8932
**C21**	165.9255	164.3548	168.0768	167.2117	167.7134	167.0333	166.4840	166.3837	165.5882
**C22**	125.7310	123.9799	126.3079	125.5863	125.6902	124.3941	125.3861	124.1648	122.9845
**C23**	118.0150	116.8039	117.3869	116.9133	117.2387	116.8948	117.9606	119.1629	116.1193
**C26**	109.0246	113.3074	109.1069	110.0816	109.5497	112.8026	109.4176	112.2572	113.3085
**C25**	53.1910	48.0786	54.8378	54.3106	53.6052	48.5133	53.4034	50.0922	46.1438
**C24**	123.5859	124.2383	122.9700	123.2080	123.6337	121.6881	124.0279	121.0137	124.6395
**C27**	131.9274	117.1299	130.7037	128.7767	132.0312	117.8601	129.7209	115.7269	115.8180
**C28**	158.3752	157.8791	158.8883	161.7005	159.1046	158.1570	160.0202	160.4326	158.2793
**C29**	160.9931	161.8909	161.5428	156.8850	162.0573	161.9117	158.9024	159.1015	162.0984
**C30**	121.0028	121.4600	120.9191	121.6729	120.9094	120.1639	121.1081	122.3175	120.5706
**C40**	180.4438	179.9876	178.0802	177.4506	179.2334	180.3400	178.0921	176.0237	180.8040
**C34**	97.9407	96.7817	97.7090	96.5605	99.3157	98.1716	98.3119	99.4862	98.3425
**C33**	162.2623	162.5247	163.1012	163.4626	162.8209	162.4255	162.8053	163.6960	162.8553
**C32**	166.3543	167.7488	167.1075	167.5899	168.3626	166.5649	167.0374	167.3239	167.9611
**C31**	166.4221	165.7654	164.9526	163.0490	164.7701	165.1420	164.4151	163.6906	165.7497
**C35**	159.1580	158.3960	159.4735	158.9929	159.9856	158.8073	158.8998	158.6093	159.5111
**C36**	173.5921	174.1589	174.3106	175.0562	174.2004	173.9487	175.1847	174.1677	174.4649
**C37**	168.3518	168.3114	168.1131	168.2215	168.1168	168.6628	168.6204	166.4774	168.1764
**C38**	134.3662	133.8390	134.5020	134.7050	133.9654	133.8990	135.0717	135.5191	134.3597
**C39**	182.4897	183.0475	183.9974	184.4565	184.6194	185.6863	183.4484	184.9489	183.6351
**C43**	170.2889	170.1406	171.3725	171.8201	170.9961	172.3934	170.7419	168.9271	172.1916
**C41**	85.9344	89.6578	84.1738	84.0656	85.2805	88.7063	84.0716	86.7697	89.4614

**H22**	27.3117	27.9064	27.8932	27.7417	27.2475	27.9587	27.3893	27.2720	27.8856
**H30**	27.9019	27.9197	27.6174	28.0422	28.0478	27.7201	27.4801	27.9255	28.0499
**H4**	27.3427	27.3815	27.3406	27.5031	27.2380	27.2192	27.4271	27.5528	27.4782
**H12**	27.6899	27.6677	27.4135	27.4311	27.8425	27.9001	27.3865	27.4435	27.6988
**H16**	26.8035	26.2834	26.6936	26.8447	26.2832	26.2112	26.7352	26.7260	26.5956
**H23**	28.0487	28.1367	28.0479	27.8930	28.1002	28.0366	27.9471	28.0034	28.0279
**H26**	27.4523	27.6478	27.7437	27.7812	27.2680	27.8074	27.4027	27.3715	27.5865
**H3a**	29.4005	29.4442	29.4211	29.3875	29.4572	29.5110	29.4721	29.4558	29.3809
**H3b**	29.9118	29.9535	30.0282	30.0270	29.9020	29.9563	30.0381	29.8760	29.9608
**C$\underline{H_3}$-2**	30.2480	30.1954	30.1761	30.2510	30.1778	30.2277	30.1959	30.2474	30.2849
**H5a**	30.2440	30.1558	30.1129	30.0087	30.1890	30.1690	30.1641	30.2458	30.1778
**H5b**	29.8868	30.1020	30.0868	30.1015	30.0194	29.9826	30.0934	30.0436	30.1447
**H6a**	29.8529	29.8897	29.6882	29.7556	29.9401	29.6999	29.8968	29.7269	29.8884
**H6b**	29.8817	29.8298	29.7529	29.9162	29.7386	29.8670	29.7788	29.7092	29.9093
**H7**	28.1366	28.2198	28.1705	28.0379	28.1540	28.1410	28.0438	28.2609	28.2143
**H11a**	29.9771	29.7421	30.0354	29.9851	29.9955	29.5436	30.1892	30.0504	30.0597
**H11b**	29.2894	29.6710	29.3255	29.1785	29.5383	29.5773	28.9256	29.4811	28.9134
**H9**	26.3976	25.9727	26.4698	26.8899	26.0119	25.8510	26.0770	26.0512	25.8281
**H13**	29.6294	29.1680	29.4360	29.1312	29.2184	29.0440	29.4718	28.9256	29.2736
**H14**	25.1943	25.0368	25.3494	25.5966	24.8962	24.8370	25.2547	25.1183	25.0013
**C$\underline{H_3}$-13**	30.1672	30.6544	30.1842	30.1839	30.6244	30.3892	30.1455	30.5617	30.2793
**H15**	25.6279	25.7088	25.7491	25.1754	25.8844	25.7574	25.7698	25.8125	25.6509
**H18a**	29.7283	29.7189	29.7656	29.4242	29.8113	29.8395	29.8217	29.7961	29.7998
**H18b**	29.7221	29.6529	29.8530	29.5521	29.7187	29.9013	29.9566	29.7305	29.8447
**H17a**	29.5705	28.9622	29.1493	29.6470	29.0103	29.1318	29.2034	29.1528	29.0670
**H17b**	30.1990	29.7374	30.0469	29.9397	29.7634	30.0015	29.9068	29.8892	29.8458
**H20a**	29.8946	29.5706	29.7772	29.7743	29.5995	29.6573	29.8054	29.6849	29.6267
**H20b**	29.4504	29.5927	29.4878	29.7313	29.5863	29.7414	29.5559	29.2351	29.7155
**H21a**	29.9457	29.6756	29.6239	29.6365	29.8863	29.6397	29.9472	29.7529	29.6002
**H21b**	29.9335	29.7572	29.7500	29.7154	29.6794	29.7512	29.7551	29.7238	29.7265
**H24**	26.5794	27.1958	27.3205	27.0374	26.3168	27.2864	27.0884	26.4205	27.1797
**H27**	27.7561	27.3083	27.3809	27.4440	27.3596	27.3684	27.4641	27.5721	27.3300
**H28a**	30.6456	30.5172	29.8042	30.7107	30.4729	29.9909	29.9026	30.6191	30.5462
**H28b**	29.4522	30.1743	30.3749	30.1419	29.2750	30.3239	29.6420	29.2812	30.1914
**H29**	30.1299	29.3656	29.7522	29.3684	30.1236	29.5501	29.6871	29.9621	29.3965
**C$\underline{H_3}$-29**	30.4266	30.4880	30.1132	30.5663	30.4675	30.1083	30.1967	30.3953	30.5081
**H33a**	29.8947	29.9607	30.0872	30.0604	29.9692	30.0711	30.0478	29.9926	29.8760
**H33b**	30.2508	30.2900	30.2870	30.4938	30.3073	30.3261	30.2570	30.2972	30.2557
**H32a**	30.1898	30.2817	30.3269	30.2799	30.3364	30.3159	30.4011	30.2077	30.3601
**H32b**	29.5142	29.4534	29.4644	29.5027	29.5793	29.5388	29.3938	29.4400	29.5429
**H31**	29.8135	29.7322	30.2193	29.9002	29.7125	30.2074	29.9676	29.7994	29.6602

**H35a**	29.9603	30.0647	30.0555	30.2190	29.9745	30.0233	30.1660	29.9836	29.9917
**H35b**	30.2392	30.3761	30.2586	30.2245	30.2363	30.2334	30.3450	30.1997	30.3674
**H36a**	29.5903	30.0704	29.5560	29.6660	30.0927	29.5253	29.7274	29.6025	29.7691
**H36b**	30.0934	29.5914	30.1344	29.9957	29.5796	30.1586	30.0569	30.0224	30.0776
**H37a**	30.0412	30.0565	30.0169	29.9487	29.9442	29.9014	30.1821	29.8456	30.2463
**H37b**	30.1146	30.2960	30.1808	30.2125	30.2174	30.1954	30.2511	30.2114	30.2414
**H38a**	27.9823	28.0796	27.9942	28.0036	28.0119	28.0148	27.9931	27.9412	27.9441
**H38b**	27.6777	27.8669	27.7074	27.6790	27.6125	27.6299	27.8165	27.6962	27.9119
**C$\underline{H}_3$-31**	30.6209	30.5281	30.6882	30.6321	30.5925	30.7436	30.5515	30.7034	30.4752
**C$\underline{H}_3$-10**	29.8366	29.7219	29.9296	29.8274	29.7463	29.7475	29.6354	29.6381	29.7176
**H41a**	25.9969	25.6499	25.7608	25.8004	25.8773	25.7495	25.7038	26.1161	25.7087
**H41b**	26.2618	26.0660	26.0754	26.2427	26.0643	26.0635	26.1795	26.1563	26.0733

**Atom (σ)**	**2-37**	**2-38**	**2-39**	**2-40**	**2-41**	**2-42**	**2-43**	**2-44**	**2-45**
**C1**	23.8397	28.0577	21.0171	21.0335	21.0271	23.1385	22.4665	22.5441	23.2094
**C2**	116.2255	117.0292	115.7210	115.6852	115.5371	116.3023	116.6124	116.3441	116.5890
**C3**	153.9250	154.1104	154.0091	154.1901	154.2374	153.7052	153.2434	152.2854	153.9303
**C4**	125.0942	124.3140	124.1249	124.2132	123.6281	124.3330	123.2950	125.6437	124.2458
**C44**	169.8302	168.6499	168.0035	167.7707	168.8402	169.2812	168.0993	167.6245	168.3976
**C5**	164.1142	163.1597	162.7674	162.6989	162.6628	163.6617	162.6280	163.3368	163.8494
**C6**	166.0755	164.6993	166.9027	166.0316	166.8753	165.3958	165.0715	166.8673	165.0584
**C7**	124.2378	124.3836	123.3112	123.4241	124.1759	124.0259	124.4495	123.4266	123.3453
**C8**	99.0007	97.1093	94.8684	95.2343	94.1350	97.6586	96.8424	96.8514	97.1008
**C12**	124.1989	123.2744	119.9301	118.7938	121.4838	122.5613	123.1673	124.0950	120.5532
**C11**	163.0294	161.2642	161.5728	161.7264	162.5097	163.4671	165.4704	162.5020	159.5569
**C10**	53.6941	54.1273	55.7839	53.9543	53.8120	56.0716	55.0711	53.6452	56.7599
**C9**	74.2413	76.0323	75.1226	75.5813	74.2291	76.5276	75.8761	72.9951	74.9671
**C13**	155.2282	149.7796	154.1248	154.4604	151.1307	151.8445	151.9514	147.5846	154.5723
**C14**	70.4287	59.1889	67.7813	66.3604	57.0675	69.0101	70.2857	67.6126	68.5394
**C42**	180.4215	181.7090	182.9105	183.1555	179.9040	176.5657	180.6477	176.6474	181.6459
**C15**	65.3703	63.8505	68.8794	69.3610	67.7233	63.0867	59.4867	63.1457	65.1751
**C16**	115.6241	113.3770	116.9714	116.9579	114.2742	117.0250	116.3565	118.6749	115.7505
**C19**	87.9131	88.0572	88.4679	87.7093	87.5957	87.7979	89.0732	87.6316	88.4628
**C18**	159.0011	156.9222	153.5262	154.0615	154.7071	159.5331	159.2155	159.2523	159.3739
**C17**	163.7926	163.9386	162.6706	162.1119	161.3898	162.0741	164.2439	163.1518	162.9941
**C20**	160.8664	160.3034	158.6025	158.7443	158.3271	162.7357	161.2214	163.5300	161.2578
**C21**	167.0740	167.1197	165.7934	166.3766	165.6051	164.0940	165.7048	166.4639	166.6087
**C22**	125.9140	124.2260	124.1285	126.0448	125.7029	121.9515	123.8410	122.9093	123.7313
**C23**	117.8614	118.2114	116.8446	117.3574	115.5847	118.1149	117.1554	117.2810	117.2955
**C26**	109.7485	111.4790	111.9166	109.5998	111.5401	112.5140	111.9605	112.7790	111.1552
**C25**	53.7112	50.1893	45.0160	51.6966	47.0034	47.5780	47.5973	47.3893	47.6245

**C24**	123.5258	121.6320	122.6507	123.4638	123.9779	123.8532	123.5561	122.4008	122.6387
**C27**	130.9630	116.6713	114.6354	131.9649	114.2241	115.0810	114.3211	117.1373	114.1584
**C28**	159.1827	161.2480	156.7080	157.2930	159.1080	157.5543	153.6241	157.4049	155.8218
**C29**	161.4471	159.2160	154.2827	161.0557	158.5120	162.2071	153.1662	159.2935	153.2304
**C30**	121.3981	122.0342	118.8861	121.2826	121.1034	121.0813	119.1275	120.6914	119.1193
**C40**	179.3447	175.4059	174.4670	179.7002	175.4842	179.2165	178.5136	181.6484	175.3246
**C34**	96.3837	97.4153	95.4880	98.8442	95.2473	96.7614	96.1765	97.5172	98.3638
**C33**	162.7987	162.8993	163.9198	162.7409	163.2992	163.2918	163.1591	162.3639	162.8873
**C32**	166.0049	167.2315	164.6698	168.5623	167.0431	167.6433	164.7904	167.9016	164.8495
**C31**	164.7179	164.0531	162.9163	164.2085	163.4925	165.6458	163.6068	164.0105	162.4205
**C35**	157.5774	158.6253	160.6221	159.5056	159.5582	158.5433	159.9952	159.9449	160.0566
**C36**	173.2404	174.0446	173.1998	174.1227	175.0203	174.8351	173.3513	173.4408	175.1028
**C37**	166.0808	168.2701	169.3219	168.3938	168.4183	168.8574	168.2987	168.5501	169.2396
**C38**	135.0196	134.4576	133.6790	134.3646	134.6294	134.7262	133.9531	134.2791	133.8438
**C39**	185.9928	183.8043	181.4934	185.3361	180.9244	183.9764	178.9281	187.3988	181.0136
**C43**	171.5977	170.4626	170.4264	169.4171	170.8153	171.9905	170.7994	172.4580	171.1871
**C41**	84.7770	86.7197	87.3006	82.7220	88.6972	89.6787	90.4028	89.9914	89.9645
**H22**	27.3886	27.0886	27.8217	27.1117	27.7150	27.1874	27.2749	27.1599	27.9153
**H30**	27.6478	27.4136	28.0214	27.2905	27.9499	27.4644	27.9178	27.1269	27.4681
**H4**	27.1810	27.3354	27.2165	27.3366	27.4130	26.9985	27.4819	27.4174	27.3249
**H12**	27.9493	27.9391	27.9545	27.8603	27.5009	27.5657	28.0305	28.0946	27.7027
**H16**	26.4987	26.0960	25.8539	26.5713	26.7179	26.7237	26.8188	26.3248	26.8129
**H23**	27.9726	28.1972	28.0152	28.1608	27.9823	28.1846	28.0837	28.0701	28.0126
**H26**	27.5338	27.5589	27.7957	27.6332	27.2535	27.3578	27.5118	27.4446	27.7823
**H3a**	29.3855	28.3867	28.3902	28.0843	29.4321	29.3964	29.3971	29.3312	29.5220
**H3b**	29.9408	30.1403	29.9744	30.0894	29.9836	30.0329	30.0298	30.0359	30.0143
**C$\underline{H_3}$-2**	30.2443	30.1953	30.1176	30.1598	30.2368	30.2675	30.2887	30.2491	30.2602
**H5a**	30.0799	30.3657	30.2114	30.3481	30.0698	30.0862	30.1961	30.1250	30.0638
**H5b**	29.8080	29.8730	29.8709	30.0623	30.1135	29.9141	30.1022	29.9036	29.9814
**H6a**	29.7083	29.8248	29.7913	29.9764	29.7598	29.7695	30.1141	29.6628	30.1016
**H6b**	29.9455	29.9363	29.9053	30.0237	29.7616	29.9264	30.0236	29.8798	29.9299
**H7**	28.1196	28.2559	28.0818	28.2815	28.2159	28.2058	28.2668	28.0868	28.0612
**H11a**	29.6973	29.6122	29.7322	29.6756	30.3303	29.9370	29.6728	29.4038	29.5362
**H11b**	29.5993	29.5036	29.5879	29.5279	29.0180	29.2346	29.6626	29.4730	29.7153
**H9**	25.9728	25.8806	26.1037	25.8882	25.9112	26.1109	25.6966	25.7851	25.9018
**H13**	29.4396	29.1698	29.2079	29.3584	29.5659	28.6464	29.3372	29.2639	29.1618
**H14**	25.2694	24.7087	25.0133	25.3059	25.3828	25.7873	25.2955	24.8162	25.2386
**C$\underline{H_3}$-13**	30.6303	30.4707	30.6019	30.6404	30.2251	30.4646	30.5548	30.4823	30.4930
**H15**	25.7198	25.5815	25.5475	25.7017	25.6598	25.1862	25.2662	25.4640	25.5252
**H18a**	29.7719	29.3972	29.1794	29.3450	29.8115	29.8290	29.7976	29.8454	29.7417
**H18b**	29.7354	29.6340	29.4123	29.6073	29.9390	29.9360	29.3419	29.9003	29.8631

**H17a**	28.9835	29.6816	29.5091	29.8094	29.1378	29.2099	29.2056	29.1125	29.1538
**H17b**	29.9748	30.3463	30.1056	30.0064	29.8232	29.3052	29.9550	29.9050	29.9287
**H20a**	29.7430	29.8716	29.6357	29.8238	29.8133	29.7175	29.7682	29.6790	29.8095
**H20b**	29.8461	29.8059	29.6154	29.7654	29.4287	29.8757	28.6887	29.8092	28.6562
**H21a**	29.9854	29.9322	29.7162	30.0341	29.7794	29.9404	29.7527	29.8485	29.4573
**H21b**	29.7181	29.8231	29.8445	29.8861	29.7773	29.8574	29.7304	29.7800	29.7447
**H24**	27.0201	26.6400	27.0941	26.4929	26.5691	26.5810	26.5447	26.5355	27.3512
**H27**	27.6825	27.7802	27.2485	27.7861	27.7408	27.4704	27.4409	27.7247	27.2341
**H28a**	29.9221	30.1100	30.4547	30.3563	30.3963	29.9921	30.5492	30.1305	29.9186
**H28b**	29.8106	29.3296	30.0934	28.9766	29.4201	29.3228	29.3899	29.2255	30.5676
**H29**	29.6960	29.4132	29.2211	29.4681	29.8351	29.4425	30.0697	29.4750	29.8207
**C$\underline{H_3}$-29**	30.1971	30.6089	30.4405	30.5141	30.3586	30.4958	30.4047	30.5995	30.1285
**H33a**	29.8834	29.9172	30.0024	29.8144	29.9652	29.7514	29.9624	29.9324	30.0211
**H33b**	30.2910	30.2590	30.4289	30.2767	30.1630	30.2380	30.4849	30.1817	30.4391
**H32a**	30.3319	30.2726	30.2339	30.2244	30.2636	30.2600	30.1940	30.2535	30.3013
**H32b**	29.5290	29.5821	29.5106	29.5519	29.5125	29.5784	29.4962	29.6762	29.2209
**H31**	30.0735	29.5947	29.8635	29.7470	29.7956	29.6971	29.7373	29.6208	30.0442
**H35a**	30.0140	30.1478	30.1907	29.9358	29.7772	29.8690	30.0630	29.8441	29.9811
**H35b**	30.3071	30.0908	30.3912	30.2541	30.3357	30.2215	30.1512	30.1849	30.1476
**H36a**	29.5017	30.1159	29.5539	30.0711	29.4670	30.1916	29.6085	30.0573	29.6123
**H36b**	30.1244	29.7761	30.1166	29.5889	30.1658	29.7077	30.0562	29.4395	30.0446
**H37a**	30.0546	29.9442	29.9739	30.0310	29.9649	29.9897	29.9023	29.9081	29.9468
**H37b**	30.1391	30.2061	30.2159	30.2476	30.3422	30.1632	30.2574	30.2793	30.2498
**H38a**	28.0203	27.9831	27.9951	27.9414	28.0610	27.8368	27.9972	27.9845	28.0130
**H38b**	27.6348	27.8175	27.6645	27.7179	27.7845	27.6008	27.7306	27.7817	27.5944
**C$\underline{H_3}$-31**	30.6522	30.3825	30.5939	30.4840	30.5524	30.3678	30.5866	30.3882	30.7255
**C$\underline{H_3}$-10**	29.7844	29.7458	29.8151	29.6176	29.7975	29.8512	29.5592	29.8673	29.7705
**H41a**	25.7513	25.8439	25.4629	25.9621	26.0090	25.9275	26.0264	26.0500	25.7572
**H41b**	26.1277	26.1222	26.0126	26.2840	26.1083	26.2259	26.1757	26.3211	26.1095

**Atom (σ)**	**2-46**	**2-47**	**2-48**
**C1**	21.9378	21.6408	21.9199
**C2**	116.8930	116.4894	115.8522
**C3**	153.5662	153.8726	153.5886
**C4**	126.3761	125.2109	124.2379
**C44**	169.0296	168.4289	169.5357
**C5**	163.6585	163.3187	161.7906
**C6**	163.5080	165.4159	168.9371
**C7**	123.2656	124.9008	122.8428
**C8**	97.5224	97.1381	95.5451
**C12**	123.1667	124.3032	123.0490

**C11**	161.6794	161.0082	160.2566
**C10**	52.7594	54.8319	55.8417
**C9**	69.9067	74.6738	74.7088
**C13**	147.7002	147.9293	148.6227
**C14**	66.5274	66.7238	62.8228
**C42**	178.1558	177.5902	178.8926
**C15**	63.8690	64.4699	62.8971
**C16**	116.7317	118.5182	116.7051
**C19**	88.7922	87.4959	88.9676
**C18**	158.6308	159.4635	155.1799
**C17**	163.7409	163.7061	163.6370
**C20**	163.3223	163.6185	160.3396
**C21**	167.1043	167.1383	167.5088
**C22**	125.6868	125.8128	125.5417
**C23**	117.6899	119.0430	117.5967
**C26**	109.5945	109.7528	110.3942
**C25**	53.7458	53.7378	54.2362
**C24**	122.9408	123.3911	122.1826
**C27**	129.7194	129.2778	128.4124
**C28**	160.5540	161.0214	161.2197
**C29**	158.8153	157.1301	159.3083
**C30**	122.8882	120.2866	122.1759
**C40**	175.9667	176.8572	176.5061
**C34**	98.3948	97.8899	97.1538
**C33**	162.7662	162.3668	162.5726
**C32**	169.2764	166.6659	167.3616
**C31**	163.7240	165.3156	165.2962
**C35**	159.4303	158.7127	159.0246
**C36**	175.3884	174.5233	174.1187
**C37**	167.8553	169.2812	168.7483
**C38**	134.4130	134.8051	134.5600
**C39**	184.5848	183.4765	184.2564
**C43**	167.1965	172.5617	170.0621
**C41**	84.6857	84.3057	84.3546
**H22**	27.9855	27.8739	27.8739
**H30**	27.6946	27.5451	27.5451
**H4**	27.4558	27.4822	27.4822
**H12**	27.8649	27.8659	27.8659
**H16**	26.8697	26.6928	26.6928
**H23**	28.0323	27.7913	27.7913
**H26**	27.7881	27.8645	27.8645

**H3a**	29.4609	29.5548	29.5548
**H3b**	29.9906	30.0062	30.0062
**C<u>H</u>$_3$-2**	30.2447	30.2110	30.2110
**H5a**	30.1194	30.2998	30.2998
**H5b**	29.9999	30.2568	30.2568
**H6a**	29.8597	29.7386	29.7386
**H6b**	29.8364	29.7948	29.7948
**H7**	28.0226	28.3352	28.3352
**H11a**	29.8111	29.5399	29.5399
**H11b**	29.8212	29.5451	29.5451
**H9**	26.1449	25.9096	25.9096
**H13**	29.5232	29.3741	29.3741
**H14**	25.2172	25.6899	25.6899
**C<u>H</u>$_3$-13**	30.5901	30.5082	30.5082
**H15**	25.4180	24.9969	24.9969
**H18a**	29.8007	29.4091	29.4091
**H18b**	29.3775	29.5832	29.5832
**H17a**	29.2142	28.4813	28.4813
**H17b**	29.9651	29.9966	29.9966
**H20a**	29.8294	29.6838	29.6838
**H20b**	28.5626	29.7218	29.7218
**H21a**	29.6531	29.6328	29.6328
**H21b**	29.8017	29.7558	29.7558
**H24**	27.2792	27.1479	27.1479
**H27**	27.3352	27.3426	27.3426
**H28a**	29.7407	29.7849	29.7849
**H28b**	30.4163	30.5675	30.5675
**H29**	29.6551	29.7621	29.7621
**C<u>H</u>$_3$-29**	30.1208	30.1281	30.1281
**H33a**	29.9628	30.1248	30.1248
**H33b**	30.3059	30.3227	30.3227
**H32a**	30.3082	30.2178	30.2178
**H32b**	29.5653	29.4407	29.4407
**H31**	30.0608	29.8814	29.8814
**H35a**	30.0218	30.0860	30.0860
**H35b**	30.2287	30.2745	30.2745
**H36a**	29.5203	29.5735	29.5735
**H36b**	30.1556	30.1512	30.1512
**H37a**	29.9104	29.9889	29.9889
**H37b**	30.1551	30.2102	30.2102
**H38a**	27.9744	28.0234	28.0234

**H38b**	27.6104	27.6495	27.6495
**C$\underline{H_3}$-31**	30.7653	30.7400	30.7400
**C$\underline{H_3}$-10**	29.6621	29.7489	29.7489
**H41a**	25.7374	25.7742	25.7742
**H41b**	25.9366	25.9913	25.9913

Table S14. Isotropic magnetic shielding values computed for conformers 3-1 to 3-16 of euphodendroid K (**3**) at the PCM/B3LYP/6-31+G** level of theory.

Atom (σ)	3-1	3-2	3-3	3-4	3-5	3-6	3-7	3-8	3-9
**C1**	141.8200	142.1600	140.4300	149.3900	140.8800	148.7800	141.8900	143.0500	149.6800
**C2**	106.8800	107.0100	105.6400	106.2700	106.0600	106.2800	106.0300	106.5400	106.4900
**C3**	119.5700	120.1300	118.7000	115.4500	118.4000	115.2400	117.4700	119.5100	115.5700
**C4**	146.3600	146.1000	144.1700	144.5100	143.9100	144.4200	144.8100	143.1900	145.5800
**C5**	122.5600	121.8900	127.0500	121.4100	126.7000	121.8200	126.8700	128.0800	121.5400
**C6**	56.2300	56.0800	51.3300	57.2700	52.1800	58.0100	52.4500	55.2500	56.7400
**C7**	126.2500	126.5500	126.9500	127.7800	126.9500	128.3600	126.8600	121.3500	127.3200
**C8**	125.0200	124.7800	122.4500	124.3900	122.5800	124.4200	122.8800	124.6800	125.2900
**C9**	114.3800	114.3200	115.9400	114.3200	115.9600	113.7900	115.7800	115.2800	113.7500
**C10**	151.6500	151.8000	152.6600	151.9400	153.5300	152.0700	153.0700	151.8100	151.6100
**C11**	58.1900	58.5800	60.2100	59.2600	59.7800	59.6700	59.9800	62.7600	59.6700
**C12**	67.8400	67.9500	65.1600	69.1900	65.9000	69.3200	65.8500	64.1800	68.8700
**C13**	148.3800	148.7400	146.3600	149.1400	146.7600	150.1100	146.8400	147.6700	149.9400
**C14**	-22.2600	-22.0800	-18.2800	-22.5000	-18.2800	-22.1300	-17.8300	-17.3900	-22.3100
**C15**	107.7400	108.4400	108.5100	109.3600	108.7400	109.0100	108.4300	107.4900	111.3800
**C16**	177.9200	177.9300	175.9700	178.2800	176.0700	178.4500	175.8800	176.2100	178.1500
**C17**	76.1800	75.4700	78.7000	74.4200	79.2100	73.7800	78.9800	80.1400	74.0000
**C18**	171.7300	171.6900	174.6100	173.0300	175.6400	172.6800	173.6300	174.3700	172.5900
**C19**	168.9200	168.8800	168.3400	169.9300	168.4800	169.9200	168.5300	169.4500	170.0400
**C20**	171.5700	171.5500	173.1200	171.3000	173.5700	170.7400	173.6600	173.6500	170.5100
**C21**	25.7600	25.9700	25.3200	26.6500	26.1700	26.7200	26.4300	27.9500	26.8600
**C22**	171.5000	171.5800	170.4400	171.8700	170.8300	171.8400	170.8700	171.4200	171.8000
**C23**	21.8500	23.3000	23.8000	21.5300	24.8000	22.9400	22.5600	24.0500	20.5600
**C24**	158.7600	158.3000	158.2300	157.7100	157.8500	158.2900	158.1600	158.1200	157.6600
**C25**	177.8500	177.6600	176.4200	174.4300	177.6800	177.2300	175.8300	178.0100	175.6600
**C26**	175.3200	177.0300	175.5200	178.2300	176.6800	177.6400	173.9000	176.6300	178.6000
**C27**	21.5700	22.5800	24.0300	21.7000	22.7700	22.3100	22.0100	24.4600	21.2400
**C28**	157.5500	157.1500	158.1500	157.4400	155.9900	157.3100	155.3700	156.0000	155.4700
**C29**	177.0400	177.2200	177.4800	176.4600	176.0300	177.6700	175.0900	177.0600	174.2400
**C30**	177.9500	177.4100	174.9200	177.8000	174.5500	177.8700	174.7700	174.4500	179.2500
**C31**	29.1200	29.1000	32.0400	32.1300	32.3400	31.6500	32.1300	30.0400	30.4300

**C32**	67.4600	67.2200	67.9600	65.7800	67.7900	66.1500	67.9800	68.6300	66.0500
**C33**	64.4200	64.4300	65.2400	66.7700	67.5500	66.6800	65.6300	68.2200	67.3000
**C34**	69.1200	69.4000	70.0700	69.9700	70.7600	70.0300	70.3600	70.3600	69.9100
**C35**	64.8600	64.7400	65.0900	65.0600	64.6200	64.7700	64.6200	64.8500	64.4700
**C36**	70.7500	70.7800	70.7300	69.7900	70.3100	69.8700	70.7900	69.8100	70.1100
**C37**	67.4300	67.5600	67.4900	68.5800	65.5700	68.5500	67.4400	66.3800	66.7700
**C38**	25.3100	25.4200	25.8600	25.2700	25.4200	25.6700	25.3000	26.5100	25.1700
**C39**	173.8300	173.7700	174.0000	173.4700	174.1300	173.4600	174.1200	174.0400	173.6800
**C40**	26.0200	25.9400	25.3700	25.8500	25.7000	26.3100	25.6200	27.3100	26.4800
**C41**	172.4800	172.8900	172.9000	173.2300	171.5800	173.6800	172.5100	173.7700	172.7400
**H1a**	28.9800	28.8600	28.7700	28.0800	28.8500	28.0300	28.9300	28.7300	27.9600
**H1b**	29.2300	29.2200	29.4100	29.3300	29.4900	29.3500	29.4900	29.5300	29.3100
**H3**	25.1200	24.8500	25.5000	26.2500	25.5300	26.1300	25.7800	25.1100	26.2400
**H4**	27.1000	27.1400	27.9600	27.0600	28.0700	27.0100	28.1300	28.2200	26.8100
**H5**	25.3800	25.5500	26.0300	25.0800	26.2100	25.2100	26.1900	26.3000	25.0400
**H7**	24.4000	24.5400	25.9700	24.8400	26.2800	24.8700	26.2100	26.2500	24.7900
**H8**	26.4800	26.5000	27.1300	26.5400	27.1700	26.5200	27.1600	26.1700	26.3600
**H9**	26.4700	26.4400	26.8200	26.5500	26.8600	26.5500	26.8400	26.7600	26.3900
**H11**	24.3800	24.5600	25.2700	24.7200	25.4700	24.8300	25.4100	25.4900	24.7100
**H12**	25.4900	25.4600	25.6200	25.5000	25.6900	25.5300	25.6700	25.5000	25.5800
**H13**	27.0300	27.0500	27.1100	27.6300	27.0900	27.5800	27.1700	27.1300	27.5800
**H16**	30.0333	30.0467	29.7767	30.0233	29.8233	30.1000	29.8067	29.8100	30.0967
**H17a**	26.1600	26.1300	25.3800	26.2800	25.4900	26.0700	25.3900	26.1400	26.2300
**H17b**	25.7600	25.7900	25.7800	25.7700	25.8500	25.6800	25.7000	26.3700	25.6900
**H18**	30.2833	30.2667	30.3067	30.2767	30.2667	30.2967	30.3167	30.4300	30.2967
**H19**	30.4967	30.5100	30.5500	30.6400	30.5600	30.6333	30.5600	30.4533	30.6267
**H20**	30.1867	30.1833	29.9933	30.2500	29.9867	30.2200	30.0433	30.1200	30.2700
**H22**	28.8200	28.8333	29.2733	29.1067	29.2833	29.0800	29.3100	29.3033	29.0067
**H24**	28.8300	29.1000	28.8400	28.8500	28.4400	29.1300	28.8600	28.6100	28.8600
**H25**	30.6667	30.6267	30.4733	30.4800	30.3867	30.4333	30.3433	30.4600	30.4967
**H26**	30.3867	30.0333	30.2867	30.5500	30.2567	30.1267	30.3900	30.3267	30.5433
**H28**	29.8700	29.8100	28.9200	29.7900	29.0800	29.7100	29.1400	29.0800	29.4500
**H29**	31.4767	31.3900	30.5833	31.2100	30.4000	31.2067	30.4000	30.5400	30.6833
**H30**	30.8367	30.8667	30.4533	30.7933	30.1900	30.7533	30.2567	30.3233	31.2533
**H33**	22.4600	22.5200	22.5600	22.7300	23.0400	22.7700	22.7200	23.2400	22.8000
**H34**	23.5200	23.4900	23.6600	23.5800	23.7200	23.5600	23.6500	23.6900	23.5600
**H35**	23.5000	23.4800	23.4900	23.4600	23.5100	23.4400	23.5100	23.5200	23.4100
**H36**	23.7400	23.7400	23.7100	23.7100	23.6600	23.6700	23.7400	23.6300	23.6700
**H37**	23.4900	23.4200	23.0900	22.6700	22.6300	22.7900	23.0400	22.8100	23.1300
**H39**	29.4000	29.3833	29.2933	29.4467	29.3200	29.3967	29.3100	29.5467	29.3600
**H41**	30.1433	30.2100	30.0433	30.5267	30.0500	30.5000	30.1200	30.2500	30.5433

Atom (σ)	3-10	3-11	3-12	3-13	3-14	3-15	3-16
**C1**	141.8500	142.4300	141.2400	148.9000	148.6700	144.1800	149.3900
**C2**	106.8900	106.9000	105.7200	106.5900	106.9600	103.9900	106.5500
**C3**	119.5400	120.0600	118.9100	115.3200	115.4300	113.0900	115.6200
**C4**	146.2100	146.4800	144.2900	144.6100	144.9900	143.4400	145.4200
**C5**	122.6600	121.5000	127.0900	121.2300	121.7600	127.5700	121.4800
**C6**	56.5400	56.2900	50.9400	57.0300	56.4700	50.5400	56.5200
**C7**	126.3500	126.2100	127.0100	127.8500	128.0500	125.7400	127.3700
**C8**	124.7500	125.0000	122.9600	124.5400	124.9200	120.2500	125.2900
**C9**	114.2200	114.5600	115.8800	114.3400	114.5600	115.7600	113.7200
**C10**	151.5900	151.8400	152.2700	151.9800	151.8100	150.8700	151.5400
**C11**	58.1400	58.5400	60.2200	59.0100	58.8600	61.8400	59.5900
**C12**	67.4900	67.9400	65.5300	69.2900	69.3900	64.3200	69.0300
**C13**	148.4000	148.5900	146.5200	149.1900	149.7700	149.0900	149.8400
**C14**	-22.4600	-21.8200	-17.9900	-22.7600	-22.5300	-17.1300	-22.5300
**C15**	107.8500	108.4700	108.5400	109.0800	108.8800	104.1700	111.4300
**C16**	177.8700	178.0000	176.0200	178.1200	178.0100	170.1600	178.1100
**C17**	76.0100	75.2000	78.3400	74.4500	74.2000	84.3000	74.0400
**C18**	171.7600	171.8400	173.0800	172.7600	172.2000	172.4300	172.5300
**C19**	169.0800	168.7700	168.1900	169.7600	169.6200	168.9400	170.0900
**C20**	171.5300	171.5600	173.4500	171.1700	170.7900	175.2400	170.4800
**C21**	25.9400	25.8700	25.4800	26.6700	26.7400	23.1100	26.8700
**C22**	171.4100	171.6400	170.9900	171.7800	171.7000	171.3600	171.7900
**C23**	21.8400	23.5700	23.7900	21.5400	23.0300	22.7100	20.5900
**C24**	158.7800	158.2500	158.2800	157.7700	157.9400	157.8300	157.5900
**C25**	177.8200	176.8600	176.4100	178.1600	178.1700	178.0900	178.7300
**C26**	175.2700	177.6400	175.4900	174.4300	177.0500	176.5700	175.6100
**C27**	21.6100	22.4100	23.8800	21.6800	22.2300	22.7900	21.1100
**C28**	157.5300	157.0800	157.9900	157.3900	157.5900	157.7600	155.4700
**C29**	176.8700	177.4200	177.5000	176.7500	178.0200	177.7000	174.1200
**C30**	177.9500	177.4900	174.8900	177.8400	177.9300	175.9000	179.3200
**C31**	29.0400	29.3700	32.2900	31.3900	31.3400	30.8500	30.4700
**C32**	67.5500	66.9600	68.2000	65.9800	66.3400	70.5300	66.0200
**C33**	67.3500	67.7000	67.4100	68.8000	68.7400	66.6400	66.8100
**C34**	70.8200	70.5000	70.6600	69.6300	69.9100	71.3900	70.2000
**C35**	64.8000	64.9300	65.0400	65.1000	64.7800	64.5400	64.4300
**C36**	69.2100	69.1800	70.1400	69.9300	70.1400	71.1800	69.9700
**C37**	64.3600	64.3700	65.1000	67.0400	66.7300	66.0400	67.1900
**C38**	25.3500	24.9900	25.8700	25.2900	25.6900	26.7700	25.1900
**C39**	173.8000	173.7800	173.9800	173.5200	173.6600	174.0200	173.7000

**C40**	25.9200	26.1400	25.1700	25.8300	26.3500	26.1700	26.5200
**C41**	172.3500	173.1800	173.4900	172.9200	173.2300	174.4400	172.7300
**H1a**	28.9500	28.8600	28.8200	28.0900	28.0500	27.6900	27.9600
**H1b**	29.2300	29.2300	29.3800	29.3400	29.3900	29.8700	29.3100
**H3**	25.1400	24.8600	25.5500	26.2700	26.1800	25.1900	26.2400
**H4**	27.1000	27.1500	28.0100	27.0100	26.9700	27.2200	26.8100
**H5**	25.3600	25.5700	26.0200	25.0800	25.1200	25.9700	25.0300
**H7**	24.3500	24.5200	25.8300	24.6900	24.6400	26.3600	24.7800
**H8**	26.5000	26.4500	27.0900	26.5100	26.4900	27.0800	26.3700
**H9**	26.5100	26.3700	26.8100	26.5300	26.4500	26.6100	26.3900
**H11**	24.3800	24.5500	25.1800	24.6700	24.7000	24.9600	24.6900
**H12**	25.4700	25.4800	25.6900	25.5100	25.4800	25.0500	25.5700
**H13**	27.0000	27.0400	27.2000	27.6400	27.6000	26.4600	27.6000
**H16**	30.0267	30.0533	29.7933	30.0467	30.1500	30.2433	30.1133
**H17a**	26.1700	26.1100	25.3100	26.2600	26.1700	26.2900	26.2200
**H17b**	25.7900	25.7600	25.7300	25.7700	25.6100	26.0500	25.6700
**H18**	30.2867	30.2700	30.3567	30.2733	30.2800	30.2733	30.2833
**H19**	30.5167	30.5033	30.5600	30.6233	30.5867	30.5167	30.6167
**H20**	30.2033	30.1700	30.0367	30.2700	30.2200	30.1900	30.2767
**H22**	28.8267	28.8367	29.2400	29.0867	29.0300	29.1233	29.0067
**H24**	28.8100	29.1000	28.8400	28.8700	29.1200	28.9600	28.8200
**H25**	30.6600	30.0367	30.4733	30.5467	30.1333	30.6633	30.5467
**H26**	30.3767	30.6367	30.2900	30.4900	30.4033	30.2033	30.4900
**H28**	29.8700	29.8100	28.9000	29.7400	29.6600	28.7100	29.4400
**H29**	31.4600	31.4133	30.5867	31.2433	31.3133	30.4100	30.6767
**H30**	30.8333	30.8667	30.4467	30.7900	30.7600	30.3033	31.2800
**H33**	23.4700	23.3900	23.0700	22.6700	22.7800	23.0000	23.1500
**H34**	23.7200	23.7900	23.7200	23.6900	23.6900	23.6600	23.6700
**H35**	23.4800	23.5000	23.4900	23.4500	23.4500	23.6500	23.4000
**H36**	23.5100	23.4900	23.6600	23.5600	23.5200	23.8000	23.5400
**H37**	22.4600	22.5000	22.6300	22.7000	22.7200	23.0800	22.8200
**H39**	29.4033	29.3733	29.2900	29.4367	29.3900	29.3967	29.3600
**H41**	30.1433	30.1900	30.0700	30.4700	30.3933	30.0600	30.5300

Table S15. Isotropic magnetic shielding values computed for conformers 4-1 to 4-14 of euphodendroid L (**4**) at the PCM/B3LYP/6-31+G** level of theory.

**Atom (σ)**	**4-1**	**4-2**	**4-3**	**4-4**	**4-5**	**4-6**	**4-7**	**4-8**	**4-9**
**C1**	151.0610	142.2516	151.0222	142.5995	145.1203	144.4870	143.8349	143.8547	141.3394
**C2**	105.7877	105.1770	105.9015	104.9248	104.9747	103.7220	104.1622	104.1180	106.0830
**C3**	114.0519	118.2529	113.9438	118.4530	111.8871	111.7508	114.3533	114.3269	118.4058

**C4**	145.6457	143.8473	146.1207	144.0875	144.1657	144.5526	142.6624	142.8102	147.0031
**C5**	123.3923	126.7405	123.2031	127.1736	119.3832	119.5691	126.7835	127.3691	122.7866
**C6**	55.1501	51.5083	56.3674	51.3542	52.1591	53.4983	50.7052	50.8753	54.9757
**C7**	127.3375	125.0691	127.3258	125.0996	126.7154	126.2645	124.8274	125.1943	126.5458
**C8**	124.6101	122.6201	124.9378	122.6533	122.8167	123.1326	121.5392	121.6540	124.2548
**C9**	114.6440	115.5797	114.1667	115.5030	112.2346	113.1066	116.8506	116.4365	114.3045
**C10**	152.2712	152.5029	152.1756	152.2059	151.6596	151.9280	153.0140	153.2287	151.7840
**C11**	59.5283	60.7834	59.1669	60.7196	60.4024	60.6082	61.6903	61.2849	58.3926
**C12**	68.7191	66.7087	68.2471	66.5286	68.8406	68.7286	64.3148	63.9922	64.9559
**C13**	149.1358	147.5404	149.2427	147.6225	148.2846	148.7885	148.2034	148.5381	148.6220
**C14**	-20.3166	-19.2055	-20.5261	-19.0354	-20.6784	-21.6660	-17.0538	-16.8076	-19.9059
**C15**	110.3955	106.8263	110.3084	106.6378	109.2174	109.6132	105.3698	105.3857	107.6022
**C16**	177.7701	176.2261	177.7871	176.2013	169.3399	169.1787	169.7538	169.9661	177.9954
**C17**	75.4517	77.6125	75.1690	77.3177	75.8362	74.9300	85.5434	85.7165	74.8488
**C18**	173.2921	173.4145	173.0960	173.0818	172.8149	173.1468	170.0536	169.9840	172.3190
**C19**	170.1841	169.1066	170.0270	169.1115	170.4974	170.5038	168.1123	167.9824	169.8047
**C20**	171.1663	172.9367	171.1953	173.0110	172.2276	172.0235	174.6253	175.2767	171.2363
**C21**	27.1887	25.8920	26.9342	26.0225	25.9385	25.9459	24.0541	23.8682	25.7792
**C22**	172.0704	170.0567	172.0376	170.3645	172.8868	172.7883	171.1095	171.1090	171.6425
**C23**	26.8446	29.0827	26.8839	29.2019	27.4252	27.5237	26.8612	27.1829	27.4942
**C24**	173.1688	172.2556	173.2112	172.3829	173.8688	173.9919	172.8949	173.0119	173.8684
**C25**	23.6734	23.2038	23.6445	22.9927	22.0054	21.7546	22.7650	23.2901	22.6665
**C26**	157.7821	158.5306	157.8572	158.6440	158.6232	158.3294	158.2748	158.1996	157.3190
**C27**	176.7876	178.1610	177.2387	178.1489	175.8244	175.9352	175.1656	177.5945	176.3903
**C28**	177.9369	175.5123	177.8426	175.4514	178.3489	178.5579	177.6994	175.1789	178.2336
**C29**	31.8209	31.6292	30.8682	31.9081	32.7011	33.5263	33.3063	32.9101	30.1865
**C30**	67.0615	68.6872	67.1409	68.6462	66.3349	66.0082	67.9463	68.0506	66.5105
**C31**	67.7763	65.6941	67.9404	65.5999	66.5237	66.1985	67.1798	67.5909	67.9245
**C32**	70.1183	69.6642	70.1718	69.5434	70.9251	70.7854	70.5060	70.1225	70.6905
**C33**	64.4596	64.6795	64.5624	64.9011	64.8727	65.0577	64.5666	64.5517	64.8534
**C34**	70.1629	70.5221	70.2265	70.5028	70.1274	70.2126	70.0770	70.5491	68.7860
**C35**	66.4256	68.2073	67.0163	68.1069	65.7781	65.8944	67.6360	67.2748	64.4363
**C36**	25.6211	24.6110	24.6021	24.5486	26.1609	26.4328	26.6274	26.8117	24.8114
**C37**	173.6529	173.8831	173.7853	173.8399	173.7409	173.6570	174.4644	174.3826	173.9100
**C38**	25.7536	26.0199	26.3063	25.9493	25.9504	25.8467	25.9261	26.0188	26.1766
**C39**	172.7020	172.6892	173.1685	172.7133	172.8720	172.8807	172.9572	173.3718	173.0038
**H1a**	29.4036	29.4170	29.4164	29.3884	29.9215	29.9860	29.8828	29.8206	29.3365
**H1b**	27.8895	28.7474	27.9342	28.7062	27.9567	28.0536	27.6240	27.6652	28.9825
**H3**	26.3990	25.6089	26.4301	25.6298	25.7817	25.8159	25.1715	25.2101	25.2556
**H4**	26.9654	27.8031	26.9651	27.8764	26.3258	26.2592	26.9807	26.9180	27.2244
**H5**	25.1213	25.9755	25.1385	25.9835	25.0802	25.1734	25.9399	25.9083	25.1953

**H7**	24.4902	25.7270	24.3617	25.6433	25.1186	25.1191	26.3711	26.2032	24.3749
**H8**	26.4139	26.9572	26.3461	26.9362	26.5160	26.4944	27.1139	27.0465	26.4772
**H9**	26.3602	26.4796	26.2777	26.4689	26.9430	26.8285	26.7113	26.7135	26.4934
**H11**	24.6220	25.0525	24.4952	24.9942	24.9239	24.9596	25.1929	24.9835	24.5015
**H12**	25.6064	25.6975	25.6808	25.7158	25.3592	25.3571	25.2584	25.2730	25.4787
**H13**	27.7132	27.1104	27.6480	27.1742	26.9702	26.8923	26.2531	26.4115	26.9923
**H16**	30.0605	29.7515	30.0653	29.7495	30.1219	30.0533	30.2128	30.2255	29.9355
**H17a**	26.1657	25.3477	26.1417	25.2761	26.0881	26.0500	26.1380	26.1095	26.1349
**H17b**	25.7119	25.7068	25.6984	25.7191	25.4851	25.5236	25.9088	25.8577	25.8756
**H18**	30.2923	30.2344	30.2970	30.2400	30.1794	30.2089	30.3669	30.3202	30.3440
**H19**	30.5771	30.5084	30.6006	30.4895	30.5810	30.5872	30.5220	30.5330	30.5666
**H20**	30.2282	30.0904	30.2500	30.1187	30.3102	30.3382	30.2099	30.2334	30.1577
**H22**	29.0513	29.2098	28.9891	29.1876	29.2377	29.1525	29.0960	29.1071	28.8501
**H24**	29.4964	29.4440	29.5142	29.4496	29.5990	29.5983	29.4065	29.4302	29.5671
**H26**	29.6797	28.9892	29.5338	28.9667	30.0589	29.8856	28.7664	28.7709	29.9257
**H27**	31.4032	30.5441	31.4397	30.5505	31.2619	31.3137	30.3235	30.4805	31.5757
**H28**	30.8293	30.3992	30.7692	30.3913	30.9411	30.8973	30.4861	30.3118	30.9052
**H31**	22.9898	22.5858	22.9687	22.6069	23.0028	22.8572	22.9964	22.9332	23.2903
**H32**	23.6726	23.6625	23.6796	23.6781	23.7254	23.7072	23.6964	23.6853	23.8036
**H33**	23.4749	23.5054	23.4324	23.5147	23.6130	23.6202	23.5194	23.5266	23.5315
**H34**	23.5156	23.6375	23.4866	23.6480	23.7686	23.8169	23.6871	23.6637	23.5332
**H35**	22.7108	23.1046	22.6732	23.1020	22.7695	22.8468	22.9205	22.9921	22.3955
**H37**	29.4212	29.3407	29.4321	29.3290	29.3383	29.3125	29.3861	29.4019	29.3916
**H39**	30.4151	30.0648	30.3997	30.0952	30.5823	30.6226	30.2183	30.2382	30.0806

Atom (σ)	4-10	4-11	4-12	4-13	4-14
**C1**	141.4953	140.8587	140.8937	142.5261	144.2209
**C2**	105.8929	106.4258	106.3993	105.1806	104.4457
**C3**	118.0097	117.1987	117.1328	117.1013	117.1943
**C4**	147.2791	144.3703	144.2592	144.8830	143.2900
**C5**	122.3943	124.7679	124.9045	127.0166	127.2240
**C6**	55.0838	51.4263	51.3415	50.6273	55.3353
**C7**	126.1559	124.7215	124.6346	125.5208	122.9220
**C8**	124.5049	122.1573	122.2870	122.2877	124.7539
**C9**	114.9148	116.1348	116.0482	116.1861	116.3899
**C10**	151.6225	152.3692	152.3450	152.0931	149.9637
**C11**	58.3883	60.0806	60.2875	60.9717	62.1361
**C12**	65.7607	66.2160	65.9689	64.4297	58.9245
**C13**	148.3858	147.8779	147.7541	147.6132	147.0014
**C14**	-20.3141	-19.5826	-19.6637	-18.4961	-17.2485
**C15**	108.2215	105.8159	105.7690	106.9835	108.3328

**C16**	178.0026	176.9969	176.9058	176.4500	175.7323
**C17**	74.2387	78.4577	78.5135	76.9824	81.6360
**C18**	172.6105	173.1346	173.2202	173.7106	174.0210
**C19**	169.9185	169.0317	168.9780	168.6532	170.1007
**C20**	171.4587	173.0556	173.0516	173.2705	174.3844
**C21**	25.6880	25.6447	25.7113	26.0565	28.7991
**C22**	171.6535	170.0497	170.0148	171.0672	170.5174
**C23**	27.9727	28.3493	28.4451	28.7544	28.3344
**C24**	173.7071	171.8279	171.8550	171.9651	171.6793
**C25**	22.0915	22.8646	22.9242	24.1106	23.3898
**C26**	157.1894	156.0608	156.0629	155.7981	155.8114
**C27**	176.5643	176.0653	176.0756	176.1564	176.2595
**C28**	178.2767	175.4056	175.4643	174.4389	173.7953
**C29**	30.4947	31.8399	31.7901	32.2177	33.4400
**C30**	66.1685	68.3414	68.2096	68.6701	68.6472
**C31**	68.2003	68.0388	65.8049	65.1329	65.5768
**C32**	70.5278	70.3986	70.1221	69.4673	72.0460
**C33**	64.7947	64.6025	64.5697	65.0622	63.1890
**C34**	68.9447	70.0994	70.3413	70.6620	72.4365
**C35**	64.5334	65.8020	68.0230	68.1486	64.7281
**C36**	24.9657	24.8334	24.7881	24.6353	27.1589
**C37**	173.6273	173.8679	173.8180	173.7316	174.7663
**C38**	26.2503	26.3348	26.3822	25.3520	27.4375
**C39**	172.7952	171.9042	171.6230	172.4392	173.3729
**H1a**	29.3343	29.5219	29.5147	29.3924	29.4470
**H1b**	28.9479	28.9967	28.9618	28.7324	28.8050
**H3**	25.2573	25.4628	25.4603	25.7814	25.4254
**H4**	27.2125	27.5295	27.5401	27.9523	28.4113
**H5**	25.2507	26.4272	26.4215	26.1046	25.8394
**H7**	24.4206	25.9400	25.9728	25.6639	25.9904
**H8**	26.3944	26.7487	26.7674	26.9968	26.5442
**H9**	26.3369	26.4121	26.4281	26.5526	26.4768
**H11**	24.5298	25.1524	25.1861	25.0139	25.3232
**H12**	25.4553	25.7005	25.6629	25.6513	25.3352
**H13**	26.9889	27.0011	27.0183	27.1815	27.0300
**H16**	29.9419	29.8535	29.8420	29.7622	29.5710
**H17a**	26.0802	25.7038	25.6829	25.2669	25.7452
**H17b**	25.8596	25.7694	25.7341	25.5561	25.9299
**H18**	30.3310	30.1837	30.1837	30.2659	30.5978
**H19**	30.5859	30.4635	30.4718	30.5059	30.6819
**H20**	30.1558	30.1437	30.1364	30.1172	30.1677

**H22**	28.8699	29.2190	29.2319	29.1926	29.1607
**H24**	29.6216	29.2992	29.2958	29.3379	29.3130
**H26**	29.8721	29.2107	29.2081	29.1996	28.9478
**H27**	31.6081	30.4538	30.4469	30.4563	30.4104
**H28**	30.8789	30.2181	30.2140	30.2041	30.1606
**H31**	23.2672	23.1859	22.6649	22.6530	22.9266
**H32**	23.8365	23.7088	23.7074	23.6768	23.7049
**H33**	23.5240	23.4583	23.4429	23.4701	23.7538
**H34**	23.5218	23.6998	23.7094	23.6868	23.6413
**H35**	22.3549	22.6523	23.2044	23.1470	22.9955
**H37**	29.3846	29.3730	29.3742	29.3253	29.5164
**H39**	30.0917	29.9540	29.9435	30.0824	30.0475

Table S16. Isotropic magnetic shielding values computed for conformers 5-1 to 5-9 of longilene peroxide (**5**) at the PCM/B3LYP/6-31+G** level of theory.

**Atom (σ)**	**5-1**	**5-2**	**5-3**	**5-4**	**5-5**	**5-6**	**5-7**	**5-8**	**5-9**
**C12**	164.0226	164.6787	164.8885	164.0708	164.1445	164.3799	164.5557	164.5743	164.3543
**C11**	110.3924	107.2235	107.3271	110.6813	109.1499	110.2407	107.5237	108.0012	107.0316
**C14**	109.3463	112.2614	111.8629	110.6893	107.8268	107.2549	111.3750	112.6964	111.1812
**C13**	164.5749	163.7485	163.4748	164.0906	164.5066	164.4972	163.9027	163.3376	163.5321
**C8**	167.7914	166.9250	166.8667	169.5777	166.0484	168.7238	167.1159	168.0004	167.3795
**C7**	111.3214	108.9300	108.8108	111.5661	111.4581	110.9817	109.0681	109.2234	109.3658
**C10**	110.5067	109.6153	107.2419	110.5360	109.6062	108.2822	109.2426	107.6927	108.3925
**C9**	164.5374	161.8357	161.9324	164.4857	162.4540	165.1556	161.9293	161.5185	160.7642
**C16**	164.4798	165.6690	167.8146	165.8032	160.8144	162.2628	164.2164	167.1544	164.3961
**C15**	108.0678	109.0985	108.6717	107.0397	110.6315	108.8637	110.8594	110.0726	110.7423
**C18**	109.3321	111.4501	112.8329	108.2907	110.8462	108.5948	111.6726	110.9478	112.6685
**C17**	167.4901	170.2261	170.5168	169.2034	162.6820	167.9550	167.3438	167.0041	167.6174
**C6**	120.2709	118.9037	118.6433	119.5687	120.3902	120.0610	118.8514	118.7238	117.8007
**C19**	117.5952	120.6744	119.8853	116.5041	122.8093	118.3016	120.8210	120.2892	121.4839
**C28**	171.7435	174.1466	176.0119	172.0084	171.4884	170.1148	173.1827	175.4326	173.2777
**C27**	172.7885	170.4284	170.4237	171.5849	171.6473	173.2530	169.9037	171.0028	169.5918
**C20**	154.9078	151.3764	152.4536	155.9036	152.1577	152.0468	149.4652	148.7950	148.9398
**C21**	71.9369	74.3333	75.2194	71.1489	71.5418	73.7382	68.9428	69.0227	70.1035
**C22**	57.1237	55.3303	54.7407	55.9757	60.6257	55.7462	60.9077	60.2920	60.3361
**C23**	122.3287	126.2055	123.6264	123.0045	122.2725	124.6921	124.6599	124.2935	122.9439
**C24**	165.9447	170.3192	165.7758	166.9382	166.8162	170.0987	167.6026	169.2077	164.9004
**C30**	166.5143	165.7758	165.9819	171.2457	166.0103	168.0688	169.4304	167.9173	168.7676
**C29**	173.5625	173.2575	172.8874	173.5698	165.9304	172.6721	168.9071	167.0790	167.9525
**C4**	71.4713	66.5886	66.5352	72.3446	71.4981	67.8225	67.6731	70.4305	71.0100

**C5**	151.6681	152.9932	153.2577	150.5432	153.1783	150.0864	153.2124	152.4453	151.3165
**C2**	113.1559	114.8350	113.4206	114.6114	113.5214	115.5302	115.0110	114.8838	114.7820
**C3**	59.6269	62.2032	62.8082	60.5622	59.4026	59.3508	60.0501	59.6910	59.7232
**C26**	171.8939	172.9846	173.3112	172.2484	170.8858	172.5454	173.2414	173.0424	172.2426
**C25**	171.1479	174.5708	175.0006	173.6143	171.3392	174.5377	174.8601	175.2859	175.0759
**C1**	166.8115	168.9328	168.7456	171.0630	167.5897	168.2796	168.5317	169.1394	168.8184
**H12b**	29.6944	29.7399	29.7484	29.6541	29.7434	29.5607	29.7399	29.7153	29.7192
**H12a**	30.0813	30.0025	29.9799	30.0010	30.1244	29.9281	30.0186	29.9594	29.9885
**H11**	27.3344	27.3491	27.3155	27.4185	27.4224	27.3117	27.3485	27.3202	27.3082
**H14**	27.1687	27.2294	27.2291	27.1232	27.3823	27.3606	27.2706	27.1902	27.2789
**H13b**	29.6195	29.5077	29.5788	29.7668	29.7459	29.7230	29.5556	29.5244	29.5273
**H13a**	30.0322	29.9408	29.9158	30.1045	30.1476	30.0033	29.9704	29.8667	29.9113
**H8a**	29.9052	29.8613	29.8634	29.8834	29.9094	30.0111	29.8455	29.8055	29.7605
**H8b**	29.6172	29.1002	29.1963	29.5148	29.3440	29.6831	29.0542	28.9815	28.9161
**H7**	27.8919	27.5151	27.4938	27.9914	27.8294	27.8678	27.4873	27.4920	27.3768
**H9b**	29.3568	29.3586	29.2612	29.4835	29.3785	29.4963	29.3416	29.3120	29.5094
**H9a**	30.1833	29.9481	29.8473	30.2786	30.1450	30.0655	29.9251	29.9432	30.0074
**H16a**	30.0348	30.1056	30.0108	30.0337	30.1696	29.8491	30.1988	30.0276	30.1752
**H16b**	29.3685	29.5777	29.5501	29.3409	29.5093	29.3188	29.6192	29.6319	29.4943
**H18**	27.6058	27.7176	27.7777	27.6399	27.7299	27.4620	27.7592	27.6336	27.7984
**H17b**	29.2551	29.3902	29.2500	29.2324	29.6943	29.0782	29.6391	29.3888	29.4974
**H17a**	29.4534	29.8950	29.9008	29.2543	30.0215	29.8044	30.0113	29.9833	29.6626
**$H_3$28**	30.5290	30.5925	30.5542	30.5648	30.5372	30.4549	30.6287	30.6351	30.6059
**$H_3$27**	30.5558	30.5105	30.4698	30.6287	30.5149	30.6475	30.5308	30.5467	30.4904
**H20a**	30.0033	29.5339	29.5617	30.0513	28.9763	29.6653	28.9189	28.9766	28.8672
**H20b**	29.2959	29.5134	29.5331	29.3055	29.5617	29.6800	29.6025	29.6131	29.4866
**H21**	25.1082	25.3781	25.0725	25.4756	24.5691	25.4685	25.3710	25.2998	25.1496
**H22**	25.4104	25.5423	25.5408	25.3884	25.7195	25.6128	25.7000	25.7271	25.5632
**$H_3$24**	30.3056	30.4317	30.4801	30.1868	30.4567	30.1306	30.3188	30.3278	30.4255
**$H_3$30**	30.0717	30.0927	30.2131	29.9582	30.3299	30.3754	30.3922	30.4103	30.3781
**$H_3$29**	30.4211	30.1053	30.0310	30.4280	30.3514	30.2028	30.1558	30.1313	30.1205
**H4**	25.0421	25.2515	25.3313	25.2415	24.5598	25.3155	25.2651	25.3193	25.2365
**H5a**	29.7795	29.6045	29.6254	29.4998	29.7787	29.5637	29.5992	29.6339	29.6371
**H5b**	29.1615	29.5455	29.6155	29.4099	28.9530	29.4451	29.5300	29.5057	29.4694
**H3**	25.6008	25.6646	25.7155	25.1372	25.6996	25.7545	25.6230	25.6465	25.5752
**$H_3$26**	30.2860	30.2831	30.2804	30.1416	30.3622	30.2700	30.2264	30.1657	30.1083
**$H_3$25**	30.1371	30.1962	30.1365	30.3127	30.1880	30.1955	30.1511	30.2040	30.1824
**$H_3$1**	30.4513	30.3788	30.3642	30.2379	30.4497	30.4005	30.3898	30.4958	30.4966

4. Combination of conformations for compound 5.

Table S17. The calculated values of isotropic magnetic shielding and selected $^{3}J_{H,H}$ for the 255 possible combinations of the comformers 5-1, 5-2, 5-5 and 5-9 at the B3LYP/6-31+G** level using molar fraction steps of 0.1.

**Conformer 5-1**	**100%**	**90%**	**90%**	**90%**	**80%**	**80%**	**80%**	**80%**
**Conformer 5-2**	**0%**	**10%**	**0%**	**0%**	**20%**	**10%**	**10%**	**0%**
**Conformer 5-5**	**0%**	**0%**	**10%**	**0%**	**0%**	**10%**	**0%**	**20%**
**Conformer 5-9**	**0%**	**0%**	**0%**	**10%**	**0%**	**0%**	**10%**	**0%**
**σC12**	164.0226	164.0883	164.0348	164.0558	164.1539	164.1004	164.1214	164.0470
**σC11**	110.3924	110.0755	110.2682	110.0563	109.7586	109.9513	109.7394	110.1439
**σC14**	109.3463	109.6378	109.1943	109.5298	109.9293	109.4859	109.8213	109.0424
**σC13**	164.5749	164.4923	164.5681	164.4706	164.4096	164.4854	164.3880	164.5612
**σC8**	167.7914	167.7048	167.6171	167.7502	167.6181	167.5305	167.6636	167.4428
**σC7**	111.3214	111.0822	111.3350	111.1258	110.8431	111.0959	110.8867	111.3487
**σC10**	110.5067	110.4176	110.4167	110.2953	110.3284	110.3275	110.2062	110.3266
**σC9**	164.5374	164.2672	164.3291	164.1601	163.9971	164.0589	163.8899	164.1207
**σC16**	164.4798	164.5987	164.1133	164.4714	164.7176	164.2322	164.5904	163.7467
**σC15**	108.0678	108.1708	108.3241	108.3352	108.2739	108.4272	108.4383	108.5805
**σC18**	109.3321	109.5439	109.4835	109.6658	109.7557	109.6953	109.8776	109.6349
**σC17**	167.4901	167.7637	167.0093	167.5029	168.0373	167.2829	167.7764	166.5285
**σC6**	120.2709	120.1341	120.2828	120.0238	119.9974	120.1461	119.8871	120.2947
**σC19**	117.5952	117.9031	118.1166	117.9841	118.2110	118.4245	118.2920	118.6380
**σC28**	171.7435	171.9838	171.7180	171.8970	172.2241	171.9583	172.1373	171.6925
**σC27**	172.7885	172.5525	172.6744	172.4688	172.3165	172.4384	172.2328	172.5602
**σC20**	154.9078	154.5546	154.6328	154.3110	154.2015	154.2796	153.9578	154.3578
**σC21**	71.9369	72.1765	71.8974	71.7535	72.4162	72.1370	71.9932	71.8579
**σC22**	57.1237	56.9444	57.4739	57.4449	56.7650	57.2946	57.2656	57.8241
**σC23**	122.3287	122.7163	122.3230	122.3902	123.1040	122.7107	122.7779	122.3174
**σC24**	165.9447	166.3822	166.0319	165.8403	166.8196	166.4693	166.2777	166.1190
**σC30**	166.5143	166.4405	166.4639	166.7396	166.3666	166.3901	166.6658	166.4135
**σC29**	173.5625	173.5320	172.7993	173.0015	173.5015	172.7688	172.9710	172.0360
**σC4**	71.4713	70.9830	71.4740	71.4252	70.4947	70.9857	70.9369	71.4767
**σC5**	151.6681	151.8006	151.8192	151.6330	151.9332	151.9517	151.7655	151.9702
**σC2**	113.1559	113.3238	113.1924	113.3185	113.4917	113.3603	113.4864	113.2290
**σC3**	59.6269	59.8845	59.6045	59.6365	60.1422	59.8621	59.8942	59.5820
**σC26**	171.8939	172.0030	171.7931	171.9288	172.1121	171.9022	172.0379	171.6923
**σC1**	171.1479	171.4902	171.1670	171.5407	171.8325	171.5093	171.8830	171.1862
**σC25**	166.8115	167.0236	166.8893	167.0122	167.2357	167.1014	167.2243	166.9671
**σH12a**	29.6944	29.6989	29.6993	29.6969	29.7035	29.7038	29.7014	29.7042
**σH12b**	30.0813	30.0734	30.0856	30.0720	30.0655	30.0777	30.0641	30.0899
**σH11**	27.3344	27.3359	27.3432	27.3318	27.3373	27.3447	27.3332	27.3520
**σH14**	27.1687	27.1748	27.1900	27.1797	27.1808	27.1961	27.1858	27.2114
**σH13a**	29.6195	29.6084	29.6322	29.6103	29.5972	29.6210	29.5991	29.6448
**σH13b**	30.0322	30.0230	30.0437	30.0201	30.0139	30.0346	30.0109	30.0552
**σH8a**	29.9052	29.9008	29.9056	29.8907	29.8964	29.9012	29.8863	29.9060
**σH8b**	29.6172	29.5655	29.5899	29.5471	29.5138	29.5382	29.4954	29.5626
**σH7**	27.8919	27.8542	27.8857	27.8404	27.8165	27.8480	27.8027	27.8794
**σH9a**	29.3568	29.3570	29.3590	29.3721	29.3572	29.3592	29.3722	29.3611
**σH9b**	30.1833	30.1598	30.1795	30.1657	30.1363	30.1560	30.1422	30.1757

**σH16a**	30.0348	30.0418	30.0482	30.0488	30.0489	30.0553	30.0559	30.0617
**σH16b**	29.3685	29.3894	29.3826	29.3811	29.4104	29.4035	29.4020	29.3967
**σH18**	27.6058	27.6170	27.6182	27.6251	27.6282	27.6294	27.6363	27.6306
**σH17a**	29.2551	29.2686	29.2990	29.2793	29.2821	29.3125	29.2928	29.3429
**σH17b**	29.4534	29.4975	29.5102	29.4743	29.5417	29.5544	29.5185	29.5670
**σ$H_3$28**	30.5290	30.5354	30.5298	30.5367	30.5417	30.5362	30.5431	30.5307
**σ$H_3$27**	30.5558	30.5513	30.5518	30.5493	30.5468	30.5472	30.5448	30.5477
**σH20a**	30.0033	29.9564	29.9006	29.8897	29.9094	29.8537	29.8427	29.7979
**σH20b**	29.2959	29.3176	29.3225	29.3149	29.3394	29.3442	29.3367	29.3490
**σH21**	25.1082	25.1352	25.0543	25.1123	25.1622	25.0813	25.1393	25.0004
**σH22**	25.4104	25.4236	25.4414	25.4257	25.4368	25.4545	25.4389	25.4723
**σ$H_3$24**	30.3056	30.3182	30.3207	30.3176	30.3308	30.3333	30.3302	30.3358
**σ$H_3$30**	30.0717	30.0738	30.0975	30.1024	30.0759	30.0996	30.1045	30.1234
**σ$H_3$29**	30.4211	30.3895	30.4141	30.3910	30.3579	30.3826	30.3595	30.4072
**σH4**	25.0421	25.0631	24.9939	25.0616	25.0840	25.0148	25.0825	24.9457
**σH5a**	29.7795	29.7620	29.7794	29.7653	29.7445	29.7619	29.7478	29.7793
**σH5b**	29.1615	29.1999	29.1407	29.1923	29.2383	29.1791	29.2307	29.1198
**σH3**	25.6008	25.6072	25.6107	25.5983	25.6136	25.6171	25.6046	25.6206
**σ$H_3$26**	30.2860	30.2857	30.2936	30.2682	30.2854	30.2933	30.2679	30.3012
**σ$H_3$1**	30.1371	30.1430	30.1422	30.1416	30.1489	30.1481	30.1475	30.1472
**σ$H_3$25**	30.4513	30.4441	30.4512	30.4559	30.4368	30.4439	30.4486	30.4510
$^3J_{H4,H5}$	9.70	9.27	9.69	9.27	8.84	9.26	8.84	9.68
$^3J_{H4',H5}$	4.21	4.68	4.22	4.66	5.16	4.70	5.13	4.23
$^3J_{H20,H21}$	9.79	9.22	9.20	9.19	8.66	8.64	8.62	8.62
$^3J_{H20',H21}$	4.39	4.90	4.89	4.89	5.40	5.39	5.39	5.38

**Conformer 5-1**	**80%**	**80%**	**70%**	**70%**	**70%**	**70%**	**70%**	**70%**
**Conformer 5-2**	**0%**	**0%**	**30%**	**20%**	**10%**	**10%**	**10%**	**0%**
**Conformer 5-5**	**10%**	**0%**	**0%**	**10%**	**20%**	**10%**	**0%**	**30%**
**Conformer 5-9**	**10%**	**20%**	**0%**	**0%**	**0%**	**10%**	**20%**	**0%**
**σC12**	164.0680	164.0890	164.2195	164.1660	164.1126	164.1336	164.1546	164.0592
**σC11**	109.9321	109.7202	109.4417	109.6344	109.8270	109.6152	109.4033	110.0197
**σC14**	109.3778	109.7133	110.2208	109.7774	109.3339	109.6694	110.0048	108.8905
**σC13**	164.4638	164.3663	164.3270	164.4028	164.4786	164.3812	164.2837	164.5544
**σC8**	167.5759	167.7090	167.5315	167.4438	167.3562	167.4893	167.6224	167.2685
**σC7**	111.1395	110.9302	110.6040	110.8568	111.1096	110.9003	110.6911	111.3624
**σC10**	110.2052	110.0839	110.2393	110.2384	110.2375	110.1161	109.9947	110.2366
**σC9**	163.9517	163.7828	163.7269	163.7887	163.8506	163.6816	163.5126	163.9124
**σC16**	164.1049	164.4631	164.8366	164.3511	163.8656	164.2238	164.5820	163.3802
**σC15**	108.5916	108.6027	108.3770	108.5303	108.6836	108.6947	108.7057	108.8369
**σC18**	109.8172	109.9994	109.9675	109.9071	109.8467	110.0290	110.2112	109.7864
**σC17**	167.0220	167.5156	168.3109	167.5565	166.8021	167.2956	167.7892	166.0477
**σC6**	120.0358	119.7768	119.8607	120.0094	120.1580	119.8991	119.6401	120.3067
**σC19**	118.5055	118.3729	118.5190	118.7325	118.9459	118.8134	118.6809	119.1594
**σC28**	171.8714	172.0504	172.4644	172.1986	171.9328	172.1117	172.2907	171.6670
**σC27**	172.3547	172.1492	172.0805	172.2024	172.3242	172.1187	171.9131	172.4461
**σC20**	154.0360	153.7142	153.8483	153.9265	154.0046	153.6828	153.3610	154.0828
**σC21**	71.7140	71.5702	72.6558	72.3767	72.0975	71.9537	71.8098	71.8184

**σC22**	57.7951	57.7662	56.5857	57.1152	57.6448	57.6158	57.5868	58.1743
**σC23**	122.3846	122.4517	123.4917	123.0984	122.7051	122.7723	122.8394	122.3118
**σC24**	165.9274	165.7359	167.2571	166.9068	166.5565	166.3649	166.1733	166.2062
**σC30**	166.6892	166.9650	166.2927	166.3162	166.3397	166.6154	166.8911	166.3631
**σC29**	172.2383	172.4405	173.4710	172.7383	172.0056	172.2078	172.4100	171.2728
**σC4**	71.4278	71.3790	70.0065	70.4974	70.9884	70.9396	70.8908	71.4793
**σC5**	151.7840	151.5978	152.0657	152.0842	152.1027	151.9165	151.7303	152.1212
**σC2**	113.3550	113.4811	113.6596	113.5282	113.3969	113.5229	113.6490	113.2655
**σC3**	59.6141	59.6462	60.3998	60.1197	59.8397	59.8717	59.9038	59.5596
**σC26**	171.8280	171.9637	172.2211	172.0112	171.8014	171.9371	172.0727	171.5915
**σC1**	171.5598	171.9335	172.1748	171.8516	171.5284	171.9021	172.2758	171.2053
**σC25**	167.0900	167.2129	167.4479	167.3136	167.1793	167.3021	167.4250	167.0450
**σH12a**	29.7018	29.6993	29.7080	29.7084	29.7087	29.7063	29.7039	29.7091
**σH12b**	30.0763	30.0627	30.0576	30.0698	30.0820	30.0684	30.0548	30.0942
**σH11**	27.3406	27.3292	27.3388	27.3461	27.3535	27.3420	27.3306	27.3608
**σH14**	27.2011	27.1907	27.1869	27.2022	27.2175	27.2071	27.1968	27.2328
**σH13a**	29.6229	29.6011	29.5860	29.6098	29.6336	29.6118	29.5899	29.6574
**σH13b**	30.0316	30.0080	30.0047	30.0254	30.0461	30.0225	29.9988	30.0668
**σH8a**	29.8911	29.8762	29.8920	29.8968	29.9016	29.8867	29.8719	29.9064
**σH8b**	29.5198	29.4770	29.4621	29.4865	29.5109	29.4681	29.4253	29.5353
**σH7**	27.8342	27.7889	27.7789	27.8103	27.8417	27.7965	27.7512	27.8732
**σH9a**	29.3742	29.3873	29.3573	29.3593	29.3613	29.3744	29.3875	29.3633
**σH9b**	30.1619	30.1481	30.1128	30.1325	30.1521	30.1384	30.1246	30.1718
**σH16a**	30.0623	30.0628	30.0560	30.0624	30.0688	30.0694	30.0699	30.0752
**σH16b**	29.3952	29.3937	29.4313	29.4244	29.4176	29.4161	29.4146	29.4108
**σH18**	27.6375	27.6443	27.6394	27.6406	27.6418	27.6487	27.6555	27.6431
**σH17a**	29.3233	29.3036	29.2956	29.3260	29.3565	29.3368	29.3171	29.3869
**σH17b**	29.5311	29.4952	29.5859	29.5985	29.6112	29.5753	29.5394	29.6238
**σ$H_3$28**	30.5375	30.5444	30.5481	30.5425	30.5370	30.5439	30.5508	30.5315
**σ$H_3$27**	30.5452	30.5428	30.5422	30.5427	30.5431	30.5407	30.5382	30.5436
**σH20a**	29.7870	29.7761	29.8625	29.8067	29.7510	29.7400	29.7291	29.6952
**σH20b**	29.3415	29.3340	29.3611	29.3660	29.3708	29.3633	29.3558	29.3756
**σH21**	25.0584	25.1165	25.1892	25.1083	25.0274	25.0854	25.1435	24.9465
**σH22**	25.4566	25.4410	25.4500	25.4677	25.4854	25.4698	25.4542	25.5032
**σ$H_3$24**	30.3327	30.3296	30.3434	30.3459	30.3484	30.3453	30.3422	30.3509
**σ$H_3$30**	30.1282	30.1330	30.0780	30.1017	30.1255	30.1303	30.1351	30.1492
**σ$H_3$29**	30.3841	30.3610	30.3264	30.3510	30.3756	30.3525	30.3294	30.4002
**σH4**	25.0133	25.0810	25.1049	25.0358	24.9666	25.0343	25.1019	24.8974
**σH5a**	29.7652	29.7510	29.7270	29.7444	29.7618	29.7477	29.7335	29.7793
**σH5b**	29.1715	29.2231	29.2767	29.2175	29.1582	29.2099	29.2615	29.0990
**σH3**	25.6082	25.5957	25.6200	25.6235	25.6270	25.6145	25.6021	25.6305
**σ$H_3$26**	30.2758	30.2504	30.2851	30.2930	30.3009	30.2755	30.2501	30.3088
**σ$H_3$1**	30.1467	30.1461	30.1548	30.1540	30.1532	30.1526	30.1520	30.1523
**σ$H_3$25**	30.4557	30.4604	30.4296	30.4367	30.4437	30.4484	30.4531	30.4508
$^3J_{H4,H5}$	9.26	8.84	8.41	8.83	9.25	8.83	8.41	9.67
$^3J_{H4',H5}$	4.67	5.11	5.64	5.17	4.71	5.15	5.58	4.25
$^3J_{H20,H21}$	8.60	8.58	8.09	8.07	8.05	8.03	8.02	8.03
$^3J_{H20',H21}$	5.38	5.38	5.90	5.89	5.88	5.88	5.88	5.87

**Conformer 5-1**	**70%**	**70%**	**70%**	**60%**	**60%**	**60%**	**60%**	**60%**
**Conformer 5-2**	**0%**	**0%**	**0%**	**40%**	**30%**	**30%**	**20%**	**20%**
**Conformer 5-5**	**20%**	**10%**	**0%**	**0%**	**10%**	**0%**	**20%**	**10%**
**Conformer 5-9**	**10%**	**20%**	**30%**	**0%**	**0%**	**10%**	**0%**	**10%**
**σC12**	164.0802	164.1012	164.1221	164.2851	164.2317	164.2526	164.1782	164.1992
**σC11**	109.8078	109.5960	109.3842	109.1248	109.3175	109.1056	109.5101	109.2983
**σC14**	109.2259	109.5613	109.8968	110.5123	110.0689	110.4043	109.6254	109.9609
**σC13**	164.4570	164.3595	164.2621	164.2443	164.3202	164.2227	164.3960	164.2985
**σC8**	167.4016	167.5347	167.6678	167.4449	167.3572	167.4903	167.2695	167.4026
**σC7**	111.1532	110.9439	110.7347	110.3648	110.6176	110.4084	110.8704	110.6612
**σC10**	110.1152	109.9938	109.8725	110.1501	110.1492	110.0279	110.1483	110.0270
**σC9**	163.7434	163.5744	163.4054	163.4567	163.5186	163.3496	163.5804	163.4114
**σC16**	163.7383	164.0965	164.4547	164.9555	164.4700	164.8282	163.9846	164.3427
**σC15**	108.8480	108.8590	108.8701	108.4801	108.6334	108.6444	108.7867	108.7977
**σC18**	109.9686	110.1508	110.3330	110.1793	110.1189	110.3012	110.0585	110.2408
**σC17**	166.5412	167.0348	167.5283	168.5845	167.8301	168.3236	167.0757	167.5692
**σC6**	120.0477	119.7888	119.5298	119.7240	119.8726	119.6137	120.0213	119.7623
**σC19**	119.0269	118.8943	118.7618	118.8269	119.0404	118.9078	119.2539	119.1213
**σC28**	171.8459	172.0249	172.2038	172.7047	172.4389	172.6179	172.1731	172.3520
**σC27**	172.2406	172.0350	171.8295	171.8445	171.9663	171.7608	172.0882	171.8827
**σC20**	153.7610	153.4392	153.1174	153.4952	153.5733	153.2516	153.6515	153.3297
**σC21**	71.6745	71.5307	71.3869	72.8955	72.6163	72.4725	72.3371	72.1933
**σC22**	58.1453	58.1164	58.0874	56.4063	56.9359	56.9069	57.4654	57.4365
**σC23**	122.3790	122.4461	122.5132	123.8794	123.4861	123.5532	123.0928	123.1599
**σC24**	166.0146	165.8230	165.6314	167.6945	167.3442	167.1526	166.9939	166.8023
**σC30**	166.6388	166.9146	167.1903	166.2189	166.2423	166.5181	166.2658	166.5415
**σC29**	171.4751	171.6773	171.8795	173.4405	172.7078	172.9100	171.9751	172.1773
**σC4**	71.4305	71.3817	71.3329	69.5182	70.0091	69.9603	70.5001	70.4513
**σC5**	151.9350	151.7488	151.5627	152.1982	152.2167	152.0305	152.2352	152.0490
**σC2**	113.3916	113.5176	113.6437	113.8275	113.6962	113.8222	113.5648	113.6909
**σC3**	59.5917	59.6237	59.6558	60.6574	60.3774	60.4094	60.0973	60.1294
**σC26**	171.7272	171.8629	171.9985	172.3302	172.1203	172.2560	171.9104	172.0461
**σC1**	171.5790	171.9526	172.3263	172.5171	172.1939	172.5676	171.8707	172.2444
**σC25**	167.1678	167.2907	167.4136	167.6600	167.5257	167.6486	167.3914	167.5143
**σH12a**	29.7066	29.7042	29.7018	29.7126	29.7129	29.7105	29.7133	29.7109
**σH12b**	30.0806	30.0670	30.0534	30.0498	30.0619	30.0484	30.0741	30.0605
**σH11**	27.3494	27.3380	27.3265	27.3403	27.3476	27.3362	27.3549	27.3435
**σH14**	27.2224	27.2121	27.2017	27.1930	27.2083	27.1979	27.2235	27.2132
**σH13a**	29.6356	29.6137	29.5919	29.5748	29.5986	29.5768	29.6224	29.6006
**σH13b**	30.0432	30.0195	29.9959	29.9956	30.0163	29.9927	30.0370	30.0133
**σH8a**	29.8916	29.8767	29.8618	29.8876	29.8924	29.8775	29.8972	29.8823
**σH8b**	29.4925	29.4497	29.4069	29.4104	29.4348	29.3920	29.4592	29.4164
**σH7**	27.8279	27.7826	27.7374	27.7412	27.7726	27.7274	27.8040	27.7588
**σH9a**	29.3764	29.3895	29.4026	29.3575	29.3595	29.3726	29.3615	29.3746
**σH9b**	30.1581	30.1443	30.1306	30.0893	30.1089	30.0952	30.1286	30.1149
**σH16a**	30.0758	30.0763	30.0769	30.0631	30.0695	30.0700	30.0759	30.0764
**σH16b**	29.4093	29.4078	29.4063	29.4522	29.4453	29.4438	29.4385	29.4370
**σH18**	27.6499	27.6567	27.6636	27.6506	27.6518	27.6586	27.6530	27.6599
**σH17a**	29.3672	29.3475	29.3278	29.3091	29.3395	29.3199	29.3700	29.3503
**σH17b**	29.5879	29.5520	29.5161	29.6300	29.6427	29.6068	29.6553	29.6194
**σ$H_3$28**	30.5383	30.5452	30.5521	30.5544	30.5489	30.5558	30.5434	30.5502

**σ$H_3$27**	30.5411	30.5387	30.5362	30.5377	30.5382	30.5357	30.5386	30.5361
**σH20a**	29.6843	29.6734	29.6625	29.8155	29.7598	29.7489	29.7040	29.6931
**σH20b**	29.3681	29.3606	29.3531	29.3829	29.3877	29.3802	29.3925	29.3850
**σH21**	25.0045	25.0626	25.1206	25.2162	25.1353	25.1933	25.0544	25.1124
**σH22**	25.4875	25.4719	25.4563	25.4632	25.4809	25.4653	25.4986	25.4830
**σ$H_3$24**	30.3478	30.3447	30.3416	30.3560	30.3585	30.3554	30.3610	30.3579
**σ$H_3$30**	30.1540	30.1588	30.1636	30.0801	30.1038	30.1087	30.1276	30.1324
**σ$H_3$29**	30.3771	30.3540	30.3309	30.2948	30.3194	30.2963	30.3440	30.3209
**σH4**	24.9651	25.0328	25.1004	25.1259	25.0567	25.1244	24.9875	25.0552
**σH5a**	29.7651	29.7509	29.7368	29.7095	29.7269	29.7128	29.7443	29.7302
**σH5b**	29.1506	29.2023	29.2539	29.3151	29.2559	29.3075	29.1966	29.2483
**σH3**	25.6180	25.6056	25.5931	25.6264	25.6299	25.6174	25.6334	25.6209
**σ$H_3$26**	30.2834	30.2580	30.2327	30.2848	30.2927	30.2673	30.3006	30.2752
**σ$H_3$1**	30.1518	30.1512	30.1507	30.1607	30.1599	30.1593	30.1591	30.1585
**σ$H_3$25**	30.4555	30.4602	30.4649	30.4223	30.4294	30.4341	30.4365	30.4412
$^3J_{H4,H5}$	9.25	8.83	8.41	7.98	8.40	7.98	8.82	8.40
$^3J_{H4',H5}$	4.68	5.12	5.55	6.11	5.65	6.08	5.19	5.62
$^3J_{H20,H21}$	8.02	8.00	7.98	7.52	7.50	7.48	7.48	7.47
$^3J_{H20',H21}$	5.87	5.87	5.87	6.41	6.40	6.40	6.39	6.39

**Conformer 5-1**	**60%**	**60%**	**60%**	**60%**	**60%**	**60%**	**60%**	**60%**
**Conformer 5-2**	**20%**	**10%**	**10%**	**10%**	**10%**	**0%**	**0%**	**0%**
**Conformer 5-5**	**0%**	**30%**	**20%**	**10%**	**0%**	**40%**	**30%**	**20%**
**Conformer 5-9**	**20%**	**0%**	**10%**	**20%**	**30%**	**0%**	**10%**	**20%**
**σC12**	164.2202	164.1248	164.1458	164.1668	164.1878	164.0714	164.0924	164.1133
**σC11**	109.0865	109.7028	109.4909	109.2791	109.0673	109.8954	109.6836	109.4717
**σC14**	110.2963	109.1820	109.5174	109.8528	110.1883	108.7385	109.0739	109.4094
**σC13**	164.2011	164.4718	164.3743	164.2769	164.1794	164.5476	164.4501	164.3527
**σC8**	167.5358	167.1819	167.3150	167.4481	167.5812	167.0942	167.2273	167.3604
**σC7**	110.4520	111.1232	110.9140	110.7048	110.4956	111.3761	111.1668	110.9576
**σC10**	109.9056	110.1474	110.0261	109.9047	109.7833	110.1465	110.0251	109.9038
**σC9**	163.2424	163.6422	163.4732	163.3043	163.1353	163.7040	163.5351	163.3661
**σC16**	164.7009	163.4991	163.8573	164.2154	164.5736	163.0136	163.3718	163.7300
**σC15**	108.8088	108.9400	108.9510	108.9621	108.9732	109.0933	109.1043	109.1154
**σC18**	110.4230	109.9982	110.1804	110.3626	110.5448	109.9378	110.1200	110.3022
**σC17**	168.0628	166.3213	166.8148	167.3084	167.8019	165.5669	166.0604	166.5540
**σC6**	119.5034	120.1700	119.9110	119.6520	119.3931	120.3186	120.0597	119.8007
**σC19**	118.9888	119.4673	119.3348	119.2023	119.0697	119.6808	119.5483	119.4158
**σC28**	172.5310	171.9073	172.0862	172.2652	172.4441	171.6415	171.8204	171.9993
**σC27**	171.6771	172.2101	172.0046	171.7990	171.5935	172.3320	172.1265	171.9209
**σC20**	153.0079	153.7296	153.4078	153.0860	152.7642	153.8077	153.4860	153.1642
**σC21**	72.0495	72.0580	71.9142	71.7703	71.6265	71.7788	71.6350	71.4912
**σC22**	57.4075	57.9950	57.9660	57.9370	57.9081	58.5245	58.4955	58.4666
**σC23**	123.2271	122.6995	122.7666	122.8338	122.9009	122.3062	122.3733	122.4405
**σC24**	166.6108	166.6436	166.4520	166.2604	166.0689	166.2933	166.1017	165.9101
**σC30**	166.8173	166.2893	166.5650	166.8407	167.1164	166.3127	166.5884	166.8642
**σC29**	172.3795	171.2423	171.4446	171.6468	171.8490	170.5096	170.7118	170.9141
**σC4**	70.4025	70.9911	70.9422	70.8934	70.8446	71.4820	71.4332	71.3844
**σC5**	151.8628	152.2537	152.0675	151.8813	151.6952	152.2722	152.0860	151.8998

**σC2**	113.8169	113.4334	113.5595	113.6856	113.8116	113.3021	113.4281	113.5542
**σC3**	60.1614	59.8172	59.8493	59.8814	59.9134	59.5372	59.5692	59.6013
**σC26**	172.1818	171.7005	171.8362	171.9719	172.1076	171.4907	171.6264	171.7620
**σC1**	172.6181	171.5476	171.9212	172.2949	172.6686	171.2244	171.5981	171.9718
**σC25**	167.6371	167.2571	167.3800	167.5028	167.6257	167.1228	167.2456	167.3685
**σH12a**	29.7084	29.7136	29.7112	29.7088	29.7064	29.7140	29.7115	29.7091
**σH12b**	30.0470	30.0863	30.0727	30.0591	30.0456	30.0985	30.0849	30.0713
**σH11**	27.3321	27.3623	27.3509	27.3394	27.3280	27.3696	27.3582	27.3468
**σH14**	27.2029	27.2388	27.2285	27.2182	27.2078	27.2541	27.2438	27.2334
**σH13a**	29.5787	29.6462	29.6244	29.6025	29.5807	29.6701	29.6482	29.6264
**σH13b**	29.9897	30.0577	30.0340	30.0104	29.9868	30.0783	30.0547	30.0311
**σH8a**	29.8675	29.9020	29.8872	29.8723	29.8574	29.9069	29.8920	29.8771
**σH8b**	29.3736	29.4836	29.4408	29.3980	29.3552	29.5080	29.4652	29.4224
**σH7**	27.7135	27.8355	27.7902	27.7450	27.6997	27.8669	27.8216	27.7764
**σH9a**	29.3877	29.3635	29.3766	29.3897	29.4028	29.3655	29.3786	29.3917
**σH9b**	30.1011	30.1483	30.1346	30.1208	30.1070	30.1680	30.1542	30.1405
**σH16a**	30.0770	30.0823	30.0829	30.0834	30.0840	30.0887	30.0893	30.0898
**σH16b**	29.4355	29.4317	29.4302	29.4287	29.4272	29.4248	29.4233	29.4218
**σH18**	27.6667	27.6542	27.6611	27.6679	27.6748	27.6555	27.6623	27.6692
**σH17a**	29.3306	29.4004	29.3807	29.3610	29.3413	29.4308	29.4111	29.3914
**σH17b**	29.5835	29.6680	29.6321	29.5962	29.5603	29.6806	29.6447	29.6088
**σ$H_3$28**	30.5571	30.5378	30.5447	30.5516	30.5584	30.5323	30.5392	30.5460
**σ$H_3$27**	30.5337	30.5390	30.5366	30.5341	30.5317	30.5395	30.5370	30.5346
**σH20a**	29.6822	29.6483	29.6373	29.6264	29.6155	29.5925	29.5816	29.5707
**σH20b**	29.3775	29.3974	29.3899	29.3824	29.3748	29.4022	29.3947	29.3872
**σH21**	25.1705	24.9735	25.0315	25.0896	25.1476	24.8926	24.9506	25.0087
**σH22**	25.4674	25.5164	25.5007	25.4851	25.4695	25.5341	25.5184	25.5028
**σ$H_3$24**	30.3548	30.3635	30.3604	30.3573	30.3542	30.3660	30.3629	30.3598
**σ$H_3$30**	30.1372	30.1513	30.1561	30.1609	30.1657	30.1750	30.1798	30.1846
**σ$H_3$29**	30.2978	30.3686	30.3455	30.3224	30.2993	30.3932	30.3701	30.3470
**σH4**	25.1229	24.9184	24.9860	25.0537	25.1214	24.8492	24.9169	24.9845
**σH5a**	29.7160	29.7618	29.7476	29.7334	29.7193	29.7792	29.7650	29.7509
**σH5b**	29.2999	29.1374	29.1890	29.2406	29.2923	29.0781	29.1298	29.1814
**σH3**	25.6085	25.6369	25.6244	25.6120	25.5995	25.6404	25.6279	25.6155
**σ$H_3$26**	30.2498	30.3085	30.2831	30.2578	30.2324	30.3164	30.2911	30.2657
**σ$H_3$1**	30.1580	30.1583	30.1577	30.1571	30.1566	30.1574	30.1569	30.1563
**σ$H_3$25**	30.4459	30.4436	30.4483	30.4530	30.4577	30.4507	30.4554	30.4601
$^3J_{H4,H5}$	7.98	9.24	8.82	8.40	7.98	9.66	9.24	8.82
$^3J_{H4',H5}$	6.06	4.72	5.16	5.59	6.03	4.26	4.70	5.13
$^3J_{H20,H21}$	7.45	7.47	7.45	7.43	7.41	7.45	7.43	7.41
$^3J_{H20',H21}$	6.39	6.38	6.38	6.38	6.38	6.37	6.37	6.37
**Conformer 5-1**	**60%**	**60%**	**50%**	**50%**	**50%**	**50%**	**50%**	**50%**
**Conformer 5-2**	**0%**	**0%**	**50%**	**40%**	**40%**	**30%**	**30%**	**30%**
**Conformer 5-5**	**10%**	**0%**	**0%**	**10%**	**0%**	**20%**	**10%**	**0%**
**Conformer 5-9**	**30%**	**40%**	**0%**	**0%**	**10%**	**0%**	**10%**	**20%**
**σC12**	164.1343	164.1553	164.3507	164.2973	164.3183	164.2438	164.2648	164.2858
**σC11**	109.2599	109.0481	108.8079	109.0006	108.7887	109.1932	108.9814	108.7696

**σC14**	109.7448	110.0803	110.8039	110.3604	110.6958	109.9169	110.2524	110.5878
**σC13**	164.2552	164.1578	164.1617	164.2375	164.1401	164.3133	164.2159	164.1184
**σC8**	167.4935	167.6267	167.3582	167.2706	167.4037	167.1829	167.3160	167.4491
**σC7**	110.7484	110.5391	110.1257	110.3785	110.1693	110.6313	110.4221	110.2128
**σC10**	109.7824	109.6610	110.0610	110.0601	109.9387	110.0592	109.9378	109.8164
**σC9**	163.1971	163.0281	163.1866	163.2484	163.0794	163.3102	163.1412	162.9723
**σC16**	164.0881	164.4463	165.0744	164.5890	164.9471	164.1035	164.4617	164.8198
**σC15**	109.1265	109.1376	108.5831	108.7364	108.7475	108.8897	108.9008	108.9119
**σC18**	110.4844	110.6667	110.3911	110.3307	110.5130	110.2703	110.4526	110.6348
**σC17**	167.0475	167.5410	168.8581	168.1037	168.5972	167.3493	167.8428	168.3364
**σC6**	119.5418	119.2828	119.5873	119.7359	119.4770	119.8846	119.6256	119.3667
**σC19**	119.2832	119.1507	119.1348	119.3483	119.2158	119.5618	119.4292	119.2967
**σC28**	172.1783	172.3572	172.9451	172.6792	172.8582	172.4134	172.5924	172.7713
**σC27**	171.7154	171.5098	171.6084	171.7303	171.5248	171.8522	171.6467	171.4411
**σC20**	152.8424	152.5206	153.1421	153.2202	152.8984	153.2983	152.9765	152.6548
**σC21**	71.3474	71.2035	73.1351	72.8559	72.7121	72.5768	72.4330	72.2891
**σC22**	58.4376	58.4087	56.2270	56.7565	56.7276	57.2861	57.2571	57.2282
**σC23**	122.5076	122.5748	124.2671	123.8738	123.9409	123.4805	123.5476	123.6148
**σC24**	165.7186	165.5270	168.1320	167.7817	167.5901	167.4314	167.2398	167.0482
**σC30**	167.1399	167.4156	166.1450	166.1685	166.4442	166.1919	166.4677	166.7434
**σC29**	171.1163	171.3185	173.4100	172.6773	172.8795	171.9446	172.1468	172.3490
**σC4**	71.3356	71.2868	69.0299	69.5209	69.4721	70.0118	69.9630	69.9142
**σC5**	151.7137	151.5275	152.3307	152.3492	152.1630	152.3677	152.1815	151.9953
**σC2**	113.6803	113.8063	113.9954	113.8641	113.9901	113.7327	113.8588	113.9848
**σC3**	59.6334	59.6654	60.9151	60.6350	60.6671	60.3549	60.3870	60.4191
**σC26**	171.8977	172.0334	172.4393	172.2294	172.3651	172.0195	172.1552	172.2909
**σC1**	172.3454	172.7191	172.8593	172.5362	172.9099	172.2130	172.5867	172.9604
**σC25**	167.4914	167.6142	167.8721	167.7378	167.8607	167.6035	167.7264	167.8493
**σH12a**	29.7067	29.7043	29.7171	29.7175	29.7151	29.7178	29.7154	29.7130
**σH12b**	30.0578	30.0442	30.0419	30.0541	30.0405	30.0663	30.0527	30.0391
**σH11**	27.3353	27.3239	27.3417	27.3491	27.3376	27.3564	27.3450	27.3336
**σH14**	27.2231	27.2128	27.1991	27.2143	27.2040	27.2296	27.2193	27.2089
**σH13a**	29.6045	29.5826	29.5636	29.5874	29.5656	29.6112	29.5894	29.5675
**σH13b**	30.0074	29.9838	29.9865	30.0072	29.9835	30.0278	30.0042	29.9806
**σH8a**	29.8622	29.8473	29.8832	29.8880	29.8731	29.8928	29.8780	29.8631
**σH8b**	29.3796	29.3368	29.3587	29.3831	29.3403	29.4075	29.3647	29.3219
**σH7**	27.7311	27.6859	27.7035	27.7349	27.6897	27.7664	27.7211	27.6758
**σH9a**	29.4048	29.4178	29.3577	29.3597	29.3728	29.3617	29.3748	29.3879
**σH9b**	30.1267	30.1130	30.0657	30.0854	30.0717	30.1051	30.0913	30.0776
**σH16a**	30.0904	30.0909	30.0702	30.0766	30.0771	30.0830	30.0835	30.0841
**σH16b**	29.4203	29.4188	29.4731	29.4663	29.4648	29.4594	29.4579	29.4564
**σH18**	27.6760	27.6829	27.6617	27.6630	27.6698	27.6642	27.6710	27.6779
**σH17a**	29.3717	29.3520	29.3226	29.3531	29.3334	29.3835	29.3638	29.3441
**σH17b**	29.5730	29.5371	29.6742	29.6868	29.6510	29.6995	29.6636	29.6277
**σ$H_3$28**	30.5529	30.5598	30.5608	30.5552	30.5621	30.5497	30.5566	30.5635
**σ$H_3$27**	30.5321	30.5297	30.5332	30.5336	30.5312	30.5341	30.5316	30.5292
**σH20a**	29.5598	29.5489	29.7686	29.7128	29.7019	29.6571	29.6462	29.6353
**σH20b**	29.3797	29.3722	29.4046	29.4095	29.4019	29.4143	29.4068	29.3993
**σH21**	25.0667	25.1248	25.2432	25.1623	25.2203	25.0813	25.1394	25.1975
**σH22**	25.4872	25.4715	25.4764	25.4941	25.4785	25.5118	25.4962	25.4806
**σ$H_3$24**	30.3567	30.3535	30.3687	30.3711	30.3680	30.3736	30.3705	30.3674

**σ$H_3$30**	30.1895	30.1943	30.0822	30.1059	30.1108	30.1297	30.1345	30.1393
**σ$H_3$29**	30.3239	30.3009	30.2632	30.2878	30.2647	30.3124	30.2893	30.2662
**σH4**	25.0522	25.1199	25.1468	25.0777	25.1453	25.0085	25.0761	25.1438
**σH5a**	29.7367	29.7225	29.6920	29.7094	29.6953	29.7269	29.7127	29.6985
**σH5b**	29.2330	29.2847	29.3535	29.2943	29.3459	29.2350	29.2866	29.3383
**σH3**	25.6030	25.5906	25.6327	25.6362	25.6238	25.6397	25.6273	25.6148
**σ$H_3$26**	30.2403	30.2149	30.2845	30.2924	30.2670	30.3003	30.2749	30.2496
**σ$H_3$1**	30.1558	30.1552	30.1666	30.1658	30.1653	30.1650	30.1644	30.1639
**σ$H_3$25**	30.4647	30.4694	30.4151	30.4222	30.4269	30.4292	30.4339	30.4386
**$^3J_{H4,H5}$**	8.40	7.98	7.55	7.97	7.55	8.39	7.97	7.55
**$^3J_{H4',H5}$**	5.57	6.00	6.59	6.13	6.56	5.66	6.10	6.53
**$^3J_{H20,H21}$**	7.39	7.37	6.95	6.94	6.92	6.92	6.90	6.88
**$^3J_{H20',H21}$**	6.37	6.37	6.91	6.90	6.90	6.89	6.89	6.89

**Conformer 5-1**	**50%**	**50%**	**50%**	**50%**	**50%**	**50%**	**50%**	**50%**
**Conformer 5-2**	**20%**	**20%**	**20%**	**20%**	**10%**	**10%**	**10%**	**10%**
**Conformer 5-5**	**30%**	**20%**	**10%**	**0%**	**40%**	**30%**	**20%**	**10%**
**Conformer 5-9**	**0%**	**10%**	**20%**	**30%**	**0%**	**10%**	**20%**	**30%**
**σC12**	164.1904	164.2114	164.2324	164.2534	164.1370	164.1580	164.1790	164.1999
**σC11**	109.3859	109.1740	108.9622	108.7504	109.5785	109.3667	109.1548	108.9430
**σC14**	109.4735	109.8089	110.1444	110.4798	109.0300	109.3655	109.7009	110.0363
**σC13**	164.3891	164.2917	164.1942	164.0968	164.4649	164.3675	164.2700	164.1726
**σC8**	167.0952	167.2283	167.3615	167.4946	167.0076	167.1407	167.2738	167.4069
**σC7**	110.8841	110.6749	110.4657	110.2564	111.1369	110.9277	110.7185	110.5092
**σC10**	110.0583	109.9369	109.8155	109.6942	110.0574	109.9360	109.8146	109.6933
**σC9**	163.3721	163.2031	163.0341	162.8651	163.4339	163.2649	163.0959	162.9269
**σC16**	163.6180	163.9762	164.3344	164.6925	163.1326	163.4907	163.8489	164.2071
**σC15**	109.0430	109.0541	109.0652	109.0763	109.1963	109.2074	109.2185	109.2296
**σC18**	110.2100	110.3922	110.5744	110.7566	110.1496	110.3318	110.5140	110.6962
**σC17**	166.5949	167.0884	167.5820	168.0755	165.8405	166.3340	166.8276	167.3211
**σC6**	120.0332	119.7743	119.5153	119.2564	120.1819	119.9229	119.6640	119.4050
**σC19**	119.7753	119.6427	119.5102	119.3777	119.9888	119.8562	119.7237	119.5911
**σC28**	172.1476	172.3265	172.5055	172.6844	171.8818	172.0607	172.2397	172.4186
**σC27**	171.9741	171.7686	171.5630	171.3575	172.0960	171.8904	171.6849	171.4794
**σC20**	153.3765	153.0547	152.7329	152.4111	153.4546	153.1328	152.8110	152.4892
**σC21**	72.2976	72.1538	72.0100	71.8662	72.0185	71.8747	71.7308	71.5870
**σC22**	57.8156	57.7867	57.7577	57.7287	58.3452	58.3162	58.2872	58.2583
**σC23**	123.0872	123.1543	123.2215	123.2886	122.6939	122.7610	122.8282	122.8953
**σC24**	167.0811	166.8895	166.6979	166.5063	166.7308	166.5392	166.3476	166.1560
**σC30**	166.2154	166.4911	166.7669	167.0426	166.2389	166.5146	166.7903	167.0660
**σC29**	171.2118	171.4141	171.6163	171.8185	170.4791	170.6813	170.8836	171.0858
**σC4**	70.5028	70.4540	70.4052	70.3564	70.9937	70.9449	70.8961	70.8473
**σC5**	152.3862	152.2000	152.0138	151.8277	152.4047	152.2185	152.0323	151.8462
**σC2**	113.6014	113.7274	113.8535	113.9795	113.4700	113.5960	113.7221	113.8482
**σC3**	60.0749	60.1069	60.1390	60.1711	59.7948	59.8269	59.8589	59.8910
**σC26**	171.8096	171.9453	172.0810	172.2167	171.5997	171.7354	171.8711	172.0068
**σC1**	171.8899	172.2635	172.6372	173.0109	171.5667	171.9404	172.3140	172.6877
**σC25**	167.4692	167.5921	167.7150	167.8378	167.3349	167.4578	167.5806	167.7035
**σH12a**	29.7182	29.7158	29.7133	29.7109	29.7185	29.7161	29.7137	29.7113
**σH12b**	30.0784	30.0649	30.0513	30.0377	30.0906	30.0770	30.0635	30.0499

**σH11**	27.3637	27.3523	27.3409	27.3295	27.3711	27.3597	27.3482	27.3368
**σH14**	27.2449	27.2346	27.2242	27.2139	27.2602	27.2499	27.2395	27.2292
**σH13a**	29.6351	29.6132	29.5913	29.5695	29.6589	29.6370	29.6152	29.5933
**σH13b**	30.0485	30.0249	30.0013	29.9776	30.0692	30.0456	30.0219	29.9983
**σH8a**	29.8977	29.8828	29.8679	29.8530	29.9025	29.8876	29.8727	29.8578
**σH8b**	29.4319	29.3891	29.3463	29.3035	29.4563	29.4135	29.3707	29.3279
**σH7**	27.7978	27.7525	27.7073	27.6620	27.8292	27.7840	27.7387	27.6934
**σH9a**	29.3637	29.3768	29.3899	29.4029	29.3657	29.3788	29.3918	29.4049
**σH9b**	30.1248	30.1110	30.0973	30.0835	30.1445	30.1307	30.1170	30.1032
**σH16a**	30.0894	30.0899	30.0905	30.0910	30.0958	30.0963	30.0969	30.0975
**σH16b**	29.4526	29.4511	29.4496	29.4481	29.4457	29.4442	29.4427	29.4412
**σH18**	27.6654	27.6723	27.6791	27.6860	27.6666	27.6735	27.6803	27.6872
**σH17a**	29.4139	29.3942	29.3745	29.3548	29.4443	29.4246	29.4049	29.3852
**σH17b**	29.7121	29.6762	29.6404	29.6045	29.7248	29.6889	29.6530	29.6171
**σH$_3$28**	30.5442	30.5511	30.5579	30.5648	30.5386	30.5455	30.5524	30.5593
**σH$_3$27**	30.5345	30.5320	30.5296	30.5271	30.5349	30.5325	30.5300	30.5276
**σH20a**	29.6013	29.5904	29.5795	29.5686	29.5456	29.5346	29.5237	29.5128
**σH20b**	29.4191	29.4116	29.4041	29.3966	29.4240	29.4164	29.4089	29.4014
**σH21**	25.0004	25.0585	25.1165	25.1746	24.9195	24.9776	25.0356	25.0937
**σH22**	25.5295	25.5139	25.4983	25.4826	25.5473	25.5316	25.5160	25.5004
**σH$_3$24**	30.3761	30.3730	30.3699	30.3668	30.3786	30.3755	30.3724	30.3693
**σH$_3$30**	30.1534	30.1582	30.1630	30.1678	30.1771	30.1819	30.1867	30.1916
**σH$_3$29**	30.3370	30.3139	30.2909	30.2678	30.3617	30.3386	30.3155	30.2924
**σH4**	24.9393	25.0070	25.0746	25.1423	24.8701	24.9378	25.0055	25.0731
**σH5a**	29.7443	29.7301	29.7159	29.7018	29.7617	29.7475	29.7334	29.7192
**σH5b**	29.1758	29.2274	29.2790	29.3307	29.1165	29.1682	29.2198	29.2714
**σH3**	25.6432	25.6308	25.6183	25.6059	25.6467	25.6343	25.6218	25.6094
**σH$_3$26**	30.3082	30.2829	30.2575	30.2321	30.3161	30.2908	30.2654	30.2400
**σH$_3$1**	30.1642	30.1636	30.1631	30.1625	30.1633	30.1628	30.1622	30.1617
**σH$_3$25**	30.4363	30.4410	30.4457	30.4504	30.4434	30.4481	30.4528	30.4575
$^3J_{H4,H5}$	8.81	8.39	7.97	7.55	9.23	8.81	8.39	7.97
$^3J_{H4',H5}$	5.20	5.63	6.07	6.51	4.74	5.17	5.61	6.04
$^3J_{H20,H21}$	6.90	6.88	6.86	6.84	6.88	6.86	6.84	6.83
$^3J_{H20',H21}$	6.88	6.88	6.88	6.88	6.87	6.87	6.87	6.87

**Conformer 5-1**	**50%**	**50%**	**50%**	**50%**	**50%**	**50%**	**50%**	**40%**
**Conformer 5-2**	**10%**	**0%**	**0%**	**0%**	**0%**	**0%**	**0%**	**60%**
**Conformer 5-5**	**0%**	**50%**	**40%**	**30%**	**20%**	**10%**	**0%**	**0%**
**Conformer 5-9**	**40%**	**0%**	**10%**	**20%**	**30%**	**40%**	**50%**	**0%**
**σC12**	164.2209	164.0836	164.1045	164.1255	164.1465	164.1675	164.1885	164.4163
**σC11**	108.7312	109.7712	109.5593	109.3475	109.1357	108.9238	108.7120	108.4910
**σC14**	110.3718	108.5866	108.9220	109.2574	109.5929	109.9283	110.2637	111.0954
**σC13**	164.0751	164.5408	164.4433	164.3459	164.2484	164.1510	164.0535	164.0791
**σC8**	167.5400	166.9199	167.0530	167.1861	167.3192	167.4523	167.5855	167.2716
**σC7**	110.3000	111.3897	111.1805	110.9713	110.7620	110.5528	110.3436	109.8866
**σC10**	109.5719	110.0565	109.9351	109.8137	109.6924	109.5710	109.4496	109.9718
**σC9**	162.7580	163.4957	163.3267	163.1577	162.9888	162.8198	162.6508	162.9164
**σC16**	164.5652	162.6471	163.0053	163.3634	163.7216	164.0798	164.4380	165.1933

**σC15**	109.2407	109.3496	109.3607	109.3718	109.3829	109.3940	109.4050	108.6862
**σC18**	110.8785	110.0892	110.2714	110.4536	110.6359	110.8181	111.0003	110.6029
**σC17**	167.8146	165.0861	165.5796	166.0731	166.5667	167.0602	167.5538	169.1317
**σC6**	119.1461	120.3306	120.0716	119.8126	119.5537	119.2947	119.0358	119.4505
**σC19**	119.4586	120.2022	120.0697	119.9372	119.8046	119.6721	119.5395	119.4427
**σC28**	172.5975	171.6160	171.7949	171.9738	172.1528	172.3317	172.5106	173.1854
**σC27**	171.2738	172.2179	172.0123	171.8068	171.6012	171.3957	171.1901	171.3724
**σC20**	152.1674	153.5327	153.2110	152.8892	152.5674	152.2456	151.9238	152.7889
**σC21**	71.4432	71.7393	71.5955	71.4517	71.3078	71.1640	71.0202	73.3747
**σC22**	58.2293	58.8747	58.8457	58.8168	58.7878	58.7589	58.7299	56.0477
**σC23**	122.9624	122.3006	122.3677	122.4349	122.5020	122.5691	122.6363	124.6547
**σC24**	165.9644	166.3805	166.1889	165.9973	165.8057	165.6141	165.4226	168.5694
**σC30**	167.3418	166.2623	166.5380	166.8138	167.0895	167.3652	167.6410	166.0712
**σC29**	171.2880	169.7464	169.9486	170.1508	170.3531	170.5553	170.7575	173.3795
**σC4**	70.7985	71.4847	71.4359	71.3871	71.3383	71.2895	71.2406	68.5416
**σC5**	151.6600	152.4232	152.2370	152.0509	151.8647	151.6785	151.4923	152.4632
**σC2**	113.9742	113.3386	113.4647	113.5907	113.7168	113.8429	113.9689	114.1634
**σC3**	59.9231	59.5147	59.5468	59.5789	59.6109	59.6430	59.6751	61.1727
**σC26**	172.1425	171.3899	171.5255	171.6612	171.7969	171.9326	172.0683	172.5483
**σC1**	173.0614	171.2435	171.6172	171.9909	172.3646	172.7382	173.1119	173.2016
**σC25**	167.8264	167.2006	167.3235	167.4463	167.5692	167.6921	167.8149	168.0843
**σH12a**	29.7089	29.7189	29.7164	29.7140	29.7116	29.7092	29.7068	29.7217
**σH12b**	30.0363	30.1028	30.0892	30.0756	30.0621	30.0485	30.0349	30.0340
**σH11**	27.3254	27.3784	27.3670	27.3556	27.3441	27.3327	27.3213	27.3432
**σH14**	27.2188	27.2755	27.2651	27.2548	27.2445	27.2341	27.2238	27.2051
**σH13a**	29.5715	29.6827	29.6608	29.6390	29.6171	29.5953	29.5734	29.5524
**σH13b**	29.9747	30.0899	30.0663	30.0426	30.0190	29.9954	29.9717	29.9773
**σH8a**	29.8429	29.9073	29.8924	29.8775	29.8626	29.8477	29.8328	29.8788
**σH8b**	29.2851	29.4806	29.4378	29.3950	29.3523	29.3095	29.2667	29.3070
**σH7**	27.6482	27.8606	27.8154	27.7701	27.7249	27.6796	27.6344	27.6658
**σH9a**	29.4180	29.3677	29.3807	29.3938	29.4069	29.4200	29.4331	29.3579
**σH9b**	30.0894	30.1642	30.1504	30.1366	30.1229	30.1091	30.0954	30.0422
**σH16a**	30.0980	30.1022	30.1028	30.1033	30.1039	30.1044	30.1050	30.0772
**σH16b**	29.4397	29.4389	29.4374	29.4359	29.4344	29.4329	29.4314	29.4940
**σH18**	27.6940	27.6679	27.6747	27.6816	27.6884	27.6953	27.7021	27.6729
**σH17a**	29.3655	29.4747	29.4550	29.4353	29.4156	29.3959	29.3763	29.3362
**σH17b**	29.5812	29.7374	29.7015	29.6657	29.6298	29.5939	29.5580	29.7184
**σ$H_3$28**	30.5661	30.5331	30.5400	30.5469	30.5537	30.5606	30.5675	30.5671
**σ$H_3$27**	30.5251	30.5354	30.5329	30.5305	30.5280	30.5256	30.5231	30.5286
**σH20a**	29.5019	29.4898	29.4789	29.4680	29.4571	29.4462	29.4352	29.7217
**σH20b**	29.3939	29.4288	29.4213	29.4138	29.4063	29.3987	29.3912	29.4264
**σH21**	25.1518	24.8386	24.8967	24.9547	25.0128	25.0708	25.1289	25.2701
**σH22**	25.4847	25.5650	25.5494	25.5337	25.5181	25.5025	25.4868	25.4896
**σ$H_3$24**	30.3662	30.3811	30.3780	30.3749	30.3718	30.3687	30.3655	30.3813
**σ$H_3$30**	30.1964	30.2008	30.2056	30.2105	30.2153	30.2201	30.2249	30.0843
**σ$H_3$29**	30.2693	30.3863	30.3632	30.3401	30.3170	30.2939	30.2708	30.2316
**σH4**	25.1408	24.8010	24.8686	24.9363	25.0040	25.0716	25.1393	25.1678
**σH5a**	29.7050	29.7791	29.7649	29.7508	29.7366	29.7225	29.7083	29.6745
**σH5b**	29.3231	29.0573	29.1089	29.1605	29.2122	29.2638	29.3155	29.3919
**σH3**	25.5970	25.6502	25.6378	25.6253	25.6129	25.6005	25.5880	25.6391

**σ$H_3$26**	30.2146	30.3241	30.2987	30.2733	30.2479	30.2225	30.1971	30.2842
**σ$H_3$1**	30.1611	30.1625	30.1620	30.1614	30.1608	30.1603	30.1597	30.1726
**σ$H_3$25**	30.4622	30.4505	30.4552	30.4599	30.4646	30.4693	30.4740	30.4078
**$^3J_{H4,H5}$**	7.55	9.64	9.23	8.81	8.39	7.97	7.55	7.12
**$^3J_{H4',H5}$**	6.48	4.27	4.71	5.14	5.58	6.02	6.45	7.06
**$^3J_{H20,H21}$**	6.81	6.86	6.84	6.83	6.81	6.79	6.77	6.39
**$^3J_{H20',H21}$**	6.87	6.86	6.86	6.86	6.86	6.86	6.86	7.41

**Conformer 5-1**	**40%**	**40%**	**40%**	**40%**	**40%**	**40%**	**40%**	**40%**
**Conformer 5-2**	**50%**	**50%**	**40%**	**40%**	**40%**	**30%**	**30%**	**30%**
**Conformer 5-5**	**10%**	**0%**	**20%**	**10%**	**0%**	**30%**	**20%**	**10%**
**Conformer 5-9**	**0%**	**10%**	**0%**	**10%**	**20%**	**0%**	**10%**	**20%**
**σC12**	164.3629	164.3839	164.3095	164.3304	164.3514	164.2560	164.2770	164.2980
**σC11**	108.6837	108.4719	108.8763	108.6645	108.4527	109.0690	108.8571	108.6453
**σC14**	110.6519	110.9874	110.2085	110.5439	110.8793	109.7650	110.1004	110.4359
**σC13**	164.1549	164.0574	164.2307	164.1332	164.0358	164.3065	164.2090	164.1116
**σC8**	167.1839	167.3170	167.0962	167.2294	167.3625	167.0086	167.1417	167.2748
**σC7**	110.1394	109.9301	110.3922	110.1829	109.9737	110.6450	110.4357	110.2265
**σC10**	109.9709	109.8496	109.9700	109.8487	109.7273	109.9691	109.8478	109.7264
**σC9**	162.9782	162.8093	163.0401	162.8711	162.7021	163.1019	162.9329	162.7639
**σC16**	164.7079	165.0661	164.2224	164.5806	164.9388	163.7369	164.0951	164.4533
**σC15**	108.8395	108.8506	108.9928	109.0039	109.0150	109.1461	109.1572	109.1683
**σC18**	110.5425	110.7248	110.4821	110.6644	110.8466	110.4217	110.6040	110.7862
**σC17**	168.3773	168.8708	167.6229	168.1164	168.6100	166.8685	167.3620	167.8556
**σC6**	119.5992	119.3402	119.7479	119.4889	119.2299	119.8965	119.6376	119.3786
**σC19**	119.6562	119.5237	119.8697	119.7372	119.6046	120.0832	119.9507	119.8181
**σC28**	172.9195	173.0985	172.6537	172.8327	173.0116	172.3879	172.5668	172.7458
**σC27**	171.4943	171.2888	171.6162	171.4107	171.2051	171.7381	171.5326	171.3270
**σC20**	152.8671	152.5453	152.9452	152.6234	152.3016	153.0233	152.7015	152.3797
**σC21**	73.0956	72.9518	72.8164	72.6726	72.5288	72.5373	72.3935	72.2496
**σC22**	56.5772	56.5483	57.1067	57.0778	57.0488	57.6363	57.6073	57.5784
**σC23**	124.2615	124.3286	123.8682	123.9353	124.0024	123.4749	123.5420	123.6091
**σC24**	168.2191	168.0275	167.8688	167.6772	167.4857	167.5185	167.3269	167.1353
**σC30**	166.0946	166.3704	166.1181	166.3938	166.6695	166.1415	166.4173	166.6930
**σC29**	172.6468	172.8490	171.9141	172.1163	172.3185	171.1814	171.3836	171.5858
**σC4**	69.0326	68.9838	69.5236	69.4747	69.4259	70.0145	69.9657	69.9169
**σC5**	152.4817	152.2955	152.5002	152.3140	152.1278	152.5187	152.3325	152.1463
**σC2**	114.0320	114.1581	113.9006	114.0267	114.1528	113.7693	113.8953	114.0214
**σC3**	60.8926	60.9247	60.6126	60.6446	60.6767	60.3325	60.3646	60.3966
**σC26**	172.3384	172.4741	172.1286	172.2642	172.3999	171.9187	172.0544	172.1900
**σC1**	172.8785	173.2521	172.5553	172.9290	173.3027	172.2321	172.6058	172.9795
**σC25**	167.9500	168.0728	167.8157	167.9385	168.0614	167.6814	167.8042	167.9271
**σH12a**	29.7220	29.7196	29.7224	29.7200	29.7175	29.7227	29.7203	29.7179
**σH12b**	30.0462	30.0326	30.0584	30.0448	30.0312	30.0706	30.0570	30.0434
**σH11**	27.3505	27.3391	27.3579	27.3465	27.3350	27.3652	27.3538	27.3424
**σH14**	27.2204	27.2101	27.2357	27.2254	27.2150	27.2510	27.2406	27.2303
**σH13a**	29.5762	29.5544	29.6001	29.5782	29.5563	29.6239	29.6020	29.5802
**σH13b**	29.9980	29.9744	30.0187	29.9951	29.9714	30.0394	30.0157	29.9921
**σH8a**	29.8836	29.8688	29.8884	29.8736	29.8587	29.8933	29.8784	29.8635
**σH8b**	29.3314	29.2886	29.3558	29.3130	29.2702	29.3802	29.3374	29.2946
**σH7**	27.6972	27.6520	27.7287	27.6834	27.6382	27.7601	27.7148	27.6696

**σH9a**	29.3599	29.3730	29.3619	29.3750	29.3880	29.3639	29.3769	29.3900
**σH9b**	30.0619	30.0481	30.0816	30.0678	30.0541	30.1013	30.0875	30.0738
**σH16a**	30.0836	30.0842	30.0901	30.0906	30.0912	30.0965	30.0970	30.0976
**σH16b**	29.4872	29.4857	29.4803	29.4788	29.4773	29.4735	29.4720	29.4705
**σH18**	27.6741	27.6810	27.6754	27.6822	27.6891	27.6766	27.6834	27.6903
**σH17a**	29.3666	29.3469	29.3970	29.3773	29.3576	29.4274	29.4077	29.3880
**σH17b**	29.7310	29.6951	29.7437	29.7078	29.6719	29.7563	29.7204	29.6845
**σ$H_3$28**	30.5616	30.5685	30.5561	30.5629	30.5698	30.5505	30.5574	30.5643
**σ$H_3$27**	30.5291	30.5266	30.5295	30.5271	30.5246	30.5300	30.5275	30.5251
**σH20a**	29.6659	29.6550	29.6101	29.5992	29.5883	29.5544	29.5435	29.5326
**σH20b**	29.4312	29.4237	29.4360	29.4285	29.4210	29.4409	29.4334	29.4259
**σH21**	25.1892	25.2473	25.1083	25.1664	25.2244	25.0274	25.0855	25.1435
**σH22**	25.5073	25.4917	25.5250	25.5094	25.4937	25.5427	25.5271	25.5115
**σ$H_3$24**	30.3838	30.3806	30.3863	30.3831	30.3800	30.3888	30.3856	30.3825
**σ$H_3$30**	30.1080	30.1129	30.1318	30.1366	30.1414	30.1555	30.1603	30.1651
**σ$H_3$29**	30.2562	30.2331	30.2809	30.2578	30.2347	30.3055	30.2824	30.2593
**σH4**	25.0986	25.1663	25.0294	25.0971	25.1648	24.9602	25.0279	25.0956
**σH5a**	29.6919	29.6778	29.7094	29.6952	29.6810	29.7268	29.7126	29.6984
**σH5b**	29.3326	29.3843	29.2734	29.3250	29.3767	29.2142	29.2658	29.3174
**σH3**	25.6426	25.6302	25.6461	25.6337	25.6212	25.6496	25.6372	25.6247
**σ$H_3$26**	30.2921	30.2667	30.3000	30.2747	30.2493	30.3079	30.2826	30.2572
**σ$H_3$1**	30.1717	30.1712	30.1709	30.1703	30.1698	30.1701	30.1695	30.1690
**σ$H_3$25**	30.4149	30.4196	30.4220	30.4267	30.4314	30.4291	30.4338	30.4385
$^3J_{H4,H5}$	7.54	7.12	7.96	7.54	7.12	8.38	7.96	7.54
$^3J_{H4',H5}$	6.60	7.04	6.14	6.57	7.01	5.67	6.11	6.55
$^3J_{H20,H21}$	6.37	6.35	6.35	6.33	6.31	6.33	6.31	6.30
$^3J_{H20',H21}$	7.40	7.40	7.39	7.39	7.39	7.38	7.38	7.38

**Conformer 5-1**	**40%**	**40%**	**40%**	**40%**	**40%**	**40%**	**40%**	**40%**
**Conformer 5-2**	**30%**	**20%**	**20%**	**20%**	**20%**	**20%**	**10%**	**10%**
**Conformer 5-5**	**0%**	**40%**	**30%**	**20%**	**10%**	**0%**	**50%**	**40%**
**Conformer 5-9**	**30%**	**0%**	**10%**	**20%**	**30%**	**40%**	**0%**	**10%**
**σC12**	164.3190	164.2026	164.2236	164.2446	164.2655	164.2865	164.1492	164.1702
**σC11**	108.4335	109.2616	109.0498	108.8380	108.6261	108.4143	109.4543	109.2424
**σC14**	110.7713	109.3215	109.6570	109.9924	110.3278	110.6633	108.8781	109.2135
**σC13**	164.0141	164.3823	164.2849	164.1874	164.0900	163.9925	164.4581	164.3607
**σC8**	167.4079	166.9209	167.0540	167.1871	167.3203	167.4534	166.8333	166.9664
**σC7**	110.0173	110.8978	110.6886	110.4793	110.2701	110.0609	111.1506	110.9414
**σC10**	109.6050	109.9682	109.8469	109.7255	109.6041	109.4827	109.9673	109.8459
**σC9**	162.5949	163.1637	162.9947	162.8257	162.6568	162.4878	163.2255	163.0566
**σC16**	164.8115	163.2515	163.6097	163.9678	164.3260	164.6842	162.7660	163.1242
**σC15**	109.1794	109.2994	109.3105	109.3216	109.3327	109.3437	109.4527	109.4638
**σC18**	110.9684	110.3614	110.5436	110.7258	110.9080	111.0903	110.3010	110.4832
**σC17**	168.3491	166.1141	166.6076	167.1011	167.5947	168.0882	165.3597	165.8532
**σC6**	119.1197	120.0452	119.7862	119.5273	119.2683	119.0094	120.1938	119.9349
**σC19**	119.6856	120.2967	120.1641	120.0316	119.8991	119.7665	120.5102	120.3776
**σC28**	172.9247	172.1221	172.3010	172.4800	172.6589	172.8378	171.8563	172.0352
**σC27**	171.1215	171.8600	171.6544	171.4489	171.2433	171.0378	171.9819	171.7763

**σC20**	152.0580	153.1015	152.7797	152.4579	152.1361	151.8143	153.1796	152.8578
**σC21**	72.1058	72.2581	72.1143	71.9705	71.8266	71.6828	71.9790	71.8352
**σC22**	57.5494	58.1658	58.1369	58.1079	58.0789	58.0500	58.6954	58.6664
**σC23**	123.6763	123.0816	123.1487	123.2158	123.2830	123.3501	122.6883	122.7554
**σC24**	166.9438	167.1682	166.9766	166.7850	166.5935	166.4019	166.8179	166.6263
**σC30**	166.9687	166.1650	166.4407	166.7165	166.9922	167.2679	166.1885	166.4642
**σC29**	171.7880	170.4486	170.6509	170.8531	171.0553	171.2575	169.7159	169.9181
**σC4**	69.8681	70.5055	70.4567	70.4078	70.3590	70.3102	70.9964	70.9476
**σC5**	151.9602	152.5372	152.3510	152.1648	151.9787	151.7925	152.5557	152.3695
**σC2**	114.1475	113.6379	113.7640	113.8900	114.0161	114.1422	113.5065	113.6326
**σC3**	60.4287	60.0524	60.0845	60.1166	60.1486	60.1807	59.7724	59.8044
**σC26**	172.3257	171.7088	171.8445	171.9802	172.1158	172.2515	171.4989	171.6346
**σC1**	173.3532	171.9090	172.2827	172.6563	173.0300	173.4037	171.5858	171.9595
**σC25**	168.0499	167.5470	167.6699	167.7928	167.9156	168.0385	167.4127	167.5356
**σH12a**	29.7155	29.7231	29.7206	29.7182	29.7158	29.7134	29.7234	29.7210
**σH12b**	30.0298	30.0828	30.0692	30.0556	30.0420	30.0284	30.0949	30.0814
**σH11**	27.3309	27.3725	27.3611	27.3497	27.3383	27.3269	27.3799	27.3685
**σH14**	27.2200	27.2663	27.2559	27.2456	27.2353	27.2249	27.2816	27.2712
**σH13a**	29.5583	29.6477	29.6258	29.6040	29.5821	29.5603	29.6715	29.6497
**σH13b**	29.9685	30.0601	30.0364	30.0128	29.9892	29.9655	30.0807	30.0571
**σH8a**	29.8486	29.8981	29.8832	29.8683	29.8534	29.8385	29.9029	29.8880
**σH8b**	29.2518	29.4046	29.3618	29.3190	29.2762	29.2334	29.4289	29.3861
**σH7**	27.6243	27.7915	27.7463	27.7010	27.6558	27.6105	27.8230	27.7777
**σH9a**	29.4031	29.3658	29.3789	29.3920	29.4051	29.4182	29.3678	29.3809
**σH9b**	30.0600	30.1210	30.1072	30.0934	30.0797	30.0659	30.1407	30.1269
**σH16a**	30.0981	30.1029	30.1034	30.1040	30.1045	30.1051	30.1093	30.1098
**σH16b**	29.4690	29.4667	29.4652	29.4637	29.4622	29.4607	29.4598	29.4583
**σH18**	27.6971	27.6778	27.6847	27.6915	27.6984	27.7052	27.6791	27.6859
**σH17a**	29.3683	29.4578	29.4381	29.4184	29.3987	29.3790	29.4882	29.4685
**σH17b**	29.6486	29.7689	29.7331	29.6972	29.6613	29.6254	29.7816	29.7457
**σ$H_3$28**	30.5711	30.5450	30.5519	30.5587	30.5656	30.5725	30.5395	30.5463
**σ$H_3$27**	30.5226	30.5304	30.5280	30.5255	30.5231	30.5206	30.5309	30.5284
**σH20a**	29.5217	29.4986	29.4877	29.4768	29.4659	29.4550	29.4429	29.4319
**σH20b**	29.4183	29.4457	29.4382	29.4307	29.4232	29.4157	29.4505	29.4430
**σH21**	25.2016	24.9465	25.0046	25.0626	25.1207	25.1787	24.8656	24.9237
**σH22**	25.4958	25.5605	25.5448	25.5292	25.5136	25.4979	25.5782	25.5625
**σ$H_3$24**	30.3794	30.3913	30.3881	30.3850	30.3819	30.3788	30.3938	30.3906
**σ$H_3$30**	30.1699	30.1792	30.1840	30.1888	30.1937	30.1985	30.2029	30.2077
**σ$H_3$29**	30.2362	30.3301	30.3070	30.2839	30.2608	30.2377	30.3547	30.3316
**σH4**	25.1633	24.8911	24.9587	25.0264	25.0941	25.1618	24.8219	24.8896
**σH5a**	29.6843	29.7442	29.7300	29.7159	29.7017	29.6875	29.7616	29.7475
**σH5b**	29.3691	29.1549	29.2065	29.2582	29.3098	29.3615	29.0957	29.1473
**σH3**	25.6123	25.6531	25.6407	25.6282	25.6158	25.6033	25.6566	25.6442
**σ$H_3$26**	30.2318	30.3159	30.2905	30.2651	30.2397	30.2143	30.3238	30.2984
**σ$H_3$1**	30.1684	30.1693	30.1687	30.1681	30.1676	30.1670	30.1684	30.1679
**σ$H_3$25**	30.4432	30.4362	30.4409	30.4455	30.4502	30.4549	30.4432	30.4479
$^3J_{H4,H5}$	7.12	8.79	8.38	7.96	7.54	7.12	9.21	8.79
$^3J_{H4',H5}$	6.98	5.21	5.65	6.08	6.52	6.95	4.75	5.18
$^3J_{H20,H21}$	6.28	6.31	6.30	6.28	6.26	6.24	6.30	6.28

$^3J_{H20',H21}$	7.38	7.37	7.37	7.37	7.37	7.37	7.37	7.37
**Conformer 5-1**	**40%**	**40%**	**40%**	**40%**	**40%**	**40%**	**40%**	**40%**
**Conformer 5-2**	**10%**	**10%**	**10%**	**10%**	**0%**	**0%**	**0%**	**0%**
**Conformer 5-5**	**30%**	**20%**	**10%**	**0%**	**60%**	**50%**	**40%**	**30%**
**Conformer 5-9**	**20%**	**30%**	**40%**	**50%**	**0%**	**10%**	**20%**	**30%**
**σC12**	164.1911	164.2121	164.2331	164.2541	164.0958	164.1167	164.1377	164.1587
**σC11**	109.0306	108.8188	108.6069	108.3951	109.6469	109.4351	109.2232	109.0114
**σC14**	109.5489	109.8844	110.2198	110.5553	108.4346	108.7701	109.1055	109.4409
**σC13**	164.2632	164.1658	164.0683	163.9709	164.5339	164.4365	164.3390	164.2416
**σC8**	167.0995	167.2326	167.3657	167.4988	166.7456	166.8787	167.0118	167.1449
**σC7**	110.7321	110.5229	110.3137	110.1044	111.4034	111.1942	110.9849	110.7757
**σC10**	109.7246	109.6032	109.4818	109.3605	109.9664	109.8450	109.7237	109.6023
**σC9**	162.8876	162.7186	162.5496	162.3806	163.2874	163.1184	162.9494	162.7804
**σC16**	163.4824	163.8405	164.1987	164.5569	162.2806	162.6387	162.9969	163.3551
**σC15**	109.4749	109.4860	109.4970	109.5081	109.6060	109.6171	109.6282	109.6393
**σC18**	110.6654	110.8477	111.0299	111.2121	110.2406	110.4228	110.6050	110.7873
**σC17**	166.3467	166.8403	167.3338	167.8274	164.6052	165.0988	165.5923	166.0859
**σC6**	119.6759	119.4170	119.1580	118.8991	120.3425	120.0835	119.8246	119.5656
**σC19**	120.2451	120.1125	119.9800	119.8475	120.7237	120.5911	120.4586	120.3260
**σC28**	172.2141	172.3931	172.5720	172.7509	171.5905	171.7694	171.9483	172.1273
**σC27**	171.5708	171.3652	171.1597	170.9541	172.1038	171.8982	171.6927	171.4871
**σC20**	152.5360	152.2142	151.8924	151.5706	153.2577	152.9359	152.6142	152.2924
**σC21**	71.6913	71.5475	71.4037	71.2598	71.6998	71.5560	71.4122	71.2683
**σC22**	58.6374	58.6085	58.5795	58.5506	59.2249	59.1959	59.1670	59.1380
**σC23**	122.8225	122.8897	122.9568	123.0240	122.2950	122.3621	122.4292	122.4964
**σC24**	166.4347	166.2432	166.0516	165.8600	166.4676	166.2760	166.0844	165.8929
**σC30**	166.7399	167.0156	167.2914	167.5671	166.2119	166.4876	166.7634	167.0391
**σC29**	170.1203	170.3226	170.5248	170.7270	168.9832	169.1854	169.3876	169.5898
**σC4**	70.8988	70.8500	70.8012	70.7524	71.4874	71.4386	71.3898	71.3410
**σC5**	152.1834	151.9972	151.8110	151.6248	152.5742	152.3880	152.2019	152.0157
**σC2**	113.7587	113.8847	114.0108	114.1368	113.3752	113.5012	113.6273	113.7534
**σC3**	59.8365	59.8686	59.9006	59.9327	59.4923	59.5244	59.5564	59.5885
**σC26**	171.7703	171.9060	172.0416	172.1773	171.2890	171.4247	171.5604	171.6961
**σC1**	172.3332	172.7068	173.0805	173.4542	171.2627	171.6363	172.0100	172.3837
**σC25**	167.6585	167.7813	167.9042	168.0271	167.2784	167.4013	167.5242	167.6470
**σH12a**	29.7186	29.7162	29.7138	29.7113	29.7238	29.7213	29.7189	29.7165
**σH12b**	30.0678	30.0542	30.0406	30.0270	30.1071	30.0935	30.0800	30.0664
**σH11**	27.3570	27.3456	27.3342	27.3228	27.3872	27.3758	27.3644	27.3529
**σH14**	27.2609	27.2505	27.2402	27.2299	27.2968	27.2865	27.2762	27.2658
**σH13a**	29.6278	29.6059	29.5841	29.5622	29.6953	29.6735	29.6516	29.6298
**σH13b**	30.0335	30.0099	29.9862	29.9626	30.1014	30.0778	30.0542	30.0305
**σH8a**	29.8731	29.8582	29.8433	29.8285	29.9077	29.8928	29.8779	29.8630
**σH8b**	29.3433	29.3006	29.2578	29.2150	29.4533	29.4105	29.3677	29.3249
**σH7**	27.7324	27.6872	27.6419	27.5967	27.8544	27.8091	27.7639	27.7186
**σH9a**	29.3940	29.4071	29.4202	29.4333	29.3698	29.3829	29.3960	29.4091
**σH9b**	30.1131	30.0994	30.0856	30.0718	30.1603	30.1466	30.1328	30.1191
**σH16a**	30.1104	30.1109	30.1115	30.1121	30.1157	30.1162	30.1168	30.1174
**σH16b**	29.4568	29.4553	29.4538	29.4523	29.4530	29.4515	29.4500	29.4485
**σH18**	27.6928	27.6996	27.7064	27.7133	27.6803	27.6871	27.6940	27.7008
**σH17a**	29.4488	29.4291	29.4095	29.3898	29.5186	29.4989	29.4792	29.4596
**σH17b**	29.7098	29.6739	29.6380	29.6022	29.7942	29.7584	29.7225	29.6866

**σ$H_3$28**	30.5532	30.5601	30.5670	30.5738	30.5339	30.5408	30.5477	30.5545
**σ$H_3$27**	30.5259	30.5235	30.5210	30.5186	30.5313	30.5288	30.5264	30.5239
**σH20a**	29.4210	29.4101	29.3992	29.3883	29.3871	29.3762	29.3653	29.3544
**σH20b**	29.4355	29.4280	29.4205	29.4130	29.4554	29.4479	29.4404	29.4328
**σH21**	24.9817	25.0398	25.0978	25.1559	24.7847	24.8428	24.9008	24.9589
**σH22**	25.5469	25.5313	25.5156	25.5000	25.5959	25.5803	25.5646	25.5490
**σ$H_3$24**	30.3875	30.3844	30.3813	30.3781	30.3963	30.3931	30.3900	30.3869
**σ$H_3$30**	30.2126	30.2174	30.2222	30.2270	30.2266	30.2315	30.2363	30.2411
**σ$H_3$29**	30.3085	30.2854	30.2623	30.2392	30.3793	30.3562	30.3331	30.3100
**σH4**	24.9572	25.0249	25.0926	25.1603	24.7527	24.8204	24.8881	24.9557
**σH5a**	29.7333	29.7191	29.7050	29.6908	29.7790	29.7649	29.7507	29.7365
**σH5b**	29.1989	29.2506	29.3022	29.3539	29.0364	29.0881	29.1397	29.1913
**σH3**	25.6317	25.6193	25.6068	25.5944	25.6601	25.6477	25.6352	25.6228
**σ$H_3$26**	30.2730	30.2476	30.2222	30.1968	30.3317	30.3063	30.2809	30.2555
**σ$H_3$1**	30.1673	30.1668	30.1662	30.1656	30.1676	30.1671	30.1665	30.1659
**σ$H_3$25**	30.4526	30.4573	30.4620	30.4667	30.4503	30.4550	30.4597	30.4644
$^{3}J_{H4,H5}$	8.38	7.96	7.54	7.12	9.63	9.21	8.79	8.38
$^{3}J_{H4',H5}$	5.62	6.06	6.49	6.93	4.29	4.72	5.16	5.59
$^{3}J_{H20,H21}$	6.26	6.24	6.22	6.20	6.28	6.26	6.24	6.22
$^{3}J_{H20',H21}$	7.37	7.37	7.37	7.37	7.36	7.36	7.36	7.36

**Conformer 5-1**	**40%**	**40%**	**40%**	**30%**	**30%**	**30%**	**30%**	**30%**
**Conformer 5-2**	**0%**	**0%**	**0%**	**70%**	**60%**	**60%**	**50%**	**50%**
**Conformer 5-5**	**20%**	**10%**	**0%**	**0%**	**10%**	**0%**	**20%**	**10%**
**Conformer 5-9**	**40%**	**50%**	**60%**	**0%**	**0%**	**10%**	**0%**	**10%**
**σC12**	164.1797	164.2007	164.2216	164.4819	164.4285	164.4495	164.3751	164.3960
**σC11**	108.7996	108.5878	108.3759	108.1741	108.3668	108.1550	108.5594	108.3476
**σC14**	109.7764	110.1118	110.4472	111.3869	110.9434	111.2789	110.5000	110.8354
**σC13**	164.1441	164.0467	163.9492	163.9964	164.0722	163.9748	164.1480	164.0506
**σC8**	167.2780	167.4112	167.5443	167.1849	167.0973	167.2304	167.0096	167.1427
**σC7**	110.5665	110.3573	110.1480	109.6474	109.9002	109.6910	110.1530	109.9438
**σC10**	109.4809	109.3596	109.2382	109.8827	109.8818	109.7604	109.8809	109.7595
**σC9**	162.6114	162.4425	162.2735	162.6462	162.7081	162.5391	162.7699	162.6009
**σC16**	163.7132	164.0714	164.4296	165.3123	164.8268	165.1850	164.3413	164.6995
**σC15**	109.6503	109.6614	109.6725	108.7893	108.9426	108.9537	109.0959	109.1070
**σC18**	110.9695	111.1517	111.3340	110.8147	110.7543	110.9366	110.6939	110.8762
**σC17**	166.5794	167.0729	167.5665	169.4053	168.6509	169.1444	167.8965	168.3900
**σC6**	119.3067	119.0477	118.7888	119.3138	119.4625	119.2035	119.6111	119.3522
**σC19**	120.1935	120.0610	119.9284	119.7507	119.9642	119.8316	120.1776	120.0451
**σC28**	172.3062	172.4851	172.6640	173.4257	173.1598	173.3388	172.8940	173.0730
**σC27**	171.2816	171.0760	170.8705	171.1364	171.2583	171.0528	171.3802	171.1747
**σC20**	151.9706	151.6488	151.3270	152.4358	152.5139	152.1921	152.5921	152.2703
**σC21**	71.1245	70.9807	70.8369	73.6144	73.3352	73.1914	73.0561	72.9122
**σC22**	59.1091	59.0801	59.0511	55.8683	56.3979	56.3689	56.9274	56.8985
**σC23**	122.5635	122.6307	122.6978	125.0424	124.6491	124.7163	124.2558	124.3230
**σC24**	165.7013	165.5097	165.3181	169.0069	168.6566	168.4650	168.3063	168.1147
**σC30**	167.3148	167.5906	167.8663	165.9973	166.0208	166.2965	166.0442	166.3200
**σC29**	169.7921	169.9943	170.1965	173.3490	172.6163	172.8185	171.8836	172.0858
**σC4**	71.2921	71.2433	71.1945	68.0534	68.5443	68.4955	69.0353	68.9865

**σC5**	151.8295	151.6433	151.4572	152.5957	152.6142	152.4280	152.6327	152.4465
**σC2**	113.8794	114.0055	114.1315	114.3313	114.1999	114.3260	114.0685	114.1946
**σC3**	59.6206	59.6526	59.6847	61.4303	61.1503	61.1823	60.8702	60.9023
**σC26**	171.8318	171.9675	172.1031	172.6574	172.4475	172.5832	172.2376	172.3733
**σC1**	172.7574	173.1310	173.5047	173.5439	173.2208	173.5944	172.8976	173.2713
**σC25**	167.7699	167.8928	168.0156	168.2964	168.1621	168.2850	168.0278	168.1507
**σH12a**	29.7141	29.7117	29.7093	29.7262	29.7266	29.7242	29.7269	29.7245
**σH12b**	30.0528	30.0392	30.0256	30.0261	30.0383	30.0247	30.0505	30.0369
**σH11**	27.3415	27.3301	27.3187	27.3447	27.3520	27.3406	27.3593	27.3479
**σH14**	27.2555	27.2451	27.2348	27.2112	27.2265	27.2162	27.2418	27.2314
**σH13a**	29.6079	29.5861	29.5642	29.5412	29.5651	29.5432	29.5889	29.5670
**σH13b**	30.0069	29.9833	29.9596	29.9682	29.9889	29.9652	30.0096	29.9859
**σH8a**	29.8481	29.8333	29.8184	29.8744	29.8792	29.8644	29.8841	29.8692
**σH8b**	29.2821	29.2393	29.1966	29.2553	29.2797	29.2369	29.3041	29.2613
**σH7**	27.6734	27.6281	27.5828	27.6281	27.6596	27.6143	27.6910	27.6457
**σH9a**	29.4222	29.4353	29.4484	29.3581	29.3601	29.3731	29.3620	29.3751
**σH9b**	30.1053	30.0915	30.0778	30.0187	30.0384	30.0246	30.0581	30.0443
**σH16a**	30.1179	30.1185	30.1190	30.0843	30.0907	30.0913	30.0971	30.0977
**σH16b**	29.4470	29.4455	29.4440	29.5149	29.5081	29.5066	29.5013	29.4998
**σH18**	27.7077	27.7145	27.7214	27.6841	27.6853	27.6922	27.6866	27.6934
**σH17a**	29.4399	29.4202	29.4005	29.3497	29.3801	29.3604	29.4105	29.3908
**σH17b**	29.6507	29.6148	29.5789	29.7625	29.7752	29.7393	29.7878	29.7519
**$\sigma H_3 28$**	30.5614	30.5683	30.5752	30.5735	30.5679	30.5748	30.5624	30.5693
**$\sigma H_3 27$**	30.5215	30.5190	30.5166	30.5241	30.5245	30.5221	30.5250	30.5225
**σH20a**	29.3435	29.3325	29.3216	29.6747	29.6190	29.6081	29.5632	29.5523
**σH20b**	29.4253	29.4178	29.4103	29.4481	29.4530	29.4454	29.4578	29.4503
**σH21**	25.0169	25.0750	25.1330	25.2971	25.2162	25.2743	25.1353	25.1934
**σH22**	25.5334	25.5177	25.5021	25.5028	25.5205	25.5048	25.5382	25.5226
**$\sigma H_3 24$**	30.3838	30.3806	30.3775	30.3939	30.3964	30.3932	30.3989	30.3957
**$\sigma H_3 30$**	30.2459	30.2507	30.2556	30.0864	30.1101	30.1149	30.1338	30.1387
**$\sigma H_3 29$**	30.2869	30.2638	30.2407	30.2000	30.2247	30.2016	30.2493	30.2262
**σH4**	25.0234	25.0911	25.1587	25.1887	25.1195	25.1872	25.0504	25.1180
**σH5a**	29.7224	29.7082	29.6941	29.6570	29.6744	29.6603	29.6919	29.6777
**σH5b**	29.2430	29.2946	29.3463	29.4303	29.3710	29.4227	29.3118	29.3634
**σH3**	25.6103	25.5979	25.5855	25.6455	25.6490	25.6365	25.6525	25.6400
**$\sigma H_3 26$**	30.2301	30.2047	30.1794	30.2839	30.2918	30.2665	30.2998	30.2744
**$\sigma H_3 1$**	30.1654	30.1648	30.1643	30.1785	30.1776	30.1771	30.1768	30.1763
**$\sigma H_3 25$**	30.4691	30.4738	30.4785	30.4006	30.4077	30.4123	30.4147	30.4194
$^3J_{H4,H5}$	7.96	7.54	7.12	6.69	7.11	6.69	7.53	7.11
$^3J_{H4',H5}$	6.03	6.46	6.90	7.54	7.08	7.51	6.61	7.05
$^3J_{H20,H21}$	6.20	6.19	6.17	5.82	5.80	5.78	5.78	5.76
$^3J_{H20',H21}$	7.36	7.36	7.36	7.91	7.91	7.91	7.90	7.90

**Conformer 5-1**	**30%**	**30%**	**30%**	**30%**	**30%**	**30%**	**30%**	**30%**
**Conformer 5-2**	**50%**	**40%**	**40%**	**40%**	**40%**	**30%**	**30%**	**30%**
**Conformer 5-5**	**0%**	**30%**	**20%**	**10%**	**0%**	**40%**	**30%**	**20%**
**Conformer 5-9**	**20%**	**0%**	**10%**	**20%**	**30%**	**0%**	**10%**	**20%**
**σC12**	164.4170	164.3216	164.3426	164.3636	164.3846	164.2682	164.2892	164.3102

**σC11**	108.1358	108.7521	108.5402	108.3284	108.1166	108.9447	108.7329	108.5211
**σC14**	111.1708	110.0565	110.3919	110.7274	111.0628	109.6131	109.9485	110.2839
**σC13**	163.9531	164.2238	164.1264	164.0289	163.9315	164.2997	164.2022	164.1048
**σC8**	167.2758	166.9219	167.0551	167.1882	167.3213	166.8343	166.9674	167.1005
**σC7**	109.7346	110.4058	110.1966	109.9874	109.7782	110.6587	110.4494	110.2402
**σC10**	109.6381	109.8800	109.7586	109.6372	109.5159	109.8791	109.7577	109.6363
**σC9**	162.4319	162.8317	162.6627	162.4938	162.3248	162.8935	162.7246	162.5556
**σC16**	165.0577	163.8559	164.2140	164.5722	164.9304	163.3704	163.7286	164.0868
**σC15**	109.1180	109.2492	109.2603	109.2713	109.2824	109.4025	109.4136	109.4246
**σC18**	111.0584	110.6335	110.8158	110.9980	111.1802	110.5732	110.7554	110.9376
**σC17**	168.8836	167.1421	167.6356	168.1292	168.6227	166.3877	166.8812	167.3747
**σC6**	119.0932	119.7598	119.5008	119.2419	118.9829	119.9084	119.6495	119.3905
**σC19**	119.9126	120.3911	120.2586	120.1260	119.9935	120.6046	120.4721	120.3395
**σC28**	173.2519	172.6282	172.8071	172.9861	173.1650	172.3624	172.5413	172.7203
**σC27**	170.9691	171.5021	171.2965	171.0910	170.8854	171.6240	171.4184	171.2129
**σC20**	151.9485	152.6702	152.3484	152.0266	151.7048	152.7483	152.4265	152.1047
**σC21**	72.7684	72.7769	72.6331	72.4893	72.3454	72.4978	72.3539	72.2101
**σC22**	56.8695	57.4569	57.4280	57.3990	57.3701	57.9865	57.9575	57.9286
**σC23**	124.3901	123.8625	123.9297	123.9968	124.0640	123.4692	123.5364	123.6035
**σC24**	167.9231	167.9560	167.7644	167.5728	167.3812	167.6057	167.4141	167.2225
**σC30**	166.5957	166.0677	166.3434	166.6191	166.8949	166.0911	166.3669	166.6426
**σC29**	172.2880	171.1509	171.3531	171.5553	171.7575	170.4181	170.6204	170.8226
**σC4**	68.9377	69.5262	69.4774	69.4286	69.3798	70.0172	69.9684	69.9196
**σC5**	152.2603	152.6512	152.4650	152.2788	152.0927	152.6697	152.4835	152.2973
**σC2**	114.3207	113.9372	114.0632	114.1893	114.3154	113.8058	113.9319	114.0579
**σC3**	60.9343	60.5901	60.6222	60.6543	60.6863	60.3101	60.3421	60.3742
**σC26**	172.5090	172.0277	172.1634	172.2991	172.4348	171.8179	171.9535	172.0892
**σC1**	173.6449	172.5744	172.9481	173.3218	173.6955	172.2513	172.6249	172.9986
**σC25**	168.2735	167.8935	168.0163	168.1392	168.2621	167.7592	167.8820	168.0049
**σH12a**	29.7221	29.7273	29.7249	29.7224	29.7200	29.7276	29.7252	29.7228
**σH12b**	30.0233	30.0627	30.0491	30.0355	30.0219	30.0749	30.0613	30.0477
**σH11**	27.3365	27.3667	27.3553	27.3438	27.3324	27.3740	27.3626	27.3512
**σH14**	27.2211	27.2571	27.2467	27.2364	27.2260	27.2723	27.2620	27.2517
**σH13a**	29.5452	29.6127	29.5908	29.5690	29.5471	29.6365	29.6147	29.5928
**σH13b**	29.9623	30.0302	30.0066	29.9830	29.9593	30.0509	30.0273	30.0037
**σH8a**	29.8543	29.8889	29.8740	29.8591	29.8442	29.8937	29.8788	29.8639
**σH8b**	29.2185	29.3285	29.2857	29.2429	29.2001	29.3529	29.3101	29.2673
**σH7**	27.6005	27.7224	27.6772	27.6319	27.5866	27.7538	27.7086	27.6633
**σH9a**	29.3882	29.3640	29.3771	29.3902	29.4033	29.3660	29.3791	29.3922
**σH9b**	30.0305	30.0778	30.0640	30.0502	30.0365	30.0974	30.0837	30.0699
**σH16a**	30.0982	30.1035	30.1041	30.1047	30.1052	30.1100	30.1105	30.1111
**σH16b**	29.4983	29.4944	29.4929	29.4914	29.4899	29.4876	29.4861	29.4846
**σH18**	27.7003	27.6878	27.6946	27.7015	27.7083	27.6890	27.6959	27.7027
**σH17a**	29.3711	29.4409	29.4212	29.4015	29.3818	29.4713	29.4516	29.4319
**σH17b**	29.7160	29.8005	29.7646	29.7287	29.6928	29.8131	29.7772	29.7413
**σ$H_3$28**	30.5762	30.5569	30.5638	30.5706	30.5775	30.5513	30.5582	30.5651
**σ$H_3$27**	30.5201	30.5254	30.5230	30.5205	30.5181	30.5259	30.5234	30.5210
**σH20a**	29.5414	29.5074	29.4965	29.4856	29.4747	29.4517	29.4408	29.4299
**σH20b**	29.4428	29.4626	29.4551	29.4476	29.4401	29.4675	29.4600	29.4524
**σH21**	25.2514	25.0544	25.1125	25.1705	25.2286	24.9735	25.0316	25.0896
**σH22**	25.5069	25.5559	25.5403	25.5247	25.5090	25.5736	25.5580	25.5424

**σ$H_3$24**	30.3926	30.4014	30.3982	30.3951	30.3920	30.4039	30.4007	30.3976
**σ$H_3$30**	30.1435	30.1576	30.1624	30.1672	30.1720	30.1813	30.1861	30.1909
**σ$H_3$29**	30.2031	30.2739	30.2508	30.2277	30.2046	30.2985	30.2754	30.2523
**σH4**	25.1857	24.9812	25.0489	25.1165	25.1842	24.9120	24.9797	25.0474
**σH5a**	29.6635	29.7093	29.6951	29.6810	29.6668	29.7267	29.7125	29.6984
**σH5b**	29.4151	29.2525	29.3042	29.3558	29.4075	29.1933	29.2449	29.2966
**σH3**	25.6276	25.6560	25.6435	25.6311	25.6187	25.6595	25.6470	25.6346
**σ$H_3$26**	30.2490	30.3077	30.2823	30.2569	30.2315	30.3156	30.2902	30.2648
**σ$H_3$1**	30.1757	30.1760	30.1754	30.1749	30.1743	30.1752	30.1746	30.1741
**σ$H_3$25**	30.4241	30.4218	30.4265	30.4312	30.4359	30.4289	30.4336	30.4383
**$^3J_{H4,H5}$**	6.69	7.95	7.53	7.11	6.69	8.36	7.95	7.53
**$^3J_{H4',H5}$**	7.48	6.15	6.59	7.02	7.46	5.69	6.12	6.56
**$^3J_{H20,H21}$**	5.75	5.76	5.75	5.73	5.71	5.75	5.73	5.71
**$^3J_{H20',H21}$**	7.90	7.89	7.89	7.89	7.89	7.88	7.88	7.88

**Conformer 5-1**	**30%**	**30%**	**30%**	**30%**	**30%**	**30%**	**30%**	**30%**
**Conformer 5-2**	**30%**	**30%**	**20%**	**20%**	**20%**	**20%**	**20%**	**20%**
**Conformer 5-5**	**10%**	**0%**	**50%**	**40%**	**30%**	**20%**	**10%**	**0%**
**Conformer 5-9**	**30%**	**40%**	**0%**	**10%**	**20%**	**30%**	**40%**	**50%**
**σC12**	164.3312	164.3521	164.2148	164.2358	164.2567	164.2777	164.2987	164.3197
**σC11**	108.3092	108.0974	109.1374	108.9255	108.7137	108.5019	108.2900	108.0782
**σC14**	110.6194	110.9548	109.1696	109.5050	109.8405	110.1759	110.5113	110.8468
**σC13**	164.0073	163.9099	164.3755	164.2780	164.1806	164.0831	163.9857	163.8882
**σC8**	167.2336	167.3667	166.7466	166.8797	167.0128	167.1460	167.2791	167.4122
**σC7**	110.0310	109.8217	110.9115	110.7022	110.4930	110.2838	110.0745	109.8653
**σC10**	109.5150	109.3936	109.8782	109.7568	109.6354	109.5141	109.3927	109.2713
**σC9**	162.3866	162.2176	162.9554	162.7864	162.6174	162.4484	162.2794	162.1105
**σC16**	164.4449	164.8031	162.8849	163.2431	163.6013	163.9595	164.3176	164.6758
**σC15**	109.4357	109.4468	109.5558	109.5669	109.5779	109.5890	109.6001	109.6112
**σC18**	111.1198	111.3021	110.5128	110.6950	110.8772	111.0595	111.2417	111.4239
**σC17**	167.8683	168.3618	165.6333	166.1268	166.6203	167.1139	167.6074	168.1010
**σC6**	119.1316	118.8726	120.0571	119.7982	119.5392	119.2802	119.0213	118.7623
**σC19**	120.2070	120.0745	120.8181	120.6856	120.5530	120.4205	120.2879	120.1554
**σC28**	172.8992	173.0781	172.0966	172.2755	172.4544	172.6334	172.8123	172.9912
**σC27**	171.0073	170.8018	171.7459	171.5403	171.3348	171.1292	170.9237	170.7181
**σC20**	151.7830	151.4612	152.8265	152.5047	152.1829	151.8611	151.5393	151.2175
**σC21**	72.0663	71.9225	72.2186	72.0748	71.9310	71.7871	71.6433	71.4995
**σC22**	57.8996	57.8706	58.5160	58.4871	58.4581	58.4291	58.4002	58.3712
**σC23**	123.6707	123.7378	123.0759	123.1431	123.2102	123.2774	123.3445	123.4116
**σC24**	167.0309	166.8393	167.2554	167.0638	166.8722	166.6806	166.4890	166.2975
**σC30**	166.9183	167.1941	166.1146	166.3903	166.6661	166.9418	167.2175	167.4932
**σC29**	171.0248	171.2270	169.6854	169.8876	170.0899	170.2921	170.4943	170.6965
**σC4**	69.8708	69.8219	70.5082	70.4593	70.4105	70.3617	70.3129	70.2641
**σC5**	152.1112	151.9250	152.6882	152.5020	152.3159	152.1297	151.9435	151.7573
**σC2**	114.1840	114.3101	113.6745	113.8005	113.9266	114.0526	114.1787	114.3048
**σC3**	60.4063	60.4383	60.0300	60.0621	60.0941	60.1262	60.1583	60.1903
**σC26**	172.2249	172.3606	171.6080	171.7437	171.8793	172.0150	172.1507	172.2864
**σC1**	173.3723	173.7460	171.9281	172.3018	172.6755	173.0491	173.4228	173.7965
**σC25**	168.1278	168.2506	167.6249	167.7477	167.8706	167.9935	168.1163	168.2392
**σH12a**	29.7204	29.7180	29.7280	29.7255	29.7231	29.7207	29.7183	29.7159

**σH12b**	30.0341	30.0205	30.0871	30.0735	30.0599	30.0463	30.0327	30.0191
**σH11**	27.3397	27.3283	27.3814	27.3699	27.3585	27.3471	27.3357	27.3242
**σH14**	27.2413	27.2310	27.2876	27.2773	27.2670	27.2566	27.2463	27.2359
**σH13a**	29.5709	29.5491	29.6603	29.6385	29.6166	29.5948	29.5729	29.5510
**σH13b**	29.9800	29.9564	30.0716	30.0480	30.0243	30.0007	29.9771	29.9535
**σH8a**	29.8490	29.8341	29.8985	29.8836	29.8687	29.8538	29.8389	29.8241
**σH8b**	29.2245	29.1817	29.3772	29.3344	29.2916	29.2489	29.2061	29.1633
**σH7**	27.6181	27.5728	27.7853	27.7400	27.6948	27.6495	27.6042	27.5590
**σH9a**	29.4053	29.4184	29.3680	29.3811	29.3942	29.4073	29.4204	29.4335
**σH9b**	30.0562	30.0424	30.1171	30.1034	30.0896	30.0758	30.0621	30.0483
**σH16a**	30.1116	30.1122	30.1164	30.1169	30.1175	30.1180	30.1186	30.1191
**σH16b**	29.4831	29.4816	29.4807	29.4792	29.4777	29.4762	29.4747	29.4732
**σH18**	27.7096	27.7164	27.6902	27.6971	27.7039	27.7108	27.7176	27.7245
**σH17a**	29.4122	29.3925	29.5017	29.4820	29.4623	29.4427	29.4230	29.4033
**σH17b**	29.7054	29.6696	29.8258	29.7899	29.7540	29.7181	29.6822	29.6463
**σ$H_3$28**	30.5720	30.5788	30.5458	30.5527	30.5596	30.5664	30.5733	30.5802
**σ$H_3$27**	30.5185	30.5161	30.5263	30.5239	30.5214	30.5190	30.5165	30.5141
**σH20a**	29.4190	29.4080	29.3959	29.3850	29.3741	29.3632	29.3523	29.3414
**σH20b**	29.4449	29.4374	29.4723	29.4648	29.4573	29.4498	29.4422	29.4347
**σH21**	25.1477	25.2057	24.8926	24.9507	25.0087	25.0668	25.1248	25.1829
**σH22**	25.5267	25.5111	25.5914	25.5757	25.5601	25.5445	25.5288	25.5132
**σ$H_3$24**	30.3945	30.3914	30.4064	30.4032	30.4001	30.3970	30.3939	30.3908
**σ$H_3$30**	30.1957	30.2006	30.2050	30.2098	30.2146	30.2195	30.2243	30.2291
**σ$H_3$29**	30.2292	30.2061	30.3231	30.3000	30.2769	30.2538	30.2307	30.2076
**σH4**	25.1150	25.1827	24.8428	24.9105	24.9782	25.0458	25.1135	25.1812
**σH5a**	29.6842	29.6700	29.7441	29.7300	29.7158	29.7016	29.6875	29.6733
**σH5b**	29.3482	29.3999	29.1341	29.1857	29.2373	29.2890	29.3406	29.3923
**σH3**	25.6222	25.6097	25.6630	25.6505	25.6381	25.6257	25.6132	25.6008
**σ$H_3$26**	30.2394	30.2140	30.3235	30.2981	30.2727	30.2473	30.2219	30.1965
**σ$H_3$1**	30.1735	30.1729	30.1743	30.1738	30.1732	30.1727	30.1721	30.1716
**σ$H_3$25**	30.4430	30.4477	30.4360	30.4407	30.4454	30.4501	30.4548	30.4595
$^3J_{H4,H5}$	7.11	6.69	8.78	8.36	7.95	7.53	7.11	6.69
$^3J_{H4',H5}$	6.99	7.43	5.22	5.66	6.10	6.53	6.97	7.40
$^3J_{H20,H21}$	5.69	5.67	5.73	5.71	5.69	5.67	5.65	5.64
$^3J_{H20',H21}$	7.88	7.88	7.87	7.87	7.87	7.87	7.87	7.87

**Conformer 5-1**	**30%**	**30%**	**30%**	**30%**	**30%**	**30%**	**30%**	**30%**
**Conformer 5-2**	**10%**	**10%**	**10%**	**10%**	**10%**	**10%**	**10%**	**0%**
**Conformer 5-5**	**60%**	**50%**	**40%**	**30%**	**20%**	**10%**	**0%**	**70%**
**Conformer 5-9**	**0%**	**10%**	**20%**	**30%**	**40%**	**50%**	**60%**	**0%**
**σC12**	164.1614	164.1823	164.2033	164.2243	164.2453	164.2663	164.2872	164.1079
**σC11**	109.3300	109.1182	108.9064	108.6945	108.4827	108.2709	108.0590	109.5227
**σC14**	108.7261	109.0616	109.3970	109.7324	110.0679	110.4033	110.7388	108.2827
**σC13**	164.4513	164.3538	164.2564	164.1589	164.0615	163.9640	163.8666	164.5271
**σC8**	166.6590	166.7921	166.9252	167.0583	167.1914	167.3245	167.4576	166.5713
**σC7**	111.1643	110.9550	110.7458	110.5366	110.3273	110.1181	109.9089	111.4171
**σC10**	109.8773	109.7559	109.6345	109.5132	109.3918	109.2704	109.1490	109.8764
**σC9**	163.0172	162.8482	162.6792	162.5103	162.3413	162.1723	162.0033	163.0790

**σC16**	162.3995	162.7576	163.1158	163.4740	163.8322	164.1903	164.5485	161.9140
**σC15**	109.7091	109.7202	109.7312	109.7423	109.7534	109.7645	109.7756	109.8624
**σC18**	110.4524	110.6346	110.8168	110.9991	111.1813	111.3635	111.5457	110.3920
**σC17**	164.8788	165.3724	165.8659	166.3595	166.8530	167.3465	167.8401	164.1244
**σC6**	120.2058	119.9468	119.6879	119.4289	119.1700	118.9110	118.6520	120.3544
**σC19**	121.0316	120.8990	120.7665	120.6340	120.5014	120.3689	120.2363	121.2451
**σC28**	171.8308	172.0097	172.1886	172.3676	172.5465	172.7254	172.9043	171.5650
**σC27**	171.8678	171.6622	171.4567	171.2511	171.0456	170.8400	170.6345	171.9896
**σC20**	152.9046	152.5828	152.2610	151.9392	151.6174	151.2956	150.9738	152.9827
**σC21**	71.9395	71.7956	71.6518	71.5080	71.3642	71.2203	71.0765	71.6603
**σC22**	59.0456	59.0166	58.9876	58.9587	58.9297	58.9008	58.8718	59.5751
**σC23**	122.6826	122.7498	122.8169	122.8841	122.9512	123.0183	123.0855	122.2894
**σC24**	166.9051	166.7135	166.5219	166.3303	166.1387	165.9472	165.7556	166.5548
**σC30**	166.1381	166.4138	166.6895	166.9652	167.2410	167.5167	167.7924	166.1615
**σC29**	168.9527	169.1549	169.3571	169.5594	169.7616	169.9638	170.1660	168.2200
**σC4**	70.9991	70.9503	70.9015	70.8527	70.8039	70.7551	70.7062	71.4901
**σC5**	152.7067	152.5205	152.3344	152.1482	151.9620	151.7758	151.5897	152.7252
**σC2**	113.5431	113.6691	113.7952	113.9213	114.0473	114.1734	114.2995	113.4117
**σC3**	59.7499	59.7820	59.8141	59.8461	59.8782	59.9103	59.9423	59.4699
**σC26**	171.3981	171.5338	171.6695	171.8051	171.9408	172.0765	172.2122	171.1882
**σC1**	171.6050	171.9786	172.3523	172.7260	173.0996	173.4733	173.8470	171.2818
**σC25**	167.4906	167.6134	167.7363	167.8592	167.9820	168.1049	168.2278	167.3563
**σH12a**	29.7283	29.7259	29.7235	29.7211	29.7186	29.7162	29.7138	29.7287
**σH12b**	30.0993	30.0857	30.0721	30.0585	30.0449	30.0313	30.0177	30.1114
**σH11**	27.3887	27.3773	27.3658	27.3544	27.3430	27.3316	27.3201	27.3960
**σH14**	27.3029	27.2926	27.2822	27.2719	27.2616	27.2512	27.2409	27.3182
**σH13a**	29.6841	29.6623	29.6404	29.6186	29.5967	29.5749	29.5530	29.7080
**σH13b**	30.0923	30.0687	30.0450	30.0214	29.9978	29.9741	29.9505	30.1130
**σH8a**	29.9033	29.8884	29.8735	29.8586	29.8438	29.8289	29.8140	29.9081
**σH8b**	29.4016	29.3588	29.3160	29.2732	29.2304	29.1876	29.1449	29.4260
**σH7**	27.8167	27.7715	27.7262	27.6809	27.6357	27.5904	27.5452	27.8481
**σH9a**	29.3700	29.3831	29.3962	29.4093	29.4224	29.4355	29.4485	29.3720
**σH9b**	30.1368	30.1231	30.1093	30.0955	30.0818	30.0680	30.0543	30.1565
**σH16a**	30.1228	30.1233	30.1239	30.1244	30.1250	30.1255	30.1261	30.1292
**σH16b**	29.4739	29.4724	29.4709	29.4694	29.4679	29.4664	29.4649	29.4671
**σH18**	27.6915	27.6983	27.7052	27.7120	27.7189	27.7257	27.7326	27.6927
**σH17a**	29.5321	29.5124	29.4928	29.4731	29.4534	29.4337	29.4140	29.5625
**σH17b**	29.8384	29.8025	29.7666	29.7307	29.6949	29.6590	29.6231	29.8511
**σ$H_3$28**	30.5403	30.5472	30.5540	30.5609	30.5678	30.5746	30.5815	30.5347
**σ$H_3$27**	30.5268	30.5243	30.5219	30.5194	30.5170	30.5145	30.5120	30.5272
**σH20a**	29.3402	29.3292	29.3183	29.3074	29.2965	29.2856	29.2747	29.2844
**σH20b**	29.4771	29.4696	29.4621	29.4546	29.4471	29.4396	29.4321	29.4820
**σH21**	24.8117	24.8698	24.9278	24.9859	25.0439	25.1020	25.1600	24.7308
**σH22**	25.6091	25.5934	25.5778	25.5622	25.5466	25.5309	25.5153	25.6268
**σ$H_3$24**	30.4089	30.4057	30.4026	30.3995	30.3964	30.3933	30.3901	30.4114
**σ$H_3$30**	30.2287	30.2335	30.2384	30.2432	30.2480	30.2528	30.2576	30.2524
**σ$H_3$29**	30.3477	30.3246	30.3015	30.2784	30.2553	30.2322	30.2091	30.3723
**σH4**	24.7737	24.8413	24.9090	24.9767	25.0443	25.1120	25.1797	24.7045
**σH5a**	29.7615	29.7474	29.7332	29.7190	29.7049	29.6907	29.6766	29.7790
**σH5b**	29.0748	29.1264	29.1781	29.2297	29.2814	29.3330	29.3846	29.0156

**σH3**	25.6665	25.6541	25.6416	25.6292	25.6167	25.6043	25.5918	25.6700
**σ$H_3$26**	30.3314	30.3060	30.2806	30.2552	30.2298	30.2045	30.1791	30.3393
**σ$H_3$1**	30.1735	30.1730	30.1724	30.1719	30.1713	30.1707	30.1702	30.1727
**σ$H_3$25**	30.4431	30.4478	30.4525	30.4572	30.4619	30.4665	30.4712	30.4502
**$^3J_{H4,H5}$**	9.20	8.78	8.36	7.95	7.53	7.11	6.69	9.62
**$^3J_{H4',H5}$**	4.76	5.20	5.63	6.07	6.50	6.94	7.37	4.30
**$^3J_{H20,H21}$**	5.71	5.69	5.67	5.65	5.64	5.62	5.60	5.69
**$^3J_{H20',H21}$**	7.86	7.86	7.86	7.86	7.86	7.86	7.86	7.85

**Conformer 5-1**	**30%**	**30%**	**30%**	**30%**	**30%**	**30%**	**30%**	**20%**
**Conformer 5-2**	**0%**	**0%**	**0%**	**0%**	**0%**	**0%**	**0%**	**80%**
**Conformer 5-5**	**60%**	**50%**	**40%**	**30%**	**20%**	**10%**	**0%**	**0%**
**Conformer 5-9**	**10%**	**20%**	**30%**	**40%**	**50%**	**60%**	**70%**	**0%**
**σC12**	164.1289	164.1499	164.1709	164.1919	164.2128	164.2338	164.2548	164.5475
**σC11**	109.3108	109.0990	108.8872	108.6753	108.4635	108.2517	108.0398	107.8572
**σC14**	108.6181	108.9535	109.2890	109.6244	109.9599	110.2953	110.6307	111.6784
**σC13**	164.4296	164.3322	164.2347	164.1373	164.0398	163.9424	163.8449	163.9138
**σC8**	166.7044	166.8375	166.9706	167.1037	167.2369	167.3700	167.5031	167.0983
**σC7**	111.2078	110.9986	110.7894	110.5802	110.3709	110.1617	109.9525	109.4083
**σC10**	109.7550	109.6336	109.5122	109.3909	109.2695	109.1481	109.0268	109.7936
**σC9**	162.9100	162.7411	162.5721	162.4031	162.2341	162.0651	161.8962	162.3761
**σC16**	162.2722	162.6304	162.9885	163.3467	163.7049	164.0630	164.4212	165.4312
**σC15**	109.8735	109.8845	109.8956	109.9067	109.9178	109.9289	109.9399	108.8924
**σC18**	110.5742	110.7564	110.9387	111.1209	111.3031	111.4854	111.6676	111.0265
**σC17**	164.6180	165.1115	165.6051	166.0986	166.5921	167.0857	167.5792	169.6789
**σC6**	120.0955	119.8365	119.5776	119.3186	119.0597	118.8007	118.5417	119.1771
**σC19**	121.1125	120.9800	120.8474	120.7149	120.5824	120.4498	120.3173	120.0586
**σC28**	171.7439	171.9228	172.1017	172.2807	172.4596	172.6385	172.8175	173.6660
**σC27**	171.7841	171.5785	171.3730	171.1675	170.9619	170.7564	170.5508	170.9004
**σC20**	152.6609	152.3391	152.0174	151.6956	151.3738	151.0520	150.7302	152.0826
**σC21**	71.5165	71.3727	71.2288	71.0850	70.9412	70.7973	70.6535	73.8540
**σC22**	59.5461	59.5172	59.4882	59.4593	59.4303	59.4013	59.3724	55.6890
**σC23**	122.3565	122.4236	122.4908	122.5579	122.6251	122.6922	122.7593	125.4301
**σC24**	166.3632	166.1716	165.9800	165.7884	165.5969	165.4053	165.2137	169.4443
**σC30**	166.4372	166.7130	166.9887	167.2644	167.5402	167.8159	168.0916	165.9235
**σC29**	168.4222	168.6244	168.8266	169.0288	169.2311	169.4333	169.6355	173.3185
**σC4**	71.4413	71.3924	71.3436	71.2948	71.2460	71.1972	71.1484	67.5651
**σC5**	152.5390	152.3529	152.1667	151.9805	151.7943	151.6082	151.4220	152.7282
**σC2**	113.5378	113.6638	113.7899	113.9160	114.0420	114.1681	114.2942	114.4992
**σC3**	59.5019	59.5340	59.5661	59.5981	59.6302	59.6623	59.6943	61.6880
**σC26**	171.3239	171.4596	171.5953	171.7310	171.8666	172.0023	172.1380	172.7664
**σC1**	171.6555	172.0291	172.4028	172.7765	173.1502	173.5238	173.8975	173.8862
**σC25**	167.4791	167.6020	167.7249	167.8477	167.9706	168.0935	168.2163	168.5085
**σH12a**	29.7262	29.7238	29.7214	29.7190	29.7166	29.7142	29.7118	29.7308
**σH12b**	30.0979	30.0843	30.0707	30.0571	30.0435	30.0299	30.0163	30.0182
**σH11**	27.3846	27.3732	27.3618	27.3503	27.3389	27.3275	27.3161	27.3461
**σH14**	27.3079	27.2975	27.2872	27.2768	27.2665	27.2562	27.2458	27.2173
**σH13a**	29.6861	29.6643	29.6424	29.6205	29.5987	29.5768	29.5550	29.5300
**σH13b**	30.0893	30.0657	30.0421	30.0184	29.9948	29.9712	29.9476	29.9591
**σH8a**	29.8932	29.8783	29.8634	29.8486	29.8337	29.8188	29.8039	29.8700
**σH8b**	29.3832	29.3404	29.2976	29.2548	29.2120	29.1692	29.1264	29.2036

**σH7**	27.8029	27.7576	27.7124	27.6671	27.6219	27.5766	27.5313	27.5904
**σH9a**	29.3851	29.3982	29.4113	29.4244	29.4374	29.4505	29.4636	29.3582
**σH9b**	30.1427	30.1290	30.1152	30.1015	30.0877	30.0739	30.0602	29.9952
**σH16a**	30.1297	30.1303	30.1308	30.1314	30.1320	30.1325	30.1331	30.0914
**σH16b**	29.4656	29.4641	29.4626	29.4611	29.4596	29.4581	29.4566	29.5358
**σH18**	27.6995	27.7064	27.7132	27.7201	27.7269	27.7338	27.7406	27.6953
**σH17a**	29.5429	29.5232	29.5035	29.4838	29.4641	29.4444	29.4247	29.3632
**σH17b**	29.8152	29.7793	29.7434	29.7075	29.6716	29.6357	29.5998	29.8067
**σ$H_3$28**	30.5416	30.5485	30.5554	30.5622	30.5691	30.5760	30.5829	30.5798
**σ$H_3$27**	30.5248	30.5223	30.5198	30.5174	30.5149	30.5125	30.5100	30.5196
**σH20a**	29.2735	29.2626	29.2517	29.2408	29.2298	29.2189	29.2080	29.6278
**σH20b**	29.4745	29.4669	29.4594	29.4519	29.4444	29.4369	29.4294	29.4699
**σH21**	24.7889	24.8469	24.9050	24.9630	25.0211	25.0791	25.1372	25.3241
**σH22**	25.6112	25.5955	25.5799	25.5643	25.5486	25.5330	25.5174	25.5159
**σ$H_3$24**	30.4082	30.4051	30.4020	30.3989	30.3958	30.3926	30.3895	30.4065
**σ$H_3$30**	30.2573	30.2621	30.2669	30.2717	30.2765	30.2814	30.2862	30.0885
**σ$H_3$29**	30.3492	30.3261	30.3030	30.2799	30.2569	30.2338	30.2107	30.1685
**σH4**	24.7722	24.8398	24.9075	24.9752	25.0428	25.1105	25.1782	25.2097
**σH5a**	29.7648	29.7506	29.7365	29.7223	29.7081	29.6940	29.6798	29.6395
**σH5b**	29.0672	29.1188	29.1705	29.2221	29.2738	29.3254	29.3770	29.4687
**σH3**	25.6576	25.6451	25.6327	25.6202	25.6078	25.5953	25.5829	25.6519
**σ$H_3$26**	30.3139	30.2885	30.2631	30.2378	30.2124	30.1870	30.1616	30.2836
**σ$H_3$1**	30.1721	30.1716	30.1710	30.1705	30.1699	30.1694	30.1688	30.1844
**σ$H_3$25**	30.4549	30.4596	30.4642	30.4689	30.4736	30.4783	30.4830	30.3933
**$^3J_{H4,H5}$**	9.20	8.78	8.36	7.95	7.53	7.11	6.69	6.26
**$^3J_{H4',H5}$**	4.73	5.17	5.61	6.04	6.48	6.91	7.35	8.02
**$^3J_{H20,H21}$**	5.67	5.65	5.64	5.62	5.60	5.58	5.56	5.25
**$^3J_{H20',H21}$**	7.85	7.85	7.85	7.85	7.85	7.85	7.85	8.42

**Conformer 5-1**	**20%**	**20%**	**20%**	**20%**	**20%**	**20%**	**20%**	**20%**
**Conformer 5-2**	**60%**	**60%**	**40%**	**40%**	**40%**	**20%**	**20%**	**20%**
**Conformer 5-5**	**20%**	**0%**	**40%**	**20%**	**0%**	**60%**	**40%**	**20%**
**Conformer 5-9**	**0%**	**20%**	**0%**	**20%**	**40%**	**0%**	**20%**	**40%**
**σC12**	164.4407	164.4826	164.3338	164.3758	164.4177	164.2270	164.2689	164.3109
**σC11**	108.2425	107.8189	108.6278	108.2042	107.7805	109.0131	108.5895	108.1658
**σC14**	110.7915	111.4624	109.9046	110.5754	111.2463	109.0176	109.6885	110.3594
**σC13**	164.0654	163.8705	164.2170	164.0221	163.8272	164.3686	164.1737	163.9788
**σC8**	166.9230	167.1892	166.7476	167.0139	167.2801	166.5723	166.8385	167.1048
**σC7**	109.9139	109.4954	110.4195	110.0011	109.5826	110.9251	110.5067	110.0882
**σC10**	109.7917	109.5490	109.7899	109.5472	109.3044	109.7881	109.5454	109.3026
**σC9**	162.4997	162.1618	162.6234	162.2854	161.9475	162.7470	162.4091	162.0711
**σC16**	164.4603	165.1766	163.4893	164.2057	164.9220	162.5184	163.2347	163.9511
**σC15**	109.1990	109.2211	109.5056	109.5277	109.5499	109.8122	109.8343	109.8565
**σC18**	110.9057	111.2702	110.7850	111.1494	111.5139	110.6642	111.0286	111.3931
**σC17**	168.1701	169.1572	166.6613	167.6483	168.6354	165.1524	166.1395	167.1266
**σC6**	119.4744	118.9565	119.7717	119.2538	118.7359	120.0690	119.5511	119.0332
**σC19**	120.4856	120.2205	120.9125	120.6475	120.3824	121.3395	121.0744	120.8093
**σC28**	173.1343	173.4922	172.6027	172.9606	173.3184	172.0711	172.4289	172.7868

**σC27**	171.1442	170.7331	171.3880	170.9769	170.5658	171.6317	171.2207	170.8096
**σC20**	152.2389	151.5953	152.3952	151.7516	151.1080	152.5515	151.9079	151.2643
**σC21**	73.2957	73.0081	72.7374	72.4498	72.1621	72.1791	71.8915	71.6038
**σC22**	56.7481	56.6902	57.8071	57.7492	57.6913	58.8662	58.8083	58.7504
**σC23**	124.6435	124.7778	123.8569	123.9912	124.1255	123.0703	123.2046	123.3389
**σC24**	168.7437	168.3606	168.0431	167.6599	167.2768	167.3425	166.9593	166.5762
**σC30**	165.9704	166.5218	166.0173	166.5687	167.1202	166.0642	166.6157	167.1671
**σC29**	171.8531	172.2575	170.3876	170.7921	171.1965	168.9222	169.3266	169.7311
**σC4**	68.5470	68.4494	69.5289	69.4313	69.3337	70.5108	70.4132	70.3156
**σC5**	152.7652	152.3928	152.8022	152.4299	152.0575	152.8392	152.4669	152.0945
**σC2**	114.2365	114.4886	113.9737	114.2259	114.4780	113.7110	113.9631	114.2153
**σC3**	61.1278	61.1920	60.5677	60.6318	60.6960	60.0076	60.0717	60.1358
**σC26**	172.3467	172.6180	171.9269	172.1983	172.4697	171.5072	171.7785	172.0499
**σC1**	173.2399	173.9872	172.5936	173.3409	174.0883	171.9472	172.6946	173.4419
**σC25**	168.2399	168.4857	167.9713	168.2170	168.4628	167.7027	167.9484	168.1942
**σH12a**	29.7315	29.7266	29.7322	29.7273	29.7225	29.7329	29.7280	29.7232
**σH12b**	30.0426	30.0154	30.0670	30.0398	30.0126	30.0914	30.0642	30.0370
**σH11**	27.3608	27.3380	27.3755	27.3526	27.3298	27.3902	27.3673	27.3445
**σH14**	27.2479	27.2272	27.2784	27.2577	27.2371	27.3090	27.2883	27.2676
**σH13a**	29.5777	29.5340	29.6253	29.5816	29.5379	29.6730	29.6292	29.5855
**σH13b**	30.0004	29.9532	30.0418	29.9945	29.9473	30.0832	30.0359	29.9886
**σH8a**	29.8797	29.8499	29.8893	29.8595	29.8297	29.8989	29.8691	29.8394
**σH8b**	29.2524	29.1668	29.3011	29.2156	29.1300	29.3499	29.2643	29.1787
**σH7**	27.6533	27.5628	27.7162	27.6256	27.5351	27.7790	27.6885	27.5980
**σH9a**	29.3622	29.3884	29.3662	29.3924	29.4186	29.3702	29.3964	29.4225
**σH9b**	30.0346	30.0070	30.0739	30.0464	30.0189	30.1133	30.0858	30.0583
**σH16a**	30.1042	30.1053	30.1170	30.1181	30.1193	30.1299	30.1310	30.1321
**σH16b**	29.5222	29.5192	29.5085	29.5055	29.5025	29.4948	29.4918	29.4888
**σH18**	27.6977	27.7114	27.7002	27.7139	27.7276	27.7026	27.7163	27.7300
**σH17a**	29.4240	29.3846	29.4848	29.4454	29.4061	29.5456	29.5063	29.4669
**σH17b**	29.8320	29.7602	29.8573	29.7855	29.7137	29.8826	29.8108	29.7390
**σ$H_3$28**	30.5688	30.5825	30.5577	30.5714	30.5852	30.5466	30.5604	30.5741
**σ$H_3$27**	30.5205	30.5155	30.5213	30.5164	30.5115	30.5222	30.5173	30.5124
**σH20a**	29.5163	29.4945	29.4047	29.3829	29.3611	29.2932	29.2714	29.2496
**σH20b**	29.4795	29.4645	29.4892	29.4742	29.4592	29.4989	29.4839	29.4688
**σH21**	25.1623	25.2784	25.0005	25.1166	25.2327	24.8387	24.9548	25.0709
**σH22**	25.5514	25.5201	25.5868	25.5556	25.5243	25.6223	25.5910	25.5597
**σ$H_3$24**	30.4115	30.4052	30.4165	30.4102	30.4040	30.4215	30.4152	30.4090
**σ$H_3$30**	30.1359	30.1456	30.1834	30.1930	30.2027	30.2308	30.2405	30.2501
**σ$H_3$29**	30.2177	30.1715	30.2669	30.2207	30.1745	30.3161	30.2699	30.2238
**σH4**	25.0713	25.2066	24.9330	25.0683	25.2036	24.7946	24.9299	25.0653
**σH5a**	29.6744	29.6460	29.7092	29.6809	29.6526	29.7440	29.7157	29.6874
**σH5b**	29.3502	29.4535	29.2317	29.3350	29.4383	29.1132	29.2165	29.3198
**σH3**	25.6589	25.6340	25.6659	25.6410	25.6161	25.6729	25.6480	25.6231
**σ$H_3$26**	30.2995	30.2487	30.3153	30.2645	30.2137	30.3311	30.2803	30.2296
**σ$H_3$1**	30.1827	30.1816	30.1811	30.1800	30.1789	30.1794	30.1783	30.1772
**σ$H_3$25**	30.4075	30.4169	30.4217	30.4310	30.4404	30.4358	30.4452	30.4546
**$^3J_{H4,H5}$**	7.10	6.26	7.93	7.10	6.26	8.77	7.93	7.10
**$^3J_{H4',H5}$**	7.09	7.96	6.16	7.03	7.91	5.24	6.11	6.98

$^{3}J_{H20,H21}$	5.22	5.18	5.18	5.14	5.11	5.14	5.11	5.07
$^{3}J_{H20',H21}$	8.40	8.40	8.38	8.38	8.38	8.36	8.36	8.36
**Conformer 5-1**	**20%**	**20%**	**20%**	**20%**	**20%**	**20%**	**10%**	**10%**
**Conformer 5-2**	**20%**	**0%**	**0%**	**0%**	**0%**	**0%**	**90%**	**80%**
**Conformer 5-5**	**0%**	**80%**	**60%**	**40%**	**20%**	**0%**	**0%**	**10%**
**Conformer 5-9**	**60%**	**0%**	**20%**	**40%**	**60%**	**80%**	**0%**	**0%**
**σC12**	164.3529	164.1201	164.1621	164.2040	164.2460	164.2880	164.6131	164.5597
**σC11**	107.7421	109.3984	108.9747	108.5511	108.1274	107.7038	107.5404	107.7330
**σC14**	111.0303	108.1307	108.8016	109.4725	110.1433	110.8142	111.9699	111.5265
**σC13**	163.7839	164.5203	164.3254	164.1305	163.9356	163.7407	163.8311	163.9069
**σC8**	167.3710	166.3970	166.6632	166.9294	167.1957	167.4619	167.0116	166.9240
**σC7**	109.6698	111.4308	111.0123	110.5938	110.1754	109.7569	109.1692	109.4220
**σC10**	109.0599	109.7863	109.5436	109.3008	109.0581	108.8153	109.7044	109.7035
**σC9**	161.7331	162.8707	162.5327	162.1948	161.8568	161.5188	162.1059	162.1677
**σC16**	164.6674	161.5475	162.2638	162.9802	163.6965	164.4128	165.5501	165.0647
**σC15**	109.8786	110.1188	110.1409	110.1631	110.1852	110.2074	108.9954	109.1487
**σC18**	111.7575	110.5434	110.9079	111.2723	111.6368	112.0012	111.2383	111.1779
**σC17**	168.1137	163.6436	164.6307	165.6178	166.6049	167.5919	169.9525	169.1981
**σC6**	118.5153	120.3664	119.8485	119.3305	118.8126	118.2947	119.0404	119.1890
**σC19**	120.5443	121.7665	121.5014	121.2363	120.9712	120.7062	120.3665	120.5800
**σC28**	173.1446	171.5395	171.8973	172.2552	172.6130	172.9709	173.9063	173.6404
**σC27**	170.3985	171.8755	171.4644	171.0533	170.6422	170.2311	170.6644	170.7863
**σC20**	150.6207	152.7077	152.0641	151.4206	150.7770	150.1334	151.7295	151.8076
**σC21**	71.3161	71.6208	71.3332	71.0455	70.7578	70.4702	74.0937	73.8145
**σC22**	58.6925	59.9253	59.8674	59.8095	59.7515	59.6936	55.5097	56.0392
**σC23**	123.4732	122.2837	122.4180	122.5523	122.6866	122.8209	125.8178	125.4245
**σC24**	166.1930	166.6419	166.2587	165.8756	165.4924	165.1093	169.8818	169.5315
**σC30**	167.7186	166.1111	166.6626	167.2140	167.7655	168.3169	165.8496	165.8731
**σC29**	170.1355	167.4568	167.8612	168.2656	168.6701	169.0745	173.2880	172.5553
**σC4**	70.2180	71.4928	71.3951	71.2975	71.1999	71.1023	67.0768	67.5678
**σC5**	151.7222	152.8762	152.5039	152.1315	151.7592	151.3868	152.8607	152.8792
**σC2**	114.4674	113.4483	113.7004	113.9525	114.2046	114.4568	114.6671	114.5357
**σC3**	60.1999	59.4474	59.5116	59.5757	59.6398	59.7039	61.9456	61.6655
**σC26**	172.3213	171.0874	171.3588	171.6301	171.9015	172.1729	172.8755	172.6656
**σC1**	174.1893	171.3009	172.0483	172.7956	173.5430	174.2903	174.2285	173.9053
**σC25**	168.4399	167.4341	167.6798	167.9256	168.1713	168.4170	168.7207	168.5864
**σH12a**	29.7184	29.7336	29.7287	29.7239	29.7191	29.7142	29.7353	29.7357
**σH12b**	30.0098	30.1158	30.0886	30.0614	30.0342	30.0071	30.0103	30.0225
**σH11**	27.3216	27.4048	27.3820	27.3591	27.3363	27.3134	27.3476	27.3549
**σH14**	27.2470	27.3396	27.3189	27.2982	27.2775	27.2569	27.2234	27.2386
**σH13a**	29.5418	29.7206	29.6769	29.6332	29.5895	29.5457	29.5189	29.5427
**σH13b**	29.9414	30.1245	30.0773	30.0300	29.9827	29.9355	29.9499	29.9706
**σH8a**	29.8096	29.9085	29.8787	29.8490	29.8192	29.7894	29.8657	29.8705
**σH8b**	29.0932	29.3987	29.3131	29.2275	29.1419	29.0563	29.1519	29.1763
**σH7**	27.5075	27.8419	27.7514	27.6609	27.5703	27.4798	27.5527	27.5842
**σH9a**	29.4487	29.3742	29.4003	29.4265	29.4527	29.4789	29.3584	29.3604
**σH9b**	30.0307	30.1527	30.1252	30.0976	30.0701	30.0426	29.9717	29.9913
**σH16a**	30.1332	30.1427	30.1438	30.1449	30.1460	30.1471	30.0985	30.1049
**σH16b**	29.4858	29.4811	29.4781	29.4751	29.4721	29.4691	29.5568	29.5499
**σH18**	27.7437	27.7051	27.7188	27.7325	27.7462	27.7599	27.7065	27.7077
**σH17a**	29.4275	29.6065	29.5671	29.5277	29.4883	29.4489	29.3767	29.4071

**σH17b**	29.6672	29.9079	29.8361	29.7643	29.6925	29.6208	29.8508	29.8635
**σ$H_3$28**	30.5879	30.5356	30.5493	30.5631	30.5768	30.5906	30.5862	30.5807
**σ$H_3$27**	30.5075	30.5231	30.5182	30.5133	30.5084	30.5035	30.5150	30.5155
**σH20a**	29.2278	29.1817	29.1599	29.1381	29.1162	29.0944	29.5809	29.5251
**σH20b**	29.4538	29.5086	29.4935	29.4785	29.4635	29.4485	29.4916	29.4965
**σH21**	25.1870	24.6769	24.7930	24.9091	25.0252	25.1413	25.3511	25.2702
**σH22**	25.5285	25.6577	25.6264	25.5952	25.5639	25.5326	25.5291	25.5468
**σ$H_3$24**	30.4027	30.4265	30.4202	30.4140	30.4077	30.4015	30.4191	30.4216
**σ$H_3$30**	30.2597	30.2783	30.2879	30.2975	30.3072	30.3168	30.0906	30.1143
**σ$H_3$29**	30.1776	30.3654	30.3192	30.2730	30.2268	30.1806	30.1369	30.1615
**σH4**	25.2006	24.6562	24.7916	24.9269	25.0623	25.1976	25.2306	25.1614
**σH5a**	29.6591	29.7789	29.7506	29.7222	29.6939	29.6656	29.6220	29.6394
**σH5b**	29.4230	28.9947	29.0980	29.2013	29.3045	29.4078	29.5071	29.4478
**σH3**	25.5982	25.6799	25.6550	25.6301	25.6052	25.5803	25.6582	25.6617
**σ$H_3$26**	30.1788	30.3469	30.2962	30.2454	30.1946	30.1438	30.2834	30.2913
**σ$H_3$1**	30.1761	30.1778	30.1767	30.1756	30.1744	30.1733	30.1903	30.1895
**σ$H_3$25**	30.4640	30.4500	30.4594	30.4688	30.4782	30.4875	30.3861	30.3932
$^3J_{H4,H5}$	6.26	9.61	8.77	7.93	7.10	6.26	5.83	6.25
$^3J_{H4',H5}$	7.85	4.31	5.18	6.05	6.92	7.80	8.49	8.03
$^3J_{H20,H21}$	5.03	5.11	5.07	5.03	5.00	4.96	4.68	4.67
$^3J_{H20',H21}$	8.36	8.34	8.34	8.34	8.34	8.34	8.92	8.91

**Conformer 5-1**	**10%**	**10%**	**10%**	**10%**	**10%**	**10%**	**10%**	**10%**
**Conformer 5-2**	**80%**	**70%**	**70%**	**70%**	**60%**	**60%**	**60%**	**60%**
**Conformer 5-5**	**0%**	**20%**	**10%**	**0%**	**30%**	**20%**	**10%**	**0%**
**Conformer 5-9**	**10%**	**0%**	**10%**	**20%**	**0%**	**10%**	**20%**	**30%**
**σC12**	164.5807	164.5063	164.5273	164.5482	164.4529	164.4738	164.4948	164.5158
**σC11**	107.5212	107.9256	107.7138	107.5020	108.1183	107.9065	107.6946	107.4828
**σC14**	111.8619	111.0830	111.4184	111.7539	110.6395	110.9750	111.3104	111.6459
**σC13**	163.8095	163.9828	163.8853	163.7879	164.0586	163.9611	163.8637	163.7662
**σC8**	167.0571	166.8363	166.9694	167.1025	166.7487	166.8818	167.0149	167.1480
**σC7**	109.2127	109.6748	109.4655	109.2563	109.9276	109.7183	109.5091	109.2999
**σC10**	109.5821	109.7026	109.5812	109.4599	109.7017	109.5803	109.4589	109.3376
**σC9**	161.9988	162.2296	162.0606	161.8916	162.2914	162.1224	161.9534	161.7844
**σC16**	165.4228	164.5792	164.9374	165.2955	164.0937	164.4519	164.8101	165.1682
**σC15**	109.1598	109.3020	109.3131	109.3242	109.4553	109.4664	109.4775	109.4886
**σC18**	111.3602	111.1175	111.2998	111.4820	111.0571	111.2394	111.4216	111.6038
**σC17**	169.6916	168.4437	168.9372	169.4308	167.6893	168.1828	168.6763	169.1699
**σC6**	118.9301	119.3377	119.0787	118.8198	119.4863	119.2274	118.9684	118.7095
**σC19**	120.4475	120.7935	120.6609	120.5284	121.0070	120.8744	120.7419	120.6094
**σC28**	173.8194	173.3746	173.5536	173.7325	173.1088	173.2877	173.4667	173.6456
**σC27**	170.5807	170.9082	170.7026	170.4971	171.0301	170.8245	170.6190	170.4134
**σC20**	151.4858	151.8858	151.5640	151.2422	151.9639	151.6421	151.3203	150.9985
**σC21**	73.6707	73.5354	73.3915	73.2477	73.2562	73.1124	72.9686	72.8247
**σC22**	56.0102	56.5687	56.5398	56.5108	57.0983	57.0693	57.0404	57.0114
**σC23**	125.4916	125.0312	125.0983	125.1655	124.6379	124.7050	124.7722	124.8393
**σC24**	169.3399	169.1812	168.9896	168.7980	168.8309	168.6393	168.4477	168.2561
**σC30**	166.1488	165.8965	166.1722	166.4480	165.9200	166.1957	166.4714	166.7472

**σC29**	172.7575	171.8226	172.0248	172.2270	171.0899	171.2921	171.4943	171.6965
**σC4**	67.5190	68.0587	68.0099	67.9611	68.5497	68.5009	68.4521	68.4033
**σC5**	152.6930	152.8977	152.7115	152.5253	152.9162	152.7300	152.5438	152.3577
**σC2**	114.6618	114.4044	114.5304	114.6565	114.2730	114.3991	114.5251	114.6512
**σC3**	61.6976	61.3855	61.4175	61.4496	61.1054	61.1375	61.1695	61.2016
**σC26**	172.8013	172.4557	172.5914	172.7271	172.2459	172.3815	172.5172	172.6529
**σC1**	174.2790	173.5822	173.9558	174.3295	173.2590	173.6327	174.0064	174.3800
**σC25**	168.7092	168.4521	168.5749	168.6978	168.3177	168.4406	168.5635	168.6863
**σH12a**	29.7333	29.7360	29.7336	29.7312	29.7364	29.7340	29.7315	29.7291
**σH12b**	30.0090	30.0347	30.0211	30.0076	30.0469	30.0333	30.0197	30.0062
**σH11**	27.3435	27.3623	27.3509	27.3394	27.3696	27.3582	27.3468	27.3353
**σH14**	27.2283	27.2539	27.2436	27.2333	27.2692	27.2589	27.2485	27.2382
**σH13a**	29.5208	29.5665	29.5446	29.5228	29.5903	29.5685	29.5466	29.5247
**σH13b**	29.9470	29.9913	29.9677	29.9440	30.0120	29.9883	29.9647	29.9411
**σH8a**	29.8556	29.8753	29.8604	29.8455	29.8801	29.8652	29.8503	29.8354
**σH8b**	29.1335	29.2007	29.1579	29.1151	29.2251	29.1823	29.1395	29.0967
**σH7**	27.5389	27.6156	27.5704	27.5251	27.6470	27.6018	27.5565	27.5113
**σH9a**	29.3735	29.3624	29.3755	29.3886	29.3644	29.3775	29.3906	29.4037
**σH9b**	29.9776	30.0110	29.9973	29.9835	30.0307	30.0170	30.0032	29.9894
**σH16a**	30.1054	30.1113	30.1119	30.1124	30.1177	30.1183	30.1188	30.1194
**σH16b**	29.5484	29.5431	29.5416	29.5401	29.5362	29.5347	29.5332	29.5317
**σH18**	27.7145	27.7089	27.7158	27.7226	27.7101	27.7170	27.7238	27.7307
**σH17a**	29.3874	29.4375	29.4178	29.3981	29.4679	29.4482	29.4285	29.4088
**σH17b**	29.8276	29.8761	29.8403	29.8044	29.8888	29.8529	29.8170	29.7811
**σ$H_3$28**	30.5875	30.5751	30.5820	30.5889	30.5696	30.5765	30.5833	30.5902
**σ$H_3$27**	30.5130	30.5159	30.5135	30.5110	30.5164	30.5139	30.5115	30.5090
**σH20a**	29.5142	29.4693	29.4584	29.4475	29.4136	29.4027	29.3918	29.3809
**σH20b**	29.4890	29.5013	29.4938	29.4863	29.5061	29.4986	29.4911	29.4836
**σH21**	25.3283	25.1893	25.2474	25.3054	25.1084	25.1665	25.2245	25.2826
**σH22**	25.5312	25.5646	25.5489	25.5333	25.5823	25.5667	25.5510	25.5354
**σ$H_3$24**	30.4185	30.4241	30.4210	30.4178	30.4266	30.4235	30.4203	30.4172
**σ$H_3$30**	30.1191	30.1380	30.1429	30.1477	30.1618	30.1666	30.1714	30.1762
**σ$H_3$29**	30.1384	30.1861	30.1630	30.1399	30.2107	30.1876	30.1645	30.1414
**σH4**	25.2291	25.0922	25.1599	25.2276	25.0231	25.0907	25.1584	25.2261
**σH5a**	29.6253	29.6569	29.6427	29.6285	29.6743	29.6601	29.6460	29.6318
**σH5b**	29.4995	29.3886	29.4402	29.4919	29.3293	29.3810	29.4326	29.4843
**σH3**	25.6493	25.6652	25.6528	25.6404	25.6687	25.6563	25.6439	25.6314
**σ$H_3$26**	30.2659	30.2992	30.2738	30.2484	30.3071	30.2817	30.2563	30.2309
**σ$H_3$1**	30.1889	30.1887	30.1881	30.1875	30.1878	30.1873	30.1867	30.1862
**σ$H_3$25**	30.3978	30.4002	30.4049	30.4096	30.4073	30.4120	30.4167	30.4214
$^3J_{H4,H5}$	5.83	6.67	6.25	5.83	7.09	6.67	6.25	5.83
$^3J_{H4',H5}$	8.46	7.57	8.00	8.44	7.10	7.54	7.97	8.41
$^3J_{H20,H21}$	4.65	4.65	4.63	4.61	4.63	4.61	4.59	4.58
$^3J_{H20',H21}$	8.91	8.90	8.90	8.90	8.89	8.89	8.89	8.89
**Conformer 5-1**	**10%**	**10%**	**10%**	**10%**	**10%**	**10%**	**10%**	**10%**
**Conformer 5-2**	**50%**	**50%**	**50%**	**50%**	**50%**	**40%**	**40%**	**40%**
**Conformer 5-5**	**40%**	**30%**	**20%**	**10%**	**0%**	**50%**	**40%**	**30%**

Conformer 5-9	0%	10%	20%	30%	40%	0%	10%	20%
σC12	164.3994	164.4204	164.4414	164.4624	164.4834	164.3460	164.3670	164.3880
σC11	108.3109	108.0991	107.8873	107.6754	107.4636	108.5036	108.2917	108.0799
σC14	110.1961	110.5315	110.8670	111.2024	111.5378	109.7526	110.0881	110.4235
σC13	164.1344	164.0369	163.9395	163.8420	163.7446	164.2102	164.1127	164.0153
σC8	166.6610	166.7941	166.9272	167.0603	167.1934	166.5733	166.7065	166.8396
σC7	110.1804	109.9712	109.7619	109.5527	109.3435	110.4332	110.2240	110.0147
σC10	109.7008	109.5794	109.4580	109.3367	109.2153	109.6999	109.5785	109.4571
σC9	162.3532	162.1842	162.0153	161.8463	161.6773	162.4150	162.2461	162.0771
σC16	163.6083	163.9664	164.3246	164.6828	165.0409	163.1228	163.4810	163.8391
σC15	109.6086	109.6197	109.6308	109.6419	109.6530	109.7619	109.7730	109.7841
σC18	110.9968	111.1790	111.3612	111.5434	111.7257	110.9364	111.1186	111.3008
σC17	166.9349	167.4284	167.9219	168.4155	168.9090	166.1804	166.6740	167.1675
σC6	119.6350	119.3761	119.1171	118.8581	118.5992	119.7837	119.5247	119.2658
σC19	121.2205	121.0879	120.9554	120.8228	120.6903	121.4339	121.3014	121.1689
σC28	172.8430	173.0219	173.2009	173.3798	173.5587	172.5772	172.7561	172.9350
σC27	171.1520	170.9464	170.7409	170.5353	170.3298	171.2738	171.0683	170.8628
σC20	152.0420	151.7203	151.3985	151.0767	150.7549	152.1202	151.7984	151.4766
σC21	72.9771	72.8332	72.6894	72.5456	72.4017	72.6979	72.5541	72.4103
σC22	57.6278	57.5989	57.5699	57.5409	57.5120	58.1573	58.1284	58.0994
σC23	124.2446	124.3117	124.3789	124.4460	124.5132	123.8513	123.9184	123.9856
σC24	168.4806	168.2890	168.0974	167.9058	167.7142	168.1303	167.9387	167.7471
σC30	165.9434	166.2192	166.4949	166.7706	167.0463	165.9669	166.2426	166.5183
σC29	170.3572	170.5594	170.7616	170.9638	171.1660	169.6244	169.8267	170.0289
σC4	69.0407	68.9918	68.9430	68.8942	68.8454	69.5316	69.4828	69.4340
σC5	152.9347	152.7485	152.5624	152.3762	152.1900	152.9532	152.7670	152.5809
σC2	114.1416	114.2677	114.3938	114.5198	114.6459	114.0103	114.1363	114.2624
σC3	60.8253	60.8574	60.8895	60.9215	60.9536	60.5453	60.5773	60.6094
σC26	172.0360	172.1717	172.3073	172.4430	172.5787	171.8261	171.9618	172.0975
σC1	172.9358	173.3095	173.6832	174.0569	174.4305	172.6127	172.9864	173.3600
σC25	168.1834	168.3063	168.4292	168.5520	168.6749	168.0491	168.1720	168.2949
σH12a	29.7367	29.7343	29.7319	29.7295	29.7271	29.7371	29.7346	29.7322
σH12b	30.0591	30.0455	30.0319	30.0183	30.0048	30.0713	30.0577	30.0441
σH11	27.3769	27.3655	27.3541	27.3427	27.3313	27.3843	27.3729	27.3614
σH14	27.2845	27.2742	27.2638	27.2535	27.2431	27.2998	27.2894	27.2791
σH13a	29.6141	29.5923	29.5704	29.5486	29.5267	29.6380	29.6161	29.5942
σH13b	30.0326	30.0090	29.9854	29.9618	29.9381	30.0533	30.0297	30.0061
σH8a	29.8849	29.8700	29.8551	29.8402	29.8253	29.8897	29.8748	29.8599
σH8b	29.2494	29.2067	29.1639	29.1211	29.0783	29.2738	29.2310	29.1882
σH7	27.6785	27.6332	27.5880	27.5427	27.4974	27.7099	27.6646	27.6194
σH9a	29.3664	29.3795	29.3926	29.4057	29.4187	29.3684	29.3815	29.3946
σH9b	30.0504	30.0366	30.0229	30.0091	29.9954	30.0701	30.0563	30.0426
σH16a	30.1241	30.1247	30.1252	30.1258	30.1263	30.1305	30.1311	30.1316
σH16b	29.5294	29.5279	29.5264	29.5249	29.5234	29.5226	29.5211	29.5196
σH18	27.7114	27.7182	27.7251	27.7319	27.7388	27.7126	27.7195	27.7263
σH17a	29.4983	29.4786	29.4589	29.4393	29.4196	29.5287	29.5091	29.4894
σH17b	29.9014	29.8656	29.8297	29.7938	29.7579	29.9141	29.8782	29.8423
σ$H_3$28	30.5641	30.5709	30.5778	30.5847	30.5915	30.5585	30.5654	30.5723
σ$H_3$27	30.5168	30.5144	30.5119	30.5094	30.5070	30.5172	30.5148	30.5123
σH20a	29.3578	29.3469	29.3360	29.3251	29.3142	29.3020	29.2911	29.2802
σH20b	29.5110	29.5035	29.4959	29.4884	29.4809	29.5158	29.5083	29.5008

**σH21**	25.0275	25.0856	25.1436	25.2017	25.2597	24.9466	25.0047	25.0627
**σH22**	25.6000	25.5844	25.5687	25.5531	25.5375	25.6177	25.6021	25.5865
**σ$H_3$24**	30.4291	30.4260	30.4228	30.4197	30.4166	30.4316	30.4285	30.4253
**σ$H_3$30**	30.1855	30.1903	30.1951	30.1999	30.2048	30.2092	30.2140	30.2188
**σ$H_3$29**	30.2353	30.2122	30.1891	30.1660	30.1430	30.2599	30.2369	30.2138
**σH4**	24.9539	25.0216	25.0892	25.1569	25.2246	24.8847	24.9524	25.0201
**σH5a**	29.6917	29.6775	29.6634	29.6492	29.6351	29.7091	29.6950	29.6808
**σH5b**	29.2701	29.3217	29.3734	29.4250	29.4766	29.2108	29.2625	29.3141
**σH3**	25.6723	25.6598	25.6474	25.6349	25.6225	25.6758	25.6633	25.6509
**σ$H_3$26**	30.3150	30.2896	30.2642	30.2388	30.2134	30.3229	30.2975	30.2721
**σ$H_3$1**	30.1870	30.1864	30.1859	30.1853	30.1848	30.1862	30.1856	30.1851
**σ$H_3$25**	30.4144	30.4191	30.4238	30.4285	30.4332	30.4215	30.4262	30.4309
**$^3J_{H4,H5}$**	7.50	7.09	6.67	6.25	5.83	7.92	7.50	7.09
**$^3J_{H4',H5}$**	6.64	7.07	7.51	7.95	8.38	6.18	6.61	7.05
**$^3J_{H20,H21}$**	4.61	4.59	4.58	4.56	4.54	4.59	4.58	4.56
**$^3J_{H20',H21}$**	8.88	8.88	8.88	8.88	8.88	8.88	8.88	8.88

**Conformer 5-1**	**10%**	**10%**	**10%**	**10%**	**10%**	**10%**	**10%**	**10%**
**Conformer 5-2**	**40%**	**40%**	**40%**	**30%**	**30%**	**30%**	**30%**	**30%**
**Conformer 5-5**	**20%**	**10%**	**0%**	**60%**	**50%**	**40%**	**30%**	**20%**
**Conformer 5-9**	**30%**	**40%**	**50%**	**0%**	**10%**	**20%**	**30%**	**40%**
**σC12**	164.4090	164.4299	164.4509	164.2926	164.3136	164.3345	164.3555	164.3765
**σC11**	107.8681	107.6563	107.4444	108.6962	108.4844	108.2726	108.0607	107.8489
**σC14**	110.7589	111.0944	111.4298	109.3092	109.6446	109.9800	110.3155	110.6509
**σC13**	163.9178	163.8204	163.7229	164.2860	164.1885	164.0911	163.9936	163.8962
**σC8**	166.9727	167.1058	167.2389	166.4857	166.6188	166.7519	166.8850	167.0181
**σC7**	109.8055	109.5963	109.3870	110.6860	110.4768	110.2675	110.0583	109.8491
**σC10**	109.3358	109.2144	109.0930	109.6990	109.5776	109.4562	109.3349	109.2135
**σC9**	161.9081	161.7391	161.5701	162.4769	162.3079	162.1389	161.9699	161.8009
**σC16**	164.1973	164.5555	164.9136	162.6373	162.9955	163.3537	163.7118	164.0700
**σC15**	109.7952	109.8063	109.8173	109.9152	109.9263	109.9374	109.9485	109.9596
**σC18**	111.4830	111.6653	111.8475	110.8760	111.0582	111.2404	111.4227	111.6049
**σC17**	167.6611	168.1546	168.6481	165.4260	165.9196	166.4131	166.9067	167.4002
**σC6**	119.0068	118.7479	118.4889	119.9323	119.6734	119.4144	119.1555	118.8965
**σC19**	121.0363	120.9038	120.7712	121.6474	121.5149	121.3823	121.2498	121.1173
**σC28**	173.1140	173.2929	173.4718	172.3114	172.4903	172.6692	172.8482	173.0271
**σC27**	170.6572	170.4517	170.2461	171.3957	171.1902	170.9846	170.7791	170.5735
**σC20**	151.1548	150.8330	150.5112	152.1983	151.8765	151.5547	151.2329	150.9111
**σC21**	72.2664	72.1226	71.9788	72.4188	72.2749	72.1311	71.9873	71.8434
**σC22**	58.0705	58.0415	58.0126	58.6869	58.6579	58.6290	58.6000	58.5710
**σC23**	124.0527	124.1199	124.1870	123.4580	123.5251	123.5923	123.6594	123.7266
**σC24**	167.5555	167.3639	167.1724	167.7800	167.5884	167.3968	167.2052	167.0136
**σC30**	166.7941	167.0698	167.3455	165.9904	166.2661	166.5418	166.8175	167.0933
**σC29**	170.2311	170.4333	170.6355	168.8917	169.0939	169.2961	169.4984	169.7006
**σC4**	69.3852	69.3364	69.2875	70.0226	69.9738	69.9249	69.8761	69.8273
**σC5**	152.3947	152.2085	152.0223	152.9717	152.7855	152.5994	152.4132	152.2270
**σC2**	114.3885	114.5145	114.6406	113.8789	114.0050	114.1310	114.2571	114.3832
**σC3**	60.6415	60.6735	60.7056	60.2652	60.2973	60.3293	60.3614	60.3935
**σC26**	172.2331	172.3688	172.5045	171.6162	171.7519	171.8876	172.0233	172.1590

**σC1**	173.7337	174.1074	174.4811	172.2895	172.6632	173.0369	173.4105	173.7842
**σC25**	168.4177	168.5406	168.6635	167.9148	168.0377	168.1606	168.2834	168.4063
**σH12a**	29.7298	29.7274	29.7250	29.7374	29.7350	29.7326	29.7302	29.7278
**σH12b**	30.0305	30.0170	30.0034	30.0835	30.0699	30.0563	30.0427	30.0291
**σH11**	27.3500	27.3386	27.3272	27.3916	27.3802	27.3688	27.3574	27.3459
**σH14**	27.2688	27.2584	27.2481	27.3151	27.3047	27.2944	27.2841	27.2737
**σH13a**	29.5724	29.5505	29.5287	29.6618	29.6399	29.6181	29.5962	29.5743
**σH13b**	29.9824	29.9588	29.9352	30.0740	30.0504	30.0268	30.0031	29.9795
**σH8a**	29.8450	29.8302	29.8153	29.8945	29.8796	29.8647	29.8499	29.8350
**σH8b**	29.1455	29.1027	29.0599	29.2982	29.2554	29.2126	29.1698	29.1270
**σH7**	27.5741	27.5289	27.4836	27.7413	27.6961	27.6508	27.6056	27.5603
**σH9a**	29.4076	29.4207	29.4338	29.3704	29.3835	29.3965	29.4096	29.4227
**σH9b**	30.0288	30.0151	30.0013	30.0898	30.0760	30.0623	30.0485	30.0347
**σH16a**	30.1322	30.1327	30.1333	30.1369	30.1375	30.1380	30.1386	30.1392
**σH16b**	29.5181	29.5166	29.5151	29.5157	29.5142	29.5127	29.5112	29.5097
**σH18**	27.7331	27.7400	27.7468	27.7138	27.7207	27.7275	27.7344	27.7412
**σH17a**	29.4697	29.4500	29.4303	29.5592	29.5395	29.5198	29.5001	29.4804
**σH17b**	29.8064	29.7705	29.7346	29.9267	29.8908	29.8550	29.8191	29.7832
**σ$H_3$28**	30.5791	30.5860	30.5929	30.5530	30.5599	30.5667	30.5736	30.5805
**σ$H_3$27**	30.5099	30.5074	30.5050	30.5177	30.5152	30.5128	30.5103	30.5079
**σH20a**	29.2693	29.2584	29.2475	29.2463	29.2354	29.2245	29.2136	29.2026
**σH20b**	29.4933	29.4858	29.4782	29.5206	29.5131	29.5056	29.4981	29.4906
**σH21**	25.1208	25.1788	25.2369	24.8657	24.9237	24.9818	25.0399	25.0979
**σH22**	25.5708	25.5552	25.5396	25.6355	25.6198	25.6042	25.5886	25.5729
**σ$H_3$24**	30.4222	30.4191	30.4160	30.4341	30.4310	30.4278	30.4247	30.4216
**σ$H_3$30**	30.2237	30.2285	30.2333	30.2329	30.2377	30.2426	30.2474	30.2522
**σ$H_3$29**	30.1907	30.1676	30.1445	30.2846	30.2615	30.2384	30.2153	30.1922
**σH4**	25.0877	25.1554	25.2231	24.8155	24.8832	24.9509	25.0186	25.0862
**σH5a**	29.6666	29.6525	29.6383	29.7265	29.7124	29.6982	29.6841	29.6699
**σH5b**	29.3658	29.4174	29.4690	29.1516	29.2032	29.2549	29.3065	29.3582
**σH3**	25.6384	25.6260	25.6135	25.6793	25.6668	25.6544	25.6419	25.6295
**σ$H_3$26**	30.2467	30.2214	30.1960	30.3308	30.3054	30.2800	30.2547	30.2293
**σ$H_3$1**	30.1845	30.1839	30.1834	30.1854	30.1848	30.1842	30.1837	30.1831
**σ$H_3$25**	30.4356	30.4403	30.4450	30.4286	30.4333	30.4380	30.4427	30.4474
$^3J_{H4,H5}$	6.67	6.25	5.83	8.34	7.92	7.50	7.09	6.67
$^3J_{H4',H5}$	7.48	7.92	8.35	5.71	6.15	6.58	7.02	7.46
$^3J_{H20,H21}$	4.54	4.52	4.50	4.58	4.56	4.54	4.52	4.50
$^3J_{H20',H21}$	8.88	8.88	8.88	8.87	8.87	8.87	8.87	8.87

**Conformer 5-1**	**10%**	**10%**	**10%**	**10%**	**10%**	**10%**	**10%**	**10%**
**Conformer 5-2**	**30%**	**30%**	**20%**	**20%**	**20%**	**20%**	**20%**	**20%**
**Conformer 5-5**	**10%**	**0%**	**70%**	**60%**	**50%**	**40%**	**30%**	**20%**
**Conformer 5-9**	**50%**	**60%**	**0%**	**10%**	**20%**	**30%**	**40%**	**50%**
**σC12**	164.3975	164.4185	164.2392	164.2601	164.2811	164.3021	164.3231	164.3441
**σC11**	107.6371	107.4252	108.8889	108.6770	108.4652	108.2534	108.0415	107.8297
**σC14**	110.9863	111.3218	108.8657	109.2011	109.5366	109.8720	110.2074	110.5429
**σC13**	163.7987	163.7013	164.3618	164.2644	164.1669	164.0695	163.9720	163.8746
**σC8**	167.1512	167.2843	166.3980	166.5311	166.6642	166.7974	166.9305	167.0636

**σC7**	109.6399	109.4306	110.9388	110.7296	110.5204	110.3111	110.1019	109.8927
**σC10**	109.0921	108.9707	109.6981	109.5767	109.4553	109.3340	109.2126	109.0912
**σC9**	161.6320	161.4630	162.5387	162.3697	162.2007	162.0318	161.8628	161.6938
**σC16**	164.4282	164.7864	162.1519	162.5100	162.8682	163.2264	163.5845	163.9427
**σC15**	109.9706	109.9817	110.0685	110.0796	110.0907	110.1018	110.1129	110.1239
**σC18**	111.7871	111.9693	110.8156	110.9978	111.1800	111.3623	111.5445	111.7267
**σC17**	167.8937	168.3873	164.6716	165.1652	165.6587	166.1523	166.6458	167.1393
**σC6**	118.6376	118.3786	120.0810	119.8220	119.5631	119.3041	119.0452	118.7862
**σC19**	120.9847	120.8522	121.8609	121.7284	121.5958	121.4633	121.3308	121.1982
**σC28**	173.2060	173.3849	172.0456	172.2245	172.4034	172.5823	172.7613	172.9402
**σC27**	170.3680	170.1624	171.5176	171.3121	171.1065	170.9010	170.6954	170.4899
**σC20**	150.5894	150.2676	152.2765	151.9547	151.6329	151.3111	150.9893	150.6675
**σC21**	71.6996	71.5558	72.1396	71.9958	71.8519	71.7081	71.5643	71.4205
**σC22**	58.5421	58.5131	59.2164	59.1875	59.1585	59.1295	59.1006	59.0716
**σC23**	123.7937	123.8608	123.0647	123.1319	123.1990	123.2661	123.3333	123.4004
**σC24**	166.8221	166.6305	167.4296	167.2381	167.0465	166.8549	166.6633	166.4718
**σC30**	167.3690	167.6447	166.0138	166.2895	166.5653	166.8410	167.1167	167.3924
**σC29**	169.9028	170.1050	168.1590	168.3612	168.5634	168.7656	168.9679	169.1701
**σC4**	69.7785	69.7297	70.5135	70.4647	70.4159	70.3671	70.3183	70.2695
**σC5**	152.0408	151.8547	152.9902	152.8041	152.6179	152.4317	152.2455	152.0594
**σC2**	114.5092	114.6353	113.7476	113.8736	113.9997	114.1257	114.2518	114.3779
**σC3**	60.4255	60.4576	59.9851	60.0172	60.0493	60.0813	60.1134	60.1455
**σC26**	172.2946	172.4303	171.4063	171.5420	171.6777	171.8134	171.9491	172.0848
**σC1**	174.1579	174.5316	171.9664	172.3400	172.7137	173.0874	173.4611	173.8347
**σC25**	168.5292	168.6520	167.7805	167.9034	168.0263	168.1491	168.2720	168.3949
**σH12a**	29.7253	29.7229	29.7378	29.7353	29.7329	29.7305	29.7281	29.7257
**σH12b**	30.0156	30.0020	30.0957	30.0821	30.0685	30.0549	30.0413	30.0277
**σH11**	27.3345	27.3231	27.3990	27.3875	27.3761	27.3647	27.3533	27.3418
**σH14**	27.2634	27.2530	27.3304	27.3200	27.3097	27.2993	27.2890	27.2787
**σH13a**	29.5525	29.5306	29.6856	29.6637	29.6419	29.6200	29.5982	29.5763
**σH13b**	29.9559	29.9322	30.0947	30.0711	30.0474	30.0238	30.0002	29.9765
**σH8a**	29.8201	29.8052	29.8993	29.8844	29.8695	29.8547	29.8398	29.8249
**σH8b**	29.0842	29.0415	29.3226	29.2798	29.2370	29.1942	29.1514	29.1086
**σH7**	27.5150	27.4698	27.7728	27.7275	27.6823	27.6370	27.5917	27.5465
**σH9a**	29.4358	29.4489	29.3724	29.3854	29.3985	29.4116	29.4247	29.4378
**σH9b**	30.0210	30.0072	30.1095	30.0957	30.0819	30.0682	30.0544	30.0407
**σH16a**	30.1397	30.1403	30.1433	30.1439	30.1445	30.1450	30.1456	30.1461
**σH16b**	29.5082	29.5067	29.5089	29.5074	29.5059	29.5044	29.5029	29.5014
**σH18**	27.7481	27.7549	27.7151	27.7219	27.7288	27.7356	27.7424	27.7493
**σH17a**	29.4607	29.4410	29.5896	29.5699	29.5502	29.5305	29.5108	29.4911
**σH17b**	29.7473	29.7114	29.9394	29.9035	29.8676	29.8317	29.7958	29.7599
**σ$H_3$28**	30.5873	30.5942	30.5475	30.5543	30.5612	30.5681	30.5749	30.5818
**σ$H_3$27**	30.5054	30.5030	30.5181	30.5157	30.5132	30.5108	30.5083	30.5059
**σH20a**	29.1917	29.1808	29.1905	29.1796	29.1687	29.1578	29.1469	29.1360
**σH20b**	29.4831	29.4756	29.5255	29.5180	29.5104	29.5029	29.4954	29.4879
**σH21**	25.1560	25.2140	24.7848	24.8428	24.9009	24.9590	25.0170	25.0751
**σH22**	25.5573	25.5417	25.6532	25.6375	25.6219	25.6063	25.5906	25.5750
**σ$H_3$24**	30.4185	30.4153	30.4366	30.4335	30.4303	30.4272	30.4241	30.4210
**σ$H_3$30**	30.2570	30.2618	30.2566	30.2615	30.2663	30.2711	30.2759	30.2807
**σ$H_3$29**	30.1691	30.1460	30.3092	30.2861	30.2630	30.2399	30.2168	30.1937
**σH4**	25.1539	25.2216	24.7464	24.8140	24.8817	24.9494	25.0171	25.0847

**σH5a**	29.6557	29.6416	29.7440	29.7298	29.7156	29.7015	29.6873	29.6732
**σH5b**	29.4098	29.4614	29.0923	29.1440	29.1956	29.2473	29.2989	29.3505
**σH3**	25.6170	25.6046	25.6828	25.6703	25.6579	25.6454	25.6330	25.6205
**σ$H_3$26**	30.2039	30.1785	30.3387	30.3133	30.2880	30.2626	30.2372	30.2118
**σ$H_3$1**	30.1826	30.1820	30.1845	30.1840	30.1834	30.1829	30.1823	30.1817
**σ$H_3$25**	30.4520	30.4567	30.4357	30.4404	30.4451	30.4497	30.4544	30.4591
**$^3J_{H4,H5}$**	6.25	5.83	8.76	8.34	7.92	7.50	7.09	6.67
**$^3J_{H4',H5}$**	7.89	8.33	5.25	5.69	6.12	6.56	6.99	7.43
**$^3J_{H20,H21}$**	4.48	4.47	4.56	4.54	4.52	4.50	4.48	4.47
**$^3J_{H20',H21}$**	8.87	8.87	8.86	8.86	8.86	8.86	8.86	8.86

**Conformer 5-1**	**10%**	**10%**	**10%**	**10%**	**10%**	**10%**	**10%**	**10%**
**Conformer 5-2**	**20%**	**20%**	**10%**	**10%**	**10%**	**10%**	**10%**	**10%**
**Conformer 5-5**	**10%**	**0%**	**80%**	**70%**	**60%**	**50%**	**40%**	**30%**
**Conformer 5-9**	**60%**	**70%**	**0%**	**10%**	**20%**	**30%**	**40%**	**50%**
**σC12**	164.3650	164.3860	164.1857	164.2067	164.2277	164.2487	164.2697	164.2906
**σC11**	107.6179	107.4061	109.0815	108.8697	108.6579	108.4460	108.2342	108.0224
**σC14**	110.8783	111.2138	108.4222	108.7577	109.0931	109.4285	109.7640	110.0994
**σC13**	163.7771	163.6797	164.4376	164.3402	164.2427	164.1453	164.0478	163.9504
**σC8**	167.1967	167.3298	166.3104	166.4435	166.5766	166.7097	166.8428	166.9759
**σC7**	109.6834	109.4742	111.1916	110.9824	110.7732	110.5639	110.3547	110.1455
**σC10**	108.9698	108.8485	109.6972	109.5758	109.4544	109.3331	109.2117	109.0903
**σC9**	161.5248	161.3558	162.6005	162.4315	162.2626	162.0936	161.9246	161.7556
**σC16**	164.3009	164.6591	161.6664	162.0246	162.3827	162.7409	163.0991	163.4572
**σC15**	110.1350	110.1461	110.2218	110.2329	110.2440	110.2551	110.2662	110.2772
**σC18**	111.9090	112.0912	110.7552	110.9374	111.1197	111.3019	111.4841	111.6663
**σC17**	167.6329	168.1264	163.9172	164.4108	164.9043	165.3978	165.8914	166.3849
**σC6**	118.5273	118.2683	120.2296	119.9707	119.7117	119.4528	119.1938	118.9349
**σC19**	121.0657	120.9331	122.0744	121.9419	121.8093	121.6768	121.5442	121.4117
**σC28**	173.1191	173.2981	171.7798	171.9587	172.1376	172.3165	172.4955	172.6744
**σC27**	170.2843	170.0788	171.6395	171.4340	171.2284	171.0229	170.8173	170.6118
**σC20**	150.3457	150.0239	152.3546	152.0328	151.7110	151.3892	151.0674	150.7456
**σC21**	71.2766	71.1328	71.8605	71.7166	71.5728	71.4290	71.2851	71.1413
**σC22**	59.0427	59.0137	59.7460	59.7170	59.6880	59.6591	59.6301	59.6012
**σC23**	123.4676	123.5347	122.6714	122.7386	122.8057	122.8728	122.9400	123.0071
**σC24**	166.2802	166.0886	167.0793	166.8878	166.6962	166.5046	166.3130	166.1215
**σC30**	167.6682	167.9439	166.0373	166.3130	166.5887	166.8644	167.1402	167.4159
**σC29**	169.3723	169.5745	167.4263	167.6285	167.8307	168.0329	168.2351	168.4374
**σC4**	70.2207	70.1718	71.0045	70.9557	70.9069	70.8580	70.8092	70.7604
**σC5**	151.8732	151.6870	153.0087	152.8226	152.6364	152.4502	152.2640	152.0779
**σC2**	114.5039	114.6300	113.6162	113.7423	113.8683	113.9944	114.1204	114.2465
**σC3**	60.1775	60.2096	59.7051	59.7371	59.7692	59.8013	59.8333	59.8654
**σC26**	172.2204	172.3561	171.1965	171.3321	171.4678	171.6035	171.7392	171.8749
**σC1**	174.2084	174.5821	171.6432	172.0169	172.3906	172.7642	173.1379	173.5116
**σC25**	168.5177	168.6406	167.6462	167.7691	167.8920	168.0148	168.1377	168.2605
**σH12a**	29.7233	29.7209	29.7381	29.7357	29.7333	29.7309	29.7284	29.7260
**σH12b**	30.0142	30.0006	30.1079	30.0943	30.0807	30.0671	30.0535	30.0399
**σH11**	27.3304	27.3190	27.4063	27.3949	27.3834	27.3720	27.3606	27.3492
**σH14**	27.2683	27.2580	27.3456	27.3353	27.3250	27.3146	27.3043	27.2939
**σH13a**	29.5545	29.5326	29.7094	29.6876	29.6657	29.6438	29.6220	29.6001

**σH13b**	29.9529	29.9293	30.1154	30.0917	30.0681	30.0445	30.0209	29.9972
**σH8a**	29.8100	29.7951	29.9041	29.8892	29.8744	29.8595	29.8446	29.8297
**σH8b**	29.0658	29.0230	29.3470	29.3042	29.2614	29.2186	29.1758	29.1330
**σH7**	27.5012	27.4560	27.8042	27.7589	27.7137	27.6684	27.6232	27.5779
**σH9a**	29.4509	29.4640	29.3744	29.3874	29.4005	29.4136	29.4267	29.4398
**σH9b**	30.0269	30.0131	30.1292	30.1154	30.1016	30.0879	30.0741	30.0604
**σH16a**	30.1467	30.1472	30.1497	30.1503	30.1509	30.1514	30.1520	30.1525
**σH16b**	29.4999	29.4984	29.5021	29.5006	29.4991	29.4976	29.4961	29.4946
**σH18**	27.7561	27.7630	27.7163	27.7231	27.7300	27.7368	27.7437	27.7505
**σH17a**	29.4714	29.4517	29.6200	29.6003	29.5806	29.5609	29.5412	29.5215
**σH17b**	29.7240	29.6882	29.9520	29.9161	29.8803	29.8444	29.8085	29.7726
**σ$H_3$28**	30.5887	30.5956	30.5419	30.5488	30.5557	30.5625	30.5694	30.5763
**σ$H_3$27**	30.5034	30.5010	30.5186	30.5161	30.5137	30.5112	30.5088	30.5063
**σH20a**	29.1251	29.1142	29.1348	29.1238	29.1129	29.1020	29.0911	29.0802
**σH20b**	29.4804	29.4729	29.5303	29.5228	29.5153	29.5078	29.5003	29.4927
**σH21**	25.1331	25.1912	24.7039	24.7619	24.8200	24.8780	24.9361	24.9942
**σH22**	25.5594	25.5437	25.6709	25.6553	25.6396	25.6240	25.6084	25.5927
**σ$H_3$24**	30.4178	30.4147	30.4391	30.4360	30.4328	30.4297	30.4266	30.4235
**σ$H_3$30**	30.2856	30.2904	30.2804	30.2852	30.2900	30.2948	30.2996	30.3045
**σ$H_3$29**	30.1706	30.1475	30.3338	30.3107	30.2876	30.2645	30.2414	30.2183
**σH4**	25.1524	25.2201	24.6772	24.7449	24.8125	24.8802	24.9479	25.0155
**σH5a**	29.6590	29.6448	29.7614	29.7472	29.7331	29.7189	29.7047	29.6906
**σH5b**	29.4022	29.4538	29.0331	29.0847	29.1364	29.1880	29.2397	29.2913
**σH3**	25.6081	25.5956	25.6863	25.6738	25.6614	25.6489	25.6365	25.6240
**σ$H_3$26**	30.1864	30.1610	30.3466	30.3212	30.2959	30.2705	30.2451	30.2197
**σ$H_3$1**	30.1812	30.1806	30.1837	30.1831	30.1826	30.1820	30.1815	30.1809
**σ$H_3$25**	30.4638	30.4685	30.4428	30.4474	30.4521	30.4568	30.4615	30.4662
**$^3J_{H4,H5}$**	6.25	5.83	9.18	8.76	8.34	7.92	7.50	7.09
**$^3J_{H4',H5}$**	7.86	8.30	4.79	5.22	5.66	6.09	6.53	6.96
**$^3J_{H20,H21}$**	4.45	4.43	4.54	4.52	4.50	4.48	4.47	4.45
**$^3J_{H20',H21}$**	8.86	8.86	8.85	8.85	8.85	8.85	8.85	8.85

**Conformer 5-1**	**10%**	**10%**	**10%**	**10%**	**10%**	**10%**	**10%**	**10%**
**Conformer 5-2**	**10%**	**10%**	**10%**	**0%**	**0%**	**0%**	**0%**	**0%**
**Conformer 5-5**	**20%**	**10%**	**0%**	**90%**	**80%**	**70%**	**60%**	**50%**
**Conformer 5-9**	**60%**	**70%**	**80%**	**0%**	**10%**	**20%**	**30%**	**40%**
**σC12**	164.3116	164.3326	164.3536	164.1323	164.1533	164.1743	164.1952	164.2162
**σC11**	107.8105	107.5987	107.3869	109.2742	109.0623	108.8505	108.6387	108.4268
**σC14**	110.4349	110.7703	111.1057	107.9788	108.3142	108.6497	108.9851	109.3205
**σC13**	163.8529	163.7555	163.6580	164.5134	164.4160	164.3185	164.2211	164.1236
**σC8**	167.1090	167.2421	167.3752	166.2227	166.3558	166.4889	166.6220	166.7551
**σC7**	109.9362	109.7270	109.5178	111.4444	111.2352	111.0260	110.8167	110.6075
**σC10**	108.9689	108.8476	108.7262	109.6963	109.5749	109.4535	109.3321	109.2108
**σC9**	161.5866	161.4177	161.2487	162.6624	162.4934	162.3244	162.1554	161.9864
**σC16**	163.8154	164.1736	164.5318	161.1809	161.5391	161.8973	162.2554	162.6136
**σC15**	110.2883	110.2994	110.3105	110.3751	110.3862	110.3973	110.4084	110.4195
**σC18**	111.8486	112.0308	112.2130	110.6948	110.8770	111.0593	111.2415	111.4237
**σC17**	166.8785	167.3720	167.8655	163.1628	163.6564	164.1499	164.6434	165.1370

**σC6**	118.6759	118.4170	118.1580	120.3783	120.1193	119.8604	119.6014	119.3425
**σC19**	121.2792	121.1466	121.0141	122.2879	122.1553	122.0228	121.8903	121.7577
**σC28**	172.8533	173.0322	173.2112	171.5139	171.6929	171.8718	172.0507	172.2296
**σC27**	170.4062	170.2007	169.9951	171.7614	171.5559	171.3503	171.1448	170.9392
**σC20**	150.4238	150.1020	149.7803	152.4327	152.1109	151.7891	151.4673	151.1456
**σC21**	70.9975	70.8536	70.7098	71.5813	71.4375	71.2936	71.1498	71.0060
**σC22**	59.5722	59.5432	59.5143	60.2755	60.2465	60.2176	60.1886	60.1597
**σC23**	123.0743	123.1414	123.2085	122.2781	122.3453	122.4124	122.4795	122.5467
**σC24**	165.9299	165.7383	165.5467	166.7290	166.5375	166.3459	166.1543	165.9627
**σC30**	167.6916	167.9674	168.2431	166.0607	166.3364	166.6122	166.8879	167.1636
**σC29**	168.6396	168.8418	169.0440	166.6936	166.8958	167.0980	167.3002	167.5024
**σC4**	70.7116	70.6628	70.6140	71.4954	71.4466	71.3978	71.3490	71.3002
**σC5**	151.8917	151.7055	151.5193	153.0272	152.8411	152.6549	152.4687	152.2825
**σC2**	114.3726	114.4986	114.6247	113.4848	113.6109	113.7369	113.8630	113.9891
**σC3**	59.8974	59.9295	59.9616	59.4250	59.4571	59.4891	59.5212	59.5533
**σC26**	172.0106	172.1462	172.2819	170.9866	171.1223	171.2579	171.3936	171.5293
**σC1**	173.8852	174.2589	174.6326	171.3200	171.6937	172.0674	172.4411	172.8147
**σC25**	168.3834	168.5063	168.6291	167.5119	167.6348	167.7576	167.8805	168.0034
**σH12a**	29.7236	29.7212	29.7188	29.7385	29.7360	29.7336	29.7312	29.7288
**σH12b**	30.0263	30.0128	29.9992	30.1201	30.1065	30.0929	30.0793	30.0657
**σH11**	27.3378	27.3263	27.3149	27.4136	27.4022	27.3908	27.3794	27.3679
**σH14**	27.2836	27.2733	27.2629	27.3609	27.3506	27.3402	27.3299	27.3196
**σH13a**	29.5783	29.5564	29.5346	29.7332	29.7114	29.6895	29.6677	29.6458
**σH13b**	29.9736	29.9500	29.9263	30.1361	30.1124	30.0888	30.0652	30.0415
**σH8a**	29.8148	29.7999	29.7850	29.9089	29.8941	29.8792	29.8643	29.8494
**σH8b**	29.0902	29.0474	29.0046	29.3713	29.3285	29.2858	29.2430	29.2002
**σH7**	27.5327	27.4874	27.4421	27.8356	27.7904	27.7451	27.6999	27.6546
**σH9a**	29.4529	29.4660	29.4791	29.3763	29.3894	29.4025	29.4156	29.4287
**σH9b**	30.0466	30.0328	30.0191	30.1488	30.1351	30.1213	30.1076	30.0938
**σH16a**	30.1531	30.1536	30.1542	30.1562	30.1567	30.1573	30.1578	30.1584
**σH16b**	29.4931	29.4916	29.4901	29.4952	29.4937	29.4922	29.4907	29.4892
**σH18**	27.7574	27.7642	27.7711	27.7175	27.7244	27.7312	27.7381	27.7449
**σH17a**	29.5018	29.4821	29.4624	29.6504	29.6307	29.6110	29.5913	29.5716
**σH17b**	29.7367	29.7008	29.6649	29.9647	29.9288	29.8929	29.8570	29.8211
**σH$_3$28**	30.5832	30.5900	30.5969	30.5364	30.5433	30.5501	30.5570	30.5639
**σH$_3$27**	30.5039	30.5014	30.4990	30.5190	30.5166	30.5141	30.5117	30.5092
**σH20a**	29.0693	29.0584	29.0475	29.0790	29.0681	29.0572	29.0463	29.0354
**σH20b**	29.4852	29.4777	29.4702	29.5351	29.5276	29.5201	29.5126	29.5051
**σH21**	25.0522	25.1103	25.1683	24.6230	24.6810	24.7391	24.7971	24.8552
**σH22**	25.5771	25.5615	25.5458	25.6886	25.6730	25.6574	25.6417	25.6261
**σH$_3$24**	30.4203	30.4172	30.4141	30.4416	30.4385	30.4353	30.4322	30.4291
**σH$_3$30**	30.3093	30.3141	30.3189	30.3041	30.3089	30.3137	30.3185	30.3234
**σH$_3$29**	30.1952	30.1721	30.1490	30.3584	30.3353	30.3122	30.2891	30.2660
**σH4**	25.0832	25.1509	25.2186	24.6080	24.6757	24.7434	24.8110	24.8787
**σH5a**	29.6764	29.6622	29.6481	29.7788	29.7646	29.7505	29.7363	29.7222
**σH5b**	29.3429	29.3946	29.4462	28.9739	29.0255	29.0771	29.1288	29.1804
**σH3**	25.6116	25.5991	25.5867	25.6898	25.6773	25.6649	25.6524	25.6400
**σH$_3$26**	30.1943	30.1689	30.1435	30.3545	30.3292	30.3038	30.2784	30.2530
**σH$_3$1**	30.1804	30.1798	30.1792	30.1829	30.1823	30.1818	30.1812	30.1807

**σ$H_3$25**	30.4709	30.4756	30.4803	30.4498	30.4545	30.4592	30.4639	30.4686
**$^3J_{H4,H5}$**	6.67	6.25	5.83	9.60	9.18	8.76	8.34	7.92
**$^3J_{H4',H5}$**	7.40	7.84	8.27	4.32	4.76	5.20	5.63	6.07
**$^3J_{H20,H21}$**	4.43	4.41	4.39	4.52	4.50	4.48	4.47	4.45
**$^3J_{H20',H21}$**	8.85	8.85	8.85	8.84	8.84	8.84	8.84	8.84

**Conformer 5-1**	**10%**	**10%**	**10%**	**10%**	**10%**	**0%**	**0%**	**0%**
**Conformer 5-2**	**0%**	**0%**	**0%**	**0%**	**0%**	**100%**	**90%**	**90%**
**Conformer 5-5**	**40%**	**30%**	**20%**	**10%**	**0%**	**0%**	**10%**	**0%**
**Conformer 5-9**	**50%**	**60%**	**70%**	**80%**	**90%**	**0%**	**0%**	**10%**
**σC12**	164.2372	164.2582	164.2792	164.3002	164.3211	164.6787	164.6253	164.6463
**σC11**	108.2150	108.0032	107.7913	107.5795	107.3677	107.2235	107.4161	107.2043
**σC14**	109.6560	109.9914	110.3268	110.6623	110.9977	112.2614	111.8180	112.1534
**σC13**	164.0262	163.9287	163.8313	163.7338	163.6364	163.7485	163.8243	163.7269
**σC8**	166.8883	167.0214	167.1545	167.2876	167.4207	166.9250	166.8373	166.9705
**σC7**	110.3983	110.1890	109.9798	109.7706	109.5614	108.9300	109.1828	108.9736
**σC10**	109.0894	108.9680	108.8467	108.7253	108.6039	109.6153	109.6144	109.4930
**σC9**	161.8174	161.6485	161.4795	161.3105	161.1415	161.8357	161.8976	161.7286
**σC16**	162.9718	163.3300	163.6881	164.0463	164.4045	165.6690	165.1836	165.5418
**σC15**	110.4305	110.4416	110.4527	110.4638	110.4748	109.0985	109.2518	109.2629
**σC18**	111.6059	111.7882	111.9704	112.1526	112.3349	111.4501	111.3897	111.5719
**σC17**	165.6305	166.1241	166.6176	167.1111	167.6047	170.2261	169.4717	169.9652
**σC6**	119.0835	118.8246	118.5656	118.3067	118.0477	118.9037	119.0523	118.7934
**σC19**	121.6252	121.4926	121.3601	121.2276	121.0950	120.6744	120.8879	120.7554
**σC28**	172.4086	172.5875	172.7664	172.9454	173.1243	174.1466	173.8808	174.0597
**σC27**	170.7337	170.5281	170.3226	170.1170	169.9115	170.4284	170.5503	170.3447
**σC20**	150.8238	150.5020	150.1802	149.8584	149.5366	151.3764	151.4545	151.1327
**σC21**	70.8622	70.7183	70.5745	70.4307	70.2868	74.3333	74.0542	73.9103
**σC22**	60.1307	60.1017	60.0728	60.0438	60.0149	55.3303	55.8599	55.8309
**σC23**	122.6138	122.6810	122.7481	122.8152	122.8824	126.2055	125.8122	125.8793
**σC24**	165.7711	165.5796	165.3880	165.1964	165.0048	170.3192	169.9689	169.7773
**σC30**	167.4394	167.7151	167.9908	168.2665	168.5423	165.7758	165.7992	166.0749
**σC29**	167.7046	167.9069	168.1091	168.3113	168.5135	173.2575	172.5248	172.7270
**σC4**	71.2514	71.2026	71.1538	71.1049	71.0561	66.5886	67.0795	67.0307
**σC5**	152.0964	151.9102	151.7240	151.5378	151.3517	152.9932	153.0117	152.8255
**σC2**	114.1151	114.2412	114.3673	114.4933	114.6194	114.8350	114.7037	114.8297
**σC3**	59.5853	59.6174	59.6494	59.6815	59.7136	62.2032	61.9232	61.9552
**σC26**	171.6650	171.8007	171.9364	172.0721	172.2077	172.9846	172.7747	172.9104
**σC1**	173.1884	173.5621	173.9358	174.3094	174.6831	174.5708	174.2476	174.6213
**σC25**	168.1262	168.2491	168.3720	168.4948	168.6177	168.9328	168.7985	168.9214
**σH12a**	29.7264	29.7240	29.7215	29.7191	29.7167	29.7399	29.7402	29.7378
**σH12b**	30.0521	30.0385	30.0250	30.0114	29.9978	30.0025	30.0147	30.0011
**σH11**	27.3565	27.3451	27.3337	27.3222	27.3108	27.3491	27.3564	27.3450
**σH14**	27.3092	27.2989	27.2886	27.2782	27.2679	27.2294	27.2447	27.2344
**σH13a**	29.6239	29.6021	29.5802	29.5584	29.5365	29.5077	29.5315	29.5096
**σH13b**	30.0179	29.9943	29.9706	29.9470	29.9234	29.9408	29.9615	29.9378
**σH8a**	29.8345	29.8196	29.8047	29.7899	29.7750	29.8613	29.8661	29.8512
**σH8b**	29.1574	29.1146	29.0718	29.0290	28.9862	29.1002	29.1246	29.0818
**σH7**	27.6093	27.5641	27.5188	27.4736	27.4283	27.5151	27.5465	27.5012
**σH9a**	29.4418	29.4549	29.4680	29.4811	29.4941	29.3586	29.3606	29.3737
**σH9b**	30.0800	30.0663	30.0525	30.0388	30.0250	29.9481	29.9678	29.9541

**σH16a**	30.1589	30.1595	30.1600	30.1606	30.1612	30.1056	30.1120	30.1125
**σH16b**	29.4877	29.4862	29.4847	29.4832	29.4817	29.5777	29.5708	29.5693
**σH18**	27.7518	27.7586	27.7654	27.7723	27.7791	27.7176	27.7189	27.7257
**σH17a**	29.5519	29.5322	29.5126	29.4929	29.4732	29.3902	29.4206	29.4009
**σH17b**	29.7852	29.7493	29.7135	29.6776	29.6417	29.8950	29.9077	29.8718
**σ$H_3$28**	30.5708	30.5776	30.5845	30.5914	30.5982	30.5925	30.5870	30.5939
**σ$H_3$27**	30.5068	30.5043	30.5019	30.4994	30.4969	30.5105	30.5109	30.5085
**σH20a**	29.0244	29.0135	29.0026	28.9917	28.9808	29.5339	29.4782	29.4673
**σH20b**	29.4976	29.4901	29.4826	29.4750	29.4675	29.5134	29.5182	29.5107
**σH21**	24.9132	24.9713	25.0294	25.0874	25.1455	25.3781	25.2972	25.3553
**σH22**	25.6105	25.5948	25.5792	25.5636	25.5479	25.5423	25.5600	25.5444
**σ$H_3$24**	30.4260	30.4228	30.4197	30.4166	30.4135	30.4317	30.4342	30.4311
**σ$H_3$30**	30.3282	30.3330	30.3378	30.3426	30.3475	30.0927	30.1164	30.1212
**σ$H_3$29**	30.2429	30.2198	30.1967	30.1736	30.1505	30.1053	30.1299	30.1068
**σH4**	24.9464	25.0140	25.0817	25.1494	25.2171	25.2515	25.1824	25.2500
**σH5a**	29.7080	29.6938	29.6797	29.6655	29.6513	29.6045	29.6220	29.6078
**σH5b**	29.2321	29.2837	29.3353	29.3870	29.4386	29.5455	29.4862	29.5379
**σH3**	25.6275	25.6151	25.6027	25.5902	25.5778	25.6646	25.6681	25.6557
**σ$H_3$26**	30.2276	30.2022	30.1768	30.1515	30.1261	30.2831	30.2910	30.2656
**σ$H_3$1**	30.1801	30.1795	30.1790	30.1784	30.1779	30.1962	30.1954	30.1948
**σ$H_3$25**	30.4733	30.4780	30.4827	30.4874	30.4921	30.3788	30.3859	30.3906
**$^3J_{H4,H5}$**	7.50	7.09	6.67	6.25	5.83	5.40	5.82	5.40
**$^3J_{H4',H5}$**	6.50	6.94	7.37	7.81	8.24	8.97	8.50	8.94
**$^3J_{H20,H21}$**	4.43	4.41	4.39	4.37	4.36	4.12	4.10	4.08
**$^3J_{H20',H21}$**	8.84	8.84	8.84	8.84	8.84	9.42	9.42	9.42

**Conformer 5-1**	**0%**	**0%**	**0%**	**0%**	**0%**	**0%**	**0%**	**0%**
**Conformer 5-2**	**80%**	**80%**	**80%**	**70%**	**70%**	**70%**	**70%**	**60%**
**Conformer 5-5**	**20%**	**10%**	**0%**	**30%**	**20%**	**10%**	**0%**	**40%**
**Conformer 5-9**	**0%**	**10%**	**20%**	**0%**	**10%**	**20%**	**30%**	**0%**
**σC12**	164.5719	164.5929	164.6139	164.5185	164.5395	164.5604	164.5814	164.4650
**σC11**	107.6088	107.3969	107.1851	107.8014	107.5896	107.3777	107.1659	107.9940
**σC14**	111.3745	111.7100	112.0454	110.9311	111.2665	111.6019	111.9374	110.4876
**σC13**	163.9001	163.8027	163.7052	163.9759	163.8785	163.7810	163.6836	164.0517
**σC8**	166.7497	166.8828	167.0159	166.6620	166.7951	166.9282	167.0614	166.5744
**σC7**	109.4356	109.2264	109.0172	109.6884	109.4792	109.2700	109.0608	109.9413
**σC10**	109.6134	109.4921	109.3707	109.6125	109.4912	109.3698	109.2484	109.6116
**σC9**	161.9594	161.7904	161.6214	162.0212	161.8522	161.6833	161.5143	162.0831
**σC16**	164.6981	165.0563	165.4145	164.2126	164.5708	164.9290	165.2872	163.7272
**σC15**	109.4051	109.4162	109.4273	109.5584	109.5695	109.5806	109.5917	109.7117
**σC18**	111.3293	111.5116	111.6938	111.2689	111.4512	111.6334	111.8156	111.2086
**σC17**	168.7173	169.2108	169.7044	167.9629	168.4564	168.9499	169.4435	167.2085
**σC6**	119.2010	118.9420	118.6831	119.3496	119.0907	118.8317	118.5728	119.4983
**σC19**	121.1014	120.9689	120.8363	121.3149	121.1824	121.0498	120.9173	121.5284
**σC28**	173.6149	173.7939	173.9728	173.3491	173.5281	173.7070	173.8859	173.0833
**σC27**	170.6722	170.4666	170.2611	170.7941	170.5885	170.3830	170.1774	170.9159
**σC20**	151.5326	151.2108	150.8891	151.6108	151.2890	150.9672	150.6454	151.6889
**σC21**	73.7750	73.6312	73.4873	73.4959	73.3520	73.2082	73.0644	73.2167

**σC22**	56.3894	56.3604	56.3315	56.9189	56.8900	56.8610	56.8321	57.4485
**σC23**	125.4189	125.4860	125.5532	125.0256	125.0927	125.1599	125.2270	124.6323
**σC24**	169.6186	169.4270	169.2355	169.2683	169.0767	168.8851	168.6936	168.9180
**σC30**	165.8227	166.0984	166.3741	165.8461	166.1219	166.3976	166.6733	165.8696
**σC29**	171.7921	171.9943	172.1965	171.0594	171.2616	171.4638	171.6660	170.3267
**σC4**	67.5705	67.5217	67.4728	68.0614	68.0126	67.9638	67.9150	68.5524
**σC5**	153.0302	152.8440	152.6578	153.0487	152.8625	152.6764	152.4902	153.0672
**σC2**	114.5723	114.6984	114.8244	114.4409	114.5670	114.6931	114.8191	114.3096
**σC3**	61.6431	61.6752	61.7072	61.3630	61.3951	61.4272	61.4592	61.0830
**σC26**	172.5648	172.7005	172.8362	172.3549	172.4906	172.6263	172.7620	172.1450
**σC1**	173.9245	174.2981	174.6718	173.6013	173.9750	174.3486	174.7223	173.2781
**σC25**	168.6642	168.7870	168.9099	168.5299	168.6527	168.7756	168.8985	168.3956
**σH12a**	29.7406	29.7382	29.7357	29.7409	29.7385	29.7361	29.7337	29.7413
**σH12b**	30.0268	30.0133	29.9997	30.0390	30.0255	30.0119	29.9983	30.0512
**σH11**	27.3637	27.3523	27.3409	27.3711	27.3597	27.3482	27.3368	27.3784
**σH14**	27.2600	27.2497	27.2393	27.2753	27.2649	27.2546	27.2443	27.2906
**σH13a**	29.5553	29.5335	29.5116	29.5791	29.5573	29.5354	29.5136	29.6029
**σH13b**	29.9821	29.9585	29.9349	30.0028	29.9792	29.9556	29.9319	30.0235
**σH8a**	29.8709	29.8560	29.8411	29.8757	29.8608	29.8459	29.8310	29.8805
**σH8b**	29.1490	29.1062	29.0634	29.1734	29.1306	29.0878	29.0450	29.1977
**σH7**	27.5779	27.5327	27.4874	27.6094	27.5641	27.5188	27.4736	27.6408
**σH9a**	29.3626	29.3757	29.3888	29.3646	29.3777	29.3908	29.4038	29.3666
**σH9b**	29.9875	29.9738	29.9600	30.0072	29.9934	29.9797	29.9659	30.0269
**σH16a**	30.1184	30.1189	30.1195	30.1248	30.1253	30.1259	30.1265	30.1312
**σH16b**	29.5640	29.5625	29.5610	29.5572	29.5557	29.5542	29.5527	29.5503
**σH18**	27.7201	27.7270	27.7338	27.7213	27.7282	27.7350	27.7419	27.7226
**σH17a**	29.4510	29.4313	29.4116	29.4814	29.4617	29.4420	29.4224	29.5118
**σH17b**	29.9203	29.8844	29.8485	29.9330	29.8971	29.8612	29.8253	29.9456
**σ$H_3$28**	30.5815	30.5883	30.5952	30.5759	30.5828	30.5897	30.5966	30.5704
**σ$H_3$27**	30.5114	30.5089	30.5065	30.5118	30.5094	30.5069	30.5045	30.5123
**σH20a**	29.4224	29.4115	29.4006	29.3666	29.3557	29.3448	29.3339	29.3109
**σH20b**	29.5230	29.5155	29.5080	29.5279	29.5204	29.5129	29.5053	29.5327
**σH21**	25.2163	25.2744	25.3324	25.1354	25.1934	25.2515	25.3096	25.0545
**σH22**	25.5778	25.5621	25.5465	25.5955	25.5798	25.5642	25.5486	25.6132
**σ$H_3$24**	30.4367	30.4336	30.4305	30.4392	30.4361	30.4329	30.4298	30.4417
**σ$H_3$30**	30.1401	30.1450	30.1498	30.1639	30.1687	30.1735	30.1783	30.1876
**σ$H_3$29**	30.1545	30.1314	30.1083	30.1791	30.1560	30.1330	30.1099	30.2038
**σH4**	25.1132	25.1809	25.2485	25.0440	25.1117	25.1794	25.2470	24.9748
**σH5a**	29.6394	29.6252	29.6110	29.6568	29.6426	29.6285	29.6143	29.6742
**σH5b**	29.4270	29.4786	29.5303	29.3677	29.4194	29.4710	29.5226	29.3085
**σH3**	25.6716	25.6592	25.6467	25.6751	25.6627	25.6502	25.6378	25.6786
**σ$H_3$26**	30.2989	30.2735	30.2481	30.3068	30.2814	30.2560	30.2306	30.3147
**σ$H_3$1**	30.1946	30.1940	30.1935	30.1937	30.1932	30.1926	30.1921	30.1929
**σ$H_3$25**	30.3930	30.3977	30.4024	30.4001	30.4048	30.4095	30.4142	30.4072
**$^3J_{H4,H5}$**	6.24	5.82	5.40	6.66	6.24	5.82	5.40	7.07
**$^3J_{H4',H5}$**	8.04	8.48	8.91	7.58	8.01	8.45	8.88	7.12
**$^3J_{H20,H21}$**	4.08	4.06	4.04	4.06	4.04	4.03	4.01	4.04
**$^3J_{H20',H21}$**	9.41	9.41	9.41	9.40	9.40	9.40	9.40	9.39

**Conformer 5-1**	**0%**	**0%**	**0%**	**0%**	**0%**	**0%**	**0%**	**0%**
**Conformer 5-2**	**60%**	**60%**	**60%**	**60%**	**50%**	**50%**	**50%**	**50%**
**Conformer 5-5**	**30%**	**20%**	**10%**	**0%**	**50%**	**40%**	**30%**	**20%**
**Conformer 5-9**	**10%**	**20%**	**30%**	**40%**	**0%**	**10%**	**20%**	**30%**
**σC12**	164.4860	164.5070	164.5280	164.5490	164.4116	164.4326	164.4536	164.4746
**σC11**	107.7822	107.5704	107.3585	107.1467	108.1867	107.9749	107.7630	107.5512
**σC14**	110.8230	111.1585	111.4939	111.8293	110.0441	110.3796	110.7150	111.0504
**σC13**	163.9543	163.8568	163.7594	163.6619	164.1275	164.0301	163.9326	163.8352
**σC8**	166.7075	166.8406	166.9737	167.1068	166.4867	166.6198	166.7529	166.8860
**σC7**	109.7320	109.5228	109.3136	109.1043	110.1941	109.9848	109.7756	109.5664
**σC10**	109.4903	109.3689	109.2475	109.1262	109.6107	109.4894	109.3680	109.2466
**σC9**	161.9141	161.7451	161.5761	161.4071	162.1449	161.9759	161.8069	161.6379
**σC16**	164.0854	164.4435	164.8017	165.1599	163.2417	163.5999	163.9581	164.3162
**σC15**	109.7228	109.7339	109.7450	109.7560	109.8650	109.8761	109.8872	109.8983
**σC18**	111.3908	111.5730	111.7552	111.9375	111.1482	111.3304	111.5126	111.6948
**σC17**	167.7020	168.1955	168.6891	169.1826	166.4540	166.9476	167.4411	167.9347
**σC6**	119.2393	118.9804	118.7214	118.4625	119.6469	119.3880	119.1290	118.8701
**σC19**	121.3958	121.2633	121.1308	120.9982	121.7419	121.6093	121.4768	121.3443
**σC28**	173.2622	173.4412	173.6201	173.7990	172.8175	172.9964	173.1754	173.3543
**σC27**	170.7104	170.5049	170.2993	170.0938	171.0378	170.8323	170.6267	170.4212
**σC20**	151.3671	151.0453	150.7235	150.4017	151.7670	151.4452	151.1235	150.8017
**σC21**	73.0729	72.9290	72.7852	72.6414	72.9376	72.7937	72.6499	72.5061
**σC22**	57.4195	57.3906	57.3616	57.3326	57.9780	57.9491	57.9201	57.8911
**σC23**	124.6994	124.7666	124.8337	124.9008	124.2390	124.3061	124.3733	124.4404
**σC24**	168.7264	168.5348	168.3433	168.1517	168.5677	168.3761	168.1845	167.9930
**σC30**	166.1453	166.4210	166.6968	166.9725	165.8930	166.1688	166.4445	166.7202
**σC29**	170.5289	170.7311	170.9333	171.1355	169.5939	169.7962	169.9984	170.2006
**σC4**	68.5036	68.4548	68.4059	68.3571	69.0433	68.9945	68.9457	68.8969
**σC5**	152.8810	152.6949	152.5087	152.3225	153.0857	152.8995	152.7134	152.5272
**σC2**	114.4356	114.5617	114.6877	114.8138	114.1782	114.3043	114.4303	114.5564
**σC3**	61.1150	61.1471	61.1792	61.2112	60.8029	60.8350	60.8670	60.8991
**σC26**	172.2807	172.4164	172.5521	172.6878	171.9352	172.0708	172.2065	172.3422
**σC1**	173.6518	174.0255	174.3992	174.7728	172.9550	173.3286	173.7023	174.0760
**σC25**	168.5184	168.6413	168.7642	168.8870	168.2613	168.3841	168.5070	168.6299
**σH12a**	29.7389	29.7364	29.7340	29.7316	29.7416	29.7392	29.7368	29.7344
**σH12b**	30.0376	30.0241	30.0105	29.9969	30.0634	30.0498	30.0362	30.0227
**σH11**	27.3670	27.3556	27.3441	27.3327	27.3858	27.3743	27.3629	27.3515
**σH14**	27.2802	27.2699	27.2596	27.2492	27.3059	27.2955	27.2852	27.2748
**σH13a**	29.5811	29.5592	29.5374	29.5155	29.6268	29.6049	29.5831	29.5612
**σH13b**	29.9999	29.9762	29.9526	29.9290	30.0442	30.0206	29.9969	29.9733
**σH8a**	29.8656	29.8507	29.8358	29.8210	29.8853	29.8704	29.8555	29.8407
**σH8b**	29.1550	29.1122	29.0694	29.0266	29.2221	29.1793	29.1365	29.0938
**σH7**	27.5955	27.5503	27.5050	27.4598	27.6722	27.6270	27.5817	27.5364
**σH9a**	29.3797	29.3927	29.4058	29.4189	29.3686	29.3817	29.3947	29.4078
**σH9b**	30.0131	29.9994	29.9856	29.9718	30.0466	30.0328	30.0191	30.0053
**σH16a**	30.1318	30.1323	30.1329	30.1334	30.1376	30.1382	30.1387	30.1393
**σH16b**	29.5488	29.5473	29.5458	29.5443	29.5435	29.5420	29.5405	29.5390
**σH18**	27.7294	27.7363	27.7431	27.7499	27.7238	27.7306	27.7375	27.7443
**σH17a**	29.4922	29.4725	29.4528	29.4331	29.5423	29.5226	29.5029	29.4832
**σH17b**	29.9097	29.8738	29.8379	29.8020	29.9583	29.9224	29.8865	29.8506
**σ$H_3$28**	30.5773	30.5841	30.5910	30.5979	30.5649	30.5717	30.5786	30.5855

**σH$_3$27**	30.5098	30.5074	30.5049	30.5025	30.5127	30.5103	30.5078	30.5054
**σH20a**	29.3000	29.2891	29.2782	29.2672	29.2551	29.2442	29.2333	29.2224
**σH20b**	29.5252	29.5177	29.5102	29.5027	29.5375	29.5300	29.5225	29.5150
**σH21**	25.1125	25.1706	25.2287	25.2867	24.9736	25.0316	25.0897	25.1477
**σH22**	25.5976	25.5819	25.5663	25.5507	25.6309	25.6153	25.5997	25.5840
**σH$_3$24**	30.4386	30.4354	30.4323	30.4292	30.4442	30.4411	30.4379	30.4348
**σH$_3$30**	30.1924	30.1972	30.2020	30.2069	30.2113	30.2161	30.2209	30.2258
**σH$_3$29**	30.1807	30.1576	30.1345	30.1114	30.2284	30.2053	30.1822	30.1591
**σH4**	25.0425	25.1102	25.1778	25.2455	24.9057	24.9733	25.0410	25.1087
**σH5a**	29.6600	29.6459	29.6317	29.6176	29.6916	29.6775	29.6633	29.6491
**σH5b**	29.3601	29.4118	29.4634	29.5150	29.2492	29.3009	29.3525	29.4042
**σH3**	25.6662	25.6537	25.6413	25.6289	25.6821	25.6697	25.6572	25.6448
**σH$_3$26**	30.2893	30.2639	30.2385	30.2132	30.3226	30.2972	30.2718	30.2465
**σH$_3$1**	30.1924	30.1918	30.1912	30.1907	30.1921	30.1915	30.1910	30.1904
**σH$_3$25**	30.4119	30.4165	30.4212	30.4259	30.4142	30.4189	30.4236	30.4283
$^3J_{H4,H5}$	6.66	6.24	5.82	5.40	7.49	7.07	6.66	6.24
$^3J_{H4',H5}$	7.55	7.99	8.42	8.86	6.65	7.09	7.52	7.96
$^3J_{H20,H21}$	4.03	4.01	3.99	3.97	4.03	4.01	3.99	3.97
$^3J_{H20',H21}$	9.39	9.39	9.39	9.39	9.38	9.38	9.38	9.38
**Conformer 5-1**	**0%**	**0%**	**0%**	**0%**	**0%**	**0%**	**0%**	**0%**
**Conformer 5-2**	**50%**	**50%**	**40%**	**40%**	**40%**	**40%**	**40%**	**40%**
**Conformer 5-5**	**10%**	**0%**	**60%**	**50%**	**40%**	**30%**	**20%**	**10%**
**Conformer 5-9**	**40%**	**50%**	**0%**	**10%**	**20%**	**30%**	**40%**	**50%**
**σC12**	164.4955	164.5165	164.3582	164.3792	164.4002	164.4211	164.4421	164.4631
**σC11**	107.3394	107.1275	108.3793	108.1675	107.9557	107.7438	107.5320	107.3202
**σC14**	111.3859	111.7213	109.6007	109.9361	110.2715	110.6070	110.9424	111.2779
**σC13**	163.7377	163.6403	164.2034	164.1059	164.0085	163.9110	163.8136	163.7161
**σC8**	167.0191	167.1523	166.3990	166.5322	166.6653	166.7984	166.9315	167.0646
**σC7**	109.3571	109.1479	110.4469	110.2376	110.0284	109.8192	109.6099	109.4007
**σC10**	109.1252	109.0039	109.6098	109.4885	109.3671	109.2457	109.1243	109.0030
**σC9**	161.4690	161.3000	162.2067	162.0377	161.8687	161.6998	161.5308	161.3618
**σC16**	164.6744	165.0326	162.7562	163.1144	163.4726	163.8308	164.1889	164.5471
**σC15**	109.9093	109.9204	110.0183	110.0294	110.0405	110.0516	110.0626	110.0737
**σC18**	111.8771	112.0593	111.0878	111.2700	111.4522	111.6345	111.8167	111.9989
**σC17**	168.4282	168.9217	165.6996	166.1932	166.6867	167.1803	167.6738	168.1673
**σC6**	118.6111	118.3522	119.7956	119.5366	119.2777	119.0187	118.7598	118.5008
**σC19**	121.2117	121.0792	121.9554	121.8228	121.6903	121.5577	121.4252	121.2927
**σC28**	173.5332	173.7121	172.5517	172.7306	172.9095	173.0885	173.2674	173.4463
**σC27**	170.2156	170.0101	171.1597	170.9542	170.7486	170.5431	170.3375	170.1320
**σC20**	150.4799	150.1581	151.8452	151.5234	151.2016	150.8798	150.5580	150.2362
**σC21**	72.3622	72.2184	72.6584	72.5146	72.3707	72.2269	72.0831	71.9393
**σC22**	57.8622	57.8332	58.5075	58.4786	58.4496	58.4207	58.3917	58.3628
**σC23**	124.5075	124.5747	123.8457	123.9128	123.9800	124.0471	124.1142	124.1814
**σC24**	167.8014	167.6098	168.2174	168.0258	167.8342	167.6427	167.4511	167.2595
**σC30**	166.9959	167.2717	165.9165	166.1922	166.4679	166.7437	167.0194	167.2951
**σC29**	170.4028	170.6050	168.8612	169.0634	169.2657	169.4679	169.6701	169.8723
**σC4**	68.8481	68.7993	69.5343	69.4855	69.4367	69.3879	69.3390	69.2902
**σC5**	152.3410	152.1548	153.1042	152.9181	152.7319	152.5457	152.3595	152.1733

**σC2**	114.6824	114.8085	114.0468	114.1729	114.2990	114.4250	114.5511	114.6771
**σC3**	60.9312	60.9632	60.5228	60.5549	60.5870	60.6190	60.6511	60.6832
**σC26**	172.4779	172.6136	171.7253	171.8610	171.9966	172.1323	172.2680	172.4037
**σC1**	174.4497	174.8233	172.6318	173.0055	173.3792	173.7528	174.1265	174.5002
**σC25**	168.7527	168.8756	168.1270	168.2498	168.3727	168.4956	168.6184	168.7413
**σH12a**	29.7320	29.7295	29.7420	29.7395	29.7371	29.7347	29.7323	29.7299
**σH12b**	30.0091	29.9955	30.0756	30.0620	30.0484	30.0348	30.0213	30.0077
**σH11**	27.3401	27.3286	27.3931	27.3817	27.3702	27.3588	27.3474	27.3360
**σH14**	27.2645	27.2542	27.3211	27.3108	27.3005	27.2901	27.2798	27.2695
**σH13a**	29.5393	29.5175	29.6506	29.6287	29.6069	29.5850	29.5632	29.5413
**σH13b**	29.9497	29.9260	30.0649	30.0412	30.0176	29.9940	29.9704	29.9467
**σH8a**	29.8258	29.8109	29.8901	29.8752	29.8603	29.8455	29.8306	29.8157
**σH8b**	29.0510	29.0082	29.2465	29.2037	29.1609	29.1181	29.0753	29.0325
**σH7**	27.4912	27.4459	27.7037	27.6584	27.6131	27.5679	27.5226	27.4774
**σH9a**	29.4209	29.4340	29.3706	29.3836	29.3967	29.4098	29.4229	29.4360
**σH9b**	29.9915	29.9778	30.0663	30.0525	30.0387	30.0250	30.0112	29.9975
**σH16a**	30.1398	30.1404	30.1440	30.1446	30.1451	30.1457	30.1462	30.1468
**σH16b**	29.5375	29.5360	29.5367	29.5352	29.5337	29.5322	29.5307	29.5292
**σH18**	27.7512	27.7580	27.7250	27.7319	27.7387	27.7456	27.7524	27.7593
**σH17a**	29.4635	29.4438	29.5727	29.5530	29.5333	29.5136	29.4939	29.4742
**σH17b**	29.8147	29.7788	29.9709	29.9350	29.8991	29.8632	29.8273	29.7915
**σ$H_3$28**	30.5924	30.5992	30.5593	30.5662	30.5731	30.5800	30.5868	30.5937
**σ$H_3$27**	30.5029	30.5004	30.5132	30.5107	30.5083	30.5058	30.5033	30.5009
**σH20a**	29.2115	29.2006	29.1993	29.1884	29.1775	29.1666	29.1557	29.1448
**σH20b**	29.5075	29.5000	29.5424	29.5349	29.5274	29.5198	29.5123	29.5048
**σH21**	25.2058	25.2639	24.8927	24.9507	25.0088	25.0668	25.1249	25.1830
**σH22**	25.5684	25.5528	25.6486	25.6330	25.6174	25.6017	25.5861	25.5705
**σ$H_3$24**	30.4317	30.4286	30.4467	30.4436	30.4404	30.4373	30.4342	30.4311
**σ$H_3$30**	30.2306	30.2354	30.2350	30.2398	30.2447	30.2495	30.2543	30.2591
**σ$H_3$29**	30.1360	30.1129	30.2530	30.2299	30.2068	30.1837	30.1606	30.1375
**σH4**	25.1763	25.2440	24.8365	24.9042	24.9718	25.0395	25.1072	25.1748
**σH5a**	29.6350	29.6208	29.7091	29.6949	29.6807	29.6666	29.6524	29.6382
**σH5b**	29.4558	29.5074	29.1900	29.2416	29.2933	29.3449	29.3965	29.4482
**σH3**	25.6324	25.6199	25.6856	25.6732	25.6607	25.6483	25.6359	25.6234
**σ$H_3$26**	30.2211	30.1957	30.3305	30.3051	30.2798	30.2544	30.2290	30.2036
**σ$H_3$1**	30.1899	30.1893	30.1913	30.1907	30.1902	30.1896	30.1890	30.1885
**σ$H_3$25**	30.4330	30.4377	30.4213	30.4260	30.4307	30.4354	30.4401	30.4448
$^3J_{H4,H5}$	5.82	5.40	7.91	7.49	7.07	6.66	6.24	5.82
$^3J_{H4',H5}$	8.39	8.83	6.19	6.62	7.06	7.50	7.93	8.37
$^3J_{H20,H21}$	3.95	3.93	4.01	3.99	3.97	3.95	3.93	3.92
$^3J_{H20',H21}$	9.38	9.38	9.37	9.37	9.37	9.37	9.37	9.37

**Conformer 5-1**	**0%**	**0%**	**0%**	**0%**	**0%**	**0%**	**0%**	**0%**
**Conformer 5-2**	**40%**	**30%**	**30%**	**30%**	**30%**	**30%**	**30%**	**30%**
**Conformer 5-5**	**0%**	**70%**	**60%**	**50%**	**40%**	**30%**	**20%**	**10%**
**Conformer 5-9**	**60%**	**0%**	**10%**	**20%**	**30%**	**40%**	**50%**	**60%**
**σC12**	164.4841	164.3048	164.3257	164.3467	164.3677	164.3887	164.4097	164.4307
**σC11**	107.1083	108.5720	108.3601	108.1483	107.9365	107.7247	107.5128	107.3010
**σC14**	111.6133	109.1572	109.4927	109.8281	110.1635	110.4990	110.8344	111.1698

**σC13**	163.6187	164.2792	164.1817	164.0843	163.9868	163.8894	163.7919	163.6945
**σC8**	167.1977	166.3114	166.4445	166.5776	166.7107	166.8438	166.9769	167.1100
**σC7**	109.1915	110.6997	110.4904	110.2812	110.0720	109.8628	109.6535	109.4443
**σC10**	108.8816	109.6089	109.4876	109.3662	109.2448	109.1234	109.0021	108.8807
**σC9**	161.1928	162.2685	162.0996	161.9306	161.7616	161.5926	161.4236	161.2546
**σC16**	164.9053	162.2708	162.6290	162.9871	163.3453	163.7035	164.0616	164.4198
**σC15**	110.0848	110.1716	110.1827	110.1938	110.2049	110.2159	110.2270	110.2381
**σC18**	112.1811	111.0274	111.2096	111.3918	111.5741	111.7563	111.9385	112.1208
**σC17**	168.6609	164.9452	165.4388	165.9323	166.4258	166.9194	167.4129	167.9065
**σC6**	118.2419	119.9443	119.6853	119.4264	119.1674	118.9084	118.6495	118.3905
**σC19**	121.1601	122.1688	122.0363	121.9038	121.7712	121.6387	121.5061	121.3736
**σC28**	173.6252	172.2859	172.4648	172.6437	172.8227	173.0016	173.1805	173.3594
**σC27**	169.9264	171.2816	171.0761	170.8705	170.6650	170.4594	170.2539	170.0483
**σC20**	149.9144	151.9233	151.6015	151.2797	150.9579	150.6361	150.3144	149.9926
**σC21**	71.7954	72.3793	72.2354	72.0916	71.9478	71.8039	71.6601	71.5163
**σC22**	58.3338	59.0371	59.0081	58.9792	58.9502	58.9212	58.8923	58.8633
**σC23**	124.2485	123.4524	123.5195	123.5867	123.6538	123.7210	123.7881	123.8552
**σC24**	167.0679	167.8671	167.6755	167.4839	167.2924	167.1008	166.9092	166.7176
**σC30**	167.5709	165.9400	166.2157	166.4914	166.7671	167.0429	167.3186	167.5943
**σC29**	170.0745	168.1285	168.3307	168.5329	168.7352	168.9374	169.1396	169.3418
**σC4**	69.2414	70.0253	69.9764	69.9276	69.8788	69.8300	69.7812	69.7324
**σC5**	151.9872	153.1227	152.9366	152.7504	152.5642	152.3780	152.1919	152.0057
**σC2**	114.8032	113.9155	114.0415	114.1676	114.2937	114.4197	114.5458	114.6718
**σC3**	60.7152	60.2428	60.2748	60.3069	60.3390	60.3710	60.4031	60.4351
**σC26**	172.5394	171.5154	171.6511	171.7868	171.9225	172.0581	172.1938	172.3295
**σC1**	174.8739	172.3087	172.6823	173.0560	173.4297	173.8033	174.1770	174.5507
**σC25**	168.8642	167.9927	168.1155	168.2384	168.3613	168.4841	168.6070	168.7299
**σH12a**	29.7275	29.7423	29.7399	29.7375	29.7351	29.7326	29.7302	29.7278
**σH12b**	29.9941	30.0878	30.0742	30.0606	30.0470	30.0335	30.0199	30.0063
**σH11**	27.3245	27.4004	27.3890	27.3776	27.3662	27.3547	27.3433	27.3319
**σH14**	27.2591	27.3364	27.3261	27.3158	27.3054	27.2951	27.2847	27.2744
**σH13a**	29.5194	29.6744	29.6525	29.6307	29.6088	29.5870	29.5651	29.5433
**σH13b**	29.9231	30.0856	30.0619	30.0383	30.0147	29.9910	29.9674	29.9438
**σH8a**	29.8008	29.8949	29.8800	29.8652	29.8503	29.8354	29.8205	29.8056
**σH8b**	28.9898	29.2709	29.2281	29.1853	29.1425	29.0997	29.0569	29.0141
**σH7**	27.4321	27.7351	27.6898	27.6446	27.5993	27.5541	27.5088	27.4635
**σH9a**	29.4491	29.3725	29.3856	29.3987	29.4118	29.4249	29.4380	29.4511
**σH9b**	29.9837	30.0860	30.0722	30.0584	30.0447	30.0309	30.0171	30.0034
**σH16a**	30.1473	30.1504	30.1510	30.1515	30.1521	30.1526	30.1532	30.1538
**σH16b**	29.5277	29.5298	29.5283	29.5268	29.5253	29.5238	29.5223	29.5208
**σH18**	27.7661	27.7262	27.7331	27.7399	27.7468	27.7536	27.7605	27.7673
**σH17a**	29.4545	29.6031	29.5834	29.5637	29.5440	29.5243	29.5046	29.4849
**σH17b**	29.7556	29.9835	29.9477	29.9118	29.8759	29.8400	29.8041	29.7682
**σ$H_3$28**	30.6006	30.5538	30.5607	30.5675	30.5744	30.5813	30.5882	30.5950
**σ$H_3$27**	30.4984	30.5136	30.5111	30.5087	30.5062	30.5038	30.5013	30.4989
**σH20a**	29.1339	29.1436	29.1327	29.1218	29.1109	29.0999	29.0890	29.0781
**σH20b**	29.4973	29.5472	29.5397	29.5322	29.5247	29.5172	29.5097	29.5021
**σH21**	25.2410	24.8118	24.8698	24.9279	24.9859	25.0440	25.1020	25.1601
**σH22**	25.5548	25.6664	25.6507	25.6351	25.6195	25.6038	25.5882	25.5726
**σ$H_3$24**	30.4280	30.4492	30.4461	30.4429	30.4398	30.4367	30.4336	30.4305
**σ$H_3$30**	30.2639	30.2587	30.2636	30.2684	30.2732	30.2780	30.2828	30.2877

**σ$H_3$29**	30.1144	30.2776	30.2545	30.2314	30.2083	30.1852	30.1621	30.1390
**σH4**	25.2425	24.7673	24.8350	24.9027	24.9703	25.0380	25.1057	25.1733
**σH5a**	29.6241	29.7265	29.7123	29.6981	29.6840	29.6698	29.6557	29.6415
**σH5b**	29.4998	29.1307	29.1824	29.2340	29.2857	29.3373	29.3889	29.4406
**σH3**	25.6110	25.6891	25.6767	25.6642	25.6518	25.6394	25.6269	25.6145
**σ$H_3$26**	30.1782	30.3384	30.3130	30.2877	30.2623	30.2369	30.2115	30.1861
**σ$H_3$1**	30.1879	30.1904	30.1899	30.1893	30.1888	30.1882	30.1877	30.1871
**σ$H_3$25**	30.4495	30.4284	30.4331	30.4378	30.4425	30.4472	30.4519	30.4566
$^3J_{H4,H5}$	5.40	8.33	7.91	7.49	7.07	6.66	6.24	5.82
$^3J_{H4',H5}$	8.80	5.73	6.16	6.60	7.03	7.47	7.90	8.34
$^3J_{H20,H21}$	3.90	3.99	3.97	3.95	3.93	3.92	3.90	3.88
$^3J_{H20',H21}$	9.37	9.36	9.36	9.36	9.36	9.36	9.36	9.36

**Conformer 5-1**	**0%**	**0%**	**0%**	**0%**	**0%**	**0%**	**0%**	**0%**
**Conformer 5-2**	**30%**	**20%**	**20%**	**20%**	**20%**	**20%**	**20%**	**20%**
**Conformer 5-5**	**0%**	**80%**	**70%**	**60%**	**50%**	**40%**	**30%**	**20%**
**Conformer 5-9**	**70%**	**0%**	**10%**	**20%**	**30%**	**40%**	**50%**	**60%**
**σC12**	164.4516	164.2513	164.2723	164.2933	164.3143	164.3353	164.3562	164.3772
**σC11**	107.0892	108.7646	108.5528	108.3410	108.1291	107.9173	107.7055	107.4936
**σC14**	111.5053	108.7138	109.0492	109.3846	109.7201	110.0555	110.3909	110.7264
**σC13**	163.5970	164.3550	164.2575	164.1601	164.0626	163.9652	163.8677	163.7703
**σC8**	167.2432	166.2237	166.3568	166.4899	166.6230	166.7562	166.8893	167.0224
**σC7**	109.2351	110.9525	110.7433	110.5340	110.3248	110.1156	109.9063	109.6971
**σC10**	108.7593	109.6080	109.4866	109.3653	109.2439	109.1225	109.0012	108.8798
**σC9**	161.0857	162.3304	162.1614	161.9924	161.8234	161.6544	161.4855	161.3165
**σC16**	164.7780	161.7853	162.1435	162.5017	162.8598	163.2180	163.5762	163.9343
**σC15**	110.2492	110.3249	110.3360	110.3471	110.3582	110.3692	110.3803	110.3914
**σC18**	112.3030	110.9670	111.1492	111.3315	111.5137	111.6959	111.8781	112.0604
**σC17**	168.4000	164.1908	164.6844	165.1779	165.6714	166.1650	166.6585	167.1521
**σC6**	118.1316	120.0929	119.8340	119.5750	119.3161	119.0571	118.7982	118.5392
**σC19**	121.2411	122.3823	122.2498	122.1172	121.9847	121.8522	121.7196	121.5871
**σC28**	173.5384	172.0201	172.1990	172.3779	172.5568	172.7358	172.9147	173.0936
**σC27**	169.8428	171.4035	171.1980	170.9924	170.7869	170.5813	170.3758	170.1702
**σC20**	149.6708	152.0014	151.6797	151.3579	151.0361	150.7143	150.3925	150.0707
**σC21**	71.3724	72.1001	71.9563	71.8124	71.6686	71.5248	71.3810	71.2371
**σC22**	58.8344	59.5666	59.5377	59.5087	59.4797	59.4508	59.4218	59.3929
**σC23**	123.9224	123.0591	123.1262	123.1934	123.2605	123.3277	123.3948	123.4619
**σC24**	166.5260	167.5168	167.3252	167.1336	166.9421	166.7505	166.5589	166.3673
**σC30**	167.8700	165.9634	166.2391	166.5149	166.7906	167.0663	167.3420	167.6178
**σC29**	169.5440	167.3958	167.5980	167.8002	168.0024	168.2046	168.4069	168.6091
**σC4**	69.6836	70.5162	70.4674	70.4186	70.3698	70.3210	70.2721	70.2233
**σC5**	151.8195	153.1412	152.9551	152.7689	152.5827	152.3965	152.2104	152.0242
**σC2**	114.7979	113.7841	113.9102	114.0362	114.1623	114.2884	114.4144	114.5405
**σC3**	60.4672	59.9627	59.9948	60.0268	60.0589	60.0910	60.1230	60.1551
**σC26**	172.4652	171.3055	171.4412	171.5769	171.7126	171.8483	171.9839	172.1196
**σC1**	174.9244	171.9855	172.3592	172.7328	173.1065	173.4802	173.8539	174.2275
**σC25**	168.8527	167.8584	167.9812	168.1041	168.2269	168.3498	168.4727	168.5955
**σH12a**	29.7254	29.7427	29.7402	29.7378	29.7354	29.7330	29.7306	29.7282
**σH12b**	29.9927	30.1000	30.0864	30.0728	30.0592	30.0456	30.0321	30.0185
**σH11**	27.3205	27.4078	27.3963	27.3849	27.3735	27.3621	27.3506	27.3392

**σH14**	27.2641	27.3517	27.3414	27.3310	27.3207	27.3104	27.3000	27.2897
**σH13a**	29.5214	29.6982	29.6764	29.6545	29.6327	29.6108	29.5889	29.5671
**σH13b**	29.9201	30.1062	30.0826	30.0590	30.0353	30.0117	29.9881	29.9645
**σH8a**	29.7907	29.8997	29.8849	29.8700	29.8551	29.8402	29.8253	29.8104
**σH8b**	28.9713	29.2953	29.2525	29.2097	29.1669	29.1241	29.0813	29.0385
**σH7**	27.4183	27.7665	27.7213	27.6760	27.6307	27.5855	27.5402	27.4950
**σH9a**	29.4642	29.3745	29.3876	29.4007	29.4138	29.4269	29.4400	29.4531
**σH9b**	29.9896	30.1056	30.0919	30.0781	30.0644	30.0506	30.0368	30.0231
**σH16a**	30.1543	30.1568	30.1574	30.1579	30.1585	30.1591	30.1596	30.1602
**σH16b**	29.5193	29.5230	29.5215	29.5200	29.5185	29.5170	29.5155	29.5140
**σH18**	27.7742	27.7275	27.7343	27.7412	27.7480	27.7549	27.7617	27.7686
**σH17a**	29.4652	29.6335	29.6138	29.5941	29.5744	29.5547	29.5350	29.5153
**σH17b**	29.7323	29.9962	29.9603	29.9244	29.8885	29.8526	29.8167	29.7809
**σ$H_3$28**	30.6019	30.5483	30.5551	30.5620	30.5689	30.5758	30.5826	30.5895
**σ$H_3$27**	30.4964	30.5140	30.5116	30.5091	30.5067	30.5042	30.5018	30.4993
**σH20a**	29.0672	29.0878	29.0769	29.0660	29.0551	29.0442	29.0333	29.0224
**σH20b**	29.4946	29.5521	29.5445	29.5370	29.5295	29.5220	29.5145	29.5070
**σH21**	25.2182	24.7309	24.7889	24.8470	24.9050	24.9631	25.0211	25.0792
**σH22**	25.5569	25.6841	25.6685	25.6528	25.6372	25.6216	25.6059	25.5903
**σ$H_3$24**	30.4273	30.4517	30.4486	30.4454	30.4423	30.4392	30.4361	30.4330
**σ$H_3$30**	30.2925	30.2825	30.2873	30.2921	30.2969	30.3017	30.3066	30.3114
**σ$H_3$29**	30.1159	30.3022	30.2791	30.2560	30.2329	30.2098	30.1867	30.1636
**σH4**	25.2410	24.6981	24.7658	24.8335	24.9011	24.9688	25.0365	25.1042
**σH5a**	29.6273	29.7439	29.7297	29.7156	29.7014	29.6872	29.6731	29.6589
**σH5b**	29.4922	29.0715	29.1231	29.1748	29.2264	29.2781	29.3297	29.3813
**σH3**	25.6020	25.6926	25.6802	25.6677	25.6553	25.6429	25.6304	25.6180
**σ$H_3$26**	30.1607	30.3463	30.3210	30.2956	30.2702	30.2448	30.2194	30.1940
**σ$H_3$1**	30.1865	30.1896	30.1891	30.1885	30.1879	30.1874	30.1868	30.1863
**σ$H_3$25**	30.4613	30.4355	30.4402	30.4449	30.4496	30.4543	30.4590	30.4637
**$^3J_{H4,H5}$**	5.40	8.75	8.33	7.91	7.49	7.07	6.66	6.24
**$^3J_{H4',H5}$**	8.77	5.26	5.70	6.13	6.57	7.01	7.44	7.88
**$^3J_{H20,H21}$**	3.86	3.97	3.95	3.93	3.92	3.90	3.88	3.86
**$^3J_{H20',H21}$**	9.36	9.35	9.35	9.35	9.35	9.35	9.35	9.35

**Conformer 5-1**	**0%**	**0%**	**0%**	**0%**	**0%**	**0%**	**0%**	**0%**
**Conformer 5-2**	**20%**	**20%**	**10%**	**10%**	**10%**	**10%**	**10%**	**10%**
**Conformer 5-5**	**10%**	**0%**	**90%**	**80%**	**70%**	**60%**	**50%**	**40%**
**Conformer 5-9**	**70%**	**80%**	**0%**	**10%**	**20%**	**30%**	**40%**	**50%**
**σC12**	164.3982	164.4192	164.1979	164.2189	164.2399	164.2609	164.2818	164.3028
**σC11**	107.2818	107.0700	108.9573	108.7454	108.5336	108.3218	108.1099	107.8981
**σC14**	111.0618	111.3972	108.2703	108.6057	108.9412	109.2766	109.6120	109.9475
**σC13**	163.6728	163.5754	164.4308	164.3333	164.2359	164.1384	164.0410	163.9435
**σC8**	167.1555	167.2886	166.1361	166.2692	166.4023	166.5354	166.6685	166.8016
**σC7**	109.4879	109.2786	111.2053	110.9961	110.7868	110.5776	110.3684	110.1591
**σC10**	108.7584	108.6371	109.6071	109.4857	109.3644	109.2430	109.1216	109.0003
**σC9**	161.1475	160.9785	162.3922	162.2232	162.0542	161.8852	161.7163	161.5473
**σC16**	164.2925	164.6507	161.2998	161.6580	162.0162	162.3744	162.7325	163.0907
**σC15**	110.4025	110.4135	110.4782	110.4893	110.5004	110.5115	110.5225	110.5336

**σC18**	112.2426	112.4248	110.9066	111.0888	111.2711	111.4533	111.6355	111.8177
**σC17**	167.6456	168.1391	163.4364	163.9299	164.4235	164.9170	165.4106	165.9041
**σC6**	118.2802	118.0213	120.2416	119.9826	119.7237	119.4647	119.2058	118.9468
**σC19**	121.4545	121.3220	122.5958	122.4633	122.3307	122.1982	122.0657	121.9331
**σC28**	173.2725	173.4515	171.7542	171.9332	172.1121	172.2910	172.4700	172.6489
**σC27**	169.9647	169.7591	171.5254	171.3198	171.1143	170.9087	170.7032	170.4977
**σC20**	149.7489	149.4271	152.0796	151.7578	151.4360	151.1142	150.7924	150.4706
**σC21**	71.0933	70.9495	71.8209	71.6771	71.5333	71.3895	71.2456	71.1018
**σC22**	59.3639	59.3349	60.0962	60.0672	60.0382	60.0093	59.9803	59.9514
**σC23**	123.5291	123.5962	122.6658	122.7329	122.8001	122.8672	122.9344	123.0015
**σC24**	166.1757	165.9842	167.1665	166.9749	166.7833	166.5918	166.4002	166.2086
**σC30**	167.8935	168.1692	165.9869	166.2626	166.5383	166.8140	167.0898	167.3655
**σC29**	168.8113	169.0135	166.6631	166.8653	167.0675	167.2697	167.4719	167.6741
**σC4**	70.1745	70.1257	71.0072	70.9584	70.9095	70.8607	70.8119	70.7631
**σC5**	151.8380	151.6518	153.1597	152.9736	152.7874	152.6012	152.4150	152.2289
**σC2**	114.6665	114.7926	113.6527	113.7788	113.9049	114.0309	114.1570	114.2831
**σC3**	60.1871	60.2192	59.6826	59.7147	59.7468	59.7788	59.8109	59.8430
**σC26**	172.2553	172.3910	171.0956	171.2313	171.3670	171.5027	171.6384	171.7741
**σC1**	174.6012	174.9749	171.6623	172.0360	172.4097	172.7834	173.1570	173.5307
**σC25**	168.7184	168.8413	167.7240	167.8469	167.9698	168.0926	168.2155	168.3384
**σH12a**	29.7258	29.7233	29.7430	29.7406	29.7382	29.7358	29.7333	29.7309
**σH12b**	30.0049	29.9913	30.1122	30.0986	30.0850	30.0714	30.0578	30.0442
**σH11**	27.3278	27.3164	27.4151	27.4037	27.3922	27.3808	27.3694	27.3580
**σH14**	27.2793	27.2690	27.3670	27.3567	27.3463	27.3360	27.3256	27.3153
**σH13a**	29.5452	29.5234	29.7220	29.7002	29.6783	29.6565	29.6346	29.6128
**σH13b**	29.9408	29.9172	30.1269	30.1033	30.0797	30.0560	30.0324	30.0088
**σH8a**	29.7955	29.7807	29.9045	29.8897	29.8748	29.8599	29.8450	29.8301
**σH8b**	28.9957	28.9529	29.3196	29.2768	29.2341	29.1913	29.1485	29.1057
**σH7**	27.4497	27.4045	27.7979	27.7527	27.7074	27.6622	27.6169	27.5717
**σH9a**	29.4662	29.4792	29.3765	29.3896	29.4027	29.4158	29.4289	29.4420
**σH9b**	30.0093	29.9955	30.1253	30.1116	30.0978	30.0840	30.0703	30.0565
**σH16a**	30.1607	30.1613	30.1632	30.1638	30.1643	30.1649	30.1655	30.1660
**σH16b**	29.5125	29.5110	29.5161	29.5146	29.5131	29.5116	29.5101	29.5086
**σH18**	27.7754	27.7822	27.7287	27.7355	27.7424	27.7492	27.7561	27.7629
**σH17a**	29.4957	29.4760	29.6639	29.6442	29.6245	29.6048	29.5851	29.5654
**σH17b**	29.7450	29.7091	30.0088	29.9730	29.9371	29.9012	29.8653	29.8294
**σ$H_3$28**	30.5964	30.6033	30.5427	30.5496	30.5565	30.5634	30.5702	30.5771
**σ$H_3$27**	30.4969	30.4944	30.5145	30.5120	30.5096	30.5071	30.5047	30.5022
**σH20a**	29.0115	29.0005	29.0321	29.0211	29.0102	28.9993	28.9884	28.9775
**σH20b**	29.4995	29.4920	29.5569	29.5494	29.5419	29.5343	29.5268	29.5193
**σH21**	25.1372	25.1953	24.6500	24.7080	24.7661	24.8241	24.8822	24.9402
**σH22**	25.5747	25.5590	25.7018	25.6862	25.6705	25.6549	25.6393	25.6236
**σ$H_3$24**	30.4298	30.4267	30.4542	30.4511	30.4479	30.4448	30.4417	30.4386
**σ$H_3$30**	30.3162	30.3210	30.3062	30.3110	30.3158	30.3206	30.3255	30.3303
**σ$H_3$29**	30.1405	30.1174	30.3268	30.3037	30.2806	30.2575	30.2344	30.2113
**σH4**	25.1718	25.2395	24.6290	24.6966	24.7643	24.8320	24.8996	24.9673
**σH5a**	29.6447	29.6306	29.7613	29.7471	29.7330	29.7188	29.7047	29.6905
**σH5b**	29.4330	29.4846	29.0122	29.0639	29.1155	29.1672	29.2188	29.2704
**σH3**	25.6055	25.5931	25.6961	25.6837	25.6712	25.6588	25.6464	25.6339
**σ$H_3$26**	30.1686	30.1433	30.3543	30.3289	30.3035	30.2781	30.2527	30.2273

**$\sigma H_3 1$**	30.1857	30.1852	30.1888	30.1882	30.1877	30.1871	30.1866	30.1860
**$\sigma H_3 25$**	30.4684	30.4730	30.4426	30.4473	30.4520	30.4567	30.4614	30.4660
**$^3J_{H4,H5}$**	5.82	5.40	9.17	8.75	8.33	7.91	7.49	7.07
**$^3J_{H4',H5}$**	8.31	8.75	4.80	5.24	5.67	6.11	6.54	6.98
**$^3J_{H20,H21}$**	3.84	3.82	3.95	3.93	3.92	3.90	3.88	3.86
**$^3J_{H20',H21}$**	9.35	9.35	9.34	9.34	9.34	9.34	9.34	9.34

**Conformer 5-1**	**0%**	**0%**	**0%**	**0%**	**0%**	**0%**	**0%**	**0%**
**Conformer 5-2**	**10%**	**10%**	**10%**	**10%**	**0%**	**0%**	**0%**	**0%**
**Conformer 5-5**	**30%**	**20%**	**10%**	**0%**	**100%**	**90%**	**80%**	**70%**
**Conformer 5-9**	**60%**	**70%**	**80%**	**90%**	**0%**	**10%**	**20%**	**30%**
**σC12**	164.3238	164.3448	164.3658	164.3867	164.1445	164.1655	164.1865	164.2074
**σC11**	107.6863	107.4744	107.2626	107.0508	109.1499	108.9381	108.7262	108.5144
**σC14**	110.2829	110.6184	110.9538	111.2892	107.8268	108.1623	108.4977	108.8331
**σC13**	163.8461	163.7486	163.6512	163.5537	164.5066	164.4091	164.3117	164.2142
**σC8**	166.9347	167.0678	167.2009	167.3341	166.0484	166.1815	166.3146	166.4477
**σC7**	109.9499	109.7407	109.5315	109.3222	111.4581	111.2489	111.0396	110.8304
**σC10**	108.8789	108.7575	108.6361	108.5148	109.6062	109.4848	109.3635	109.2421
**σC9**	161.3783	161.2093	161.0403	160.8714	162.4540	162.2850	162.1160	161.9471
**σC16**	163.4489	163.8071	164.1652	164.5234	160.8144	161.1726	161.5307	161.8889
**σC15**	110.5447	110.5558	110.5668	110.5779	110.6315	110.6426	110.6537	110.6648
**σC18**	112.0000	112.1822	112.3644	112.5467	110.8462	111.0284	111.2107	111.3929
**σC17**	166.3976	166.8912	167.3847	167.8783	162.6820	163.1755	163.6691	164.1626
**σC6**	118.6879	118.4289	118.1699	117.9110	120.3902	120.1313	119.8723	119.6134
**σC19**	121.8006	121.6680	121.5355	121.4030	122.8093	122.6768	122.5442	122.4117
**σC28**	172.8278	173.0067	173.1857	173.3646	171.4884	171.6674	171.8463	172.0252
**σC27**	170.2921	170.0866	169.8810	169.6755	171.6473	171.4417	171.2362	171.0306
**σC20**	150.1488	149.8270	149.5052	149.1835	152.1577	151.8359	151.5141	151.1923
**σC21**	70.9580	70.8141	70.6703	70.5265	71.5418	71.3980	71.2541	71.1103
**σC22**	59.9224	59.8934	59.8645	59.8355	60.6257	60.5967	60.5678	60.5388
**σC23**	123.0686	123.1358	123.2029	123.2701	122.2725	122.3396	122.4068	122.4739
**σC24**	166.0170	165.8254	165.6339	165.4423	166.8162	166.6246	166.4330	166.2415
**σC30**	167.6412	167.9170	168.1927	168.4684	166.0103	166.2861	166.5618	166.8375
**σC29**	167.8764	168.0786	168.2808	168.4830	165.9304	166.1326	166.3348	166.5370
**σC4**	70.7143	70.6655	70.6167	70.5679	71.4981	71.4493	71.4005	71.3517
**σC5**	152.0427	151.8565	151.6703	151.4842	153.1783	152.9921	152.8059	152.6197
**σC2**	114.4091	114.5352	114.6612	114.7873	113.5214	113.6474	113.7735	113.8996
**σC3**	59.8750	59.9071	59.9391	59.9712	59.4026	59.4346	59.4667	59.4988
**σC26**	171.9097	172.0454	172.1811	172.3168	170.8858	171.0214	171.1571	171.2928
**σC1**	173.9044	174.2780	174.6517	175.0254	171.3392	171.7128	172.0865	172.4602
**σC25**	168.4612	168.5841	168.7070	168.8298	167.5897	167.7126	167.8355	167.9583
**σH12a**	29.7285	29.7261	29.7237	29.7213	29.7434	29.7409	29.7385	29.7361
**σH12b**	30.0307	30.0171	30.0035	29.9899	30.1244	30.1108	30.0972	30.0836
**σH11**	27.3466	27.3351	27.3237	27.3123	27.4224	27.4110	27.3996	27.3882
**σH14**	27.3050	27.2946	27.2843	27.2740	27.3823	27.3719	27.3616	27.3513
**σH13a**	29.5909	29.5690	29.5472	29.5253	29.7459	29.7240	29.7021	29.6803
**σH13b**	29.9851	29.9615	29.9379	29.9142	30.1476	30.1240	30.1003	30.0767
**σH8a**	29.8152	29.8003	29.7855	29.7706	29.9094	29.8945	29.8796	29.8647
**σH8b**	29.0629	29.0201	28.9773	28.9345	29.3440	29.3012	29.2584	29.2156
**σH7**	27.5264	27.4811	27.4359	27.3906	27.8294	27.7841	27.7389	27.6936
**σH9a**	29.4551	29.4681	29.4812	29.4943	29.3785	29.3916	29.4047	29.4178

**σH9b**	30.0428	30.0290	30.0152	30.0015	30.1450	30.1313	30.1175	30.1037
**σH16a**	30.1666	30.1671	30.1677	30.1682	30.1696	30.1702	30.1708	30.1713
**σH16b**	29.5071	29.5056	29.5041	29.5026	29.5093	29.5078	29.5063	29.5048
**σH18**	27.7698	27.7766	27.7835	27.7903	27.7299	27.7368	27.7436	27.7505
**σH17a**	29.5458	29.5261	29.5064	29.4867	29.6943	29.6746	29.6549	29.6352
**σH17b**	29.7935	29.7576	29.7217	29.6858	30.0215	29.9856	29.9497	29.9138
**σ$H_3$28**	30.5840	30.5908	30.5977	30.6046	30.5372	30.5441	30.5510	30.5578
**σ$H_3$27**	30.4998	30.4973	30.4949	30.4924	30.5149	30.5125	30.5100	30.5076
**σH20a**	28.9666	28.9557	28.9448	28.9339	28.9763	28.9654	28.9545	28.9436
**σH20b**	29.5118	29.5043	29.4968	29.4893	29.5617	29.5542	29.5467	29.5392
**σH21**	24.9983	25.0563	25.1144	25.1725	24.5691	24.6271	24.6852	24.7432
**σH22**	25.6080	25.5924	25.5767	25.5611	25.7195	25.7039	25.6883	25.6726
**σ$H_3$24**	30.4355	30.4323	30.4292	30.4261	30.4567	30.4536	30.4504	30.4473
**σ$H_3$30**	30.3351	30.3399	30.3447	30.3496	30.3299	30.3347	30.3395	30.3444
**σ$H_3$29**	30.1882	30.1651	30.1420	30.1190	30.3514	30.3283	30.3052	30.2821
**σH4**	25.0350	25.1027	25.1703	25.2380	24.5598	24.6275	24.6951	24.7628
**σH5a**	29.6763	29.6622	29.6480	29.6338	29.7787	29.7646	29.7504	29.7362
**σH5b**	29.3221	29.3737	29.4254	29.4770	28.9530	29.0046	29.0563	29.1079
**σH3**	25.6215	25.6090	25.5966	25.5841	25.6996	25.6872	25.6747	25.6623
**σ$H_3$26**	30.2019	30.1765	30.1512	30.1258	30.3622	30.3368	30.3114	30.2860
**σ$H_3$1**	30.1855	30.1849	30.1843	30.1838	30.1880	30.1874	30.1869	30.1863
**σ$H_3$25**	30.4707	30.4754	30.4801	30.4848	30.4497	30.4544	30.4591	30.4637
$^3J_{H4,H5}$	6.66	6.24	5.82	5.40	9.59	9.17	8.75	8.33
$^3J_{H4',H5}$	7.41	7.85	8.28	8.72	4.34	4.77	5.21	5.64
$^3J_{H20,H21}$	3.84	3.82	3.81	3.79	3.93	3.92	3.90	3.88
$^3J_{H20',H21}$	9.34	9.34	9.34	9.34	9.33	9.33	9.33	9.33

**Conformer 5-1**	**0%**	**0%**	**0%**	**0%**	**0%**	**0%**	**0%**
**Conformer 5-2**	**0%**	**0%**	**0%**	**0%**	**0%**	**0%**	**0%**
**Conformer 5-5**	**60%**	**50%**	**40%**	**30%**	**20%**	**10%**	**0%**
**Conformer 5-9**	**40%**	**50%**	**60%**	**70%**	**80%**	**90%**	**100%**
**σC12**	164.2284	164.2494	164.2704	164.2914	164.3123	164.3333	164.3543
**σC11**	108.3026	108.0908	107.8789	107.6671	107.4553	107.2434	107.0316
**σC14**	109.1686	109.5040	109.8395	110.1749	110.5103	110.8458	111.1812
**σC13**	164.1168	164.0193	163.9219	163.8244	163.7270	163.6295	163.5321
**σC8**	166.5808	166.7139	166.8471	166.9802	167.1133	167.2464	167.3795
**σC7**	110.6212	110.4120	110.2027	109.9935	109.7843	109.5750	109.3658
**σC10**	109.1207	108.9994	108.8780	108.7566	108.6352	108.5139	108.3925
**σC9**	161.7781	161.6091	161.4401	161.2711	161.1022	160.9332	160.7642
**σC16**	162.2471	162.6052	162.9634	163.3216	163.6798	164.0379	164.3961
**σC15**	110.6758	110.6869	110.6980	110.7091	110.7201	110.7312	110.7423
**σC18**	111.5751	111.7574	111.9396	112.1218	112.3040	112.4863	112.6685
**σC17**	164.6562	165.1497	165.6432	166.1368	166.6303	167.1239	167.6174
**σC6**	119.3544	119.0955	118.8365	118.5776	118.3186	118.0597	117.8007
**σC19**	122.2791	122.1466	122.0141	121.8815	121.7490	121.6164	121.4839
**σC28**	172.2041	172.3831	172.5620	172.7409	172.9198	173.0988	173.2777
**σC27**	170.8251	170.6195	170.4140	170.2084	170.0029	169.7973	169.5918
**σC20**	150.8705	150.5488	150.2270	149.9052	149.5834	149.2616	148.9398

**σC21**	70.9665	70.8226	70.6788	70.5350	70.3912	70.2473	70.1035
**σC22**	60.5099	60.4809	60.4519	60.4230	60.3940	60.3651	60.3361
**σC23**	122.5411	122.6082	122.6753	122.7425	122.8096	122.8768	122.9439
**σC24**	166.0499	165.8583	165.6667	165.4751	165.2836	165.0920	164.9004
**σC30**	167.1132	167.3890	167.6647	167.9404	168.2161	168.4919	168.7676
**σC29**	166.7392	166.9414	167.1436	167.3459	167.5481	167.7503	167.9525
**σC4**	71.3029	71.2541	71.2052	71.1564	71.1076	71.0588	71.0100
**σC5**	152.4336	152.2474	152.0612	151.8750	151.6889	151.5027	151.3165
**σC2**	114.0256	114.1517	114.2777	114.4038	114.5299	114.6559	114.7820
**σC3**	59.5308	59.5629	59.5949	59.6270	59.6591	59.6911	59.7232
**σC26**	171.4285	171.5642	171.6999	171.8355	171.9712	172.1069	172.2426
**σC1**	172.8339	173.2075	173.5812	173.9549	174.3286	174.7022	175.0759
**σC25**	168.0812	168.2041	168.3269	168.4498	168.5727	168.6955	168.8184
**σH12a**	29.7337	29.7313	29.7289	29.7264	29.7240	29.7216	29.7192
**σH12b**	30.0700	30.0564	30.0428	30.0293	30.0157	30.0021	29.9885
**σH11**	27.3767	27.3653	27.3539	27.3425	27.3310	27.3196	27.3082
**σH14**	27.3409	27.3306	27.3203	27.3099	27.2996	27.2892	27.2789
**σH13a**	29.6584	29.6366	29.6147	29.5929	29.5710	29.5492	29.5273
**σH13b**	30.0531	30.0295	30.0058	29.9822	29.9586	29.9349	29.9113
**σH8a**	29.8498	29.8349	29.8200	29.8052	29.7903	29.7754	29.7605
**σH8b**	29.1728	29.1301	29.0873	29.0445	29.0017	28.9589	28.9161
**σH7**	27.6483	27.6031	27.5578	27.5126	27.4673	27.4221	27.3768
**σH9a**	29.4309	29.4440	29.4570	29.4701	29.4832	29.4963	29.5094
**σH9b**	30.0900	30.0762	30.0624	30.0487	30.0349	30.0212	30.0074
**σH16a**	30.1719	30.1724	30.1730	30.1735	30.1741	30.1746	30.1752
**σH16b**	29.5033	29.5018	29.5003	29.4988	29.4973	29.4958	29.4943
**σH18**	27.7573	27.7642	27.7710	27.7779	27.7847	27.7916	27.7984
**σH17a**	29.6155	29.5959	29.5762	29.5565	29.5368	29.5171	29.4974
**σH17b**	29.8779	29.8420	29.8062	29.7703	29.7344	29.6985	29.6626
**σ$H_3$28**	30.5647	30.5716	30.5784	30.5853	30.5922	30.5991	30.6059
**σ$H_3$27**	30.5051	30.5027	30.5002	30.4978	30.4953	30.4929	30.4904
**σH20a**	28.9327	28.9217	28.9108	28.8999	28.8890	28.8781	28.8672
**σH20b**	29.5317	29.5242	29.5166	29.5091	29.5016	29.4941	29.4866
**σH21**	24.8013	24.8593	24.9174	24.9754	25.0335	25.0915	25.1496
**σH22**	25.6570	25.6414	25.6257	25.6101	25.5945	25.5788	25.5632
**σ$H_3$24**	30.4442	30.4411	30.4380	30.4348	30.4317	30.4286	30.4255
**σ$H_3$30**	30.3492	30.3540	30.3588	30.3636	30.3685	30.3733	30.3781
**σ$H_3$29**	30.2590	30.2359	30.2128	30.1898	30.1667	30.1436	30.1205
**σH4**	24.8305	24.8981	24.9658	25.0335	25.1012	25.1688	25.2365
**σH5a**	29.7221	29.7079	29.6938	29.6796	29.6654	29.6513	29.6371
**σH5b**	29.1596	29.2112	29.2628	29.3145	29.3661	29.4178	29.4694
**σH3**	25.6499	25.6374	25.6250	25.6125	25.6001	25.5876	25.5752
**σ$H_3$26**	30.2606	30.2352	30.2098	30.1845	30.1591	30.1337	30.1083
**σ$H_3$1**	30.1857	30.1852	30.1846	30.1841	30.1835	30.1830	30.1824
**σ$H_3$25**	30.4684	30.4731	30.4778	30.4825	30.4872	30.4919	30.4966
$^3J_{H4,H5}$	7.91	7.49	7.07	6.66	6.24	5.82	5.40
$^3J_{H4',H5}$	6.08	6.51	6.95	7.39	7.82	8.26	8.69
$^3J_{H20,H21}$	3.86	3.84	3.82	3.81	3.79	3.77	3.75
$^3J_{H20',H21}$	9.33	9.33	9.33	9.33	9.33	9.33	9.33

5. CMAE, Max Error and DP4+ values for compounds 1-5.

Table S18. Correlation between computed (9 conformers) and experimental data of peloruside (**1**). All calculations were done at the mPW1PW91/6-31+G** level. *Dihedral angle of the conformation in $CDCl_3$ *versus* dihedral angle of the nine conformers of peloruside (**1**).

Conformer	1-1	1-2	1-3	1-4	1-5	1-6	1-7	1-8	1-9
^{13}C CMAE	2.31	1.76	2.07	2.67	1.51	2.35	2.65	2.53	1.82
^{13}C Max Error	11.48	7.07	8.17	7.82	6.56	8.27	6.81	7.81	6.80
^{1}H CMAE	0.34	0.15	0.34	0.35	0.17	0.32	0.26	0.40	0.15
^{1}H Max Error	1.61	0.39	1.43	1.86	0.76	1.03	1.67	2.00	0.34
H (DP4+)	0.00%	55.22%	0.00%	0.00%	0.03%	0.00%	0.00%	0.00%	44.75%
C (DP4+)	0.00%	0.01%	0.00%	0.00%	99.99%	0.00%	0.00%	0.00%	0.00%
DP4+	0.00%	15.56%	0.00%	0.00%	84.44%	0.00%	0.00%	0.00%	0.00%
Dihedral angle (RMSD)*	34.41	28.24	37.67	33.91	23.83	35.48	41.73	34.63	27.17
Boltzmann population	0.14%	58.23%	0.00%	0.00%	40.57%	0.00%	0.61%	0.00%	0.44%

Table S19. Correlation between computed (48 conformers) and experimental data of okadaic acid (**2**). All calculations were done at the mPW1PW91/6-31+G** level.

Conformer	2-1	2-2	2-3	2-4	2-5	2-6	2-7	2-8	2-9	2-10
^{13}C CMAE	1.84	1.70	1.82	2.03	2.39	2.10	1.95	2.26	2.03	3.01
^{13}C Max Error	7.78	8.57	7.13	8.23	12.91	13.50	8.00	8.86	8.23	15.87
^{1}H CMAE	0.18	0.19	0.24	0.25	0.26	0.24	0.23	0.24	0.25	0.31
^{1}H Max Error	0.70	0.91	0.74	1.44	1.73	0.90	0.68	1.32	1.44	0.84
H (DP4+)	100.00%	0.00%	0.00%	0.00%	0.00%	0.00%	0.00%	0.00%	0.00%	0.00%
C (DP4+)	0.27%	96.67%	0.00%	0.00%	0.00%	0.00%	0.00%	0.00%	0.00%	0.00%
DP4+	99.97%	0.03%	0.00%	0.00%	0.00%	0.00%	0.00%	0.00%	0.00%	0.00%
Boltzmann population	88.09%	0.00%	2.07%	0.99%	0.11%	0.02%	0.17%	0.19%	0.99%	0.00%

Conformer	2-11	2-12	2-13	2-14	2-15	2-16	2-17	2-18	2-19	2-20
^{13}C CMAE	2.64	2.19	2.38	2.64	2.73	2.03	2.18	1.93	2.81	2.49
^{13}C Max Error	15.11	8.06	12.13	15.11	13.56	8.13	8.84	8.32	15.05	14.68
^{1}H CMAE	0.28	0.23	0.21	0.28	0.24	0.22	0.28	0.26	0.24	0.26
^{1}H Max Error	1.71	0.79	0.70	1.71	0.76	0.76	1.24	0.78	0.82	0.97
H (DP4+)	0.00%	0.00%	0.00%	0.00%	0.00%	0.00%	0.00%	0.00%	0.00%	0.00%
C (DP4+)	0.00%	0.00%	0.00%	0.00%	0.00%	0.00%	0.00%	0.00%	0.00%	0.00%
DP4+	0.00%	0.00%	0.00%	0.00%	0.00%	0.00%	0.00%	0.00%	0.00%	0.00%
Boltzmann population	0.08%	3.32%	0.62%	0.08%	0.00%	0.13%	0.02%	0.01%	0.40%	0.00%

Conformer	2-21	2-22	2-23	2-24	2-25	2-26	2-27	2-28	2-29	2-30
^{13}C CMAE	1.99	1.91	2.31	2.49	2.52	2.50	3.08	1.94	2.51	1.95
^{13}C Max Error	7.32	7.37	8.60	15.90	13.36	12.85	16.21	7.54	13.45	7.00
^{1}H CMAE	0.24	0.24	0.26	0.30	0.28	0.24	0.27	0.23	0.26	0.21
^{1}H Max Error	0.87	0.70	0.78	0.91	0.88	0.89	0.94	0.93	0.81	0.92
H (DP4+)	0.00%	0.00%	0.00%	0.00%	0.00%	0.00%	0.00%	0.00%	0.00%	0.00%
C (DP4+)	0.00%	0.00%	0.00%	0.00%	0.00%	0.00%	0.00%	0.02%	0.00%	0.11%
DP4+	0.00%	0.00%	0.00%	0.00%	0.00%	0.00%	0.00%	0.00%	0.00%	0.00%
Boltzmann population	1.29%	0.01%	0.22%	0.04%	0.00%	0.00%	0.00%	0.35%	0.00%	0.04%

Conformer	2-31	2-32	2-33	2-34	2-35	2-36	2-37	2-38	2-39	2-40
^{13}C CMAE	2.24	1.87	2.42	1.93	2.62	2.77	2.10	2.41	2.97	2.26
^{13}C Max Error	7.74	7.28	12.23	9.45	14.64	14.60	9.59	13.33	14.93	8.86
^{1}H CMAE	0.27	0.24	0.24	0.24	0.28	0.25	0.24	0.24	0.27	0.24
^{1}H Max Error	0.77	0.83	0.87	0.76	0.75	0.86	0.83	0.89	1.31	1.32
H (DP4+)	0.00%	0.00%	0.00%	0.00%	0.00%	0.00%	0.00%	0.00%	0.00%	0.00%
C (DP4+)	0.00%	2.16%	0.00%	0.01%	0.00%	0.00%	0.00%	0.00%	0.00%	0.00%
DP4+	0.00%	0.00%	0.00%	0.00%	0.00%	0.00%	0.00%	0.00%	0.00%	0.00%
Boltzmann population	0.02%	0.22%	0.00%	0.01%	0.00%	0.01%	0.13%	0.00%	0.00%	0.19%

Conformer	2-41	2-42	2-43	2-44	2-45	2-46	2-47	2-48
^{13}C CMAE	2.71	2.52	3.11	2.67	2.75	2.07	1.80	1.90
^{13}C Max Error	14.99	15.34	15.56	12.95	15.90	7.34	6.34	8.22
^{1}H CMAE	0.29	0.28	0.28	0.26	0.28	0.27	0.26	0.26
^{1}H Max Error	1.63	0.75	0.78	1.23	0.88	1.27	1.37	1.36
H (DP4+)	0.00%	0.00%	0.00%	0.00%	0.00%	0.00%	0.00%	0.00%
C (DP4+)	0.00%	0.00%	0.00%	0.00%	0.00%	0.00%	0.53%	0.23%
DP4+	0.00%	0.00%	0.00%	0.00%	0.00%	0.00%	0.00%	0.00%
Boltzmann population	0.07%	0.00%	0.00%	0.00%	0.00%	0.01%	0.02%	0.06%

Table S20. Correlation between computed (16 conformers) and experimental data of the *exo*-conformation of euphodendroid K (**3**). All calculations were done at the mPW1PW91/6-31+G** level.

Conformer	3-1	3-2	3-3	3-4	3-5	3-6	3-7	3-8	3-9	3-10
^{13}C CMAE	2.07	2.19	1.89	2.39	1.88	2.43	1.59	2.03	2.50	2.08
^{13}C Max Error	7.37	8.26	7.08	9.54	8.11	10.39	6.37	6.73	9.84	7.51
^{1}H CMAE	0.31	0.31	0.20	0.28	0.24	0.28	0.24	0.19	0.28	0.31
^{1}H Max Error	0.85	0.78	0.86	0.79	0.87	0.80	0.83	0.60	0.97	0.89
H (DP4+)	0.00%	0.00%	0.00%	0.00%	0.00%	0.00%	0.00%	99.99%	0.00%	0.00%
C (DP4+)	0.00%	0.00%	0.00%	0.00%	0.00%	0.00%	100.00%	0.00%	0.00%	0.00%

**DP4+**	0.00%	0.00%	0.00%	0.00%	0.00%	0.00%	97.22%	0.03%	0.00%	0.00%
**Boltzmann population**	2.41%	0.69%	19.32%	21.98%	1.54%	2.94%	1.78%	1.61%	1.38%	3.10%

**Conformer**	**3-11**	**3-12**	**3-13**	**3-14**	**3-15**	**3-16**
**^{13}C CMAE**	2.21	1.82	2.41	2.35	2.14	2.52
**^{13}C Max Error**	8.54	5.63	9.45	9.89	4.97	9.77
**^{1}H CMAE**	0.31	0.19	0.28	0.30	0.29	0.29
**^{1}H Max Error**	0.80	0.82	0.83	0.81	0.92	0.97
**H (DP4+)**	0.00%	0.01%	0.00%	0.00%	0.00%	0.00%
**C (DP4+)**	0.00%	0.00%	0.00%	0.00%	0.00%	0.00%
**DP4+**	0.00%	2.75%	0.00%	0.00%	0.00%	0.00%
**Boltzmann population**	0.38%	19.25%	18.88%	2.30%	1.00%	1.42%

Table S21. Correlation between computed (16 conformers) and experimental data of the *endo*-conformation of euphodendroid K (**3**). All calculations were done at the mPW1PW91/6-31+G** level.

**Conformer**	**3-1**	**3-2**	**3-3**	**3-4**	**3-5**	**3-6**	**3-7**	**3-8**	**3-9**	**3-10**
**^{13}C CMAE**	1.95	2.01	2.40	1.90	2.39	1.89	2.07	2.58	1.85	1.93
**^{13}C Max Error**	5.23	5.35	8.30	6.02	7.57	5.85	7.14	7.71	5.96	5.41
**^{1}H CMAE**	0.23	0.24	0.33	0.19	0.39	0.18	0.37	0.35	0.20	0.24
**^{1}H Max Error**	0.76	0.61	1.09	0.65	1.35	0.54	1.26	1.29	0.65	0.75
**H (DP4+)**	0.00%	0.00%	0.00%	24.72%	0.00%	65.31%	0.00%	0.00%	0.01%	0.00%
**C (DP4+)**	0.01%	0.00%	0.00%	58.88%	0.00%	32.67%	0.00%	0.00%	1.22%	0.02%
**DP4+**	0.00%	0.00%	0.00%	39.85%	0.00%	58.43%	0.00%	0.00%	0.00%	0.00%
**Boltzmann population**	2.41%	0.69%	19.32%	21.98%	1.54%	2.94%	1.78%	1.61%	1.38%	3.10%

**Conformer**	**3-11**	**3-12**	**3-13**	**3-14**	**3-15**	**3-16**
**^{13}C CMAE**	2.03	2.33	1.94	1.96	2.84	1.87
**^{13}C Max Error**	5.09	8.72	6.19	6.13	12.55	5.94
**^{1}H CMAE**	0.24	0.32	0.19	0.18	0.41	0.21
**^{1}H Max Error**	0.61	1.05	0.68	0.62	1.49	0.66
**H (DP4+)**	0.00%	0.00%	9.11%	0.86%	0.00%	0.00%
**C (DP4+)**	0.00%	0.00%	6.91%	0.11%	0.00%	0.18%
**DP4+**	0.00%	0.00%	1.72%	0.00%	0.00%	0.00%
**Boltzmann population**	0.38%	19.25%	18.88%	2.30%	1.00%	1.42%

Table S22. Correlation between computed (14 conformers) and experimental data of the *exo*-conformation of euphodendroid L (**4**). All calculations were done at the mPW1PW91/6-31+G** level.

**Conformer**	**4-1**	**4-2**	**4-3**	**4-4**	**4-5**	**4-6**	**4-7**	**4-8**	**4-9**	**4-10**
**^{13}C CMAE**	2.20	1.74	2.12	1.71	2.35	2.45	1.78	1.75	1.97	1.99
**^{13}C Max Error**	8.62	5.87	8.38	6.17	8.05	8.26	3.99	3.93	8.37	9.01
**^{1}H CMAE**	0.33	0.21	0.33	0.20	0.38	0.38	0.31	0.30	0.34	0.33
**^{1}H Max Error**	0.93	0.90	0.98	0.96	1.49	1.54	1.12	0.96	0.85	0.87
**H (DP4+)**	0.00%	6.52%	0.00%	21.61%	0.00%	0.00%	0.00%	0.00%	0.00%	0.00%
**C (DP4+)**	0.00%	0.27%	0.00%	1.09%	0.00%	0.00%	19.57%	78.95%	0.00%	0.00%
**DP4+**	0.00%	6.30%	0.00%	84.14%	0.00%	0.00%	0.00%	0.00%	0.00%	0.00%
**Boltzmann population**	21.64%	16.58%	13.30%	10.73%	7.76%	5.51%	6.22%	4.02%	4.24%	2.57%

**Conformer**	**4-11**	**4-12**	**4-13**	**4-14**
**^{13}C CMAE**	1.75	1.77	1.82	2.21
**^{13}C Max Error**	4.80	4.87	6.39	8.61
**^{1}H CMAE**	0.22	0.23	0.22	0.20
**^{1}H Max Error**	0.61	0.63	0.99	0.58
**H (DP4+)**	31.01%	7.34%	0.59%	32.92%
**C (DP4+)**	0.08%	0.04%	0.00%	0.00%
**DP4+**	8.61%	0.94%	0.01%	0.00%
**Boltzmann population**	3.24%	3.30%	0.84%	0.03%

Table S23. Correlation between computed (14 conformers) and experimental data of the *endo*-conformation of euphodendroid L (**4**). All calculations were done at the mPW1PW91/6-31+G** level.

**Conformer**	**4-1**	**4-2**	**4-3**	**4-4**	**4-5**	**4-6**	**4-7**	**4-8**	**4-9**	**4-10**
**^{13}C CMAE**	2.38	2.48	2.31	2.46	2.36	2.38	2.86	2.86	2.15	2.15
**^{13}C Max Error**	8.65	10.94	8.77	11.37	9.99	10.15	13.56	13.67	6.89	6.47
**^{1}H CMAE**	0.22	0.33	0.24	0.33	0.29	0.30	0.44	0.43	0.24	0.23
**^{1}H Max Error**	0.83	0.96	0.88	0.92	0.98	1.03	1.58	1.43	0.70	0.72
**H (DP4+)**	97.50%	0.00%	0.03%	0.00%	0.00%	0.00%	0.00%	0.00%	2.40%	0.07%
**C (DP4+)**	0.00%	0.00%	0.01%	0.00%	0.03%	0.01%	0.00%	0.00%	64.00%	35.96%
**DP4+**	0.02%	0.00%	0.00%	0.00%	0.00%	0.00%	0.00%	0.00%	98.44%	1.55%
**Boltzmann population**	21.64%	16.58%	13.30%	10.73%	7.76%	5.51%	6.22%	4.02%	4.24%	2.57%

**Conformer**	**4-11**	**4-12**	**4-13**	**4-14**
**^{13}C CMAE**	2.54	2.57	2.64	3.04
**^{13}C Max Error**	9.25	9.41	11.45	11.43
**^{1}H CMAE**	0.34	0.35	0.35	0.37

**^{1}H Max Error**	1.12	1.15	0.93	1.16
**H (DP4+)**	0.00%	0.00%	0.00%	0.00%
**C (DP4+)**	0.00%	0.00%	0.00%	0.00%
**DP4+**	0.00%	0.00%	0.00%	0.00%
**Boltzmann population**	3.24%	3.30%	0.84%	0.03%

Table S24. Correlation between computed (9 conformers) and experimental data of longilene peroxide (**5**). All calculations were done at the mPW1PW91/6-31+G** level.

**Conformer**	**5-1**	**5-2**	**5-3**	**5-4**	**5-5**	**5-6**	**5-7**	**5-8**	**5-9**
**^{13}C CMAE**	1.43	1.67	1.85	1.81	1.82	1.68	1.90	2.15	1.91
**^{13}C Max Error**	3.66	4.64	4.73	5.11	5.54	4.65	6.38	5.78	5.43
**^{1}H CMAE**	0.10	0.10	0.13	0.15	0.17	0.12	0.14	0.14	0.13
**^{1}H Max Error**	0.31	0.31	0.31	0.48	0.61	0.38	0.66	0.59	0.67
**H (DP4+)**	9.49%	86.85%	0.00%	0.00%	0.00%	3.66%	0.00%	0.00%	0.00%
**C (DP4+)**	100.00%	0.00%	0.00%	0.00%	0.00%	0.00%	0.00%	0.00%	0.00%
**DP4+**	100.00%	0.00%	0.00%	0.00%	0.00%	0.00%	0.00%	0.00%	0.00%
**$^{3}J_{H,H}$ RMSD**	0.91	0.51	0.49	1.25	0.63	0.62	0.30	0.41	0.38
**Boltzmann population**	98.70%	0.31%	0.73%	0.15%	0.00%	0.11%	0.00%	0.00%	0.00%

Table S25. Results of the correlation between computed and experimental data for the 255 possible combinations for conformers 5-1, 5-2, 5-5 and 5-9 of the longilene peroxide (**5**) using molar fraction steps of 0.1. All calculations were done at the mPW1PW91/6-31+G** level.

**Conformer 5-1**	100%	90%	90%	90%	80%	80%	80%	80%	80%	80%	70%	70%
**Conformer 5-2**	0%	10%	0%	0%	20%	10%	10%	0%	0%	0%	30%	20%
**Conformer 5-5**	0%	0%	10%	0%	0%	10%	0%	20%	10%	0%	0%	10%
**Conformer 5-9**	0%	0%	0%	10%	0%	0%	10%	0%	10%	20%	0%	0%
**^{13}C CMAE**	1.43	1.35	1.39	1.36	1.28	1.31	1.29	1.36	1.32	1.30	1.23	1.23
**^{13}C Max Error**	3.66	3.38	3.12	3.26	3.14	2.84	2.98	3.08	3.14	3.21	3.03	2.57
**^{1}H CMAE**	0.10	0.09	0.10	0.09	0.08	0.09	0.08	0.10	0.09	0.08	0.07	0.08
**^{1}H Max Error**	0.31	0.27	0.28	0.24	0.23	0.24	0.22	0.28	0.24	0.22	0.23	0.23
**H (DP4+)**	0.00%	0.00%	0.00%	0.00%	0.02%	0.00%	0.05%	0.00%	0.01%	0.15%	0.39%	0.10%
**C (DP4+)**	0.00%	0.00%	0.00%	0.00%	0.08%	0.05%	0.08%	0.01%	0.03%	0.05%	0.41%	0.70%
**DP4+**	0.00%	0.00%	0.00%	0.00%	0.00%	0.00%	0.01%	0.00%	0.00%	0.01%	0.22%	0.10%
**$^{3}J_{H,H}$ RMSD**	0.65	0.61	0.49	0.59	0.56	0.46	0.54	0.49	0.45	0.53	0.50	0.43

**Conformer 5-1**	70%	70%	70%	70%	70%	70%	70%	60%	60%	60%	60%	60%
**Conformer 5-2**	10%	10%	10%	0%	0%	0%	0%	40%	30%	30%	20%	20%
**Conformer 5-5**	20%	10%	0%	30%	20%	10%	0%	0%	10%	0%	20%	10%

**Conformer 5-9**	0%	10%	20%	0%	10%	20%	30%	0%	0%	10%	0%	10%
**^{13}C CMAE**	1.27	1.25	1.27	1.33	1.29	1.28	1.28	1.22	1.20	1.23	1.20	1.22
**^{13}C Max Error**	2.92	2.99	3.05	3.28	3.34	3.40	3.47	2.92	2.41	2.52	2.76	2.83
**^{1}H CMAE**	0.09	0.08	0.07	0.09	0.08	0.08	0.07	0.07	0.07	0.07	0.08	0.07
**^{1}H Max Error**	0.24	0.22	0.21	0.27	0.23	0.21	0.21	0.23	0.22	0.22	0.22	0.22
**H (DP4+)**	0.02%	0.43%	4.91%	0.00%	0.05%	1.02%	5.57%	1.51%	1.28%	6.03%	0.24%	3.92%
**C (DP4+)**	0.20%	0.42%	0.27%	0.02%	0.09%	0.18%	0.12%	0.36%	2.43%	0.70%	2.20%	1.99%
**DP4+**	0.00%	0.25%	1.81%	0.00%	0.01%	0.24%	0.95%	0.75%	4.29%	5.77%	0.72%	10.67%
**$^{3}J_{H,H}$ RMSD**	0.49	0.43	0.49	0.67	0.51	0.44	0.48	0.08	0.39	0.071	0.50	0.40

**Conformer 5-1**	60%	60%	60%	60%	60%	60%	60%	60%	60%	60%	50%	50%
**Conformer 5-2**	20%	10%	10%	10%	10%	0%	0%	0%	0%	0%	50%	40%
**Conformer 5-5**	0%	30%	20%	10%	0%	40%	30%	20%	10%	0%	0%	10%
**Conformer 5-9**	20%	0%	10%	20%	30%	0%	10%	20%	30%	40%	0%	0%
**^{13}C CMAE**	1.25	1.24	1.23	1.24	1.27	1.31	1.27	1.26	1.27	1.30	1.24	1.20
**^{13}C Max Error**	2.89	3.12	3.18	3.25	3.31	3.48	3.54	3.60	3.66	3.73	2.82	2.65
**^{1}H CMAE**	0.07	0.09	0.08	0.07	0.07	0.10	0.09	0.08	0.08	0.07	0.07	0.07
**^{1}H Max Error**	0.21	0.24	0.22	0.21	0.21	0.29	0.25	0.22	0.20	0.20	0.22	0.22
**H (DP4+)**	16.13%	0.01%	0.26%	2.97%	13.17%	0.00%	0.01%	0.08%	0.87%	3.88%	1.56%	2.67%
**C (DP4+)**	0.55%	0.54%	1.13%	0.99%	0.27%	0.04%	0.18%	0.40%	0.31%	0.08%	0.07%	1.93%
**DP4+**	12.24%	0.01%	0.40%	4.03%	4.88%	0.00%	0.00%	0.05%	0.37%	0.44%	0.16%	7.07%
**$^{3}J_{H,H}$ RMSD**	0.08	0.69	0.52	0.42	0.11	0.91	0.72	0.55	0.44	0.14	0.43	0.23

**Conformer 5-1**	50%	50%	50%	50%	50%	50%	50%	50%	50%	50%	50%	50%
**Conformer 5-2**	40%	30%	30%	30%	20%	20%	20%	20%	10%	10%	10%	10%
**Conformer 5-5**	0%	20%	10%	0%	30%	20%	10%	0%	40%	30%	20%	10%
**Conformer 5-9**	10%	0%	10%	20%	0%	10%	20%	30%	0%	10%	20%	30%
**^{13}C CMAE**	1.24	1.17	1.21	1.24	1.19	1.20	1.23	1.27	1.23	1.22	1.23	1.26
**^{13}C Max Error**	2.41	2.61	2.67	2.73	2.96	3.02	3.09	3.15	3.32	3.38	3.44	3.50
**^{1}H CMAE**	0.07	0.08	0.07	0.07	0.09	0.08	0.07	0.07	0.09	0.09	0.08	0.07
**^{1}H Max Error**	0.22	0.22	0.22	0.21	0.23	0.21	0.21	0.20	0.28	0.24	0.21	0.20
**H (DP4+)**	5.92%	0.60%	3.87%	7.32%	0.02%	0.24%	1.49%	3.08%	0.00%	0.01%	0.07%	0.44%
**C (DP4+)**	0.35%	7.45%	3.22%	0.53%	4.44%	4.70%	1.73%	0.28%	0.70%	1.81%	1.62%	0.57%
**DP4+**	2.81%	6.10%	17.12%	5.33%	0.13%	1.52%	3.54%	1.19%	0.00%	0.02%	0.16%	0.35%
**$^{3}J_{H,H}$ RMSD**	0.40	0.44	0.22	0.38	0.62	0.45	0.20	0.36	0.84	0.62	0.45	0.20

**Conformer 5-1**	50%	50%	50%	50%	50%	50%	50%	40%	40%	40%	40%	40%
**Conformer 5-2**	10%	0%	0%	0%	0%	0%	0%	60%	50%	50%	40%	40%
**Conformer 5-5**	0%	50%	40%	30%	20%	10%	0%	0%	10%	0%	20%	10%
**Conformer 5-9**	40%	0%	10%	20%	30%	40%	50%	0%	0%	10%	0%	10%

**^{13}C CMAE**	1.30	1.30	1.28	1.27	1.27	1.29	1.32	1.29	1.23	1.28	1.19	1.23
**^{13}C Max Error**	3.57	3.71	3.74	3.80	3.86	3.92	3.99	3.17	3.02	2.75	2.87	2.60
**^{1}H CMAE**	0.07	0.11	0.10	0.09	0.08	0.08	0.08	0.08	0.08	0.07	0.08	0.07
**^{1}H Max Error**	0.20	0.33	0.29	0.26	0.22	0.20	0.20	0.22	0.22	0.22	0.22	0.21
**H (DP4+)**	1.00%	0.00%	0.00%	0.00%	0.02%	0.12%	0.31%	0.80%	1.26%	1.73%	0.28%	0.80%
**C (DP4+)**	0.10%	0.04%	0.16%	0.41%	0.32%	0.13%	0.03%	0.01%	0.46%	0.03%	5.05%	1.13%
**DP4+**	0.14%	0.00%	0.00%	0.00%	0.01%	0.02%	0.01%	0.01%	0.79%	0.08%	1.97%	1.24%
**$^{3}J_{H,H}$ RMSD**	0.34	1.07	0.84	0.62	0.47	0.20	0.32	0.40	0.38	0.39	0.09	0.37
**Conformer 5-1**	40%	40%	40%	40%	40%	40%	40%	40%	40%	40%	40%	40%
**Conformer 5-2**	40%	30%	30%	30%	30%	20%	20%	20%	20%	20%	10%	10%
**Conformer 5-5**	0%	30%	20%	10%	0%	40%	30%	20%	10%	0%	50%	40%
**Conformer 5-9**	20%	0%	10%	20%	30%	0%	10%	20%	30%	40%	0%	10%
**^{13}C CMAE**	1.27	1.17	1.20	1.23	1.27	1.19	1.20	1.22	1.26	1.30	1.24	1.24
**^{13}C Max Error**	2.81	2.80	2.87	2.93	3.00	3.16	3.22	3.28	3.35	3.41	3.52	3.58
**^{1}H CMAE**	0.07	0.09	0.08	0.08	0.07	0.10	0.09	0.08	0.08	0.08	0.10	0.10
**^{1}H Max Error**	0.21	0.22	0.21	0.21	0.20	0.27	0.23	0.21	0.20	0.20	0.32	0.28
**H (DP4+)**	1.17%	0.01%	0.09%	0.28%	0.43%	0.00%	0.00%	0.03%	0.09%	0.15%	0.00%	0.00%
**C (DP4+)**	0.10%	8.98%	5.63%	1.42%	0.16%	2.83%	4.25%	2.09%	0.59%	0.08%	0.28%	0.79%
**DP4+**	0.16%	0.17%	0.68%	0.54%	0.09%	0.00%	0.03%	0.08%	0.07%	0.02%	0.00%	0.00%
**$^{3}J_{H,H}$ RMSD**	0.39	0.37	0.11	0.36	0.39	0.44	0.39	0.13	0.35	0.40	0.58	0.45
**Conformer 5-1**	40%	40%	40%	40%	40%	40%	40%	40%	40%	40%	40%	30%
**Conformer 5-2**	10%	10%	10%	10%	0%	0%	0%	0%	0%	0%	0%	70%
**Conformer 5-5**	30%	20%	10%	0%	60%	50%	40%	30%	20%	10%	0%	0%
**Conformer 5-9**	20%	30%	40%	50%	0%	10%	20%	30%	40%	50%	60%	0%
**^{13}C CMAE**	1.24	1.25	1.28	1.33	1.34	1.31	1.30	1.29	1.29	1.33	1.38	1.35
**^{13}C Max Error**	3.64	3.70	3.76	3.83	4.08	3.94	4.00	4.06	4.12	4.18	4.25	3.54
**^{1}H CMAE**	0.09	0.08	0.08	0.08	0.12	0.11	0.10	0.09	0.08	0.08	0.08	0.08
**^{1}H Max Error**	0.24	0.21	0.21	0.22	0.37	0.33	0.29	0.26	0.27	0.27	0.28	0.22
**H (DP4+)**	0.00%	0.01%	0.03%	0.05%	0.00%	0.00%	0.00%	0.00%	0.00%	0.01%	0.02%	0.18%
**C (DP4+)**	0.86%	0.49%	0.15%	0.02%	0.00%	0.03%	0.09%	0.10%	0.07%	0.02%	0.00%	0.00%
**DP4+**	0.00%	0.01%	0.01%	0.00%	0.00%	0.00%	0.00%	0.00%	0.00%	0.00%	0.00%	0.00%
**$^{3}J_{H,H}$ RMSD**	0.41	0.15	0.35	0.41	0.79	0.59	0.46	0.44	0.18	0.34	0.43	0.37
**Conformer 5-1**	30%	30%	30%	30%	30%	30%	30%	30%	30%	30%	30%	30%
**Conformer 5-2**	60%	60%	50%	50%	50%	40%	40%	40%	40%	30%	30%	30%
**Conformer 5-5**	10%	0%	20%	10%	0%	30%	20%	10%	0%	40%	30%	20%
**Conformer 5-9**	0%	10%	0%	10%	20%	0%	10%	20%	30%	0%	10%	20%
**^{13}C CMAE**	1.27	1.32	1.22	1.27	1.32	1.19	1.22	1.27	1.32	1.18	1.19	1.23

**^{13}C Max Error**	3.39	3.12	3.24	2.97	3.08	3.09	2.82	2.97	3.27	3.00	3.06	3.12
**^{1}H CMAE**	0.08	0.08	0.08	0.08	0.08	0.09	0.08	0.08	0.08	0.10	0.09	0.08
**^{1}H Max Error**	0.22	0.21	0.22	0.21	0.21	0.22	0.21	0.21	0.21	0.25	0.22	0.20
**H (DP4+)**	0.25%	0.17%	0.05%	0.10%	0.06%	0.00%	0.02%	0.03%	0.02%	0.00%	0.00%	0.01%
**C (DP4+)**	0.03%	0.00%	0.69%	0.11%	0.01%	3.45%	1.23%	0.20%	0.01%	3.46%	2.94%	1.06%
**DP4+**	0.01%	0.00%	0.05%	0.01%	0.00%	0.02%	0.03%	0.01%	0.00%	0.00%	0.01%	0.01%
**$^{3}J_{H,H}$ RMSD**	0.39	0.37	0.55	0.41	0.37	0.41	0.56	0.43	0.38	0.53	0.42	0.57
**Conformer 5-1**	30%	30%	30%	30%	30%	30%	30%	30%	30%	30%	30%	30%
**Conformer 5-2**	30%	30%	20%	20%	20%	20%	20%	20%	10%	10%	10%	10%
**Conformer 5-5**	10%	0%	50%	40%	30%	20%	10%	0%	60%	50%	40%	30%
**Conformer 5-9**	30%	40%	0%	10%	20%	30%	40%	50%	0%	10%	20%	30%
**^{13}C CMAE**	1.27	1.33	1.21	1.21	1.22	1.25	1.30	1.37	1.30	1.29	1.28	1.27
**^{13}C Max Error**	3.19	3.46	3.36	3.42	3.48	3.54	3.61	3.67	3.87	3.78	3.84	3.90
**^{1}H CMAE**	0.08	0.08	0.11	0.10	0.09	0.08	0.08	0.08	0.12	0.11	0.10	0.09
**^{1}H Max Error**	0.21	0.23	0.30	0.27	0.24	0.25	0.25	0.26	0.35	0.32	0.29	0.30
**H (DP4+)**	0.01%	0.01%	0.00%	0.00%	0.00%	0.00%	0.00%	0.00%	0.00%	0.00%	0.00%	0.00%
**C (DP4+)**	0.21%	0.02%	0.74%	1.25%	0.94%	0.33%	0.07%	0.01%	0.02%	0.07%	0.14%	0.13%
**DP4+**	0.00%	0.00%	0.00%	0.00%	0.00%	0.00%	0.00%	0.00%	0.00%	0.00%	0.00%	0.00%
**$^{3}J_{H,H}$ RMSD**	0.46	0.39	0.46	0.56	0.44	0.57	0.49	0.40	0.44	0.47	0.58	0.46
**Conformer 5-1**	30%	30%	30%	30%	30%	30%	30%	30%	30%	30%	30%	20%
**Conformer 5-2**	10%	10%	10%	0%	0%	0%	0%	0%	0%	0%	0%	80%
**Conformer 5-5**	20%	10%	0%	70%	60%	50%	40%	30%	20%	10%	0%	0%
**Conformer 5-9**	40%	50%	60%	0%	10%	20%	30%	40%	50%	60%	70%	0%
**^{13}C CMAE**	1.29	1.34	1.41	1.41	1.38	1.36	1.35	1.35	1.38	1.42	1.48	1.43
**^{13}C Max Error**	3.96	4.02	4.09	4.44	4.13	4.19	4.26	4.32	4.38	4.44	4.51	3.91
**^{1}H CMAE**	0.09	0.09	0.09	0.13	0.12	0.11	0.10	0.09	0.09	0.09	0.09	0.09
**^{1}H Max Error**	0.31	0.31	0.32	0.40	0.37	0.35	0.35	0.36	0.36	0.37	0.37	0.22
**H (DP4+)**	0.00%	0.00%	0.00%	0.00%	0.00%	0.00%	0.00%	0.00%	0.00%	0.00%	0.00%	0.01%
**C (DP4+)**	0.05%	0.01%	0.00%	0.00%	0.00%	0.00%	0.01%	0.01%	0.00%	0.00%	0.00%	0.00%
**DP4+**	0.00%	0.00%	0.00%	0.00%	0.00%	0.00%	0.00%	0.00%	0.00%	0.00%	0.00%	0.00%
**$^{3}J_{H,H}$ RMSD**	0.57	0.52	0.42	0.55	0.45	0.49	0.61	0.48	0.58	0.55	0.44	0.34
**Conformer 5-1**	20%	20%	20%	20%	20%	20%	20%	20%	20%	20%	20%	20%
**Conformer 5-2**	60%	60%	40%	40%	40%	20%	20%	20%	20%	0%	0%	0%
**Conformer 5-5**	20%	0%	40%	20%	0%	60%	40%	20%	0%	80%	60%	40%
**Conformer 5-9**	0%	20%	0%	20%	40%	0%	20%	40%	60%	0%	20%	40%
**^{13}C CMAE**	1.27	1.37	1.19	1.27	1.40	1.28	1.26	1.32	1.45	1.54	1.47	1.44
**^{13}C Max Error**	3.61	3.35	3.31	3.13	3.73	3.67	3.68	3.80	4.12	4.81	4.39	4.52

**^{1}H CMAE**	0.09	0.09	0.10	0.09	0.09	0.12	0.10	0.09	0.10	0.14	0.12	0.11
**^{1}H Max Error**	0.22	0.24	0.24	0.23	0.27	0.34	0.33	0.35	0.36	0.44	0.44	0.45
**H (DP4+)**	0.01%	0.00%	0.00%	0.00%	0.00%	0.00%	0.00%	0.00%	0.00%	0.00%	0.00%	0.00%
**C (DP4+)**	0.04%	0.00%	1.26%	0.13%	0.00%	0.03%	0.12%	0.02%	0.00%	0.00%	0.00%	0.00%
**DP4+**	0.00%	0.00%	0.00%	0.00%	0.00%	0.00%	0.00%	0.00%	0.00%	0.00%	0.00%	0.00%
**$^{3}J_{H,H}$ RMSD**	0.58	0.35	0.73	0.64	0.38	0.67	0.76	0.71	0.43	0.46	0.70	0.80

**Conformer 5-1**	20%	20%	10%	10%	10%	10%	10%	10%	10%	10%	10%	10%
**Conformer 5-2**	0%	0%	90%	80%	80%	70%	70%	70%	60%	60%	60%	60%
**Conformer 5-5**	20%	0%	0%	10%	0%	20%	10%	0%	30%	20%	10%	0%
**Conformer 5-9**	60%	80%	0%	0%	10%	0%	10%	20%	0%	10%	20%	30%
**^{13}C CMAE**	1.50	1.61	1.53	1.41	1.49	1.32	1.37	1.45	1.27	1.32	1.38	1.46
**^{13}C Max Error**	4.64	4.76	4.27	4.12	3.85	3.97	3.70	3.62	3.83	3.55	3.51	3.81
**^{1}H CMAE**	0.10	0.10	0.10	0.09	0.10	0.10	0.10	0.10	0.10	0.10	0.10	0.10
**^{1}H Max Error**	0.46	0.47	0.26	0.24	0.28	0.22	0.26	0.29	0.22	0.24	0.27	0.30
**H (DP4+)**	0.00%	0.00%	0.00%	0.00%	0.00%	0.00%	0.00%	0.00%	0.00%	0.00%	0.00%	0.00%
**C (DP4+)**	0.00%	0.00%	0.00%	0.00%	0.00%	0.00%	0.00%	0.00%	0.00%	0.00%	0.00%	0.00%
**DP4+**	0.00%	0.00%	0.00%	0.00%	0.00%	0.00%	0.00%	0.00%	0.00%	0.00%	0.00%	0.00%
**$^{3}J_{H,H}$ RMSD**	0.78	0.48	0.32	0.41	0.33	0.61	0.44	0.35	0.84	0.64	0.47	0.37

**Conformer 5-1**	10%	10%	10%	10%	10%	10%	10%	10%	10%	10%	10%	10%
**Conformer 5-2**	50%	50%	50%	50%	50%	40%	40%	40%	40%	40%	40%	30%
**Conformer 5-5**	40%	30%	20%	10%	0%	50%	40%	30%	20%	10%	0%	60%
**Conformer 5-9**	0%	10%	20%	30%	40%	0%	10%	20%	30%	40%	50%	0%
**^{13}C CMAE**	1.22	1.26	1.33	1.40	1.47	1.24	1.23	1.28	1.34	1.41	1.49	1.30
**^{13}C Max Error**	3.68	3.40	3.40	3.70	4.00	3.53	3.26	3.30	3.60	3.90	4.20	3.47
**^{1}H CMAE**	0.10	0.10	0.10	0.10	0.10	0.11	0.10	0.10	0.10	0.10	0.10	0.12
**^{1}H Max Error**	0.26	0.26	0.27	0.28	0.32	0.31	0.32	0.32	0.33	0.33	0.34	0.36
**H (DP4+)**	0.00%	0.00%	0.00%	0.00%	0.00%	0.00%	0.00%	0.00%	0.00%	0.00%	0.00%	0.00%
**C (DP4+)**	0.00%	0.00%	0.00%	0.00%	0.00%	0.00%	0.00%	0.00%	0.00%	0.00%	0.00%	0.00%
**DP4+**	0.00%	0.00%	0.00%	0.00%	0.00%	0.00%	0.00%	0.00%	0.00%	0.00%	0.00%	0.00%
**$^{3}J_{H,H}$ RMSD**	1.09	0.88	0.67	0.50	0.39	1.05	1.13	0.91	0.71	0.53	0.41	1.09

**Conformer 5-1**	10%	10%	10%	10%	10%	10%	10%	10%	10%	10%	10%	10%
**Conformer 5-2**	30%	30%	30%	30%	30%	30%	20%	20%	20%	20%	20%	20%
**Conformer 5-5**	50%	40%	30%	20%	10%	0%	70%	60%	50%	40%	30%	20%
**Conformer 5-9**	10%	20%	30%	40%	50%	60%	0%	10%	20%	30%	40%	50%
**^{13}C CMAE**	1.29	1.29	1.33	1.39	1.45	1.51	1.40	1.37	1.35	1.36	1.40	1.45
**^{13}C Max Error**	3.46	3.52	3.58	3.79	4.09	4.39	4.04	3.81	3.88	3.94	4.00	4.06
**^{1}H CMAE**	0.11	0.11	0.10	0.10	0.10	0.11	0.13	0.12	0.11	0.11	0.10	0.10

**^{1}H Max Error**	0.37	0.37	0.38	0.39	0.39	0.40	0.41	0.42	0.43	0.43	0.44	0.44
**H (DP4+)**	0.00%	0.00%	0.00%	0.00%	0.00%	0.00%	0.00%	0.00%	0.00%	0.00%	0.00%	0.00%
**C (DP4+)**	0.00%	0.00%	0.00%	0.00%	0.00%	0.00%	0.00%	0.00%	0.00%	0.00%	0.00%	0.00%
**DP4+**	0.00%	0.00%	0.00%	0.00%	0.00%	0.00%	0.00%	0.00%	0.00%	0.00%	0.00%	0.00%
**$^{3}J_{H,H}$ RMSD**	1.06	1.15	0.95	0.74	0.56	0.44	0.94	1.12	1.08	1.16	0.98	0.78

**Conformer 5-1**	10%	10%	10%	10%	10%	10%	10%	10%	10%	10%	10%	10%
**Conformer 5-2**	20%	20%	10%	10%	10%	10%	10%	10%	10%	10%	10%	0%
**Conformer 5-5**	10%	0%	80%	70%	60%	50%	40%	30%	20%	10%	0%	90%
**Conformer 5-9**	60%	70%	0%	10%	20%	30%	40%	50%	60%	70%	80%	0%
**^{13}C CMAE**	1.51	1.57	1.53	1.48	1.46	1.46	1.47	1.49	1.54	1.60	1.66	1.68
**^{13}C Max Error**	4.28	4.58	4.60	4.17	4.23	4.30	4.36	4.42	4.48	4.54	4.77	5.17
**^{1}H CMAE**	0.11	0.11	0.14	0.13	0.12	0.12	0.11	0.11	0.11	0.11	0.11	0.15
**^{1}H Max Error**	0.45	0.45	0.47	0.47	0.48	0.48	0.49	0.49	0.50	0.51	0.51	0.52
**H (DP4+)**	0.00%	0.00%	0.00%	0.00%	0.00%	0.00%	0.00%	0.00%	0.00%	0.00%	0.00%	0.00%
**C (DP4+)**	0.00%	0.00%	0.00%	0.00%	0.00%	0.00%	0.00%	0.00%	0.00%	0.00%	0.00%	0.00%
**DP4+**	0.00%	0.00%	0.00%	0.00%	0.00%	0.00%	0.00%	0.00%	0.00%	0.00%	0.00%	0.00%
**$^{3}J_{H,H}$ RMSD**	0.60	0.47	0.74	0.96	1.14	1.10	1.17	1.02	0.82	0.63	0.50	0.58

**Conformer 5-1**	10%	10%	10%	10%	10%	10%	10%	10%	10%	0%	0%	0%
**Conformer 5-2**	0%	0%	0%	0%	0%	0%	0%	0%	0%	100%	90%	90%
**Conformer 5-5**	80%	70%	60%	50%	40%	30%	20%	10%	0%	0%	10%	0%
**Conformer 5-9**	10%	20%	30%	40%	50%	60%	70%	80%	90%	0%	0%	10%
**^{13}C CMAE**	1.64	1.60	1.59	1.58	1.57	1.59	1.64	1.69	1.74	1.67	1.52	1.61
**^{13}C Max Error**	4.74	4.59	4.65	4.71	4.78	4.84	4.90	4.96	5.02	4.64	4.49	4.22
**^{1}H CMAE**	0.14	0.13	0.13	0.12	0.11	0.11	0.11	0.11	0.12	0.10	0.10	0.11
**^{1}H Max Error**	0.52	0.53	0.54	0.54	0.55	0.55	0.56	0.56	0.57	0.31	0.29	0.32
**H (DP4+)**	0.00%	0.00%	0.00%	0.00%	0.00%	0.00%	0.00%	0.00%	0.00%	0.00%	0.00%	0.00%
**C (DP4+)**	0.00%	0.00%	0.00%	0.00%	0.00%	0.00%	0.00%	0.00%	0.00%	0.00%	0.00%	0.00%
**DP4+**	0.00%	0.00%	0.00%	0.00%	0.00%	0.00%	0.00%	0.00%	0.00%	0.00%	0.00%	0.00%
**$^{3}J_{H,H}$ RMSD**	0.75	0.97	1.16	1.12	1.18	1.06	0.85	0.67	0.53	0.31	0.43	0.33

**Conformer 5-1**	0%	0%	0%	0%	0%	0%	0%	0%	0%	0%	0%	0%
**Conformer 5-2**	80%	80%	80%	70%	70%	70%	70%	60%	60%	60%	60%	60%
**Conformer 5-5**	20%	10%	0%	30%	20%	10%	0%	40%	30%	20%	10%	0%
**Conformer 5-9**	0%	10%	20%	0%	10%	20%	30%	0%	10%	20%	30%	40%
**^{13}C CMAE**	1.40	1.48	1.57	1.31	1.38	1.46	1.55	1.28	1.33	1.39	1.47	1.56
**^{13}C Max Error**	4.34	4.07	3.89	4.19	3.92	3.78	4.08	4.04	3.77	3.67	3.97	4.27
**^{1}H CMAE**	0.10	0.10	0.11	0.10	0.11	0.11	0.11	0.11	0.11	0.11	0.11	0.11
**^{1}H Max Error**	0.27	0.30	0.34	0.26	0.28	0.32	0.35	0.30	0.31	0.31	0.33	0.36

**H (DP4+)**	0.00%	0.00%	0.00%	0.00%	0.00%	0.00%	0.00%	0.00%	0.00%	0.00%	0.00%	0.00%
**C (DP4+)**	0.00%	0.00%	0.00%	0.00%	0.00%	0.00%	0.00%	0.00%	0.00%	0.00%	0.00%	0.00%
**DP4+**	0.00%	0.00%	0.00%	0.00%	0.00%	0.00%	0.00%	0.00%	0.00%	0.00%	0.00%	0.00%
**$^3J_{H,H}$ RMSD**	0.64	0.46	0.35	0.88	0.67	0.49	0.37	1.13	0.92	0.71	0.53	0.40
**Conformer 5-1**	0%	0%	0%	0%	0%	0%	0%	0%	0%	0%	0%	0%
**Conformer 5-2**	50%	50%	50%	50%	50%	50%	40%	40%	40%	40%	40%	40%
**Conformer 5-5**	50%	40%	30%	20%	10%	0%	60%	50%	40%	30%	20%	10%
**Conformer 5-9**	0%	10%	20%	30%	40%	50%	0%	10%	20%	30%	40%	50%
**^{13}C CMAE**	1.28	1.32	1.36	1.41	1.49	1.58	1.34	1.33	1.38	1.43	1.48	1.53
**^{13}C Max Error**	3.89	3.62	3.57	3.87	4.16	4.46	3.74	3.47	3.46	3.76	4.06	4.36
**^{1}H CMAE**	0.12	0.11	0.11	0.11	0.11	0.11	0.13	0.12	0.11	0.11	0.11	0.11
**^{1}H Max Error**	0.35	0.36	0.36	0.37	0.37	0.38	0.40	0.41	0.41	0.42	0.43	0.43
**H (DP4+)**	0.00%	0.00%	0.00%	0.00%	0.00%	0.00%	0.00%	0.00%	0.00%	0.00%	0.00%	0.00%
**C (DP4+)**	0.00%	0.00%	0.00%	0.00%	0.00%	0.00%	0.00%	0.00%	0.00%	0.00%	0.00%	0.00%
**DP4+**	0.00%	0.00%	0.00%	0.00%	0.00%	0.00%	0.00%	0.00%	0.00%	0.00%	0.00%	0.00%
**$^3J_{H,H}$ RMSD**	1.39	1.17	0.95	0.75	0.56	0.43	1.37	1.42	1.20	0.99	0.78	0.60
**Conformer 5-1**	0%	0%	0%	0%	0%	0%	0%	0%	0%	0%	0%	0%
**Conformer 5-2**	40%	30%	30%	30%	30%	30%	30%	30%	30%	20%	20%	20%
**Conformer 5-5**	0%	70%	60%	50%	40%	30%	20%	10%	0%	80%	70%	60%
**Conformer 5-9**	60%	0%	10%	20%	30%	40%	50%	60%	70%	0%	10%	20%
**^{13}C CMAE**	1.61	1.44	1.41	1.41	1.45	1.50	1.54	1.59	1.67	1.53	1.50	1.49
**^{13}C Max Error**	4.66	3.83	3.66	3.72	3.78	3.95	4.25	4.55	4.85	4.40	4.01	4.07
**^{1}H CMAE**	0.12	0.14	0.13	0.12	0.11	0.11	0.11	0.12	0.12	0.15	0.14	0.13
**^{1}H Max Error**	0.44	0.45	0.46	0.47	0.47	0.48	0.48	0.49	0.49	0.51	0.51	0.52
**H (DP4+)**	0.00%	0.00%	0.00%	0.00%	0.00%	0.00%	0.00%	0.00%	0.00%	0.00%	0.00%	0.00%
**C (DP4+)**	0.00%	0.00%	0.00%	0.00%	0.00%	0.00%	0.00%	0.00%	0.00%	0.00%	0.00%	0.00%
**DP4+**	0.00%	0.00%	0.00%	0.00%	0.00%	0.00%	0.00%	0.00%	0.00%	0.00%	0.00%	0.00%
**$^3J_{H,H}$ RMSD**	0.46	1.40	1.38	1.45	1.24	1.02	0.82	0.63	0.49	1.24	1.42	1.40
**Conformer 5-1**	0%	0%	0%	0%	0%	0%	0%	0%	0%	0%	0%	0%
**Conformer 5-2**	20%	20%	20%	20%	20%	20%	10%	10%	10%	10%	10%	10%
**Conformer 5-5**	50%	40%	30%	20%	10%	0%	90%	80%	70%	60%	50%	40%
**Conformer 5-9**	30%	40%	50%	60%	70%	80%	0%	10%	20%	30%	40%	50%
**^{13}C CMAE**	1.49	1.51	1.56	1.61	1.66	1.73	1.67	1.63	1.60	1.59	1.59	1.60
**^{13}C Max Error**	4.14	4.20	4.26	4.44	4.74	5.04	4.97	4.54	4.43	4.49	4.56	4.62
**^{1}H CMAE**	0.12	0.12	0.12	0.12	0.12	0.12	0.16	0.15	0.14	0.13	0.13	0.12
**^{1}H Max Error**	0.52	0.53	0.54	0.54	0.55	0.55	0.56	0.56	0.57	0.58	0.58	0.59
**H (DP4+)**	0.00%	0.00%	0.00%	0.00%	0.00%	0.00%	0.00%	0.00%	0.00%	0.00%	0.00%	0.00%

**C (DP4+)**	0.00%	0.00%	0.00%	0.00%	0.00%	0.00%	0.00%	0.00%	0.00%	0.00%	0.00%	0.00%
**DP4+**	0.00%	0.00%	0.00%	0.00%	0.00%	0.00%	0.00%	0.00%	0.00%	0.00%	0.00%	0.00%
**$^{3}J_{H,H}$ RMSD**	1.47	1.27	1.06	0.85	0.67	0.52	1.02	1.26	1.44	1.42	1.48	1.31

**Conformer 5-1**	0%	0%	0%	0%	0%	0%	0%	0%	0%	0%	0%	0%
**Conformer 5-2**	10%	10%	10%	10%	0%	0%	0%	0%	0%	0%	0%	0%
**Conformer 5-5**	30%	20%	10%	0%	100%	90%	80%	70%	60%	50%	40%	30%
**Conformer 5-9**	60%	70%	80%	90%	0%	10%	20%	30%	40%	50%	60%	70%
**^{13}C CMAE**	1.65	1.70	1.75	1.82	1.82	1.78	1.75	1.73	1.72	1.71	1.70	1.74
**^{13}C Max Error**	4.68	4.74	4.94	5.24	5.54	5.10	4.79	4.85	4.91	4.97	5.04	5.10
**^{1}H CMAE**	0.12	0.12	0.13	0.13	0.17	0.16	0.15	0.14	0.14	0.13	0.12	0.13
**^{1}H Max Error**	0.59	0.60	0.60	0.61	0.61	0.62	0.62	0.63	0.63	0.64	0.64	0.65
**H (DP4+)**	0.00%	0.00%	0.00%	0.00%	0.00%	0.00%	0.00%	0.00%	0.00%	0.00%	0.00%	0.00%
**C (DP4+)**	0.00%	0.00%	0.00%	0.00%	0.00%	0.00%	0.00%	0.00%	0.00%	0.00%	0.00%	0.00%
**DP4+**	0.00%	0.00%	0.00%	0.00%	0.00%	0.00%	0.00%	0.00%	0.00%	0.00%	0.00%	0.00%
**$^{3}J_{H,H}$ RMSD**	1.10	0.89	0.70	0.55	0.81	1.04	1.27	1.46	1.43	1.49	1.35	1.13

**Conformer 5-1**	0%	0%	0%
**Conformer 5-2**	0%	0%	0%
**Conformer 5-5**	20%	10%	0%
**Conformer 5-9**	80%	90%	100%
**^{13}C CMAE**	1.79	1.84	1.91
**^{13}C Max Error**	5.16	5.22	5.43
**^{1}H CMAE**	0.13	0.13	0.13
**^{1}H Max Error**	0.66	0.66	0.67
**H (DP4+)**	0.00%	0.00%	0.00%
**C (DP4+)**	0.00%	0.00%	0.00%
**DP4+**	0.00%	0.00%	0.00%
**$^{3}J_{H,H}$ RMSD**	0.93	0.74	0.59

6. Cartesian coordinates for compounds 1-5.

Cartesian coordinates for conformer 1-1 after optimization at the PCM/B3LYP/6-31G* level of theory. Number of imaginary frequencies = 0. SCF Energy (PCM/mPW1PW91/6-31+G**) = -1884.87730667.

Atom	X	Y	Z
O	2.578261	-0.157963	0.421051
O	2.760516	1.069757	-1.567415
O	5.301079	0.798597	0.964376
O	6.466295	-0.626911	-0.978593
O	0.981736	3.993911	0.848563
O	-1.229241	2.107392	-1.302196
O	0.014494	-3.367957	1.501343
O	-1.622104	-0.662016	0.289334
O	-0.048138	-3.517574	-1.216406
O	-2.525642	-2.328687	-0.963758
O	-4.959757	-1.471066	-2.347442
C	2.736117	2.267386	0.604309
C	3.168329	0.980553	-0.205463
C	4.702917	0.7856	-0.326107
C	1.267464	2.721281	0.249066
C	5.040616	-0.542184	-1.015204
C	2.851929	-1.431906	-0.197327
C	4.358209	-1.694249	-0.276416
C	2.861743	2.046271	2.126914
C	3.657852	3.440637	0.19789
C	0.139675	1.735142	0.597557
C	2.128313	-2.508348	0.615027
C	-1.231236	2.239476	0.121608
C	0.617709	-2.287928	0.777579
C	-2.444758	1.535351	0.779712
C	-0.123308	-2.270478	-0.570962
C	-2.826104	0.153142	0.232851
C	7.028102	-1.58232	-1.858778
C	-1.575873	-1.796759	-0.434773
C	-3.95692	-0.493854	1.016478
C	-2.150125	2.949091	-1.973198
C	0.117336	-3.269679	2.910816
C	-5.225857	-0.499085	0.581471
C	-3.575373	-1.158104	2.319527
C	-5.811376	0.117043	-0.66806
C	-7.073769	0.939606	-0.328481
C	-6.122169	-0.987355	-1.702658
C	-6.805688	2.148275	0.573466
H	5.11939	1.598803	-0.932115
H	1.228806	2.932371	-0.824003
H	4.698609	-0.507263	-2.058739
H	2.457856	-1.419175	-1.222169
H	4.533889	-2.64989	-0.784937
H	4.780208	-1.772112	0.731543
H	2.278571	1.187083	2.467499
H	3.90114	1.876363	2.407947
H	2.517616	2.937623	2.666484
H	4.690899	3.251087	0.500194
H	3.63188	3.609432	-0.884737
H	3.325303	4.356149	0.690586
H	0.332188	0.763632	0.14459
H	0.118443	1.5798	1.684164
H	2.587115	-2.55309	1.610679
H	2.291418	-3.482625	0.141559
H	-1.301474	3.305587	0.383099
H	1.790883	1.112496	-1.593462
H	0.444291	-1.347882	1.310496
H	6.227887	0.544679	0.813232
H	-2.257222	1.475028	1.858902

H	-3.334932	2.165682	0.664537
H	0.816475	3.839443	1.792472
H	0.348914	-1.544997	-1.242615
H	-3.094217	0.237264	-0.821384
H	6.757667	-2.610905	-1.583046
H	6.716563	-1.39883	-2.897602
H	8.114231	-1.477921	-1.791564
H	-0.406342	-4.168663	-0.588982
H	-3.192954	2.720548	-1.709832
H	-1.953823	4.009199	-1.755439
H	-2.01912	2.775123	-3.04437
H	-0.343783	-2.342555	3.280804
H	1.160097	-3.309824	3.253543
H	-0.4204	-4.124774	3.328764
H	-5.960232	-1.021421	1.199351
H	-3.038133	-0.468171	2.983181
H	-4.46317	-1.509885	2.853298
H	-2.918724	-2.020722	2.153654
H	-5.088687	0.788667	-1.147082
H	-7.818532	0.286397	0.148515
H	-7.52416	1.287986	-1.267308
H	-6.682194	-1.805527	-1.218113
H	-6.75749	-0.579785	-2.497939
H	-6.06407	2.819412	0.122366
H	-7.722366	2.727411	0.732692
H	-6.428848	1.842852	1.555673
H	-4.341999	-1.833596	-1.686295

Cartesian coordinates for conformer 1-2 after optimization at the PCM/B3LYP/6-31G* level of theory. Number of imaginary frequencies = 0. SCF Energy (PCM/mPW1PW91/6-31+G**) = -1884.88296999.

Atom	X	Y	Z
O	-2.570293	0.180592	0.286425
O	-3.158091	-1.132925	-1.600449
O	-4.819774	-0.93909	1.654859
O	-6.704253	0.239027	0.086856
O	-0.647057	-1.783022	-1.377349
O	1.938523	-2.568777	-1.275063
O	-0.111033	3.574168	0.618301
O	1.340995	0.361044	0.002113
O	-0.241064	2.875406	-2.028012
O	2.243022	1.913171	-1.446215
O	5.130774	1.704928	-1.990677
C	-2.261305	-2.240088	0.422293
C	-3.161219	-1.053781	-0.16293
C	-4.659263	-1.020008	0.238621
C	-0.741705	-1.980352	0.035501
C	-5.351929	0.213396	-0.387088
C	-3.104576	1.358844	-0.316916
C	-4.596332	1.492708	-0.001028
C	-2.356027	-2.322061	1.962809
C	-2.737029	-3.591705	-0.154964
C	0.276728	-3.064079	0.438865
C	-2.3056	2.567317	0.196202
C	1.766536	-2.766964	0.127806
C	-0.777906	2.34375	0.281385
C	2.386247	-1.621014	0.965015
C	-0.160847	1.854282	-1.043213
C	2.61926	-0.256579	0.265603
C	-7.5528	1.099036	-0.661205
C	1.287577	1.413791	-0.867474
C	3.469058	0.64677	1.153662
C	3.182796	-3.062476	-1.757148
C	-0.324255	4.004055	1.955827
C	4.784056	0.879462	0.960672

C	2.766982	1.304102	2.316843
C	5.701829	0.2865	-0.090928
C	6.927721	-0.370975	0.576034
C	6.161996	1.383665	-1.064232
C	6.568536	-1.568245	1.448225
H	-5.177943	-1.923777	-0.100101
H	-0.448544	-1.048319	0.521615
H	-5.361067	0.101811	-1.478963
H	-2.96884	1.299381	-1.403659
H	-5.011491	2.361386	-0.523678
H	-4.745996	1.68122	1.07006
H	-2.119898	-1.361015	2.431252
H	-3.350288	-2.63803	2.291122
H	-1.67449	-3.068663	2.379481
H	-2.190375	-4.430621	0.289208
H	-3.797649	-3.763191	0.055425
H	-2.598568	-3.646348	-1.239396
H	0.200327	-3.292788	1.505447
H	0.028275	-3.979214	-0.113678
H	-2.659653	2.811911	1.205602
H	-2.529945	3.452026	-0.412918
H	2.290737	-3.689889	0.417397
H	-2.262369	-1.387708	-1.881018
H	-0.568645	1.611948	1.071652
H	-5.767642	-0.794608	1.82446
H	1.787872	-1.490355	1.875479
H	3.366335	-1.98478	1.304313
H	0.291462	-1.645488	-1.598137
H	-0.690914	0.98748	-1.446388
H	3.105007	-0.418078	-0.699779
H	-8.57356	0.97796	-0.288201
H	-7.264574	2.144966	-0.529345
H	-7.538356	0.83163	-1.722025
H	0.175182	3.670114	-1.649244
H	3.276495	-2.785011	-2.81039
H	4.02156	-2.630636	-1.204217
H	3.208277	-4.15366	-1.681493
H	-0.126106	3.196333	2.666572
H	-1.341827	4.38175	2.083588
H	0.368244	4.824732	2.162415
H	5.282905	1.560961	1.650842
H	3.459594	1.85471	2.962615
H	2.019526	2.017199	1.960453
H	2.268554	0.557615	2.942448
H	5.182932	-0.485164	-0.667413
H	7.47598	0.363328	1.179953
H	7.619781	-0.715865	-0.202536
H	6.463995	2.299848	-0.54444
H	7.017194	1.041129	-1.65612
H	7.479241	-2.036141	1.835597
H	5.955929	-1.274131	2.305701
H	6.020592	-2.321084	0.872778
H	4.337567	1.962825	-1.488087

Cartesian coordinates for conformer 1-3 after optimization at the PCM/B3LYP/6-31G* level of theory. Number of imaginary frequencies = 0. SCF Energy (PCM/mPW1PW91/6-31+G**) = -1884.87249243.

Atom	X	Y	Z
O	2.635007	-0.185356	0.378375
O	2.902106	1.063391	-1.610687
O	5.326112	0.712424	1.106663
O	6.599081	-0.781177	-0.77758
O	1.018146	3.987868	0.795944
O	-1.273622	2.201082	-1.352756
O	0.027448	-3.417256	1.314676

O	-1.553118	-0.636985	0.199748
O	0.024208	-3.403254	-1.443787
O	-2.426229	-2.196161	-1.255846
O	-7.231503	-1.762787	-1.184772
C	2.797831	2.288256	0.53629
C	3.269925	0.959444	-0.226283
C	4.811284	0.72272	-0.224728
C	1.328292	2.744844	0.145486
C	5.181879	-0.610005	-0.898336
C	2.922321	-1.447956	-0.23615
C	4.421119	-1.74619	-0.221061
C	2.869654	2.1207	2.076035
C	3.741811	3.465793	0.159198
C	0.189012	1.770355	0.512222
C	2.159458	-2.554389	0.511913
C	-1.202435	2.296934	0.073455
C	0.644267	-2.30684	0.640403
C	-2.375442	1.556757	0.760614
C	-0.047252	-2.178889	-0.729281
C	-2.739914	0.174207	0.186439
C	7.129482	-1.766091	-1.653856
C	-1.492941	-1.719677	-0.625044
C	-3.849715	-0.476218	0.99956
C	-2.254743	3.054608	-1.927322
C	0.131682	-3.350706	2.730786
C	-5.144937	-0.499416	0.623181
C	-3.441226	-1.132878	2.29591
C	-5.766504	0.126325	-0.60911
C	-6.989556	0.989372	-0.23599
C	-6.143068	-0.96219	-1.628627
C	-6.62289	2.213392	0.595351
H	5.329127	1.520405	-0.769881
H	1.284654	2.948205	-0.931209
H	4.916243	-0.558384	-1.962055
H	2.583109	-1.420528	-1.277554
H	4.618696	-2.698512	-0.725426
H	4.784071	-1.864163	0.808159
H	2.315009	1.241887	2.418891
H	3.896731	2.025345	2.436305
H	2.451285	2.991243	2.593625
H	3.732703	3.647151	-0.920944
H	3.457545	4.397933	0.658751
H	4.775609	3.27867	0.465725
H	0.384055	0.815304	0.023544
H	0.175574	1.618936	1.596821
H	2.576673	-2.642045	1.522684
H	2.338855	-3.526099	0.034542
H	-1.277717	3.351858	0.366296
H	1.934021	1.110277	-1.658535
H	0.481852	-1.397014	1.230074
H	6.262834	0.45355	1.046436
H	-2.144719	1.481104	1.830507
H	-3.268209	2.192315	0.689366
H	1.384723	4.70536	0.251886
H	0.442109	-1.419975	-1.347397
H	-3.035204	0.297353	-0.86036
H	8.218314	-1.76003	-1.553107
H	6.768773	-2.7629	-1.387564
H	6.877394	-1.53622	-2.693406
H	-0.350951	-4.09265	-0.867522
H	-2.216501	2.934478	-3.013329
H	-3.258553	2.786697	-1.587212
H	-2.040955	4.100358	-1.686999
H	-0.413119	-4.200011	3.151968
H	-0.32202	-2.429013	3.107496
H	1.173423	-3.418334	3.05358

H	-5.853095	-1.009918	1.276035
H	-2.790545	-1.991536	2.104045
H	-2.906713	-0.426602	2.938021
H	-4.302776	-1.498349	2.864983
H	-5.045723	0.787688	-1.101146
H	-7.730679	0.396355	0.314187
H	-7.480124	1.334152	-1.154858
H	-6.448599	-0.495277	-2.571137
H	-5.293737	-1.6181	-1.849935
H	-5.889919	2.836489	0.07277
H	-7.513803	2.822354	0.779218
H	-6.206128	1.930673	1.566656
H	-6.919053	-2.288347	-0.429162

Cartesian coordinates for conformer 1-4 after optimization at the PCM/B3LYP/6-31G* level of theory. Number of imaginary frequencies = 0. SCF Energy (PCM/mPW1PW91/6-31+G**) = -1884.87381522.

Atom	X	Y	Z
O	-2.565775	0.110461	0.446439
O	-2.615241	-1.289214	-1.454046
O	-5.1458	-1.050456	1.236785
O	-6.589947	0.216852	-0.634586
O	-0.459906	-1.175442	1.659502
O	1.940138	-2.423897	-1.780051
O	-0.195308	3.644529	0.923547
O	1.384606	0.492449	-0.006559
O	-0.155995	3.225064	-1.755124
O	2.334495	2.216931	-1.212173
O	5.190409	1.79533	-1.909958
C	-2.419392	-2.334492	0.724436
C	-3.07065	-1.127771	-0.099227
C	-4.632563	-1.073547	-0.096239
C	-0.835964	-2.259307	0.808703
C	-5.167141	0.190436	-0.804847
C	-2.981519	1.285024	-0.261609
C	-4.502327	1.433787	-0.208181
C	-2.913318	-2.376874	2.196504
C	-2.809522	-3.688179	0.0802
C	-0.060955	-2.142682	-0.514395
C	-2.287679	2.515754	0.345866
C	1.44111	-2.510889	-0.434973
C	-0.752038	2.40275	0.454907
C	2.321071	-1.682547	0.528241
C	-0.080474	2.097076	-0.896849
C	2.626203	-0.233279	0.093279
C	-7.252739	1.127055	-1.501301
C	1.364177	1.652431	-0.727341
C	3.554751	0.441992	1.093643
C	3.101288	-3.20955	-2.013053
C	-0.360654	3.859305	2.31866
C	4.882346	0.59386	0.908633
C	2.917119	0.966361	2.357746
C	5.730564	0.095539	-0.245347
C	6.933055	-0.714508	0.282562
C	6.229406	1.285711	-1.081543
C	6.528016	-1.991957	1.008475
H	-5.051091	-1.95713	-0.591106
H	-0.486584	-3.16894	1.31412
H	-4.945973	0.129435	-1.876872
H	-2.681338	1.19894	-1.311762
H	-4.81056	2.333003	-0.75272
H	-4.843087	1.567716	0.826445
H	-3.932442	-2.766792	2.277897
H	-2.292685	-3.050176	2.800562
H	-2.878708	-1.389052	2.667477

H	-2.503671	-3.757442	-0.967577
H	-2.342301	-4.524603	0.613092
H	-3.892011	-3.849888	0.116418
H	-0.501209	-2.815694	-1.259449
H	-0.158383	-1.137054	-0.932941
H	-2.688136	2.675149	1.354646
H	-2.552943	3.415539	-0.22381
H	1.487351	-3.56075	-0.114357
H	-3.313989	-1.04312	-2.077889
H	-0.500351	1.622254	1.178702
H	-6.107145	-0.910225	1.170171
H	1.87527	-1.702522	1.52698
H	3.282021	-2.203163	0.63274
H	-0.92667	-0.387693	1.333959
H	-0.570082	1.271432	-1.420205
H	3.070764	-0.253257	-0.906778
H	-8.330689	0.999684	-1.368032
H	-6.999617	2.160399	-1.251182
H	-7.00659	0.917538	-2.546456
H	0.222927	3.978997	-1.269028
H	3.355945	-3.133129	-3.073635
H	3.949859	-2.839864	-1.432318
H	2.910128	-4.260855	-1.778424
H	0.044951	3.021254	2.892918
H	-1.412483	4.0171	2.569162
H	0.191937	4.76294	2.590376
H	5.444442	1.117923	1.682323
H	3.658122	1.348902	3.068013
H	2.360154	0.174819	2.868162
H	2.229745	1.786822	2.131481
H	5.147045	-0.562089	-0.897573
H	7.545288	-0.098289	0.953183
H	7.576815	-0.994492	-0.560794
H	6.611042	2.100741	-0.456532
H	7.03806	0.974763	-1.751181
H	5.956993	-1.777424	1.916749
H	5.921777	-2.634072	0.361874
H	7.419845	-2.554911	1.301888
H	4.441826	2.047439	-1.340612

Cartesian coordinates for conformer 1-5 after optimization at the PCM/B3LYP/6-31G* level of theory. Number of imaginary frequencies = 0. SCF Energy (PCM/mPW1PW91/6-31+G**) = -1884.88262893.

Atom	X	Y	Z
O	-2.600715	0.327194	0.250844
O	-3.188063	-1.055513	-1.585946
O	-4.92687	-0.662507	1.610298
O	-6.716098	0.623416	-0.073985
O	-0.706469	-1.773578	-1.26952
O	1.847438	-2.633441	-1.068004
O	-0.038474	3.653184	0.483015
O	1.318749	0.36767	0.058599
O	-0.129474	2.830632	-2.12881
O	2.30694	1.818001	-1.438512
O	5.216616	1.447389	-1.871026
C	-2.375871	-2.092502	0.509061
C	-3.22247	-0.907501	-0.154087
C	-4.730443	-0.810164	0.20492
C	-0.840052	-1.899685	0.148429
C	-5.361417	0.418443	-0.492856
C	-3.082145	1.492908	-0.41866
C	-4.572946	1.684886	-0.137943
C	-2.509147	-2.096415	2.048698
C	-2.881708	-3.454899	-0.014779
C	0.1325	-2.994581	0.627718

C	-2.255199	2.698046	0.055946
C	1.638871	-2.759744	0.338315
C	-0.737917	2.429752	0.189005
C	2.280008	-1.59708	1.135189
C	-0.107	1.856603	-1.094538
C	2.569407	-0.276073	0.383742
C	-7.638919	-0.264903	-0.690158
C	1.321518	1.378444	-0.861512
C	3.42518	0.639844	1.253341
C	3.082746	-3.192823	-1.498661
C	-0.268985	4.155117	1.792386
C	4.754887	0.806487	1.096973
C	2.711789	1.38916	2.352106
C	5.680382	0.112681	0.114063
C	6.814755	-0.559004	0.91471
C	6.213699	1.131302	-0.904455
C	7.659027	-1.515209	0.081869
H	-5.255388	-1.717579	-0.108615
H	-0.527646	-0.955235	0.597153
H	-5.340208	0.294047	-1.583278
H	-2.92666	1.378228	-1.498208
H	-4.966847	2.54191	-0.697498
H	-4.733944	1.917642	0.923061
H	-2.253762	-1.121332	2.476246
H	-3.520356	-2.36522	2.367099
H	-1.861505	-2.842251	2.517541
H	-2.72151	-3.565566	-1.092025
H	-3.951527	-3.583678	0.178849
H	-2.371854	-4.287961	0.480942
H	-0.133743	-3.926982	0.113665
H	0.024476	-3.168836	1.701724
H	-2.623613	3.002325	1.043748
H	-2.436189	3.559187	-0.599603
H	2.124862	-3.684999	0.682371
H	-2.294203	-1.350183	-1.831567
H	-0.571441	1.730899	1.018437
H	-5.869026	-0.461979	1.749735
H	1.670138	-1.405925	2.027021
H	3.242441	-1.975501	1.509168
H	0.240819	-1.67549	-1.473248
H	-0.656511	0.989062	-1.469076
H	3.074126	-0.495556	-0.560459
H	-7.590605	-0.17785	-1.779819
H	-7.453651	-1.297559	-0.384009
H	-8.645826	0.011884	-0.365704
H	0.303595	3.629207	-1.778074
H	3.208803	-2.967809	-2.561001
H	3.92488	-2.766288	-0.946934
H	3.066381	-4.279688	-1.373241
H	0.445907	4.961766	1.97675
H	-0.115422	3.37736	2.54613
H	-1.275968	4.571898	1.875625
H	5.259077	1.50528	1.765796
H	2.147559	0.701413	2.988881
H	3.405704	1.93375	3.00174
H	2.019864	2.122129	1.929699
H	5.140783	-0.670789	-0.425962
H	6.384524	-1.132736	1.746309
H	7.468737	0.200755	1.361455
H	6.542547	2.060749	-0.426391
H	7.063363	0.730318	-1.462572
H	8.380427	-2.031849	0.723329
H	7.033199	-2.273024	-0.400007
H	8.225008	-0.989773	-0.691868
H	4.428257	1.773698	-1.401873

Cartesian coordinates for conformer 1-6 after optimization at the PCM/B3LYP/6-31G* level of theory. Number of imaginary frequencies = 0. SCF Energy (PCM/mPW1PW91/6-31+G**) = -1884.86864026.

Atom	X	Y	Z
O	2.60838	-0.209563	0.378788
O	2.783212	1.04186	-1.620362
O	5.394889	0.590975	0.952799
O	6.505319	-1.080933	-0.844077
O	1.254703	4.039636	0.760716
O	-1.228031	2.421607	-1.268295
O	-0.180983	-3.259082	1.426725
O	-1.61661	-0.428179	0.334701
O	-0.217123	-3.272257	-1.333606
O	-2.576632	-1.888534	-1.169591
O	-5.637511	-2.475348	-1.318983
C	2.924382	2.235642	0.486411
C	3.286883	0.87734	-0.28397
C	4.814187	0.552444	-0.352212
C	1.472365	2.771087	0.122123
C	5.096873	-0.838838	-0.949712
C	2.806797	-1.503022	-0.203517
C	4.285882	-1.888783	-0.196071
C	3.028035	2.080432	2.025032
C	3.924149	3.352587	0.069231
C	0.285898	1.870365	0.525952
C	1.990656	-2.540634	0.585414
C	-1.08686	2.491902	0.153988
C	0.496057	-2.197407	0.732035
C	-2.274921	1.821122	0.887662
C	-0.205937	-2.039721	-0.629338
C	-2.742719	0.463926	0.323086
C	6.961695	-2.159956	-1.648236
C	-1.62048	-1.491364	-0.517497
C	-3.886494	-0.12422	1.137867
C	-2.183286	3.336868	-1.788068
C	-0.034027	-3.199791	2.839164
C	-5.13089	-0.339426	0.662141
C	-3.573888	-0.494271	2.568373
C	-5.652033	-0.098878	-0.739811
C	-6.462624	1.208873	-0.824077
C	-6.458736	-1.314381	-1.229073
C	-7.733634	1.250099	0.015447
H	5.340046	1.293257	-0.965843
H	1.413107	2.957453	-0.957305
H	4.81925	-0.842374	-2.010478
H	2.455347	-1.484226	-1.241047
H	4.417981	-2.876913	-0.650481
H	4.661566	-1.970314	0.832004
H	2.685903	2.984504	2.541362
H	2.423275	1.24736	2.395883
H	4.055298	1.916105	2.358623
H	3.712872	4.302006	0.572648
H	4.955234	3.105354	0.339033
H	3.887994	3.530039	-1.010994
H	0.399286	0.912282	0.016753
H	0.307677	1.704221	1.607954
H	2.419417	-2.628096	1.591467
H	2.101911	-3.533341	0.130814
H	-1.081264	3.545164	0.461325
H	3.317617	0.525456	-2.241902
H	0.400754	-1.273082	1.313956
H	6.314052	0.281959	0.864826
H	-1.997731	1.727538	1.944515
H	-3.131241	2.507924	0.855077

H	1.644761	4.725072	0.192204
H	0.320673	-1.316911	-1.259988
H	-3.028977	0.627841	-0.718635
H	8.051983	-2.200073	-1.576228
H	6.562267	-3.111868	-1.289415
H	6.690016	-2.004713	-2.696637
H	-0.627347	-3.932499	-0.74702
H	-1.905667	4.365071	-1.537507
H	-2.193122	3.236959	-2.876849
H	-3.186229	3.114351	-1.414334
H	-0.395431	-2.242039	3.225366
H	-0.638836	-3.998437	3.277472
H	1.006077	-3.358088	3.134324
H	-5.86899	-0.75294	1.349868
H	-4.405835	-1.01719	3.052667
H	-2.707381	-1.161012	2.614358
H	-3.362265	0.398242	3.16422
H	-4.809414	0.002845	-1.430721
H	-6.734956	1.389022	-1.871669
H	-5.82147	2.048504	-0.52594
H	-6.872606	-1.123969	-2.225134
H	-7.291007	-1.552102	-0.558862
H	-7.519363	1.1189	1.080103
H	-8.226335	2.220596	-0.105489
H	-8.444959	0.479281	-0.29445
H	-4.794804	-2.220529	-1.734453

Cartesian coordinates for conformer 1-7 after optimization at the PCM/B3LYP/6-31G* level of theory. Number of imaginary frequencies = 0. SCF Energy (PCM/mPW1PW91/6-31+G**) = -1884.87866246.

Atom	X	Y	Z
O	-2.538624	0.419622	-0.90934
O	-4.592305	-0.500482	-1.59529
O	-2.908255	-1.083081	1.603829
O	-4.966839	0.516665	2.477213
O	-1.827089	-3.271024	0.6201
O	1.646266	-2.747926	-0.807094
O	-0.187397	3.862892	0.089373
O	1.345589	0.755161	-0.037977
O	0.347919	3.163572	-2.490129
O	2.593366	1.936517	-1.568654
O	5.341012	0.809495	-1.889636
C	-2.672394	-2.02212	-1.354383
C	-3.414144	-0.722922	-0.789552
C	-3.949574	-0.808779	0.669924
C	-1.425596	-2.567593	-0.557198
C	-4.611333	0.520492	1.089532
C	-3.149215	1.674897	-0.582293
C	-3.628821	1.67283	0.868696
C	-3.69409	-3.187839	-1.472775
C	-2.170222	-1.73417	-2.803329
C	-0.332924	-1.546155	-0.193637
C	-2.185292	2.842487	-0.866956
C	0.953573	-2.224532	0.333211
C	-0.740659	2.619741	-0.374803
C	1.854407	-1.267463	1.150647
C	0.163226	2.134292	-1.52607
C	2.448248	-0.083377	0.363193
C	-6.155997	-0.211447	2.753652
C	1.522898	1.640403	-1.056177
C	3.429811	0.703014	1.218686
C	2.58328	-3.767305	-0.490223
C	-0.539401	4.158366	1.435587
C	4.770359	0.588359	1.123576
C	2.836409	1.665669	2.218414

C	5.566932	-0.353894	0.242216
C	6.615866	-1.115677	1.079593
C	6.266565	0.436972	-0.874709
C	6.001412	-2.026445	2.135777
H	-4.682261	-1.617415	0.754832
H	-0.955367	-3.338638	-1.181504
H	-5.51085	0.709857	0.489569
H	-4.016008	1.827664	-1.238416
H	-4.112939	2.623917	1.121746
H	-2.783469	1.568557	1.56017
H	-3.214136	-4.089394	-1.871717
H	-4.128746	-3.449903	-0.503296
H	-4.518991	-2.94115	-2.149801
H	-1.607795	-2.586647	-3.202968
H	-2.9951	-1.561474	-3.501514
H	-1.512528	-0.861073	-2.845247
H	-0.088813	-0.970252	-1.08996
H	-0.706364	-0.865154	0.575265
H	-2.608185	3.767451	-0.457449
H	-2.168884	2.996439	-1.953986
H	0.669664	-3.05367	0.993509
H	-4.319178	-0.449655	-2.524133
H	-0.727067	1.897118	0.448852
H	-3.274073	-0.934439	2.494528
H	1.257986	-0.892591	1.99275
H	2.678374	-1.847829	1.583088
H	-2.141081	-2.611508	1.262283
H	-0.300916	1.29174	-2.049236
H	2.923785	-0.460409	-0.547894
H	-6.38212	-0.102355	3.817838
H	-6.996481	0.188888	2.17868
H	-6.023239	-1.27512	2.540058
H	0.669033	3.948235	-2.011296
H	2.991923	-4.156389	-1.426784
H	3.410719	-3.369237	0.10176
H	2.096078	-4.589435	0.042292
H	-1.623339	4.23759	1.553443
H	-0.089976	5.11853	1.703219
H	-0.1445	3.391946	2.109327
H	5.380365	1.224767	1.765502
H	3.599021	2.117423	2.862031
H	2.317028	2.481816	1.707121
H	2.124543	1.15356	2.873001
H	4.90823	-1.096564	-0.220168
H	7.300883	-0.410848	1.567707
H	7.227466	-1.735755	0.412124
H	6.757023	1.3416	-0.498046
H	7.031835	-0.177551	-1.360248
H	5.451782	-1.456788	2.890916
H	5.316423	-2.74814	1.680083
H	6.788534	-2.587041	2.650323
H	4.642616	1.351192	-1.481104

Cartesian coordinates for conformer 1-8 after optimization at the PCM/B3LYP/6-31G* level of theory. Number of imaginary frequencies = 0. SCF Energy (PCM/mPW1PW91/6-31+G**) = -1884.87083787.

Atom	X	Y	Z
O	-2.524199	0.158776	0.467477
O	-2.615261	-1.295654	-1.389864
O	-5.084019	-0.982955	1.350168
O	-6.572673	0.231195	-0.520712
O	-0.387918	-1.088539	1.667127
O	2.039756	-2.402167	-1.736854
O	-0.142008	3.706011	0.768589
O	1.419697	0.521333	-0.048835

O	-0.171328	3.162348	-1.890647
O	2.329777	2.18082	-1.372507
O	5.438546	1.771415	-1.752069
C	-2.367377	-2.275583	0.813112
C	-3.039834	-1.094826	-0.030305
C	-4.601087	-1.042186	0.006975
C	-0.782496	-2.196622	0.857409
C	-5.154117	0.201146	-0.723554
C	-2.958562	1.311828	-0.263962
C	-4.477777	1.461342	-0.177521
C	-2.825513	-2.274069	2.297416
C	-2.771143	-3.648349	0.219651
C	-0.037295	-2.120422	-0.486869
C	-2.249877	2.559232	0.289454
C	1.466474	-2.487148	-0.42245
C	-0.712047	2.44633	0.369142
C	2.362553	-1.645317	0.511685
C	-0.071764	2.076488	-0.981658
C	2.664852	-0.197399	0.063708
C	-7.25684	1.116656	-1.396563
C	1.377061	1.641609	-0.82859
C	3.583637	0.484216	1.070287
C	1.843159	-3.568091	-2.524663
C	-0.285911	3.989855	2.153598
C	4.924004	0.566685	0.938014
C	2.921185	1.086229	2.28599
C	5.796271	-0.015581	-0.157934
C	6.904395	-0.905487	0.442366
C	6.43415	1.118321	-0.975236
C	6.370235	-2.147053	1.145388
H	-5.029082	-1.93971	-0.453749
H	-0.420797	-3.091024	1.381225
H	-4.958067	0.110494	-1.798366
H	-2.684607	1.195471	-1.318461
H	-4.800265	2.344748	-0.739406
H	-4.793485	1.623622	0.860882
H	-3.841656	-2.662799	2.415044
H	-2.189048	-2.927934	2.906335
H	-2.781599	-1.272399	2.737344
H	-2.492014	-3.748649	-0.832959
H	-2.289216	-4.467888	0.765322
H	-3.852206	-3.809877	0.288021
H	-0.506455	-2.810393	-1.194135
H	-0.139216	-1.127581	-0.932925
H	-2.627956	2.751114	1.301208
H	-2.526264	3.441004	-0.302657
H	1.542671	-3.524915	-0.068846
H	-3.325144	-1.058649	-2.004589
H	-0.446183	1.698745	1.122169
H	-6.046853	-0.845445	1.301914
H	1.948642	-1.662536	1.524431
H	3.326953	-2.167591	0.577301
H	-0.856683	-0.309216	1.32462
H	-0.572762	1.226881	-1.453097
H	3.12253	-0.221986	-0.930753
H	-8.331175	0.991927	-1.234676
H	-6.999545	2.15672	-1.180933
H	-7.034688	0.878618	-2.441093
H	0.227842	3.936036	-1.454127
H	2.34136	-3.416792	-3.486248
H	2.291376	-4.439543	-2.037891
H	0.781595	-3.745525	-2.713322
H	0.118658	3.176218	2.762822
H	-1.332731	4.17124	2.409489
H	0.279323	4.899648	2.373892
H	5.477658	1.095251	1.714441

H	3.649973	1.442738	3.021823
H	2.290298	0.346812	2.788719
H	2.30008	1.940838	2.001897
H	5.198484	-0.632794	-0.836861
H	7.51646	-0.329323	1.147999
H	7.577301	-1.2342	-0.35964
H	6.919808	1.85905	-0.329903
H	7.182971	0.720076	-1.668309
H	5.750605	-1.887994	2.009229
H	5.773828	-2.757328	0.460133
H	7.202369	-2.760955	1.50492
H	5.875964	2.494305	-2.232568

Cartesian coordinates for conformer 1-9 after optimization at the PCM/B3LYP/6-31G* level of theory. Number of imaginary frequencies = 0. SCF Energy (PCM/mPW1PW91/6-31+G**) = -1884.87835606.

Atom	X	Y	Z
O	2.520826	-0.407811	0.267169
O	3.340251	0.843311	-1.581003
O	4.810643	0.472968	1.748696
O	6.621685	-0.996199	0.244233
O	0.895658	1.702613	-1.467679
O	-1.646775	2.62172	-1.429601
O	-0.102343	-3.717702	0.446013
O	-1.356161	-0.194374	0.023736
O	-0.078216	-2.778858	-2.117362
O	-2.437374	-1.596035	-1.452526
O	-5.240586	-2.270055	-1.369557
C	2.465999	2.040143	0.396855
C	3.260285	0.762346	-0.144704
C	4.727633	0.578148	0.328302
C	0.950349	1.939636	-0.058317
C	5.318415	-0.714752	-0.280354
C	2.963377	-1.628016	-0.327695
C	4.418382	-1.908746	0.055962
C	2.495888	2.116846	1.940361
C	3.104213	3.333211	-0.156722
C	0.042896	3.145242	0.249825
C	2.043913	-2.775602	0.115378
C	-1.468553	2.944076	-0.051166
C	0.537511	-2.449271	0.207875
C	-2.178007	1.938782	0.887682
C	-0.039372	-1.817868	-1.071443
C	-2.559156	0.551016	0.31813
C	7.646838	-0.196365	-0.32972
C	-1.426181	-1.221226	-0.875057
C	-3.397126	-0.219774	1.332681
C	-2.840891	3.166425	-1.978393
C	-1.138415	-3.67497	1.413348
C	-4.689844	-0.568187	1.16345
C	-2.710808	-0.577949	2.630876
C	-5.616509	-0.275453	-0.001405
C	-6.773353	0.638434	0.454315
C	-6.190541	-1.591973	-0.55653
C	-6.314953	2.019176	0.907177
H	5.336451	1.434903	0.024026
H	0.535864	1.064037	0.441964
H	5.384565	-0.630898	-1.372746
H	2.883214	-1.541193	-1.418311
H	4.786737	-2.809575	-0.449797
H	4.493076	-2.116045	1.131932
H	2.149093	1.183109	2.394975
H	3.499677	2.337319	2.314793
H	1.86694	2.923411	2.326885
H	2.623275	4.225287	0.259233

H	4.165007	3.398448	0.105632
H	3.024615	3.395487	-1.246746
H	0.137448	3.4555	1.293801
H	0.376373	3.984663	-0.374138
H	2.348429	-3.12062	1.113613
H	2.206792	-3.64153	-0.540459
H	-1.913972	3.931452	0.14038
H	2.486355	1.177431	-1.905611
H	0.388997	-1.784825	1.068666
H	5.722988	0.211946	1.965047
H	-1.569401	1.826949	1.793618
H	-3.108978	2.422518	1.215938
H	-0.04249	1.647242	-1.723969
H	0.584453	-0.996018	-1.431771
H	-3.103362	0.695547	-0.616689
H	8.605811	-0.529618	0.076551
H	7.670439	-0.319351	-1.41673
H	7.514077	0.857118	-0.070498
H	-0.559841	-3.553655	-1.777326
H	-2.942804	2.803333	-3.004484
H	-3.718585	2.852432	-1.406724
H	-2.780508	4.258727	-2.000845
H	-1.970014	-3.059312	1.066441
H	-0.757878	-3.304968	2.369856
H	-1.508613	-4.693146	1.561716
H	-5.172109	-1.107844	1.979961
H	-3.2695	-1.327939	3.201208
H	-1.713854	-0.988359	2.451775
H	-2.606841	0.309067	3.263588
H	-5.081425	0.226325	-0.812898
H	-7.338657	0.163788	1.266502
H	-7.474761	0.772294	-0.378858
H	-6.510903	-2.270804	0.241664
H	-7.060816	-1.396842	-1.191976
H	-7.183312	2.645526	1.135707
H	-5.700121	1.964602	1.810415
H	-5.736756	2.517222	0.122874
H	-4.377992	-2.249731	-0.919405

Cartesian coordinates for conformer 2-1 after optimization at the PCM/B3LYP/6-31G* level of theory. Number of imaginary frequencies = 0. SCF Energy (PCM/mPW1PW91/6-31+G**) = -2695.11990204.

Atom	X	Y	Z
O	-3.842492	-1.166814	-3.067173
C	-2.864556	-1.299574	-2.132739
C	-3.05342	-2.585766	-1.313669
O	-1.953703	-0.494769	-1.999959
C	-2.883991	-2.30959	0.19094
C	-3.929588	-1.349384	0.789164
C	-2.029819	-3.61084	-1.798465
O	-4.355993	-3.121441	-1.619302
C	-3.65118	-1.154761	2.282107
C	-4.733994	-0.308287	2.928911
C	-6.106152	-0.92088	2.658673
C	-6.308022	-1.169724	1.129995
O	-5.223474	-1.933367	0.579285
O	-6.420951	0.121976	0.513326
C	-6.949494	0.08706	-0.825174
C	-8.414319	-0.378376	-0.761738
C	-8.545501	-1.637025	0.056027
C	-7.556494	-1.991277	0.896385
C	-6.789895	1.509532	-1.423718
C	-5.314938	1.809862	-1.597315
C	-7.492643	1.682608	-2.771259
C	-4.610417	2.637154	-0.807573

C	-3.128941	2.806165	-0.923694
C	-2.555303	2.434619	0.343326
C	-1.386194	3.240785	0.505131
C	-1.815794	4.600336	-0.023922
C	-2.67373	4.233318	-1.218956
C	-0.96097	3.296159	1.975997
C	-0.414003	1.951554	2.456633
C	0.642638	1.426458	1.484987
C	0.054358	1.407859	0.073051
O	-0.318911	2.740171	-0.309069
O	1.057419	0.123892	1.898205
C	2.068552	-0.477756	1.055409
C	1.597769	-0.502347	-0.385008
C	1.091865	0.828564	-0.901487
C	3.449461	0.200512	1.298641
C	4.596791	-0.511909	0.564215
C	6.013857	0.032539	0.869752
C	7.110967	-0.755642	0.093754
C	6.112789	1.532568	0.540604
O	8.386179	-0.189719	0.441367
C	9.48301	-0.678035	-0.338369
C	9.607827	-2.20114	-0.172369
C	8.284064	-2.905864	-0.451951
C	7.129971	-2.280987	0.345719
C	10.772171	-0.017723	0.185338
C	10.803782	1.477183	-0.120007
C	10.544925	1.71194	-1.600833
C	9.27475	0.998799	-2.03638
O	9.326865	-0.395051	-1.735055
H	1.493222	2.117544	1.533107
H	6.936859	-0.583558	-0.977122
H	-3.883181	-0.385341	0.270698
O	-7.097582	-0.032145	3.194945
H	-6.360693	-0.624162	-1.422146
H	-2.719048	2.128659	-1.682459
H	-0.830827	0.759103	0.038868
O	0.546857	0.691142	-2.216286
H	2.143775	-1.506183	1.432612
O	3.726454	0.188324	2.706907
C	7.246033	-2.642015	1.832518
C	-9.79991	-2.437639	-0.117404
C	1.613381	-1.638204	-1.107765
H	-4.429168	-1.959897	-2.985129
H	-1.878507	-1.915017	0.385981
H	-2.969601	-3.270104	0.717463
H	-1.003673	-3.273712	-1.621547
H	-2.17317	-4.573888	-1.295924
H	-2.142094	-3.797935	-2.872933
H	-4.952033	-2.866779	-0.878539
H	-3.627677	-2.129705	2.785751
H	-2.672145	-0.68759	2.430589
H	-4.692153	0.719414	2.545759
H	-4.564658	-0.230497	4.00946
H	-6.174494	-1.868527	3.207437
H	-8.784102	-0.561062	-1.776738
H	-9.042879	0.394552	-0.302734
H	-7.637332	-2.910782	1.470634
H	-7.216598	2.236187	-0.718864
H	-4.798506	1.261889	-2.385014
H	-7.267914	2.661794	-3.20958
H	-7.180501	0.913913	-3.487271
H	-8.580036	1.622436	-2.66025
H	-5.102834	3.166087	0.004147
H	-0.977491	5.245461	-0.303416
H	-2.439182	5.125429	0.709696
H	-2.075231	4.245819	-2.137985

H	-3.496319	4.941062	-1.366971
H	-0.172579	4.049356	2.098398
H	-1.804948	3.584277	2.613275
H	-1.229801	1.224824	2.549635
H	0.013484	2.05021	3.461539
H	1.934765	1.522558	-1.000309
H	3.408275	1.249767	0.994937
H	4.4254	-0.468399	-0.517932
H	4.560302	-1.56783	0.854111
H	6.217101	-0.059724	1.943907
H	5.865025	1.721721	-0.509195
H	5.435994	2.123954	1.16434
H	7.119827	1.919584	0.726242
H	9.921946	-2.442157	0.850475
H	10.370867	-2.599624	-0.852039
H	8.376071	-3.974898	-0.227062
H	8.057612	-2.833237	-1.523478
H	6.202249	-2.726245	-0.03266
H	10.865749	-0.160436	1.268577
H	11.645117	-0.481307	-0.291926
H	10.042543	1.995394	0.475396
H	11.774893	1.897676	0.163662
H	11.393802	1.318674	-2.173685
H	10.472029	2.784077	-1.810626
H	9.152262	1.095423	-3.120007
H	8.390108	1.447885	-1.572491
H	-7.226274	0.651829	2.507422
H	-0.3703	0.351354	-2.122111
H	2.8725	0.347395	3.155199
H	8.095377	-2.145925	2.312331
H	6.341953	-2.356267	2.378852
H	7.369204	-3.722853	1.960192
H	-9.893352	-2.778465	-1.153385
H	-9.814463	-3.321702	0.528542
H	-10.67523	-1.828362	0.129506
H	1.27443	-1.658658	-2.140366
H	1.973343	-2.57628	-0.695916

Cartesian coordinates for conformer 2-2 after optimization at the PCM/B3LYP/6-31G* level of theory. Number of imaginary frequencies = 0. SCF Energy (PCM/mPW1PW91/6-31+G**) = -2695.11695434.

Atom	X	Y	Z
O	6.580572	-1.144935	3.0419
C	5.275763	-1.424323	2.777126
C	5.134654	-2.761574	2.035356
O	4.358001	-0.692582	3.113081
C	4.135998	-2.665392	0.867931
C	4.474635	-1.592163	-0.18404
C	4.692711	-3.821123	3.043279
O	6.444798	-3.147348	1.569888
C	3.501318	-1.690389	-1.363035
C	3.885215	-0.721947	-2.471133
C	5.347968	-0.919902	-2.868353
C	6.266945	-0.86436	-1.608246
O	5.825147	-1.805561	-0.617065
O	6.225219	0.488824	-1.130047
C	7.22074	0.778957	-0.132598
C	8.605136	0.726104	-0.801424
C	8.767931	-0.533593	-1.616128
C	7.684725	-1.257464	-1.955142
C	6.885797	2.167714	0.470334
C	5.538664	2.103802	1.166221
C	7.940692	2.65979	1.462209
C	4.405245	2.608579	0.650904
C	3.049885	2.432382	1.274009

C	2.223281	1.794318	0.282589
C	1.121344	2.655172	-0.025639
C	1.557606	4.051859	0.400767
C	2.382962	3.752597	1.631281
C	0.805868	2.592046	-1.525623
C	0.224393	1.23699	-1.925859
C	-0.949623	0.887913	-1.016642
C	-0.494139	0.970673	0.440485
O	-0.024991	2.290333	0.758702
O	-1.432546	-0.417228	-1.338251
C	-2.559634	-0.856092	-0.539971
C	-2.239089	-0.728721	0.936988
C	-1.687131	0.619392	1.339056
C	-3.85723	-0.132701	-1.007351
C	-5.121362	-0.685544	-0.329393
C	-6.456873	-0.095466	-0.845515
C	-7.682433	-0.725841	-0.118883
C	-6.482539	1.435036	-0.687029
O	-8.870636	-0.125077	-0.661849
C	-10.075026	-0.45761	0.037173
C	-10.291596	-1.979221	0.015383
C	-9.05821	-2.728228	0.510869
C	-7.785522	-2.265699	-0.213921
C	-11.251834	0.218274	-0.691408
C	-11.207397	1.736716	-0.546372
C	-11.087756	2.11645	0.922217
C	-9.924093	1.380005	1.567013
O	-10.044504	-0.03279	1.406264
H	-1.736256	1.627765	-1.211309
H	-7.608201	-0.448128	0.941441
H	4.398063	-0.601863	0.27714
O	5.684298	0.095008	-3.825916
H	7.16541	0.02099	0.659746
H	3.102969	1.776854	2.150004
H	0.309521	0.249538	0.646779
O	-1.276929	0.62797	2.708202
H	-2.667981	-1.916898	-0.801383
O	-3.988556	-0.293122	-2.427599
C	-7.77205	-2.78016	-1.659336
C	10.162378	-0.913325	-2.012138
C	-2.398872	-1.761936	1.78488
H	7.115393	-1.897148	2.685452
H	3.12464	-2.478569	1.250735
H	4.122592	-3.641888	0.364525
H	3.7104	-3.589945	3.468821
H	4.647943	-4.810972	2.575334
H	5.408582	-3.897474	3.870329
H	6.530904	-2.789627	0.656405
H	3.520596	-2.707475	-1.774946
H	2.476611	-1.491417	-1.029952
H	3.717519	0.312035	-2.143283
H	3.237436	-0.864529	-3.344268
H	5.444357	-1.894275	-3.363262
H	9.384519	0.770262	-0.032548
H	8.738626	1.584179	-1.471556
H	7.801914	-2.177403	-2.522002
H	6.821067	2.896716	-0.349122
H	5.500111	1.555531	2.107156
H	7.626196	3.595286	1.939049
H	8.12055	1.923041	2.253177
H	8.892968	2.858141	0.960236
H	4.438762	3.123413	-0.306084
H	0.720864	4.722475	0.618509
H	2.196339	4.518105	-0.358804
H	1.717375	3.606599	2.491239
H	3.082511	4.55402	1.88831

H	0.072601	3.368631	-1.777224
H	1.709852	2.777537	-2.117283
H	0.992733	0.457099	-1.862966
H	-0.100296	1.25427	-2.972832
H	-2.46876	1.383257	1.250289
H	-3.772106	0.940976	-0.819531
H	-5.058421	-0.528279	0.753943
H	-5.131522	-1.76809	-0.49771
H	-6.553232	-0.293873	-1.920214
H	-6.333195	1.724621	0.358581
H	-5.703791	1.912349	-1.289159
H	-7.434595	1.858391	-1.023104
H	-10.514106	-2.311542	-1.006009
H	-11.14773	-2.2538	0.64339
H	-9.203413	-3.807914	0.388116
H	-8.939444	-2.552235	1.587738
H	-6.936599	-2.722328	0.308701
H	-11.2423	-0.036369	-1.757887
H	-12.200804	-0.136437	-0.268819
H	-10.353029	2.13938	-1.103749
H	-12.110883	2.181663	-0.977734
H	-12.017616	1.840473	1.434455
H	-10.960135	3.198771	1.027577
H	-9.908605	1.587298	2.641897
H	-8.966152	1.721316	1.160688
H	5.896055	0.887798	-3.293269
H	-0.70268	1.412009	2.801381
H	-3.082461	-0.250668	-2.792141
H	-8.528754	-2.288973	-2.279066
H	-6.797564	-2.611664	-2.128055
H	-7.959213	-3.859064	-1.688267
H	10.777027	-1.083887	-1.122607
H	10.182837	-1.82788	-2.613965
H	10.619211	-0.113421	-2.603597
H	-2.14915	-1.671305	2.838917
H	-2.778268	-2.723977	1.454631

Cartesian coordinates for conformer 2-3 after optimization at the PCM/B3LYP/6-31G* level of theory. Number of imaginary frequencies = 0. SCF Energy (PCM/mPW1PW91/6-31+G**) = -2695.11636256.

Atom	X	Y	Z
O	-4.040843	-1.234486	-2.928021
C	-3.059658	-1.540501	-2.038608
C	-3.478433	-2.708984	-1.13352
O	-1.985456	-0.957448	-2.001458
C	-3.148168	-2.408354	0.339256
C	-3.903584	-1.203781	0.932123
C	-2.748851	-3.963072	-1.611252
O	-4.886217	-2.944438	-1.332205
C	-3.482969	-1.00288	2.390333
C	-4.296687	0.103244	3.039729
C	-5.787543	-0.189656	2.89016
C	-6.149393	-0.465189	1.395946
O	-5.307648	-1.483869	0.835273
O	-6.013082	0.784067	0.701261
C	-6.631357	0.801127	-0.598809
C	-8.154704	0.686666	-0.418887
C	-8.506287	-0.463631	0.488168
C	-7.564336	-0.990152	1.291663
C	-6.200952	2.116443	-1.300349
C	-4.711721	2.065417	-1.574422
C	-6.938566	2.371004	-2.615831
C	-3.789119	2.756591	-0.885023
C	-2.318703	2.575353	-1.088123
C	-1.762662	2.159369	0.172804

C	-0.433697	2.683284	0.217265
C	-0.576481	4.069725	-0.390947
C	-1.573296	3.839249	-1.509921
C	0.084847	2.729724	1.658091
C	0.336705	1.326989	2.211933
C	1.181608	0.510908	1.234256
C	0.52026	0.543136	-0.144604
O	0.438331	1.899662	-0.606224
O	1.310218	-0.825015	1.722938
C	2.108113	-1.695241	0.884449
C	1.558949	-1.696806	-0.52848
C	1.338847	-0.318781	-1.118247
C	3.618923	-1.335678	1.021009
C	4.53391	-2.362319	0.331233
C	6.055644	-2.068819	0.350546
C	6.44547	-0.676531	-0.213336
C	6.662771	-2.283551	1.746241
O	7.879677	-0.575774	-0.191996
C	8.37989	0.731358	-0.498768
C	7.913265	1.159539	-1.900437
C	6.402894	1.009686	-2.063301
C	5.925773	-0.387351	-1.640198
C	9.918761	0.668119	-0.489105
C	10.464702	0.43899	0.917245
C	9.880524	1.465576	1.876476
C	8.363513	1.481146	1.776222
O	7.929689	1.720132	0.437616
H	2.169928	0.985351	1.192352
H	6.043442	0.082308	0.469748
H	-3.676695	-0.303727	0.349901
O	-6.511041	0.930527	3.421575
H	-6.261746	-0.055886	-1.180028
H	-2.122397	1.777239	-1.814228
H	-0.489863	0.115652	-0.092326
O	0.701497	-0.406532	-2.394899
H	1.970984	-2.688054	1.332427
O	3.942306	-1.269045	2.415452
C	6.367775	-1.438299	-2.667996
C	-9.917744	-0.963965	0.442812
C	1.270296	-2.84677	-1.166278
H	-4.784205	-1.867886	-2.766409
H	-2.068202	-2.248959	0.453245
H	-3.415031	-3.296235	0.928826
H	-1.663428	-3.862315	-1.514212
H	-3.070622	-4.842723	-1.042714
H	-2.974797	-4.170126	-2.664052
H	-5.356908	-2.523801	-0.576982
H	-3.646572	-1.929663	2.955163
H	-2.415418	-0.767958	2.451428
H	-4.050157	1.072076	2.586616
H	-4.037841	0.196117	4.10108
H	-6.029427	-1.065995	3.504464
H	-8.628413	0.540116	-1.395965
H	-8.559026	1.605963	0.022505
H	-7.808269	-1.835365	1.930332
H	-6.405968	2.957591	-0.624064
H	-4.384892	1.369137	-2.346176
H	-6.532523	3.24895	-3.131365
H	-6.855065	1.5142	-3.293998
H	-8.001864	2.564291	-2.441458
H	-4.095513	3.430361	-0.089091
H	0.366407	4.484202	-0.760035
H	-1.013523	4.769514	0.331425
H	-1.050279	3.658834	-2.456955
H	-2.222792	4.706915	-1.666624
H	1.031477	3.283956	1.686125

H	-0.628278	3.246292	2.310812
H	-0.617041	0.818216	2.396463
H	0.837044	1.38476	3.1858
H	2.309527	0.155171	-1.303627
H	3.802137	-0.338973	0.61208
H	4.225948	-2.47296	-0.713937
H	4.375406	-3.343676	0.798223
H	6.531296	-2.834114	-0.278119
H	6.414953	-1.467283	2.430372
H	6.314001	-3.223229	2.186889
H	7.755361	-2.339213	1.689062
H	8.407875	0.545919	-2.663197
H	8.187489	2.204002	-2.093017
H	6.117216	1.219068	-3.100808
H	5.897423	1.762381	-1.444693
H	4.829897	-0.371236	-1.636488
H	10.275614	-0.136351	-1.143334
H	10.329949	1.614232	-0.863963
H	10.208253	-0.572053	1.256357
H	11.558091	0.508336	0.911638
H	10.269575	2.456619	1.612127
H	10.193166	1.250079	2.903531
H	7.962209	2.286846	2.399256
H	7.933833	0.544399	2.146229
H	-6.530615	1.588456	2.697823
H	-0.262651	-0.513005	-2.238731
H	3.149451	-0.91735	2.867093
H	7.45015	-1.598999	-2.656507
H	5.887258	-2.402623	-2.477638
H	6.081937	-1.133274	-3.680605
H	-10.159998	-1.328357	-0.560522
H	-10.084035	-1.785906	1.146932
H	-10.61344	-0.158479	0.698447
H	0.874014	-2.850653	-2.178461
H	1.430901	-3.816093	-0.70366

Cartesian coordinates for conformer 2-4 after optimization at the PCM/B3LYP/6-31G* level of theory. Number of imaginary frequencies = 0. SCF Energy (PCM/mPW1PW91/6-31+G**) = -2695.11566251.

Atom	X	Y	Z
O	4.272031	-0.107904	3.286228
C	3.159731	-0.539437	2.632163
C	3.334639	-1.987911	2.148736
O	2.163294	0.151977	2.482687
C	2.841626	-2.156242	0.701761
C	3.598713	-1.307681	-0.336089
C	2.563533	-2.900997	3.100011
O	4.729388	-2.333049	2.277776
C	3.000318	-1.540796	-1.726438
C	3.800652	-0.805922	-2.787654
C	5.271378	-1.205667	-2.701796
C	5.81018	-1.025289	-1.246589
O	4.977542	-1.704171	-0.294704
O	5.863934	0.388137	-0.998155
C	6.659146	0.751158	0.146158
C	8.129393	0.410592	-0.152276
C	8.264234	-1.005281	-0.648928
C	7.182464	-1.650492	-1.121198
C	6.426354	2.262333	0.411188
C	5.003658	2.466901	0.891002
C	7.385695	2.854124	1.445468
C	4.008975	2.955736	0.132519
C	2.590349	3.019862	0.596013
C	1.793863	2.228146	-0.294736
C	0.442436	2.703549	-0.197543

C	0.497079	4.042919	0.550339
C	1.9664	4.397638	0.527825
C	-0.132653	2.865585	-1.613442
C	-0.346947	1.514463	-2.295249
C	-1.141764	0.592032	-1.374106
C	-0.420996	0.494432	-0.029641
O	-0.346189	1.792677	0.578261
O	-1.284417	-0.687156	-1.989575
C	-2.049121	-1.639265	-1.212232
C	-1.441256	-1.782308	0.168081
C	-1.184021	-0.473651	0.888215
C	-3.563818	-1.271834	-1.246454
C	-4.450541	-2.368805	-0.632159
C	-5.971724	-2.080532	-0.558652
C	-6.337518	-0.7552	0.161127
C	-6.636011	-2.152953	-1.942905
O	-7.771333	-0.658126	0.209164
C	-8.257998	0.608444	0.669151
C	-7.734154	0.891448	2.087444
C	-6.218395	0.731625	2.171897
C	-5.759478	-0.612403	1.587639
C	-9.796006	0.540715	0.715623
C	-10.399091	0.455909	-0.683581
C	-9.854332	1.578377	-1.55454
C	-8.334483	1.589136	-1.51506
O	-7.846143	1.690329	-0.177617
H	-2.130523	1.048607	-1.241728
H	-5.963502	0.071466	-0.455966
H	3.523424	-0.247365	-0.071584
O	5.999575	-0.408275	-3.647757
H	6.315395	0.173878	1.016619
H	2.481146	2.606192	1.605816
H	0.595275	0.099972	-0.156534
O	-0.454982	-0.712252	2.095533
H	-1.933203	-2.580889	-1.764566
O	-3.94605	-1.05666	-2.610461
C	-6.158819	-1.765493	2.51884
C	9.626808	-1.625157	-0.587699
C	-1.131774	-2.993228	0.669572
H	4.915664	-0.859726	3.283788
H	1.773186	-1.922706	0.645708
H	2.963266	-3.213175	0.427464
H	1.490757	-2.683187	3.091134
H	2.709279	-3.954097	2.834461
H	2.921552	-2.785096	4.129808
H	5.131294	-2.231259	1.384941
H	3.014207	-2.612715	-1.961602
H	1.955222	-1.21397	-1.755773
H	3.691441	0.278999	-2.662176
H	3.408934	-1.031039	-3.786749
H	5.361167	-2.255063	-3.009649
H	8.725975	0.537025	0.758033
H	8.530715	1.08233	-0.920833
H	7.270593	-2.680304	-1.45843
H	6.561609	2.808652	-0.532297
H	4.781123	2.132444	1.904057
H	7.118891	3.890664	1.68223
H	7.370748	2.281046	2.379468
H	8.413801	2.865337	1.069651
H	4.205791	3.25705	-0.893066
H	0.156097	3.924964	1.586623
H	-0.122476	4.823572	0.097699
H	2.264311	5.047823	1.355284
H	2.208884	4.901578	-0.41592
H	-1.10387	3.373456	-1.557857
H	0.532406	3.477724	-2.233457

H	0.617483	1.055551	-2.547798
H	-0.875536	1.645895	-3.246665
H	-2.137032	-0.025277	1.191444
H	-3.728043	-0.325519	-0.724758
H	-4.100252	-2.587537	0.382305
H	-4.31164	-3.294968	-1.20591
H	-6.421487	-2.908338	0.006672
H	-6.416429	-1.269691	-2.549199
H	-6.306142	-3.041	-2.492071
H	-7.725457	-2.218281	-1.8471
H	-8.197178	0.200323	2.802234
H	-7.999934	1.909191	2.398138
H	-5.89016	0.834013	3.212883
H	-5.738767	1.54637	1.61414
H	-4.664582	-0.591793	1.5406
H	-10.126183	-0.328334	1.297231
H	-10.191165	1.441682	1.202504
H	-10.157327	-0.513732	-1.135353
H	-11.491276	0.520377	-0.625997
H	-10.231938	2.535186	-1.173594
H	-10.208859	1.468724	-2.58474
H	-7.958528	2.456385	-2.067442
H	-7.920595	0.697315	-1.997115
H	6.168043	0.439936	-3.190342
H	0.199584	0.012768	2.198518
H	-3.168509	-0.668279	-3.058656
H	-7.240777	-1.928541	2.535392
H	-5.686748	-2.703184	2.210363
H	-5.83108	-1.565263	3.544704
H	9.978732	-1.668431	0.447881
H	9.631302	-2.645074	-0.985889
H	10.338371	-1.034207	-1.173211
H	-0.691065	-3.100937	1.656929
H	-1.316671	-3.909503	0.116914

Cartesian coordinates for conformer 2-5 after optimization at the PCM/B3LYP/6-31G* level of theory. Number of imaginary frequencies = 0. SCF Energy (PCM/mPW1PW91/6-31+G**) = -2695.11358314.

Atom	X	Y	Z
O	5.645455	0.452979	2.737085
C	4.323522	0.141381	2.646175
C	4.120226	-1.382166	2.624737
O	3.441996	0.986083	2.608073
C	3.112455	-1.801105	1.541354
C	3.522241	-1.429819	0.104379
C	3.648482	-1.818009	4.01054
O	5.409162	-1.99644	2.411244
C	2.444748	-1.901275	-0.876867
C	2.874414	-1.648431	-2.311831
C	4.234012	-2.292214	-2.571139
C	5.273905	-1.842051	-1.495902
O	4.779291	-2.068032	-0.166648
O	5.543684	-0.452404	-1.738331
C	6.722889	0.038344	-1.07332
C	7.952973	-0.649209	-1.690193
C	7.77	-2.144113	-1.734122
C	6.536014	-2.667328	-1.617401
C	6.740599	1.58102	-1.239486
C	5.601742	2.178348	-0.438214
C	8.052461	2.226327	-0.789059
C	4.4571	2.637143	-0.970399
C	3.30125	3.092561	-0.140735
C	2.172607	2.268113	-0.456414
C	0.994116	2.998208	-0.080048
C	1.429942	4.454153	0.141922

C	2.825083	4.498031	-0.439283
C	-0.046345	2.882336	-1.204292
C	-0.603421	1.463989	-1.314441
C	-1.08481	0.990606	0.055863
C	0.064581	1.124298	1.053614
O	0.480045	2.496272	1.158552
O	-1.528421	-0.359699	-0.043834
C	-2.033519	-0.908058	1.186511
C	-1.000161	-0.778232	2.29322
C	-0.376827	0.597876	2.427976
C	-3.459229	-0.401265	1.580266
C	-4.443605	-0.559579	0.405923
C	-5.904523	-0.136968	0.693682
C	-6.816834	-0.33384	-0.554583
C	-6.480313	-0.913031	1.89122
O	-8.141297	0.09535	-0.195609
C	-9.144626	-0.203386	-1.173142
C	-8.797322	0.477929	-2.506739
C	-7.376062	0.147888	-2.952328
C	-6.35738	0.405613	-1.832263
C	-10.486482	0.358871	-0.667364
C	-10.994509	-0.404879	0.552173
C	-11.020015	-1.897445	0.257962
C	-9.668194	-2.357629	-0.264274
O	-9.267651	-1.61121	-1.412519
H	-1.918551	1.642732	0.335497
H	-6.847058	-1.41094	-0.767716
H	3.64789	-0.344537	0.026554
O	4.644361	-1.933508	-3.899254
H	6.658479	-0.209644	-0.004193
H	3.511252	2.98475	0.930258
H	0.925262	0.522359	0.734486
O	0.747938	0.536694	3.317979
H	-2.153522	-1.97682	0.962302
O	-3.442489	0.96845	1.993187
C	-6.164136	1.912535	-1.617258
C	9.001145	-2.979368	-1.910857
C	-0.663717	-1.835387	3.057535
H	6.13926	-0.403836	2.773584
H	2.132251	-1.364464	1.757948
H	3.004706	-2.893411	1.590339
H	2.68508	-1.368694	4.271541
H	3.552595	-2.908122	4.065474
H	4.374013	-1.528188	4.779761
H	5.468093	-2.202574	1.450372
H	2.273707	-2.978053	-0.751166
H	1.494154	-1.394871	-0.678403
H	2.9181	-0.569843	-2.510492
H	2.130632	-2.04999	-3.010294
H	4.109699	-3.381967	-2.54228
H	8.842507	-0.406911	-1.098095
H	8.116872	-0.294624	-2.715178
H	6.40227	-3.746145	-1.624721
H	6.588018	1.821116	-2.300615
H	5.714178	2.167835	0.64582
H	7.984635	3.319859	-0.821037
H	8.310949	1.935031	0.235201
H	8.88038	1.936826	-1.444121
H	4.312076	2.616478	-2.047281
H	1.461792	4.693209	1.212289
H	0.766528	5.186604	-0.328766
H	3.453809	5.271616	0.010662
H	2.767588	4.680901	-1.519405
H	-0.88459	3.560092	-0.998675
H	0.391399	3.170474	-2.167106
H	0.161878	0.780174	-1.703313

H	-1.428644	1.432228	-2.035767
H	-1.08517	1.294244	2.888074
H	-3.78652	-0.994718	2.441225
H	-4.432327	-1.602794	0.06586
H	-4.069019	0.052524	-0.421409
H	-5.930728	0.923556	0.972593
H	-6.437434	-1.993391	1.71673
H	-5.932695	-0.692583	2.812043
H	-7.523707	-0.642466	2.082251
H	-8.886918	1.566429	-2.406194
H	-9.495791	0.162926	-3.291522
H	-7.122132	0.727133	-3.847922
H	-7.326991	-0.909389	-3.242988
H	-5.399369	-0.007156	-2.169797
H	-10.385954	1.418903	-0.405144
H	-11.242762	0.278176	-1.458865
H	-10.343887	-0.208247	1.412731
H	-11.997828	-0.056944	0.821672
H	-11.786099	-2.093954	-0.502097
H	-11.293493	-2.459398	1.156976
H	-9.727913	-3.40956	-0.561702
H	-8.899439	-2.287225	0.512593
H	5.040067	-1.043092	-3.811802
H	1.414288	1.177205	2.988918
H	-4.27618	1.141649	2.46506
H	-7.048741	2.383785	-1.177816
H	-5.31741	2.109932	-0.952413
H	-5.949522	2.414868	-2.566617
H	9.693483	-2.817185	-1.078551
H	8.768821	-4.048735	-1.949628
H	9.508569	-2.713328	-2.843538
H	0.077081	-1.746096	3.8473
H	-1.120632	-2.810906	2.925372

Cartesian coordinates for conformer 2-6 after optimization at the PCM/B3LYP/6-31G* level of theory. Number of imaginary frequencies = 0. SCF Energy (PCM/mPW1PW91/6-31+G**) = -2695.11187732.

Atom	X	Y	Z
O	7.2314	-1.74115	-1.36383
C	5.923099	-1.954025	-1.670854
C	5.519748	-1.187581	-2.93902
O	5.195607	-2.679828	-1.011454
C	4.156894	-0.490438	-2.77853
C	4.07139	0.497779	-1.599732
C	5.491543	-2.175528	-4.104069
O	6.562575	-0.232195	-3.226381
C	2.731655	1.240154	-1.636064
C	2.668339	2.308314	-0.555108
C	3.882283	3.232647	-0.647658
C	5.20137	2.39882	-0.662314
O	5.16763	1.416149	-1.709607
O	5.324516	1.804001	0.638555
C	6.613882	1.214568	0.883424
C	7.655573	2.344222	0.957171
C	7.519856	3.286972	-0.213378
C	6.395424	3.279162	-0.953726
C	6.509176	0.392524	2.194011
C	5.51195	-0.735422	2.003258
C	7.849395	-0.191477	2.642918
C	4.2618	-0.719946	2.495071
C	3.230224	-1.771007	2.198885
C	2.105771	-1.087799	1.614447
C	0.956151	-1.29412	2.44382
C	1.502906	-1.667769	3.81675
C	2.725429	-2.477203	3.449071

C	0.110003	-0.016056	2.499745
C	-0.563916	0.275373	1.160283
C	-1.323112	-0.959534	0.683198
C	-0.365436	-2.149897	0.657182
O	0.189629	-2.405513	1.958124
O	-1.865457	-0.70999	-0.612258
C	-2.618948	-1.805409	-1.167168
C	-1.801432	-3.088634	-1.150809
C	-1.132043	-3.387247	0.171991
C	-4.055109	-1.955478	-0.567884
C	-4.819902	-0.619519	-0.63163
C	-6.279189	-0.656428	-0.116437
C	-6.962861	0.74	-0.225131
C	-7.108718	-1.70676	-0.875975
O	-8.30073	0.608597	0.284878
C	-9.128666	1.758313	0.074742
C	-8.505369	2.986054	0.75889
C	-7.050584	3.18198	0.342988
C	-6.235846	1.890572	0.507758
C	-10.501812	1.481438	0.715317
C	-11.267561	0.397499	-0.03793
C	-11.332943	0.740259	-1.519082
C	-9.94068	1.028206	-2.058163
O	-9.294305	2.061809	-1.316413
H	-2.130627	-1.127862	1.403648
H	-7.021571	0.990278	-1.293128
H	4.161682	-0.057637	-0.660146
O	3.830314	4.139418	0.463846
H	6.86195	0.538531	0.054943
H	3.607556	-2.49686	1.470421
H	0.461784	-1.971874	-0.045053
O	-0.219228	-4.486949	0.053385
H	-2.765399	-1.514835	-2.216473
O	-4.021271	-2.407625	0.789385
C	-5.990312	1.595345	1.993438
C	8.674263	4.202017	-0.48822
C	-1.667559	-3.852627	-2.251592
H	7.596392	-1.137438	-2.057391
H	3.360552	-1.237991	-2.671243
H	3.961791	0.067928	-3.704437
H	4.738715	-2.956806	-3.953944
H	5.279044	-1.660782	-5.047817
H	6.465164	-2.664902	-4.226081
H	6.300659	0.605701	-2.780106
H	2.606905	1.730891	-2.609779
H	1.901119	0.535492	-1.515903
H	2.622894	1.839428	0.436369
H	1.744964	2.892183	-0.648854
H	3.799005	3.823739	-1.568149
H	8.662681	1.912724	0.967243
H	7.524399	2.925043	1.878501
H	6.30492	3.940903	-1.811198
H	6.140653	1.053046	2.990907
H	5.827684	-1.566294	1.37268
H	7.722008	-0.842866	3.515213
H	8.314614	-0.78292	1.846199
H	8.548715	0.599905	2.930694
H	3.935408	0.127675	3.092756
H	0.797084	-2.240658	4.425755
H	1.812833	-0.777643	4.377242
H	2.423475	-3.50119	3.195846
H	3.460965	-2.540871	4.2568
H	-0.672247	-0.128943	3.261078
H	0.729856	0.844171	2.778431
H	0.181625	0.571334	0.412402
H	-1.248727	1.12659	1.25396

H	-1.873258	-3.695396	0.916458
H	-4.571423	-2.724623	-1.153028
H	-4.814163	-0.251229	-1.665369
H	-4.264043	0.106225	-0.028489
H	-6.288046	-0.960608	0.937406
H	-7.102687	-1.508457	-1.953157
H	-6.723246	-2.717162	-0.711301
H	-8.150492	-1.716989	-0.539541
H	-8.543609	2.867333	1.848676
H	-9.069665	3.892543	0.508256
H	-6.60407	3.998604	0.922508
H	-7.016432	3.493887	-0.708807
H	-5.260546	2.065426	0.038389
H	-10.384245	1.170959	1.760455
H	-11.107027	2.39725	0.702348
H	-10.771257	-0.571211	0.096363
H	-12.279298	0.299589	0.3709
H	-11.960674	1.63068	-1.647588
H	-11.798092	-0.077068	-2.080009
H	-10.011555	1.365964	-3.09725
H	-9.321396	0.12503	-2.056157
H	4.211373	3.642763	1.215773
H	0.325253	-4.448077	0.862582
H	-4.916209	-2.716878	1.015978
H	-6.908265	1.305083	2.514054
H	-5.266223	0.784424	2.119186
H	-5.577509	2.474477	2.500016
H	9.575219	3.621385	-0.710736
H	8.48111	4.860583	-1.341454
H	8.8729	4.833488	0.38365
H	-1.06806	-4.759262	-2.23808
H	-2.146637	-3.598005	-3.191438

Cartesian coordinates for conformer 2-7 after optimization at the PCM/B3LYP/6-31G* level of theory. Number of imaginary frequencies = 0. SCF Energy (PCM/mPW1PW91/6-31+G**) = -2695.11398409.

Atom	X	Y	Z
O	9.172555	-2.810824	-0.787884
C	9.429619	-2.047489	-1.883711
C	10.655335	-1.150821	-1.660485
O	8.757972	-2.094157	-2.902555
C	10.415667	0.2764	-2.185519
C	9.182822	0.983123	-1.589177
C	11.853655	-1.793919	-2.355759
O	10.944309	-1.143602	-0.247244
C	9.136721	2.439916	-2.062333
C	7.999567	3.195949	-1.39291
C	8.091494	3.053999	0.1253
C	8.195204	1.549464	0.528988
O	9.278224	0.908421	-0.162312
O	6.924567	0.947354	0.215958
C	6.723278	-0.330274	0.839162
C	6.672285	-0.164575	2.369473
C	7.835139	0.653387	2.863471
C	8.523896	1.419232	1.998998
C	5.435032	-0.993741	0.281109
C	4.182022	-0.181864	0.553011
C	5.54552	-1.225814	-1.229175
C	3.131123	-0.642388	1.253106
C	1.840799	0.065331	1.575661
C	0.793272	-0.757879	1.028678
C	0.069186	-0.006292	0.04625
C	0.953909	1.184144	-0.308364
C	1.621694	1.465677	1.017388
C	-0.227392	-0.881013	-1.178645

C	-1.239234	-1.980741	-0.858529
C	-2.475531	-1.371376	-0.204309
C	-2.041594	-0.546342	1.007404
O	-1.144138	0.506193	0.619752
O	-3.382465	-2.409759	0.1685
C	-4.601791	-1.948303	0.801107
C	-4.277242	-1.041824	1.973134
C	-3.295061	0.060188	1.651566
C	-5.558382	-1.341739	-0.267483
C	-6.943429	-0.998515	0.304204
C	-7.984226	-0.514449	-0.735159
C	-9.358575	-0.205003	-0.069981
C	-7.475231	0.725809	-1.490737
O	-10.258305	0.228842	-1.104163
C	-11.508718	0.736793	-0.626442
C	-12.241605	-0.346774	0.180673
C	-11.350599	-0.931615	1.272243
C	-9.990658	-1.377563	0.714856
C	-12.370415	1.109258	-1.847619
C	-11.801012	2.312572	-2.593399
C	-11.56858	3.463005	-1.625027
C	-10.739721	2.997371	-0.438394
O	-11.343631	1.883225	0.217579
H	-2.948864	-0.724778	-0.954078
H	-9.200939	0.631632	0.624363
H	8.274612	0.461343	-1.912187
O	6.938064	3.687618	0.697447
H	7.57066	-0.977578	0.576093
H	1.729062	0.077578	2.665881
H	-1.54505	-1.175829	1.759209
O	-2.925468	0.790549	2.823751
H	-5.070376	-2.870095	1.169744
O	-5.721088	-2.290409	-1.332343
C	-10.145913	-2.649672	-0.128955
C	8.15543	0.573442	4.324849
C	-4.799112	-1.254849	3.195422
H	9.861604	-2.591878	-0.112468
H	10.322604	0.266312	-3.278819
H	11.304071	0.872859	-1.936287
H	11.703397	-1.86448	-3.438204
H	12.768941	-1.222272	-2.16537
H	12.031028	-2.806302	-1.973747
H	10.447713	-0.383455	0.133293
H	10.078989	2.94176	-1.807793
H	9.025486	2.48498	-3.15144
H	7.032774	2.820377	-1.751746
H	8.028716	4.255892	-1.672166
H	8.978862	3.599608	0.469561
H	6.676815	-1.156364	2.836366
H	5.757589	0.350784	2.68404
H	9.374299	1.998474	2.350444
H	5.338341	-1.978717	0.757738
H	4.17255	0.819706	0.130196
H	4.652465	-1.73144	-1.614181
H	5.661581	-0.286574	-1.781368
H	6.405699	-1.861758	-1.456874
H	3.179534	-1.655609	1.652029
H	0.393275	2.047335	-0.679666
H	1.713009	0.910237	-1.050251
H	0.920316	2.00782	1.664933
H	2.521266	2.08066	0.937755
H	-0.640582	-0.257034	-1.981136
H	0.693203	-1.344335	-1.552424
H	-0.792389	-2.730635	-0.193492
H	-1.521218	-2.518615	-1.771365
H	-3.75914	0.786442	0.973704

H	-5.105253	-0.449926	-0.708672
H	-6.841487	-0.236594	1.086263
H	-7.328243	-1.90442	0.785445
H	-8.134637	-1.294368	-1.492094
H	-7.243073	1.540751	-0.79704
H	-6.573796	0.499927	-2.067996
H	-8.216735	1.092118	-2.207924
H	-12.558019	-1.159785	-0.484058
H	-13.144906	0.06646	0.645705
H	-11.861809	-1.768618	1.762105
H	-11.186877	-0.172314	2.047589
H	-9.358479	-1.625106	1.575638
H	-12.441548	0.263231	-2.541673
H	-13.386857	1.361859	-1.519347
H	-10.855382	2.038464	-3.076286
H	-12.489375	2.622217	-3.387512
H	-12.540388	3.823817	-1.266734
H	-11.072725	4.296209	-2.133872
H	-10.661371	3.806476	0.294953
H	-9.719593	2.742036	-0.744345
H	6.23306	3.010425	0.675736
H	-2.128952	1.295084	2.570822
H	-4.853176	-2.726709	-1.443985
H	-10.679128	-2.46085	-1.065833
H	-9.170125	-3.07591	-0.38168
H	-10.696942	-3.41716	0.425132
H	8.41391	-0.453685	4.601552
H	9.000127	1.215719	4.595019
H	7.292079	0.887939	4.9199
H	-4.547829	-0.614123	4.036788
H	-5.485554	-2.072413	3.393238

Cartesian coordinates for conformer 2-8 after optimization at the PCM/B3LYP/6-31G* level of theory. Number of imaginary frequencies = 0. SCF Energy (PCM/mPW1PW91/6-31+G**) = -2695.1141137.

Atom	X	Y	Z
O	4.555871	1.097959	3.00948
C	3.326929	0.572181	2.763137
C	3.333348	-0.954941	2.930695
O	2.351414	1.252936	2.481703
C	2.632717	-1.639819	1.74347
C	3.357314	-1.50525	0.390585
C	2.618973	-1.281338	4.242088
O	4.699599	-1.387024	3.078986
C	2.523883	-2.187249	-0.699889
C	3.275751	-2.239035	-2.018626
C	4.640792	-2.892899	-1.816506
C	5.420724	-2.17851	-0.669111
O	4.640835	-2.128622	0.534199
O	5.754732	-0.869151	-1.156502
C	6.730344	-0.179016	-0.353255
C	8.072321	-0.926059	-0.457069
C	7.896185	-2.40612	-0.236066
C	6.669791	-2.952882	-0.317122
C	6.815705	1.288975	-0.862642
C	5.438873	1.919119	-0.744285
C	7.925281	2.084786	-0.168204
C	5.045183	2.925627	0.054307
C	3.625341	3.385043	0.221
C	2.716324	2.536709	-0.492601
C	1.522672	3.287864	-0.748158
C	1.837958	4.747926	-0.399795
C	3.34783	4.771238	-0.321129
C	1.138941	3.12245	-2.227369
C	0.670933	1.699478	-2.534895

C	-0.404043	1.279207	-1.534519
C	0.143876	1.456946	-0.118497
O	0.46591	2.838466	0.1077
O	-0.78468	-0.072827	-1.786409
C	-1.816541	-0.574643	-0.904625
C	-1.39079	-0.406	0.539508
C	-0.907374	0.984797	0.898722
C	-3.196258	0.046948	-1.273573
C	-4.359836	-0.589373	-0.495895
C	-5.771339	-0.104434	-0.909373
C	-6.886635	-0.813295	-0.084338
C	-5.896991	1.422098	-0.759601
O	-8.154495	-0.307533	-0.536008
C	-9.272715	-0.718158	0.258181
C	-9.377954	-2.251642	0.263496
C	-8.058078	-2.902772	0.665258
C	-6.883182	-2.358248	-0.160451
C	-10.549457	-0.138648	-0.379487
C	-10.605347	1.380695	-0.248534
C	-10.399097	1.786336	1.203425
C	-9.137715	1.144518	1.758943
O	-9.166446	-0.275198	1.617841
H	-1.265082	1.939836	-1.694483
H	-6.750414	-0.517916	0.964899
H	3.499734	-0.446394	0.147933
O	5.345298	-2.835063	-3.065556
H	6.387839	-0.172321	0.691516
H	3.36403	3.337468	1.284742
H	1.044552	0.845803	0.029049
O	-0.380045	0.994849	2.228871
H	-1.86922	-1.643918	-1.148058
O	-3.425054	-0.139701	-2.678172
C	-6.944899	-2.888133	-1.598917
C	9.129041	-3.202395	0.066447
C	-1.420791	-1.441193	1.400523
H	5.150479	0.341785	3.240041
H	1.611892	-1.253457	1.646422
H	2.557534	-2.711333	1.975392
H	1.570188	-0.965853	4.221602
H	2.657033	-2.35637	4.450455
H	3.105227	-0.780729	5.087689
H	4.966116	-1.800603	2.227057
H	2.290328	-3.216394	-0.3978
H	1.570621	-1.66441	-0.834245
H	3.392696	-1.227791	-2.428768
H	2.699477	-2.798022	-2.765456
H	4.485348	-3.950142	-1.567666
H	8.771183	-0.526504	0.286238
H	8.513013	-0.781207	-1.45105
H	6.537533	-4.016237	-0.133894
H	7.044311	1.270861	-1.937245
H	4.683064	1.44039	-1.368841
H	7.91681	3.133948	-0.484722
H	7.820853	2.053409	0.921956
H	8.91312	1.687893	-0.423193
H	5.760376	3.422271	0.704174
H	1.40898	5.01648	0.573932
H	1.449597	5.466394	-1.12858
H	3.731868	5.570543	0.319032
H	3.768422	4.897185	-1.326439
H	0.317638	3.808331	-2.470914
H	1.986841	3.36814	-2.877259
H	1.51666	1.001071	-2.493694
H	0.280518	1.6382	-3.557594
H	-1.756012	1.678653	0.904304
H	-3.177919	1.125668	-1.097809

H	-4.224949	-0.415401	0.57828
H	-4.303071	-1.671615	-0.656735
H	-5.936382	-0.322835	-1.971772
H	-5.687632	1.734804	0.268809
H	-5.205344	1.947063	-1.424924
H	-6.901077	1.770785	-1.021927
H	-9.654741	-2.611904	-0.734678
H	-10.159946	-2.58004	0.958919
H	-8.132703	-3.991588	0.561406
H	-7.86899	-2.705498	1.728401
H	-5.964899	-2.745407	0.296666
H	-10.604837	-0.405007	-1.441701
H	-11.433799	-0.556071	0.118934
H	-9.829082	1.837517	-0.874041
H	-11.570145	1.753265	-0.610051
H	-11.263067	1.449771	1.789487
H	-10.343564	2.8763	1.291172
H	-9.053226	1.365748	2.827926
H	-8.242231	1.549436	1.275834
H	5.744877	-1.942793	-3.095045
H	0.488269	1.455139	2.203393
H	-2.556589	-0.033681	-3.113878
H	-7.781444	-2.460891	-2.160494
H	-6.024913	-2.655261	-2.143992
H	-7.054263	-3.978038	-1.605894
H	9.5958	-2.84438	0.989665
H	8.908356	-4.267237	0.193784
H	9.852003	-3.105875	-0.749873
H	-1.110706	-1.324245	2.435772
H	-1.76103	-2.428278	1.102425

Cartesian coordinates for conformer 2-9 after optimization at the PCM/B3LYP/6-31G* level of theory. Number of imaginary frequencies = 0. SCF Energy (PCM/mPW1PW91/6-31+G**) = -2695.11566179.

Atom	X	Y	Z
O	-6.606966	2.095838	1.991192
C	-5.368494	2.43039	1.538177
C	-5.447224	3.123812	0.170319
O	-4.347407	2.207376	2.169435
C	-4.37899	2.59244	-0.802288
C	-4.434697	1.073814	-1.0549
C	-5.285163	4.627272	0.388305
O	-6.776369	2.917704	-0.352035
C	-3.428783	0.687846	-2.143883
C	-3.545889	-0.786446	-2.498687
C	-4.99167	-1.135704	-2.850892
C	-5.957737	-0.659519	-1.72154
O	-5.771398	0.738627	-1.452151
O	-5.680851	-1.476388	-0.573806
C	-6.659438	-1.350342	0.473393
C	-7.98359	-1.952656	-0.027122
C	-8.339934	-1.423032	-1.394344
C	-7.401051	-0.815154	-2.143502
C	-6.096365	-2.069016	1.726716
C	-4.833056	-1.365095	2.187106
C	-7.093037	-2.121016	2.885465
C	-3.593152	-1.836267	1.973501
C	-2.339618	-1.082284	2.313891
C	-1.592809	-0.954004	1.089383
C	-0.327815	-1.606209	1.247635
C	-0.509851	-2.584745	2.40185
C	-1.451515	-1.821208	3.304742
C	0.06111	-2.324898	-0.050586
C	0.393331	-1.336959	-1.1679
C	1.419396	-0.32223	-0.67388

C	0.899434	0.33032	0.607035
O	0.675319	-0.652388	1.630166
O	1.661785	0.649327	-1.692672
C	2.634534	1.664594	-1.338423
C	2.251822	2.318272	-0.024415
C	1.944851	1.343961	1.089232
C	4.071878	1.064207	-1.397024
C	5.164918	2.137064	-1.252396
C	6.633459	1.642406	-1.228319
C	6.926796	0.561722	-0.154001
C	7.103829	1.176622	-2.615573
O	8.330383	0.254362	-0.206372
C	8.714524	-0.870705	0.593183
C	8.358793	-0.615746	2.067805
C	6.900419	-0.195926	2.232419
C	6.53588	0.958893	1.288188
C	10.240791	-1.041099	0.476177
C	10.652366	-1.48918	-0.92329
C	9.858115	-2.721564	-1.330306
C	8.368526	-2.473753	-1.152446
O	8.059122	-2.078482	0.18378
H	2.344402	-0.876063	-0.469138
H	6.3722	-0.342836	-0.433762
H	-4.193963	0.547066	-0.125477
O	-5.06257	-2.554144	-3.058469
H	-6.80247	-0.286459	0.703782
H	-2.573531	-0.076741	2.680047
H	-0.039479	0.871972	0.424567
O	1.46197	2.017144	2.253574
H	2.556436	2.398411	-2.151317
O	4.228808	0.38795	-2.65052
C	7.197773	2.262465	1.755972
C	-9.754523	-1.612316	-1.850757
C	2.15431	3.655478	0.095411
H	-7.256066	2.399	1.308789
H	-3.376147	2.854322	-0.441414
H	-4.526254	3.102239	-1.764234
H	-4.304798	4.871441	0.811068
H	-5.406686	5.173992	-0.553559
H	-6.051739	5.008412	1.073277
H	-6.734148	2.103103	-0.903826
H	-3.621796	1.274909	-3.050982
H	-2.40742	0.914849	-1.818207
H	-3.199136	-1.404833	-1.660651
H	-2.885997	-1.030578	-3.339671
H	-5.246545	-0.644213	-3.798048
H	-8.784939	-1.712754	0.680582
H	-7.904899	-3.044599	-0.09537
H	-7.661393	-0.41231	-3.118954
H	-5.839907	-3.101008	1.450377
H	-4.960618	-0.39167	2.660217
H	-6.630999	-2.548457	3.782822
H	-7.462104	-1.121177	3.140629
H	-7.955315	-2.747882	2.63714
H	-3.466302	-2.790633	1.468212
H	0.42605	-2.843237	2.906157
H	-0.998054	-3.507807	2.067293
H	-0.881087	-1.095643	3.898049
H	-1.996159	-2.463106	4.003901
H	0.944867	-2.950156	0.12917
H	-0.751699	-2.979115	-0.387062
H	-0.513594	-0.820347	-1.504251
H	0.779146	-1.867936	-2.046013
H	2.861814	0.827097	1.395673
H	4.187904	0.304821	-0.619516
H	4.987786	2.709364	-0.335575

H	5.065355	2.853303	-2.079047
H	7.256872	2.519372	-1.0055
H	6.693021	0.198235	-2.879652
H	6.815388	1.894411	-3.390561
H	8.195558	1.089603	-2.645485
H	8.997178	0.177653	2.47518
H	8.536564	-1.517573	2.666384
H	6.707601	0.080767	3.275557
H	6.251868	-1.054149	2.014336
H	5.45082	1.100823	1.349149
H	10.75283	-0.100654	0.712167
H	10.583952	-1.8001	1.191246
H	10.47316	-0.679869	-1.641379
H	11.725586	-1.708226	-0.946909
H	10.162279	-3.562718	-0.695206
H	10.079034	-2.992717	-2.36784
H	7.815328	-3.396118	-1.357438
H	8.00679	-1.71862	-1.858099
H	-5.149067	-2.938888	-2.163169
H	1.040419	1.324851	2.797805
H	3.354114	0.01111	-2.872747
H	8.283804	2.246503	1.621832
H	6.80816	3.123015	1.203967
H	6.990458	2.442506	2.816503
H	-10.444659	-1.099856	-1.172919
H	-9.91693	-1.214443	-2.857905
H	-10.008043	-2.677135	-1.866721
H	1.859863	4.118776	1.03373
H	2.36162	4.324271	-0.734337

Cartesian coordinates for conformer 2-10 after optimization at the PCM/B3LYP/6-31G* level of theory. Number of imaginary frequencies = 0. SCF Energy (PCM/mPW1PW91/6-31+G**) = -2695.11023554.

Atom	X	Y	Z
O	-9.15426	0.851145	0.169704
C	-9.087118	0.073139	-0.943758
C	-9.261861	-1.411594	-0.594655
O	-8.915588	0.525407	-2.065079
C	-8.257491	-2.295067	-1.356944
C	-6.77873	-1.916649	-1.146139
C	-10.69893	-1.814753	-0.918601
O	-9.098377	-1.550394	0.831814
C	-5.872582	-2.953899	-1.81744
C	-4.407836	-2.666839	-1.527026
C	-4.178649	-2.546369	-0.021123
C	-5.180302	-1.526519	0.606277
O	-6.534236	-1.858496	0.263534
O	-4.804276	-0.227247	0.115139
C	-5.374254	0.862016	0.855894
C	-4.8407	0.851387	2.301006
C	-4.955969	-0.518851	2.911974
C	-5.122062	-1.589349	2.115485
C	-5.069428	2.202893	0.133703
C	-3.58385	2.475754	0.017402
C	-5.682716	2.224611	-1.26958
C	-2.966894	3.560317	0.518169
C	-1.488465	3.85984	0.439785
C	-0.792582	2.761031	-0.170286
C	0.106518	2.184574	0.785722
C	-0.364498	2.675975	2.149599
C	-0.866413	4.061502	1.815206
C	0.069066	0.654309	0.691829
C	0.678011	0.154905	-0.617118
C	2.068784	0.756949	-0.800963
C	1.967205	2.278378	-0.697824

O	1.434117	2.68659	0.57433
O	2.591214	0.364273	-2.068719
C	3.911781	0.867358	-2.353709
C	3.965284	2.378782	-2.181886
C	3.364409	2.881524	-0.888928
C	5.049676	0.092114	-1.615063
C	4.904256	-1.428172	-1.819141
C	6.214238	-2.252518	-1.722273
C	6.950275	-2.367459	-0.356649
C	5.983361	-3.643852	-2.331455
O	7.147212	-1.058068	0.195946
C	8.095337	-1.018521	1.276647
C	7.631956	-1.945122	2.412962
C	7.334822	-3.354177	1.907432
C	6.375269	-3.333605	0.704882
C	8.153879	0.425409	1.810005
C	8.772577	1.37732	0.790322
C	10.114151	0.836023	0.318686
C	9.970427	-0.602259	-0.15345
O	9.40202	-1.431314	0.860549
H	2.691658	0.354673	0.004919
H	7.943444	-2.760248	-0.619958
H	-6.592913	-0.927577	-1.580724
O	-2.814086	-2.15177	0.185352
H	-6.46447	0.731874	0.87365
H	-1.348743	4.749908	-0.183238
H	1.319688	2.680832	-1.48993
O	3.257677	4.312649	-0.887642
H	4.048465	0.648668	-3.42161
O	5.035708	0.37885	-0.215298
C	4.95753	-2.999078	1.171553
C	-4.862982	-0.612768	4.404334
C	4.470039	3.17994	-3.13933
H	-9.30737	0.246801	0.937844
H	-8.477101	-2.276276	-2.431879
H	-8.397783	-3.328157	-1.010268
H	-10.91741	-1.711029	-1.986661
H	-10.890412	-2.851768	-0.620758
H	-11.413874	-1.192885	-0.366833
H	-8.142864	-1.731774	0.984578
H	-6.108859	-3.955014	-1.434756
H	-6.043264	-2.968096	-2.899672
H	-4.096106	-1.744394	-2.033412
H	-3.773512	-3.460695	-1.939284
H	-4.314628	-3.537463	0.429533
H	-5.402022	1.58287	2.893885
H	-3.781971	1.132053	2.337297
H	-5.220836	-2.578859	2.555183
H	-5.541658	3.007735	0.712919
H	-3.001489	1.724064	-0.514003
H	-5.500949	3.186927	-1.761643
H	-5.269087	1.438365	-1.910897
H	-6.766238	2.083307	-1.216192
H	-3.563274	4.317381	1.02458
H	0.42784	2.694466	2.904007
H	-1.197195	2.069898	2.526187
H	-0.015619	4.749898	1.737052
H	-1.551337	4.464873	2.567312
H	0.639161	0.222568	1.524091
H	-0.961624	0.289302	0.765616
H	0.035119	0.421191	-1.465687
H	0.737882	-0.939959	-0.617805
H	4.010095	2.632459	-0.040868
H	6.010658	0.441998	-2.011931
H	4.507943	-1.589417	-2.830891
H	4.151924	-1.822146	-1.132192

H	6.922088	-1.73916	-2.390324
H	5.199458	-4.191297	-1.798792
H	5.676021	-3.561067	-3.379801
H	6.900981	-4.241193	-2.306548
H	6.722146	-1.542898	2.875185
H	8.399299	-2.005209	3.194194
H	6.92606	-3.964279	2.721546
H	8.275235	-3.833762	1.606489
H	6.353267	-4.351163	0.29588
H	7.150034	0.784073	2.066417
H	8.765095	0.457833	2.721218
H	8.902371	2.370377	1.234543
H	8.098586	1.494341	-0.067006
H	10.821973	0.87124	1.155906
H	10.518355	1.462591	-0.483223
H	10.956818	-1.010574	-0.396141
H	9.364527	-0.660799	-1.064863
H	-2.80719	-1.181682	0.060609
H	2.674705	4.515971	-0.132004
H	5.834535	-0.060532	0.142259
H	4.224318	-3.269264	0.407465
H	4.694545	-3.574405	2.066202
H	4.837327	-1.938461	1.409009
H	-5.671968	-0.042728	4.872036
H	-4.936064	-1.647354	4.755185
H	-3.906378	-0.208605	4.750327
H	4.488454	4.259529	-3.017477
H	4.875076	2.785272	-4.065527

Cartesian coordinates for conformer 2-11 after optimization at the PCM/B3LYP/6-31G* level of theory. Number of imaginary frequencies = 0. SCF Energy (PCM/mPW1PW91/6-31+G**) = -2695.11328725.

Atom	X	Y	Z
O	4.500734	1.711819	-2.790224
C	3.297192	1.762647	-2.15637
C	3.305214	2.830065	-1.050094
O	2.352203	1.052836	-2.465352
C	2.672017	2.30168	0.247866
C	3.385691	1.083396	0.861723
C	2.554585	4.055804	-1.567178
O	4.674126	3.237861	-0.84021
C	2.665281	0.656727	2.144326
C	3.412589	-0.472742	2.832215
C	4.863838	-0.066596	3.075367
C	5.52684	0.436592	1.753598
O	4.741208	1.467845	1.136896
O	5.670665	-0.710108	0.900917
C	6.568253	-0.505983	-0.206322
C	7.992311	-0.325295	0.34665
C	8.018305	0.70929	1.441685
C	6.872447	1.059962	2.053354
C	6.431553	-1.733199	-1.146182
C	5.059618	-1.71342	-1.789016
C	7.492186	-1.777213	-2.247731
C	4.039095	-2.50791	-1.426752
C	2.66139	-2.366076	-1.986707
C	1.76652	-2.084411	-0.903753
C	0.447346	-2.457552	-1.333132
C	0.621265	-3.315526	-2.595001
C	2.100997	-3.626372	-2.609638
C	-0.24255	-3.239891	-0.205347
C	-0.566136	-2.342725	0.988201
C	-1.322788	-1.10383	0.512297
C	-0.498199	-0.411289	-0.571639
O	-0.301553	-1.290711	-1.691262

O	-1.548508	-0.238482	1.621348
C	-2.281963	0.956812	1.296576
C	-1.593767	1.717866	0.175156
C	-1.207395	0.874893	-1.025048
C	-3.811687	0.725449	1.077177
C	-4.419061	-0.088879	2.235239
C	-5.931247	0.131771	2.499035
C	-6.971951	-0.332494	1.440128
C	-6.288769	-0.420314	3.887304
O	-6.615326	0.190903	0.15279
C	-7.667989	0.104241	-0.823477
C	-8.088274	-1.362899	-1.010755
C	-8.428875	-2.027472	0.320382
C	-7.301467	-1.841072	1.350822
C	-7.124282	0.63663	-2.162796
C	-6.849901	2.136546	-2.10242
C	-8.082489	2.872188	-1.597619
C	-8.571259	2.257971	-0.295404
O	-8.821598	0.859664	-0.438212
H	-2.276082	-1.458903	0.10792
H	-7.904156	0.165701	1.744754
H	3.387281	0.256994	0.142724
O	5.551978	-1.20086	3.624131
H	6.264673	0.400769	-0.748835
H	2.603654	-1.534861	-2.699797
H	0.48524	-0.120017	-0.180545
O	-0.348308	1.630766	-1.892975
H	-2.203486	1.563553	2.209061
O	-4.051937	0.040929	-0.153169
C	-6.11279	-2.735149	0.993966
C	9.349575	1.299086	1.794481
C	-1.332189	3.034858	0.286188
H	5.075532	2.397206	-2.366951
H	1.621251	2.049829	0.072284
H	2.697019	3.114977	0.986299
H	1.506368	3.827942	-1.785557
H	2.590558	4.872415	-0.837362
H	3.014431	4.436954	-2.486731
H	5.002854	2.733294	-0.061497
H	2.606863	1.506002	2.837178
H	1.638943	0.344267	1.924685
H	3.367862	-1.384209	2.222335
H	2.930366	-0.727149	3.783557
H	4.876349	0.729677	3.830259
H	8.662829	-0.017202	-0.463315
H	8.366241	-1.270017	0.76001
H	6.883334	1.823079	2.827371
H	6.525067	-2.647751	-0.544525
H	4.892499	-0.952469	-2.551189
H	7.296022	-2.595015	-2.950692
H	7.515974	-0.841843	-2.818269
H	8.489448	-1.946016	-1.829253
H	4.176695	-3.244294	-0.639252
H	0.348432	-2.747805	-3.493512
H	0.011073	-4.22439	-2.594545
H	2.489158	-3.821228	-3.613402
H	2.298078	-4.50514	-1.983272
H	-1.184952	-3.662675	-0.576346
H	0.389217	-4.069916	0.131674
H	0.354605	-2.047372	1.507345
H	-1.164557	-2.889613	1.726522
H	-2.09181	0.628025	-1.621064
H	-4.294906	1.70775	1.004978
H	-3.895477	0.204554	3.155391
H	-4.206685	-1.150429	2.09064
H	-6.051091	1.223842	2.563223

H	-6.086724	-1.493709	3.95753
H	-5.701077	0.07917	4.665425
H	-7.345893	-0.250986	4.117654
H	-7.274534	-1.927279	-1.482446
H	-8.96111	-1.429179	-1.671614
H	-8.640531	-3.091577	0.163043
H	-9.352295	-1.585851	0.716604
H	-7.690965	-2.185249	2.316753
H	-6.198619	0.11762	-2.438239
H	-7.859749	0.456317	-2.957341
H	-6.570325	2.507312	-3.094745
H	-6.002044	2.333675	-1.435151
H	-8.873958	2.792263	-2.352999
H	-7.86268	3.93587	-1.459522
H	-9.512133	2.730746	0.004309
H	-7.853464	2.426766	0.515321
H	5.79479	-1.749701	2.851547
H	0.288176	0.99983	-2.291936
H	-5.025244	0.040798	-0.259518
H	-5.442127	-2.847721	1.849518
H	-6.447941	-3.744925	0.732078
H	-5.537301	-2.344635	0.150004
H	9.779539	1.812638	0.928592
H	9.272846	2.025238	2.610338
H	10.040578	0.511386	2.111357
H	-0.837359	3.579525	-0.513027
H	-1.611545	3.605285	1.166093

Cartesian coordinates for conformer 2-12 after optimization at the PCM/B3LYP/6-31G* level of theory. Number of imaginary frequencies = 0. SCF Energy (PCM/mPW1PW91/6-31+G**) = -2695.11680744.

Atom	X	Y	Z
O	4.051971	0.102641	3.174372
C	2.965764	-0.44629	2.571658
C	3.185273	-1.936866	2.27438
O	1.942498	0.180639	2.336348
C	2.801634	-2.263265	0.818449
C	3.712463	-1.63277	-0.253937
C	2.328799	-2.738959	3.253875
O	4.559635	-2.25622	2.563029
C	3.203055	-2.022965	-1.645739
C	4.169648	-1.583519	-2.732692
C	5.569446	-2.119197	-2.437953
C	6.010588	-1.716769	-0.996757
O	5.038016	-2.129026	-0.025602
O	6.201149	-0.293142	-1.011541
C	6.874035	0.217012	0.154029
C	8.319364	-0.313935	0.164159
C	8.366963	-1.796851	-0.099928
C	7.291543	-2.42048	-0.614117
C	6.809449	1.771245	0.106509
C	5.355477	2.196103	0.003087
C	7.565659	2.419328	1.27049
C	4.60113	2.827312	0.918546
C	3.12411	3.077685	0.7935
C	2.60398	2.486566	-0.409423
C	1.450497	3.24707	-0.76681
C	1.858701	4.682414	-0.472535
C	2.723058	4.550066	0.768309
C	1.099824	3.03056	-2.242958
C	0.59444	1.609874	-2.499103
C	-0.510854	1.250863	-1.505946
C	-0.002457	1.504188	-0.0854
O	0.342188	2.890228	0.068023
O	-0.885405	-0.114823	-1.694389

C	-1.928856	-0.578883	-0.80478
C	-1.529992	-0.329828	0.636131
C	-1.084305	1.088967	0.923707
C	-3.305589	0.010195	-1.232669
C	-4.47797	-0.584301	-0.435556
C	-5.885126	-0.134123	-0.899684
C	-7.009885	-0.797933	-0.050374
C	-6.021021	1.397798	-0.845241
O	-8.273017	-0.328584	-0.551848
C	-9.401647	-0.696614	0.248293
C	-9.498909	-2.227634	0.345763
C	-8.182166	-2.84482	0.807313
C	-6.996964	-2.344418	-0.031664
C	-10.670853	-0.165017	-0.443614
C	-10.73672	1.359114	-0.406685
C	-10.555764	1.85387	1.020702
C	-9.30021	1.254756	1.634105
O	-9.31954	-0.171174	1.579662
H	-1.365593	1.902311	-1.726052
H	-6.89211	-0.437926	0.980862
H	3.71512	-0.542248	-0.145534
O	6.458376	-1.602105	-3.438919
H	6.341813	-0.13196	1.050365
H	2.629841	2.587261	1.639775
H	0.883821	0.886632	0.109743
O	-0.615243	1.212276	2.26863
H	-1.968704	-1.660058	-0.991479
O	-3.510573	-0.264631	-2.626521
C	-7.033021	-2.961729	-1.435838
C	9.64385	-2.507655	0.231465
C	-1.547015	-1.31848	1.550101
H	4.716754	-0.621811	3.28458
H	1.762083	-1.964976	0.631733
H	2.855653	-3.3544	0.699751
H	1.260801	-2.539523	3.116575
H	2.50225	-3.814328	3.134402
H	2.586686	-2.493328	4.290925
H	5.02261	-2.351395	1.700095
H	3.09321	-3.113442	-1.706882
H	2.213804	-1.586589	-1.825109
H	4.186537	-0.488517	-2.804745
H	3.830869	-1.940682	-3.712364
H	5.550999	-3.211631	-2.537331
H	8.779772	-0.100202	1.13519
H	8.913328	0.186497	-0.610556
H	7.31697	-3.494162	-0.782481
H	7.28404	2.110211	-0.824977
H	4.877804	1.893999	-0.930219
H	7.44141	3.50805	1.265381
H	7.219027	2.038394	2.237368
H	8.640586	2.224516	1.198602
H	5.031602	3.126589	1.870524
H	1.009014	5.352645	-0.311671
H	2.475219	5.085926	-1.284838
H	2.149327	4.795537	1.669791
H	3.576228	5.236551	0.737315
H	0.310974	3.734211	-2.536963
H	1.972535	3.215764	-2.879927
H	1.421392	0.893311	-2.413003
H	0.223552	1.515414	-3.526533
H	-1.944318	1.764032	0.845603
H	-3.295353	1.097927	-1.124954
H	-4.360834	-0.344255	0.627914
H	-4.413183	-1.674095	-0.528878
H	-6.031969	-0.417886	-1.949228
H	-5.829468	1.773832	0.165279

H	-5.32162	1.885725	-1.530516
H	-7.022462	1.72386	-1.143853
H	-9.757764	-2.64995	-0.632814
H	-10.290157	-2.517772	1.047545
H	-8.249575	-3.938376	0.769042
H	-8.011137	-2.581675	1.859087
H	-6.0842	-2.697177	0.4626
H	-10.707732	-0.496085	-1.488394
H	-11.561017	-0.55671	0.065434
H	-9.952768	1.781572	-1.046934
H	-11.697435	1.703154	-0.805438
H	-11.427368	1.548522	1.612714
H	-10.507121	2.947572	1.042483
H	-9.233935	1.541363	2.688747
H	-8.399084	1.634813	1.141163
H	6.704853	-0.708167	-3.127788
H	0.32519	0.926657	2.278586
H	-2.637821	-0.167419	-3.055893
H	-7.862733	-2.574814	-2.035493
H	-6.105852	-2.757086	-1.97987
H	-7.136587	-4.050673	-1.377724
H	9.864337	-2.415289	1.299642
H	9.593437	-3.574729	-0.00903
H	10.475474	-2.076069	-0.334665
H	-1.255605	-1.141926	2.582216
H	-1.860347	-2.327414	1.298994

Cartesian coordinates for conformer 2-13 after optimization at the PCM/B3LYP/6-31G* level of theory. Number of imaginary frequencies = 0. SCF Energy (PCM/mPW1PW91/6-31+G**) = -2695.11522495).

Atom	X	Y	Z
O	-5.059345	-1.82149	2.307302
C	-3.778999	-1.366168	2.278653
C	-3.64939	-0.032142	3.028698
O	-2.865809	-1.971721	1.736081
C	-2.822113	0.978468	2.214951
C	-3.44133	1.369815	0.859641
C	-2.995082	-0.310308	4.380532
O	-4.977384	0.457256	3.300689
C	-2.532724	2.3798	0.154153
C	-3.170641	2.871139	-1.133991
C	-4.562078	3.429988	-0.846399
C	-5.421365	2.393917	-0.053919
O	-4.733647	1.936238	1.120446
O	-5.709767	1.317789	-0.959779
C	-6.766523	0.446489	-0.516114
C	-8.085618	1.238092	-0.511144
C	-7.927543	2.546671	0.218566
C	-6.696386	3.042849	0.437408
C	-6.796768	-0.773699	-1.474399
C	-5.521897	-1.572763	-1.297123
C	-7.995615	-1.695234	-1.243712
C	-4.508266	-1.589832	-2.178285
C	-3.205175	-2.270211	-1.903954
C	-2.178358	-1.264553	-1.970605
C	-0.995432	-1.932019	-2.415979
C	-1.507255	-2.886175	-3.484186
C	-2.827429	-3.358472	-2.905341
C	0.019308	-0.927484	-2.968958
C	0.593047	-0.046019	-1.860248
C	1.09303	-0.903878	-0.698046
C	-0.022214	-1.850309	-0.254758
O	-0.419866	-2.692736	-1.348231
O	1.483098	-0.050747	0.375164
C	2.029223	-0.736573	1.514625

C	1.069232	-1.807616	2.010857
C	0.474731	-2.692481	0.9303
C	3.499159	-1.229768	1.321095
C	4.388538	-0.120582	0.730267
C	5.873718	-0.507912	0.524409
C	6.688881	0.658614	-0.110188
C	6.522075	-0.940962	1.851177
O	8.046419	0.210404	-0.264308
C	8.967343	1.234146	-0.656248
C	8.538689	1.837844	-2.003275
C	7.081845	2.289242	-1.977015
C	6.153557	1.178209	-1.464599
C	10.355367	0.588303	-0.825042
C	10.932906	0.134917	0.512704
C	10.901446	1.283981	1.509663
C	9.50485	1.880134	1.587398
O	9.040589	2.296742	0.303954
H	1.956011	-1.468193	-1.068385
H	6.67849	1.489223	0.608772
H	-3.560809	0.475723	0.237641
O	-5.163849	3.779766	-2.101748
H	-6.538476	0.100618	0.502242
H	-3.191629	-2.688361	-0.890081
H	-0.89177	-1.275193	0.090436
O	-0.578003	-3.502571	1.467349
H	2.083941	0.048104	2.281716
O	3.58793	-2.37219	0.46492
C	6.00769	0.070731	-2.516549
C	-9.1803	3.235909	0.665953
C	0.765419	-1.914815	3.318556
H	-5.587211	-1.165793	2.827995
H	-1.81118	0.584774	2.049042
H	-2.725905	1.892101	2.817762
H	-1.982537	-0.710061	4.266077
H	-2.944701	0.601978	4.985307
H	-3.580602	-1.036654	4.956639
H	-5.178408	1.13483	2.615469
H	-2.361248	3.245051	0.807347
H	-1.555976	1.932924	-0.057628
H	-3.230413	2.053818	-1.864154
H	-2.54631	3.642398	-1.600321
H	-4.448368	4.351489	-0.261615
H	-8.867905	0.641941	-0.028247
H	-8.406053	1.455724	-1.537374
H	-6.577626	3.974949	0.983902
H	-6.841044	-0.405815	-2.508519
H	-5.415014	-2.111779	-0.356121
H	-7.924307	-2.596368	-1.863638
H	-8.061912	-2.01141	-0.196613
H	-8.933627	-1.196829	-1.508463
H	-4.583197	-1.029403	-3.106422
H	-0.827323	-3.718695	-3.688285
H	-1.705372	-2.352495	-4.421452
H	-2.697604	-4.312968	-2.381094
H	-3.579168	-3.524461	-3.684314
H	0.848166	-1.468706	-3.442394
H	-0.443273	-0.286065	-3.727928
H	-0.170284	0.656898	-1.505923
H	1.410056	0.573432	-2.248948
H	1.230399	-3.402338	0.579588
H	3.881731	-1.529924	2.303206
H	4.330271	0.7694	1.369317
H	3.970742	0.153787	-0.243887
H	5.935693	-1.375639	-0.144214
H	6.446878	-0.148041	2.602753
H	6.047441	-1.840916	2.252952

H	7.581339	-1.184649	1.72101
H	8.659251	1.095985	-2.801936
H	9.170583	2.697773	-2.256655
H	6.775784	2.619518	-2.976683
H	6.988339	3.163646	-1.320037
H	5.165366	1.630115	-1.319223
H	10.299386	-0.27582	-1.497701
H	11.049711	1.314522	-1.267125
H	10.35165	-0.709345	0.902722
H	11.961152	-0.217969	0.377415
H	11.605696	2.057158	1.17888
H	11.225899	0.941254	2.497738
H	9.518184	2.763878	2.233636
H	8.796274	1.17098	2.028402
H	-5.530134	2.942398	-2.450913
H	-1.398019	-2.961575	1.464555
H	3.42202	-3.156175	1.019439
H	6.931041	-0.503064	-2.642623
H	5.21442	-0.631154	-2.241247
H	5.736753	0.494117	-3.489698
H	-9.733712	2.602161	1.366286
H	-8.968523	4.185207	1.168952
H	-9.823099	3.449057	-0.194131
H	0.076774	-2.674587	3.679829
H	1.19777	-1.257006	4.066289

Cartesian coordinates for conformer 2-14 after optimization at the PCM/B3LYP/6-31G* level of theory. Number of imaginary frequencies = 0. SCF Energy (PCM/mPW1PW91/6-31+G**) = -2695.11328838.

Atom	X	Y	Z
O	-6.579796	2.885106	-0.076007
C	-5.297697	2.891354	-0.531437
C	-5.23787	2.532041	-2.023312
O	-4.34265	3.167923	0.177076
C	-4.105628	1.534946	-2.328214
C	-4.180287	0.212196	-1.541953
C	-5.053234	3.824294	-2.817334
O	-6.522476	1.991081	-2.397444
C	-3.101489	-0.754209	-2.041539
C	-3.227735	-2.111668	-1.367369
C	-4.649819	-2.651402	-1.518969
C	-5.688884	-1.588733	-1.042996
O	-5.489727	-0.342693	-1.728951
O	-5.522865	-1.460927	0.377335
C	-6.575852	-0.717584	1.016713
C	-7.871864	-1.541475	0.927687
C	-8.108146	-2.033017	-0.479417
C	-7.099285	-2.02527	-1.370856
C	-6.12868	-0.436019	2.474491
C	-4.888307	0.438276	2.460528
C	-7.212781	0.242459	3.31285
C	-3.646938	-0.020167	2.69253
C	-2.404473	0.81162	2.547565
C	-1.566952	0.13276	1.593393
C	-0.328317	-0.210857	2.225664
C	-0.618432	-0.209799	3.722202
C	-1.610173	0.924665	3.840837
C	0.146887	-1.586648	1.742325
C	0.58402	-1.552326	0.279112
C	1.595465	-0.429185	0.067406
C	0.992733	0.880999	0.572297
O	0.655776	0.800651	1.967254
O	1.920911	-0.341987	-1.318425
C	2.882763	0.681747	-1.642939
C	2.441106	2.032316	-1.097886

C	2.007612	2.010445	0.350787
C	4.354839	0.29324	-1.292014
C	4.709809	-1.095314	-1.85768
C	6.211294	-1.33927	-2.159872
C	7.230891	-1.400551	-0.986604
C	6.352182	-2.572681	-3.064725
O	7.074192	-0.24425	-0.151528
C	8.167417	-0.030697	0.758395
C	8.332069	-1.254701	1.67432
C	8.443197	-2.549095	0.873203
C	7.290789	-2.69074	-0.136494
C	7.833519	1.195833	1.628612
C	7.827641	2.483032	0.808817
C	9.128777	2.611024	0.030207
C	9.396968	1.344912	-0.768077
O	9.403951	0.189411	0.070602
H	2.484348	-0.69677	0.648379
H	8.210792	-1.313879	-1.478826
H	-4.027398	0.419501	-0.477681
O	-4.740408	-3.869317	-0.764746
H	-6.709864	0.237343	0.491794
H	-2.641316	1.804951	2.151204
H	0.082891	1.134946	0.00943
O	1.408628	3.257594	0.730526
H	2.843711	0.736479	-2.739479
O	4.556407	0.285724	0.122577
C	5.992409	-3.034399	0.595269
C	-9.487799	-2.515866	-0.809438
C	2.406968	3.129987	-1.877344
H	-7.158008	2.650966	-0.843837
H	-3.13031	2.002203	-2.1419
H	-4.156882	1.296269	-3.399553
H	-4.106426	4.317545	-2.572952
H	-5.077816	3.62759	-3.895054
H	-5.865107	4.531233	-2.60898
H	-6.456189	1.015354	-2.281713
H	-3.206879	-0.898601	-3.124484
H	-2.103597	-0.338269	-1.862697
H	-2.963315	-2.031324	-0.30513
H	-2.510322	-2.820391	-1.798136
H	-4.817221	-2.896495	-2.575157
H	-8.719335	-0.926055	1.24957
H	-7.81506	-2.415486	1.588207
H	-7.273241	-2.35719	-2.391111
H	-5.87624	-1.392655	2.95226
H	-5.028562	1.481537	2.178396
H	-6.831917	0.512104	4.304541
H	-7.576348	1.157341	2.831616
H	-8.06799	-0.42332	3.466162
H	-3.504061	-1.069982	2.93686
H	0.270869	-0.046281	4.338383
H	-1.098764	-1.144571	4.034923
H	-1.072102	1.880709	3.85833
H	-2.219874	0.873067	4.748203
H	1.001598	-1.911896	2.348929
H	-0.649761	-2.331159	1.856034
H	-0.282901	-1.406881	-0.376666
H	1.021972	-2.514391	-0.011957
H	2.872353	1.884337	1.010096
H	5.016686	1.059715	-1.713776
H	4.176817	-1.210005	-2.811455
H	4.320695	-1.873368	-1.196952
H	6.521419	-0.476116	-2.768055
H	5.954189	-3.471879	-2.584125
H	5.805276	-2.428657	-4.003069
H	7.401005	-2.75319	-3.322853

H	7.469931	-1.335872	2.347609
H	9.227897	-1.147732	2.298035
H	8.472383	-3.408127	1.553868
H	9.397038	-2.554831	0.330174
H	7.536125	-3.541392	-0.784268
H	6.854227	1.075714	2.107018
H	8.584424	1.300495	2.422283
H	7.696931	3.347727	1.468619
H	6.979299	2.478179	0.113646
H	9.949737	2.769672	0.740167
H	9.092354	3.48082	-0.633988
H	10.380858	1.411024	-1.243657
H	8.659784	1.218981	-1.569275
H	-4.899234	-3.586003	0.158064
H	0.958501	3.070385	1.576052
H	5.517804	0.142206	0.241515
H	5.247353	-3.427129	-0.101369
H	5.564429	-2.170191	1.110962
H	6.159877	-3.820032	1.340449
H	-10.214911	-1.708235	-0.677494
H	-9.562335	-2.868035	-1.843685
H	-9.765724	-3.346165	-0.152376
H	2.074063	4.087685	-1.485757
H	2.707467	3.105595	-2.91972

Cartesian coordinates for conformer 2-15 after optimization at the PCM/B3LYP/6-31G* level of theory. Number of imaginary frequencies = 0. SCF Energy (PCM/mPW1PW91/6-31+G**) = -2695.10935256.

Atom	X	Y	Z
O	-7.985558	-2.620887	-2.565786
C	-8.160248	-3.372405	-1.44595
C	-9.544837	-3.133607	-0.827548
O	-7.309731	-4.134897	-1.014029
C	-9.465103	-2.999065	0.703987
C	-8.512853	-1.895712	1.204809
C	-10.455682	-4.292676	-1.227004
O	-10.095032	-1.942176	-1.426127
C	-8.63805	-1.748803	2.725004
C	-7.801836	-0.584188	3.232204
C	-8.152789	0.691267	2.468261
C	-8.060157	0.449463	0.928786
O	-8.861128	-0.676404	0.539516
O	-6.666706	0.251178	0.623101
C	-6.361135	0.367065	-0.774909
C	-6.630137	1.806135	-1.252732
C	-7.992854	2.273938	-0.817428
C	-8.631663	1.628547	0.174479
C	-4.893502	-0.068703	-1.033224
C	-3.88007	0.791938	-0.30154
C	-4.673779	-1.52974	-0.627732
C	-2.913709	1.494383	-0.917432
C	-1.854552	2.36896	-0.297959
C	-0.59344	1.819114	-0.722562
C	0.156889	1.417584	0.431619
C	-0.846501	1.343051	1.577736
C	-1.791419	2.466729	1.220953
C	0.833629	0.064595	0.179267
C	1.94757	0.175068	-0.860494
C	2.911109	1.292265	-0.468885
C	2.116336	2.580947	-0.26367
O	1.121673	2.427626	0.763212
O	3.892147	1.452101	-1.491937
C	4.86854	2.479093	-1.232015
C	4.19058	3.801968	-0.905592
C	3.088566	3.702069	0.124664

C	5.973265	2.057784	-0.208973
C	6.61613	0.717768	-0.615355
C	7.764588	0.220143	0.295363
C	8.34651	-1.137103	-0.202805
C	8.887841	1.267456	0.393605
O	9.390265	-1.521951	0.707935
C	10.159879	-2.652078	0.281331
C	9.244292	-3.874258	0.104512
C	8.052518	-3.557597	-0.793739
C	7.321137	-2.286764	-0.33685
C	11.194751	-2.966417	1.377893
C	12.259234	-1.87759	1.477925
C	12.868153	-1.617718	0.108017
C	11.77587	-1.3487	-0.914804
O	10.829247	-2.415257	-0.963851
H	3.389675	0.979784	0.46535
H	8.795605	-0.955275	-1.188682
H	-7.481981	-2.160955	0.943307
O	-7.260956	1.726448	2.906405
H	-7.013908	-0.322507	-1.32685
H	-1.951722	3.370783	-0.731693
H	1.612704	2.882721	-1.193169
O	2.362059	4.934414	0.229632
H	5.385533	2.595801	-2.1942
O	5.452468	1.940426	1.118608
C	6.542488	-2.550941	0.958642
C	-8.563733	3.466128	-1.522258
C	4.534702	4.943503	-1.531323
H	-8.818175	-2.104387	-2.702505
H	-9.16538	-3.954532	1.152427
H	-10.475826	-2.767534	1.067268
H	-10.091489	-5.247555	-0.833541
H	-11.476582	-4.130447	-0.86368
H	-10.521248	-4.381073	-2.317967
H	-9.820992	-1.191454	-0.851185
H	-9.685601	-1.56138	2.993769
H	-8.332403	-2.674436	3.225347
H	-6.733353	-0.810265	3.12386
H	-7.966438	-0.434748	4.30593
H	-9.172377	0.988532	2.743401
H	-6.544772	1.841522	-2.345031
H	-5.898699	2.507561	-0.835604
H	-9.622841	1.954859	0.479944
H	-4.713963	0.00147	-2.114577
H	-3.971124	0.803188	0.781782
H	-3.648577	-1.847895	-0.849483
H	-4.848916	-1.689791	0.441871
H	-5.34825	-2.187128	-1.183708
H	-2.853122	1.447457	-2.004735
H	-0.391899	1.483298	2.5631
H	-1.390027	0.39111	1.571279
H	-1.334173	3.423868	1.503365
H	-2.756026	2.412558	1.731222
H	1.271537	-0.305145	1.115118
H	0.100364	-0.674275	-0.164315
H	1.526272	0.370969	-1.854686
H	2.487354	-0.775588	-0.945342
H	3.507988	3.519225	1.11927
H	6.722766	2.856893	-0.193773
H	6.983932	0.793829	-1.646376
H	5.825786	-0.040387	-0.610866
H	7.387233	0.076813	1.315182
H	9.283795	1.512999	-0.597552
H	8.534276	2.191374	0.860024
H	9.718381	0.910193	1.010981
H	8.867154	-4.204487	1.080057

H	9.802598	-4.711693	-0.33134
H	7.366271	-4.412211	-0.81769
H	8.406102	-3.415476	-1.823062
H	6.592563	-2.038615	-1.117679
H	10.703488	-3.072439	2.352548
H	11.698354	-3.914602	1.149741
H	11.81252	-0.954062	1.865603
H	13.039327	-2.179086	2.185663
H	13.438637	-2.503107	-0.197931
H	13.564893	-0.774213	0.153606
H	12.220078	-1.258822	-1.911502
H	11.262649	-0.404132	-0.705901
H	-6.454546	1.62093	2.363577
H	1.560924	4.70704	0.738612
H	6.210974	1.925272	1.728802
H	7.20596	-2.703778	1.815345
H	5.879311	-1.713628	1.197002
H	5.911292	-3.44023	0.85648
H	-8.681533	3.255845	-2.590093
H	-9.545488	3.745386	-1.125777
H	-7.898986	4.32834	-1.407934
H	4.034132	5.881674	-1.306342
H	5.31772	4.975432	-2.282005

Cartesian coordinates for conformer 2-16 after optimization at the PCM/B3LYP/6-31G* level of theory. Number of imaginary frequencies = 0. SCF Energy (PCM/mPW1PW91/6-31+G**) = -2695.11376038.

Atom	X	Y	Z
O	7.013193	1.104954	2.604145
C	5.866257	0.438971	2.906052
C	6.131453	-1.059869	3.109074
O	4.784518	0.99248	3.023605
C	5.08595	-1.928581	2.384439
C	5.010541	-1.72396	0.858139
C	6.122667	-1.341822	4.611115
O	7.46611	-1.342356	2.640663
C	4.059186	-2.755924	0.241149
C	4.076398	-2.687177	-1.278699
C	5.50971	-2.788586	-1.801067
C	6.415143	-1.733131	-1.095866
O	6.334113	-1.870116	0.330618
O	5.973773	-0.444476	-1.55518
C	6.841928	0.631761	-1.160747
C	8.192289	0.468731	-1.885695
C	8.698215	-0.950217	-1.819457
C	7.870925	-1.941338	-1.44046
C	6.144883	1.977247	-1.505742
C	4.744171	2.009012	-0.914791
C	7.007099	3.184774	-1.120719
C	4.298575	2.736972	0.123794
C	2.900499	2.711348	0.696109
C	2.079719	1.777909	-0.028568
C	0.99991	2.487117	-0.64473
C	1.432105	3.947637	-0.686571
C	2.214207	4.067964	0.60101
C	0.734383	1.929743	-2.049113
C	0.161076	0.513824	-1.996124
C	-1.047645	0.48036	-1.065402
C	-0.642501	1.044493	0.296749
O	-0.176975	2.397987	0.174804
O	-1.53048	-0.859194	-0.953563
C	-2.685149	-1.014251	-0.091828
C	-2.411032	-0.404567	1.269614
C	-1.866415	1.004122	1.220532
C	-3.962075	-0.49111	-0.813824

C	-5.250873	-0.792973	-0.032247
C	-6.565105	-0.411679	-0.75721
C	-7.817527	-0.770255	0.097323
C	-6.584262	1.084929	-1.114996
O	-8.983525	-0.387276	-0.651707
C	-10.211732	-0.473869	0.078891
C	-10.438679	-1.917653	0.554979
C	-9.227114	-2.456067	1.30975
C	-7.928995	-2.255335	0.513964
C	-11.360051	-0.081627	-0.86981
C	-11.309174	1.39941	-1.234393
C	-11.233134	2.244213	0.028733
C	-10.095836	1.766904	0.917917
O	-10.221514	0.380172	1.230182
H	-1.821174	1.110449	-1.522246
H	-7.774576	-0.157078	1.007868
H	4.650661	-0.710745	0.646811
O	5.474295	-2.605848	-3.223842
H	6.995062	0.583119	-0.075173
H	2.953855	2.382464	1.738348
H	0.149393	0.437522	0.757976
O	-1.5007	1.468835	2.521979
H	-2.792472	-2.102261	0.00867
O	-4.049603	-1.11314	-2.104479
C	-7.874081	-3.219211	-0.678514
C	10.130757	-1.179787	-2.195253
C	-2.601028	-1.100766	2.405803
H	7.742701	0.436873	2.602497
H	4.090526	-1.759052	2.814179
H	5.349087	-2.979126	2.570708
H	5.145056	-1.129907	5.057021
H	6.381221	-2.387044	4.814136
H	6.869263	-0.727971	5.128893
H	7.37703	-1.635245	1.705075
H	4.36728	-3.766408	0.539019
H	3.039013	-2.60353	0.611013
H	3.612786	-1.7521	-1.618758
H	3.465557	-3.492288	-1.70412
H	5.881788	-3.801061	-1.600866
H	8.930687	1.142626	-1.437149
H	8.088095	0.738529	-2.943883
H	8.241039	-2.96096	-1.37006
H	5.998027	2.018609	-2.594404
H	4.044912	1.332255	-1.406735
H	6.472653	4.124917	-1.297573
H	7.300284	3.149646	-0.065765
H	7.921466	3.222796	-1.721433
H	4.985778	3.389683	0.657676
H	0.593997	4.649569	-0.727843
H	2.099122	4.141144	-1.535203
H	1.51953	4.18852	1.441556
H	2.897732	4.922589	0.611997
H	0.012859	2.574038	-2.567141
H	1.657708	1.91569	-2.639814
H	0.923607	-0.195315	-1.649461
H	-0.125803	0.17946	-3.000113
H	-2.640748	1.690638	0.858018
H	-3.875122	0.584383	-0.989438
H	-5.220993	-0.285884	0.939494
H	-5.263678	-1.870216	0.167059
H	-6.629127	-0.955125	-1.708102
H	-6.465542	1.704744	-0.219899
H	-5.783506	1.338621	-1.8159
H	-7.522056	1.370031	-1.602465
H	-10.631714	-2.570146	-0.30523
H	-11.316334	-1.971888	1.210532

H	-9.376321	-3.516113	1.546674
H	-9.141016	-1.933111	2.27091
H	-7.10031	-2.510404	1.185181
H	-11.318914	-0.674955	-1.790995
H	-12.324333	-0.279757	-0.384391
H	-10.434663	1.597862	-1.865739
H	-12.19536	1.673156	-1.817554
H	-12.18066	2.150022	0.573261
H	-11.101017	3.300943	-0.226005
H	-10.112623	2.318478	1.863471
H	-9.123017	1.95792	0.452515
H	5.462271	-1.637097	-3.358941
H	-0.929766	2.245549	2.367347
H	-3.131942	-1.191104	-2.43268
H	-8.607108	-2.963291	-1.449805
H	-6.884248	-3.212403	-1.145073
H	-8.068472	-4.247361	-0.354786
H	10.793431	-0.622975	-1.525199
H	10.405925	-2.237939	-2.135068
H	10.311552	-0.844929	-3.22168
H	-2.383873	-0.665571	3.378035
H	-2.9734	-2.120596	2.40021

Cartesian coordinates for conformer 2-17 after optimization at the PCM/B3LYP/6-31G* level of theory. Number of imaginary frequencies = 0. SCF Energy (PCM/mPW1PW91/6-31+G**) = -2695.11188711.

Atom	X	Y	Z
O	4.266202	1.26741	2.909671
C	3.181589	0.468308	2.730975
C	3.56465	-1.016334	2.825911
O	2.052148	0.901676	2.554734
C	2.949876	-1.816653	1.663723
C	3.504671	-1.47351	0.26816
C	3.060748	-1.541027	4.170419
O	5.001934	-1.110685	2.851219
C	2.76934	-2.306092	-0.787756
C	3.397846	-2.137117	-2.160268
C	4.891604	-2.446701	-2.092639
C	5.572178	-1.597855	-0.973821
O	4.907273	-1.771935	0.285314
O	5.541825	-0.23326	-1.421585
C	6.388645	0.648977	-0.661223
C	7.856556	0.251855	-0.900566
C	8.057953	-1.232605	-0.738507
C	6.995138	-2.057074	-0.753956
C	6.07822	2.107945	-1.1045
C	4.605809	2.383286	-0.853608
C	7.020296	3.128173	-0.457465
C	4.052073	3.242272	0.018772
C	2.582923	3.340901	0.312668
C	1.845556	2.319179	-0.371132
C	0.48991	2.766435	-0.498599
C	0.47643	4.249537	-0.107351
C	1.938561	4.634018	-0.140477
C	0.030844	2.5527	-1.949787
C	-0.107801	1.067379	-2.286017
C	-0.960308	0.373114	-1.226032
C	-0.350966	0.640641	0.150104
O	-0.351682	2.052223	0.414148
O	-1.02725	-1.023872	-1.509866
C	-1.832897	-1.782958	-0.57683
C	-1.336328	-1.554314	0.836205
C	-1.168172	-0.098112	1.221622
C	-3.346843	-1.496785	-0.81333
C	-4.257659	-2.44338	-0.012291

C	-5.784829	-2.200705	-0.117409
C	-6.228502	-0.756187	0.236026
C	-6.339108	-2.623729	-1.487264
O	-7.663447	-0.702309	0.159392
C	-8.206779	0.619124	0.266731
C	-7.797525	1.250522	1.608452
C	-6.290064	1.169936	1.834204
C	-5.763659	-0.257142	1.623456
C	-9.742279	0.509141	0.22145
C	-10.235544	0.071736	-1.154793
C	-9.646791	0.973241	-2.229932
C	-8.1348	1.04784	-2.088744
O	-7.751532	1.4823	-0.784205
H	-1.965025	0.809662	-1.287821
H	-5.824012	-0.087276	-0.534047
H	3.369294	-0.405216	0.065725
O	5.453985	-2.184622	-3.386795
H	6.143635	0.542946	0.405374
H	2.430646	3.201468	1.389349
H	0.678856	0.26183	0.199619
O	-0.547353	0.003311	2.50639
H	-1.652898	-2.826989	-0.865281
O	-3.620914	-1.641756	-2.212284
C	-6.211837	-1.168701	2.774291
C	9.466157	-1.715565	-0.567592
C	-1.042006	-2.588408	1.647312
H	5.040661	0.670594	3.059804
H	1.861863	-1.68542	1.662012
H	3.151768	-2.880693	1.849836
H	1.96912	-1.486693	4.243206
H	3.37049	-2.580737	4.324821
H	3.483387	-0.961725	4.999804
H	5.286826	-1.423397	1.963013
H	2.815055	-3.369244	-0.51838
H	1.710934	-2.025876	-0.824848
H	3.234944	-1.116308	-2.529236
H	2.910904	-2.7976	-2.887567
H	5.014784	-3.516806	-1.883953
H	8.499685	0.78698	-0.193045
H	8.164884	0.527317	-1.91656
H	7.136348	-3.125574	-0.612761
H	6.213399	2.176366	-2.192905
H	3.93628	1.753953	-1.441786
H	6.735503	4.152677	-0.721743
H	7.018258	3.041184	0.634574
H	8.049263	2.989223	-0.804289
H	4.680286	3.878707	0.636023
H	0.078701	4.38025	0.907052
H	-0.13124	4.872637	-0.770988
H	2.173317	5.484012	0.506499
H	2.231265	4.88604	-1.167122
H	-0.948348	3.025584	-2.096859
H	0.736405	3.013346	-2.650803
H	0.880699	0.593711	-2.34286
H	-0.559055	0.940207	-3.277123
H	-2.153388	0.371168	1.32439
H	-3.572487	-0.458298	-0.558263
H	-3.981859	-2.397197	1.04671
H	-4.058992	-3.475378	-0.33131
H	-6.26184	-2.885894	0.596971
H	-6.088637	-1.902648	-2.270377
H	-5.952295	-3.60464	-1.782587
H	-7.431385	-2.70297	-1.455779
H	-8.300989	0.733486	2.434387
H	-8.104814	2.302824	1.646172
H	-6.044034	1.529164	2.840443

H	-5.784222	1.844923	1.131892
H	-4.669024	-0.20884	1.649083
H	-10.099612	-0.207662	0.970328
H	-10.189691	1.485311	0.449181
H	-9.942443	-0.968349	-1.342632
H	-11.329846	0.108446	-1.190498
H	-10.069754	1.979017	-2.117208
H	-9.919868	0.608593	-3.225666
H	-7.733884	1.771468	-2.805833
H	-7.669168	0.082354	-2.31246
H	5.626002	-1.221905	-3.403125
H	0.18281	0.657824	2.435968
H	-2.818585	-1.34289	-2.684554
H	-7.288897	-1.361706	2.754219
H	-5.700945	-2.135452	2.733617
H	-5.966865	-0.718399	3.74234
H	9.909906	-1.282009	0.334342
H	9.517284	-2.805475	-0.475352
H	10.073827	-1.423895	-1.430039
H	-0.679587	-2.427898	2.659468
H	-1.161505	-3.620246	1.331098

Cartesian coordinates for conformer 2-18 after optimization at the PCM/B3LYP/6-31G* level of theory. Number of imaginary frequencies = 0. SCF Energy (PCM/mPW1PW91/6-31+G**) = -2695.11122225.

Atom	X	Y	Z
O	8.606049	0.043552	-2.938038
C	8.73168	1.356027	-2.604532
C	9.933412	1.580025	-1.676152
O	7.974383	2.223613	-3.010588
C	9.587615	2.541693	-0.52477
C	8.384331	2.109769	0.335596
C	11.090915	2.12288	-2.5117
O	10.346812	0.293177	-1.172527
C	8.234593	3.052693	1.53423
C	7.131186	2.579261	2.467442
C	7.364394	1.12327	2.866525
C	7.566857	0.234409	1.599134
O	8.609879	0.762505	0.765619
O	6.299713	0.192051	0.915327
C	6.208703	-0.852805	-0.064948
C	6.287261	-2.22726	0.625422
C	7.454361	-2.289752	1.573576
C	8.030659	-1.149873	1.994342
C	4.904754	-0.689576	-0.892336
C	3.648209	-0.786841	-0.04691
C	4.888377	0.649998	-1.635502
C	2.685677	-1.704522	-0.24253
C	1.402368	-1.890527	0.524323
C	0.343648	-1.764808	-0.443765
C	-0.495794	-0.661014	-0.081775
C	0.30456	0.165521	0.919174
C	1.078062	-0.910634	1.644711
C	-0.866732	0.154417	-1.327142
C	-1.800163	-0.621913	-2.255759
C	-2.995269	-1.147121	-1.466052
C	-2.486924	-1.953404	-0.271016
O	-1.672118	-1.141258	0.589073
O	-3.821925	-1.941919	-2.317674
C	-4.999296	-2.491375	-1.674614
C	-4.607026	-3.243408	-0.417134
C	-3.699644	-2.471497	0.512345
C	-6.067824	-1.372506	-1.485902
C	-7.421822	-1.924718	-1.007962
C	-8.554976	-0.895294	-0.766218

C	-8.171384	0.266885	0.187381
C	-9.128563	-0.354906	-2.086169
O	-9.330912	1.099488	0.360079
C	-9.074805	2.323996	1.058376
C	-8.517248	2.024359	2.460196
C	-7.325032	1.072837	2.401638
C	-7.642037	-0.178502	1.569753
C	-10.410259	3.076258	1.208617
C	-10.928712	3.577965	-0.135953
C	-9.84506	4.374585	-0.847758
C	-8.557539	3.569084	-0.921097
O	-8.125636	3.153195	0.374156
H	-3.560721	-0.272282	-1.120788
H	-7.397691	0.863792	-0.311787
H	7.473823	2.140393	-0.27389
O	6.241351	0.696474	3.651099
H	7.055692	-0.74917	-0.75642
H	1.389021	-2.915829	0.911072
H	-1.899097	-2.822342	-0.599112
O	-3.254361	-3.282466	1.60208
H	-5.397299	-3.197338	-2.415285
O	-6.239409	-0.696337	-2.737295
C	-8.619099	-1.090097	2.325113
C	7.90675	-3.648876	2.012243
C	-5.007589	-4.509827	-0.19931
H	9.342785	-0.4422	-2.490848
H	9.397708	3.548597	-0.917385
H	10.468885	2.603829	0.128578
H	10.847649	3.092996	-2.957973
H	11.994595	2.23426	-1.901999
H	11.344339	1.432382	-3.324904
H	9.8546	0.153384	-0.331558
H	9.173953	3.082454	2.10119
H	8.024634	4.073028	1.194536
H	6.152807	2.688154	1.982237
H	7.090702	3.210887	3.36283
H	8.255579	1.079397	3.50522
H	6.374747	-3.006912	-0.140178
H	5.38355	-2.43129	1.210994
H	8.884036	-1.192992	2.666733
H	4.893241	-1.484852	-1.650034
H	3.558367	-0.046909	0.744565
H	3.986712	0.745347	-2.251233
H	4.913972	1.501929	-0.946942
H	5.750901	0.728026	-2.303442
H	2.812618	-2.422395	-1.053001
H	-0.321833	0.752388	1.59777
H	1.003708	0.839956	0.411254
H	0.417095	-1.399138	2.372482
H	1.941424	-0.536834	2.200081
H	-1.375998	1.076981	-1.020856
H	0.033894	0.435998	-1.885031
H	-1.264596	-1.455621	-2.727339
H	-2.142086	0.018623	-3.077146
H	-4.249728	-1.635264	0.959801
H	-5.700038	-0.624187	-0.779065
H	-7.271191	-2.484817	-0.078597
H	-7.783168	-2.654779	-1.744645
H	-9.388433	-1.447216	-0.310288
H	-8.461317	0.373115	-2.555522
H	-9.31127	-1.166583	-2.79793
H	-10.087514	0.146604	-1.915173
H	-9.296151	1.568288	3.083318
H	-8.204699	2.952145	2.954662
H	-7.019251	0.79562	3.417385
H	-6.470266	1.593925	1.951807

H	-6.705258	-0.73368	1.443747
H	-11.1693	2.427407	1.661564
H	-10.27356	3.944396	1.866335
H	-11.232532	2.727955	-0.758792
H	-11.817462	4.201043	0.013165
H	-9.66077	5.298856	-0.286554
H	-10.176471	4.657189	-1.852301
H	-7.761444	4.186945	-1.348962
H	-8.673607	2.697534	-1.573749
H	5.566123	0.412308	3.003631
H	-2.50208	-2.798269	1.992461
H	-5.363671	-0.695866	-3.172912
H	-9.620824	-0.655004	2.395491
H	-8.713474	-2.06198	1.831341
H	-8.261296	-1.281961	3.342495
H	8.2316	-4.23818	1.148911
H	8.74456	-3.594864	2.715224
H	7.087449	-4.17917	2.50813
H	-4.706521	-5.052673	0.693138
H	-5.641504	-5.041774	-0.902265

Cartesian coordinates for conformer 2-19 after optimization at the PCM/B3LYP/6-31G* level of theory. Number of imaginary frequencies = 0. SCF Energy (PCM/mPW1PW91/6-31+G**) = -2695.1148011.

Atom	X	Y	Z
O	4.117371	2.116091	-2.456329
C	3.033843	2.101664	-1.635519
C	3.265395	2.983438	-0.399458
O	2.014214	1.475094	-1.885065
C	2.821472	2.25975	0.883942
C	3.602118	0.968343	1.193779
C	2.485808	4.282853	-0.591066
O	4.662036	3.337622	-0.359698
C	3.076614	0.350843	2.492196
C	3.901153	-0.863302	2.884184
C	5.378389	-0.485942	2.966613
C	5.844676	0.214511	1.650521
O	4.988628	1.318191	1.316236
O	5.847223	-0.791511	0.625962
C	6.568304	-0.414862	-0.561736
C	8.061589	-0.29546	-0.211708
C	8.263236	0.56172	1.010758
C	7.227154	0.801003	1.834838
C	6.283797	-1.493347	-1.640485
C	4.820771	-1.430094	-2.028787
C	7.138228	-1.331322	-2.898806
C	3.896492	-2.329629	-1.653645
C	2.436854	-2.158668	-1.930681
C	1.762716	-2.139319	-0.659733
C	0.463312	-2.690013	-0.888486
C	0.731093	-3.844138	-1.842284
C	1.806549	-3.280899	-2.75159
C	-0.167092	-3.155874	0.426745
C	-0.523347	-1.972437	1.325778
C	-1.334314	-0.935238	0.548155
C	-0.579052	-0.56494	-0.729156
O	-0.377293	-1.733047	-1.541339
O	-1.535497	0.210229	1.374018
C	-2.313067	1.255098	0.760295
C	-1.706358	1.666274	-0.572819
C	-1.362863	0.51304	-1.495932
C	-3.846482	0.958384	0.712148
C	-4.368798	0.524434	2.095015
C	-5.8689	0.804809	2.371379
C	-6.955773	0.039391	1.563266

C	-6.135627	0.690892	3.879951
O	-6.683725	0.156528	0.159474
C	-7.78659	-0.224298	-0.681578
C	-8.181733	-1.682974	-0.397593
C	-8.432266	-1.921117	1.089052
C	-7.254157	-1.4285	1.94753
C	-7.330793	-0.113189	-2.148903
C	-7.089133	1.337861	-2.554772
C	-8.308988	2.183315	-2.219015
C	-8.710115	1.983717	-0.765921
O	-8.935245	0.605166	-0.470069
H	-2.29312	-1.406544	0.309097
H	-7.881039	0.600379	1.761191
H	3.483434	0.259208	0.367109
O	6.124226	-1.683301	3.232
H	6.191904	0.55628	-0.913709
H	2.244521	-1.196849	-2.421257
H	0.399833	-0.136756	-0.473919
O	-0.612975	0.981014	-2.627133
H	-2.198583	2.101478	1.451437
O	-4.137221	-0.066112	-0.239049
C	-6.066468	-2.381496	1.803069
C	9.638726	1.108305	1.243515
C	-1.478442	2.960846	-0.867006
H	4.786188	2.715484	-2.040496
H	1.749803	2.028772	0.830095
H	2.968318	2.95215	1.724406
H	1.408004	4.103363	-0.654714
H	2.676418	4.978128	0.234052
H	2.79657	4.794004	-1.510013
H	5.092178	2.730884	0.285044
H	3.136406	1.086636	3.304452
H	2.024143	0.070332	2.383718
H	3.753452	-1.673199	2.158403
H	3.562126	-1.261238	3.848041
H	5.513691	0.188775	3.821189
H	8.601757	0.142033	-1.05869
H	8.488207	-1.285428	-0.008855
H	7.363439	1.438185	2.705018
H	6.491368	-2.482471	-1.210078
H	4.507763	-0.554698	-2.597253
H	6.834308	-2.042692	-3.67535
H	7.050631	-0.321398	-3.314927
H	8.195175	-1.520751	-2.685853
H	4.184751	-3.190559	-1.056283
H	-0.153525	-4.16283	-2.401501
H	1.141333	-4.707493	-1.304833
H	1.359547	-2.86474	-3.662618
H	2.517362	-4.050618	-3.070558
H	-1.086911	-3.714745	0.213055
H	0.514239	-3.820918	0.969829
H	0.389477	-1.513234	1.723241
H	-1.089842	-2.313959	2.200283
H	-2.277987	0.082113	-1.914
H	-4.356992	1.870337	0.378787
H	-3.802246	1.083077	2.85255
H	-4.139487	-0.530484	2.261397
H	-6.010752	1.865333	2.114386
H	-5.904846	-0.31127	4.254864
H	-5.517756	1.403551	4.437152
H	-7.182096	0.915196	4.112446
H	-7.382088	-2.357421	-0.727561
H	-9.087902	-1.948857	-0.955191
H	-8.627216	-2.984945	1.268932
H	-9.342516	-1.386623	1.390228
H	-7.581426	-1.47064	2.993727

H	-6.409891	-0.68527	-2.312475
H	-8.104805	-0.527195	-2.808031
H	-6.873926	1.396825	-3.627408
H	-6.210467	1.730522	-2.028717
H	-9.139023	1.876792	-2.867302
H	-8.107123	3.241067	-2.417602
H	-9.643763	2.518947	-0.56488
H	-7.952684	2.391369	-0.086867
H	6.241659	-2.109191	2.35902
H	0.324969	1.057072	-2.347119
H	-5.114542	-0.103476	-0.284702
H	-5.346794	-2.228869	2.611281
H	-5.548157	-2.258237	0.848018
H	-6.391793	-3.425191	1.875197
H	9.938025	1.75229	0.410537
H	9.693907	1.701717	2.162124
H	10.361613	0.290726	1.329363
H	-1.045231	3.256053	-1.819012
H	-1.728947	3.760044	-0.176785

Cartesian coordinates for conformer 2-20 after optimization at the PCM/B3LYP/6-31G* level of theory. Number of imaginary frequencies = 0. SCF Energy (PCM/mPW1PW91/6-31+G**) = -2695.10942467.

Atom	X	Y	Z
O	-5.867104	-2.904086	1.8277
C	-5.777568	-1.722541	2.494574
C	-7.138032	-1.303095	3.069065
O	-4.732542	-1.101291	2.608825
C	-7.409314	0.197983	2.8582
C	-7.340189	0.6673	1.392243
C	-7.160189	-1.657177	4.554691
O	-8.150297	-2.108283	2.430475
C	-7.798676	2.125502	1.285569
C	-7.850828	2.57629	-0.166473
C	-8.697973	1.609973	-0.993314
C	-8.211844	0.141775	-0.784635
O	-8.179982	-0.190649	0.61222
O	-6.914862	0.043173	-1.399196
C	-6.440525	-1.30788	-1.528195
C	-7.346485	-2.06273	-2.520688
C	-8.806335	-1.841234	-2.223338
C	-9.177898	-0.835834	-1.411347
C	-4.964188	-1.265623	-2.006629
C	-4.097224	-0.479208	-1.039122
C	-4.367014	-2.665916	-2.171361
C	-3.318796	0.553086	-1.407674
C	-2.430997	1.407027	-0.541804
C	-1.082412	1.135484	-0.964064
C	-0.342551	0.608483	0.14619
C	-1.379173	0.165384	1.173474
C	-2.464857	1.194293	0.965288
C	0.538451	-0.560551	-0.312115
C	1.674681	-0.090337	-1.218956
C	2.441864	1.04114	-0.540373
C	1.457107	2.141803	-0.149262
O	0.445393	1.649439	0.744709
O	3.438904	1.538727	-1.430682
C	4.240594	2.609503	-0.895296
C	3.360631	3.73439	-0.369778
C	2.232994	3.278094	0.528204
C	5.340253	2.134449	0.109495
C	6.185289	0.998253	-0.497989
C	7.340304	0.472581	0.388179
C	8.127021	-0.675416	-0.313098
C	8.303368	1.608892	0.774058

O	9.161648	-1.104624	0.588343
C	10.099418	-2.024127	0.016867
C	9.36839	-3.288494	-0.462991
C	8.196322	-2.945979	-1.377538
C	7.275799	-1.890472	-0.747281
C	11.105723	-2.422637	1.112658
C	12.005664	-1.25411	1.503837
C	12.647852	-0.653204	0.262278
C	11.58687	-0.320628	-0.774702
O	10.797714	-1.463338	-1.102559
H	2.913425	0.609984	0.34912
H	8.600982	-0.245803	-1.206124
H	-6.308089	0.578044	1.034445
O	-8.615057	2.015433	-2.367132
H	-6.484369	-1.792471	-0.544784
H	-2.65505	2.455772	-0.768453
H	0.962429	2.558732	-1.038163
O	1.328647	4.35412	0.814765
H	4.784466	2.989603	-1.770755
O	4.774053	1.683758	1.344137
C	6.467912	-2.500863	0.405729
C	-9.783807	-2.774862	-2.870168
C	3.566182	5.015881	-0.728001
H	-6.808547	-3.202143	1.889019
H	-6.708046	0.796456	3.45328
H	-8.4204	0.405399	3.234795
H	-6.396404	-1.105888	5.113223
H	-8.140349	-1.440082	4.993684
H	-6.979462	-2.728234	4.704642
H	-8.442119	-1.606783	1.635004
H	-8.804059	2.229865	1.7134
H	-7.130088	2.779563	1.856317
H	-6.835502	2.638968	-0.578674
H	-8.263164	3.589839	-0.237258
H	-9.743638	1.710194	-0.67647
H	-7.122803	-3.134417	-2.475824
H	-7.155619	-1.720022	-3.545261
H	-10.229294	-0.688304	-1.177707
H	-4.936293	-0.762956	-2.983199
H	-4.126158	-0.805517	-0.002031
H	-3.302704	-2.612914	-2.428439
H	-4.460774	-3.251018	-1.249481
H	-4.863436	-3.217321	-2.976003
H	-3.300636	0.836794	-2.45943
H	-1.001524	0.166393	2.200438
H	-1.768352	-0.832898	0.942846
H	-2.177274	2.126116	1.469221
H	-3.438634	0.90426	1.365416
H	0.978651	-1.052364	0.564476
H	-0.059661	-1.304794	-0.850853
H	1.279553	0.24757	-2.185496
H	2.352116	-0.922075	-1.445645
H	2.622597	2.952015	1.497869
H	5.967681	3.002936	0.338415
H	6.594124	1.329708	-1.460828
H	5.509353	0.162128	-0.706483
H	6.932136	0.082113	1.328341
H	9.139855	1.240145	1.376472
H	8.715858	2.094069	-0.116963
H	7.802303	2.370807	1.37806
H	8.986017	-3.849908	0.398156
H	10.057867	-3.947954	-1.004012
H	7.634093	-3.855497	-1.619508
H	8.584069	-2.55911	-2.3287
H	6.565543	-1.581461	-1.523258
H	10.580774	-2.780457	2.006361

H	11.744831	-3.23739	0.748728
H	11.417407	-0.488368	2.023783
H	12.779476	-1.593503	2.201236
H	13.349134	-1.383221	-0.160438
H	13.220239	0.242767	0.523841
H	12.068549	0.020303	-1.696959
H	10.939598	0.493259	-0.431059
H	-7.784026	1.622301	-2.701947
H	0.54311	3.920643	1.199214
H	5.49402	1.645854	1.998288
H	7.096639	-2.740527	1.268721
H	5.685484	-1.814874	0.744753
H	5.96919	-3.421564	0.084373
H	-9.595427	-3.804436	-2.549453
H	-10.818849	-2.527916	-2.612271
H	-9.689844	-2.726507	-3.959655
H	2.922851	5.812327	-0.363402
H	4.373981	5.305881	-1.392094

Cartesian coordinates for conformer 2-21 after optimization at the PCM/B3LYP/6-31G* level of theory. Number of imaginary frequencies = 0. SCF Energy (PCM/mPW1PW91/6-31+G**) = -2695.11591533.

Atom	X	Y	Z
O	3.725895	-1.577694	-0.469904
C	4.523392	-2.284791	-1.314659
C	5.232798	-3.431554	-0.577689
O	4.632379	-2.022538	-2.502274
C	6.695782	-3.59146	-1.0309
C	7.548769	-2.317522	-0.895535
C	4.445248	-4.717438	-0.822913
O	5.154537	-3.155062	0.835798
C	9.010612	-2.614752	-1.24262
C	9.877042	-1.381812	-1.029688
C	9.683632	-0.828978	0.382148
C	8.166748	-0.63317	0.694806
O	7.437197	-1.847415	0.45366
O	7.697233	0.440959	-0.142872
C	6.404184	0.930708	0.248824
C	6.516184	1.619855	1.620339
C	7.227159	0.726027	2.605508
C	7.961369	-0.309624	2.158509
C	5.851957	1.839928	-0.880426
C	4.387087	2.153457	-0.648458
C	6.745054	3.057384	-1.155725
C	3.804101	3.360288	-0.536906
C	2.332381	3.620455	-0.310438
C	1.626414	2.381646	-0.138301
C	0.701108	2.214046	-1.219467
C	1.146353	3.185001	-2.307314
C	1.682801	4.335846	-1.486938
C	0.713507	0.761327	-1.712643
C	0.130108	-0.193416	-0.671874
C	-1.240838	0.305912	-0.226534
C	-1.113843	1.753141	0.250483
O	-0.616956	2.601674	-0.797265
O	-1.749149	-0.541062	0.805528
C	-3.055666	-0.164498	1.306341
C	-3.05962	1.293016	1.726248
C	-2.501198	2.239081	0.689116
C	-4.154927	-0.566918	0.279275
C	-5.577841	-0.362453	0.824071
C	-6.715388	-0.867077	-0.097278
C	-8.117309	-0.631507	0.540286
C	-6.647077	-0.193715	-1.479353
O	-9.105399	-1.132842	-0.376016

C	-10.455961	-0.815526	-0.022378
C	-10.787855	-1.400079	1.360058
C	-9.757715	-0.985674	2.406258
C	-8.324328	-1.269876	1.933699
C	-11.386734	-1.460219	-1.066528
C	-11.249264	-0.793455	-2.432365
C	-11.419816	0.71241	-2.29617
C	-10.488738	1.256433	-1.224373
O	-10.687879	0.598534	0.026049
H	-1.900979	0.251584	-1.101366
H	-8.251978	0.454716	0.633482
H	7.158513	-1.552007	-1.576387
O	10.417693	0.400044	0.478636
H	5.7293	0.068863	0.338369
H	2.225268	4.206665	0.608922
H	-0.44021	1.828032	1.115717
O	-2.403779	3.572594	1.195087
H	-3.1901	-0.800926	2.190802
O	-3.984963	-1.95139	-0.059005
C	-8.041134	-2.777717	1.950802
C	7.073508	1.055615	4.058906
C	-3.486805	1.6752	2.944211
H	3.81937	-1.981802	0.428467
H	6.732162	-3.931706	-2.073481
H	7.151838	-4.375716	-0.411321
H	4.428665	-4.985006	-1.884693
H	4.874113	-5.552302	-0.25755
H	3.40719	-4.611388	-0.486338
H	5.962508	-2.640565	1.063207
H	9.388738	-3.418245	-0.597696
H	9.096966	-2.956869	-2.279846
H	9.628872	-0.614039	-1.773625
H	10.93363	-1.624387	-1.194943
H	10.124906	-1.539538	1.092239
H	5.514859	1.86589	1.990683
H	7.091083	2.549705	1.551266
H	8.456437	-0.9681	2.867961
H	5.888459	1.242756	-1.803493
H	3.753962	1.26715	-0.581011
H	6.395634	3.599985	-2.041561
H	6.756663	3.761773	-0.318221
H	7.778142	2.751362	-1.351487
H	4.410685	4.260456	-0.60331
H	0.335606	3.495347	-2.973384
H	1.957352	2.761298	-2.911523
H	0.847452	4.950441	-1.129037
H	2.358609	4.98786	-2.048574
H	0.113211	0.681549	-2.62771
H	1.734303	0.442548	-1.952585
H	0.801283	-0.274946	0.192513
H	0.047894	-1.205609	-1.084888
H	-3.17785	2.288756	-0.172271
H	-4.024505	0.000463	-0.646012
H	-5.740894	0.699942	1.04096
H	-5.639348	-0.898895	1.777489
H	-6.592635	-1.942893	-0.273858
H	-6.70429	0.896248	-1.388954
H	-5.719477	-0.445949	-2.001419
H	-7.46499	-0.523033	-2.128149
H	-10.810797	-2.495317	1.308322
H	-11.779564	-1.066863	1.689376
H	-9.962013	-1.497989	3.353712
H	-9.861146	0.088519	2.606602
H	-7.649763	-0.7971	2.657259
H	-11.167255	-2.529483	-1.170525
H	-12.430726	-1.360504	-0.742702

H	-10.264048	-1.017301	-2.858971
H	-11.997721	-1.196302	-3.123643
H	-12.458127	0.925358	-2.013762
H	-11.228965	1.206513	-3.254421
H	-10.689403	2.321514	-1.069702
H	-9.440524	1.168622	-1.529332
H	9.826286	1.08602	0.109637
H	-1.817918	4.041185	0.570463
H	-3.020445	-2.109626	-0.082742
H	-8.602263	-3.311779	1.177599
H	-6.977826	-2.980552	1.789727
H	-8.306481	-3.210682	2.921273
H	6.020388	0.998428	4.352223
H	7.636783	0.36683	4.697124
H	7.436481	2.069007	4.258045
H	-3.468978	2.718895	3.24745
H	-3.861112	0.962152	3.672359

Cartesian coordinates for conformer 2-22 after optimization at the PCM/B3LYP/6-31G* level of theory. Number of imaginary frequencies = 0. SCF Energy (PCM/mPW1PW91/6-31+G**) = -2695.11168272.

Atom	X	Y	Z
O	5.518957	3.00194	-1.639122
C	5.112388	3.062725	-0.343055
C	6.236318	3.573366	0.569561
O	3.988303	2.748837	0.016441
C	6.322409	2.765738	1.877849
C	6.50431	1.247311	1.688998
C	5.98459	5.052072	0.857677
O	7.471634	3.49447	-0.171098
C	6.758501	0.576506	3.043011
C	7.056418	-0.905298	2.87022
C	8.192579	-1.106135	1.868123
C	7.890738	-0.343504	0.540316
O	7.607866	1.039816	0.800974
O	6.784556	-1.019346	-0.083748
C	6.550135	-0.606777	-1.440989
C	7.747981	-1.040244	-2.308496
C	9.061751	-0.678657	-1.666701
C	9.099899	-0.34587	-0.364286
C	5.220959	-1.246358	-1.925543
C	4.061847	-0.845411	-1.030085
C	4.882294	-0.858672	-3.367724
C	3.240005	-1.72839	-0.436507
C	2.074193	-1.449996	0.474487
C	0.900862	-1.885061	-0.236161
C	0.031697	-0.76225	-0.435234
C	0.874068	0.481685	-0.172615
C	1.81236	-0.012802	0.902323
C	-0.532349	-0.776302	-1.861917
C	-1.503832	-1.936648	-2.075981
C	-2.563789	-1.931603	-0.978886
C	-1.870462	-1.938543	0.383304
O	-1.026342	-0.78676	0.537624
O	-3.421274	-3.063599	-1.133539
C	-4.477991	-3.160288	-0.145653
C	-3.895643	-3.087289	1.253106
C	-2.949321	-1.930215	1.473268
C	-5.594966	-2.120855	-0.464227
C	-6.849523	-2.319016	0.403813
C	-8.002126	-1.300662	0.21237
C	-7.578085	0.183081	0.376019
C	-8.741219	-1.515098	-1.118323

O	-8.754412	0.998286	0.23727
C	-8.488286	2.404325	0.167541
C	-7.76085	2.86717	1.441382
C	-6.533132	2.008554	1.73411
C	-6.873761	0.511264	1.712491
C	-9.837128	3.141757	0.074126
C	-10.530418	2.881971	-1.260242
C	-9.578507	3.182413	-2.408794
C	-8.266024	2.439327	-2.216886
O	-7.668825	2.747996	-0.957701
H	-3.148502	-1.011077	-1.10237
H	-6.898604	0.426812	-0.450267
H	5.597805	0.83045	1.235466
O	8.349432	-2.516642	1.656448
H	6.451391	0.485597	-1.465461
H	2.188675	-2.081422	1.363222
H	-1.261982	-2.844293	0.515174
O	-2.330161	-1.996978	2.760102
H	-4.905371	-4.157362	-0.314696
O	-5.946425	-2.245031	-1.847898
C	-7.709512	0.13432	2.943612
C	10.280158	-0.716585	-2.53805
C	-4.177388	-4.018136	2.183622
H	6.457247	3.314756	-1.665011
H	5.42839	2.94018	2.489704
H	7.185875	3.14456	2.441819
H	5.043657	5.204542	1.396992
H	6.801352	5.479462	1.450144
H	5.936533	5.629612	-0.073038
H	7.851379	2.605894	0.017748
H	7.622051	1.045218	3.531985
H	5.894967	0.709736	3.70416
H	6.155422	-1.434296	2.533599
H	7.32299	-1.356196	3.833403
H	9.119659	-0.728982	2.317707
H	7.677831	-0.562054	-3.291843
H	7.734515	-2.12688	-2.459062
H	10.043518	-0.062742	0.095052
H	5.331456	-2.338545	-1.880161
H	3.914829	0.223495	-0.893339
H	3.901982	-1.251485	-3.661561
H	4.85924	0.229615	-3.49373
H	5.614659	-1.267006	-4.071013
H	3.40778	-2.790287	-0.613865
H	0.286051	1.343408	0.157451
H	1.449155	0.768656	-1.060386
H	1.28582	-0.011576	1.865609
H	2.708974	0.597715	1.028952
H	-1.070037	0.161407	-2.051336
H	0.278724	-0.853477	-2.595455
H	-0.965109	-2.8926	-2.07513
H	-1.979322	-1.860704	-3.060986
H	-3.50484	-0.98521	1.451918
H	-5.206002	-1.107657	-0.333826
H	-6.56108	-2.306514	1.460273
H	-7.253298	-3.322479	0.213308
H	-8.75171	-1.525748	0.983617
H	-8.165026	-1.144921	-1.970797
H	-8.958881	-2.575806	-1.281253
H	-9.699782	-0.984536	-1.118989
H	-8.438785	2.809364	2.301633
H	-7.445235	3.913042	1.343872
H	-6.100654	2.293579	2.700338
H	-5.763572	2.211065	0.978139
H	-5.928419	-0.039954	1.775131
H	-10.504075	2.829827	0.886784

H	-9.674465	4.223249	0.16826
H	-10.856076	1.836104	-1.31371
H	-11.429054	3.503131	-1.343629
H	-9.382325	4.261396	-2.431094
H	-10.036068	2.908864	-3.365264
H	-7.557737	2.736737	-2.996927
H	-8.407882	1.357346	-2.307546
H	7.676768	-2.753032	0.986441
H	-1.57312	-1.382161	2.712701
H	-5.125486	-2.489787	-2.31995
H	-8.718524	0.555999	2.90394
H	-7.807223	-0.951533	3.036617
H	-7.229564	0.491634	3.861151
H	10.17839	-0.006593	-3.364914
H	11.188578	-0.457752	-1.984153
H	10.4169	-1.719599	-2.954744
H	-3.740839	-3.97339	3.178212
H	-4.844999	-4.850101	1.981445

Cartesian coordinates for conformer 2-23 after optimization at the PCM/B3LYP/6-31G* level of theory. Number of imaginary frequencies = 0. SCF Energy (PCM/mPW1PW91/6-31+G**) = -2695.11425764.

Atom	X	Y	Z
O	4.066362	0.116735	3.075859
C	3.077469	-0.644338	2.540115
C	3.593107	-2.037277	2.150762
O	1.923427	-0.257034	2.423199
C	3.154803	-2.398118	0.718584
C	3.796339	-1.54578	-0.394425
C	3.032923	-3.040886	3.158523
O	5.024916	-2.046884	2.303839
C	3.254914	-1.997237	-1.755274
C	3.991766	-1.317982	-2.897306
C	5.497036	-1.533702	-2.752793
C	5.974288	-1.087097	-1.336218
O	5.214416	-1.737276	-0.307262
O	5.843358	0.342595	-1.293849
C	6.495338	0.954735	-0.165984
C	8.016512	0.762008	-0.308039
C	8.36517	-0.663175	-0.651606
C	7.410221	-1.496454	-1.102773
C	6.084986	2.45547	-0.127546
C	4.570051	2.546483	-0.0837
C	6.786916	3.220109	0.999239
C	3.785819	2.963268	0.924532
C	2.28489	2.880232	0.939603
C	1.79589	2.227497	-0.244373
C	0.474221	2.720432	-0.460086
C	0.581019	4.200136	-0.124656
C	1.568957	4.225001	1.027596
C	0.040583	2.479828	-1.910235
C	-0.1595	0.990966	-2.198213
C	-1.057133	0.359249	-1.134492
C	-0.483981	0.672983	0.248527
O	-0.442884	2.09509	0.446042
O	-1.136429	-1.048804	-1.363143
C	-1.964668	-1.765185	-0.415461
C	-1.495157	-1.480484	0.997392
C	-1.346488	-0.008249	1.321957
C	-3.471222	-1.481443	-0.697121
C	-4.404831	-2.380703	0.13136
C	-5.928116	-2.139271	-0.02195
C	-6.373314	-0.676188	0.241831
C	-6.452905	-2.634957	-1.37888
O	-7.805644	-0.621672	0.127756

C	-8.34518	0.705471	0.150367
C	-7.965154	1.407449	1.46523
C	-6.463794	1.334256	1.731246
C	-5.939271	-0.104044	1.610649
C	-9.879755	0.598402	0.074003
C	-10.342216	0.08859	-1.287944
C	-9.723609	0.928523	-2.395829
C	-8.214986	1.005428	-2.222444
O	-7.860959	1.50857	-0.93454
H	-2.052356	0.807572	-1.244794
H	-5.947131	-0.051596	-0.553355
H	3.567883	-0.486543	-0.230648
O	6.14932	-0.799298	-3.79893
H	6.141608	0.46741	0.753717
H	1.993528	2.263767	1.797551
H	0.53146	0.264896	0.333914
O	-0.791456	0.172045	2.627021
H	-1.784578	-2.821142	-0.656097
O	-3.716897	-1.695446	-2.09259
C	-6.419268	-0.95046	2.797834
C	9.793395	-1.078643	-0.468954
C	-1.20534	-2.477331	1.85477
H	4.882523	-0.442115	3.092665
H	2.062017	-2.337081	0.637346
H	3.438638	-3.444761	0.540323
H	1.939103	-3.083302	3.123571
H	3.428172	-4.045369	2.970508
H	3.327103	-2.774486	4.180678
H	5.41351	-2.009429	1.400705
H	3.383817	-3.082005	-1.863015
H	2.181226	-1.789248	-1.825492
H	3.758893	-0.245499	-2.913905
H	3.648211	-1.711581	-3.861341
H	5.711345	-2.59861	-2.906643
H	8.509073	1.043669	0.629303
H	8.408466	1.407052	-1.104228
H	7.65607	-2.531052	-1.328019
H	6.382279	2.921177	-1.077449
H	4.084295	2.174614	-0.987167
H	6.42533	4.252681	1.061102
H	6.626478	2.740914	1.971223
H	7.866379	3.273911	0.825363
H	4.228544	3.321179	1.850408
H	-0.376451	4.655811	0.145313
H	1.011459	4.759163	-0.964276
H	1.044979	4.306283	1.987363
H	2.242666	5.086004	0.959106
H	-0.909189	2.996668	-2.096989
H	0.785467	2.878341	-2.608797
H	0.809964	0.476875	-2.222187
H	-0.597182	0.84966	-3.193482
H	-2.336782	0.46005	1.353752
H	-3.694367	-0.429744	-0.500739
H	-4.153198	-2.278969	1.1926
H	-4.203388	-3.428877	-0.127515
H	-6.424317	-2.78273	0.717716
H	-6.182023	-1.958608	-2.194305
H	-6.063561	-3.63167	-1.611261
H	-7.545921	-2.709364	-1.368043
H	-8.490687	0.93785	2.305624
H	-8.26814	2.461231	1.438509
H	-6.240217	1.746622	2.722125
H	-5.93824	1.968399	1.005724
H	-4.845431	-0.058402	1.659918
H	-10.258327	-0.075514	0.851854
H	-10.327895	1.587014	0.237594

H	-10.049548	-0.961022	-1.412079
H	-11.435163	0.127255	-1.351918
H	-10.144346	1.940389	-2.347902
H	-9.974402	0.511304	-3.376673
H	-7.793566	1.687805	-2.967812
H	-7.748748	0.02763	-2.382317
H	6.220958	0.117326	-3.464656
H	0.185554	0.105758	2.542461
H	-2.907536	-1.412694	-2.562781
H	-7.4964	-1.140724	2.761796
H	-5.911813	-1.919576	2.821839
H	-6.195914	-0.449064	3.745788
H	10.089055	-0.972456	0.579702
H	9.957295	-2.121707	-0.759089
H	10.450885	-0.45364	-1.081512
H	-0.862449	-2.274667	2.866059
H	-1.311354	-3.522075	1.577883

Cartesian coordinates for conformer 2-24 after optimization at the PCM/B3LYP/6-31G* level of theory. Number of imaginary frequencies = 0. SCF Energy (PCM/mPW1PW91/6-31+G**) = -2695.11254775.

Atom	X	Y	Z
O	8.942499	-2.787034	0.213501
C	9.682324	-1.937765	-0.548734
C	10.479269	-0.959117	0.325142
O	9.696436	-1.976107	-1.76925
C	10.421851	0.4745	-0.233261
C	8.999029	1.037688	-0.415131
C	11.921602	-1.454876	0.407905
O	9.937729	-1.019442	1.66077
C	9.069723	2.513086	-0.823492
C	7.680639	3.128799	-0.890428
C	6.935478	2.897192	0.422949
C	6.95572	1.383371	0.803699
O	8.300139	0.87998	0.824751
O	6.141147	0.700913	-0.167915
C	5.757807	-0.624328	0.228881
C	4.851829	-0.557729	1.472783
C	5.460699	0.314813	2.537477
C	6.430897	1.185656	2.207981
C	5.069743	-1.352888	-0.95714
C	3.796933	-0.668472	-1.414167
C	6.016975	-1.472758	-2.154968
C	2.584301	-1.247066	-1.397344
C	1.31758	-0.564373	-1.830305
C	0.40266	-0.641831	-0.724783
C	-0.790604	-1.305937	-1.158042
C	-0.367149	-2.1503	-2.353744
C	0.650642	-1.24958	-3.016504
C	-1.358276	-2.167539	-0.023846
C	-1.91469	-1.309792	1.111697
C	-2.898524	-0.285548	0.551784
C	-2.206548	0.51121	-0.553401
O	-1.753994	-0.346195	-1.615671
O	-3.338086	0.571926	1.603109
C	-4.281123	1.583654	1.196186
C	-3.742548	2.386098	0.020257
C	-3.193477	1.545259	-1.109809
C	-5.73322	1.042014	0.997572
C	-6.189613	0.22407	2.220985
C	-7.719767	0.173672	2.468728
C	-8.63787	-0.568616	1.455818
C	-7.985449	-0.31628	3.900256
O	-8.365445	-0.101351	0.12698
C	-9.368205	-0.46878	-0.836439

C	-9.502593	-1.999437	-0.893388
C	-9.732228	-2.598413	0.491452
C	-8.676105	-2.114001	1.500035
C	-8.913479	0.038452	-2.218022
C	-8.926186	1.562799	-2.290037
C	-10.282457	2.094281	-1.850352
C	-10.667567	1.514218	-0.498522
O	-10.649301	0.086765	-0.517773
H	-3.744067	-0.853168	0.149156
H	-9.651865	-0.230933	1.716175
H	8.479349	0.464365	-1.191448
O	5.599732	3.397095	0.265415
H	6.669257	-1.184415	0.477967
H	1.501099	0.49332	-2.050765
H	-1.339199	1.057932	-0.156287
O	-2.518123	2.357436	-2.081143
H	-4.331241	2.256374	2.063157
O	-5.81572	0.221121	-0.169205
C	-7.334813	-2.794496	1.221577
C	4.926062	0.168139	3.929221
C	-3.727384	3.73245	0.033213
H	9.115238	-2.554373	1.15941
H	10.948231	0.527064	-1.19463
H	10.95804	1.124599	0.471732
H	12.402182	-1.467501	-0.576141
H	12.514142	-0.82327	1.079282
H	11.96328	-2.471734	0.81604
H	9.238	-0.328341	1.707621
H	9.658025	3.073469	-0.085523
H	9.572515	2.618584	-1.791288
H	7.116492	2.699301	-1.728149
H	7.748643	4.20326	-1.097796
H	7.424722	3.490146	1.205824
H	4.694145	-1.573086	1.854674
H	3.870796	-0.133841	1.229658
H	6.882543	1.806816	2.977654
H	4.831147	-2.372263	-0.6245
H	3.899949	0.35746	-1.765178
H	5.543975	-2.025264	-2.974862
H	6.315552	-0.492095	-2.541892
H	6.923281	-2.016506	-1.87367
H	2.482821	-2.268983	-1.038906
H	-1.193754	-2.401532	-3.025056
H	0.123976	-3.076389	-2.032077
H	0.133662	-0.500595	-3.629368
H	1.342283	-1.788227	-3.67164
H	-2.171176	-2.794045	-0.412394
H	-0.584925	-2.831277	0.379929
H	-1.100037	-0.800128	1.641876
H	-2.411341	-1.939998	1.858872
H	-4.00544	1.049772	-1.651636
H	-6.396928	1.901567	0.841726
H	-5.744007	0.685952	3.112502
H	-5.777223	-0.785789	2.16407
H	-8.046145	1.224246	2.43923
H	-7.583724	-1.321501	4.062418
H	-7.515045	0.350542	4.631139
H	-9.058667	-0.333067	4.11743
H	-8.590121	-2.438959	-1.314842
H	-10.337321	-2.285968	-1.544618
H	-9.73734	-3.693001	0.428653
H	-10.728301	-2.306478	0.848603
H	-9.008548	-2.440722	2.492861
H	-7.902702	-0.319022	-2.447924
H	-9.589699	-0.344995	-2.992889
H	-8.705702	1.89207	-3.311461

H	-8.14068	1.972919	-1.6437
H	-11.033108	1.801988	-2.594851
H	-10.268152	3.18821	-1.806538
H	-11.684902	1.825767	-0.240543
H	-10.006715	1.884402	0.293322
H	5.093513	2.664736	-0.139644
H	-2.031388	1.721879	-2.638949
H	-6.76849	0.018577	-0.270367
H	-6.667787	-2.702768	2.082445
H	-7.469341	-3.868431	1.050917
H	-6.830729	-2.377143	0.345405
H	5.090091	-0.850937	4.29384
H	5.411209	0.856487	4.629103
H	3.851345	0.37565	3.946281
H	-3.324672	4.298846	-0.802342
H	-4.111795	4.304273	0.871479

Cartesian coordinates for conformer 2-25 after optimization at the PCM/B3LYP/6-31G* level of theory. Number of imaginary frequencies = 0. SCF Energy (PCM/mPW1PW91/6-31+G**) = -2695.10962586.

Atom	X	Y	Z
O	-5.132213	0.951941	2.094673
C	-6.469838	1.185228	2.007665
C	-7.26211	0.14768	2.818182
O	-6.944094	2.113625	1.372054
C	-8.54907	-0.298692	2.102367
C	-8.339801	-0.829011	0.672322
C	-7.582496	0.751497	4.184539
O	-6.387622	-0.976701	3.051508
C	-9.641865	-1.432633	0.135184
C	-9.430527	-2.046268	-1.241174
C	-8.259515	-3.02878	-1.216087
C	-6.995627	-2.359063	-0.590958
O	-7.301265	-1.816183	0.70388
O	-6.565575	-1.337191	-1.509889
C	-5.249545	-0.836696	-1.221099
C	-4.219898	-1.951204	-1.476027
C	-4.630904	-3.22477	-0.779407
C	-5.902602	-3.380193	-0.367003
C	-5.030685	0.454272	-2.047563
C	-3.760102	1.170052	-1.650564
C	-5.052458	0.222638	-3.562401
C	-3.744795	2.317787	-0.950458
C	-2.526469	3.089303	-0.508402
C	-1.330282	2.382574	-0.877287
C	-0.612758	2.029247	0.311871
C	-1.624397	2.116358	1.448211
C	-2.476853	3.281232	1.001885
C	-0.029354	0.617181	0.17791
C	1.091388	0.565557	-0.859274
C	2.128199	1.643518	-0.553946
C	1.423001	2.995819	-0.460343
O	0.418471	2.994286	0.568629
O	3.119354	1.651679	-1.579695
C	4.162734	2.628455	-1.398358
C	3.576262	4.016423	-1.185116
C	2.46891	4.076227	-0.15748
C	5.235688	2.218562	-0.337708
C	5.788279	0.81049	-0.631035
C	6.900713	0.313312	0.323748
C	7.391399	-1.115371	-0.059676
C	8.090964	1.288407	0.343679
O	8.407164	-1.49304	0.885235
C	9.10064	-2.702183	0.55577
C	8.106216	-3.871782	0.473561

C	6.937967	-3.551258	-0.453918
C	6.292136	-2.202031	-0.105822
C	10.112471	-2.993814	1.679902
C	11.246177	-1.972497	1.697191
C	11.871149	-1.865245	0.314016
C	10.799049	-1.609051	-0.733307
O	9.784054	-2.611578	-0.70084
H	2.583038	1.376955	0.406017
H	7.851945	-1.04418	-1.054427
H	-8.025936	0.0012	0.028635
O	-8.027054	-3.471143	-2.56105
H	-5.216431	-0.55638	-0.159897
H	-2.527986	4.057838	-1.020318
H	0.942518	3.254029	-1.414767
O	1.828531	5.359633	-0.157748
H	4.687212	2.630863	-2.363562
O	4.706549	2.242848	0.991646
C	5.49751	-2.307708	1.202531
C	-3.574662	-4.270278	-0.590018
C	3.997936	5.07705	-1.899485
H	-5.014399	0.140121	2.64747
H	-9.269796	0.527891	2.068267
H	-8.998467	-1.101702	2.702483
H	-8.222031	1.636081	4.095269
H	-8.083882	0.019452	4.827579
H	-6.665458	1.050608	4.705966
H	-6.505724	-1.579456	2.28185
H	-9.990658	-2.222299	0.813046
H	-10.427231	-0.670062	0.087803
H	-9.246068	-1.256535	-1.980783
H	-10.340974	-2.558126	-1.574939
H	-8.555054	-3.902477	-0.621815
H	-3.235095	-1.629996	-1.118888
H	-4.137149	-2.177176	-2.544666
H	-6.196178	-4.288718	0.152786
H	-5.889201	1.108443	-1.835924
H	-2.818847	0.719434	-1.959753
H	-5.033895	1.179543	-4.097061
H	-4.187991	-0.356457	-3.903214
H	-5.960282	-0.307089	-3.868721
H	-4.695385	2.761059	-0.652364
H	-1.167436	2.290155	2.427003
H	-2.2421	1.212035	1.500525
H	-1.963177	4.221999	1.236283
H	-3.456748	3.311132	1.48765
H	0.380901	0.297714	1.144304
H	-0.810171	-0.096978	-0.108754
H	0.685954	0.706687	-1.869221
H	1.565408	-0.423185	-0.861423
H	2.873917	3.946629	0.851361
H	6.036603	2.964845	-0.381697
H	6.161096	0.7788	-1.662466
H	4.949258	0.109191	-0.570311
H	6.514092	0.278369	1.349659
H	8.89574	0.927963	0.992527
H	8.502756	1.42647	-0.661611
H	7.799399	2.268433	0.732187
H	7.708077	-4.096029	1.470751
H	8.608017	-4.77684	0.110193
H	6.19669	-4.358009	-0.412024
H	7.300211	-3.51696	-1.489451
H	5.581733	-1.970592	-0.908229
H	9.615141	-2.98804	2.657225
H	10.552528	-3.988515	1.532031
H	10.861071	-0.993297	2.006404
H	12.004541	-2.266054	2.43139

H	12.382177	-2.808167	0.083931
H	12.621958	-1.068414	0.294769
H	11.24833	-1.629753	-1.731457
H	10.349119	-0.618999	-0.604446
H	-7.472749	-2.777382	-2.970838
H	1.010283	5.228461	0.358037
H	5.461777	2.228889	1.605921
H	6.149339	-2.433589	2.07258
H	4.891186	-1.411672	1.368119
H	4.808598	-3.158721	1.169688
H	-2.757736	-3.877978	0.024247
H	-3.966943	-5.164323	-0.094212
H	-3.166529	-4.576357	-1.558579
H	3.562166	6.062264	-1.755227
H	4.782042	4.99435	-2.645283

Cartesian coordinates for conformer 2-26 after optimization at the PCM/B3LYP/6-31G* level of theory. Number of imaginary frequencies = 0. SCF Energy (PCM/mPW1PW91/6-31+G**) = -2695.1096278.

Atom	X	Y	Z
O	7.485599	-1.555581	-0.098668
C	6.446713	-1.910577	-0.901855
C	6.590917	-1.294158	-2.300891
O	5.546184	-2.650817	-0.5393
C	5.266815	-0.683514	-2.79727
C	4.688374	0.436279	-1.909229
C	7.072408	-2.389432	-3.251968
O	7.637935	-0.303386	-2.244159
C	3.477431	1.074674	-2.599936
C	2.972998	2.285675	-1.829231
C	4.116188	3.266887	-1.568701
C	5.316138	2.528762	-0.900574
O	5.722193	1.404194	-1.694457
O	4.880547	2.150513	0.416087
C	5.939188	1.63939	1.243915
C	6.929285	2.782444	1.54129
C	7.274516	3.564702	0.299013
C	6.530742	3.421879	-0.813089
C	5.310868	1.049651	2.536984
C	4.212068	0.060746	2.181995
C	6.374522	0.480169	3.482721
C	4.235437	-1.278889	2.29019
C	3.126586	-2.224873	1.89146
C	2.007811	-1.49031	1.364163
C	0.864849	-1.722048	2.195162
C	1.416178	-2.220621	3.525474
C	2.607093	-3.033629	3.072961
C	0.061043	-0.426523	2.361742
C	-0.609144	-0.007412	1.05408
C	-1.415451	-1.174542	0.490542
C	-0.500995	-2.392041	0.363065
O	0.054437	-2.766511	1.635707
O	-1.959432	-0.807221	-0.775749
C	-2.754796	-1.830279	-1.405324
C	-1.98073	-3.137117	-1.497882
C	-1.312562	-3.560865	-0.209186
C	-4.189656	-1.977398	-0.801947
C	-4.904347	-0.613526	-0.745959
C	-6.355203	-0.637344	-0.207318
C	-6.985068	0.788074	-0.187895
C	-7.237251	-1.589319	-1.034144
O	-8.317588	0.665568	0.337807
C	-9.103578	1.859065	0.240056
C	-8.42045	3.001462	1.009185
C	-6.966994	3.176705	0.579986

C	-6.200041	1.846751	0.621049
C	-10.47482	1.581904	0.884571
C	-11.295665	0.593632	0.061036
C	-11.373937	1.060156	-1.385059
C	-9.981459	1.339108	-1.927741
O	-9.282134	2.282764	-1.117317
H	-2.222725	-1.370105	1.2042
H	-7.053021	1.127217	-1.23052
H	4.388136	0.012834	-0.944289
O	3.608665	4.327313	-0.746163
H	6.453255	0.834155	0.703897
H	3.498784	-2.890528	1.106829
H	0.326698	-2.188776	-0.331563
O	-0.43766	-4.6774	-0.421585
H	-2.901275	-1.453652	-2.426793
O	-4.158939	-2.540348	0.513203
C	-5.937839	1.421352	2.071797
C	8.454672	4.484686	0.380643
C	-1.878945	-3.815132	-2.656759
H	8.086266	-0.985281	-0.639294
H	4.509244	-1.468644	-2.915353
H	5.456928	-0.259103	-3.792917
H	6.342125	-3.201096	-3.337713
H	7.263244	-1.983039	-4.25157
H	8.017807	-2.822241	-2.903817
H	7.196209	0.558511	-2.066818
H	3.759766	1.406872	-3.607238
H	2.671555	0.340447	-2.712584
H	2.521447	1.967039	-0.880802
H	2.171086	2.785268	-2.385896
H	4.425739	3.70268	-2.526652
H	7.843867	2.367874	1.979731
H	6.493623	3.480627	2.266802
H	6.79255	3.967716	-1.715791
H	4.802598	1.863092	3.074047
H	3.315716	0.52844	1.773598
H	5.913104	-0.002236	4.351844
H	7.009094	-0.256226	2.977803
H	7.023188	1.27322	3.868016
H	5.132541	-1.773863	2.655586
H	0.70084	-2.819494	4.097049
H	1.761766	-1.388523	4.15037
H	2.268418	-4.017499	2.724899
H	3.342296	-3.199323	3.866381
H	-0.720447	-0.575585	3.117467
H	0.709312	0.388491	2.704017
H	0.14331	0.31932	0.324843
H	-1.262703	0.85728	1.219183
H	-2.058236	-3.901889	0.516252
H	-4.740387	-2.67652	-1.441073
H	-4.900751	-0.164498	-1.747172
H	-4.312039	0.041036	-0.097913
H	-6.3589	-1.026578	0.818085
H	-7.242384	-1.301965	-2.090962
H	-6.887863	-2.623289	-0.961806
H	-8.272641	-1.589269	-0.6784
H	-8.443365	2.794483	2.086034
H	-8.953485	3.946126	0.845869
H	-6.478534	3.925339	1.214673
H	-6.939752	3.572936	-0.44313
H	-5.227725	2.023649	0.146053
H	-10.350613	1.181726	1.897962
H	-11.043761	2.517549	0.959416
H	-12.30303	0.500443	0.481596
H	-10.835359	-0.400717	0.104844
H	-11.968495	1.980998	-1.426778

H	-11.880765	0.31025	-2.001331
H	-10.057526	1.764068	-2.933828
H	-9.397986	0.416309	-2.012859
H	3.648371	3.984484	0.169133
H	0.109298	-4.722038	0.385641
H	-5.061787	-2.836907	0.724439
H	-5.243751	0.57619	2.115073
H	-5.481435	2.239912	2.638855
H	-6.85635	1.124091	2.587244
H	9.363466	3.91778	0.606652
H	8.620663	5.022897	-0.558283
H	8.301984	5.227671	1.169826
H	-1.309617	-4.738819	-2.720164
H	-2.3551	-3.471034	-3.569191

Cartesian coordinates for conformer 2-27 after optimization at the PCM/B3LYP/6-31G* level of theory. Number of imaginary frequencies = 0. SCF Energy (PCM/mPW1PW91/6-31+G**) = -2695.10943804.

Atom	X	Y	Z
O	-8.392228	-1.74715	-2.505409
C	-8.565029	-2.605103	-1.464552
C	-9.816513	-2.24622	-0.651213
O	-7.808991	-3.535969	-1.233844
C	-9.550601	-2.329373	0.862866
C	-8.382102	-1.4564	1.360378
C	-10.942756	-3.193301	-1.060185
O	-10.225404	-0.91741	-1.03539
C	-8.315262	-1.496807	2.890886
C	-7.249374	-0.547666	3.416387
C	-7.468121	0.856241	2.855354
C	-7.585177	0.811319	1.299197
O	-8.596902	-0.121131	0.889695
O	-6.28482	0.450874	0.795594
C	-6.118647	0.70571	-0.607502
C	-6.199298	2.219636	-0.878488
C	-7.411226	2.822565	-0.220434
C	-8.035019	2.152647	0.764746
C	-4.77858	0.095643	-1.100681
C	-3.564907	0.689983	-0.410346
C	-4.757665	-1.422852	-0.898788
C	-2.570265	1.319329	-1.059357
C	-1.321762	1.936039	-0.483721
C	-0.220931	1.268963	-1.128637
C	0.576375	0.609854	-0.135544
C	-0.294897	0.52845	1.113654
C	-1.078258	1.81668	1.015542
C	0.994401	-0.780806	-0.628747
C	1.992069	-0.694911	-1.782765
C	3.160374	0.205362	-1.389351
C	2.611001	1.556926	-0.935205
O	1.721352	1.414097	0.185208
O	4.037056	0.357217	-2.503988
C	5.18927	1.185824	-2.25096
C	4.772327	2.538605	-1.69156
C	3.786324	2.463976	-0.547983
C	6.310364	0.466277	-1.434569
C	6.651197	-0.90297	-2.053238
C	8.086354	-1.427881	-1.788508
C	8.505382	-1.822967	-0.343443
C	8.398893	-2.56487	-2.773133
O	8.196329	-0.750463	0.557993
C	8.830955	-0.868393	1.843314
C	8.410349	-2.185768	2.515494
C	8.644587	-3.385825	1.602102
C	8.008387	-3.175517	0.217072

C	8.357609	0.305985	2.720589
C	8.890332	1.641225	2.208714
C	10.400159	1.567496	2.033909
C	10.77861	0.361339	1.188994
O	10.25896	-0.84825	1.74169
H	3.679173	-0.298357	-0.566941
H	9.601604	-1.897738	-0.39331
H	-7.443086	-1.83449	0.939984
O	-6.376166	1.679386	3.290431
H	-6.931491	0.200693	-1.146284
H	-1.301474	2.990722	-0.781123
H	2.064184	2.050539	-1.751439
O	3.279849	3.764212	-0.213178
H	5.612304	1.355185	-3.250601
O	5.909788	0.27777	-0.075982
C	6.487609	-3.306683	0.31403
C	-7.852726	4.169845	-0.704393
C	5.224772	3.690806	-2.221455
H	-9.138934	-1.098743	-2.475222
H	-9.36453	-3.369851	1.15757
H	-10.464401	-2.000193	1.376435
H	-10.699529	-4.235487	-0.827479
H	-11.877989	-2.930112	-0.553382
H	-11.139538	-3.125782	-2.136768
H	-9.771939	-0.298646	-0.418348
H	-9.28228	-1.193086	3.311761
H	-8.112435	-2.515997	3.238266
H	-6.250666	-0.91373	3.146047
H	-7.269239	-0.519989	4.512358
H	-8.389007	1.262545	3.292085
H	-6.229053	2.387449	-1.961257
H	-5.321842	2.742658	-0.48133
H	-8.920008	2.579168	1.230681
H	-4.708508	0.28332	-2.180759
H	-3.534021	0.567696	0.669513
H	-3.828435	-1.856747	-1.285135
H	-4.840017	-1.697465	0.158635
H	-5.586573	-1.892209	-1.436387
H	-2.636939	1.411888	-2.143372
H	0.280924	0.465256	2.041937
H	-0.983016	-0.323527	1.065893
H	-0.44374	2.64906	1.346744
H	-1.977402	1.836137	1.635975
H	1.465906	-1.333863	0.193504
H	0.11861	-1.351098	-0.959522
H	1.502659	-0.306028	-2.684849
H	2.358433	-1.693875	-2.046813
H	4.274994	2.094179	0.35911
H	7.194871	1.115141	-1.428899
H	6.549823	-0.807546	-3.142981
H	5.909584	-1.642382	-1.743104
H	8.748633	-0.593264	-2.064009
H	7.704675	-3.402594	-2.653923
H	8.319747	-2.213561	-3.807858
H	9.418937	-2.937681	-2.632536
H	7.345169	-2.149348	2.774826
H	8.970789	-2.33604	3.446126
H	8.256742	-4.295818	2.074716
H	9.724554	-3.539205	1.480157
H	8.366376	-3.988664	-0.42605
H	7.262494	0.349339	2.751221
H	8.716894	0.167153	3.748386
H	8.631142	2.442337	2.909542
H	8.41846	1.887944	1.249841
H	10.864969	1.474722	3.023118
H	10.776954	2.487987	1.575928

H	11.868405	0.264109	1.153469
H	10.43175	0.477472	0.155941
H	-5.662495	1.528838	2.639351
H	2.514715	3.589071	0.366716
H	6.702164	-0.078648	0.375614
H	6.04824	-3.461055	-0.674794
H	6.207864	-4.180205	0.913414
H	6.022434	-2.427097	0.767793
H	-8.119433	4.123928	-1.765113
H	-8.726042	4.536705	-0.155067
H	-7.047268	4.900348	-0.578878
H	4.908757	4.653366	-1.828353
H	5.91719	3.707302	-3.056886

Cartesian coordinates for conformer 2-28 after optimization at the PCM/B3LYP/6-31G* level of theory. Number of imaginary frequencies = 0. SCF Energy (PCM/mPW1PW91/6-31+G**) = -2695.1146771.

Atom	X	Y	Z
O	5.822791	1.598712	-2.44628
C	5.386509	2.369745	-1.415723
C	6.493286	3.308473	-0.916277
O	4.251159	2.31204	-0.96883
C	6.515524	3.372121	0.621546
C	6.715623	2.00962	1.310857
C	6.252752	4.692608	-1.515664
O	7.74845	2.834716	-1.443026
C	6.781447	2.198807	2.829201
C	7.092028	0.883208	3.524064
C	8.363005	0.270176	2.941293
C	8.266565	0.172498	1.384733
O	7.929239	1.440842	0.806055
O	7.279276	-0.832326	1.090945
C	7.310228	-1.31534	-0.267189
C	8.652095	-2.034553	-0.50874
C	9.819864	-1.209626	-0.042988
C	9.614261	-0.190444	0.807886
C	6.083232	-2.258276	-0.462971
C	4.790491	-1.459103	-0.347516
C	6.174751	-3.090918	-1.749027
C	3.776597	-1.401306	-1.229391
C	2.532442	-0.578529	-1.032828
C	1.446766	-1.50576	-0.847663
C	0.445487	-1.248315	-1.836779
C	1.180524	-0.574703	-2.98689
C	2.185722	0.277311	-2.246022
C	-0.218548	-2.560647	-2.272315
C	-1.076403	-3.158326	-1.156925
C	-2.044303	-2.104411	-0.626729
C	-1.251046	-0.864844	-0.213037
O	-0.528104	-0.322857	-1.329275
O	-2.785682	-2.640853	0.469915
C	-3.742597	-1.72458	1.057229
C	-3.063917	-0.419391	1.427176
C	-2.23523	0.185324	0.317933
C	-4.98793	-1.591501	0.131693
C	-6.123338	-0.781386	0.77811
C	-7.437167	-0.721057	-0.039432
C	-8.535425	0.101798	0.697992
C	-7.189993	-0.131234	-1.439266
O	-9.712667	0.100182	-0.127457
C	-10.751655	0.977081	0.32123
C	-11.196792	0.581667	1.73844
C	-10.010138	0.484794	2.692179
C	-8.891917	-0.397499	2.117609
C	-11.951083	0.823333	-0.632844

C	-11.642202	1.37781	-2.020695
C	-11.118987	2.802066	-1.907996
C	-9.96164	2.865222	-0.923882
O	-10.331051	2.347663	0.353615
H	-2.731148	-1.859271	-1.446618
H	-8.168919	1.134561	0.775421
H	5.880831	1.345799	1.059812
O	8.552084	-1.014702	3.552142
H	7.21919	-0.458653	-0.949055
H	2.610764	0.04201	-0.133186
H	-0.539043	-1.098642	0.590959
O	-1.513386	1.333372	0.770351
H	-4.070743	-2.238569	1.970202
O	-5.476598	-2.905689	-0.175693
C	-9.302939	-1.875392	2.148748
C	11.17451	-1.583951	-0.562332
C	-3.170479	0.106255	2.661702
H	6.768666	1.837585	-2.608705
H	5.587505	3.82606	0.991691
H	7.345922	4.028378	0.915976
H	5.29753	5.115756	-1.187092
H	7.056414	5.383673	-1.237572
H	6.241966	4.648359	-2.611217
H	8.134213	2.242534	-0.757932
H	7.571842	2.91776	3.079975
H	5.836196	2.605693	3.20566
H	6.249612	0.18827	3.414961
H	7.211755	1.039244	4.602781
H	9.211579	0.904205	3.227643
H	8.76405	-2.245455	-1.578209
H	8.675962	-2.988435	0.03271
H	10.452851	0.422119	1.129998
H	6.067021	-2.963583	0.380083
H	4.693452	-0.888487	0.57658
H	5.292779	-3.729282	-1.873103
H	6.266838	-2.451447	-2.63374
H	7.035832	-3.766162	-1.726322
H	3.817236	-1.969629	-2.15345
H	0.529132	0.023145	-3.631344
H	1.712678	-1.309393	-3.602809
H	1.704658	1.206139	-1.914475
H	3.045999	0.554272	-2.862684
H	-0.864571	-2.372725	-3.139279
H	0.538478	-3.295685	-2.569445
H	-0.440999	-3.529397	-0.342766
H	-1.630973	-4.029025	-1.52599
H	-2.890707	0.535674	-0.488105
H	-4.697348	-1.135019	-0.818302
H	-5.780687	0.241207	0.97667
H	-6.33841	-1.243098	1.748225
H	-7.816213	-1.738308	-0.198026
H	-6.752936	0.870724	-1.372009
H	-6.514085	-0.761357	-2.024868
H	-8.118428	-0.057966	-2.0146
H	-11.704765	-0.390109	1.714254
H	-11.91009	1.314467	2.1349
H	-10.343486	0.103357	3.664536
H	-9.612388	1.491449	2.874297
H	-8.022848	-0.280767	2.775778
H	-12.235404	-0.23138	-0.727995
H	-12.814479	1.369962	-0.232257
H	-10.894217	0.747067	-2.516105
H	-12.543698	1.356217	-2.642756
H	-11.93064	3.448576	-1.552111
H	-10.806268	3.172992	-2.889651
H	-9.658432	3.907399	-0.779873

H	-9.087767	2.327111	-1.306014
H	7.994214	-1.631062	3.037006
H	-0.82742	1.489968	0.093238
H	-4.687466	-3.476228	-0.263177
H	-10.101434	-2.096616	1.433628
H	-8.454647	-2.524954	1.911721
H	-9.654362	-2.158754	3.146691
H	11.201863	-1.494087	-1.652894
H	11.962009	-0.942157	-0.153712
H	11.411482	-2.617839	-0.291616
H	-2.665339	1.031388	2.927281
H	-3.757684	-0.369803	3.440999

Cartesian coordinates for conformer 2-29 after optimization at the PCM/B3LYP/6-31G* level of theory. Number of imaginary frequencies = 0. SCF Energy (PCM/mPW1PW91/6-31+G**) = -2695.10849222.

Atom	X	Y	Z
O	-8.486415	-1.31872	-0.614179
C	-7.887684	-1.958438	-1.654315
C	-7.503643	-3.395694	-1.274904
O	-7.69915	-1.434752	-2.741188
C	-6.096436	-3.759421	-1.782681
C	-4.97679	-2.814309	-1.306004
C	-8.553966	-4.341874	-1.853278
O	-7.582985	-3.508113	0.161019
C	-3.611485	-3.362121	-1.735069
C	-2.481571	-2.511097	-1.176329
C	-2.628228	-2.362794	0.337167
C	-4.061163	-1.863224	0.702165
O	-5.063118	-2.71082	0.119741
O	-4.159465	-0.509874	0.223701
C	-5.251374	0.229902	0.789366
C	-5.044027	0.393662	2.306937
C	-4.718637	-0.925218	2.953851
C	-4.283094	-1.94643	2.195491
C	-5.384642	1.600703	0.071978
C	-4.134059	2.445948	0.196938
C	-5.704086	1.41577	-1.414581
C	-4.078932	3.639891	0.812246
C	-2.849377	4.490934	0.973289
C	-1.717589	3.907088	0.31019
C	-0.575617	3.968352	1.175848
C	-0.973111	4.888967	2.326658
C	-2.458699	4.664542	2.432948
C	-0.242035	2.545733	1.662137
C	0.288574	1.675186	0.522779
C	1.441804	2.387203	-0.179353
C	0.97649	3.775809	-0.615288
O	0.542267	4.560928	0.507946
O	1.863667	1.614514	-1.301417
C	2.963046	2.180742	-2.040195
C	2.663369	3.617356	-2.442569
C	2.140064	4.485192	-1.320313
C	4.353831	1.985455	-1.353318
C	4.580498	0.506944	-0.983181
C	5.949406	0.177138	-0.340526
C	6.077297	-1.340087	-0.007969
C	7.109561	0.61365	-1.252339
O	7.367311	-1.544969	0.592995
C	7.72055	-2.920399	0.779802
C	6.687711	-3.609255	1.686451
C	5.26468	-3.39508	1.179924
C	4.975596	-1.910206	0.914859
C	9.095248	-2.970215	1.472579
C	10.208856	-2.478579	0.552502

C	10.156186	-3.223856	-0.772923
C	8.758897	-3.149015	-1.367563
O	7.775128	-3.640726	-0.458464
H	2.253382	2.463014	0.551973
H	6.048304	-1.884818	-0.961451
H	-5.130606	-1.821596	-1.74477
O	-1.612765	-1.456852	0.7925
H	-6.177296	-0.333292	0.611196
H	-3.025077	5.467412	0.50669
H	0.145679	3.703486	-1.331669
O	1.685547	5.753127	-1.813524
H	2.998834	1.576259	-2.956842
O	4.482391	2.785604	-0.173914
C	4.844316	-1.143422	2.237313
C	-4.888906	-1.019576	4.439249
C	2.817754	4.040391	-3.711427
H	-8.526013	-1.960207	0.137912
H	-6.08698	-3.793272	-2.879418
H	-5.866269	-4.770775	-1.419987
H	-8.579975	-4.297403	-2.947165
H	-8.357991	-5.376177	-1.548991
H	-9.554458	-4.090055	-1.482206
H	-6.688335	-3.284317	0.505915
H	-3.488416	-4.38566	-1.358603
H	-3.542406	-3.404501	-2.827767
H	-2.478953	-1.523889	-1.656057
H	-1.510377	-2.960003	-1.415824
H	-2.438766	-3.339515	0.799735
H	-5.95229	0.821373	2.747136
H	-4.212781	1.073826	2.525165
H	-4.060559	-2.905094	2.657491
H	-6.230607	2.134235	0.525796
H	-3.230244	2.029129	-0.246223
H	-5.830816	2.38442	-1.911445
H	-4.911522	0.871691	-1.94009
H	-6.63725	0.858753	-1.539302
H	-4.987252	4.054249	1.245694
H	-0.774614	5.936692	2.066242
H	-0.436081	4.682931	3.257593
H	-2.984633	5.494157	2.91526
H	-2.658669	3.756891	3.015881
H	0.529429	2.596297	2.440825
H	-1.123905	2.058559	2.093627
H	-0.511116	1.453078	-0.19517
H	0.623871	0.704952	0.907897
H	2.938705	4.713465	-0.607115
H	5.116192	2.327838	-2.061779
H	4.44704	-0.112069	-1.879327
H	3.79513	0.22149	-0.275243
H	6.061522	0.742575	0.592695
H	7.039205	0.134412	-2.234492
H	7.117388	1.697484	-1.399451
H	8.081114	0.355667	-0.818867
H	6.758497	-3.210296	2.705662
H	6.884399	-4.686836	1.742162
H	4.547042	-3.80722	1.898959
H	5.126677	-3.957534	0.24743
H	4.007013	-1.859048	0.403661
H	9.091049	-2.357945	2.382325
H	9.325315	-4.003438	1.76321
H	10.098633	-1.401754	0.376294
H	11.183521	-2.627752	1.029886
H	10.417524	-4.274652	-0.597028
H	10.891345	-2.812023	-1.472023
H	8.709981	-3.767708	-2.269579
H	8.510884	-2.125296	-1.667117

H	-1.970961	-0.562487	0.623722
H	1.154944	6.127669	-1.084593
H	5.4273	2.812678	0.058295
H	5.801109	-1.05748	2.761596
H	4.465274	-0.130628	2.069127
H	4.135576	-1.64345	2.906311
H	-5.932886	-0.838418	4.714305
H	-4.607927	-2.006228	4.822126
H	-4.261961	-0.275249	4.940532
H	2.584379	5.062944	-3.99613
H	3.176127	3.384015	-4.497948

Cartesian coordinates for conformer 2-30 after optimization at the PCM/B3LYP/6-31G* level of theory. Number of imaginary frequencies = 0. SCF Energy (PCM/mPW1PW91/6-31+G**) = -2695.11261039.

Atom	X	Y	Z
O	2.753714	-0.180925	1.762132
C	3.98376	-0.420949	2.264542
C	4.276847	-1.925913	2.403263
O	4.746081	0.495829	2.526254
C	3.89701	-2.681622	1.111534
C	4.533104	-2.097862	-0.165144
C	3.478568	-2.464472	3.591957
O	5.653821	-2.174179	2.703856
C	4.128229	-2.938735	-1.378551
C	4.80803	-2.420156	-2.634424
C	6.318509	-2.369741	-2.425447
C	6.678711	-1.582954	-1.122275
O	5.952822	-2.081664	0.006934
O	6.385462	-0.195092	-1.378724
C	7.029549	0.724811	-0.470784
C	8.556936	0.616812	-0.652116
C	9.023943	-0.809912	-0.603451
C	8.13815	-1.799987	-0.798292
C	6.491156	2.15643	-0.787714
C	5.028087	2.269899	-0.367653
C	7.377687	3.272048	-0.215951
C	4.484312	3.180253	0.46202
C	3.044494	3.278221	0.899159
C	2.312394	2.111181	0.474594
C	1.304009	2.522079	-0.467092
C	1.70787	3.916558	-0.935899
C	2.337721	4.489379	0.311444
C	1.229791	1.536375	-1.641445
C	0.646821	0.18797	-1.224698
C	-0.675914	0.401131	-0.496092
C	-0.45523	1.37115	0.664633
O	0.036844	2.636198	0.196822
O	-1.177738	-0.854529	-0.038344
C	-2.441666	-0.77824	0.667145
C	-2.354666	0.233604	1.793771
C	-1.795262	1.575331	1.382248
C	-3.603248	-0.552709	-0.345455
C	-4.989813	-0.64794	0.311788
C	-6.188657	-0.559332	-0.664471
C	-7.549504	-0.687301	0.083247
C	-6.151452	0.753539	-1.46666
O	-8.597865	-0.598365	-0.896701
C	-9.916318	-0.504943	-0.346502
C	-10.213343	-1.74101	0.517731
C	-9.121301	-1.973842	1.557146
C	-7.723973	-1.975843	0.919844
C	-10.919471	-0.467947	-1.514766
C	-10.812391	0.830761	-2.308848
C	-10.910023	2.024455	-1.370322

C	-9.909626	1.887777	-0.233598
O	-10.0829	0.659774	0.472315
H	-1.376221	0.831976	-1.222735
H	-7.631507	0.176854	0.75646
H	4.185352	-1.068984	-0.312439
O	6.902946	-1.775033	-3.594203
H	6.757534	0.45309	0.558233
H	3.020744	3.307628	1.994107
H	0.259069	0.964423	1.392612
O	-1.605981	2.431856	2.510714
H	-2.567979	-1.784795	1.086998
O	-3.513937	-1.545119	-1.378524
C	-7.50692	-3.256098	0.102398
C	10.478064	-1.049582	-0.334389
C	-2.710565	-0.081345	3.053187
H	2.728484	0.7844	1.548978
H	2.806698	-2.698875	0.987329
H	4.231684	-3.722524	1.218482
H	2.399441	-2.342483	3.4503
H	3.690122	-3.527033	3.756901
H	3.757373	-1.941708	4.514615
H	6.157446	-1.936921	1.89746
H	4.427149	-3.983402	-1.224909
H	3.04043	-2.922512	-1.510559
H	4.425362	-1.42377	-2.890366
H	4.569145	-3.060908	-3.491561
H	6.686767	-3.401343	-2.356972
H	9.057868	1.185895	0.139217
H	8.857123	1.036234	-1.620301
H	8.464022	-2.835499	-0.737103
H	6.490244	2.27688	-1.880596
H	4.382689	1.511023	-0.807031
H	6.961565	4.262061	-0.434391
H	7.492993	3.175865	0.86921
H	8.375095	3.259154	-0.666682
H	5.118603	3.940348	0.913081
H	0.864457	4.519252	-1.286169
H	2.459936	3.86677	-1.732285
H	1.550974	4.826988	0.997505
H	2.993454	5.342232	0.111621
H	0.588181	1.956179	-2.42669
H	2.223516	1.373964	-2.074029
H	1.351225	-0.35369	-0.582256
H	0.494758	-0.449851	-2.103421
H	-2.506702	2.088881	0.724486
H	-3.487275	0.418842	-0.833024
H	-5.092515	0.13483	1.072732
H	-5.035649	-1.611015	0.832101
H	-6.121787	-1.370363	-1.400259
H	-6.156737	1.621976	-0.799553
H	-5.259529	0.811205	-2.097347
H	-7.010753	0.838241	-2.139725
H	-10.286974	-2.63293	-0.116326
H	-11.174098	-1.625881	1.034127
H	-9.306469	-2.914998	2.088006
H	-9.168176	-1.178368	2.311963
H	-6.998243	-1.979751	1.741667
H	-10.752522	-1.313045	-2.193344
H	-11.94291	-0.545777	-1.125587
H	-11.60745	0.877436	-3.061205
H	-9.857697	0.860613	-2.847551
H	-11.924525	2.064493	-0.955145
H	-10.739667	2.956594	-1.919066
H	-10.055748	2.70154	0.484055
H	-8.880717	1.965002	-0.600811
H	6.864336	-0.810053	-3.439907

H	-1.068467	3.177104	2.182279
H	-2.560507	-1.690829	-1.536802
H	-8.123944	-3.278288	-0.801207
H	-6.46157	-3.353542	-0.206213
H	-7.748765	-4.142337	0.699049
H	10.756008	-0.64377	0.643584
H	10.724931	-2.116388	-0.336895
H	11.09003	-0.562899	-1.100553
H	-2.628473	0.641066	3.861377
H	-3.088618	-1.064852	3.315392

Cartesian coordinates for conformer 2-31 after optimization at the PCM/B3LYP/6-31G* level of theory. Number of imaginary frequencies = 0. SCF Energy (PCM/mPW1PW91/6-31+G**) = -2695.1117943.

Atom	X	Y	Z
O	-9.090621	0.507548	-0.229151
C	-8.817265	-0.361361	-1.239038
C	-8.927321	-1.816979	-0.763421
O	-8.530705	-0.001632	-2.370216
C	-7.767923	-2.677475	-1.297652
C	-6.362759	-2.15293	-0.944901
C	-10.274569	-2.371493	-1.221769
O	-8.945961	-1.808379	0.679033
C	-5.300297	-3.166286	-1.383339
C	-3.912456	-2.725805	-0.944009
C	-3.896456	-2.441417	0.557004
C	-5.045783	-1.457304	0.940788
O	-6.313411	-1.938174	0.470246
O	-4.705689	-0.184751	0.361421
C	-5.437571	0.920603	0.911015
C	-5.088017	1.092767	2.401422
C	-5.191156	-0.215822	3.136593
C	-5.182547	-1.368306	2.444215
C	-5.136793	2.206454	0.093746
C	-3.665599	2.56767	0.09957
C	-5.598297	2.056397	-1.359045
C	-3.161104	3.703402	0.61189
C	-1.705672	4.080094	0.654728
C	-0.888633	3.087854	0.013516
C	0.252878	2.809803	0.834835
C	0.272598	3.896841	1.905503
C	-1.18898	4.216919	2.078646
C	0.101083	1.401599	1.440943
C	0.226597	0.314528	0.372635
C	1.504196	0.525336	-0.434059
C	1.517547	1.951395	-0.984882
O	1.464991	2.913861	0.079469
O	1.574457	-0.439366	-1.484989
C	2.758132	-0.341983	-2.315941
C	2.910948	1.071431	-2.844991
C	2.809562	2.145908	-1.787332
C	3.990362	-0.908979	-1.548448
C	5.231994	-1.046229	-2.446328
C	6.537453	-1.531174	-1.766266
C	6.970882	-0.686748	-0.538599
C	6.47633	-3.024742	-1.408655
O	8.222522	-1.208987	-0.061177
C	8.649358	-0.656185	1.189763
C	8.809664	0.868563	1.065245
C	7.558872	1.521927	0.483146
C	7.110511	0.828111	-0.811445
C	10.017909	-1.269488	1.540681
C	9.902051	-2.75483	1.870852
C	8.835186	-2.970167	2.934194
C	7.532084	-2.305722	2.519033

O	7.716165	-0.916814	2.246575
H	2.345381	0.377138	0.25479
H	6.220477	-0.836049	0.247956
H	-6.194822	-1.197679	-1.455731
O	-2.605208	-1.9106	0.891025
H	-6.509956	0.704591	0.815345
H	-1.561085	5.017792	0.105264
H	0.668976	2.124176	-1.661872
O	2.824575	3.453103	-2.365679
H	2.551806	-1.026485	-3.14917
O	3.640779	-2.193231	-1.017433
C	8.082749	1.148499	-1.955134
C	-5.284645	-0.155893	4.630571
C	3.071245	1.319505	-4.158288
H	-9.299871	-0.033933	0.571805
H	-7.843366	-2.779348	-2.387605
H	-7.87569	-3.681535	-0.864766
H	-10.355562	-2.386377	-2.313869
H	-10.426829	-3.389063	-0.845038
H	-11.100715	-1.765452	-0.831245
H	-8.008377	-1.888993	0.968304
H	-5.510253	-4.143066	-0.928866
H	-5.323393	-3.298902	-2.470738
H	-3.604979	-1.832706	-1.503079
H	-3.171927	-3.497173	-1.186293
H	-4.017791	-3.392413	1.0904
H	-5.764461	1.833662	2.843184
H	-4.061954	1.455753	2.531306
H	-5.27153	-2.315097	2.971368
H	-5.714245	3.027984	0.538852
H	-2.992848	1.834213	-0.343664
H	-5.415498	2.976781	-1.925343
H	-5.079063	1.239526	-1.872326
H	-6.672491	1.853882	-1.400414
H	-3.84039	4.435952	1.044064
H	0.805697	4.784744	1.541361
H	0.761638	3.587801	2.834387
H	-1.36373	5.212664	2.497247
H	-1.654995	3.484825	2.749837
H	0.888302	1.237979	2.187509
H	-0.866753	1.289693	1.942708
H	-0.645605	0.329022	-0.293203
H	0.233367	-0.678493	0.837111
H	3.680228	2.098264	-1.122844
H	4.217558	-0.271309	-0.690305
H	5.43913	-0.082406	-2.923405
H	4.999596	-1.743335	-3.262598
H	7.331162	-1.460475	-2.522831
H	5.852687	-3.209764	-0.52969
H	6.082319	-3.613779	-2.243411
H	7.476817	-3.410922	-1.184537
H	9.660197	1.103471	0.413799
H	9.016847	1.3133	2.046191
H	7.742876	2.588424	0.307587
H	6.746791	1.464391	1.219311
H	6.133774	1.244071	-1.083802
H	10.720835	-1.145308	0.708212
H	10.438584	-0.757837	2.416004
H	9.642157	-3.318333	0.966601
H	10.866496	-3.137643	2.222164
H	9.181775	-2.527579	3.876097
H	8.679769	-4.039952	3.108005
H	6.803145	-2.38617	3.331907
H	7.095926	-2.802747	1.646246
H	-2.657488	-0.957825	0.676163
H	2.479679	4.04645	-1.671169

H	2.691386	-2.147268	-0.785892
H	9.057961	0.672277	-1.814901
H	7.684742	0.812284	-2.917218
H	8.242565	2.229197	-2.035455
H	-6.18289	0.391656	4.933415
H	-5.333484	-1.153883	5.078518
H	-4.409224	0.354402	5.044822
H	3.158821	2.335295	-4.535209
H	3.115849	0.521575	-4.893326

Cartesian coordinates for conformer 2-32 after optimization at the PCM/B3LYP/6-31G* level of theory. Number of imaginary frequencies = 0. SCF Energy (PCM/mPW1PW91/6-31+G**) = -2695.1142333.

Atom	X	Y	Z
O	-8.823811	3.131713	0.872938
C	-9.313243	2.974127	-0.386011
C	-10.648404	2.21672	-0.371518
O	-8.745234	3.396227	-1.38114
C	-10.710157	1.157051	-1.486711
C	-9.566793	0.124161	-1.457612
C	-11.778601	3.233108	-0.521712
O	-10.79792	1.611986	0.929428
C	-9.820207	-0.964556	-2.505961
C	-8.772936	-2.063675	-2.416547
C	-8.691861	-2.602641	-0.989378
C	-8.492598	-1.433708	0.025966
O	-9.509362	-0.43397	-0.140109
O	-7.172819	-0.904544	-0.204702
C	-6.692986	-0.068921	0.859595
C	-6.524048	-0.90173	2.14416
C	-7.7581	-1.715431	2.427062
C	-8.649907	-1.934006	1.444382
C	-5.369221	0.622468	0.434006
C	-4.254161	-0.361066	0.131513
C	-5.58262	1.509336	-0.796827
C	-3.081879	-0.388329	0.788692
C	-1.928978	-1.319046	0.553139
C	-0.731093	-0.528514	0.474097
C	0.18001	-1.150433	-0.445564
C	-0.478045	-2.459085	-0.882961
C	-1.946303	-2.164698	-0.710311
C	0.413805	-0.200193	-1.634157
C	1.208306	1.038325	-1.217869
C	2.477061	0.616151	-0.482748
C	2.097764	-0.304944	0.677233
O	1.415289	-1.476162	0.202321
O	3.174023	1.775205	-0.023533
C	4.403421	1.496707	0.691044
C	4.147035	0.515234	1.818722
C	3.382684	-0.720389	1.404205
C	5.519978	1.084015	-0.312878
C	6.897728	0.95058	0.355691
C	8.075132	0.67114	-0.610479
C	9.430052	0.566059	0.151435
C	7.8298	-0.611395	-1.424807
O	10.460424	0.313162	-0.818949
C	11.738843	-0.00196	-0.256714
C	12.226357	1.160436	0.623144
C	11.176035	1.562338	1.654042
C	9.805607	1.80114	1.003209
C	12.735331	-0.194789	-1.415314
C	12.423697	-1.450979	-2.223735
C	12.311174	-2.652395	-1.296801
C	11.33453	-2.361563	-0.16844
O	11.700994	-1.185484	0.551888

H	3.101852	0.077269	-1.20615
H	9.360398	-0.305356	0.816797
H	-8.617421	0.628521	-1.671502
O	-7.616505	-3.551394	-0.937402
H	-7.43634	0.719635	1.038106
H	-1.82209	-1.966467	1.432746
H	1.45177	0.212465	1.400573
O	3.05337	-1.534923	2.532091
H	4.688688	2.470427	1.11042
O	5.602345	2.077973	-1.345174
C	9.813104	3.106355	0.196524
C	-7.916683	-2.256811	3.814888
C	4.538035	0.772751	3.080672
H	-9.4722	2.713025	1.491814
H	-10.721805	1.643382	-2.470283
H	-11.660229	0.616796	-1.375309
H	-11.722659	3.762124	-1.479039
H	-12.756374	2.74411	-0.448228
H	-11.741176	3.981398	0.278897
H	-10.405221	0.711514	0.863289
H	-10.806514	-1.416422	-2.339409
H	-9.823729	-0.53252	-3.512882
H	-7.794653	-1.680732	-2.734073
H	-9.012949	-2.877694	-3.110866
H	-9.62327	-3.139273	-0.769989
H	-6.310765	-0.22722	2.981536
H	-5.687057	-1.603699	2.056506
H	-9.549935	-2.508195	1.650485
H	-5.0591	1.277151	1.259656
H	-4.452955	-1.06829	-0.671019
H	-4.657572	2.02983	-1.069937
H	-5.909859	0.930756	-1.667872
H	-6.339585	2.272024	-0.592601
H	-2.916304	0.327795	1.592772
H	-0.182966	-3.281317	-0.21844
H	-0.216991	-2.761856	-1.901624
H	-2.557756	-3.06639	-0.611848
H	-2.305326	-1.596425	-1.576487
H	0.982814	-0.722421	-2.413768
H	-0.538421	0.121529	-2.071164
H	0.598629	1.68655	-0.575697
H	1.464263	1.641477	-2.096952
H	4.006391	-1.342413	0.751053
H	5.250404	0.1446	-0.802915
H	6.865066	0.15935	1.11411
H	7.095006	1.891847	0.88072
H	8.151425	1.487308	-1.339641
H	7.683525	-1.473638	-0.765729
H	6.94761	-0.51576	-2.064676
H	8.670509	-0.832428	-2.090179
H	12.453965	2.033168	-0.001135
H	13.148834	0.883591	1.147895
H	11.508371	2.455374	2.19618
H	11.081793	0.763768	2.401123
H	9.081207	1.918577	1.817792
H	12.71835	0.671876	-2.086817
H	13.752994	-0.290995	-1.015247
H	11.483384	-1.317396	-2.772113
H	13.208629	-1.622224	-2.968501
H	13.300302	-2.863723	-0.872095
H	11.994883	-3.538547	-1.856906
H	11.335649	-3.194567	0.542129
H	10.31135	-2.263883	-0.546336
H	-6.809787	-3.020187	-0.786
H	2.355877	-2.140882	2.216379
H	4.685458	2.372629	-1.513676

H	10.435898	3.033494	-0.700432
H	8.802013	3.378123	-0.121798
H	10.191251	3.935353	0.804528
H	-7.96464	-1.437482	4.539146
H	-8.829847	-2.851536	3.92091
H	-7.067701	-2.898826	4.070648
H	4.334121	0.075071	3.888898
H	5.066355	1.683899	3.344584

Cartesian coordinates for conformer 2-33 after optimization at the PCM/B3LYP/6-31G* level of theory. Number of imaginary frequencies = 0. SCF Energy (PCM/mPW1PW91/6-31+G**) = -2695.10875627.

Atom	X	Y	Z
O	2.743012	1.442299	1.595374
C	3.963115	1.082444	2.04713
C	3.884759	0.004372	3.143343
O	4.979324	1.564306	1.572283
C	2.944928	-1.143438	2.714619
C	3.291955	-1.756267	1.343515
C	3.377373	0.659402	4.429457
O	5.173395	-0.535084	3.453907
C	2.325294	-2.899661	1.025304
C	2.70384	-3.56513	-0.287111
C	4.160773	-4.016029	-0.241248
C	5.099108	-2.83788	0.180163
O	4.645851	-2.215653	1.387928
O	5.12745	-1.91243	-0.924281
C	6.2336	-0.984634	-0.891833
C	7.550958	-1.774785	-1.024822
C	7.609	-2.92862	-0.064822
C	6.473826	-3.381436	0.491702
C	6.030169	0.034572	-2.057751
C	4.828396	0.928161	-1.765676
C	7.305456	0.818746	-2.397684
C	4.790558	2.273851	-1.751131
C	3.605826	3.150824	-1.431764
C	2.52719	2.35684	-0.899066
C	1.416883	2.420531	-1.81397
C	1.98699	2.934038	-3.132932
C	3.067556	3.874947	-2.655363
C	0.779026	1.034896	-1.983573
C	0.044219	0.584243	-0.723863
C	-0.931746	1.670298	-0.281784
C	-0.17202	2.98622	-0.122604
O	0.461353	3.383569	-1.35027
O	-1.546283	1.28206	0.944573
C	-2.506366	2.226124	1.458423
C	-1.895954	3.616168	1.563541
C	-1.159548	4.071912	0.324126
C	-3.886024	2.18838	0.723813
C	-4.437271	0.750927	0.663698
C	-5.823566	0.591128	-0.00588
C	-6.288004	-0.896493	-0.020171
C	-6.880361	1.463983	0.693337
O	-7.56782	-0.94408	-0.673461
C	-8.223866	-2.215077	-0.596385
C	-7.347185	-3.29811	-1.245817
C	-5.932485	-3.292386	-0.674545
C	-5.315817	-1.88612	-0.703165
C	-9.5471	-2.117953	-1.378781
C	-10.548669	-1.202033	-0.681413
C	-10.71734	-1.624542	0.770377
C	-9.362795	-1.724253	1.453887
O	-8.487154	-2.609271	0.756517
H	-1.685741	1.752215	-1.071736

H	-6.420309	-1.205354	1.025679
H	3.206087	-0.990118	0.564645
O	4.498784	-4.538976	-1.53489
H	6.216743	-0.448802	0.066854
H	3.908395	3.865525	-0.658322
H	0.601533	2.900974	0.652387
O	-0.436234	5.286214	0.566881
H	-2.703815	1.867613	2.477935
O	-3.793822	2.707467	-0.606744
C	-4.961006	-1.483526	-2.140773
C	8.95631	-3.517374	0.222281
C	-1.984545	4.340097	2.695296
H	2.895525	1.981081	0.780402
H	1.904335	-0.795264	2.693455
H	3.010884	-1.935491	3.473054
H	2.376957	1.087549	4.304804
H	3.346312	-0.064393	5.251743
H	4.050641	1.465318	4.745276
H	5.448628	-1.055764	2.670494
H	2.362791	-3.652632	1.822747
H	1.296033	-2.527597	0.970814
H	2.545268	-2.873107	-1.12418
H	2.051249	-4.424278	-0.482816
H	4.239696	-4.838124	0.481399
H	8.396999	-1.105053	-0.833328
H	7.65388	-2.176755	-2.040428
H	6.512388	-4.197828	1.208806
H	5.755938	-0.538202	-2.955196
H	3.914446	0.378869	-1.546038
H	7.127121	1.537608	-3.205281
H	7.684884	1.363139	-1.526025
H	8.098435	0.154883	-2.756319
H	5.695249	2.838822	-1.964792
H	1.246275	3.440455	-3.75933
H	2.442594	2.122441	-3.712817
H	2.616182	4.827774	-2.352029
H	3.820333	4.091643	-3.419472
H	0.054682	1.067173	-2.807593
H	1.538561	0.286583	-2.236813
H	0.756439	0.362305	0.079616
H	-0.494426	-0.352506	-0.910663
H	-1.866932	4.302219	-0.478953
H	-4.568012	2.840963	1.280021
H	-4.480108	0.339767	1.680261
H	-3.716945	0.146375	0.102546
H	-5.771624	0.942555	-1.043768
H	-6.955732	1.213598	1.756865
H	-6.641147	2.527999	0.608116
H	-7.870248	1.33441	0.244223
H	-7.286544	-3.132142	-2.32829
H	-7.786662	-4.290989	-1.090829
H	-5.304723	-4.002014	-1.226302
H	-5.962707	-3.647631	0.363536
H	-4.381383	-1.93497	-0.131729
H	-9.368468	-1.74129	-2.392944
H	-9.999579	-3.114366	-1.464623
H	-11.513854	-1.238255	-1.198565
H	-10.198417	-0.163743	-0.726444
H	-11.208152	-2.605245	0.79747
H	-11.361863	-0.916746	1.301809
H	-9.490662	-2.120371	2.466471
H	-8.895868	-0.738529	1.551721
H	4.771117	-3.762891	-2.06405
H	0.128416	5.401806	-0.220393
H	-4.700266	2.893356	-0.909888
H	-5.851651	-1.310616	-2.752791

H	-4.363466	-0.566798	-2.157105
H	-4.363745	-2.263643	-2.625125
H	9.613268	-2.764319	0.668894
H	8.894973	-4.36219	0.916218
H	9.417271	-3.877397	-0.703066
H	-1.532361	5.325603	2.770526
H	-2.504956	3.973588	3.574341

Cartesian coordinates for conformer 2-34 after optimization at the PCM/B3LYP/6-31G* level of theory. Number of imaginary frequencies = 0. SCF Energy (PCM/mPW1PW91/6-31+G**) = -2695.11149054.

Atom	X	Y	Z
O	-6.712649	1.898819	1.961029
C	-5.725552	2.473586	1.2225
C	-6.244622	2.908147	-0.155829
O	-4.589672	2.636949	1.638965
C	-5.27561	2.506621	-1.283799
C	-5.001052	0.994199	-1.402274
C	-6.446294	4.422835	-0.128486
O	-7.547158	2.318501	-0.346189
C	-4.161113	0.714299	-2.654017
C	-3.999266	-0.779845	-2.890651
C	-5.364135	-1.468669	-2.904429
C	-6.165178	-1.101034	-1.618242
O	-6.264752	0.323899	-1.474681
O	-5.479349	-1.722408	-0.518269
C	-6.206535	-1.656134	0.720309
C	-7.476869	-2.519655	0.594292
C	-8.202997	-2.265559	-0.702603
C	-7.588259	-1.600329	-1.697423
C	-5.273348	-2.14113	1.864497
C	-3.959937	-1.375637	1.829921
C	-5.973533	-2.10892	3.227873
C	-3.52243	-0.439514	2.689651
C	-2.218988	0.318516	2.595063
C	-1.484121	-0.092516	1.428371
C	-0.241893	-0.672165	1.840071
C	-0.425247	-1.051394	3.304286
C	-1.316659	0.065467	3.796256
C	0.085654	-1.892787	0.97044
C	0.412872	-1.491082	-0.46757
C	1.487728	-0.408148	-0.46931
C	1.028616	0.750283	0.416574
O	0.805488	0.308253	1.764931
O	1.732663	0.023688	-1.808658
C	2.746039	1.052755	-1.935193
C	2.421504	2.220235	-1.023027
C	2.119034	1.82846	0.404821
C	4.161429	0.427232	-1.748277
C	5.291292	1.413066	-2.091655
C	6.743395	0.918953	-1.869309
C	7.029763	0.400578	-0.434957
C	7.161528	-0.121257	-2.920735
O	8.421172	0.045802	-0.361574
C	8.789876	-0.635288	0.843748
C	8.480018	0.247884	2.064426
C	7.040104	0.754574	2.043915
C	6.688017	1.398821	0.695194
C	10.306537	-0.89965	0.798284
C	10.667994	-1.9262	-0.271256
C	9.825052	-3.180643	-0.094941
C	8.349107	-2.821762	-0.028558
O	8.086703	-1.874047	1.006068
H	2.399344	-0.861019	-0.059249
H	6.439978	-0.513137	-0.28886

H	-4.465658	0.653196	-0.509173
O	-5.143498	-2.881939	-3.018121
H	-6.484755	-0.61191	0.911874
H	-2.439811	1.386121	2.501629
H	0.102401	1.201003	0.032885
O	1.691489	2.953074	1.176223
H	2.668038	1.371239	-2.98288
O	4.261189	-0.726412	-2.592458
C	7.402131	2.749044	0.544009
C	-9.603273	-2.788164	-0.811757
C	2.36592	3.482939	-1.48614
H	-7.533664	1.918093	1.409775
H	-4.319673	3.032188	-1.165345
H	-5.719861	2.843365	-2.230807
H	-5.502865	4.954055	0.036471
H	-6.887295	4.77546	-1.067492
H	-7.139235	4.711183	0.670889
H	-7.402659	1.473738	-0.830685
H	-4.65481	1.146442	-3.534052
H	-3.17685	1.187981	-2.566672
H	-3.36018	-1.217327	-2.112896
H	-3.47903	-0.961835	-3.838684
H	-5.910288	-1.140426	-3.797392
H	-8.145672	-2.309186	1.436292
H	-7.21518	-3.584525	0.628941
H	-8.119009	-1.396338	-2.623729
H	-5.000959	-3.187341	1.66523
H	-3.326897	-1.629325	0.97924
H	-5.282184	-2.379403	4.033884
H	-6.384173	-1.116951	3.445801
H	-6.798085	-2.828084	3.263718
H	-4.151825	-0.139164	3.524622
H	0.514543	-1.105705	3.861869
H	-0.953121	-2.007205	3.404718
H	-0.709933	0.957794	3.994959
H	-1.850286	-0.18341	4.718642
H	0.955313	-2.415671	1.387994
H	-0.755647	-2.595447	0.958806
H	-0.488513	-1.128569	-0.978272
H	0.752859	-2.361912	-1.040229
H	3.028318	1.455163	0.890111
H	4.275553	0.069683	-0.721844
H	5.155933	2.331668	-1.510871
H	5.193731	1.705468	-3.145812
H	7.400307	1.779833	-2.055625
H	6.712133	-1.099741	-2.729494
H	6.87644	0.200199	-3.927808
H	8.248665	-0.25717	-2.920314
H	9.153351	1.113522	2.08062
H	8.644413	-0.311184	2.993495
H	6.882891	1.463705	2.865067
H	6.359465	-0.08691	2.226565
H	5.609818	1.595526	0.698498
H	10.853768	0.028979	0.597586
H	10.643476	-1.286203	1.768848
H	10.496343	-1.50203	-1.267934
H	11.732823	-2.175971	-0.20729
H	10.118719	-3.674311	0.839533
H	10.010714	-3.883666	-0.913668
H	7.762177	-3.719097	0.192556
H	7.993367	-2.434122	-0.988875
H	-4.965969	-3.18929	-2.106587
H	1.269596	2.5757	1.971684
H	3.368581	-1.125259	-2.621661
H	8.483397	2.633798	0.42034
H	7.026095	3.299355	-0.323763

H	7.227365	3.37954	1.422424
H	-10.240524	-2.330071	-0.048729
H	-10.045739	-2.573994	-1.790215
H	-9.614573	-3.873547	-0.669688
H	2.112435	4.313996	-0.832782
H	2.567578	3.724022	-2.525454

Cartesian coordinates for conformer 2-35 after optimization at the PCM/B3LYP/6-31G* level of theory. Number of imaginary frequencies = 0. SCF Energy (PCM/mPW1PW91/6-31+G**) = -2695.10604188.

Atom	X	Y	Z
O	-9.48442	0.091691	-0.388399
C	-9.211981	-0.894726	-1.284144
C	-9.259569	-2.277332	-0.61858
O	-8.970547	-0.679418	-2.461718
C	-8.088069	-3.166366	-1.073847
C	-6.691263	-2.560434	-0.837533
C	-10.600311	-2.926506	-0.955671
O	-9.237002	-2.078951	0.810137
C	-5.609624	-3.591215	-1.177369
C	-4.225122	-3.057595	-0.844302
C	-4.174146	-2.582094	0.606933
C	-5.34378	-1.591044	0.901037
O	-6.608145	-2.164356	0.535985
O	-5.062079	-0.39202	0.156728
C	-5.825573	0.747769	0.578678
C	-5.45481	1.122825	2.026092
C	-5.483087	-0.084142	2.924175
C	-5.442562	-1.315065	2.384355
C	-5.592618	1.926976	-0.405005
C	-4.142084	2.360214	-0.458605
C	-6.047359	1.561182	-1.820949
C	-3.705685	3.600553	-0.177898
C	-2.268316	4.064332	-0.200044
C	-1.394339	2.95242	-0.452873
C	-0.569645	2.730468	0.698199
C	-1.242141	3.475088	1.845842
C	-1.833775	4.666543	1.129023
C	-0.455619	1.22906	0.988177
C	0.341652	0.506603	-0.095684
C	1.6825	1.205067	-0.302189
C	1.435889	2.685847	-0.584556
O	0.72377	3.317429	0.492404
O	2.371224	0.59695	-1.391314
C	3.675983	1.141615	-1.658479
C	3.614678	2.65466	-1.817371
C	2.794026	3.373545	-0.767786
C	4.780454	0.641645	-0.671207
C	4.694084	-0.883512	-0.472267
C	5.870059	-1.557896	0.278431
C	7.26646	-1.296282	-0.346083
C	5.868978	-1.204081	1.774153
O	8.232893	-2.053041	0.402644
C	9.594856	-1.759653	0.068563
C	9.845928	-2.042738	-1.422371
C	8.816325	-1.349674	-2.310898
C	7.381996	-1.643583	-1.847551
C	10.501542	-2.682696	0.904288
C	10.454739	-2.327637	2.387463
C	10.737916	-0.84472	2.576484
C	9.82023	-0.014242	1.693609
O	9.932005	-0.388969	0.321026
H	2.255396	1.077482	0.623526
H	7.495379	-0.231508	-0.212218
H	-6.569176	-1.674438	-1.471206

O	-2.891715	-1.975442	0.825372
H	-6.890292	0.482312	0.537091
H	-2.152149	4.791093	-1.011354
H	0.861193	2.821159	-1.511732
O	2.586852	4.745978	-1.122538
H	3.938732	0.710196	-2.634062
O	4.68816	1.279368	0.604154
C	7.004744	-3.097942	-2.1613
C	-5.542204	0.16299	4.400669
C	4.215285	3.273759	-2.851319
H	-9.652673	-0.345299	0.483062
H	-8.191002	-3.411733	-2.1385
H	-8.150926	-4.107995	-0.511436
H	-10.712173	-3.087832	-2.033031
H	-10.7078	-3.889404	-0.443976
H	-11.434527	-2.298505	-0.62099
H	-8.289562	-2.095824	1.077182
H	-5.774086	-4.506644	-0.594747
H	-5.660218	-3.863628	-2.237459
H	-3.964192	-2.235174	-1.52273
H	-3.466799	-3.831854	-1.010978
H	-4.24745	-3.459759	1.261119
H	-6.154959	1.88489	2.387619
H	-4.443965	1.542567	2.08288
H	-5.478173	-2.18894	3.030339
H	-6.210366	2.769754	-0.06681
H	-3.425945	1.591755	-0.747408
H	-5.919595	2.408326	-2.504499
H	-5.482274	0.715413	-2.228272
H	-7.108024	1.293625	-1.825351
H	-4.433233	4.367277	0.083015
H	-0.548951	3.772068	2.638663
H	-2.050238	2.880293	2.288007
H	-1.05041	5.414903	0.955402
H	-2.637758	5.151861	1.690583
H	0.053176	1.079134	1.948759
H	-1.450222	0.77425	1.059788
H	-0.222733	0.485328	-1.036706
H	0.501288	-0.542042	0.181722
H	3.333636	3.395609	0.184538
H	5.754886	0.909442	-1.094675
H	4.591719	-1.360783	-1.453902
H	3.773342	-1.120828	0.077241
H	5.683777	-2.640445	0.247308
H	6.218143	-0.18276	1.951315
H	4.86624	-1.306429	2.201761
H	6.529325	-1.876197	2.333009
H	9.798073	-3.122031	-1.611596
H	10.847997	-1.704433	-1.712847
H	8.957036	-1.656757	-3.353967
H	8.98677	-0.265905	-2.280917
H	6.712659	-0.996473	-2.426628
H	10.202826	-3.730609	0.781089
H	11.540637	-2.586596	0.564035
H	9.46743	-2.571261	2.798095
H	11.188768	-2.924576	2.939718
H	11.780934	-0.646873	2.300115
H	10.614325	-0.563304	3.627359
H	10.096137	1.042612	1.76793
H	8.777898	-0.097461	2.018974
H	-2.978526	-1.064184	0.480766
H	1.873815	5.054878	-0.531504
H	5.167873	2.124833	0.534082
H	7.556319	-3.81123	-1.541061
H	5.937372	-3.273496	-1.997792
H	7.210654	-3.332932	-3.211196

H	-6.455116	0.709266	4.658202
H	-5.537842	-0.770445	4.973124
H	-4.679256	0.755797	4.720462
H	4.152745	4.351753	-2.977036
H	4.776943	2.728207	-3.603133

Cartesian coordinates for conformer 2-36 after optimization at the PCM/B3LYP/6-31G* level of theory. Number of imaginary frequencies = 0. SCF Energy (PCM/mPW1PW91/6-31+G**) = -2695.11131348.

Atom	X	Y	Z
O	2.554864	-1.3259	-0.063316
C	3.018699	-2.412447	-0.736411
C	3.410135	-3.539482	0.232102
O	3.093841	-2.458831	-1.954414
C	4.708259	-4.245912	-0.201189
C	5.915713	-3.307899	-0.377301
C	2.250461	-4.531177	0.304622
O	3.54807	-2.958875	1.545041
C	7.179503	-4.115947	-0.686149
C	8.393081	-3.202204	-0.779886
C	8.511314	-2.338792	0.475842
C	7.160991	-1.615963	0.778318
O	6.079851	-2.560666	0.834625
O	6.958501	-0.642393	-0.264305
C	5.916517	0.298146	0.044705
C	6.362617	1.180683	1.223831
C	6.862756	0.328256	2.363348
C	7.204095	-0.953863	2.13788
C	5.555904	1.085557	-1.242456
C	4.281342	1.881311	-1.043428
C	6.741246	1.880851	-1.806716
C	4.098412	3.20626	-1.183194
C	2.799714	3.951473	-0.973714
C	1.780819	3.056633	-0.501261
C	0.736436	2.975637	-1.479845
C	1.337208	3.50877	-2.775938
C	2.276201	4.575073	-2.259968
C	0.256897	1.526329	-1.629497
C	-0.472374	1.04239	-0.377486
C	-1.578673	2.028853	-0.013785
C	-0.974455	3.426681	0.114253
O	-0.34578	3.843836	-1.109916
O	-2.193992	1.623347	1.207512
C	-3.268752	2.475385	1.648577
C	-2.816464	3.926964	1.715907
C	-2.089998	4.413009	0.48203
C	-4.608119	2.25799	0.871608
C	-4.994164	0.766606	0.849296
C	-6.330255	0.429334	0.144462
C	-6.624508	-1.100808	0.170757
C	-7.501166	1.199869	0.779402
O	-7.869082	-1.314115	-0.516851
C	-8.380695	-2.647767	-0.412828
C	-7.367743	-3.645557	-0.997585
C	-5.982033	-3.461464	-0.386541
C	-5.525921	-1.996257	-0.44829
C	-9.680508	-2.726152	-1.235735
C	-10.800435	-1.906916	-0.600923
C	-10.967981	-2.298152	0.859839
C	-9.633677	-2.222349	1.584964
O	-8.642839	-3.025439	0.945175
H	-2.307529	1.996116	-0.830584
H	-6.755571	-1.388172	1.222873
H	5.713361	-2.61487	-1.202442
O	9.586567	-1.410714	0.272974

H	5.025784	-0.270735	0.343791
H	2.964024	4.718011	-0.208507
H	-0.224496	3.456524	0.91759
O	-1.514843	5.709845	0.694642
H	-3.464328	2.136597	2.675064
O	-4.52652	2.737718	-0.474219
C	-5.171408	-1.603275	-1.888522
C	6.955926	0.977262	3.710461
C	-3.020704	4.67849	2.814218
H	2.600512	-1.536071	0.902426
H	4.544957	-4.79172	-1.138885
H	4.959185	-4.984622	0.572287
H	2.057277	-4.998977	-0.666617
H	2.455061	-5.318592	1.038567
H	1.328629	-4.033173	0.627592
H	4.491581	-2.692431	1.633572
H	7.357281	-4.846159	0.113659
H	7.059653	-4.67426	-1.621286
H	8.317197	-2.565403	-1.670645
H	9.306776	-3.793214	-0.914721
H	8.784824	-2.990331	1.315209
H	5.522616	1.799568	1.558073
H	7.181416	1.847459	0.932865
H	7.543427	-1.576312	2.962147
H	5.323618	0.333096	-2.010469
H	3.421562	1.270113	-0.763871
H	6.486471	2.314153	-2.780654
H	7.042661	2.700792	-1.147506
H	7.612059	1.234405	-1.958495
H	4.936016	3.840076	-1.46489
H	0.59223	3.91401	-3.4672
H	1.916576	2.735281	-3.294184
H	1.705884	5.482183	-2.024346
H	3.054211	4.849253	-2.979008
H	-0.433494	1.455796	-2.479598
H	1.102033	0.857346	-1.827497
H	0.229939	0.93573	0.459106
H	-0.895939	0.044617	-0.542876
H	-2.788048	4.53159	-0.352986
H	-5.377937	2.849047	1.380125
H	-5.026185	0.38819	1.878783
H	-4.192257	0.227846	0.333402
H	-6.282783	0.75002	-0.903439
H	-8.454641	0.944726	0.30572
H	-7.584024	0.977783	1.848438
H	-7.3796	2.280669	0.662316
H	-7.290921	-3.508835	-2.083115
H	-7.69835	-4.676118	-0.819783
H	-5.261221	-4.113474	-0.893892
H	-6.006118	-3.783865	0.662365
H	-4.611045	-1.92071	0.151382
H	-9.512296	-2.364983	-2.257397
H	-10.01577	-3.76962	-1.298491
H	-10.567325	-0.837636	-0.673524
H	-11.738061	-2.068899	-1.144025
H	-11.346751	-3.326442	0.908235
H	-11.704371	-1.650371	1.346578
H	-9.749329	-2.597254	2.607049
H	-9.282886	-1.187714	1.66059
H	9.195647	-0.66394	-0.223004
H	-0.900189	5.833468	-0.053351
H	-5.435987	2.807655	-0.814338
H	-6.055386	-1.551814	-2.531675
H	-4.679503	-0.626174	-1.920379
H	-4.475323	-2.326513	-2.327036
H	5.967897	1.315691	4.038534

H	7.344143	0.290812	4.470131
H	7.62468	1.842929	3.66854
H	-2.679279	5.70913	2.862649
H	-3.528272	4.290357	3.691364

Cartesian coordinates for conformer 2-37 after optimization at the PCM/B3LYP/6-31G* level of theory. Number of imaginary frequencies = 0. SCF Energy (PCM/mPW1PW91/6-31+G**) = -2695.11375193.

Atom	X	Y	Z
O	-9.46913	-1.07629	-2.34136
C	-9.984992	-1.395679	-1.124089
C	-10.77268	-0.219783	-0.529658
O	-9.830988	-2.487599	-0.599375
C	-10.467833	-0.03197	0.967522
C	-8.975762	0.16046	1.300326
C	-12.261332	-0.475687	-0.755653
O	-10.431647	0.963205	-1.281543
C	-8.81033	0.499341	2.785395
C	-7.358264	0.808192	3.115464
C	-6.82169	1.892545	2.18249
C	-7.08381	1.511539	0.691672
O	-8.471165	1.215051	0.473552
O	-6.242516	0.380963	0.40505
C	-6.053166	0.130835	-0.994521
C	-5.314282	1.314883	-1.645429
C	-5.964644	2.622299	-1.282364
C	-6.779177	2.682985	-0.214406
C	-5.304165	-1.2193	-1.182671
C	-3.89854	-1.210331	-0.600347
C	-6.165456	-2.384682	-0.675211
C	-3.487186	-1.785397	0.54417
C	-2.086266	-1.767536	1.107378
C	-1.201174	-1.054124	0.228551
C	-0.180683	-1.947919	-0.231339
C	-0.729855	-3.351719	-0.008358
C	-1.51214	-3.169131	1.271766
C	0.128268	-1.684511	-1.710637
C	0.818323	-0.335878	-1.913167
C	2.026547	-0.228559	-0.987727
C	1.578179	-0.494604	0.449535
O	0.99988	-1.803647	0.575221
O	2.618287	1.064616	-1.119243
C	3.782915	1.283314	-0.284981
C	3.463382	0.958352	1.161628
C	2.804186	-0.385844	1.364393
C	5.011964	0.536505	-0.882391
C	6.322429	0.873776	-0.153067
C	7.600889	0.264921	-0.779962
C	8.879048	0.672323	0.012311
C	7.499885	-1.268496	-0.861469
O	10.009884	0.069813	-0.640182
C	11.24193	0.189877	0.078934
C	11.58388	1.67355	0.290516
C	10.420056	2.432837	0.920161
C	9.109004	2.194403	0.156634
C	12.354071	-0.456321	-0.768471
C	12.184716	-1.969961	-0.861114
C	12.043085	-2.564497	0.532238
C	10.94886	-1.846537	1.306073
O	11.185423	-0.440536	1.365484
H	2.744806	-0.993486	-1.308754
H	8.788736	0.238705	1.017667
H	-8.432364	-0.763104	1.070185
O	-5.422516	2.055613	2.457818
H	-7.042823	0.030549	-1.460829

H	-2.110132	-1.245011	2.07005
H	0.841348	0.250736	0.780214
O	2.405311	-0.573729	2.724302
H	3.97776	2.3592	-0.384423
O	5.146314	0.901306	-2.264144
C	9.129516	2.92837	-1.190679
C	-5.64476	3.808037	-2.140271
C	3.713463	1.833413	2.153315
H	-9.742646	-0.146162	-2.53825
H	-10.850495	-0.885303	1.541509
H	-11.010509	0.861743	1.305044
H	-12.599627	-1.379441	-0.237937
H	-12.860867	0.372863	-0.40769
H	-12.480002	-0.597557	-1.823194
H	-9.662706	1.371049	-0.821293
H	-9.413899	1.381801	3.033335
H	-9.167501	-0.327831	3.408912
H	-6.7497	-0.101291	3.030518
H	-7.264029	1.132199	4.158753
H	-7.32442	2.836825	2.425852
H	-5.309752	1.17855	-2.733008
H	-4.273262	1.371902	-1.308733
H	-7.260088	3.623117	0.044626
H	-5.181182	-1.380467	-2.263014
H	-3.162283	-0.675988	-1.199859
H	-5.649864	-3.342607	-0.805988
H	-6.426382	-2.277833	0.382762
H	-7.099024	-2.440107	-1.243621
H	-4.210938	-2.305633	1.16792
H	0.048447	-4.114528	0.08872
H	-1.414728	-3.643629	-0.813374
H	-0.825174	-3.187459	2.126967
H	-2.261803	-3.949731	1.433512
H	0.792723	-2.471055	-2.090287
H	-0.79167	-1.704527	-2.306244
H	0.118224	0.485746	-1.715473
H	1.130944	-0.219153	-2.95755
H	3.518236	-1.18792	1.142765
H	4.838196	-0.542636	-0.858603
H	6.254338	0.556485	0.894311
H	6.421107	1.964924	-0.153274
H	7.706727	0.620564	-1.812408
H	7.333242	-1.704738	0.12907
H	6.680418	-1.58071	-1.515557
H	8.411243	-1.710172	-1.277322
H	11.827221	2.142977	-0.670418
H	12.463794	1.775933	0.93723
H	10.653765	3.503124	0.964409
H	10.293881	2.10034	1.95855
H	8.30424	2.630687	0.760044
H	12.359095	-0.037524	-1.78183
H	13.331797	-0.250352	-0.313966
H	11.296486	-2.209822	-1.457994
H	13.045561	-2.413195	-1.373782
H	12.995876	-2.448536	1.063418
H	11.826742	-3.636105	0.469983
H	10.922772	-2.218195	2.335634
H	9.963257	-2.040996	0.869915
H	-4.971464	1.368977	1.925163
H	1.786508	-1.328579	2.708441
H	4.237088	0.992307	-2.611885
H	9.838903	2.480608	-1.893524
H	8.14171	2.915126	-1.661377
H	9.40554	3.979694	-1.05492
H	-5.966945	3.629203	-3.171087
H	-6.143387	4.716414	-1.786369

H	-4.566378	3.996344	-2.139862
H	3.463829	1.603776	3.186039
H	4.16829	2.80131	1.965512

Cartesian coordinates for conformer 2-38 after optimization at the PCM/B3LYP/6-31G* level of theory. Number of imaginary frequencies = 0. SCF Energy (PCM/mPW1PW91/6-31+G**) = -2695.10776485.

Atom	X	Y	Z
O	6.765259	-0.063397	2.87171
C	5.422405	-0.274841	2.92645
C	5.093109	-1.772279	2.840935
O	4.611523	0.630275	3.045085
C	3.919053	-2.046426	1.883919
C	4.129294	-1.542945	0.443171
C	4.774635	-2.271208	4.249307
O	6.28614	-2.46178	2.412887
C	2.971273	-2.007196	-0.44592
C	3.210404	-1.626364	-1.89882
C	4.576827	-2.130162	-2.36292
C	5.692644	-1.664878	-1.376038
O	5.378717	-2.062961	-0.031953
O	5.792094	-0.239137	-1.512445
C	6.951904	0.322402	-0.872398
C	8.20122	-0.151576	-1.634176
C	8.170331	-1.644422	-1.8529
C	7.014667	-2.319597	-1.707633
C	6.783281	1.864007	-0.871634
C	5.571563	2.235065	-0.037033
C	8.009978	2.600678	-0.332487
C	4.388144	2.602497	-0.556173
C	3.15003	2.829243	0.262645
C	2.149489	1.932376	-0.254628
C	1.029622	2.700835	-0.707864
C	1.573171	4.100734	-0.968086
C	2.598049	4.241633	0.133915
C	0.438336	2.077515	-1.97809
C	-0.231436	0.736026	-1.689052
C	-1.225422	0.887288	-0.541635
C	-0.513316	1.509274	0.657689
O	0.043519	2.792779	0.330445
O	-1.751562	-0.392626	-0.201183
C	-2.741372	-0.377201	0.843211
C	-2.222632	0.351982	2.074713
C	-1.531796	1.669697	1.792797
C	-4.15486	0.089252	0.364666
C	-4.550168	-0.607903	-0.951067
C	-6.01665	-0.435062	-1.42065
C	-7.081487	-0.87096	-0.379252
C	-6.292339	0.994429	-1.913831
O	-8.376248	-0.73501	-0.98948
C	-9.473091	-0.912503	-0.085012
C	-9.411283	-2.311036	0.552437
C	-8.040724	-2.595009	1.161392
C	-6.909444	-2.307815	0.16319
C	-10.780699	-0.796201	-0.890716
C	-11.009813	0.626386	-1.392533
C	-10.905843	1.609036	-0.235706
C	-9.601917	1.402013	0.518308
O	-9.466309	0.055708	0.972151
H	-2.028359	1.542447	-0.898696
H	-7.029886	-0.168213	0.461779
H	4.175566	-0.448842	0.449675
O	4.796561	-1.641073	-3.694309
H	6.996232	-0.033993	0.1651
H	3.323927	2.57832	1.314642

H	0.294928	0.857616	1.018999
O	-0.867804	2.164682	2.96138
H	-2.856033	-1.438513	1.102811
O	-4.216793	1.503823	0.174044
C	-6.887043	-3.372283	-0.942397
C	9.461248	-2.304271	-2.231495
C	-2.340516	-0.182882	3.304789
H	7.194712	-0.951409	2.796091
H	2.996504	-1.604938	2.281762
H	3.773236	-3.134718	1.845213
H	3.890114	-1.775953	4.663804
H	4.60325	-3.35342	4.25097
H	5.614305	-2.085625	4.929619
H	6.2361	-2.51824	1.430981
H	2.876137	-3.0991	-0.389339
H	2.025632	-1.577929	-0.096249
H	3.146663	-0.537212	-2.018868
H	2.421459	-2.041146	-2.537267
H	4.54564	-3.225883	-2.408212
H	9.099293	0.118728	-1.067643
H	8.257925	0.335045	-2.615591
H	6.99507	-3.397969	-1.842094
H	6.611424	2.196392	-1.904546
H	5.669213	2.122601	1.042541
H	7.819878	3.677466	-0.256174
H	8.289666	2.236948	0.662627
H	8.871296	2.471434	-0.995493
H	4.279392	2.677879	-1.635391
H	0.80771	4.881233	-0.921565
H	2.077631	4.156668	-1.940045
H	2.095181	4.525271	1.066932
H	3.356124	5.0027	-0.075393
H	-0.314594	2.754433	-2.401111
H	1.219228	1.927527	-2.732798
H	0.52127	-0.020593	-1.437073
H	-0.742649	0.362554	-2.58419
H	-2.268804	2.434735	1.528402
H	-4.874976	-0.160937	1.151685
H	-4.34151	-1.680046	-0.857019
H	-3.901021	-0.242871	-1.758162
H	-6.138057	-1.066022	-2.312004
H	-6.376054	1.702407	-1.084279
H	-5.498241	1.339135	-2.584525
H	-7.231833	1.037333	-2.475641
H	-9.620883	-3.076813	-0.204283
H	-10.172974	-2.408828	1.335669
H	-7.995544	-3.633457	1.5099
H	-7.903596	-1.963736	2.048756
H	-5.963025	-2.381985	0.711919
H	-10.765544	-1.477037	-1.750102
H	-11.631878	-1.074305	-0.255816
H	-10.265543	0.875891	-2.158405
H	-11.995395	0.703709	-1.864981
H	-11.747961	1.440995	0.44662
H	-10.976018	2.63855	-0.601741
H	-9.581357	2.048959	1.401272
H	-8.740543	1.675317	-0.100226
H	5.124712	-0.726609	-3.578812
H	-0.256731	2.852852	2.635239
H	-4.407898	1.899687	1.043993
H	-7.752966	-3.295842	-1.607426
H	-5.98763	-3.283096	-1.558789
H	-6.880484	-4.378639	-0.510092
H	10.206989	-2.160965	-1.443015
H	9.340806	-3.381389	-2.386789
H	9.848688	-1.873397	-3.160261

H	-1.950433	0.327742	4.181774
H	-2.820193	-1.142017	3.473605

Cartesian coordinates for conformer 2-39 after optimization at the PCM/B3LYP/6-31G* level of theory. Number of imaginary frequencies = 0. SCF Energy (PCM/mPW1PW91/6-31+G**) = -2695.10930916.

Atom	X	Y	Z
O	4.47744	1.704647	2.621595
C	3.304036	1.019368	2.669565
C	3.536435	-0.450096	3.052821
O	2.217196	1.539956	2.466958
C	2.77399	-1.395762	2.108043
C	3.263937	-1.398882	0.647114
C	3.068117	-0.633845	4.496371
O	4.956052	-0.69993	3.038863
C	2.400456	-2.362746	-0.174187
C	2.950117	-2.53711	-1.579474
C	4.41699	-2.956975	-1.518424
C	5.230995	-1.958567	-0.637841
O	4.637701	-1.811667	0.660288
O	5.275339	-0.718987	-1.362216
C	6.230638	0.225635	-0.844416
C	7.646989	-0.336928	-1.061023
C	7.747137	-1.768392	-0.600994
C	6.627076	-2.484148	-0.393652
C	6.001959	1.582819	-1.571147
C	4.571748	2.026313	-1.318852
C	7.05334	2.63127	-1.194638
C	4.134864	3.089041	-0.622046
C	2.697155	3.359317	-0.282858
C	1.851956	2.280885	-0.703195
C	0.520086	2.794182	-0.838954
C	0.629919	4.322995	-0.763697
C	2.107851	4.57931	-0.958582
C	-0.056005	2.330664	-2.186069
C	-0.311006	0.823781	-2.202897
C	-1.13522	0.424109	-0.979902
C	-0.415775	0.913812	0.276155
O	-0.291307	2.346599	0.251754
O	-1.294129	-0.991835	-0.966016
C	-2.077574	-1.486693	0.135569
C	-1.495196	-1.016936	1.458658
C	-1.186557	0.466655	1.52928
C	-3.614042	-1.247512	-0.017614
C	-4.110576	-1.713949	-1.399291
C	-5.602001	-2.131637	-1.477839
C	-6.711759	-1.059074	-1.283869
C	-5.837448	-2.926244	-2.771313
O	-6.468429	-0.327844	-0.073721
C	-7.592015	0.451599	0.372069
C	-7.993113	1.460798	-0.71644
C	-8.21382	0.780034	-2.064451
C	-7.013785	-0.098895	-2.45776
C	-7.166482	1.228168	1.632503
C	-6.921248	0.293316	2.81343
C	-8.126478	-0.612055	3.020772
C	-8.498231	-1.307414	1.720579
O	-8.728299	-0.366753	0.671642
H	-2.10823	0.914398	-1.086932
H	-7.628157	-1.647134	-1.127664
H	3.199187	-0.387432	0.231043
O	4.913607	-3.018497	-2.863665
H	6.045305	0.363621	0.230531
H	2.607232	3.453688	0.805564
H	0.58838	0.472293	0.338397

O	-0.422495	0.747713	2.712812
H	-1.941832	-2.575438	0.079613
O	-3.934063	0.133829	0.152546
C	-5.837242	0.781098	-2.88288
C	9.123456	-2.325393	-0.398513
C	-1.252231	-1.885026	2.459825
H	5.192624	1.063765	2.858032
H	1.705371	-1.155034	2.12908
H	2.887977	-2.416466	2.498876
H	1.994539	-0.443949	4.6015
H	3.280043	-1.649323	4.849458
H	3.599141	0.049185	5.170016
H	5.15161	-1.20154	2.215332
H	2.381729	-3.347327	0.310781
H	1.366613	-2.004204	-0.219785
H	2.842307	-1.604307	-2.147595
H	2.368086	-3.287741	-2.127092
H	4.471197	-3.967368	-1.094402
H	8.371091	0.274674	-0.511317
H	7.911454	-0.303237	-2.125134
H	6.696993	-3.509224	-0.038525
H	6.075582	1.413581	-2.654336
H	3.823665	1.345467	-1.728167
H	6.829074	3.599531	-1.656106
H	7.11008	2.772163	-0.109617
H	8.046851	2.337302	-1.548342
H	4.842805	3.785875	-0.181906
H	0.314118	4.687062	0.222209
H	0.017931	4.843781	-1.506935
H	2.441566	5.522399	-0.516545
H	2.342769	4.593301	-2.02994
H	-1.011691	2.83771	-2.369466
H	0.624363	2.586893	-3.006629
H	0.639006	0.273949	-2.213377
H	-0.834794	0.534866	-3.121737
H	-2.110577	1.045041	1.6283
H	-4.123369	-1.802334	0.779987
H	-3.523251	-2.598944	-1.679924
H	-3.886737	-0.951368	-2.148468
H	-5.74043	-2.844222	-0.650699
H	-5.607127	-2.328157	-3.658542
H	-5.201906	-3.818125	-2.798784
H	-6.876798	-3.263963	-2.841597
H	-7.206355	2.216117	-0.833463
H	-8.91326	1.985175	-0.431258
H	-8.413725	1.533753	-2.835048
H	-9.112959	0.152944	-2.00742
H	-7.318903	-0.682842	-3.334868
H	-6.254001	1.805327	1.441822
H	-7.957659	1.9344	1.915349
H	-6.728321	0.876984	3.72032
H	-6.02845	-0.315792	2.627286
H	-8.972727	-0.000262	3.356899
H	-7.919956	-1.349904	3.803045
H	-9.422867	-1.877033	1.85903
H	-7.723019	-2.020417	1.41781
H	5.151741	-2.097665	-3.092213
H	0.280493	1.386047	2.461704
H	-4.912443	0.171326	0.154276
H	-5.099081	0.198655	-3.44013
H	-6.169375	1.579398	-3.555906
H	-5.33842	1.249071	-2.029698
H	9.654595	-1.755954	0.370758
H	9.099006	-3.373483	-0.082387
H	9.69528	-2.270046	-1.330352
H	-0.83091	-1.550852	3.404256

H	-1.473944	-2.943623	2.371086

Cartesian coordinates for conformer 2-40 after optimization at the PCM/B3LYP/6-31G* level of theory. Number of imaginary frequencies = 0. SCF Energy (PCM/mPW1PW91/6-31+G**) = -2695.11411354.

Atom	X	Y	Z
O	6.627166	0.748431	2.525985
C	5.300017	0.68451	2.818008
C	4.901824	-0.723653	3.283725
O	4.554154	1.648185	2.746412
C	3.615828	-1.214851	2.593184
C	3.689891	-1.309989	1.056206
C	4.71968	-0.683584	4.800905
O	6.010379	-1.609112	3.022643
C	2.421957	-1.983204	0.517565
C	2.520151	-2.235135	-0.979005
C	3.802414	-3.000242	-1.307092
C	5.0366	-2.270946	-0.69262
O	4.855203	-2.07246	0.71746
O	5.180943	-1.035505	-1.411817
C	6.405553	-0.339115	-1.118403
C	7.583538	-1.168846	-1.662565
C	7.460242	-2.624988	-1.287967
C	6.289078	-3.104279	-0.829893
C	6.324592	1.077767	-1.755414
C	5.060754	1.77228	-1.275985
C	7.605162	1.886306	-1.524263
C	4.942741	2.857539	-0.491665
C	3.642324	3.418333	0.015923
C	2.550736	2.530011	-0.270916
C	1.402221	3.298969	-0.645783
C	1.778599	4.763233	-0.429884
C	3.276423	4.752142	-0.61204
C	1.064267	2.999271	-2.117317
C	0.588199	1.557163	-2.298279
C	-0.531572	1.247344	-1.308318
C	-0.066489	1.597459	0.105203
O	0.289128	2.985747	0.200454
O	-0.890454	-0.131816	-1.408422
C	-1.957792	-0.542289	-0.518533
C	-1.62766	-0.15694	0.910767
C	-1.203627	1.283759	1.084319
C	-3.325945	-0.024273	-1.052095
C	-4.519909	-0.577495	-0.257502
C	-5.914315	-0.201517	-0.816736
C	-7.061568	-0.819088	0.037537
C	-6.083394	1.325929	-0.89758
O	-8.31066	-0.423364	-0.554443
C	-9.465658	-0.750625	0.225714
C	-9.535822	-2.269965	0.447887
C	-8.228513	-2.81485	1.014628
C	-7.018303	-2.357928	0.186803
C	-10.713955	-0.30938	-0.561421
C	-10.812718	1.210484	-0.655951
C	-10.704899	1.826494	0.731085
C	-9.465347	1.311183	1.444936
O	-9.452508	-0.114174	1.51001
H	-1.388787	1.871332	-1.590576
H	-6.996318	-0.371698	1.03868
H	3.786776	-0.302463	0.637129
O	3.896865	-3.112026	-2.734409
H	6.499534	-0.230814	-0.030109
H	3.6936	3.518345	1.105944
H	0.800325	0.988772	0.397595
O	-0.766937	1.53959	2.421333

H	-1.967569	-1.636254	-0.60969
O	-3.464361	-0.418129	-2.425341
C	-6.980404	-3.090858	-1.160585
C	8.681783	-3.476403	-1.457473
C	-1.671348	-1.056238	1.911302
H	7.005103	-0.147665	2.705144
H	2.769592	-0.572103	2.867533
H	3.402231	-2.220398	2.981839
H	3.904577	-0.012946	5.093369
H	4.508845	-1.683751	5.195865
H	5.634861	-0.337594	5.295927
H	5.839893	-2.016826	2.142902
H	2.277939	-2.950754	1.015611
H	1.541005	-1.370981	0.737451
H	2.496771	-1.283292	-1.523652
H	1.647092	-2.798557	-1.329511
H	3.717835	-4.013433	-0.895147
H	8.524722	-0.767912	-1.270156
H	7.618422	-1.104611	-2.757227
H	6.205903	-4.147998	-0.537831
H	6.200184	0.960834	-2.84097
H	4.143748	1.281934	-1.605386
H	7.508148	2.902509	-1.922356
H	7.84906	1.956441	-0.458564
H	8.458189	1.427928	-2.034756
H	5.830191	3.367423	-0.12617
H	1.530912	5.077091	0.592305
H	1.267403	5.451734	-1.109529
H	3.774139	5.599225	-0.130829
H	3.523125	4.774818	-1.680597
H	0.262669	3.668946	-2.452883
H	1.934854	3.165758	-2.761799
H	1.422367	0.859923	-2.152639
H	0.239295	1.39569	-3.325063
H	-2.056327	1.949564	0.906896
H	-3.341233	1.068704	-1.036171
H	-4.454603	-0.246491	0.785802
H	-4.429054	-1.669419	-0.255106
H	-6.009096	-0.575248	-1.843853
H	-5.944331	1.789428	0.084802
H	-5.364374	1.771292	-1.591323
H	-7.077172	1.601815	-1.264837
H	-9.742884	-2.778568	-0.501544
H	-10.351272	-2.519321	1.137852
H	-8.271915	-3.909077	1.066178
H	-8.109101	-2.460897	2.046706
H	-6.120929	-2.646307	0.746849
H	-10.69829	-0.727205	-1.575072
H	-11.617398	-0.67869	-0.059081
H	-10.010361	1.59708	-1.295886
H	-11.761753	1.496723	-1.122341
H	-11.595228	1.550483	1.30948
H	-10.679927	2.919077	0.662791
H	-9.451306	1.686148	2.473426
H	-8.551513	1.670728	0.960067
H	4.280063	-2.263447	-3.034321
H	-0.258561	2.372345	2.372278
H	-2.572482	-0.351076	-2.82025
H	-7.790727	-2.77515	-1.824976
H	-6.034495	-2.910156	-1.680503
H	-7.064217	-4.173331	-1.01523
H	9.496261	-3.101962	-0.829326
H	8.496651	-4.519119	-1.179097
H	9.011711	-3.462124	-2.501069
H	-1.416574	-0.779232	2.931177
H	-1.961035	-2.089154	1.744236

Cartesian coordinates for conformer 2-41 after optimization at the PCM/B3LYP/6-31G* level of theory. Number of imaginary frequencies = 0. SCF Energy (PCM/mPW1PW91/6-31+G**) = -2695.11321305.

Atom	X	Y	Z
O	4.711598	0.711262	2.976997
C	3.518232	0.101565	2.737653
C	3.698676	-1.418041	2.593037
O	2.46136	0.710845	2.67169
C	2.91131	-1.967806	1.392065
C	3.32417	-1.38123	0.029771
C	3.24519	-2.076502	3.89447
O	5.111775	-1.683755	2.462249
C	2.474689	-2.008083	-1.079977
C	2.935991	-1.530989	-2.44645
C	4.428457	-1.80243	-2.617832
C	5.239059	-1.213197	-1.419548
O	4.71684	-1.670757	-0.163372
O	5.172589	0.21646	-1.539265
C	6.1389	0.915712	-0.732487
C	7.544263	0.604498	-1.274851
C	7.742634	-0.880222	-1.438262
C	6.672323	-1.694538	-1.476015
C	5.784666	2.425451	-0.789838
C	4.476108	2.654687	-0.06117
C	6.857317	3.325521	-0.173736
C	3.296798	2.871116	-0.666402
C	2.005327	2.957348	0.079322
C	1.143696	1.916901	-0.398448
C	-0.204334	2.305059	-0.090053
C	-0.160825	3.79553	0.276921
C	1.219128	4.227879	-0.164608
C	-1.091185	2.048497	-1.317889
C	-1.273531	0.554549	-1.581809
C	-1.727319	-0.144404	-0.301654
C	-0.726188	0.165534	0.809483
O	-0.676475	1.581585	1.052003
O	-1.822416	-1.545112	-0.540469
C	-2.264665	-2.313106	0.594394
C	-1.374142	-2.045716	1.796332
C	-1.122802	-0.579896	2.093446
C	-3.792906	-2.188624	0.883931
C	-4.627232	-2.506886	-0.376018
C	-6.154292	-2.397435	-0.135853
C	-6.809128	-1.099142	-0.691957
C	-6.880468	-3.663965	-0.59885
O	-5.964427	0.022072	-0.363102
C	-6.592461	1.298287	-0.576539
C	-7.040221	1.436621	-2.042975
C	-7.884107	0.250438	-2.498352
C	-7.187746	-1.083797	-2.191438
C	-5.562586	2.408111	-0.283754
C	-5.235865	2.530784	1.201186
C	-6.514022	2.601742	2.022414
C	-7.44619	1.45926	1.653779
O	-7.743653	1.468487	0.257748
H	-2.71255	0.265653	-0.059441
H	-7.745269	-0.989061	-0.125704
H	3.182288	-0.295095	0.038753
O	4.836656	-1.237719	-3.87299
H	6.057262	0.564387	0.306064
H	2.154624	2.807233	1.155393
H	0.27867	-0.177406	0.530918
O	-0.082723	-0.453774	3.075351
H	-2.105251	-3.353912	0.281324

O	-4.130401	-0.902181	1.407984
C	-6.011732	-1.295283	-3.149754
C	9.152536	-1.373718	-1.553339
C	-0.838832	-3.059573	2.503781
H	5.396865	-0.00222	3.002688
H	1.83993	-1.800789	1.543186
H	3.073742	-3.053918	1.355488
H	2.183845	-1.896777	4.092403
H	3.417435	-3.158192	3.865534
H	3.816171	-1.691045	4.747533
H	5.292726	-1.782109	1.499532
H	2.566789	-3.101266	-1.045401
H	1.415992	-1.76563	-0.939119
H	2.726003	-0.460254	-2.56543
H	2.370167	-2.035264	-3.238827
H	4.576398	-2.888077	-2.675551
H	8.298306	1.000345	-0.585444
H	7.69191	1.078732	-2.252743
H	6.812171	-2.76851	-1.569886
H	5.65751	2.716018	-1.841658
H	4.505778	2.572734	1.025239
H	6.522343	4.368525	-0.136703
H	7.102435	3.014496	0.848007
H	7.777925	3.304923	-0.765702
H	3.242741	2.914812	-1.751077
H	-0.268682	3.933985	1.359993
H	-0.948654	4.387525	-0.199999
H	1.600437	5.085977	0.396151
H	1.199319	4.488819	-1.229937
H	-2.08324	2.486041	-1.149873
H	-0.662388	2.519675	-2.209954
H	-0.336579	0.111377	-1.942678
H	-2.008426	0.394464	-2.37969
H	-2.01161	-0.120286	2.537461
H	-4.051101	-2.911501	1.667496
H	-4.367551	-3.51837	-0.711988
H	-4.324431	-1.825497	-1.175323
H	-6.302183	-2.357581	0.953229
H	-6.722141	-3.862826	-1.662601
H	-6.517705	-4.535865	-0.043196
H	-7.957609	-3.583377	-0.419132
H	-6.162036	1.510678	-2.695874
H	-7.624828	2.354914	-2.177738
H	-8.10343	0.337408	-3.568963
H	-8.852105	0.277512	-1.981658
H	-7.916156	-1.880455	-2.386793
H	-4.63437	2.225224	-0.837885
H	-5.964385	3.374686	-0.615259
H	-4.626684	3.424744	1.376487
H	-4.636163	1.674831	1.525526
H	-7.013481	3.555945	1.813975
H	-6.281671	2.575838	3.092165
H	-8.393486	1.569139	2.191501
H	-7.022342	0.48996	1.940181
H	4.992825	-0.289985	-3.687294
H	0.41819	0.363112	2.865246
H	-4.739345	-0.468907	0.774212
H	-5.608981	-2.308446	-3.064582
H	-5.196111	-0.589065	-2.966694
H	-6.334098	-1.170068	-4.189446
H	9.71771	-1.124415	-0.649573
H	9.197025	-2.459472	-1.687373
H	9.647163	-0.909916	-2.412757
H	-0.200389	-2.87157	3.362449
H	-1.030582	-4.098749	2.25635

Cartesian coordinates for conformer 2-42 after optimization at the PCM/B3LYP/6-31G* level of theory. Number of imaginary frequencies = 0. SCF Energy (PCM/mPW1PW91/6-31+G**) = -2695.10816028.

Atom	X	Y	Z
O	-9.188022	-0.563841	-0.58345
C	-8.757481	-1.451742	-1.519328
C	-8.646982	-2.866129	-0.932957
O	-8.504791	-1.137196	-2.671845
C	-7.354176	-3.568537	-1.386438
C	-6.057678	-2.802843	-1.05906
C	-9.878752	-3.661014	-1.361379
O	-8.700057	-2.752086	0.504048
C	-4.839433	-3.663921	-1.407994
C	-3.549187	-2.976393	-0.989546
C	-3.610533	-2.584001	0.485833
C	-4.91003	-1.771898	0.784398
O	-6.07466	-2.481124	0.336024
O	-4.762111	-0.504409	0.119116
C	-5.680254	0.501521	0.572109
C	-5.408861	0.833228	2.051686
C	-5.313615	-0.419492	2.879784
C	-5.095545	-1.60044	2.275006
C	-5.569916	1.757809	-0.334359
C	-4.184686	2.370939	-0.313524
C	-5.935757	1.424483	-1.783897
C	-3.915714	3.63323	0.063601
C	-2.550574	4.277735	0.111707
C	-1.536803	3.316521	-0.224044
C	-0.685037	3.115989	0.910503
C	-1.458302	3.647306	2.111605
C	-2.209615	4.80636	1.498787
C	-0.350042	1.627584	1.064599
C	0.548653	1.131786	-0.066949
C	1.769918	2.039462	-0.193679
C	1.300389	3.484502	-0.351673
O	0.508385	3.901597	0.77394
O	2.562108	1.613404	-1.299812
C	3.726544	2.414897	-1.576823
C	3.390148	3.897952	-1.618522
C	2.535949	4.382635	-0.472479
C	4.952283	2.063153	-0.675818
C	5.35273	0.581873	-0.837999
C	6.566911	0.126343	0.008234
C	6.903674	-1.376373	-0.231259
C	7.804274	0.993353	-0.283537
O	8.021421	-1.711117	0.608037
C	8.580158	-3.006372	0.359824
C	7.51206	-4.089398	0.58303
C	6.241257	-3.796317	-0.208947
C	5.742251	-2.363461	0.030243
C	9.727651	-3.231742	1.362164
C	10.91388	-2.31515	1.076867
C	11.330937	-2.442223	-0.380933
C	10.1332	-2.236893	-1.294651
O	9.072849	-3.1387	-0.979982
H	2.338366	1.927501	0.734902
H	7.214473	-1.475879	-1.280165
H	-6.030758	-1.872668	-1.638461
O	-2.427763	-1.828304	0.788322
H	-6.699763	0.105466	0.473183
H	-2.523461	5.084902	-0.628178
H	0.70176	3.607686	-1.265418
O	2.111557	5.736586	-0.673682
H	4.012293	2.11626	-2.594813

O	4.712482	2.353546	0.70484
C	5.14635	-2.229637	1.437966
C	-5.458311	-0.271878	4.363757
C	3.798612	4.682033	-2.634516
H	-9.328126	-1.070142	0.254966
H	-7.387348	-3.761447	-2.466138
H	-7.312422	-4.542194	-0.8791
H	-9.931083	-3.770667	-2.449818
H	-9.87695	-4.658761	-0.908307
H	-10.79898	-3.166809	-1.02816
H	-7.76837	-2.662159	0.809193
H	-4.902431	-4.625371	-0.882285
H	-4.81722	-3.878316	-2.482284
H	-3.375485	-2.089	-1.611709
H	-2.690329	-3.635063	-1.165751
H	-3.588663	-3.500245	1.088865
H	-6.21219	1.47869	2.425736
H	-4.462233	1.372237	2.172441
H	-5.041568	-2.509223	2.8695
H	-6.298606	2.495189	0.028331
H	-3.371197	1.721187	-0.633547
H	-5.895122	2.320292	-2.414048
H	-5.258619	0.68018	-2.21755
H	-6.954242	1.029684	-1.840642
H	-4.740188	4.281101	0.356845
H	-0.816006	3.962618	2.939174
H	-2.175813	2.906087	2.483201
H	-1.539266	5.670284	1.408699
H	-3.07585	5.119022	2.089678
H	0.173164	1.466983	2.015782
H	-1.26622	1.026427	1.079568
H	-0.004908	1.109657	-1.01436
H	0.863872	0.098526	0.120405
H	3.109057	4.380672	0.460915
H	5.783014	2.703938	-0.9904
H	5.555346	0.378566	-1.897197
H	4.486999	-0.027268	-0.556294
H	6.342238	0.263505	1.073184
H	8.062676	0.964694	-1.347328
H	7.637358	2.0362	0.001463
H	8.675977	0.655174	0.285794
H	7.253717	-4.147253	1.6474
H	7.895211	-5.072931	0.28499
H	5.46433	-4.525248	0.049734
H	6.446359	-3.928859	-1.279037
H	4.936522	-2.180514	-0.690468
H	9.383247	-3.056664	2.388345
H	10.076564	-4.27045	1.296657
H	10.642341	-1.275324	1.29495
H	11.753199	-2.572398	1.73222
H	11.741007	-3.44645	-0.54391
H	12.119482	-1.720035	-0.61687
H	10.426242	-2.425357	-2.332531
H	9.771229	-1.20448	-1.24623
H	-2.627199	-0.916887	0.495015
H	1.399706	5.874895	-0.020596
H	4.4044	1.537359	1.135262
H	5.911029	-2.296718	2.218016
H	4.632074	-1.271248	1.558389
H	4.406126	-3.015834	1.62139
H	-6.442948	0.137633	4.610617
H	-5.354274	-1.23029	4.883033
H	-4.691385	0.405053	4.753572
H	3.542311	5.73769	-2.669883
H	4.396662	4.297852	-3.454649

Cartesian coordinates for conformer 2-43 after optimization at the PCM/B3LYP/6-31G* level of theory. Number of imaginary frequencies = 0. SCF Energy (PCM/mPW1PW91/6-31+G**) = -2695.10984038.

Atom	X	Y	Z
O	4.49694	-1.436089	1.949593
C	5.75445	-1.955765	1.939101
C	6.653835	-1.23781	2.957178
O	6.087007	-2.879821	1.213759
C	8.079294	-1.010396	2.424023
C	8.152584	-0.269989	1.076252
C	6.679831	-2.06654	4.240298
O	6.020504	0.017825	3.282259
C	9.606015	0.083823	0.744142
C	9.688741	0.898807	-0.538368
C	8.760006	2.111047	-0.464463
C	7.319614	1.673088	-0.052689
O	7.352918	0.915815	1.168267
O	6.788533	0.900347	-1.145587
C	5.371762	0.679859	-1.044279
C	4.639806	2.022324	-1.215508
C	5.231264	3.070618	-0.305875
C	6.451464	2.879435	0.229174
C	4.97763	-0.412606	-2.06835
C	3.54631	-0.863908	-1.889517
C	5.222124	-0.003344	-3.524918
C	3.20541	-2.055872	-1.36924
C	1.80758	-2.578447	-1.150248
C	0.840767	-1.578693	-1.513798
C	0.080094	-1.222555	-0.352627
C	0.911359	-1.674875	0.841808
C	1.54317	-2.938398	0.30595
C	-0.172644	0.289889	-0.328817
C	-1.129131	0.719884	-1.439995
C	-2.398505	-0.126397	-1.385183
C	-2.009997	-1.603502	-1.42999
O	-1.153959	-1.955326	-0.329521
O	-3.242683	0.215022	-2.482836
C	-4.482767	-0.518425	-2.535257
C	-4.229479	-2.018428	-2.481154
C	-3.286193	-2.452842	-1.382365
C	-5.555159	-0.007414	-1.520246
C	-5.732299	1.519477	-1.626493
C	-7.117578	2.07124	-1.200416
C	-7.553036	1.985535	0.290426
C	-7.276628	3.504568	-1.729452
O	-7.387727	0.641306	0.764106
C	-8.060144	0.374974	2.00734
C	-7.539512	1.326411	3.0974
C	-7.616346	2.785789	2.6574
C	-6.94829	3.000189	1.287973
C	-7.741782	-1.073585	2.423457
C	-8.381957	-2.084765	1.47653
C	-9.86851	-1.794109	1.330758
C	-10.090787	-0.33712	0.956591
O	-9.478231	0.544011	1.898244
H	-2.893874	0.115534	-0.438968
H	-8.633693	2.189442	0.265905
H	7.743287	-0.918512	0.29268
O	8.782185	2.761293	-1.743267
H	5.159067	0.282813	-0.04292
H	1.66153	-3.451664	-1.795617
H	-1.486221	-1.841581	-2.366941
O	-2.930033	-3.836005	-1.520016
H	-4.881635	-0.284217	-3.531627

O	-5.188803	-0.342262	-0.180208
C	-5.427051	2.927103	1.429979
C	4.408108	4.296468	-0.051693
C	-4.77708	-2.859014	-3.379119
H	4.494301	-0.695419	2.605273
H	8.604929	-1.968712	2.327039
H	8.619565	-0.413688	3.171664
H	7.120458	-3.055248	4.073659
H	7.250629	-1.555864	5.023992
H	5.666945	-2.211405	4.634214
H	6.351308	0.669964	2.622627
H	10.036549	0.680835	1.55822
H	10.209918	-0.825441	0.648753
H	9.424884	0.27208	-1.399947
H	10.719499	1.227837	-0.716105
H	9.166197	2.8121	0.27539
H	3.57504	1.891024	-0.992812
H	4.729496	2.394338	-2.241997
H	6.871314	3.624182	0.900639
H	5.646139	-1.266464	-1.885095
H	2.765131	-0.175975	-2.207857
H	5.059894	-0.855481	-4.19514
H	4.546728	0.796052	-3.847146
H	6.251432	0.339209	-3.672465
H	3.998419	-2.737329	-1.059847
H	0.317502	-1.859749	1.741925
H	1.696577	-0.947094	1.078155
H	0.817881	-3.760297	0.356002
H	2.431186	-3.248346	0.864695
H	-0.615383	0.571595	0.634869
H	0.769149	0.839954	-0.439856
H	-0.648306	0.616673	-2.421036
H	-1.379783	1.782441	-1.338804
H	-3.768623	-2.367419	-0.403532
H	-6.500283	-0.519193	-1.739863
H	-5.597173	1.795283	-2.681185
H	-4.933837	2.022491	-1.076443
H	-7.847763	1.458968	-1.750919
H	-6.508432	4.170692	-1.324109
H	-7.191044	3.525838	-2.821497
H	-8.259669	3.912301	-1.471327
H	-6.494686	1.089004	3.332274
H	-8.120139	1.20512	4.019849
H	-7.159975	3.429518	3.41846
H	-8.670093	3.086351	2.592413
H	-7.198158	4.0187	0.966429
H	-6.658511	-1.241732	2.441987
H	-8.127972	-1.260757	3.43368
H	-8.232669	-3.101537	1.856303
H	-7.897021	-2.034142	0.494174
H	-10.3629	-1.999398	2.288163
H	-10.314869	-2.453725	0.579295
H	-11.16337	-0.117734	0.951888
H	-9.714315	-0.125484	-0.050621
H	8.14256	2.269643	-2.296118
H	-2.170621	-3.95632	-0.919018
H	-5.958702	-0.081917	0.366148
H	-4.935087	3.364388	0.557521
H	-5.087072	3.505778	2.296067
H	-5.069855	1.900861	1.552378
H	3.461662	4.025827	0.427224
H	4.923138	5.007847	0.602302
H	4.188093	4.807337	-0.994547
H	-4.577083	-3.926616	-3.34528
H	-5.434382	-2.509476	-4.168699

Cartesian coordinates for conformer 2-44 after optimization at the PCM/B3LYP/6-31G* level of theory. Number of imaginary frequencies = 0. SCF Energy (PCM/mPW1PW91/6-31+G**) = -2695.10988487.

Atom	X	Y	Z
O	4.193941	-3.198318	1.329283
C	3.716872	-2.795385	0.12191
C	4.467918	-3.473496	-1.030622
O	2.798102	-2.001133	-0.001106
C	4.778432	-2.485958	-2.17064
C	5.557502	-1.22592	-1.744882
C	3.619443	-4.638716	-1.5362
O	5.679873	-4.039842	-0.492041
C	6.025282	-0.456333	-2.985571
C	6.922875	0.71233	-2.60754
C	8.064106	0.239738	-1.706719
C	7.497083	-0.561028	-0.4938
O	6.669689	-1.641984	-0.947761
O	6.782216	0.384775	0.319467
C	6.40036	-0.119501	1.61043
C	7.672059	-0.42083	2.430882
C	8.714365	-1.153361	1.628869
C	8.607923	-1.214199	0.290372
C	5.520044	0.956211	2.303925
C	4.260313	1.265037	1.514275
C	5.114515	0.558307	3.727115
C	4.080224	2.405409	0.824048
C	2.840792	2.826696	0.08091
C	1.680098	2.189829	0.637044
C	0.954518	1.561522	-0.424739
C	2.002814	1.252932	-1.485289
C	2.891455	2.474824	-1.401199
C	0.249353	0.301736	0.088705
C	-0.904167	0.639645	1.032423
C	-1.834345	1.652584	0.372677
C	-1.011836	2.861512	-0.068132
O	0.016013	2.485077	-1.000011
O	-2.851574	2.034355	1.297014
C	-3.801568	2.987727	0.783563
C	-3.09086	4.206785	0.214035
C	-1.950095	3.882633	-0.72374
C	-4.869101	2.363815	-0.173491
C	-5.5385	1.13719	0.475633
C	-6.649044	0.457412	-0.361009
C	-7.254562	-0.773926	0.377751
C	-7.764265	1.456286	-0.716586
O	-8.257412	-1.343414	-0.480879
C	-9.046816	-2.369475	0.131685
C	-8.141597	-3.523576	0.592035
C	-6.991067	-3.022522	1.459608
C	-6.238034	-1.864789	0.787836
C	-10.032679	-2.906982	-0.922679
C	-11.089632	-1.868572	-1.287685
C	-11.758076	-1.341013	-0.026802
C	-10.711796	-0.862841	0.967314
O	-9.770047	-1.890406	1.272704
H	-2.280961	1.151182	-0.492535
H	-7.746841	-0.39853	1.285188
H	4.906104	-0.587883	-1.137306
O	8.804737	1.396311	-1.29144
H	5.815035	-1.036523	1.473397
H	2.715453	3.907447	0.213958
H	-0.536718	3.34877	0.795304
O	-1.199843	5.059279	-1.056032
H	-4.356171	3.308715	1.675799

O	-4.297711	1.973441	-1.426155
C	-5.400916	-2.379546	-0.39057
C	9.843965	-1.778896	2.389468
C	-3.4417	5.457279	0.568956
H	4.926317	-3.840015	1.155593
H	3.851313	-2.173476	-2.667724
H	5.381833	-3.025536	-2.913543
H	2.660707	-4.2957	-1.939744
H	4.14823	-5.197696	-2.316394
H	3.409522	-5.349764	-0.728464
H	6.359553	-3.329057	-0.541578
H	6.597772	-1.125524	-3.640677
H	5.163501	-0.096702	-3.558961
H	6.337044	1.489403	-2.100561
H	7.329596	1.188221	-3.507773
H	8.734634	-0.394433	-2.299701
H	7.401773	-1.016373	3.310365
H	8.123293	0.514824	2.784755
H	9.349329	-1.759231	-0.288269
H	6.110699	1.880892	2.373009
H	3.465276	0.520266	1.540315
H	4.42822	1.293987	4.162349
H	4.615202	-0.41714	3.740752
H	5.984967	0.503822	4.388382
H	4.893636	3.128042	0.789239
H	1.584368	1.116993	-2.487027
H	2.586165	0.365237	-1.220588
H	2.452612	3.291506	-1.987965
H	3.898892	2.298896	-1.790574
H	-0.152313	-0.265663	-0.760031
H	0.954234	-0.341123	0.624771
H	-0.519314	1.041235	1.978531
H	-1.460432	-0.267686	1.296299
H	-2.332689	3.497217	-1.674352
H	-5.610552	3.142503	-0.38433
H	-5.950212	1.426238	1.450831
H	-4.753776	0.397054	0.664452
H	-6.229994	0.111604	-1.314016
H	-8.201848	1.89448	0.186725
H	-7.388556	2.269238	-1.344387
H	-8.568316	0.976424	-1.283686
H	-7.721527	-4.040803	-0.279164
H	-8.719732	-4.260214	1.162889
H	-6.30775	-3.849359	1.686569
H	-7.390442	-2.677861	2.422008
H	-5.545678	-1.45796	1.534312
H	-9.498901	-3.20454	-1.833213
H	-10.547635	-3.792986	-0.52928
H	-10.624529	-1.03969	-1.834647
H	-11.838122	-2.312849	-1.953058
H	-12.343464	-2.150237	0.426946
H	-12.450255	-0.528957	-0.272726
H	-11.199451	-0.576345	1.904703
H	-10.187974	0.023657	0.594227
H	8.313548	1.755181	-0.52527
H	-0.386305	4.722915	-1.477216
H	-5.031633	1.835188	-2.050612
H	-6.024818	-2.70812	-1.227527
H	-4.726551	-1.602551	-0.763693
H	-4.776195	-3.224636	-0.081978
H	9.461844	-2.5311	3.086993
H	10.561866	-2.271884	1.725686
H	10.38454	-1.017051	2.960011
H	-2.917951	6.322986	0.172254
H	-4.253483	5.652835	1.262082

Cartesian coordinates for conformer 2-45 after optimization at the PCM/B3LYP/6-31G* level of theory. Number of imaginary frequencies = 0. SCF Energy (PCM/mPW1PW91/6-31+G**) = -2695.10948446.

Atom	X	Y	Z
O	6.722948	-2.581785	0.088369
C	5.613484	-2.670137	-0.693918
C	5.894871	-2.161514	-2.115252
O	4.553864	-3.129703	-0.298806
C	4.777663	-1.23149	-2.624592
C	4.536878	0.03148	-1.773484
C	6.046315	-3.376587	-3.030114
O	7.171717	-1.490043	-2.102033
C	3.538515	0.954576	-2.482774
C	3.394949	2.283385	-1.755503
C	4.766701	2.920373	-1.529683
C	5.722471	1.903292	-0.835038
O	5.798063	0.686328	-1.592017
O	5.207463	1.701394	0.491225
C	6.088046	0.942482	1.33767
C	7.353785	1.779144	1.605937
C	7.894967	2.398268	0.341032
C	7.136113	2.430413	-0.770073
C	5.323051	0.583555	2.642082
C	3.999365	-0.080606	2.297603
C	6.191246	-0.22578	3.611342
C	3.637859	-1.362538	2.478066
C	2.322429	-1.981095	2.064837
C	1.511603	-1.015372	1.372786
C	0.297379	-0.816078	2.104706
C	0.585655	-1.30047	3.520413
C	1.511346	-2.466402	3.25901
C	-0.096867	0.665733	2.077626
C	-0.516105	1.110754	0.677275
C	-1.590377	0.169939	0.137257
C	-1.073017	-1.266052	0.210464
O	-0.745244	-1.638838	1.5603
O	-1.905022	0.532824	-1.205848
C	-2.920153	-0.283543	-1.823546
C	-2.564169	-1.7599	-1.722894
C	-2.150282	-2.209815	-0.339773
C	-4.371327	0.064721	-1.360714
C	-4.637437	1.57837	-1.470783
C	-6.118466	1.992783	-1.670274
C	-7.143531	1.755467	-0.524493
C	-6.175932	3.449376	-2.154985
O	-7.065719	0.392458	-0.083454
C	-8.178038	-0.021244	0.728959
C	-8.27577	0.87285	1.976059
C	-8.30053	2.35405	1.608603
C	-7.132417	2.726336	0.678811
C	-7.927517	-1.472587	1.180603
C	-7.99264	-2.446303	0.00731
C	-9.291999	-2.251476	-0.760137
C	-9.475271	-0.788426	-1.131786
O	-9.419569	0.054573	0.019241
H	-2.468443	0.298837	0.778669
H	-8.122165	1.883194	-1.010026
H	4.142524	-0.263769	-0.794775
O	4.577946	4.10695	-0.745244
H	6.360374	0.012517	0.822608
H	2.525891	-2.809254	1.378931
H	-0.174538	-1.389	-0.41142
O	-1.630056	-3.546873	-0.363823
H	-2.871203	-0.000847	-2.884022

O	-4.588897	-0.350016	-0.010852
C	-5.822172	2.750827	1.467693
C	9.282457	2.961501	0.399698
C	-2.580816	-2.564325	-2.802687
H	7.445698	-2.212372	-0.476686
H	3.834301	-1.785366	-2.710371
H	5.060164	-0.907148	-3.635817
H	5.122378	-3.96238	-3.081603
H	6.32339	-3.069723	-4.044926
H	6.844984	-4.037937	-2.673664
H	6.982528	-0.536016	-1.94881
H	3.888522	1.16212	-3.502267
H	2.561004	0.466294	-2.56674
H	2.882605	2.13397	-0.796272
H	2.755928	2.965175	-2.329199
H	5.175498	3.221009	-2.502376
H	8.121589	1.143804	2.061326
H	7.128139	2.591897	2.307468
H	7.533931	2.855341	-1.687966
H	5.052567	1.519578	3.150943
H	3.283529	0.591676	1.823696
H	5.618097	-0.532347	4.493552
H	6.595067	-1.125479	3.134276
H	7.035756	0.369041	3.973775
H	4.34208	-2.06575	2.916745
H	-0.311676	-1.60095	4.06976
H	1.118504	-0.538016	4.10116
H	0.921326	-3.345101	2.970234
H	2.111852	-2.744003	4.130814
H	-0.940043	0.832394	2.759788
H	0.736811	1.293357	2.41304
H	0.349839	1.122557	0.00294
H	-0.893125	2.140044	0.699845
H	-3.014639	-2.240121	0.331299
H	-5.071832	-0.494786	-1.992895
H	-4.089069	1.947435	-2.348283
H	-4.208967	2.091862	-0.607152
H	-6.47457	1.376442	-2.509568
H	-5.728657	4.133658	-1.427075
H	-5.630123	3.565402	-3.09772
H	-7.209548	3.763171	-2.335632
H	-7.41636	0.692379	2.633351
H	-9.181966	0.635669	2.546415
H	-8.283424	2.964747	2.519015
H	-9.247303	2.582998	1.102709
H	-7.319281	3.747158	0.323573
H	-6.947185	-1.564013	1.663204
H	-8.690758	-1.768355	1.911842
H	-7.920943	-3.477234	0.370849
H	-7.139327	-2.281337	-0.661565
H	-10.127462	-2.568919	-0.124197
H	-9.30287	-2.877858	-1.658219
H	-10.456943	-0.646456	-1.594963
H	-8.724619	-0.469145	-1.863618
H	4.53	3.797952	0.181525
H	-1.182949	-3.654541	0.497036
H	-5.541109	-0.191092	0.153656
H	-5.048493	3.292028	0.917082
H	-5.948461	3.279779	2.418914
H	-5.451994	1.746169	1.690382
H	10.002049	2.174524	0.647129
H	9.585855	3.404439	-0.55465
H	9.342546	3.741886	1.165045
H	-2.308164	-3.613605	-2.726489
H	-2.864168	-2.202597	-3.785696

Cartesian coordinates for conformer 2-46 after optimization at the PCM/B3LYP/6-31G* level of theory. Number of imaginary frequencies = 0. SCF Energy (PCM/mPW1PW91/6-31+G**) = -2695.11166012.

Atom	X	Y	Z
O	-5.264969	0.581632	3.248656
C	-4.147388	1.053994	2.635142
C	-4.286394	2.544167	2.295488
O	-3.169091	0.361057	2.406245
C	-3.733172	2.873099	0.896214
C	-4.34047	2.048728	-0.256097
C	-3.558565	3.349696	3.370174
O	-5.684266	2.886842	2.390592
C	-3.913172	2.637468	-1.605614
C	-4.611014	1.937239	-2.76258
C	-6.124365	1.919788	-2.545791
C	-6.459627	1.341489	-1.136816
O	-5.763219	2.071994	-0.116197
O	-6.100054	-0.050847	-1.170021
C	-6.54336	-0.789615	-0.020197
C	-8.086262	-0.820446	-0.00351
C	-8.690741	0.525749	-0.304673
C	-7.924198	1.505792	-0.813927
C	-5.948959	-2.222276	-0.09548
C	-4.435258	-2.210814	-0.220521
C	-6.332792	-3.070003	1.121632
C	-3.776075	-2.787538	-1.241647
C	-2.28422	-2.87347	-1.434335
C	-1.59108	-2.238938	-0.349301
C	-0.699521	-1.257184	-0.883458
C	-1.295272	-0.869584	-2.229541
C	-1.822974	-2.197492	-2.721083
C	-0.593129	-0.053148	0.058144
C	0.123448	-0.415211	1.358342
C	1.458772	-1.080841	1.040542
C	1.215447	-2.265447	0.105108
O	0.593982	-1.837399	-1.118342
O	2.094206	-1.493527	2.251308
C	3.382033	-2.133701	2.070395
C	3.264913	-3.278105	1.081654
C	2.568191	-2.915994	-0.209188
C	4.460228	-1.060063	1.732711
C	5.888217	-1.632083	1.750471
C	7.037681	-0.6599	1.381982
C	6.855849	0.057783	0.018295
C	7.310464	0.355502	2.503379
O	8.019196	0.870131	-0.214046
C	7.906668	1.746124	-1.342271
C	7.658627	0.929077	-2.621592
C	6.488365	-0.037223	-2.456493
C	6.633169	-0.886511	-1.185348
C	9.238172	2.505646	-1.492063
C	9.458323	3.488475	-0.345652
C	8.243191	4.391369	-0.193344
C	6.975405	3.559131	-0.085103
O	6.828858	2.680795	-1.200556
H	2.080768	-0.326566	0.541848
H	5.989875	0.725677	0.108144
H	-3.992642	1.012465	-0.177534
O	-6.711095	1.144993	-3.601024
H	-6.170094	-0.29299	0.882837
H	-2.003804	-3.932963	-1.441896
H	0.576806	-3.023045	0.580716
O	2.366974	-4.06445	-1.03625
H	3.620425	-2.530466	3.065841

O	4.356717	0.000941	2.690186
C	7.755063	-1.92001	-1.356983
C	-10.152176	0.694791	-0.019303
C	3.707972	-4.514065	1.377994
H	-5.901644	1.335105	3.319669
H	-2.642911	2.750237	0.878755
H	-3.9446	3.934092	0.703983
H	-2.486383	3.12686	3.385412
H	-3.690772	4.425521	3.209241
H	-3.962211	3.129508	4.365614
H	-6.071987	2.704734	1.504056
H	-4.178449	3.701831	-1.644164
H	-2.826113	2.567366	-1.725559
H	-4.233244	0.91244	-2.867111
H	-4.374622	2.437281	-3.709358
H	-6.499334	2.947102	-2.63327
H	-8.4312	-1.165899	0.97754
H	-8.457495	-1.526617	-0.756886
H	-8.35493	2.483946	-1.012055
H	-6.36052	-2.710175	-0.990116
H	-3.878111	-1.726573	0.581203
H	-5.850886	-4.053797	1.084877
H	-6.032544	-2.58361	2.056656
H	-7.412582	-3.243856	1.16089
H	-4.356433	-3.280264	-2.020457
H	-0.567917	-0.433315	-2.920525
H	-2.131675	-0.172925	-2.104417
H	-1.004154	-2.776478	-3.166478
H	-2.601304	-2.093426	-3.483372
H	-0.028033	0.747894	-0.435097
H	-1.587669	0.337224	0.294311
H	-0.49761	-1.086724	1.96468
H	0.283284	0.480859	1.969574
H	3.194782	-2.229242	-0.790245
H	4.247726	-0.616418	0.756551
H	5.939626	-2.488387	1.069295
H	6.094268	-2.031076	2.752663
H	7.95432	-1.263211	1.327854
H	6.550635	1.140966	2.538457
H	7.345215	-0.137004	3.480751
H	8.278493	0.846493	2.354428
H	8.554699	0.350051	-2.875629
H	7.449771	1.595619	-3.467341
H	6.405659	-0.677165	-3.342781
H	5.553798	0.535324	-2.399212
H	5.695399	-1.438077	-1.051949
H	10.08035	1.804377	-1.524505
H	9.236071	3.073254	-2.4315
H	9.630752	2.939394	0.587905
H	10.353821	4.090778	-0.534472
H	8.174103	5.041231	-1.074432
H	8.353599	5.035208	0.685465
H	6.102258	4.219444	-0.071321
H	6.958411	2.984855	0.846951
H	-6.632932	0.213813	-3.311092
H	1.708419	-3.789848	-1.70219
H	3.409528	0.068353	2.925471
H	8.745392	-1.455278	-1.384654
H	7.751685	-2.645314	-0.537717
H	7.620795	-2.48589	-2.285236
H	-10.351726	0.539961	1.045861
H	-10.508477	1.695636	-0.284603
H	-10.736833	-0.031515	-0.592719
H	3.602285	-5.336243	0.674842
H	4.183026	-4.742617	2.32723

Cartesian coordinates for conformer 2-47 after optimization at the PCM/B3LYP/6-31G* level of theory. Number of imaginary frequencies = 0. SCF Energy (PCM/mPW1PW91/6-31+G**) = -2695.11213942.

Atom	X	Y	Z
O	4.780948	1.674025	-2.853673
C	4.005252	2.14318	-1.84135
C	4.645641	3.360251	-1.162924
O	2.932476	1.646055	-1.537022
C	4.526208	3.281418	0.370413
C	5.120353	2.007223	1.002826
C	3.967071	4.619618	-1.698169
O	6.025746	3.419919	-1.575885
C	5.136131	2.142051	2.529704
C	5.85389	0.969857	3.181262
C	7.241269	0.787612	2.566466
C	7.135668	0.697223	1.01247
O	6.442564	1.836697	0.485544
O	6.468969	-0.542845	0.721395
C	6.509262	-0.924077	-0.664045
C	7.97385	-1.18265	-1.076978
C	8.912026	-0.119411	-0.573819
C	8.502733	0.737893	0.376747
C	5.642465	-2.202141	-0.834927
C	4.195016	-1.977118	-0.434804
C	5.669923	-2.745804	-2.267595
C	3.652901	-2.47698	0.690279
C	2.215114	-2.377566	1.124807
C	1.357305	-2.21165	-0.014937
C	0.536697	-1.058607	0.1955
C	1.330247	-0.177179	1.150795
C	1.959462	-1.205672	2.064995
C	0.245571	-0.367429	-1.140941
C	-0.686768	-1.197681	-2.023009
C	-1.92852	-1.597288	-1.234043
C	-1.493146	-2.298356	0.052052
O	-0.682572	-1.431675	0.860711
O	-2.755938	-2.442202	-2.03554
C	-3.974149	-2.886366	-1.387673
C	-3.651895	-3.52579	-0.050522
C	-2.751544	-2.695127	0.833549
C	-5.004522	-1.717673	-1.342597
C	-6.396282	-2.176627	-0.874908
C	-7.480203	-1.079688	-0.72089
C	-7.070721	0.098643	0.202286
C	-7.969501	-0.562996	-2.083236
O	-8.192333	0.992718	0.303139
C	-7.89842	2.229603	0.963715
C	-7.401787	1.958532	2.394076
C	-6.257265	0.948459	2.412279
C	-6.609456	-0.317419	1.617557
C	-9.198866	3.051129	1.040155
C	-9.64814	3.524655	-0.339193
C	-8.503771	4.239469	-1.042562
C	-7.256378	3.370201	-1.041895
O	-6.887801	2.984303	0.282003
H	-2.470621	-0.672716	-0.998289
H	-6.252302	0.637579	-0.291481
H	4.513596	1.14175	0.713724
O	7.824112	-0.392669	3.137578
H	6.085502	-0.111545	-1.266697
H	1.939349	-3.322746	1.606268
H	-0.92521	-3.213808	-0.165706
O	-2.377935	-3.40542	2.016499
H	-4.374587	-3.645662	-2.071986

O	-5.104482	-1.152209	-2.655513
C	-7.654371	-1.150323	2.37297
C	10.286349	-0.084888	-1.170098
C	-4.103079	-4.752149	0.272266
H	5.582374	2.252054	-2.899678
H	3.475507	3.368089	0.675049
H	5.060033	4.14785	0.7845
H	2.900396	4.640735	-1.450742
H	4.439275	5.520871	-1.291297
H	4.060694	4.68133	-2.788921
H	6.533001	2.888726	-0.920019
H	5.66185	3.062752	2.813701
H	4.11365	2.217229	2.916562
H	5.263185	0.052653	3.063493
H	5.941709	1.12916	4.262568
H	7.863731	1.643679	2.855116
H	8.036146	-1.238333	-2.169789
H	8.315613	-2.143877	-0.672591
H	9.173128	1.518635	0.727171
H	6.057177	-2.979399	-0.177396
H	3.576279	-1.400906	-1.121939
H	4.987669	-3.596388	-2.380241
H	5.372434	-1.977693	-2.99044
H	6.669078	-3.100011	-2.539915
H	4.292334	-3.031254	1.375242
H	0.712115	0.538746	1.700522
H	2.121318	0.366696	0.625535
H	1.238185	-1.503688	2.836224
H	2.851739	-0.834227	2.578184
H	-0.228238	0.604425	-0.955427
H	1.17434	-0.194042	-1.693043
H	-0.169039	-2.093518	-2.388689
H	-0.972653	-0.630888	-2.916938
H	-3.285903	-1.800196	1.173304
H	-4.633939	-0.922331	-0.691038
H	-6.300827	-2.692352	0.086538
H	-6.775064	-2.926016	-1.582865
H	-8.359778	-1.566993	-0.277898
H	-7.242538	0.105826	-2.552218
H	-8.171106	-1.391959	-2.769516
H	-8.903378	-0.001	-1.971998
H	-8.222228	1.565819	3.006958
H	-7.05962	2.888786	2.863671
H	-5.998757	0.696866	3.447531
H	-5.363344	1.40926	1.972628
H	-5.697805	-0.921528	1.544221
H	-10.003612	2.457952	1.490774
H	-9.04033	3.936504	1.669204
H	-9.973569	2.666751	-0.939899
H	-10.509071	4.195507	-0.243826
H	-8.292001	5.174837	-0.510181
H	-8.788073	4.49872	-2.067738
H	-6.416997	3.931407	-1.465066
H	-7.394645	2.480592	-1.665195
H	7.467888	-1.13646	2.611318
H	-1.625249	-2.907416	2.388026
H	-4.214324	-1.223295	-3.054813
H	-8.63425	-0.663657	2.395047
H	-7.783143	-2.134139	1.911908
H	-7.338196	-1.321061	3.407756
H	10.228486	0.094728	-2.24841
H	10.903756	0.705896	-0.731606
H	10.796135	-1.039046	-1.002563
H	-3.851702	-5.214858	1.223202
H	-4.729955	-5.329864	-0.400217

Cartesian coordinates for conformer 2-48 after optimization at the PCM/B3LYP/6-31G* level of theory. Number of imaginary frequencies = 0. SCF Energy (PCM/mPW1PW91/6-31+G**) = -2695.11301901.

Atom	X	Y	Z
O	6.386417	2.78281	-1.926351
C	5.986083	3.089551	-0.663645
C	7.184667	3.427904	0.23382
O	4.814598	3.091143	-0.319108
C	7.053843	2.778955	1.624048
C	6.881361	1.248164	1.604658
C	7.283548	4.947668	0.348646
O	8.378099	2.979091	-0.440359
C	6.913119	0.697883	3.034066
C	6.856393	-0.822157	3.032267
C	7.955514	-1.393104	2.137428
C	7.90536	-0.737672	0.721474
O	7.94329	0.694302	0.819695
O	6.701856	-1.207203	0.08893
C	6.642692	-0.92815	-1.320901
C	7.744035	-1.735897	-2.034746
C	9.073236	-1.599702	-1.340188
C	9.123687	-1.122423	-0.084159
C	5.224236	-1.301138	-1.829634
C	4.158458	-0.474371	-1.131764
C	5.073209	-1.109963	-3.341529
C	3.172746	-1.003826	-0.386833
C	2.080154	-0.272545	0.334466
C	0.828956	-0.883578	-0.027871
C	-0.186316	0.129259	-0.091468
C	0.460872	1.415372	0.418148
C	1.912327	1.214473	0.06563
C	-0.665707	0.264615	-1.548784
C	-1.445972	-0.970012	-2.002261
C	-2.549821	-1.282685	-0.996369
C	-1.933627	-1.407208	0.397308
O	-1.278414	-0.187684	0.780024
O	-3.22354	-2.482806	-1.379025
C	-4.302894	-2.877592	-0.49525
C	-3.808107	-2.946654	0.937022
C	-3.056016	-1.718645	1.394704
C	-5.542441	-1.963249	-0.734098
C	-6.795684	-2.464706	0.003661
C	-8.069653	-1.588894	-0.105136
C	-7.867664	-0.110326	0.320809
C	-8.705457	-1.67368	-1.501894
O	-9.140144	0.553542	0.233461
C	-9.073157	1.974325	0.406106
C	-8.47765	2.310643	1.783902
C	-7.155014	1.585865	2.019558
C	-7.278164	0.081433	1.737445
C	-10.50743	2.531619	0.339025
C	-11.095013	2.407855	-1.063867
C	-10.144093	3.01921	-2.082477
C	-8.749365	2.435706	-1.921622
O	-8.260231	2.606648	-0.591692
H	-3.256049	-0.44321	-1.024857
H	-7.192928	0.355451	-0.408473
H	5.922728	0.998797	1.135325
O	7.78158	-2.816246	2.079545
H	6.810243	0.145165	-1.476492
H	2.209197	-0.438498	1.411542
H	-1.205591	-2.229582	0.438357
O	-2.502202	-1.899694	2.700071
H	-4.571208	-3.884393	-0.840973

O	-5.804257	-1.915321	-2.142162
C	-8.106171	-0.603045	2.833857
C	10.290594	-2.018762	-2.106758
C	-3.994318	-4.042937	1.695446
H	7.372379	2.862815	-1.946107
H	6.211816	3.22231	2.170194
H	7.969661	3.011729	2.184615
H	6.396631	5.375749	0.827571
H	8.168556	5.239188	0.925321
H	7.387832	5.409085	-0.640562
H	8.542225	2.058908	-0.130905
H	7.842436	1.006846	3.529685
H	6.079321	1.102223	3.618745
H	5.871483	-1.162818	2.68784
H	6.964992	-1.209893	4.052159
H	8.924331	-1.192524	2.611779
H	7.836495	-1.389791	-3.070227
H	7.481615	-2.80095	-2.055403
H	10.083712	-1.002037	0.411248
H	5.046939	-2.361045	-1.600451
H	4.226641	0.603607	-1.265662
H	4.040287	-1.291073	-3.660788
H	5.345195	-0.09251	-3.64453
H	5.70601	-1.810416	-3.895478
H	3.137475	-2.085389	-0.263884
H	0.350359	1.497902	1.507047
H	0.032858	2.323756	-0.016748
H	2.586455	1.831002	0.666434
H	2.065666	1.461667	-0.991166
H	-1.327311	1.135719	-1.636794
H	0.180918	0.415203	-2.228155
H	-0.774077	-1.831661	-2.104266
H	-1.877082	-0.805843	-2.99689
H	-3.743064	-0.867961	1.471881
H	-5.311838	-0.939815	-0.427773
H	-6.562055	-2.58807	1.06662
H	-7.044654	-3.467447	-0.36888
H	-8.816317	-2.035492	0.565987
H	-8.145503	-1.095453	-2.242062
H	-8.764387	-2.711786	-1.845242
H	-9.727635	-1.279635	-1.487063
H	-9.179167	2.020842	2.575646
H	-8.309794	3.390658	1.875345
H	-6.811797	1.760749	3.045955
H	-6.38845	2.011796	1.359492
H	-6.267999	-0.342032	1.77567
H	-11.159691	2.003073	1.044366
H	-10.504199	3.594232	0.614324
H	-11.266059	1.351642	-1.304511
H	-12.068066	2.909329	-1.108939
H	-10.102773	4.103288	-1.91976
H	-10.513802	2.850664	-3.099194
H	-8.055633	2.950512	-2.594315
H	-8.731467	1.374858	-2.192373
H	7.103116	-2.964919	1.390592
H	-1.827223	-1.200143	2.792724
H	-4.934219	-1.968135	-2.586145
H	-9.161949	-0.317659	2.793869
H	-8.053534	-1.692341	2.746
H	-7.722958	-0.343441	3.826658
H	10.396127	-1.413768	-3.012966
H	11.206197	-1.902074	-1.517623
H	10.211584	-3.071078	-2.397512
H	-3.619174	-4.095744	2.714399
H	-4.520277	-4.917139	1.323831

Cartesian coordinates for conformer 3-1 after optimization at the PCM/B3LYP/6-31G* level of theory. Number of imaginary frequencies = 0. SCF Energy (PCM/mPW1PW91/6-31+G**) = -2647.61115821.

Atom	X	Y	Z
O	-2.5361	1.7609	-2.6253
O	-1.2432	1.1578	-0.8614
O	-3.7372	-0.6548	-0.5243
O	1.3732	1.0866	-0.1639
O	-2.1969	-2.6794	-2.0299
H	-1.5964	-3.2713	-2.5291
O	3.2485	0.8837	-2.0315
O	5.2188	1.7653	0.0463
O	-2.2758	-0.2111	3.3634
O	-1.9593	-2.2156	2.323
O	3.7949	0.2015	0.8781
O	-0.1134	-4.1295	-1.7309
O	0.7275	0.0487	1.7491
C	-2.7388	-3.1671	0.3039
H	-2.458	-4.0849	0.8257
H	-3.6376	-3.3672	-0.2823
C	-3.0055	-2.0332	1.3061
C	-2.6979	-0.7776	0.4683
H	-2.6327	0.1332	1.0541
C	-1.4099	-1.1615	-0.2623
H	-0.6322	-1.147	0.4997
C	-1.0521	-0.1895	-1.3905
H	-1.7466	-0.3364	-2.2145
C	0.3796	-0.2785	-1.8992
C	1.4801	-0.1823	-0.8553
H	1.3102	-0.9609	-0.1211
C	2.916	-0.3433	-1.3544
H	2.9439	-1.166	-2.0698
C	3.9813	-0.6506	-0.2764
H	4.9525	-0.4117	-0.7138
C	4.0419	-2.1328	0.22
C	2.674	-2.559	0.7178
H	2.2967	-1.9712	1.5555
C	1.8818	-3.4778	0.1621
H	2.2145	-4.0624	-0.692
C	0.4446	-3.7331	0.5586
H	0.1265	-3.037	1.3377
C	-0.4071	-3.5363	-0.7023
C	-1.6651	-2.6427	-0.722
C	-4.3772	-2.0565	1.9626
H	-4.5083	-2.9861	2.5263
H	-5.1549	-1.9997	1.1966
H	-4.4856	-1.206	2.639
C	0.6013	-0.467	-3.2029
H	1.5963	-0.5226	-3.6326
H	-0.2289	-0.5733	-3.8951
C	4.5685	-3.009	-0.9325
H	3.9144	-3.0031	-1.8087
H	4.6668	-4.0466	-0.5973
H	5.5531	-2.6591	-1.2586
C	5.05	-2.2083	1.3892
H	4.7027	-1.6376	2.2542
H	6.0269	-1.812	1.0866
H	5.184	-3.2511	1.6958
C	0.2639	-5.1807	1.0706
H	0.5305	-5.8956	0.2861
H	-0.7678	-5.3751	1.3812
H	0.9179	-5.3462	1.9325
C	-1.6942	-1.2693	3.2605
C	-0.5441	-1.7055	4.1354

H	0.3758	-1.325	3.6794
H	-0.4837	-2.7934	4.2118
H	-0.6552	-1.2551	5.124
C	0.9708	1.0669	1.1182
C	0.8489	2.4305	1.6838
C	0.1671	2.5782	2.8983
H	-0.2829	1.7051	3.362
C	0.0365	3.8433	3.4648
H	-0.5051	3.9637	4.3986
C	0.5898	4.9561	2.8269
H	0.4877	5.9424	3.2723
C	1.2667	4.8082	1.613
H	1.6922	5.6761	1.1166
C	1.3934	3.5474	1.0353
H	1.9159	3.4154	0.0933
C	4.4945	1.3625	0.9301
C	4.2849	2.0543	2.2564
H	5.1787	1.8978	2.8712
H	3.414	1.6652	2.7849
H	4.1716	3.1276	2.0903
C	-1.9086	2.0588	-1.6297
C	-4.526	0.4508	-0.5199
O	-4.5036	1.2913	0.3523
C	-1.7307	3.4656	-1.0797
C	-2.3429	3.6062	0.3268
C	-2.2955	4.5004	-2.0553
H	-0.6443	3.6006	-0.9833
H	-3.4289	3.4892	0.2911
H	-1.947	2.8543	1.0118
H	-2.1026	4.5962	0.7293
H	-1.8554	4.3974	-3.052
H	-3.3794	4.3834	-2.1571
H	-2.0928	5.5113	-1.6852
C	4.2841	0.8331	-2.9056
O	4.8311	-0.1995	-3.2306
C	4.653	2.2199	-3.3571
H	5.3455	2.1641	-4.1979
H	3.7594	2.7892	-3.6287
H	5.1309	2.7257	-2.5105
C	-5.4353	0.4453	-1.736
C	-5.9698	1.8518	-2.012
C	-6.5619	-0.583	-1.5124
H	-6.5134	2.2395	-1.1444
H	-5.1444	2.5338	-2.2358
H	-6.6477	1.8356	-2.8727
H	-7.2126	-0.625	-2.3924
H	-6.1536	-1.5842	-1.3401
H	-7.1777	-0.3028	-0.6489
H	-4.8203	0.1126	-2.5789

Cartesian coordinates for conformer 3-2 after optimization at the PCM/B3LYP/6-31G* level of theory. Number of imaginary frequencies = 0. SCF Energy (PCM/mPW1PW91/6-31+G**) = -2647.60997669.

Atom	X	Y	Z
O	-2.334136	1.82117	-2.668511
O	-1.412202	1.105685	-0.726326
O	-3.885331	-0.770802	-0.265225
O	1.242447	1.104815	-0.157796
O	-2.397226	-2.731202	-1.862813
H	-1.803972	-3.278125	-2.418758
O	3.04967	0.896268	-2.07815
O	5.054354	1.824832	-0.046013
O	-2.253357	-0.260307	3.52753
O	-1.911329	-2.255196	2.476182
O	3.695838	0.225692	0.826831

O	-0.243662	-4.107498	-1.732975
O	0.658235	0.08891	1.786503
C	-2.765493	-3.242078	0.49995
H	-2.419571	-4.142838	1.012013
H	-3.691614	-3.480607	-0.025791
C	-3.009307	-2.108486	1.510634
C	-2.77695	-0.849548	0.65395
H	-2.700456	0.068825	1.22629
C	-1.517518	-1.210224	-0.1396
H	-0.708855	-1.191363	0.590128
C	-1.213934	-0.231171	-1.275565
H	-1.931709	-0.387026	-2.077903
C	0.200584	-0.296557	-1.835335
C	1.33553	-0.175792	-0.831664
H	1.200361	-0.941629	-0.077378
C	2.754795	-0.329024	-1.380181
H	2.764375	-1.157106	-2.089752
C	3.860696	-0.616126	-0.338267
H	4.812615	-0.356248	-0.804989
C	3.964477	-2.098691	0.148501
C	2.616215	-2.558739	0.668749
H	2.254184	-2.003922	1.535501
C	1.822767	-3.472055	0.106182
H	2.139715	-4.022571	-0.776269
C	0.408048	-3.775187	0.544693
H	0.103082	-3.114724	1.359949
C	-0.501343	-3.557925	-0.671019
C	-1.776174	-2.692666	-0.593431
C	-4.34776	-2.168452	2.231267
H	-4.42828	-3.103358	2.795682
H	-5.160389	-2.128609	1.501414
H	-4.445801	-1.324871	2.918183
C	0.378838	-0.462342	-3.148546
H	1.359077	-0.494806	-3.613269
H	-0.473923	-0.566766	-3.812752
C	4.489624	-2.956475	-1.018893
H	3.830488	-2.944583	-1.891146
H	4.599692	-3.997124	-0.696931
H	5.468407	-2.593194	-1.347362
C	4.995376	-2.160095	1.298289
H	4.650928	-1.606176	2.175302
H	5.956309	-1.736901	0.981382
H	5.161128	-3.202096	1.592086
C	0.285189	-5.244739	1.010625
H	0.546456	-5.923012	0.192557
H	-0.729693	-5.482774	1.345997
H	0.971712	-5.420509	1.844974
C	-1.637992	-1.296631	3.39995
C	-0.435162	-1.688651	4.222603
H	0.450499	-1.290776	3.717135
H	-0.341671	-2.773359	4.310478
H	-0.510991	-1.227068	5.209359
C	0.889693	1.099395	1.139236
C	0.811454	2.468235	1.700868
C	0.157827	2.636839	2.928117
H	-0.303411	1.77629	3.404325
C	0.072694	3.906327	3.493918
H	-0.447007	4.04355	4.437938
C	0.643786	5.002435	2.842756
H	0.576548	5.992125	3.287327
C	1.295057	4.833364	1.617642
H	1.735961	5.687836	1.111741
C	1.376605	3.568119	1.04127
H	1.881879	3.41981	0.092201
C	4.380541	1.396726	0.865519
C	4.230411	2.060112	2.21408

H	5.139801	1.866423	2.794913
H	3.369328	1.675707	2.761949
H	4.136137	3.139288	2.079036
C	-1.866335	2.064681	-1.575208
C	-4.589572	0.386606	-0.358619
O	-4.418135	1.337196	0.372074
C	-1.653802	3.453647	-0.996682
C	-2.336922	3.632422	0.370241
C	-2.088908	4.523737	-2.000572
H	-0.568641	3.520564	-0.836659
H	-3.424432	3.598292	0.267
H	-2.045998	2.847582	1.069632
H	-2.047582	4.599253	0.795997
H	-1.579325	4.40483	-2.961241
H	-3.166275	4.461256	-2.187227
H	-1.865469	5.520461	-1.604551
C	4.059699	0.854655	-2.981586
O	4.61356	-0.172004	-3.314285
C	4.391634	2.243477	-3.455321
H	3.481087	2.791397	-3.714887
H	4.876245	2.768834	-2.624518
H	5.067451	2.192246	-4.309795
C	-5.618394	0.301029	-1.478633
C	-6.238422	1.680481	-1.722161
C	-5.018788	-0.300167	-2.764187
H	-5.475539	2.373682	-2.094023
H	-6.655601	2.101202	-0.803005
H	-7.033307	1.609543	-2.472133
H	-4.23323	0.354859	-3.153883
H	-5.801719	-0.393283	-3.525321
H	-4.58125	-1.284679	-2.581726
H	-6.394045	-0.387378	-1.107416

Cartesian coordinates for conformer 3-3 after optimization at the PCM/B3LYP/6-31G* level of theory. Number of imaginary frequencies = 0. SCF Energy (PCM/mPW1PW91/6-31+G**) = -2647.61312288.

Atom	X	Y	Z
O	-0.08567	-1.499185	-3.716251
O	-1.178418	-0.101112	-2.30122
O	-3.379298	-0.339667	-0.481371
O	1.442875	1.401183	1.421967
O	-2.244674	-2.681646	-1.951986
H	-1.710146	-3.282134	-2.512503
O	3.233345	1.757165	-0.424748
O	4.909194	1.70081	2.017147
O	-2.768956	-0.272746	3.582815
O	-2.13573	-2.153209	2.459875
O	3.637632	-0.181432	2.004685
O	-0.314965	-4.378465	-1.779363
O	0.093256	0.047653	2.62341
C	-2.6975	-3.145195	0.38739
H	-2.390581	-3.987355	1.014705
H	-3.591345	-3.440268	-0.164926
C	-2.975938	-1.915391	1.280981
C	-2.38666	-0.718227	0.489886
H	-2.151269	0.13043	1.128577
C	-1.151495	-1.331551	-0.183349
H	-0.477546	-1.508255	0.658812
C	-0.343656	-0.505307	-1.206028
H	0.433892	-1.168662	-1.60639
C	0.324923	0.718078	-0.611403
C	1.439384	0.415045	0.370021
H	1.272337	-0.557498	0.823238
C	2.840012	0.385502	-0.251505
H	2.774796	-0.084009	-1.235749

C	3.875266	-0.396344	0.591465
H	4.867424	-0.012499	0.350323
C	3.88812	-1.939801	0.366389
C	2.551387	-2.547403	0.759192
H	2.213287	-2.285602	1.763173
C	1.796443	-3.358139	0.013358
H	2.113116	-3.653223	-0.984796
C	0.466408	-3.951459	0.43873
H	0.085738	-3.431889	1.324323
C	-0.482442	-3.750741	-0.745179
C	-1.631918	-2.733245	-0.678246
C	-4.444523	-1.782717	1.669416
H	-4.736727	-2.643847	2.279948
H	-5.060019	-1.769337	0.767671
H	-4.62115	-0.869083	2.236829
C	-0.009891	1.97125	-0.916549
H	0.502584	2.806761	-0.452585
H	-0.818129	2.191934	-1.60091
C	4.280669	-2.233575	-1.094346
H	5.182216	-1.677458	-1.367275
H	3.497798	-1.960271	-1.807842
H	4.482931	-3.302155	-1.220754
C	4.975739	-2.536855	1.294104
H	4.744595	-2.360056	2.347757
H	5.954215	-2.090925	1.076903
H	5.047728	-3.617586	1.134467
C	0.598876	-5.456531	0.748192
H	0.950221	-5.99544	-0.136191
H	-0.365922	-5.881899	1.048763
H	1.314253	-5.605181	1.562587
C	-2.13274	-1.301615	3.517026
C	-1.230823	-1.843661	4.599402
H	-1.263511	-1.178465	5.463534
H	-0.209158	-1.896451	4.215587
H	-1.54733	-2.851322	4.887851
C	0.671071	1.113926	2.487317
C	0.617552	2.230183	3.460193
C	-0.372209	2.19304	4.451461
H	-1.091275	1.378821	4.451995
C	-0.442017	3.219675	5.389906
H	-1.215547	3.20201	6.152644
C	0.474807	4.272618	5.346526
H	0.419755	5.069953	6.083529
C	1.458258	4.310081	4.354362
H	2.167862	5.13223	4.320552
C	1.528289	3.295184	3.403624
H	2.288158	3.31192	2.628649
C	4.220573	0.890764	2.596605
C	3.924508	0.891496	4.077183
H	4.614523	0.199975	4.575136
H	2.905297	0.553105	4.275797
H	4.072084	1.894133	4.479084
C	-1.000105	-0.737377	-3.4865
C	-3.572772	0.981746	-0.71452
O	-2.998692	1.868986	-0.122583
C	-2.1038	-0.363447	-4.460057
C	-1.959371	-1.143423	-5.767943
C	-2.139959	1.158625	-4.690689
H	-3.031636	-0.665055	-3.95508
H	-1.952624	-2.222104	-5.586157
H	-2.789812	-0.907322	-6.442313
H	-1.020382	-0.887731	-6.269894
H	-2.246923	1.704546	-3.750001
H	-1.218246	1.496165	-5.179151
H	-2.981597	1.422194	-5.34074
C	4.307657	1.991068	-1.214537

O	4.8739	1.126141	-1.849237
C	4.699615	3.442666	-1.140658
H	5.455557	3.660658	-1.895816
H	3.825129	4.08641	-1.27308
H	5.10438	3.629136	-0.139438
C	-4.60563	1.182067	-1.813897
C	-4.742166	2.666542	-2.159098
C	-5.952241	0.549025	-1.417671
H	-5.115043	3.231385	-1.298399
H	-3.778234	3.100046	-2.441554
H	-5.441064	2.798144	-2.992357
H	-6.683344	0.689613	-2.221508
H	-5.844486	-0.523023	-1.232019
H	-6.352117	1.020208	-0.51201
H	-4.222369	0.635711	-2.684463

Cartesian coordinates for conformer 3-4 after optimization at the PCM/B3LYP/6-31G* level of theory. Number of imaginary frequencies = 0. SCF Energy (PCM/mPW1PW91/6-31+G**) = -2647.61324467.

Atom	X	Y	Z
O	-2.645615	1.914057	-2.413084
O	-1.202725	1.250878	-0.789727
O	-3.759596	-0.569525	-0.682788
O	1.456259	1.243008	-0.249788
O	-2.054041	-2.561973	-2.132826
H	-1.440715	-3.175047	-2.588609
O	3.29101	0.754741	-2.109017
O	5.327068	1.789919	-0.110871
O	-2.576141	-4.042767	3.048744
O	-2.194728	-1.944218	2.258264
O	3.834872	0.385204	0.871864
O	-0.002811	-4.032902	-1.694623
O	0.711157	0.443298	1.74424
C	-2.739687	-3.138355	0.144628
H	-2.47925	-4.064743	0.652662
H	-3.586839	-3.346398	-0.512377
C	-3.137925	-2.034637	1.138384
C	-2.794624	-0.747381	0.366634
H	-2.772109	0.129667	1.011587
C	-1.444862	-1.075316	-0.281848
H	-0.720264	-1.03609	0.53298
C	-1.041449	-0.076364	-1.371079
H	-1.726563	-0.168383	-2.210394
C	0.39223	-0.222905	-1.858013
C	1.488394	-0.088219	-0.813443
H	1.270416	-0.780918	-0.008115
C	2.915438	-0.369413	-1.288076
H	2.902143	-1.27374	-1.897932
C	3.986359	-0.582629	-0.192077
H	4.955617	-0.405185	-0.661621
C	4.029142	-2.013025	0.438692
C	2.633936	-2.416891	0.872139
H	2.208561	-1.80043	1.666188
C	1.876951	-3.352599	0.297194
H	2.261494	-3.963511	-0.515622
C	0.421881	-3.604318	0.617548
H	0.0708	-2.881751	1.358512
C	-0.355717	-3.427683	-0.69241
C	-1.618772	-2.550068	-0.787511
C	-4.564341	-2.106672	1.661845
H	-4.724075	-3.044847	2.196859
H	-5.266805	-2.050469	0.826443
H	-4.758496	-1.265565	2.334593
C	0.616009	-0.533116	-3.137812
H	1.61059	-0.6705	-3.549042

H	-0.212979	-0.674095	-3.824805
C	4.638399	-2.972618	-0.602276
H	4.088087	-2.988776	-1.546491
H	4.658717	-3.991789	-0.203155
H	5.666064	-2.675159	-0.834022
C	4.958081	-1.983828	1.672248
H	4.543105	-1.367207	2.4735
H	5.943791	-1.582697	1.407134
H	5.095185	-3.001036	2.054912
C	0.216606	-5.039282	1.15789
H	0.864147	-5.192323	2.02712
H	0.486222	-5.770198	0.389602
H	-0.815846	-5.214885	1.472284
C	-2.011317	-2.970951	3.115587
C	-1.001722	-2.580142	4.170694
H	-0.195033	-1.983433	3.736081
H	-0.611228	-3.479011	4.649708
H	-1.497699	-1.960497	4.926348
C	1.065136	1.367884	1.031501
C	1.129707	2.783036	1.480666
C	0.784031	3.070327	2.80592
H	0.467421	2.255775	3.449518
C	0.847505	4.379797	3.27327
H	0.579422	4.601884	4.302308
C	1.252619	5.406792	2.417206
H	1.301032	6.42947	2.782103
C	1.596589	5.122628	1.093171
H	1.912532	5.922005	0.428616
C	1.538958	3.812576	0.622366
H	1.816723	3.576042	-0.398999
C	4.593848	1.509136	0.811947
C	4.435028	2.339108	2.064144
H	5.394574	2.350269	2.592496
H	3.658562	1.944819	2.720018
H	4.192672	3.36751	1.785388
C	-1.983641	2.159424	-1.425881
C	-4.635585	0.464178	-0.598032
O	-4.709607	1.205645	0.358868
C	-1.88869	3.510855	-0.732062
C	-2.296158	3.412536	0.750658
C	-2.704947	4.56555	-1.48147
H	-0.823088	3.777187	-0.76621
H	-3.354605	3.154487	0.842066
H	-1.711865	2.651529	1.272159
H	-2.118494	4.374455	1.243896
H	-2.423822	4.615579	-2.537454
H	-3.773639	4.332817	-1.430236
H	-2.547399	5.550284	-1.028085
C	4.305905	0.552715	-2.984746
O	4.794095	-0.536823	-3.199003
C	4.736305	1.858689	-3.594943
H	5.240387	2.438518	-2.813499
H	5.420705	1.672853	-4.423426
H	3.867968	2.432456	-3.931824
C	-5.503436	0.512869	-1.841087
C	-6.541881	-0.624837	-1.764766
C	-6.149001	1.890686	-1.991995
H	-7.200457	-0.493251	-0.897487
H	-6.051992	-1.600732	-1.68677
H	-7.165041	-0.625291	-2.665447
H	-6.750069	2.138129	-1.111271
H	-6.796277	1.909716	-2.875638
H	-5.379373	2.658664	-2.11115
H	-4.836213	0.322168	-2.688059

Cartesian coordinates for conformer 3-5 after optimization at the PCM/B3LYP/6-31G* level of theory. Number of imaginary frequencies = 0. SCF Energy (PCM/mPW1PW91/6-31+G**) = -2647.61073739.

Atom	X	Y	Z
O	0.183196	-1.574179	-3.62268
O	-1.052175	-0.164671	-2.341936
O	-3.345109	-0.345547	-0.547254
O	1.456571	1.4037	1.451102
O	-2.200289	-2.699874	-1.950674
H	-1.68351	-3.338159	-2.484972
O	3.212452	1.775325	-0.425456
O	4.95493	1.702264	1.960894
O	-2.814743	-0.218359	3.535052
O	-2.19223	-2.127184	2.454603
O	3.680994	-0.178587	1.974681
O	-0.335166	-4.454989	-1.701237
O	0.083603	0.057515	2.634751
C	-2.713152	-3.139031	0.38298
H	-2.426815	-3.974602	1.028748
H	-3.596001	-3.435156	-0.186214
C	-3.001684	-1.895086	1.253458
C	-2.383493	-0.713601	0.458939
H	-2.160924	0.145472	1.088998
C	-1.141812	-1.345995	-0.176205
H	-0.488338	-1.514559	0.683969
C	-0.29732	-0.537512	-1.179209
H	0.510479	-1.200767	-1.510812
C	0.312852	0.706768	-0.568049
C	1.442586	0.420948	0.39811
H	1.289926	-0.553821	0.852509
C	2.828199	0.401036	-0.25545
H	2.741986	-0.058658	-1.242591
C	3.881663	-0.388422	0.554832
H	4.868647	-0.007637	0.288858
C	3.881315	-1.930789	0.321247
C	2.557355	-2.539123	0.756458
H	2.243926	-2.270148	1.766416
C	1.789438	-3.361091	0.036528
H	2.086451	-3.663866	-0.965191
C	0.468894	-3.954811	0.491939
H	0.092081	-3.415975	1.36774
C	-0.485913	-3.784643	-0.691886
C	-1.61941	-2.747713	-0.663334
C	-4.47721	-1.742142	1.606199
H	-4.795711	-2.596732	2.212894
H	-5.070794	-1.723769	0.689675
H	-4.65372	-0.82381	2.166479
C	-0.085214	1.947974	-0.843404
H	0.384375	2.798132	-0.360711
H	-0.902814	2.142862	-1.524899
C	4.219332	-2.216671	-1.155101
H	5.103969	-1.650622	-1.461266
H	3.405961	-1.950453	-1.83658
H	4.42783	-3.28261	-1.29333
C	4.999523	-2.53493	1.206782
H	4.809181	-2.357039	2.268579
H	5.97171	-2.094621	0.9528
H	5.058772	-3.615988	1.044272
C	0.614913	-5.450353	0.836288
H	0.9616	-6.008802	-0.037634
H	-0.3444	-5.874401	1.156334
H	1.338677	-5.574227	1.647399
C	-2.201059	-1.262214	3.500722
C	-1.332729	-1.80182	4.611255
H	-1.466185	-1.189055	5.503782
H	-0.289598	-1.750885	4.288445

H	-1.582386	-2.845215	4.825475
C	0.676666	1.117049	2.511892
C	0.633098	2.224331	3.494653
C	1.558814	3.277297	3.454336
H	2.322771	3.292122	2.683467
C	1.496221	4.283368	4.414841
H	2.216745	5.096461	4.39349
C	0.506147	4.249211	5.400451
H	0.457103	5.039776	6.145066
C	-0.425248	3.208451	5.427701
H	-1.2039	3.193922	6.185389
C	-0.363476	2.1907	4.479426
H	-1.094118	1.386942	4.466051
C	4.278516	0.892194	2.55449
C	4.012668	0.891715	4.040598
H	2.976938	0.617422	4.253076
H	4.662887	0.148886	4.517257
H	4.230922	1.877528	4.451561
C	-0.71102	-0.762162	-3.511136
C	-3.642212	0.966806	-0.705719
O	-3.133887	1.860614	-0.064985
C	-1.606727	-0.300493	-4.646769
C	-1.766596	1.227231	-4.67426
C	-2.960961	-1.029755	-4.545682
H	-1.098024	-0.625515	-5.560476
H	-0.795138	1.728928	-4.747342
H	-2.369147	1.524776	-5.539627
H	-2.261467	1.590372	-3.769195
H	-2.834106	-2.109526	-4.6703
H	-3.417049	-0.869002	-3.566353
H	-3.639936	-0.668789	-5.326466
C	4.259954	2.020004	-1.247264
O	4.81223	1.161916	-1.902987
C	4.641961	3.474545	-1.180908
H	5.08025	3.660822	-0.194025
H	5.368853	3.702183	-1.961435
H	3.757597	4.110828	-1.279548
C	-4.671011	1.162482	-1.812859
C	-5.22842	2.588434	-1.771829
C	-5.775482	0.093761	-1.781324
H	-5.80055	2.754532	-0.852276
H	-4.422746	3.326785	-1.796411
H	-5.892356	2.759232	-2.625783
H	-6.482927	0.261989	-2.600782
H	-5.358529	-0.911332	-1.882997
H	-6.334361	0.140938	-0.83902
H	-4.109016	1.031579	-2.747243

Cartesian coordinates for conformer 3-6 after optimization at the PCM/B3LYP/6-31G* level of theory. Number of imaginary frequencies = 0. SCF Energy (PCM/mPW1PW91/6-31+G**) = -2647.61134408.

Atom	X	Y	Z
O	-2.578913	1.933177	-2.42779
O	-1.343911	1.209299	-0.669493
O	-3.896169	-0.674655	-0.470834
O	1.335242	1.212788	-0.215055
O	-2.224343	-2.617653	-1.948881
H	-1.627086	-3.226872	-2.430536
O	3.104254	0.779127	-2.144269
O	5.212445	1.805787	-0.213642
O	-2.470834	-4.024969	3.276284
O	-2.183605	-1.93314	2.431058
O	3.770239	0.371921	0.800662
O	-0.13858	-4.062206	-1.599721
O	0.697496	0.379419	1.802063

C	-2.7894	-3.182936	0.365963
H	-2.485272	-4.091	0.883124
H	-3.659599	-3.425488	-0.247671
C	-3.16861	-2.066005	1.353269
C	-2.882581	-0.789373	0.541692
H	-2.859888	0.107568	1.158239
C	-1.546552	-1.107784	-0.14406
H	-0.798429	-1.052571	0.648182
C	-1.186523	-0.117419	-1.253574
H	-1.898157	-0.221962	-2.068612
C	0.228822	-0.249168	-1.796541
C	1.364169	-0.112126	-0.795471
H	1.188539	-0.816924	0.009687
C	2.775932	-0.365035	-1.330809
H	2.751813	-1.258326	-1.956151
C	3.892011	-0.579989	-0.281118
H	4.839818	-0.385112	-0.786353
C	3.976724	-2.015392	0.33261
C	2.607967	-2.426822	0.835479
H	2.218592	-1.810655	1.647833
C	1.82702	-3.365398	0.298986
H	2.172955	-3.977177	-0.530307
C	0.388794	-3.616279	0.688476
H	0.070629	-2.886701	1.437894
C	-0.449086	-3.453591	-0.58573
C	-1.723825	-2.589343	-0.625915
C	-4.571375	-2.157109	1.933701
H	-4.695013	-3.094642	2.479853
H	-5.305976	-2.114876	1.125224
H	-4.750924	-1.316875	2.612217
C	0.404914	-0.531266	-3.090194
H	1.383994	-0.647311	-3.543244
H	-0.449256	-0.664868	-3.74722
C	4.535259	-2.963805	-0.74585
H	3.928978	-2.985971	-1.655096
H	4.59243	-3.983673	-0.351906
H	5.543111	-2.652264	-1.038222
C	4.96927	-1.988988	1.515759
H	4.593998	-1.379732	2.341796
H	5.937753	-1.58086	1.201752
H	5.131643	-3.007699	1.884219
C	0.212392	-5.046079	1.252471
H	0.445131	-5.784526	0.479075
H	-0.80263	-5.21995	1.619925
H	0.902666	-5.189169	2.089812
C	-1.930765	-2.938837	3.29543
C	-0.87944	-2.500959	4.290211
H	-1.362293	-1.919979	5.084714
H	-0.136831	-1.854717	3.813993
H	-0.411187	-3.381158	4.733466
C	1.00672	1.317193	1.085428
C	1.077303	2.72706	1.549308
C	0.742879	3.000298	2.880438
H	0.43206	2.178797	3.518156
C	0.807987	4.304929	3.36086
H	0.546438	4.51625	4.394031
C	1.205521	5.341294	2.51247
H	1.254953	6.360365	2.887459
C	1.540787	5.070734	1.183258
H	1.851736	5.877252	0.525016
C	1.479875	3.765655	0.699224
H	1.751339	3.540414	-0.326566
C	4.519013	1.502115	0.732405
C	4.407479	2.304267	2.007609
H	5.378961	2.279787	2.513666
H	3.638444	1.91024	2.67221

H	4.182824	3.344552	1.760293
C	-2.004816	2.155369	-1.381125
C	-4.685443	0.428071	-0.498557
O	-4.646864	1.296526	0.347494
C	-1.888189	3.515621	-0.709541
C	-2.2997	3.473375	0.772994
C	-2.673936	4.569884	-1.492395
H	-0.815408	3.753932	-0.74914
H	-3.367331	3.261109	0.86756
H	-1.749001	2.70368	1.316792
H	-2.082934	4.44061	1.239619
H	-2.371043	4.598263	-2.542943
H	-3.746169	4.349401	-1.459164
H	-2.512837	5.559716	-1.051521
C	4.09036	0.612036	-3.059661
O	4.587116	-0.464858	-3.31468
C	4.478274	1.938068	-3.65487
H	5.000598	2.508306	-2.878371
H	5.135796	1.782293	-4.510998
H	3.589607	2.504758	-3.948107
C	-5.636337	0.398993	-1.688847
C	-6.102208	1.825358	-2.008989
C	-5.042885	-0.304068	-2.920828
H	-5.254414	2.418275	-2.369032
H	-6.514129	2.31656	-1.123411
H	-6.868174	1.804274	-2.791519
H	-4.18164	0.259451	-3.290989
H	-5.797171	-0.34761	-3.714601
H	-4.710677	-1.319304	-2.692145
H	-6.501513	-0.186595	-1.336743

Cartesian coordinates for conformer 3-7 after optimization at the PCM/B3LYP/6-31G* level of theory. Number of imaginary frequencies = 0. SCF Energy (PCM/mPW1PW91/6-31+G**) = -2647.61087291.

Atom	X	Y	Z
O	0.097955	-1.600647	-3.624529
O	-1.089634	-0.145453	-2.348169
O	-3.404186	-0.362559	-0.467495
O	1.46021	1.433643	1.415644
O	-2.210452	-2.704351	-1.949592
H	-1.663745	-3.305603	-2.496957
O	3.203097	1.807318	-0.468707
O	4.972867	1.74418	1.906254
O	-2.710826	-0.287453	3.596406
O	-2.102324	-2.165312	2.454883
O	3.693075	-0.132381	1.934026
O	-0.307856	-4.421154	-1.715917
O	0.137675	0.06884	2.633786
C	-2.697965	-3.14418	0.387725
H	-2.412497	-4.000251	1.006419
H	-3.592968	-3.413604	-0.175439
C	-2.960769	-1.920302	1.29146
C	-2.386126	-0.720445	0.489389
H	-2.153864	0.137008	1.118732
C	-1.151565	-1.330348	-0.183188
H	-0.481308	-1.496012	0.664583
C	-0.322541	-0.513678	-1.19294
H	0.483578	-1.175973	-1.532092
C	0.29734	0.731604	-0.591471
C	1.438593	0.449451	0.363468
H	1.291572	-0.524897	0.820862
C	2.822183	0.432037	-0.295271
H	2.734788	-0.031044	-1.281034
C	3.883714	-0.349266	0.513871
H	4.867136	0.034651	0.239315

C	3.891	-1.893197	0.291144
C	2.571601	-2.504633	0.735086
H	2.259697	-2.229316	1.743909
C	1.804966	-3.333817	0.02232
H	2.10096	-3.641394	-0.978314
C	0.485909	-3.926752	0.481392
H	0.109631	-3.387208	1.35667
C	-0.469787	-3.76028	-0.702543
C	-1.617946	-2.739006	-0.667569
C	-4.422729	-1.789968	1.706337
H	-4.703535	-2.65162	2.321651
H	-5.053465	-1.778251	0.814863
H	-4.590429	-0.877371	2.278513
C	-0.101744	1.972263	-0.86623
H	0.377517	2.823135	-0.394104
H	-0.93092	2.165775	-1.533553
C	4.22591	-2.187166	-1.184175
H	5.107688	-1.620123	-1.496214
H	3.409514	-1.927696	-1.864738
H	4.437356	-3.253245	-1.316613
C	5.015721	-2.485475	1.176304
H	4.826884	-2.304852	2.237956
H	5.984253	-2.039804	0.918104
H	5.081768	-3.566797	1.018216
C	0.632063	-5.421956	0.827757
H	0.976155	-5.981958	-0.046273
H	-0.326637	-5.844847	1.151177
H	1.357989	-5.545204	1.63722
C	-2.085886	-1.322469	3.519516
C	-1.173077	-1.872647	4.588296
H	-1.303067	-1.295874	5.505093
H	-0.140815	-1.77399	4.242077
H	-1.382036	-2.930786	4.770901
C	0.721241	1.132521	2.499603
C	0.712855	2.226474	3.498568
C	-0.232968	2.167273	4.530926
H	-0.955999	1.356123	4.53963
C	-0.253736	3.166356	5.501062
H	-0.991974	3.130759	6.297504
C	0.668443	4.214522	5.446777
H	0.652798	4.989564	6.208935
C	1.606412	4.275041	4.412839
H	2.319737	5.09366	4.371102
C	1.627439	3.28752	3.43162
H	2.35283	3.321269	2.625144
C	4.304576	0.933242	2.507948
C	4.070053	0.924839	3.999436
H	4.765596	0.214176	4.461682
H	3.053225	0.604844	4.236507
H	4.254196	1.920142	4.404476
C	-0.780677	-0.771567	-3.512129
C	-3.711969	0.947837	-0.61719
O	-3.103797	1.859232	-0.099516
C	-1.670162	-0.289281	-4.642417
C	-1.526405	1.230048	-4.83766
C	-3.129834	-0.695115	-4.377816
H	-1.303621	-0.80527	-5.535764
H	-0.484885	1.513327	-5.027945
H	-2.129842	1.558727	-5.691174
H	-1.865993	1.768346	-3.947525
H	-3.231359	-1.77893	-4.27379
H	-3.48204	-0.246695	-3.44693
H	-3.769255	-0.356607	-5.201106
C	4.249883	2.052375	-1.291834
O	4.798867	1.195143	-1.95153
C	4.637089	3.505253	-1.219721

H	3.754891	4.145553	-1.311829
H	5.079702	3.684393	-0.233352
H	5.362196	3.734141	-2.001475
C	-4.966423	1.118332	-1.459051
C	-4.830285	2.333852	-2.382078
C	-6.159362	1.271995	-0.492998
H	-4.613845	3.230787	-1.79396
H	-4.017959	2.197985	-3.103905
H	-5.758988	2.494375	-2.940649
H	-7.091337	1.381458	-1.058563
H	-6.257201	0.400473	0.163588
H	-6.028182	2.160832	0.133603
H	-5.109214	0.206369	-2.047404

Cartesian coordinates for conformer 3-8 after optimization at the PCM/B3LYP/6-31G* level of theory. Number of imaginary frequencies = 0. SCF Energy (PCM/mPW1PW91/6-31+G**) = -2647.61077689.

Atom	X	Y	Z
O	-0.011379	-1.585478	-3.63114
O	-1.092193	-0.06962	-2.332351
O	-3.348684	-0.33816	-0.485792
O	1.474165	1.639811	1.358731
O	-2.209112	-2.682448	-1.948296
H	-1.608067	-3.171499	-2.546348
O	3.188969	1.583032	-0.76069
O	4.706848	1.894839	2.034026
O	-2.703084	-0.277451	3.599567
O	-2.033948	-2.125603	2.440228
O	3.541305	-0.053002	2.063301
O	-0.209242	-4.315536	-1.779625
O	0.048226	0.54615	2.730484
C	-2.645427	-3.129315	0.386664
H	-2.338481	-3.978038	1.004321
H	-3.543721	-3.416158	-0.162517
C	-2.915991	-1.905052	1.288788
C	-2.352958	-0.702588	0.487024
H	-2.125865	0.153508	1.120492
C	-1.120484	-1.305339	-0.19372
H	-0.44117	-1.481362	0.644201
C	-0.313641	-0.455563	-1.191917
H	0.511371	-1.079728	-1.553685
C	0.244613	0.793408	-0.54059
C	1.367776	0.53664	0.443657
H	1.158427	-0.361555	1.022111
C	2.723226	0.327771	-0.237547
H	2.551778	-0.339077	-1.082445
C	3.809163	-0.299412	0.663781
H	4.766228	0.163299	0.407381
C	3.92036	-1.848505	0.508262
C	2.580759	-2.482385	0.846983
H	2.210829	-2.237445	1.844177
C	1.850284	-3.282385	0.06668
H	2.192491	-3.55817	-0.92859
C	0.519513	-3.897236	0.456262
H	0.113537	-3.393655	1.339711
C	-0.413751	-3.705922	-0.743574
C	-1.585529	-2.714088	-0.682543
C	-4.376588	-1.787338	1.709238
H	-4.649558	-2.652261	2.323263
H	-5.00881	-1.778365	0.818538
H	-4.549163	-0.877221	2.283083
C	-0.207834	2.021463	-0.793236
H	0.203791	2.884078	-0.280927
H	-1.015819	2.191213	-1.491976
C	4.40825	-2.178652	-0.914541

H	5.367756	-1.685481	-1.10796
H	3.709935	-1.868556	-1.697433
H	4.562314	-3.257358	-1.018206
C	4.974952	-2.366103	1.515321
H	4.664892	-2.186969	2.54787
H	5.938952	-1.866856	1.356208
H	5.120586	-3.443005	1.38123
C	0.670796	-5.403121	0.755543
H	1.044613	-5.928508	-0.127836
H	-0.292422	-5.84562	1.035882
H	1.374532	-5.548431	1.580691
C	-2.01184	-1.264046	3.494873
C	-0.987155	-1.726613	4.502305
H	-1.111378	-1.160874	5.426512
H	0.00791	-1.528864	4.092929
H	-1.081444	-2.799757	4.692464
C	0.695486	1.542542	2.459612
C	0.730726	2.768258	3.2896
C	1.668721	3.786566	3.067878
H	2.399448	3.682178	2.272526
C	1.669052	4.908732	3.89281
H	2.399354	5.696807	3.731635
C	0.732536	5.020862	4.923793
H	0.733477	5.901012	5.561622
C	-0.206545	4.008034	5.136876
H	-0.938275	4.102537	5.934366
C	-0.207558	2.877746	4.324357
H	-0.93225	2.079655	4.460347
C	4.050406	1.073124	2.629594
C	3.709987	1.111677	4.100239
H	3.789657	2.135866	4.464885
H	2.70548	0.72257	4.282464
H	4.421635	0.479325	4.644398
C	-0.829166	-0.699848	-3.504692
C	-3.715955	0.964112	-0.573045
O	-3.240304	1.85061	0.101305
C	-1.730787	-0.190477	-4.614422
C	-1.880572	1.337341	-4.598611
C	-3.089389	-0.912701	-4.509118
H	-1.239693	-0.497309	-5.543881
H	-0.904634	1.832528	-4.642347
H	-2.471563	1.664877	-5.461131
H	-2.385358	1.675279	-3.688892
H	-2.973734	-1.990546	-4.657837
H	-3.52817	-0.76744	-3.518917
H	-3.779267	-0.52932	-5.269245
C	3.016723	1.787491	-2.095442
O	2.585016	0.955788	-2.862301
C	3.425457	3.19226	-2.45642
H	4.38397	3.444985	-1.994589
H	3.479472	3.290847	-3.541142
H	2.675597	3.887627	-2.062752
C	-4.778315	1.158329	-1.648419
C	-5.396591	2.554982	-1.535123
C	-5.83434	0.04118	-1.631656
H	-5.949915	2.658358	-0.595202
H	-4.625446	3.329554	-1.550252
H	-6.090751	2.729199	-2.364198
H	-6.568994	0.207587	-2.427283
H	-5.377276	-0.940338	-1.779663
H	-6.370923	0.030267	-0.675157
H	-4.234848	1.089575	-2.60062

Cartesian coordinates for conformer 3-9 after optimization at the PCM/B3LYP/6-31G* level of theory. Number of imaginary frequencies = 0. SCF Energy (PCM/mPW1PW91/6-31+G**) = -2647.61063031.

Atom	X	Y	Z
O	-2.845891	1.617843	-2.693801
O	-1.383663	1.212955	-1.002466
O	-3.84555	-0.62095	-0.649998
O	1.264389	1.191902	-0.223036
O	-2.183241	-2.695186	-2.039887
H	-1.567302	-3.304683	-2.496824
O	3.148005	0.975947	-2.064578
O	5.190206	1.912641	-0.041917
O	-2.409086	-3.832322	3.24532
O	-2.139123	-1.78129	2.300642
O	3.73787	0.406076	0.84411
O	-0.077216	-4.087452	-1.629653
O	0.642849	0.229299	1.741807
C	-2.751818	-3.132052	0.298983
H	-2.454843	-4.017691	0.857624
H	-3.624157	-3.395644	-0.303238
C	-3.125041	-1.967743	1.231939
C	-2.831974	-0.731844	0.362168
H	-2.788311	0.183404	0.949045
C	-1.504453	-1.08971	-0.314939
H	-0.760857	-1.010173	0.477329
C	-1.139582	-0.150427	-1.469463
H	-1.807712	-0.346154	-2.305094
C	0.30563	-0.224388	-1.940212
C	1.386538	-0.09213	-0.880051
H	1.213392	-0.855502	-0.130242
C	2.833599	-0.241121	-1.359621
H	2.884914	-1.081703	-2.053345
C	3.910239	-0.493044	-0.277863
H	4.871697	-0.262029	-0.740317
C	4.000077	-1.95194	0.280098
C	2.637603	-2.377477	0.787414
H	2.24197	-1.756572	1.59226
C	1.864003	-3.329534	0.263492
H	2.213246	-3.951951	-0.556405
C	0.422422	-3.57186	0.64941
H	0.097927	-2.818307	1.371788
C	-0.404309	-3.451381	-0.63772
C	-1.68396	-2.595054	-0.721888
C	-4.525454	-2.024522	1.82215
H	-4.640552	-2.922907	2.432157
H	-5.265813	-2.044103	1.018172
H	-4.70747	-1.139021	2.438946
C	0.55675	-0.442056	-3.234285
H	1.560289	-0.495233	-3.643345
H	-0.257747	-0.578786	-3.939591
C	4.54533	-2.863903	-0.835308
H	3.897921	-2.894933	-1.715551
H	4.65121	-3.888025	-0.462846
H	5.529369	-2.516533	-1.165385
C	5.006927	-1.963315	1.452575
H	4.644257	-1.373801	2.298615
H	5.975214	-1.554476	1.138615
H	5.165007	-2.992179	1.793335
C	0.239738	-4.983012	1.257119
H	0.479629	-5.746274	0.51059
H	-0.77894	-5.14382	1.620312
H	0.921191	-5.098631	2.105709
C	-1.87659	-2.742667	3.21096
C	-0.825364	-2.249718	4.180506
H	-0.312112	-3.105429	4.622718
H	-1.319994	-1.687882	4.98193

H	-0.121538	-1.575737	3.684213
C	0.94584	1.211353	1.083358
C	1.007533	2.58922	1.63268
C	0.46711	2.816506	2.903944
H	0.011258	1.98549	3.43281
C	0.513929	4.091387	3.460539
H	0.087248	4.269924	4.443538
C	1.108392	5.139162	2.753294
H	1.145992	6.134179	3.189235
C	1.657025	4.911632	1.488825
H	2.125173	5.726152	0.943011
C	1.607829	3.639128	0.924931
H	2.043904	3.445284	-0.04914
C	4.492464	1.534159	0.873704
C	4.393231	2.212033	2.219587
H	5.272905	1.926303	2.808833
H	3.493916	1.913772	2.759016
H	4.408413	3.294857	2.084301
C	-2.210401	2.00281	-1.733543
C	-4.730427	0.404469	-0.568618
O	-4.764648	1.192104	0.354128
C	-2.191393	3.436421	-1.229285
C	-0.876491	4.088228	-1.701921
C	-2.371828	3.541406	0.291986
H	-3.033002	3.925365	-1.73128
H	-0.018592	3.545588	-1.295842
H	-0.802143	4.083872	-2.795143
H	-0.824784	5.126706	-1.356229
H	-3.304217	3.068631	0.61131
H	-1.542523	3.062085	0.817291
H	-2.392781	4.596802	0.587391
C	4.174786	0.918664	-2.948657
O	4.724021	-0.115564	-3.264746
C	4.532096	2.300756	-3.422744
H	3.632498	2.864695	-3.685786
H	5.023155	2.816569	-2.589937
H	5.210681	2.236892	-4.274147
C	-5.668789	0.387757	-1.759702
C	-6.759389	-0.672985	-1.508428
C	-6.251256	1.781974	-1.999381
H	-7.349711	-0.420427	-0.619198
H	-6.319845	-1.665701	-1.365121
H	-7.440188	-0.72112	-2.364845
H	-6.776441	2.142199	-1.109199
H	-6.956021	1.757625	-2.837713
H	-5.452823	2.490239	-2.240363
H	-5.070249	0.082012	-2.623522

Cartesian coordinates for conformer 3-10 after optimization at the PCM/B3LYP/6-31G* level of theory. Number of imaginary frequencies = 0. SCF Energy (PCM/mPW1PW91/6-31+G**) = -2647.61139406.

Atom	X	Y	Z
O	-3.734587	-0.653573	-0.509332
O	1.381526	1.103528	-0.186881
O	3.255756	0.862271	-2.051001
O	5.229881	1.760312	0.024545
O	-1.241792	1.159516	-0.863186
H	0.722112	0.094673	1.736613
O	3.790739	0.218123	0.870136
O	-2.225861	-2.670384	-2.031042
O	-1.626149	-3.248805	-2.546359
O	-0.121915	-4.109864	-1.772602
O	-2.557943	1.761403	-2.611205
O	4.836034	-0.245772	-3.230304
O	-4.506244	1.282221	0.385456

C	-2.73112	-3.168121	0.309227
H	-3.637019	-3.373728	-0.26395
H	-2.438996	-4.083356	0.829308
C	-2.989133	-2.034135	1.314544
C	-2.688435	-0.777969	0.4751
H	-2.61686	0.132186	1.061425
C	-1.407615	-1.162425	-0.266661
H	-0.625099	-1.154814	0.490617
C	-1.052197	-0.187487	-1.393323
H	-1.747793	-0.332883	-2.216917
C	0.379058	-0.279972	-1.902435
C	1.480236	-0.176149	-0.859925
H	1.305754	-0.943275	-0.114224
C	2.915239	-0.353216	-1.357074
H	2.93881	-1.186345	-2.061036
C	3.977057	-0.649521	-0.272549
H	4.950116	-0.41882	-0.710262
C	4.031837	-2.126238	0.240374
C	2.657284	-2.556973	0.716074
H	2.269281	-1.980924	1.557223
C	1.873736	-3.47066	0.139869
H	2.21709	-4.042295	-0.71905
C	0.437175	-3.744875	0.524364
H	0.109094	-3.06579	1.315047
C	-0.414794	-3.533633	-0.733953
C	-1.673136	-2.641209	-0.731087
C	4.582921	-3.011194	-0.893833
H	5.578481	-2.669449	-1.194566
H	3.953776	-3.004985	-1.787948
H	4.665081	-4.046973	-0.549299
C	5.018365	-2.188903	1.428501
H	4.651799	-1.6155	2.283683
H	5.998201	-1.787802	1.141743
C	5.153774	-3.229122	1.743287
H	-4.355035	-2.058969	1.982947
H	-5.139615	-2.005168	1.223829
H	-4.459113	-1.208045	2.659416
C	-4.478679	-2.988327	2.548727
H	-1.920591	2.059116	-1.621849
H	-4.529342	0.44726	-0.492025
H	-1.743889	3.465011	-1.069269
C	-0.657653	3.603981	-0.979393
H	-2.348008	3.599749	0.341387
H	-3.434475	3.484334	0.311489
H	-2.104226	4.587598	0.74711
C	-1.949661	2.844312	1.020829
C	-2.318796	4.50055	-2.038154
H	-1.883689	4.403014	-3.037555
H	-2.118445	5.511233	-1.665768
H	-3.40279	4.378934	-2.134406
C	0.975983	1.103142	1.094436
C	0.866871	2.474201	1.645478
C	1.410536	3.581403	0.979904
H	1.92168	3.437069	0.033592
C	1.29813	4.848734	1.546682
H	1.722877	5.709161	1.036738
C	0.637525	5.012538	2.767472
H	0.547465	6.003683	3.204646
C	0.085729	3.909389	3.423192
H	-0.442299	4.042024	4.363254
C	0.200867	2.638086	2.866879
H	-0.246531	1.771735	3.345227
C	0.600479	-0.479418	-3.204437
C	1.595522	-0.540936	-3.633269
H	-0.229726	-0.589844	-3.895975
H	4.294069	0.794244	-2.920589

H	4.673046	2.172856	-3.388683
C	3.783933	2.743561	-3.671918
C	5.369034	2.102091	-4.225345
O	5.149712	2.686951	-2.546372
C	4.4999	1.373703	0.911184
C	4.293572	2.083391	2.228546
C	4.181373	3.154571	2.048345
H	5.188294	1.933946	2.84351
H	3.422818	1.703028	2.763627
H	0.270246	-5.203464	1.009602
H	0.549665	-5.90084	0.21379
H	-0.760853	-5.416096	1.310271
H	0.920964	-5.376988	1.872425
H	-1.933583	-2.214479	2.322041
C	-1.663356	-1.2667	3.257325
O	-2.250887	-0.212823	3.367936
C	-0.499768	-1.696476	4.117529
H	-0.599128	-1.243976	5.106438
H	-0.434545	-2.784023	4.195908
H	0.412322	-1.314194	3.648074
C	-5.446442	0.443264	-1.701899
C	-5.992197	1.847542	-1.964985
C	-6.563839	-0.594758	-1.476477
H	-6.532311	2.225747	-1.091175
H	-5.172785	2.536968	-2.18925
H	-6.675606	1.832726	-2.821237
H	-7.22132	-0.635667	-2.351379
H	-6.146566	-1.593906	-1.314403
H	-7.174477	-0.325116	-0.60602
H	-4.834718	0.119865	-2.551056

Cartesian coordinates for conformer 3-11 after optimization at the PCM/B3LYP/6-31G* level of theory. Number of imaginary frequencies = 0. SCF Energy (PCM/mPW1PW91/6-31+G**) = -2647.60941885.

Atom	X	Y	Z
O	-3.820052	-0.760068	-0.422479
O	1.282885	1.094172	-0.130141
O	3.148199	0.980164	-1.998419
O	5.100513	1.838569	0.110795
O	-1.348966	1.121471	-0.800376
H	0.664756	0.007427	1.764391
O	3.721278	0.208523	0.888523
O	-2.279448	-2.700801	-2.017265
O	-1.672452	-3.248533	-2.557429
O	-0.139135	-4.088532	-1.827099
O	-2.146117	1.875409	-2.782192
O	4.767029	-0.020106	-3.22116
O	-4.36559	1.342148	0.223989
C	-2.725623	-3.244597	0.3237
H	-3.636727	-3.465667	-0.234882
H	-2.400688	-4.15864	0.825777
C	-2.993207	-2.131467	1.349811
C	-2.736372	-0.855566	0.524341
H	-2.675093	0.051852	1.116766
C	-1.45754	-1.20249	-0.245152
H	-0.666521	-1.194352	0.503415
C	-1.127271	-0.207352	-1.360413
H	-1.820887	-0.356874	-2.184882
C	0.302507	-0.254828	-1.883365
C	1.409683	-0.16049	-0.846898
H	1.26419	-0.955955	-0.125272
C	2.845894	-0.275766	-1.361401
H	2.88738	-1.072769	-2.105381
C	3.922567	-0.595283	-0.297914
H	4.886451	-0.318081	-0.728773

C	4.01871	-2.091456	0.146923
C	2.663967	-2.560069	0.642604
H	2.287151	-2.012413	1.50747
C	1.879844	-3.468198	0.05914
H	2.211215	-4.011997	-0.822125
C	0.454514	-3.767898	0.466688
H	0.133952	-3.108982	1.277506
C	-0.424685	-3.542919	-0.770631
C	-1.701293	-2.67812	-0.727137
C	4.552362	-2.918611	-1.038161
H	5.525002	-2.535756	-1.362856
H	3.889191	-2.900164	-1.907152
H	4.677673	-3.963588	-0.737062
C	5.039192	-2.185234	1.30424
H	4.689329	-1.650135	2.190787
H	6.005284	-1.759896	1.006059
C	5.196949	-3.234529	1.575467
H	-4.349798	-2.20248	2.034113
H	-5.143247	-2.142863	1.284849
H	-4.462827	-1.374636	2.737589
C	-4.447622	-3.149775	2.57451
H	-1.738311	2.098298	-1.660969
H	-4.514344	0.402378	-0.525301
H	-1.537481	3.476519	-1.053391
C	-0.461496	3.524456	-0.834684
H	-2.289904	3.648218	0.277582
H	-3.370881	3.622397	0.117862
H	-2.017429	4.609419	0.726593
C	-2.040628	2.85503	0.983803
C	-1.902256	4.564983	-2.065251
H	-1.344856	4.448021	-2.999343
H	-1.683825	5.55333	-1.646208
H	-2.969097	4.522579	-2.308955
C	0.896671	1.041597	1.156008
C	0.777961	2.390444	1.756429
C	1.369169	3.513853	1.162472
H	1.924608	3.398352	0.237052
C	1.248433	4.759406	1.77342
H	1.710149	5.631809	1.319516
C	0.530405	4.885718	2.966037
H	0.432022	5.86036	3.437124
C	-0.067221	3.766472	3.550399
H	-0.639	3.870707	4.468135
C	0.058138	2.515603	2.951525
H	-0.422044	1.638204	3.375801
C	0.516794	-0.376193	-3.195913
C	1.509629	-0.387816	-3.634392
H	-0.31687	-0.461012	-3.886139
H	4.189706	0.986123	-2.867249
H	4.522448	2.396408	-3.271531
C	3.61502	2.948334	-3.533429
C	5.222928	2.386905	-4.107327
O	4.978869	2.888515	-2.40516
C	4.400306	1.379038	0.986345
C	4.205858	1.997479	2.350638
C	4.12352	3.081369	2.249717
H	5.092227	1.776528	2.956886
H	3.323253	1.600497	2.853647
H	0.316003	-5.238559	0.924232
H	0.590081	-5.915231	0.108757
H	-0.705956	-5.472685	1.239526
H	0.985676	-5.420747	1.770505
H	-1.921764	-2.303785	2.340664
C	-1.670977	-1.369867	3.294204
O	-2.282327	-0.331282	3.425178
C	-0.499559	-1.79172	4.146848

H	-0.605139	-1.354208	5.1418
H	-0.417134	-2.878914	4.210476
H	0.405684	-1.388881	3.681421
C	-5.500498	0.336096	-1.684282
C	-4.850999	-0.241252	-2.956689
C	-6.114204	1.718418	-1.926813
H	-4.052196	0.421254	-3.305215
H	-4.419557	-1.228646	-2.775585
H	-5.604063	-0.321204	-3.748994
H	-6.881598	1.658242	-2.70595
H	-6.564829	2.122992	-1.016347
H	-5.340377	2.419166	-2.259855
H	-6.287917	-0.360411	-1.355628

Cartesian coordinates for conformer 3-12 after optimization at the PCM/B3LYP/6-31G* level of theory. Number of imaginary frequencies = 0. SCF Energy (PCM/mPW1PW91/6-31+G**) = -2647.6131195.

Atom	X	Y	Z
O	-3.381019	-0.323861	-0.456751
O	1.43105	1.400452	1.425479
O	3.24639	1.738145	-0.408519
O	4.931493	1.696756	2.034794
O	-1.196251	-0.088688	-2.290465
H	0.102835	0.031418	2.632362
O	3.62132	-0.15892	2.046361
O	-2.215184	-2.683223	-1.966552
O	-1.657356	-3.271129	-2.517461
O	-0.283585	-4.369858	-1.744356
O	-0.100366	-1.454191	-3.734866
O	4.887812	1.084131	-1.821048
O	-2.968006	1.877402	-0.090616
C	-2.725968	-3.134291	0.360351
H	-3.620297	-3.380514	-0.214578
H	-2.459566	-4.005356	0.966399
C	-2.979466	-1.921992	1.281187
C	-2.385328	-0.717535	0.50577
H	-2.145284	0.121794	1.154898
C	-1.153933	-1.326469	-0.178579
H	-0.474709	-1.505352	0.6583
C	-0.351493	-0.499554	-1.205695
H	0.418033	-1.166113	-1.61629
C	0.330889	0.719598	-0.616378
C	1.435132	0.412261	0.375087
H	1.256914	-0.557614	0.828937
C	2.843218	0.37041	-0.226578
H	2.78942	-0.10989	-1.206228
C	3.864959	-0.404705	0.639338
H	4.861501	-0.033641	0.396687
C	3.86901	-1.952157	0.446095
C	2.523423	-2.545128	0.831169
H	2.167671	-2.257168	1.821768
C	1.780478	-3.371371	0.090402
H	2.116322	-3.689678	-0.894069
C	0.436773	-3.947296	0.494516
H	0.0411	-3.41744	1.367039
C	-0.481473	-3.745739	-0.71374
C	-1.632849	-2.72853	-0.678725
C	4.279485	-2.2756	-1.002932
H	5.191059	-1.734344	-1.272328
H	3.509779	-2.007139	-1.732421
H	4.471886	-3.348083	-1.106538
C	4.940401	-2.538237	1.39919
H	4.695256	-2.342662	2.446287
H	5.924141	-2.100873	1.188747
C	5.009147	-3.621899	1.259072

H	-4.44161	-1.780203	1.690402
H	-5.067903	-1.742948	0.796833
H	-4.60094	-0.875743	2.277109
C	-4.736297	-2.6493	2.28833
H	-1.022308	-0.708079	-3.485723
H	-3.561178	1.001701	-0.680115
H	-2.141997	-0.336714	-4.441671
C	-3.061051	-0.641943	-3.923208
H	-2.014936	-1.113685	-5.753102
H	-1.083808	-0.85545	-6.268046
H	-2.855611	-0.877678	-6.414755
C	-2.004198	-2.192592	-5.573283
C	-2.185586	1.185824	-4.668457
H	-2.278227	1.729445	-3.724855
H	-3.038654	1.448956	-5.303645
H	-1.272971	1.526512	-5.171514
C	0.672272	1.102385	2.496668
C	0.622121	2.210705	3.478392
C	1.51413	3.29091	3.410727
H	2.254609	3.327129	2.61795
C	1.45127	4.294905	4.373313
H	2.146544	5.128754	4.33068
C	0.493782	4.231012	5.389124
H	0.444897	5.019108	6.136311
C	-0.404813	3.163055	5.443955
H	-1.157999	3.124592	6.226027
C	-0.342215	2.147661	4.493135
H	-1.046786	1.320725	4.503219
C	0.01297	1.974313	-0.932471
C	0.534412	2.806996	-0.473347
H	-0.789879	2.199849	-1.621645
H	4.326762	1.957872	-1.194595
H	4.731923	3.406131	-1.127271
C	3.863848	4.057504	-1.263941
C	5.491006	3.613301	-1.882388
O	5.137334	3.592993	-0.126498
C	4.225928	0.908855	2.624346
C	3.932518	0.935182	4.105551
C	4.065022	1.948231	4.486292
H	4.637885	0.267435	4.614831
H	2.920352	0.58377	4.315356
H	0.545661	-5.451401	0.817434
H	0.908751	-6.001163	-0.055475
H	-0.429928	-5.863476	1.101707
H	1.242401	-5.60245	1.647405
H	-2.12852	-2.195559	2.444076
C	-2.108739	-1.37128	3.522911
O	-2.731886	-0.33712	3.61818
C	-1.193413	-1.939154	4.579948
H	-1.3335	-1.388757	5.511266
H	-1.38855	-3.00448	4.733104
H	-0.161593	-1.816936	4.239999
C	-4.60751	1.22189	-1.762894
C	-4.730617	2.71026	-2.095722
C	-5.956037	0.603564	-1.350041
H	-5.08462	3.273192	-1.225835
H	-3.765714	3.134266	-2.388607
H	-5.439672	2.856272	-2.918052
H	-6.697698	0.759544	-2.141046
H	-5.859914	-0.471166	-1.173672
H	-6.335219	1.073242	-0.434602
H	-4.244423	0.67659	-2.642775

Cartesian coordinates for conformer 3-13 after optimization at the PCM/B3LYP/6-31G* level of theory. Number of imaginary frequencies = 0. SCF Energy (PCM/mPW1PW91/6-31+G**) = -2647.6131009.

Atom	X	Y	Z
O	-3.756778	-0.607261	-0.528693
O	1.475228	1.165743	-0.262115
O	3.247825	0.727633	-2.201333
O	5.346551	1.714652	-0.241792
O	-1.196479	1.208207	-0.736539
H	0.815174	0.307927	1.738038
O	3.889987	0.278878	0.749464
O	-2.131038	-2.598317	-2.05327
O	-1.54481	-3.214069	-2.539985
O	-0.069453	-4.08633	-1.717876
O	-2.726126	1.868125	-2.280041
O	4.713781	-0.534453	-3.373695
O	-4.669476	1.150946	0.574057
C	-2.70814	-3.178009	0.255805
H	-3.586381	-3.385584	-0.359287
H	-2.42587	-4.10395	0.753365
C	-3.056122	-2.071489	1.265673
C	-2.745469	-0.78775	0.475468
H	-2.690876	0.090279	1.116227
C	-1.42635	-1.121535	-0.230341
H	-0.66984	-1.091398	0.554964
C	-1.060145	-0.117916	-1.328797
H	-1.775674	-0.204833	-2.14322
C	0.355026	-0.261535	-1.868654
C	1.490048	-0.149898	-0.863319
H	1.304067	-0.865182	-0.069767
C	2.899349	-0.417003	-1.397726
H	2.863421	-1.30516	-2.030689
C	4.007082	-0.659872	-0.344929
H	4.960576	-0.470031	-0.841048
C	4.068916	-2.105117	0.248917
C	2.692999	-2.505091	0.741991
H	2.308895	-1.893684	1.560477
C	1.90267	-3.428727	0.192971
H	2.243964	-4.035317	-0.641936
C	0.460966	-3.668688	0.576529
H	0.148342	-2.94422	1.333675
C	-0.37154	-3.481906	-0.698317
C	-1.631139	-2.59396	-0.730747
C	4.617455	-3.047376	-0.840382
H	5.629336	-2.744876	-1.12793
H	4.011759	-3.052948	-1.750621
H	4.66193	-4.072045	-0.457426
C	5.057792	-2.109148	1.43573
H	4.688854	-1.504417	2.26797
H	6.033515	-1.712256	1.130312
C	5.202757	-3.134729	1.792154
H	-4.45575	-2.139973	1.857259
H	-5.198276	-2.090247	1.056768
H	-4.617759	-1.294402	2.532716
C	-4.588258	-3.074601	2.40573
H	-2.014831	2.113934	-1.327588
H	-4.636925	0.417121	-0.391182
H	-1.893686	3.463546	-0.634675
C	-0.829734	3.729326	-0.704416
H	-2.250087	3.362051	0.860968
H	-3.304849	3.103572	0.988658
H	-1.64782	2.600405	1.360661
C	-2.055682	4.32297	1.349698
C	-2.735167	4.520325	-1.35242
H	-2.4896	4.575053	-2.417218
H	-2.563532	5.503419	-0.900792

H	-3.801401	4.286184	-1.26684
C	1.129877	1.254751	1.035105
C	1.187079	2.658989	1.517877
C	1.550991	3.717368	0.674397
H	1.801308	3.51186	-0.360814
C	1.597751	5.016304	1.176719
H	1.878405	5.83768	0.523206
C	1.287566	5.260834	2.516681
H	1.326738	6.275084	2.905162
C	0.928204	4.205506	3.357947
H	0.686195	4.396593	4.399671
C	0.876283	2.907117	2.859912
H	0.594321	2.070773	3.491718
C	0.531268	-0.549485	-3.161072
C	1.509897	-0.680423	-3.610791
H	-0.322522	-0.676581	-3.819998
H	4.233385	0.549001	-3.114949
H	4.644779	1.870901	-3.703166
C	5.301212	1.707531	-4.55858
C	5.175172	2.428907	-2.923202
O	3.766607	2.454044	-3.995261
C	4.641826	1.407563	0.694728
C	4.51394	2.207083	1.970104
C	3.763496	1.788854	2.641424
H	4.250363	3.238501	1.722289
H	5.489866	2.219227	2.467635
H	0.268378	-5.102789	1.124033
H	0.497258	-5.835125	0.343717
H	-0.74995	-5.270628	1.485144
H	0.953152	-5.261821	1.962943
H	-2.061781	-1.977699	2.339617
C	-1.832985	-3.000185	3.190742
O	-2.395212	-4.074703	3.154958
C	-0.776514	-2.598769	4.195188
H	-0.340707	-3.494139	4.640835
H	-0.008895	-1.976905	3.725843
H	-1.246219	-2.001982	4.985793
C	-5.571259	0.464002	-1.584638
C	-6.213309	1.846111	-1.70983
C	-6.613587	-0.663113	-1.436535
H	-6.758433	2.106324	-0.797027
H	-5.445443	2.605988	-1.882012
H	-6.912294	1.863099	-2.553266
H	-7.288182	-0.666814	-2.299253
H	-6.128881	-1.643113	-1.377002
H	-7.218935	-0.516556	-0.533586
H	-4.955827	0.259414	-2.466808

Cartesian coordinates for conformer 3-14 after optimization at the PCM/B3LYP/6-31G* level of theory. Number of imaginary frequencies = 0. SCF Energy (PCM/mPW1PW91/6-31+G**) = -2647.611114.

Atom	X	Y	Z
O	-3.754415	-0.654215	-0.563692
O	1.458016	1.102292	-0.179425
O	3.2599	0.800259	-2.10906
O	5.356517	1.700689	-0.113504
O	-1.194615	1.194893	-0.745971
H	0.837922	0.138448	1.784473
O	3.893347	0.22224	0.802205
O	-2.135491	-2.609312	-2.060754
O	-1.545185	-3.211628	-2.559324
O	-0.05619	-4.075148	-1.756402
O	-2.508186	1.908215	-2.451996
O	4.763167	-0.364198	-3.334372
O	-4.602584	1.210371	0.395258

C	-2.684968	-3.18885	0.254498
H	-3.563331	-3.418037	-0.352517
H	-2.387011	-4.102689	0.763856
C	-3.043404	-2.07345	1.251964
C	-2.743708	-0.796109	0.447341
H	-2.704542	0.094427	1.072214
C	-1.416164	-1.130019	-0.245361
H	-0.668411	-1.102759	0.547833
C	-1.03916	-0.129438	-1.340019
H	-1.743465	-0.221811	-2.163182
C	0.382167	-0.257312	-1.867809
C	1.504319	-0.18139	-0.845181
H	1.322283	-0.939235	-0.091855
C	2.924413	-0.394481	-1.376145
H	2.913498	-1.245682	-2.058869
C	4.025293	-0.674477	-0.325529
H	4.981118	-0.460772	-0.807505
C	4.092186	-2.139786	0.216925
C	2.721857	-2.550939	0.715701
H	2.345644	-1.953988	1.5478
C	1.922892	-3.460779	0.156474
H	2.253353	-4.055096	-0.691578
C	0.47993	-3.69095	0.543275
H	0.176673	-2.96924	1.307053
C	-0.356981	-3.486993	-0.727022
C	-1.6204	-2.603453	-0.743461
C	4.623775	-3.0464	-0.909682
H	5.624252	-2.724748	-1.216254
H	3.992993	-3.037785	-1.802811
H	4.689887	-4.079843	-0.554662
C	5.097033	-2.182479	1.389822
H	4.741746	-1.600042	2.243808
H	6.070693	-1.78166	1.082762
C	5.241133	-3.218509	1.715152
H	-4.442467	-2.151721	1.84267
H	-5.183585	-2.109612	1.04016
H	-4.611907	-1.306958	2.517912
C	-4.567491	-3.086356	2.393428
H	-1.900655	2.132086	-1.424812
H	-4.602728	0.401109	-0.509165
H	-1.794771	3.486576	-0.739154
C	-0.723139	3.731386	-0.760097
H	-2.227149	3.419757	0.737324
H	-3.290007	3.177517	0.816617
H	-2.043792	4.388048	1.216022
C	-1.661658	2.660876	1.281599
C	-2.578349	4.545247	-1.517866
H	-2.266914	4.585793	-2.565532
H	-2.42498	5.53119	-1.065796
H	-3.650157	4.321012	-1.496501
C	1.120359	1.124277	1.121987
C	1.134551	2.507652	1.662056
C	1.516627	3.603407	0.876462
H	1.819143	3.442128	-0.152451
C	1.513008	4.882782	1.427859
H	1.808103	5.733772	0.82014
C	1.131559	5.071378	2.758868
H	1.128888	6.071119	3.185343
C	0.754396	3.978208	3.542712
H	0.456623	4.125867	4.577102
C	0.755923	2.698147	2.995857
H	0.462003	1.833511	3.582492
C	0.574202	-0.483782	-3.170095
C	1.558536	-0.588657	-3.614639
H	-0.27155	-0.579099	-3.844587
H	4.259851	0.693457	-3.019382

H	4.652459	2.055189	-3.523779
C	5.166492	2.57365	-2.706508
C	3.766518	2.639642	-3.788856
O	5.318997	1.955022	-4.381131
C	4.639661	1.355207	0.800512
C	4.488147	2.105473	2.103244
C	4.232747	3.146629	1.89055
H	5.453429	2.094749	2.62098
H	3.723002	1.664125	2.742369
H	0.275787	-5.127646	1.080517
H	0.492915	-5.856081	0.293292
H	-0.741575	-5.288351	1.447444
H	0.964303	-5.299506	1.913773
H	-2.051154	-1.955039	2.323946
C	-1.793092	-2.968962	3.176914
O	-2.339325	-4.05218	3.155357
C	-0.727549	-2.544377	4.162645
H	-0.23254	-3.430529	4.563681
H	-0.009152	-1.866171	3.693446
H	-1.205049	-2.006072	4.990201
C	-5.565691	0.404876	-1.690032
C	-5.015288	-0.312299	-2.931831
C	-5.99097	1.847806	-1.997422
H	-4.138301	0.220507	-3.310678
H	-4.71594	-1.339938	-2.71365
H	-5.780989	-0.324546	-3.715585
H	-6.775249	1.854503	-2.761948
H	-6.366057	2.348257	-1.101022
H	-5.132477	2.412311	-2.377018
H	-6.446338	-0.149115	-1.325071

Cartesian coordinates for conformer 3-15 after optimization at the PCM/B3LYP/6-31G* level of theory. Number of imaginary frequencies = 0. SCF Energy (PCM/mPW1PW91/6-31+G**) = -2647.61033048.

Atom	X	Y	Z
O	-2.913627	0.153496	-0.449732
O	1.329418	1.537957	1.362901
O	3.228154	1.518678	-0.304968
O	4.463524	1.393239	2.507838
O	-1.034167	-0.205749	-2.446867
H	-0.260436	0.651113	2.717284
O	3.192688	-0.469499	2.239173
O	-2.55847	-2.551097	-1.921104
O	-2.248074	-3.238525	-2.548818
O	-0.808781	-4.435666	-2.143737
O	-0.101087	-1.846405	-3.701091
O	4.826696	0.674211	-1.665053
O	-3.214088	1.760359	1.123105
C	-2.644817	-3.115667	0.436108
H	-3.510706	-3.666082	0.061297
H	-2.121581	-3.745146	1.154586
C	-3.065463	-1.77747	1.107903
C	-2.173466	-0.667471	0.470081
H	-1.765947	-0.024793	1.247228
C	-1.084309	-1.415533	-0.29927
H	-0.386864	-1.728058	0.476202
C	-0.259388	-0.590315	-1.304509
H	0.563992	-1.225716	-1.651618
C	0.303381	0.661464	-0.657847
C	1.280601	0.407408	0.475512
H	0.992156	-0.466471	1.058022
C	2.708759	0.197648	-0.066888
H	2.644729	-0.322194	-1.024055
C	3.627438	-0.62171	0.866541
H	4.642569	-0.229097	0.784108

C	3.662489	-2.147677	0.554997
C	2.27551	-2.762402	0.646255
H	1.799592	-2.663827	1.623179
C	1.636039	-3.459137	-0.300947
H	2.059819	-3.589148	-1.295649
C	0.344969	-4.216903	-0.043758
H	-0.012595	-3.939606	0.950469
C	-0.719915	-3.847815	-1.07835
C	-1.743822	-2.734231	-0.777749
C	4.334016	-2.373955	-0.811873
H	5.321444	-1.905225	-0.837942
H	3.762663	-1.959686	-1.645711
H	4.454169	-3.447868	-0.986177
C	4.53809	-2.828399	1.637149
H	4.099555	-2.723848	2.632814
H	5.540978	-2.383989	1.65389
C	4.640077	-3.895911	1.41587
H	-4.554425	-1.503456	0.952032
H	-4.815253	-1.502992	-0.108846
H	-4.811672	-0.535742	1.390963
C	-5.122935	-2.287336	1.46171
H	-0.93321	-0.97629	-3.556007
H	-3.304591	1.377985	-0.02492
H	-2.019401	-0.615067	-4.556103
C	-2.952606	-0.89269	-4.045996
H	-1.868616	-1.441429	-5.834913
H	-0.934175	-1.192706	-6.34961
H	-2.70252	-1.240363	-6.516285
C	-1.845514	-2.512092	-5.613495
C	-2.059495	0.897236	-4.837225
H	-2.217987	1.468145	-3.918798
H	-2.8756	1.127404	-5.530984
H	-1.122435	1.234286	-5.296796
C	0.493687	1.561056	2.421142
C	0.627506	2.825147	3.185833
C	1.723109	3.682098	3.006198
H	2.500228	3.418422	2.296621
C	1.81366	4.846567	3.765117
H	2.666538	5.507815	3.638587
C	0.812499	5.163593	4.686685
H	0.884943	6.076612	5.272426
C	-0.281924	4.311947	4.858182
H	-1.062859	4.563672	5.570375
C	-0.372775	3.139729	4.113407
H	-1.216627	2.465676	4.222071
C	0.021869	1.897789	-1.070729
C	0.455079	2.760224	-0.576536
H	-0.640904	2.068757	-1.909732
H	4.313574	1.619124	-1.104285
H	4.787465	3.048769	-1.156868
C	5.150478	3.332395	-0.162704
C	3.95791	3.71616	-1.409639
O	5.590522	3.143715	-1.888696
C	3.679051	0.582118	2.946672
C	3.119337	0.562611	4.348649
C	3.253798	1.542407	4.807474
H	3.65744	-0.190387	4.93627
H	2.062436	0.284959	4.338558
H	0.611689	-5.733813	-0.09427
H	0.969949	-6.028382	-1.08471
H	-0.304203	-6.298344	0.113232
H	1.366073	-6.001473	0.651763
H	-2.873615	-1.773387	2.562799
C	-1.676244	-1.989466	3.139404
O	-0.683106	-2.400984	2.567761
C	-1.711431	-1.613028	4.597896

H	-1.466356	-0.546203	4.651751
H	-2.700732	-1.770829	5.032769
H	-0.95019	-2.177216	5.140011
C	-3.833496	2.200131	-1.194215
C	-4.472141	3.498033	-0.695734
C	-4.774565	1.380522	-2.093635
H	-5.367431	3.284869	-0.101139
H	-3.78082	4.059772	-0.061798
H	-4.76483	4.125818	-1.544271
H	-5.09096	1.983667	-2.951879
H	-4.279569	0.478543	-2.460882
H	-5.673902	1.079854	-1.543068
H	-2.939114	2.444468	-1.786512

Cartesian coordinates for conformer 3-16 after optimization at the PCM/B3LYP/6-31G* level of theory. Number of imaginary frequencies = 0. SCF Energy (PCM/mPW1PW91/6-31+G**) = -2647.61066125.

Atom	X	Y	Z
O	-3.76424	-0.618319	-0.555074
O	1.38593	1.066152	-0.152519
O	3.244166	0.856311	-2.025111
O	5.312204	1.690613	0.008519
O	-1.27168	1.173279	-0.909942
H	0.767363	0.063873	1.793049
O	3.846262	0.17971	0.864471
O	-2.198597	-2.678206	-2.030401
O	-1.604713	-3.280564	-2.524579
O	-0.11027	-4.130102	-1.717443
O	-2.743892	1.642337	-2.5761
O	4.779175	-0.241811	-3.271694
O	-4.624365	1.202325	0.486071
C	-2.72011	-3.17086	0.308038
H	-3.611956	-3.39927	-0.279606
H	-2.431348	-4.076968	0.837046
C	-3.044434	-2.023298	1.27995
C	-2.737125	-0.772235	0.437263
H	-2.66282	0.127911	1.045168
C	-1.431614	-1.144555	-0.274072
H	-0.671484	-1.109099	0.506125
C	-1.061795	-0.184073	-1.409131
H	-1.743487	-0.347025	-2.24078
C	0.375811	-0.277845	-1.898235
C	1.471086	-0.200931	-0.847509
H	1.289413	-0.981607	-0.1178
C	2.907427	-0.37167	-1.35012
H	2.928182	-1.192982	-2.067902
C	3.988503	-0.683462	-0.289248
H	4.950915	-0.45894	-0.752772
C	4.04778	-2.162573	0.216151
C	2.680814	-2.57284	0.724469
H	2.309797	-1.97207	1.555955
C	1.87865	-3.487087	0.176667
H	2.202841	-4.087548	-0.669303
C	0.437032	-3.709202	0.572711
H	0.13917	-2.974398	1.325825
C	-0.405143	-3.523699	-0.696773
C	-1.663094	-2.632394	-0.723133
C	4.556462	-3.04877	-0.936395
H	5.543168	-2.712456	-1.270434
H	3.896236	-3.039719	-1.808038
H	4.64497	-4.08513	-0.595814
C	5.067088	-2.239536	1.375138
H	4.730068	-1.668267	2.244008
H	6.042378	-1.84663	1.06295
C	5.201008	-3.282708	1.680571

H	-4.434063	-2.061828	1.896357
H	-5.190268	-2.04037	1.107217
H	-4.581587	-1.190083	2.541331
C	-4.559852	-2.973963	2.483556
H	-2.08709	1.995675	-1.61811
H	-4.622962	0.425887	-0.446669
H	-2.024371	3.419966	-1.092472
C	-2.855417	3.939825	-1.580932
H	-0.694927	4.041714	-1.565065
H	0.150085	3.46902	-1.173683
H	-0.628452	4.051274	-2.658769
C	-0.611558	5.073176	-1.204871
C	-2.191153	3.506424	0.431471
H	-3.134183	3.054879	0.75033
H	-2.180376	4.557287	0.743039
H	-1.372061	2.995846	0.94306
C	1.078518	1.057657	1.156227
C	1.162073	2.421238	1.737305
C	1.775886	3.478242	1.051974
H	2.208032	3.300365	0.073278
C	1.842177	4.737423	1.643253
H	2.320905	5.557249	1.114791
C	1.296318	4.944929	2.912471
H	1.346505	5.929959	3.369314
C	0.688311	3.889716	3.5974
H	0.263713	4.052748	4.58395
C	0.62516	2.627697	3.01344
H	0.158256	1.791683	3.524319
C	0.607122	-0.466319	-3.200646
C	1.604444	-0.532244	-3.623162
H	-0.218163	-0.56457	-3.899719
H	4.259189	0.796848	-2.922997
H	4.644149	2.181401	-3.366877
C	5.314038	2.122489	-4.225409
C	5.153402	2.666537	-2.526559
O	3.755409	2.771477	-3.608975
C	4.617111	1.295817	0.919135
C	4.537255	1.936599	2.284308
C	3.638346	1.634671	2.822315
H	4.56569	3.022487	2.179567
H	5.417443	1.622987	2.858491
H	0.229864	-5.136535	1.133229
H	0.442028	-5.87839	0.357049
H	-0.787162	-5.287536	1.505145
H	0.919598	-5.297699	1.967587
H	-2.034327	-1.891228	2.333698
C	-1.775947	-2.885285	3.209478
O	-2.332658	-3.963402	3.222331
C	-0.694086	-2.445741	4.170995
H	-0.19181	-3.325901	4.576509
H	0.014659	-1.773107	3.680142
H	-1.158907	-1.897659	4.999339
C	-5.576913	0.443986	-1.62532
C	-6.152589	1.846909	-1.825437
C	-6.67054	-0.616933	-1.388085
H	-6.667814	2.188583	-0.922251
H	-5.351678	2.556015	-2.055731
H	-6.864468	1.847549	-2.658106
H	-7.360945	-0.640812	-2.237779
H	-6.234176	-1.614777	-1.274138
H	-7.249879	-0.383457	-0.4864
H	-4.989964	0.155092	-2.502914

Cartesian coordinates for conformer 4-1 after optimization at the PCM/B3LYP/6-31G* level of theory. Number of imaginary frequencies = 0. SCF Energy (PCM/mPW1PW91/6-31+G**) = -2568.99811652.

Atom	X	Y	Z
O	-1.996255	2.65803	-2.607995
O	-1.099861	1.607801	-0.807732
O	-3.942571	0.420859	-0.985002
O	1.402955	0.931058	0.024899
O	-2.636804	-1.815129	-2.51697
H	-2.124524	-2.493046	-3.004441
O	3.321245	0.336334	-1.712628
O	5.281484	0.662472	0.579391
O	-3.750319	-3.527614	2.494121
O	-2.927382	-1.492246	1.899038
O	3.44452	-0.523099	1.200348
O	-0.954585	-3.710281	-2.134675
O	0.339762	0.047651	1.831558
C	-3.565811	-2.401203	-0.331154
H	-3.538826	-3.393265	0.115837
H	-4.394631	-2.379622	-1.042355
C	-3.788774	-1.307029	0.726612
C	-3.122079	-0.07334	0.088635
H	-2.946017	0.716147	0.817205
C	-1.836024	-0.648955	-0.515654
H	-1.189871	-0.841661	0.341201
C	-1.139103	0.312324	-1.481489
H	-1.744203	0.414363	-2.379057
C	0.280279	-0.079807	-1.861775
C	1.26007	-0.300665	-0.720972
H	0.834313	-1.03575	-0.047477
C	2.655322	-0.792792	-1.112864
H	2.545673	-1.583537	-1.856552
C	3.536238	-1.360253	0.024535
H	4.568841	-1.324889	-0.327719
C	3.234904	-2.835309	0.445392
C	1.756702	-2.97629	0.747961
H	1.406842	-2.368124	1.584038
C	0.866817	-3.657023	0.024079
H	1.174601	-4.250551	-0.833018
C	-0.629577	-3.604609	0.232028
H	-0.871234	-2.890942	1.024142
C	-1.249237	-3.141327	-1.092911
C	-2.290542	-2.00547	-1.159686
C	-5.230329	-1.111141	1.168415
H	-5.609284	-2.026477	1.627614
H	-5.851523	-0.866387	0.302709
H	-5.294543	-0.286336	1.883797
C	0.581072	-0.263528	-3.150149
H	1.570391	-0.547544	-3.493239
H	-0.177003	-0.136463	-3.917301
C	3.714122	-3.765833	-0.68566
H	4.791115	-3.648644	-0.842493
H	3.22473	-3.567698	-1.643042
H	3.518284	-4.808738	-0.416758
C	4.04997	-3.157634	1.717565
H	3.711325	-2.565519	2.571599
H	5.115582	-2.951651	1.559204
H	3.940652	-4.218203	1.968394
C	-1.184125	-4.998816	0.609629
H	-1.02348	-5.701509	-0.213818
H	-2.25038	-4.964505	0.849228
H	-0.653038	-5.368596	1.492356
C	-2.997605	-2.593464	2.675908
C	-2.000727	-2.479059	3.807427
H	-1.769484	-3.474954	4.189142
H	-2.448581	-1.88851	4.615411

H	-1.096167	-1.959501	3.479306
C	0.900908	0.975934	1.272092
C	1.114364	2.309236	1.892097
C	1.800905	3.333356	1.225451
H	2.199654	3.150057	0.233563
C	1.968377	4.569722	1.845405
H	2.498592	5.365368	1.329259
C	1.455506	4.786224	3.127041
H	1.586319	5.752774	3.606714
C	0.775642	3.763857	3.793069
H	0.375665	3.933433	4.788814
C	0.605276	2.527097	3.177302
H	0.074625	1.71923	3.671121
C	4.393928	0.431205	1.37126
C	4.217044	1.140577	2.693432
H	4.177685	2.218856	2.518821
H	5.093218	0.933113	3.316985
H	3.311726	0.821095	3.210649
C	-1.512791	2.701341	-1.49533
C	-4.646897	1.563338	-0.778731
O	-4.71926	2.12784	0.291765
C	-5.266603	2.030414	-2.068237
H	-4.469487	2.462351	-2.684013
H	-5.69516	1.188733	-2.619067
H	-6.025903	2.785133	-1.859017
C	-1.268308	3.969824	-0.689547
C	-1.923658	3.899181	0.702412
C	-1.723886	5.199795	-1.477508
H	-0.180149	4.01019	-0.540544
H	-1.660612	4.794222	1.276561
H	-1.577085	3.026469	1.259532
H	-3.01287	3.840905	0.620196
H	-1.247528	5.245195	-2.461042
H	-2.807563	5.176359	-1.634454
H	-1.476853	6.111645	-0.923315
C	4.376702	0.061908	-2.518397
O	4.682137	-1.062189	-2.856053
C	5.100166	1.331111	-2.877905
H	4.392155	2.102545	-3.194809
H	5.60733	1.689706	-1.975008
H	5.830241	1.132258	-3.66326

Cartesian coordinates for conformer 4-2 after optimization at the PCM/B3LYP/6-31G* level of theory. Number of imaginary frequencies = 0. SCF Energy (PCM/mPW1PW91/6-31+G**) = -2568.99786497.

Atom	X	Y	Z
O	-3.094496	-2.910471	-0.846399
O	-2.532204	-0.828749	-1.550866
O	-3.271485	1.851408	-0.792571
O	2.066985	0.309575	-0.879959
O	-4.105625	-0.52527	0.771283
H	-4.232223	-1.437507	1.105983
O	2.446143	-2.125923	-1.716066
O	5.042772	-1.512199	-0.399168
O	-0.449226	4.296418	0.938022
O	-1.336223	2.630769	2.217428
O	3.383625	-1.086417	1.092865
O	-3.216834	-2.109702	2.59367
O	1.192817	1.780603	0.590581
C	-3.305198	1.318306	2.1347
H	-3.056429	1.350502	3.199455
H	-4.382851	1.456125	2.032193
C	-2.530898	2.431181	1.392382
C	-2.107404	1.771912	0.052318
H	-1.265181	2.272167	-0.422974

C	-1.800036	0.326728	0.473605
H	-0.897254	0.437024	1.079664
C	-1.473604	-0.744419	-0.586913
H	-1.423485	-1.706733	-0.060429
C	-0.159601	-0.524843	-1.311961
C	1.080321	-0.650756	-0.448003
H	0.830089	-0.434033	0.586324
C	1.735983	-2.035282	-0.469376
H	0.948137	-2.792279	-0.461533
C	2.675837	-2.29841	0.732536
H	3.410807	-3.045306	0.429744
C	1.974891	-2.81052	2.028285
C	0.982553	-1.777352	2.535386
H	1.399632	-0.777718	2.669097
C	-0.302956	-1.98684	2.828826
H	-0.745037	-2.975046	2.722798
C	-1.271955	-0.929026	3.319158
H	-0.849694	0.070906	3.174096
C	-2.539124	-1.100532	2.478696
C	-2.962935	-0.042123	1.449875
C	-3.331877	3.722947	1.263278
H	-3.531583	4.125433	2.262267
H	-4.285163	3.511173	0.774568
H	-2.78806	4.468109	0.682907
C	-0.067624	-0.255916	-2.613962
H	0.902118	-0.111487	-3.077565
H	-0.947327	-0.139	-3.232566
C	1.331362	-4.181352	1.745733
H	2.055534	-4.854085	1.277091
H	0.46624	-4.116054	1.079705
H	0.993049	-4.636785	2.682106
C	3.067942	-2.998283	3.109463
H	3.553668	-2.05063	3.357024
H	3.837235	-3.698835	2.762109
H	2.620667	-3.40441	4.022555
C	-1.612421	-1.128036	4.810523
H	-2.064871	-2.111405	4.966895
H	-2.319424	-0.365163	5.157656
H	-0.70059	-1.056857	5.411245
C	-0.409215	3.573394	1.909423
C	0.663752	3.591748	2.97076
H	1.385384	4.376987	2.741522
H	1.1673	2.62188	2.981596
H	0.219443	3.764249	3.956384
C	1.972294	1.526435	-0.314045
C	2.915358	2.501804	-0.908085
C	2.738719	3.857633	-0.600921
H	1.901227	4.154429	0.024407
C	3.612811	4.801489	-1.133836
H	3.472688	5.855032	-0.908413
C	4.662264	4.39598	-1.961889
H	5.344851	5.135416	-2.373178
C	4.834043	3.043874	-2.270816
H	5.648748	2.731696	-2.918526
C	3.958136	2.093584	-1.752469
H	4.081215	1.040334	-1.984239
C	4.555005	-0.81778	0.464545
C	5.174367	0.43546	1.034967
H	4.411995	1.183581	1.262308
H	5.898157	0.840368	0.327259
H	5.68918	0.181022	1.969001
C	-3.31247	-1.936463	-1.534574
C	-3.095178	2.047921	-2.119618
O	-2.019207	2.152617	-2.663118
C	-4.437258	2.13769	-2.805329
H	-4.978663	3.017466	-2.441132

H	-4.291845	2.213977	-3.883477
H	-5.039806	1.257817	-2.564528
C	-4.493313	-1.766806	-2.475434
C	-5.347128	-3.036146	-2.503355
C	-4.036633	-1.338565	-3.882002
H	-5.075799	-0.946294	-2.031968
H	-4.777836	-3.875697	-2.916383
H	-6.235025	-2.880928	-3.125674
H	-5.667885	-3.319226	-1.497172
H	-3.449938	-0.417002	-3.85255
H	-3.421572	-2.121085	-4.341807
H	-4.907718	-1.175286	-4.526035
C	2.863388	-3.357151	-2.094047
O	2.583354	-4.373148	-1.493667
C	3.741408	-3.256057	-3.312564
H	4.678529	-2.774737	-3.010812
H	3.943243	-4.252646	-3.70668
H	3.273079	-2.627309	-4.07541

Cartesian coordinates for conformer 4-3 after optimization at the PCM/B3LYP/6-31G* level of theory. Number of imaginary frequencies = 0. SCF Energy (PCM/mPW1PW91/6-31+G**) = -2568.9976574.

Atom	X	Y	Z
O	-3.923249	0.395288	-1.094893
O	1.378174	0.949205	-0.125173
O	3.317801	0.386439	-1.836099
O	5.323281	0.767375	0.396225
O	-1.097129	1.602656	-1.044695
H	0.406907	0.06683	1.732819
O	3.49488	-0.404909	1.065567
O	-2.627449	-1.94988	-2.46367
O	-2.129449	-2.672133	-2.899822
O	-0.955131	-3.817665	-1.950716
O	-1.967059	2.507694	-2.93428
O	4.705127	-0.984372	-2.98212
O	-4.68257	2.201627	0.048871
C	-3.529982	-2.364612	-0.227573
H	-4.366865	-2.3981	-0.928673
H	-3.495728	-3.319724	0.293562
C	-3.741266	-1.192628	0.744937
C	-3.087749	-0.011293	0.003735
H	-2.905888	0.833709	0.665629
C	-1.807502	-0.628737	-0.571288
H	-1.1495	-0.754589	0.28855
C	-1.131335	0.259232	-1.61832
H	-1.7492	0.286295	-2.512543
C	0.289298	-0.134819	-1.989679
C	1.274171	-0.302196	-0.844672
H	0.871132	-1.036583	-0.15697
C	2.685121	-0.756132	-1.226419
H	2.605109	-1.558687	-1.961166
C	3.580893	-1.282545	-0.081376
H	4.608878	-1.25168	-0.448157
C	3.298119	-2.744017	0.398038
C	1.835804	-2.869031	0.775683
H	1.520457	-2.205552	1.581692
C	0.917629	-3.595759	0.136764
H	1.190465	-4.248428	-0.688491
C	-0.571174	-3.514206	0.388806
H	-0.78518	-2.733322	1.12401
C	-1.223738	-3.160409	-0.955063
C	-2.265469	-2.032121	-1.099645
C	3.720774	-3.711927	-0.723402
H	4.78077	-3.579102	-0.962481
H	3.157149	-3.567286	-1.649099

H	3.569816	-4.746888	-0.399607
C	4.173908	-3.023645	1.640072
H	3.881316	-2.395856	2.485614
H	5.231801	-2.833583	1.42127
C	4.069457	-4.072423	1.937833
H	-5.176941	-0.965162	1.190565
H	-5.809941	-0.787462	0.317141
H	-5.233178	-0.088235	1.841951
C	-5.547623	-1.843127	1.723369
H	-1.494248	2.638434	-1.823096
H	-4.620906	1.553791	-0.974106
H	-5.247473	1.921184	-2.292545
C	-5.683856	1.041069	-2.772702
H	-4.452644	2.298772	-2.946132
H	-6.001533	2.694252	-2.138777
H	-1.243259	3.966452	-1.121458
C	-0.152039	4.019787	-0.995446
C	-1.872889	4.004785	0.283585
H	-2.96237	3.922315	0.227625
H	-1.616203	4.950386	0.773575
H	-1.500673	3.189669	0.907403
C	-1.714955	5.13001	-1.996186
C	-1.256586	5.097032	-2.988776
C	-1.460424	6.083403	-1.520907
H	-2.801372	5.093375	-2.13109
C	0.912928	1.00348	1.134437
H	1.085462	2.359659	1.71488
C	1.723595	3.389032	1.009703
H	2.115544	3.192004	0.017848
C	1.852802	4.647752	1.59216
H	2.346757	5.447367	1.047028
C	1.348065	4.881893	2.874405
H	1.448487	5.865917	3.325049
C	0.715312	3.854324	3.578205
C	0.321765	4.037548	4.574222
H	0.58468	2.594745	3.000231
H	0.091918	1.782002	3.524203
H	0.590412	-0.352407	-3.272729
C	1.584176	-0.628061	-3.610646
C	-0.171221	-0.25983	-4.041301
O	4.375784	0.133648	-2.646666
C	5.069097	1.417164	-3.013536
H	4.342552	2.175572	-3.31946
H	5.792599	1.233578	-3.8086
H	5.58157	1.782553	-2.116451
C	4.438901	0.559983	1.198443
C	4.258528	1.319361	2.492136
C	5.150758	1.169334	3.109243
H	3.371718	0.99356	3.036609
H	4.179563	2.387274	2.271952
H	-1.121975	-4.868062	0.896496
H	-0.986984	-5.638069	0.130652
H	-2.181124	-4.807252	1.161307
H	-0.568052	-5.166928	1.792037
H	-2.860482	-1.284922	1.913658
C	-2.912856	-2.32471	2.772594
O	-3.672731	-3.26618	2.679871
C	-1.886338	-2.131867	3.866801
H	-1.624357	-3.102693	4.29139
H	-1.002392	-1.612793	3.48619
H	-2.325317	-1.51239	4.658241

Cartesian coordinates for conformer 4-4 after optimization at the PCM/B3LYP/6-31G* level of theory. Number of imaginary frequencies = 0. SCF Energy (PCM/mPW1PW91/6-31+G**) = -2568.99745457.

Atom	X	Y	Z
O	-3.233956	1.795601	-0.817041
O	2.08201	0.304017	-0.836896
O	2.511273	-2.159165	-1.575039
O	5.091679	-1.432799	-0.293231
O	-2.495554	-0.886434	-1.502736
H	1.196698	1.800838	0.600168
O	3.428483	-0.976907	1.184981
O	-4.078915	-0.574943	0.818574
O	-4.174637	-1.491969	1.150013
O	-3.145324	-2.11293	2.663277
O	-3.088084	-2.952022	-0.775234
O	2.699661	-4.390101	-1.251274
O	-1.934812	2.086702	-2.657117
C	-3.313367	1.314953	2.130054
H	-4.390006	1.438661	2.00229
H	-3.0875	1.376646	3.198624
C	-2.537977	2.416899	1.374959
C	-2.089019	1.734068	0.054935
H	-1.239008	2.228839	-0.410828
C	-1.779847	0.298878	0.506514
H	-0.881128	0.42537	1.11415
C	-1.444592	-0.792309	-0.531554
H	-1.402568	-1.746311	0.010809
C	-0.123605	-0.588382	-1.250537
C	1.11209	-0.659246	-0.373516
H	0.851146	-0.407048	0.64976
C	1.798742	-2.028158	-0.333345
H	1.028076	-2.801357	-0.290601
C	2.745092	-2.216849	0.878267
H	3.495609	-2.960144	0.606289
C	2.057628	-2.688073	2.196626
C	1.033143	-1.663734	2.655239
H	1.419793	-0.64723	2.746486
C	-0.246981	-1.898875	2.952903
H	-0.659092	-2.903427	2.886005
C	-1.251406	-0.85232	3.393041
H	-0.853687	0.153506	3.222963
C	-2.501802	-1.082784	2.540258
C	-2.94491	-0.057682	1.486565
C	1.456953	-4.089075	1.976105
H	2.204301	-4.762509	1.546622
H	0.598052	-4.078951	1.299462
H	1.121734	-4.508916	2.93015
C	3.153396	-2.793545	3.286168
H	3.608404	-1.821301	3.493132
H	3.945394	-3.483896	2.970748
C	2.716787	-3.173629	4.215493
H	-3.347253	3.699154	1.207513
H	-4.293609	3.468677	0.713777
H	-2.80315	4.435013	0.61578
C	-3.56058	4.12343	2.194521
H	-3.295155	-1.98012	-1.469353
H	-3.024256	1.988006	-2.140327
H	-4.347867	2.091038	-2.859273
C	-4.857693	3.011075	-2.552728
H	-4.992676	1.250926	-2.588947
H	-4.177404	2.109252	-3.936159
H	-4.481737	-1.793165	-2.39961
C	-5.028916	-0.942623	-1.968729
C	-5.379172	-3.031882	-2.387549
H	-4.844564	-3.901069	-2.785165
H	-6.268233	-2.860503	-3.003895
H	-5.698796	-3.278167	-1.371171
C	-4.027372	-1.413384	-3.820929
C	-4.899784	-1.231893	-4.458287

C	-3.446944	-2.227727	-4.270492
H	-3.40748	-0.513354	-3.818518
C	1.976003	1.533289	-0.300241
H	2.909404	2.503122	-0.919572
C	2.723836	3.864249	-0.641556
H	1.886401	4.168472	-0.019538
C	3.591404	4.802025	-1.195772
H	3.445025	5.859135	-0.992159
C	4.642718	4.385642	-2.015729
H	5.320182	5.120487	-2.44313
C	4.823509	3.028632	-2.295399
C	5.640147	2.707525	-2.936178
H	3.954631	2.083846	-1.755851
H	4.085986	1.026522	-1.963002
H	-0.023018	-0.369039	-2.561279
C	0.948998	-0.231222	-3.022256
C	-0.898352	-0.286359	-3.191498
O	2.958062	-3.396282	-1.896461
C	3.837169	-3.329845	-3.116672
H	4.064736	-4.33835	-3.463641
H	4.761064	-2.811621	-2.835782
H	3.355387	-2.74908	-3.908724
C	4.596222	-0.715312	0.546861
C	5.201868	0.56327	1.074309
C	5.73125	0.342581	2.00889
H	4.430995	1.306289	1.289324
H	5.911574	0.958713	0.347228
H	-1.611247	-1.012705	4.88476
H	-2.04054	-2.002362	5.065583
H	-2.343397	-0.258213	5.196307
H	-0.711639	-0.898489	5.497081
H	-1.356832	2.643109	2.213085
C	-0.434955	3.589961	1.900033
O	-0.468167	4.292463	0.913835
C	0.629913	3.633868	2.96851
H	1.208972	2.708097	2.917511
H	1.288402	4.484247	2.786747
H	0.177441	3.708722	3.961982

Cartesian coordinates for conformer 4-5 after optimization at the PCM/B3LYP/6-31G* level of theory. Number of imaginary frequencies = 0. SCF Energy (PCM/mPW1PW91/6-31+G**) = -2568.99714872.

Atom	X	Y	Z
O	-1.398296	2.758644	-2.586816
O	-0.90852	1.561049	-0.728009
O	-3.654551	0.885897	-0.922217
O	1.706505	0.911306	-0.353568
O	-2.901629	-1.718095	-2.324468
H	-2.46977	-2.352744	-2.933105
O	3.30508	0.113705	-2.265321
O	5.471927	0.511428	-0.15202
O	-2.227241	-1.427666	2.507216
O	-4.391667	-1.175135	1.862066
O	3.742109	-0.693812	0.696906
O	-1.094261	-3.560768	-2.332312
O	0.744475	0.286731	1.610675
C	-3.385286	-2.426128	-0.051673
H	-2.931061	-2.933576	0.796709
H	-4.078436	-3.112757	-0.544389
C	-4.085735	-1.134216	0.432771
C	-3.097315	0.03298	0.099745
H	-2.898006	0.641029	0.979391
C	-1.80823	-0.617871	-0.456473
H	-1.173879	-0.813575	0.41026
C	-1.084886	0.305784	-1.446327

H	-1.740273	0.489559	-2.293816
C	0.259806	-0.175032	-1.966132
C	1.380592	-0.361332	-0.957291
H	1.022983	-1.00778	-0.161802
C	2.680693	-0.949532	-1.51711
H	2.427317	-1.764178	-2.196047
C	3.682971	-1.519435	-0.488673
H	4.669739	-1.499146	-0.955134
C	3.395905	-2.991861	-0.049171
C	1.940312	-3.109983	0.356385
H	1.676359	-2.573637	1.270028
C	0.972715	-3.688251	-0.357216
H	1.193769	-4.20674	-1.28723
C	-0.495138	-3.62728	-0.012469
H	-0.654096	-2.9844	0.859324
C	-1.251759	-3.071223	-1.223348
C	-2.30567	-1.963554	-1.063732
C	-5.455444	-0.967017	-0.21348
H	-6.091754	-1.80704	0.081354
H	-5.347919	-0.961059	-1.299823
H	-5.928332	-0.03929	0.118845
C	0.380724	-0.441062	-3.269836
H	1.300504	-0.796678	-3.721729
H	-0.464074	-0.312379	-3.939641
C	3.775047	-3.92213	-1.21902
H	4.848542	-3.853169	-1.421854
H	3.259998	-3.678738	-2.151802
H	3.539577	-4.9592	-0.959192
C	4.295903	-3.338096	1.156385
H	4.029999	-2.750145	2.038473
H	5.349823	-3.144938	0.921486
H	4.191368	-4.399903	1.404325
C	-1.039266	-5.045319	0.289876
H	-0.887019	-5.696923	-0.576346
H	-2.108786	-5.032517	0.525648
H	-0.501775	-5.465339	1.145091
C	-3.406109	-1.296514	2.773501
C	-3.973964	-1.24104	4.171416
H	-4.423591	-0.257181	4.342814
H	-3.176484	-1.416459	4.894389
H	-4.765551	-1.987936	4.288732
C	1.368651	1.087877	0.940099
C	1.871849	2.392123	1.449054
C	2.700992	3.216965	0.675789
H	2.984447	2.902719	-0.322882
C	3.164754	4.420033	1.204505
H	3.808839	5.059451	0.607153
C	2.804163	4.801765	2.499262
H	3.166481	5.741719	2.907699
C	1.98084	3.977301	3.270717
H	1.701128	4.275021	4.277551
C	1.516878	2.773489	2.74775
H	0.876982	2.114767	3.326383
C	4.721044	0.245484	0.760897
C	4.781575	0.864435	2.137024
H	5.009028	1.928215	2.046593
H	5.595741	0.38349	2.692385
H	3.848963	0.724854	2.684443
C	-1.064863	2.71296	-1.420224
C	-4.292543	2.01377	-0.528215
O	-4.48434	2.312031	0.631882
C	-4.70465	2.822806	-1.731473
H	-3.812597	3.111424	-2.296603
H	-5.326813	2.211993	-2.393587
H	-5.255541	3.706062	-1.405564
C	-0.746543	3.920853	-0.548427

C	-1.38179	3.822837	0.850098
C	-1.127829	5.216146	-1.270174
H	0.3445	3.891509	-0.419283
H	-1.081972	2.904786	1.359739
H	-2.474817	3.835732	0.791321
H	-1.058096	4.673243	1.460154
H	-0.671597	5.267854	-2.26247
H	-2.214278	5.282649	-1.399812
H	-0.801045	6.081868	-0.684172
C	4.219508	-0.25015	-3.194436
O	4.427944	-1.40265	-3.512369
C	4.935484	0.964131	-3.722518
H	5.552111	0.688164	-4.578748
H	4.21879	1.743445	-3.998366
H	5.564785	1.357748	-2.916298

Cartesian coordinates for conformer 4-6 after optimization at the PCM/B3LYP/6-31G* level of theory. Number of imaginary frequencies = 0. SCF Energy (PCM/mPW1PW91/6-31+G**) = -2568.996825.

Atom	X	Y	Z
O	-3.690506	0.803346	-1.018968
O	1.620397	1.000801	-0.307152
O	3.334308	0.225852	-2.105353
O	5.390554	0.725317	0.090116
O	-0.978177	1.573049	-0.861525
H	0.590274	0.407722	1.631873
O	3.659906	-0.516282	0.882679
O	-2.877817	-1.818709	-2.294077
O	-2.407373	-2.44823	-2.878856
O	-1.005613	-3.597041	-2.217129
O	-1.384997	2.610228	-2.833605
O	4.582332	-1.265095	-3.26069
O	-4.486275	2.356689	0.428502
C	-3.370939	-2.436272	0.000685
H	-4.064856	-3.12679	-0.485418
H	-2.924951	-2.935257	0.858533
C	-4.071565	-1.134227	0.461261
C	-3.111461	0.032367	0.054496
H	-2.92737	0.69901	0.894837
C	-1.811469	-0.620582	-0.465949
H	-1.181458	-0.771961	0.41159
C	-1.095314	0.265211	-1.494065
H	-1.733877	0.36768	-2.367991
C	0.283422	-0.189687	-1.943603
C	1.367413	-0.301083	-0.884188
H	1.004314	-0.938372	-0.083706
C	2.713155	-0.849398	-1.372511
H	2.524377	-1.682086	-2.050244
C	3.68028	-1.368918	-0.28556
H	4.687603	-1.332114	-0.704784
C	3.410835	-2.83837	0.175616
C	1.945935	-2.984523	0.536395
H	1.638914	-2.431429	1.426234
C	1.016238	-3.610113	-0.187543
H	1.277519	-4.148075	-1.09588
C	-0.461438	-3.582145	0.117844
H	-0.661917	-2.919767	0.9666
C	-1.204535	-3.084093	-1.125546
C	-2.287698	-1.998264	-1.019633
C	3.851413	-3.786526	-0.957423
H	4.927833	-3.690863	-1.132311
H	3.355549	-3.584482	-1.910172
H	3.638663	-4.823026	-0.676261
C	4.276312	-3.136614	1.41923
H	3.968407	-2.533502	2.2771

H	5.333433	-2.927186	1.214892
C	4.184708	-4.194027	1.689927
H	-5.464166	-1.002822	-0.14291
H	-5.394883	-1.041783	-1.231842
H	-5.930786	-0.063304	0.164728
C	-6.084444	-1.832907	0.208572
H	-1.093055	2.662818	-1.655681
H	-4.311718	1.965379	-0.706142
H	-4.728311	2.682898	-1.96514
C	-5.26965	3.593164	-1.704172
H	-5.360098	2.028458	-2.574292
H	-3.838508	2.920772	-2.557347
H	-0.78003	3.93595	-0.880846
C	0.305165	3.893067	-0.708749
C	-1.464994	3.974797	0.496934
H	-2.554553	4.008033	0.398593
H	-1.138601	4.866683	1.042944
H	-1.208782	3.097858	1.094642
C	-1.103749	5.17096	-1.725552
C	-0.610084	5.127302	-2.700034
C	-0.77918	6.077696	-1.203947
H	-2.182807	5.247078	-1.902662
C	1.223749	1.204788	0.965132
H	1.677347	2.532881	1.458292
C	2.537339	3.34567	0.70658
H	2.879049	3.004846	-0.26491
C	2.959966	4.568303	1.224738
H	3.629716	5.197642	0.645212
C	2.527197	4.981542	2.487243
H	2.858252	5.93609	2.888055
C	1.672544	4.169361	3.237204
C	1.33724	4.491324	4.219287
H	1.249602	2.946088	2.725139
H	0.588275	2.29497	3.288355
H	0.469804	-0.482896	-3.233557
C	1.421011	-0.812651	-3.637879
C	-0.349182	-0.400496	-3.941676
O	4.313127	-0.116661	-2.97524
C	5.014287	1.11539	-3.481168
H	4.287945	1.869798	-3.797542
H	5.679671	0.852168	-4.304292
H	5.592418	1.533969	-2.649459
C	4.606916	0.452591	0.973136
C	4.586462	1.094483	2.339868
C	5.36742	0.624379	2.949841
H	3.624106	0.959362	2.834527
H	4.815648	2.157413	2.245214
H	-0.973309	-5.006749	0.445495
H	-0.780023	-5.677172	-0.397939
H	-2.048902	-5.018166	0.652329
H	-0.447986	-5.388058	1.326098
H	-4.330418	-1.121329	1.901535
C	-3.324321	-1.22922	2.791466
O	-2.154778	-1.390297	2.503304
C	-3.85834	-1.122231	4.199934
H	-4.640384	-1.87038	4.364567
H	-3.042338	-1.265367	4.909248
H	-4.312592	-0.136442	4.345251

Cartesian coordinates for conformer 4-7 after optimization at the PCM/B3LYP/6-31G* level of theory. Number of imaginary frequencies = 0. SCF Energy (PCM/mPW1PW91/6-31+G**) = -2568.99693987.

Atom	X	Y	Z
O	-2.95472	1.537357	-1.092822
O	1.994795	0.417225	-1.215926
O	2.375144	-2.016553	-1.727891
O	4.939357	-1.028299	-0.374174
O	-2.567556	-1.182664	-1.316103
H	1.207738	2.250648	-0.140586
O	3.226899	-0.740162	1.089486
O	-4.184235	-0.293181	0.886054
O	-4.535083	-1.075219	1.363755
O	-3.593374	-1.924216	2.792178
O	-3.099558	-3.003442	-0.073472
O	2.485359	-4.248047	-1.3704
O	-1.804228	3.075284	-2.308689
C	-3.084853	1.397394	2.239555
H	-4.127064	1.553328	2.526634
H	-2.466415	1.468525	3.13347
C	-2.622588	2.440655	1.181711
C	-2.020314	1.621556	-0.000667
H	-1.103439	2.085506	-0.360019
C	-1.795836	0.217572	0.563063
H	-0.913976	0.322798	1.195442
C	-1.458831	-0.884725	-0.459205
H	-1.214671	-1.791048	0.105131
C	-0.272433	-0.48419	-1.313924
C	1.041753	-0.434866	-0.560103
H	0.891084	-0.055979	0.450629
C	1.689538	-1.832375	-0.47552
H	0.900423	-2.582668	-0.405264
C	2.642654	-2.014845	0.726457
H	3.449366	-2.686207	0.427965
C	1.969146	-2.598096	2.003584
C	0.853302	-1.693113	2.496665
H	1.16335	-0.671584	2.722555
C	-0.414715	-2.03979	2.743269
H	-0.778461	-3.045704	2.540317
C	-1.416711	-1.113569	3.408001
H	-0.990612	-0.107876	3.427879
C	-2.716035	-1.09677	2.604606
C	-2.969993	-0.000587	1.553038
C	1.498556	-4.036313	1.718448
H	2.318212	-4.637095	1.315078
H	0.680351	-4.082345	0.995576
H	1.149909	-4.501225	2.646231
C	3.046357	-2.655014	3.116081
H	3.410455	-1.656857	3.373268
H	3.90164	-3.26076	2.791737
C	2.625885	-3.11102	4.018327
H	-3.756077	3.359629	0.747947
H	-4.588878	2.759916	0.374255
H	-3.415924	4.039963	-0.038316
C	-4.093504	3.949528	1.605628
H	-3.372554	-2.208633	-0.947901
H	-2.700972	2.268092	-2.203077
H	-3.703536	1.916214	-3.275713
C	-3.662173	0.839954	-3.472346
H	-4.716307	2.14304	-2.926142
H	-3.486136	2.479729	-4.183859
H	-4.638795	-2.205772	-1.785795
C	-5.050353	-1.194944	-1.667891
C	-4.302925	-2.425685	-3.272962
H	-3.856155	-3.415135	-3.427865
H	-5.214317	-2.369314	-3.878278

H	-3.597509	-1.673115	-3.637919
C	-5.635902	-3.239388	-1.259143
C	-6.565721	-3.198533	-1.837213
C	-5.222275	-4.250458	-1.33435
H	-5.871605	-3.059244	-0.205719
C	1.987126	1.73201	-0.919671
H	3.066878	2.451967	-1.642389
C	3.08893	3.85035	-1.574133
H	2.30241	4.360938	-1.02716
C	4.103581	4.561103	-2.208504
H	4.118298	5.646487	-2.159389
C	5.101984	3.876601	-2.906688
H	5.897348	4.431855	-3.397762
C	5.081718	2.481246	-2.974483
C	5.862321	1.950459	-3.512386
H	4.064597	1.764091	-2.348548
H	4.051739	0.680216	-2.379133
H	-0.34934	-0.226604	-2.619088
C	0.531018	0.062919	-3.182107
C	-1.29212	-0.29453	-3.148772
O	2.758337	-3.274734	-2.040572
C	3.590184	-3.265847	-3.296767
H	3.093574	-2.689457	-4.082835
H	3.770529	-4.289459	-3.62714
H	4.542784	-2.772052	-3.073457
C	4.373405	-0.365992	0.46693
C	4.840485	0.965741	1.002613
C	5.305171	0.811835	1.983581
H	3.996311	1.646727	1.136872
H	5.571289	1.397535	0.318485
H	-1.698348	-1.586424	4.8474
H	-2.126336	-2.592869	4.84532
H	-2.410665	-0.918645	5.344983
H	-0.766778	-1.596465	5.421267
H	-1.610773	3.37872	1.681377
C	-0.449189	2.993846	2.243974
O	-0.161778	1.857028	2.57004
C	0.483389	4.168818	2.391977
H	-0.061984	5.101315	2.552481
H	1.182866	3.978674	3.208269
H	1.046576	4.242978	1.45504

Cartesian coordinates for conformer 4-8 after optimization at the PCM/B3LYP/6-31G* level of theory. Number of imaginary frequencies = 0. SCF Energy (PCM/mPW1PW91/6-31+G**) = -2568.99652721.

Atom	X	Y	Z
O	-3.128909	-3.007376	-0.087884
O	-2.557614	-1.188408	-1.31482
O	-2.936496	1.534578	-1.088751
O	1.988552	0.427968	-1.206442
O	-4.192938	-0.293253	0.87968
H	-4.539353	-1.088184	1.339275
O	2.40345	-2.029226	-1.621041
O	4.95452	-0.900931	-0.275787
O	-0.149666	1.814024	2.578442
O	-1.58335	3.354819	1.699586
O	3.212942	-0.606915	1.151945
O	-3.611428	-1.944161	2.771044
O	1.213894	2.278254	-0.1492
C	-3.091739	1.393337	2.237022
H	-2.485369	1.460591	3.139526
H	-4.13622	1.55968	2.509753
C	-2.605126	2.432864	1.186896
C	-2.006126	1.60784	0.007915
H	-1.083492	2.063469	-0.346187

C	-1.798151	0.20237	0.573831
H	-0.92308	0.301738	1.215186
C	-1.456668	-0.902178	-0.44403
H	-1.224601	-1.811148	0.122841
C	-0.261491	-0.505921	-1.290687
C	1.044654	-0.41515	-0.525578
H	0.879305	-0.000208	0.468189
C	1.713402	-1.79729	-0.37885
H	0.934859	-2.554695	-0.272941
C	2.669805	-1.91015	0.830053
H	3.498271	-2.56475	0.553894
C	2.011898	-2.472772	2.12415
C	0.846353	-1.607077	2.570915
H	1.106061	-0.568361	2.781591
C	-0.41088	-2.003973	2.798451
H	-0.72805	-3.027629	2.60635
C	-1.458085	-1.112345	3.439895
H	-1.054372	-0.098958	3.482496
C	-2.738579	-1.107498	2.606136
C	-2.981503	-0.007132	1.554165
C	-3.719122	3.372036	0.745622
H	-4.054266	3.965623	1.601633
H	-4.558654	2.787925	0.362343
H	-3.360257	4.048308	-0.035802
C	-0.327923	-0.27247	-2.601203
H	0.554543	0.018399	-3.160041
H	-1.26377	-0.361317	-3.139515
C	1.618226	-3.9432	1.895414
H	2.479094	-4.523522	1.552395
H	0.83192	-4.065356	1.146996
H	1.259174	-4.376121	2.83442
C	3.076006	-2.429911	3.249669
H	3.378746	-1.404565	3.477086
H	3.968792	-2.995645	2.955527
H	2.67053	-2.879535	4.161903
C	-1.767113	-1.60831	4.865638
H	-2.173081	-2.623417	4.840747
H	-2.506988	-0.962134	5.351478
H	-0.851291	-1.605206	5.464316
C	-0.427193	2.956323	2.261922
C	0.512446	4.123715	2.424601
H	1.067486	4.214478	1.484442
H	1.217274	3.914746	3.231631
H	-0.027733	5.05517	2.607904
C	1.980756	1.748062	-0.933194
C	3.046034	2.459672	-1.684691
C	4.035381	1.764485	-2.395286
H	4.025404	0.680328	-2.408255
C	5.04078	2.475342	-3.046717
H	5.815429	1.939163	-3.587818
C	5.057732	3.871408	-2.999312
H	5.844554	4.421475	-3.50939
C	4.067584	4.563166	-2.296785
H	4.079831	5.649051	-2.263524
C	3.06453	3.859071	-1.63719
H	2.284616	4.374928	-1.086009
C	4.359469	-0.22514	0.533603
C	4.785913	1.134668	1.031539
H	5.495844	1.573062	0.329817
H	5.266067	1.019568	2.010362
H	3.920715	1.790059	1.158723
C	-3.377503	-2.208279	-0.965207
C	-2.660544	2.251419	-2.203383
O	-1.747128	3.039328	-2.309591
C	-3.662507	1.911293	-3.280207
H	-4.673346	2.157992	-2.938847

H	-3.428993	2.465502	-4.190135
H	-3.638331	0.833517	-3.470583
C	-4.627211	-2.190384	-1.827927
C	-5.642583	-3.219552	-1.328582
C	-4.264622	-2.40287	-3.309798
H	-5.033411	-1.17721	-1.71119
H	-5.89812	-3.044826	-0.278982
H	-5.235689	-4.233362	-1.402773
H	-6.560216	-3.167177	-1.924975
H	-3.546174	-1.654088	-3.656484
H	-3.82328	-3.394946	-3.463187
H	-5.163522	-2.33457	-3.932288
C	2.794438	-3.29683	-1.880797
O	2.528991	-4.24257	-1.16972
C	3.622776	-3.335568	-3.138962
H	3.820079	-4.37094	-3.418839
H	3.112233	-2.808607	-3.950495
H	4.567008	-2.813868	-2.946191

Cartesian coordinates for conformer 4-9 after optimization at the PCM/B3LYP/6-31G* level of theory. Number of imaginary frequencies = 0. SCF Energy (PCM/mPW1PW91/6-31+G**) = -2568.99657788.

Atom	X	Y	Z
O	-1.834968	2.449742	-3.086806
O	-1.219314	1.630663	-1.063342
O	-3.94256	0.332955	-1.075386
O	1.229707	1.120709	0.024128
O	-2.644896	-1.943435	-2.487892
H	-2.069538	-2.587858	-2.950218
O	3.319688	0.616468	-1.538491
O	5.041244	1.116056	0.86932
O	-3.003799	0.350054	2.994165
O	-2.875608	-1.637089	1.882976
O	3.262262	-0.199578	1.388586
O	-0.850122	-3.71311	-2.0271
O	0.15219	0.187262	1.791083
C	-3.540911	-2.382237	-0.2608
H	-3.490165	-3.346749	0.249962
H	-4.381459	-2.406993	-0.95679
C	-3.731195	-1.243118	0.753333
C	-3.092441	-0.041476	0.029537
H	-2.931238	0.813166	0.67935
C	-1.817329	-0.639375	-0.566381
H	-1.155255	-0.794556	0.283915
C	-1.148086	0.272782	-1.597882
H	-1.723213	0.238981	-2.520399
C	0.31349	-0.033313	-1.889063
C	1.235725	-0.143796	-0.686518
H	0.827389	-0.889215	-0.014078
C	2.682957	-0.545173	-0.971811
H	2.68022	-1.347825	-1.710938
C	3.497069	-1.048529	0.241644
H	4.552434	-0.96679	-0.025096
C	3.233177	-2.528807	0.669769
C	1.749922	-2.735469	0.914557
H	1.338156	-2.129751	1.722931
C	0.923557	-3.482464	0.179564
H	1.294236	-4.075063	-0.65335
C	-0.579741	-3.523248	0.341898
H	-0.906468	-2.825103	1.116393
C	-1.194498	-3.131417	-1.007626
C	-2.275395	-2.036419	-1.127947
C	-5.16035	-1.034912	1.229356
H	-5.518355	-1.934527	1.740939
H	-5.809843	-0.840192	0.372187

H	-5.211737	-0.183487	1.91014
C	0.708515	-0.202207	-3.153805
H	1.738938	-0.401069	-3.430228
H	-0.006474	-0.138762	-3.968768
C	3.800897	-3.456316	-0.420886
H	4.871634	-3.274492	-0.557312
H	3.321612	-3.316981	-1.393366
H	3.664245	-4.501816	-0.127002
C	4.002732	-2.794629	1.983406
H	3.609845	-2.191815	2.806216
H	5.067368	-2.559021	1.864149
H	3.915224	-3.851375	2.257523
C	-1.051006	-4.947918	0.714685
H	-0.770636	-5.656194	-0.071177
H	-2.136619	-4.994352	0.850635
H	-0.574817	-5.255943	1.650804
C	-2.590227	-0.785448	2.90173
C	-1.652601	-1.441283	3.885939
H	-1.815021	-1.011427	4.876585
H	-0.630977	-1.209639	3.567754
H	-1.784192	-2.525322	3.909361
C	0.628511	1.160473	1.226156
C	0.598613	2.531515	1.787593
C	1.394011	3.559668	1.263389
H	2.051456	3.351524	0.425477
C	1.336848	4.830925	1.828828
H	1.955384	5.629692	1.428886
C	0.480373	5.079154	2.905415
H	0.432566	6.074375	3.34007
C	-0.319619	4.055815	3.419013
H	-0.997415	4.255228	4.244098
C	-0.260216	2.778913	2.866165
H	-0.895924	1.977885	3.232663
C	4.115799	0.833442	1.598661
C	3.780227	1.553069	2.883768
H	3.810952	2.631277	2.711361
H	4.546669	1.307835	3.627457
H	2.800168	1.266456	3.2669
C	-1.482654	2.634114	-1.939673
C	-4.626342	1.504114	-0.997919
O	-4.713619	2.173562	0.006981
C	-5.200202	1.85121	-2.347444
H	-5.639427	0.968949	-2.821315
H	-5.941939	2.643901	-2.239929
H	-4.376896	2.194534	-2.985057
C	-1.257069	3.991302	-1.291928
C	-2.161504	4.189267	-0.061214
C	-1.429388	5.111024	-2.320547
H	-0.217568	3.971211	-0.937106
H	-2.020755	3.392717	0.671451
H	-3.218342	4.19996	-0.344104
H	-1.917934	5.142672	0.419916
H	-0.771301	4.968671	-3.183059
H	-2.459272	5.13902	-2.691633
H	-1.201925	6.079132	-1.861235
C	4.456705	0.402053	-2.245872
O	4.869403	-0.704232	-2.523295
C	5.114205	1.712239	-2.584183
H	4.381053	2.421991	-2.978645
H	5.520375	2.128359	-1.65541
H	5.917736	1.548875	-3.303399

Cartesian coordinates for conformer 4-10 after optimization at the PCM/B3LYP/6-31G* level of theory. Number of imaginary frequencies = 0. SCF Energy (PCM/mPW1PW91/6-31+G**) = -2568.99610645.

Atom	X	Y	Z
O	-3.930583	0.364815	-1.052435
O	1.193919	1.114021	0.067541
O	3.325671	0.708478	-1.421161
O	4.981421	1.09271	1.050784
O	-1.232886	1.629075	-1.07064
H	0.122611	0.124791	1.807973
O	3.204322	-0.267448	1.446626
O	-2.678526	-1.919565	-2.498294
O	-2.108686	-2.559499	-2.973711
O	-0.890409	-3.70265	-2.073351
O	-1.690743	2.446463	-3.135483
O	4.939506	-0.526673	-2.414853
O	-4.641178	2.221643	0.043981
C	-3.55778	-2.371705	-0.269183
H	-4.403418	-2.387023	-0.95922
H	-3.509209	-3.339775	0.235216
C	-3.733242	-1.238438	0.754209
C	-3.080139	-0.03762	0.042159
H	-2.903006	0.805484	0.702899
C	-1.816953	-0.644415	-0.570525
H	-1.151814	-0.819646	0.273428
C	-1.145026	0.269871	-1.599457
H	-1.712489	0.227528	-2.526399
C	0.323657	-0.014054	-1.88005
C	1.233561	-0.134151	-0.669939
H	0.832641	-0.902995	-0.019684
C	2.694969	-0.494562	-0.94214
H	2.729867	-1.252176	-1.726126
C	3.480139	-1.054947	0.265098
H	4.542007	-0.953559	0.033194
C	3.214885	-2.555981	0.613354
C	1.733634	-2.771049	0.864667
H	1.329333	-2.188317	1.693285
C	0.901249	-3.501529	0.120106
H	1.264505	-4.072349	-0.731201
C	-0.600719	-3.54804	0.294755
H	-0.922285	-2.863934	1.084246
C	-1.224139	-3.133436	-1.043757
C	-2.296951	-2.029439	-1.143037
C	3.769753	-3.426855	-0.52983
H	4.832539	-3.218395	-0.6881
H	3.259359	-3.260524	-1.482573
H	3.660539	-4.485793	-0.275789
C	3.995493	-2.891443	1.904764
H	3.618459	-2.322079	2.758319
H	5.061831	-2.664041	1.784587
C	3.896819	-3.958274	2.131936
H	-5.160414	-1.015831	1.230089
H	-5.804906	-0.804719	0.373087
H	-5.202133	-0.1702	1.918589
C	-5.531455	-1.91526	1.732606
H	-1.406057	2.632006	-1.969487
H	-4.572042	1.559234	-0.966645
H	-5.124921	1.940178	-2.316179
C	-5.831821	2.763682	-2.206585
H	-5.600341	1.081025	-2.797672
H	-4.28596	2.250861	-2.950599
H	-1.16575	3.986276	-1.320322
C	-0.134077	3.938131	-0.943809
C	-2.087948	4.214745	-0.108607
H	-3.138325	4.256315	-0.412293
H	-1.826413	5.16234	0.374669

H	-1.987042	3.417017	0.629133
C	-1.283136	5.105991	-2.356319
C	-0.6102	4.941312	-3.203007
C	-1.039514	6.069399	-1.895371
H	-2.303242	5.161501	-2.750916
C	0.58894	1.117114	1.268061
H	0.542392	2.47361	1.86156
C	1.351757	3.513178	1.382825
H	2.031751	3.323667	0.558537
C	1.28135	4.770127	1.97848
H	1.912264	5.577038	1.615783
C	0.396642	4.993023	3.037682
H	0.338146	5.97724	3.495416
C	-0.41684	3.958298	3.505066
C	-1.115582	4.137953	4.31722
H	-0.342915	2.694684	2.924322
H	-0.986673	1.885805	3.257584
H	0.738683	-0.134581	-3.143942
C	1.777203	-0.302611	-3.411507
C	0.034592	-0.056411	-3.966817
O	4.495159	0.554434	-2.090962
C	5.144493	1.891912	-2.320263
H	4.41602	2.617704	-2.693104
H	5.975317	1.785635	-3.018913
H	5.512412	2.249407	-1.351817
C	4.037873	0.762523	1.735613
C	3.652083	1.411221	3.044314
C	4.393022	1.130336	3.80131
H	2.661253	1.098321	3.375751
H	3.681152	2.497103	2.93043
H	-1.068793	-4.980065	0.64336
H	-0.791325	-5.67308	-0.156929
H	-2.154038	-5.029955	0.782611
H	-0.588448	-5.305276	1.571557
H	-2.883789	-1.653899	1.880862
C	-2.598295	-0.821605	2.914581
O	-3.001498	0.316454	3.021029
C	-1.677354	-1.504052	3.896589
H	-0.650147	-1.288976	3.585106
H	-1.836872	-1.081249	4.890706
H	-1.829543	-2.585596	3.908145

Cartesian coordinates for conformer 4-11 after optimization at the PCM/B3LYP/6-31G* level of theory. Number of imaginary frequencies = 0. SCF Energy (PCM/mPW1PW91/6-31+G**) = -2568.99632434.

Atom	X	Y	Z
O	-2.164785	-3.151043	-1.467088
O	-2.506271	-0.909925	-1.516826
O	-3.313946	1.833519	-0.774525
O	2.130009	0.203689	-0.802535
O	-4.085563	-0.536577	0.754269
H	-4.334129	-1.353291	1.234051
O	2.373674	-2.197153	-1.786496
O	4.985825	-1.858963	-0.459052
O	-0.392426	4.222483	0.88522
O	-1.332234	2.623065	2.210169
O	3.368938	-1.400988	1.069731
O	-3.295442	-2.040894	2.676779
O	1.271769	1.656981	0.695476
C	-3.307545	1.326555	2.132032
H	-3.061985	1.364413	3.197758
H	-4.384735	1.463065	2.022501
C	-2.529885	2.434978	1.386911
C	-2.127587	1.771135	0.041769
H	-1.300813	2.27289	-0.457829

C	-1.80537	0.334175	0.470566
H	-0.901185	0.460921	1.073052
C	-1.448719	-0.744177	-0.563263
H	-1.354844	-1.685487	-0.007094
C	-0.136097	-0.49495	-1.282377
C	1.092909	-0.726473	-0.430875
H	0.846795	-0.556149	0.613413
C	1.662933	-2.144644	-0.537866
H	0.829162	-2.848477	-0.587513
C	2.572883	-2.537543	0.64926
H	3.249837	-3.323083	0.311744
C	1.8263	-3.056132	1.916687
C	0.925475	-1.968175	2.477159
H	1.420261	-1.008996	2.638153
C	-0.369718	-2.086339	2.77724
H	-0.883841	-3.036576	2.651389
C	-1.255931	-0.968647	3.29699
H	-0.784737	0.004655	3.122661
C	-2.55643	-1.083035	2.500641
C	-2.959824	-0.038763	1.447974
C	-3.312678	3.738067	1.266541
H	-3.509215	4.136431	2.267737
H	-4.267388	3.546027	0.772748
H	-2.75445	4.478768	0.693205
C	-0.045803	-0.127169	-2.559652
H	0.924103	0.033763	-3.017698
H	-0.926442	0.053605	-3.161788
C	1.064333	-4.346546	1.557844
H	1.724472	-5.047419	1.038386
H	0.202694	-4.165092	0.908447
H	0.696657	-4.830954	2.468418
C	2.895836	-3.3957	2.983943
H	3.46732	-2.509899	3.274221
H	3.597231	-4.147096	2.600805
H	2.412305	-3.801598	3.878436
C	-1.548771	-1.130909	4.802627
H	-2.048907	-2.084823	4.991153
H	-2.196582	-0.324078	5.166175
H	-0.61083	-1.104407	5.365416
C	-0.385458	3.541862	1.88753
C	0.664202	3.590006	2.970915
H	0.197599	3.743345	3.948975
H	1.366913	4.396559	2.757455
H	1.197243	2.636066	2.984077
C	2.063257	1.404599	-0.199549
C	3.049129	2.364966	-0.748419
C	4.103476	1.942263	-1.571134
H	4.205473	0.890014	-1.816444
C	5.015307	2.876805	-2.054171
H	5.83835	2.552862	-2.685301
C	4.867944	4.229101	-1.733207
H	5.578117	4.956693	-2.118022
C	3.806679	4.650048	-0.928153
H	3.684897	5.704264	-0.695046
C	2.896657	3.721007	-0.429901
H	2.047142	4.031263	0.171819
C	4.550877	-1.179427	0.443726
C	5.253146	0.00257	1.067459
H	6.057114	0.337859	0.411777
H	5.675392	-0.301603	2.032313
H	4.552981	0.820849	1.25035
C	-2.772212	-2.187948	-1.886468
C	-3.182003	2.055748	-2.101723
O	-2.124277	2.17934	-2.676811
C	-4.545553	2.14898	-2.743851
H	-4.432797	2.263236	-3.822628

H	-5.130201	1.253697	-2.516655
H	-5.08618	3.009408	-2.334845
C	-3.935527	-2.236635	-2.859995
C	-3.662873	-1.35834	-4.092716
C	-5.229508	-1.830645	-2.129187
H	-4.009086	-3.283443	-3.171997
H	-3.545093	-0.308553	-3.81092
H	-4.496011	-1.435943	-4.800404
H	-2.748831	-1.67235	-4.609326
H	-5.465659	-2.541937	-1.329982
H	-5.121558	-0.845584	-1.667475
H	-6.070391	-1.814834	-2.831707
C	2.716683	-3.425047	-2.242236
O	2.391779	-4.458093	-1.6968
C	3.577561	-3.299903	-3.47109
H	3.128272	-2.605902	-4.187522
H	4.544553	-2.886084	-3.163805
H	3.718618	-4.281721	-3.924435

Cartesian coordinates for conformer 4-12 after optimization at the PCM/B3LYP/6-31G* level of theory. Number of imaginary frequencies = 0. SCF Energy (PCM/mPW1PW91/6-31+G**) = -2568.99634148.

Atom	X	Y	Z
O	-3.319611	1.82509	-0.77876
O	2.130049	0.220108	-0.802479
O	2.381187	-2.183273	-1.781931
O	4.993383	-1.824078	-0.465876
O	-2.501574	-0.919253	-1.508992
H	1.256381	1.677164	0.682869
O	3.38142	-1.375408	1.070632
O	-4.091103	-0.523165	0.758395
O	-4.329802	-1.352775	1.220789
O	-3.287138	-2.054925	2.653463
O	-2.166703	-3.161029	-1.437479
O	2.416619	-4.44343	-1.682202
O	-2.131543	2.178589	-2.680765
C	-3.297559	1.32918	2.13987
H	-4.377007	1.464299	2.052232
H	-3.030982	1.367258	3.200481
C	-2.534296	2.439182	1.379979
C	-2.133176	1.76957	0.037696
H	-1.308132	2.270724	-0.464266
C	-1.806009	0.335807	0.472533
H	-0.903144	0.468694	1.075763
C	-1.444152	-0.744554	-0.557216
H	-1.344668	-1.683763	0.001366
C	-0.13387	-0.489801	-1.278688
C	1.097087	-0.713871	-0.428268
H	0.850901	-0.542281	0.615671
C	1.672801	-2.129776	-0.532311
H	0.841077	-2.837112	-0.576945
C	2.587156	-2.514952	0.654433
H	3.266544	-3.299139	0.318623
C	1.843113	-3.032668	1.924211
C	0.93202	-1.950486	2.479241
H	1.417818	-0.986581	2.639308
C	-0.362221	-2.081146	2.778991
H	-0.867153	-3.036308	2.653245
C	-1.260084	-0.972645	3.297365
H	-0.793417	0.004038	3.131523
C	-2.55553	-1.088761	2.493169
C	-2.960179	-0.035488	1.450647
C	1.092982	-4.331999	1.570726
H	1.760867	-5.03023	1.057673
H	0.231773	-4.162386	0.917846

H	0.726773	-4.81433	2.483035
C	2.913159	-3.35848	2.995267
H	3.474663	-2.466181	3.284989
H	3.623197	-4.103676	2.615954
C	2.431166	-3.767122	3.88929
H	-3.330223	3.733693	1.253526
H	-4.286692	3.528306	0.768283
H	-2.783124	4.474503	0.669673
C	-3.52333	4.139034	2.252615
H	-2.769661	-2.200043	-1.867658
H	-3.188601	2.048743	-2.105878
H	-4.55242	2.134334	-2.748396
C	-5.131181	1.234414	-2.523678
H	-4.439649	2.251835	-3.826757
H	-5.099257	2.989842	-2.337468
H	-3.93004	-2.254016	-2.844398
C	-4.002238	-3.302367	-3.151356
C	-3.654537	-1.381364	-4.080484
H	-3.541218	-0.329696	-3.803673
H	-4.483986	-1.465436	-4.791515
H	-2.736993	-1.695433	-4.590901
C	-5.226119	-1.845447	-2.118773
C	-6.064856	-1.831267	-2.823883
C	-5.11915	-0.859003	-1.659526
H	-5.465037	-2.554588	-1.31832
C	2.053265	1.424614	-0.20708
H	3.035402	2.388441	-0.757319
C	2.867585	3.746562	-0.45493
H	2.009036	4.056235	0.133794
C	3.775774	4.678007	-0.951874
H	3.642114	5.733406	-0.730886
C	4.850785	4.257818	-1.739053
H	5.559811	4.987414	-2.122107
C	5.013502	2.903692	-2.043956
C	5.847423	2.579838	-2.660681
H	4.103554	1.966384	-1.562751
H	4.218188	0.912389	-1.795248
H	-0.046726	-0.122306	-2.556158
C	0.921974	0.042918	-3.015317
C	-0.928794	0.054375	-3.157357
O	2.735886	-3.410233	-2.230686
C	3.602797	-3.283686	-3.455082
H	3.751451	-4.265762	-3.905349
H	4.565708	-2.864387	-3.142544
H	3.154568	-2.593268	-4.175636
C	4.56084	-1.149632	0.441777
C	5.261888	0.031539	1.068948
C	6.082638	0.353045	0.427327
H	5.658761	-0.265721	2.04651
H	4.564965	0.85789	1.227517
H	-1.563001	-1.14501	4.800021
H	-2.057618	-2.103742	4.979038
H	-2.219895	-0.345379	5.163065
H	-0.62973	-1.114862	5.370422
H	-1.333021	2.64759	2.193232
C	-0.396951	3.571042	1.854934
O	-0.415659	4.23972	0.844905
C	0.65706	3.642324	2.932978
H	0.193317	3.817716	3.908877
H	1.189861	2.688837	2.966196
H	1.358947	4.444265	2.699923

Cartesian coordinates for conformer 4-13 after optimization at the PCM/B3LYP/6-31G* level of theory. Number of imaginary frequencies = 0. SCF Energy (PCM/mPW1PW91/6-31+G**) = -2568.99505101.

Atom	X	Y	Z
O	-3.166097	1.810596	-0.969014
O	2.067887	0.310891	-0.789444
O	2.532853	-2.174291	-1.454379
O	5.090309	-1.435433	-0.145321
O	-2.474421	-0.956505	-1.518963
H	1.191378	1.808249	0.653702
O	3.398782	-0.914755	1.278336
O	-4.103906	-0.567703	0.763109
O	-4.168744	-1.490178	1.085052
O	-3.188258	-2.021955	2.687041
O	-3.032674	-3.006311	-0.72019
O	2.717836	-4.395526	-1.067548
O	-1.730222	2.143839	-2.697858
C	-3.397888	1.381453	2.007323
H	-4.465712	1.49631	1.813284
H	-3.235158	1.480726	3.084586
C	-2.585741	2.459346	1.2596
C	-2.080166	1.743864	-0.022829
H	-1.198014	2.21977	-0.446003
C	-1.806564	0.316753	0.469895
H	-0.920731	0.451135	1.095582
C	-1.449661	-0.805679	-0.525817
H	-1.408858	-1.737183	0.054368
C	-0.117652	-0.614719	-1.228667
C	1.098766	-0.652132	-0.32516
H	0.811141	-0.377964	0.684325
C	1.793431	-2.013433	-0.23221
H	1.027139	-2.79073	-0.183618
C	2.714005	-2.161752	1.004327
H	3.465276	-2.918265	0.774048
C	1.999836	-2.581528	2.3263
C	0.97154	-1.537612	2.731301
H	1.355925	-0.517373	2.779709
C	-0.310648	-1.763489	3.028354
H	-0.717771	-2.771739	3.002228
C	-1.328307	-0.705011	3.406106
H	-0.935924	0.29656	3.200062
C	-2.559619	-0.986194	2.538772
C	-2.993028	-0.010825	1.433835
C	1.396226	-3.986982	2.143896
H	2.146571	-4.677166	1.747516
H	0.546627	-3.995163	1.455576
H	1.045851	-4.374443	3.10605
C	3.07593	-2.65302	3.438076
H	3.531938	-1.675926	3.618045
H	3.869819	-3.357855	3.161765
C	2.620771	-2.997303	4.372335
H	-3.37824	3.741713	1.026128
H	-4.304092	3.504666	0.497704
H	-2.803979	4.459944	0.441299
C	-3.631325	4.191612	1.992369
H	-3.201911	-2.099754	-1.507784
H	-2.857127	2.066526	-2.264687
H	-4.115847	2.280765	-3.070235
C	-3.862234	2.333579	-4.129672
H	-4.584614	3.221389	-2.759838
H	-4.835154	1.478732	-2.891257
H	-4.279659	-2.070716	-2.576873
C	-4.574351	-3.116309	-2.716594
C	-3.775964	-1.499829	-3.910605
H	-3.479722	-0.452495	-3.806414
H	-4.567603	-1.560898	-4.665812

H	-2.908961	-2.05789	-4.281946
C	-5.486649	-1.284696	-2.025407
C	-6.259442	-1.193476	-2.797201
C	-5.179829	-0.288786	-1.695917
H	-5.921117	-1.79094	-1.158171
C	1.966542	1.539335	-0.250777
H	2.899905	2.506888	-0.873206
C	2.714825	3.869214	-0.602008
H	1.876227	4.177751	0.016212
C	3.583964	4.803942	-1.159299
H	3.437885	5.862191	-0.961037
C	4.636317	4.382958	-1.975915
H	5.315147	5.115365	-2.405686
C	4.816053	3.024251	-2.249164
C	5.633134	2.699643	-2.88766
H	3.945431	2.083199	-1.706552
H	4.075532	1.024988	-1.909332
H	0.005155	-0.430827	-2.542839
C	0.984064	-0.29666	-2.989749
C	-0.859242	-0.37309	-3.190953
O	2.987346	-3.418746	-1.734
C	3.888965	-3.383792	-2.938912
H	4.810149	-2.864188	-2.652202
H	3.425055	-2.818476	-3.752547
H	4.117648	-4.400982	-3.259011
C	4.582276	-0.684783	0.657492
C	5.188188	0.60653	1.153245
C	5.718839	0.407423	2.091945
H	4.418463	1.355342	1.350643
H	5.898397	0.983212	0.416722
H	-1.715908	-0.803337	4.896058
H	-2.144	-1.786542	5.111763
H	-2.457124	-0.039824	5.160859
H	-0.828838	-0.657835	5.520006
H	-1.44078	2.69946	2.14408
C	-0.502015	3.636684	1.85451
O	-0.486629	4.317188	0.852202
C	0.517041	3.698424	2.965769
H	0.029116	3.701923	3.94458
H	1.153016	2.81183	2.893667
H	1.13061	4.592032	2.841988

Cartesian coordinates for conformer 4-14 after optimization at the PCM/B3LYP/6-31G* level of theory. Number of imaginary frequencies = 0. SCF Energy (PCM/mPW1PW91/6-31+G**) = -2568.99204766.

Atom	X	Y	Z
O	-3.658543	-2.562918	-1.388531
O	-2.733234	-0.540943	-1.847565
O	-3.006879	2.132859	-0.778976
O	2.007769	0.007661	-1.378139
O	-4.321681	-0.396441	0.550135
H	-4.397891	-1.372462	0.559166
O	1.665697	-2.718139	-1.348734
O	4.875748	-1.161821	0.228282
O	0.080177	3.781387	1.46784
O	-1.129054	2.055631	2.349376
O	2.865914	-0.689714	1.178857
O	-3.440969	-2.328004	2.042364
O	1.604326	2.036134	-0.446548
C	-3.346184	1.239656	2.029346
H	-3.274247	1.146096	3.116713
H	-4.355429	1.578544	1.787498
C	-2.314969	2.260904	1.50701
C	-1.944361	1.724048	0.103297
H	-0.995833	2.115986	-0.265865

C	-1.910532	0.204199	0.33366
H	-1.03481	0.060239	0.97101
C	-1.696099	-0.726869	-0.870583
H	-1.75341	-1.7645	-0.516921
C	-0.35588	-0.5102	-1.564415
C	0.877579	-0.532016	-0.678893
H	0.718654	0.077732	0.209755
C	1.249293	-1.938737	-0.211072
H	0.338577	-2.384325	0.187128
C	2.356216	-2.010331	0.867801
H	3.18308	-2.604322	0.473809
C	1.83974	-2.632557	2.199106
C	0.685376	-1.779501	2.688612
H	0.961017	-0.752885	2.930447
C	-0.596695	-2.136607	2.79704
H	-0.924662	-3.148228	2.565213
C	-1.699194	-1.192991	3.223884
H	-1.308703	-0.172607	3.259772
C	-2.817623	-1.289522	2.180762
C	-3.140674	-0.098917	1.259718
C	-2.828847	3.694987	1.567396
H	-2.985184	3.981148	2.613131
H	-3.78528	3.754537	1.042513
H	-2.122958	4.389763	1.115429
C	-0.250061	-0.337046	-2.882235
H	0.717675	-0.193988	-3.348679
H	-1.125635	-0.29033	-3.515592
C	1.450472	-4.098493	1.936458
H	2.32917	-4.666728	1.611244
H	0.677303	-4.211155	1.170189
H	1.076594	-4.556264	2.85763
C	2.975638	-2.596209	3.243816
H	3.249664	-1.568739	3.501403
H	3.870189	-3.101936	2.861401
H	2.659363	-3.103402	4.161302
C	-2.268167	-1.577065	4.605704
H	-2.691159	-2.585314	4.572578
H	-3.060352	-0.883591	4.912144
H	-1.472492	-1.549214	5.356493
C	0.001276	2.800676	2.166545
C	1.151985	2.193656	2.933568
H	1.903242	2.9626	3.122424
H	1.602882	1.416502	2.305646
H	0.821158	1.738536	3.870586
C	2.263618	1.322557	-1.180706
C	3.468571	1.764445	-1.924742
C	4.32902	0.850762	-2.549318
H	4.114085	-0.210414	-2.497757
C	5.468129	1.315328	-3.202036
H	6.142679	0.608891	-3.677564
C	5.746843	2.683204	-3.238418
H	6.636664	3.041113	-3.75036
C	4.888557	3.593678	-2.615364
H	5.107638	4.657563	-2.644047
C	3.75263	3.136081	-1.954098
H	3.072726	3.820199	-1.455487
C	4.150773	-0.399869	0.823544
C	4.52817	0.979097	1.306082
H	5.473096	1.272316	0.84841
H	4.639374	0.958776	2.396462
H	3.748391	1.704411	1.062802
C	-3.649276	-1.525615	-2.014761
C	-2.661057	2.567047	-2.019803
O	-1.540773	2.524901	-2.471089
C	-3.864514	3.143871	-2.725797
H	-3.607189	3.35221	-3.764913

H	-4.713666	2.459031	-2.675332
H	-4.159682	4.074971	-2.228892
C	-4.695746	-1.124617	-3.040839
C	-4.09756	-0.404779	-4.258062
C	-5.761523	-0.270669	-2.323157
H	-5.157415	-2.064007	-3.363383
H	-3.616641	0.533255	-3.968282
H	-4.88637	-0.178836	-4.98457
H	-3.345831	-1.026003	-4.758473
H	-6.276789	-0.853024	-1.553673
H	-5.297214	0.582622	-1.822326
H	-6.504275	0.088547	-3.04464
C	0.775887	-3.628376	-1.828909
O	-0.283598	-3.887386	-1.302451
C	1.301576	-4.2467	-3.098318
H	1.295132	-3.487714	-3.888356
H	2.336328	-4.575709	-2.964707
H	0.665536	-5.083851	-3.387686

Cartesian coordinates for conformer 5-1 after optimization at the PCM/B3LYP/6-31G* level of theory. Number of imaginary frequencies = 0. SCF Energy (PCM/mPW1PW91/6-31+G**) = -1776.18906634.

Atom	X	Y	Z
C	4.8198	-2.7085	-0.3696
C	3.4395	-2.0808	-0.5938
O	3.4593	-0.8809	0.2025
C	4.8158	-0.4664	0.4891
C	5.7159	-1.4692	-0.2521
C	0.6024	-2.56	1.5707
C	-0.0322	-2.5549	0.1748
O	1.0627	-2.212	-0.6834
C	2.2239	-2.9421	-0.2288
C	2.0079	-3.1399	1.3021
C	4.6082	1.3635	-1.3257
C	5.0083	1.0172	0.124
O	4.0918	1.7514	0.9614
C	3.2933	2.6916	0.2224
C	3.9039	2.7221	-1.1941
C	-1.2274	-1.6092	-0.0788
C	1.7948	2.2971	0.3169
C	6.4334	1.4696	0.4706
C	2.2941	-4.2771	-0.988
C	0.9244	3.4147	-0.319
C	-0.5014	2.9884	-0.5043
C	-1.5557	3.4566	0.1658
C	-2.9578	2.8899	0.0354
C	-3.582	2.7164	1.4331
C	-3.844	3.7798	-0.8479
C	1.4073	2.0715	1.7858
O	1.5468	1.0964	-0.4386
C	-3.7002	-1.3618	0.6413
C	-2.4054	-2.1085	0.801
C	-6.1611	-1.1573	0.0676
C	-4.8383	-1.8968	0.195
C	-1.5881	-1.607	-1.5711
O	-0.9359	-0.2688	0.3564
C	-6.9608	-1.669	-1.1385
C	-6.9756	-1.3061	1.365
O	-2.9094	1.6146	-0.6327
O	-5.98	0.2696	-0.0149
O	-5.3943	0.6131	-1.3026
H	5.1167	-3.3775	-1.1819
H	4.8311	-3.2809	0.5662
H	3.325	-1.7979	-1.6515
H	4.9527	-0.5539	1.5751

H	6.6503	-1.6615	0.2825
H	5.9721	-1.0944	-1.2504
H	0.0363	-3.1509	2.2965
H	0.6614	-1.5306	1.9353
H	-0.3786	-3.5719	-0.0765
H	2.7723	-2.6192	1.8838
H	2.0557	-4.2031	1.5607
H	5.472	1.4019	-1.9963
H	3.9038	0.6234	-1.7124
H	3.4013	3.667	0.7162
H	3.1569	2.8682	-1.977
H	4.6263	3.5426	-1.2663
H	6.5507	2.5359	0.2513
H	7.187	0.9185	-0.1032
H	6.631	1.3245	1.5382
H	3.1181	-4.9022	-0.6269
H	1.3658	-4.8399	-0.8495
H	2.4268	-4.1073	-2.0623
H	0.9868	4.3074	0.3157
H	1.348	3.6809	-1.2962
H	-0.6582	2.1871	-1.2206
H	-1.436	4.2693	0.8842
H	-4.5765	2.2673	1.3459
H	-2.9571	2.0638	2.0541
H	-3.6756	3.6811	1.946
H	-4.8347	3.3267	-0.9601
H	-3.394	3.8874	-1.8395
H	-3.9576	4.7756	-0.4063
H	1.5685	2.9773	2.3829
H	2.0179	1.2731	2.2153
H	0.3532	1.7912	1.8551
H	2.1663	0.4033	-0.1208
H	-3.6952	-0.313	0.924
H	-2.5677	-3.1721	0.5843
H	-2.08	-2.0357	1.8478
H	-4.863	-2.9513	-0.0871
H	-1.8277	-2.6187	-1.92
H	-0.7452	-1.2373	-2.1616
H	-2.4583	-0.9702	-1.7493
H	-0.1066	0.0557	-0.0638
H	-7.9053	-1.1213	-1.2185
H	-6.3959	-1.523	-2.0614
H	-7.184	-2.7363	-1.0244
H	-7.944	-0.8037	1.2674
H	-6.4311	-0.8673	2.2067
H	-7.1491	-2.3653	1.5828
H	-2.2507	1.033	-0.1887
H	-4.4872	0.8931	-1.0242

Cartesian coordinates for conformer 5-2 after optimization at the PCM/B3LYP/6-31G* level of theory. Number of imaginary frequencies = 0. SCF Energy (PCM/mPW1PW91/6-31+G**) = -1776.18361675.

Atom	X	Y	Z
C	4.824228	-2.715413	-0.371657
C	3.423905	-2.111696	-0.532946
O	3.460108	-0.875933	0.203385
C	4.807853	-0.463151	0.491134
C	5.710664	-1.467254	-0.250928
C	1.337344	-2.254044	2.102122
C	0.379322	-1.830599	0.976582
O	1.060598	-2.116713	-0.254888
C	2.219832	-2.954473	-0.065065
C	2.216355	-3.321811	1.439675
C	4.707525	1.342472	-1.378115
C	5.02585	1.008367	0.105222

O	4.074909	1.782481	0.858996
C	3.903742	3.033882	0.180922
C	3.902397	2.663033	-1.315078
C	-0.037694	-0.339027	0.99831
C	2.653837	3.784421	0.703328
C	6.455879	1.421001	0.499933
C	2.075094	-4.184644	-0.967499
C	2.690979	5.198171	0.060414
C	1.546428	6.111106	0.404346
C	0.661058	6.549526	-0.494051
C	-0.579721	7.388987	-0.249529
C	-0.706332	7.902576	1.192445
C	-0.624429	8.558969	-1.24115
C	2.667449	3.837822	2.237605
O	1.445155	3.153961	0.251819
C	-1.556676	1.23967	2.355521
C	-0.866007	-0.094298	2.288725
C	-1.744015	3.65826	3.106609
C	-1.126915	2.276856	3.076109
C	-0.818327	0.019348	-0.273246
O	1.131705	0.49344	1.094049
C	-3.213703	3.7175	2.682287
C	-1.550025	4.299913	4.48681
O	-1.715408	6.57272	-0.576124
O	-0.95255	4.539346	2.254314
O	-1.09915	4.11361	0.871276
H	5.102015	-3.353925	-1.215027
H	4.88649	-3.318896	0.541053
H	3.242376	-1.868431	-1.590908
H	4.948999	-0.54289	1.578233
H	6.646259	-1.658208	0.281956
H	5.965339	-1.08843	-1.247508
H	0.801141	-2.636304	2.97571
H	1.937244	-1.396923	2.415741
H	-0.54125	-2.43435	1.003564
H	3.220478	-3.359636	1.871197
H	1.775744	-4.317756	1.564172
H	5.6231	1.441185	-1.969654
H	4.105156	0.548126	-1.827243
H	4.769473	3.676363	0.408026
H	2.870885	2.51073	-1.642214
H	4.342335	3.450574	-1.933492
H	6.628903	2.481097	0.291296
H	7.206218	0.851955	-0.059705
H	6.619998	1.257758	1.570902
H	2.910807	-4.880766	-0.829412
H	1.146379	-4.711162	-0.726887
H	2.035294	-3.895007	-2.02315
H	3.640012	5.665001	0.36389
H	2.728717	5.076249	-1.028315
H	1.453662	6.411542	1.446073
H	0.76025	6.222112	-1.531258
H	-1.631656	8.481045	1.283977
H	-0.757795	7.067445	1.897762
H	0.13455	8.548894	1.471451
H	-1.580639	9.085299	-1.146286
H	-0.54085	8.191783	-2.269606
H	0.189537	9.267838	-1.053214
H	3.555926	4.361099	2.610805
H	2.677984	2.822058	2.643251
H	1.772714	4.349318	2.602339
H	1.376966	2.244658	0.63162
H	-2.459922	1.343272	1.758014
H	-1.619533	-0.891552	2.363257
H	-0.188195	-0.20364	3.142043
H	-0.2092	2.172364	3.657143

H	-1.753866	-0.54996	-0.342233
H	-0.216209	-0.207423	-1.156456
H	-1.058247	1.086275	-0.274792
H	1.873823	0.070121	0.611522
H	-3.563893	4.753599	2.727476
H	-3.344824	3.363764	1.657726
H	-3.832877	3.107828	3.348702
H	-1.912698	5.332492	4.483961
H	-0.492175	4.302641	4.770064
H	-2.107549	3.734028	5.24047
H	-1.612068	5.738931	-0.072177
H	-0.162407	3.855394	0.65812

Cartesian coordinates for conformer 5-3 after optimization at the PCM/B3LYP/6-31G* level of theory. Number of imaginary frequencies = 0. SCF Energy (PCM/mPW1PW91/6-31+G**) = -1776.18443405.

Atom	X	Y	Z
C	4.673381	-2.817402	-0.6442
C	3.288804	-2.160709	-0.711617
O	3.400667	-0.955895	0.069418
C	4.771332	-0.636167	0.367201
C	5.606963	-1.60934	-0.48332
C	1.287765	-2.322348	1.995192
C	0.305742	-1.843938	0.912992
O	0.938446	-2.116395	-0.346814
C	2.081999	-2.985698	-0.219376
C	2.124438	-3.388669	1.275541
C	4.713874	1.353652	-1.306718
C	5.053479	0.853688	0.119033
O	4.154178	1.5896	0.965692
C	4.066157	2.92562	0.452052
C	4.127174	2.76962	-1.085077
C	-0.062816	-0.340615	0.981079
C	2.81408	3.646182	1.010747
C	6.511342	1.157179	0.512076
C	1.872829	-4.189719	-1.14473
C	2.928248	5.135401	0.588989
C	1.788363	6.023752	1.002232
C	1.060656	6.757596	0.158584
C	-0.106196	7.646421	0.562342
C	0.312359	9.123522	0.500946
C	-1.299072	7.381498	-0.378396
C	2.743798	3.479372	2.535678
O	1.618127	3.126753	0.410109
C	-1.556792	1.239894	2.355533
C	-0.865891	-0.094522	2.288714
C	-1.731572	3.673086	3.057807
C	-1.123588	2.287419	3.058526
C	-0.848298	0.075348	-0.270448
O	1.136619	0.448818	1.078613
C	-3.177139	3.745443	2.561002
C	-1.594226	4.323629	4.44034
O	-0.496229	7.418276	1.915786
O	-0.88846	4.532692	2.229033
O	-0.952853	4.085372	0.847371
H	4.895555	-3.411271	-1.5354
H	4.751093	-3.478893	0.226293
H	3.06383	-1.866948	-1.747756
H	4.929815	-0.825954	1.438565
H	6.559345	-1.864929	-0.010861
H	5.82543	-1.168093	-1.462832
H	0.770473	-2.719575	2.873606
H	1.9146	-1.487202	2.314889
H	-0.631561	-2.420235	0.95416
H	3.142394	-3.452852	1.670211

H	1.671278	-4.379769	1.392133
H	5.592548	1.359793	-1.959022
H	3.963711	0.697422	-1.756953
H	4.939609	3.49383	0.808014
H	3.11907	2.845203	-1.49916
H	4.742283	3.549883	-1.542737
H	6.735787	2.222071	0.398676
H	7.217882	0.605814	-0.117605
H	6.690298	0.885922	1.558537
H	2.695759	-4.908757	-1.056171
H	0.941345	-4.700002	-0.881241
H	1.800075	-3.872602	-2.190655
H	3.865396	5.516823	1.022886
H	3.042562	5.181019	-0.500844
H	1.550847	6.079602	2.061877
H	1.28266	6.730558	-0.910355
H	-0.536684	9.764066	0.765033
H	1.123722	9.310345	1.211204
H	0.656556	9.395832	-0.503119
H	-2.145969	8.011877	-0.085643
H	-1.598512	6.330114	-0.315347
H	-1.045076	7.603811	-1.422386
H	3.631544	3.899165	3.023474
H	2.686163	2.417595	2.79043
H	1.85529	3.981951	2.926248
H	1.47274	2.197634	0.710691
H	-2.461155	1.33792	1.75885
H	-1.617952	-0.891552	2.379395
H	-0.171611	-0.200782	3.129196
H	-0.206958	2.192638	3.642129
H	-1.806285	-0.456161	-0.335709
H	-0.269488	-0.156012	-1.167633
H	-1.047212	1.150245	-0.244086
H	1.855429	0.017356	0.568326
H	-3.525698	4.782391	2.602686
H	-3.254556	3.405394	1.526421
H	-3.832671	3.131521	3.187647
H	-1.928331	5.365545	4.410854
H	-0.553863	4.302752	4.781243
H	-2.206426	3.780117	5.167689
H	-0.752633	6.478443	1.993908
H	-0.001465	3.840564	0.701514

Cartesian coordinates for conformer 5-4 after optimization at the PCM/B3LYP/6-31G* level of theory. Number of imaginary frequencies = 0. SCF Energy (PCM/mPW1PW91/6-31+G**) = -1776.18295182.

Atom	X	Y	Z
C	4.818911	-2.707407	-0.369247
C	3.445334	-2.071509	-0.605684
O	3.458204	-0.881536	0.202534
C	4.815291	-0.457128	0.473654
C	5.717884	-1.469656	-0.252487
C	0.554812	-2.594458	1.500623
C	-0.040801	-2.5358	0.089343
O	1.07541	-2.162616	-0.725402
C	2.216797	-2.923795	-0.272578
C	1.972586	-3.155165	1.249776
C	4.555477	1.340716	-1.363982
C	4.995675	1.019598	0.080335
O	4.097488	1.760082	0.930157
C	3.26938	2.678672	0.200416
C	3.83187	2.689809	-1.236277
C	-1.239382	-1.584996	-0.151086
C	1.773824	2.286217	0.347256
C	6.426156	1.485849	0.383931

C	2.281448	-4.242541	-1.061109
C	0.903486	3.433883	-0.230474
C	-0.551749	3.077761	-0.352828
C	-1.476705	3.395926	0.554812
C	-2.916065	2.922237	0.59043
C	-3.815028	4.006995	1.192048
C	-3.460623	2.455777	-0.764069
C	1.445029	2.031744	1.825306
O	1.47639	1.105616	-0.423168
C	-3.814133	-1.661607	0.267988
C	-2.448432	-2.193441	0.610672
C	-6.043144	-0.585102	0.916082
C	-4.63292	-1.089612	1.153447
C	-1.495951	-1.457744	-1.660655
O	-1.013944	-0.287799	0.416188
C	-6.562482	-0.815654	-0.504962
C	-6.997458	-1.186183	1.958687
O	-2.957715	1.817675	1.538739
O	-6.070015	0.864908	1.018643
O	-5.560342	1.270931	2.317388
H	5.118995	-3.380464	-1.176969
H	4.819941	-3.276123	0.568834
H	3.350272	-1.778007	-1.662976
H	4.957026	-0.522668	1.560317
H	6.647116	-1.660374	0.291434
H	5.983914	-1.103723	-1.251488
H	-0.029292	-3.214836	2.186737
H	0.597147	-1.579451	1.905005
H	-0.375213	-3.541956	-0.214678
H	2.719083	-2.635512	1.855354
H	2.029505	-4.222308	1.488685
H	5.40291	1.383438	-2.055217
H	3.855368	0.583287	-1.724149
H	3.386415	3.663572	0.673096
H	3.053895	2.803225	-1.994044
H	4.535291	3.521659	-1.352315
H	6.528277	2.551052	0.151515
H	7.169108	0.935517	-0.204477
H	6.653712	1.353287	1.447326
H	3.094726	-4.88303	-0.70265
H	1.34716	-4.801255	-0.948372
H	2.431436	-4.049842	-2.129253
H	1.025922	4.306426	0.42291
H	1.291756	3.713727	-1.21743
H	-0.820484	2.478676	-1.219635
H	-1.177245	3.976172	1.429633
H	-4.83623	3.635459	1.31585
H	-3.438373	4.308816	2.175681
H	-3.834589	4.88914	0.543059
H	-4.482698	2.090491	-0.632149
H	-2.858283	1.63645	-1.170093
H	-3.462361	3.274889	-1.491875
H	1.658067	2.916063	2.437917
H	2.054949	1.208289	2.205229
H	0.388868	1.776288	1.939111
H	2.083852	0.392388	-0.129216
H	-4.146807	-1.816933	-0.756953
H	-2.447949	-3.276059	0.413855
H	-2.263652	-2.064285	1.683086
H	-4.283609	-0.957489	2.176703
H	-1.727862	-2.430844	-2.110921
H	-0.607337	-1.061125	-2.15881
H	-2.334372	-0.780857	-1.845586
H	-0.224452	0.129574	0.003221
H	-7.549739	-0.356328	-0.611126
H	-5.895871	-0.368267	-1.248166

H	-6.654782	-1.886646	-0.711338
H	-8.008996	-0.793327	1.810025
H	-6.67002	-0.919205	2.966703
H	-7.026017	-2.278597	1.874817
H	-2.413483	1.088861	1.171984
H	-4.613031	1.448369	2.08263

Cartesian coordinates for conformer 5-5 after optimization at the PCM/B3LYP/6-31G* level of theory. Number of imaginary frequencies = 0. SCF Energy (PCM/mPW1PW91/6-31+G**) = -1776.17741962.

Atom	X	Y	Z
C	4.825727	-2.71425	-0.371645
C	3.438603	-2.090187	-0.570029
O	3.460447	-0.875661	0.20339
C	4.813916	-0.523233	0.562549
C	5.708826	-1.46869	-0.250945
C	0.662945	-2.161093	1.512388
C	-0.025525	-2.337081	0.155494
O	1.068758	-2.311716	-0.776048
C	2.228061	-2.958605	-0.187353
C	1.938256	-3.005586	1.33891
C	4.442128	1.557719	-0.928432
C	5.052919	0.987206	0.375929
O	4.335418	1.643916	1.450839
C	3.481989	2.688549	0.943241
C	3.133449	2.220533	-0.471758
C	-1.025471	-1.252756	-0.299136
C	2.342003	2.848314	1.978858
C	6.540838	1.319469	0.540851
C	2.34909	-4.354043	-0.813841
C	1.243286	3.834864	1.512136
C	0.273298	3.316566	0.484681
C	-0.025064	3.932452	-0.661412
C	-1.036442	3.46111	-1.691229
C	-2.160534	4.498007	-1.838522
C	-0.338703	3.222112	-3.044796
C	2.941306	3.350242	3.306739
O	1.726184	1.576607	2.189625
C	-3.187369	-0.066386	0.408514
C	-2.122071	-1.063228	0.777258
C	-5.529091	0.606991	-0.289875
C	-4.452069	-0.382165	0.123758
C	-1.619188	-1.618538	-1.666775
O	-0.296257	-0.011329	-0.413643
C	-6.455053	-0.008005	-1.350159
C	-6.33469	1.061377	0.940353
O	-1.691039	2.256157	-1.276639
O	-4.976558	1.847458	-0.764389
O	-4.284614	1.622468	-2.027493
H	5.112945	-3.373764	-1.195197
H	4.85914	-3.29643	0.558004
H	3.305422	-1.825546	-1.631013
H	4.944507	-0.726267	1.636684
H	6.668553	-1.659975	0.236926
H	5.912179	-1.051444	-1.245263
H	0.046911	-2.496611	2.351799
H	0.907574	-1.104291	1.655577
H	-0.531334	-3.315482	0.107318
H	2.773417	-2.613039	1.923996
H	1.763254	-4.040466	1.654242
H	5.12128	2.30178	-1.361682
H	4.27731	0.782318	-1.681758
H	4.049971	3.632781	0.900036

H	2.327716	1.485919	-0.417552
H	2.817017	3.038359	-1.12341
H	6.672632	2.405885	0.564574
H	7.137895	0.921099	-0.28689
H	6.93235	0.912947	1.48017
H	3.152711	-4.936221	-0.349705
H	1.412028	-4.903427	-0.676995
H	2.543615	-4.284986	-1.889968
H	0.669383	4.104011	2.41127
H	1.721498	4.760756	1.165206
H	-0.224011	2.386792	0.74436
H	0.481106	4.864927	-0.921925
H	-2.904176	4.148098	-2.562324
H	-2.661142	4.644074	-0.876994
H	-1.762314	5.460257	-2.177645
H	-1.072112	2.910028	-3.796165
H	0.420192	2.436047	-2.95237
H	0.159121	4.130996	-3.402966
H	3.339258	4.368069	3.208066
H	3.754527	2.695953	3.634014
H	2.164113	3.354924	4.077335
H	2.461615	0.938058	2.186813
H	-2.876964	0.973674	0.345186
H	-2.579008	-2.04416	0.966977
H	-1.624829	-0.745552	1.702335
H	-4.774427	-1.423869	0.184081
H	-2.20922	-2.541008	-1.603784
H	-0.819304	-1.774552	-2.396607
H	-2.278362	-0.82035	-2.017751
H	0.583868	-0.260221	-0.752992
H	-7.226636	0.715027	-1.633846
H	-5.887145	-0.277108	-2.243481
H	-6.945711	-0.907231	-0.959302
H	-7.129279	1.753523	0.642137
H	-5.678317	1.562389	1.658165
H	-6.78936	0.195855	1.434086
H	-1.051881	1.530914	-1.099495
H	-3.357255	1.81826	-1.751313

Cartesian coordinates for conformer 5-6 after optimization at the PCM/B3LYP/6-31G* level of theory. Number of imaginary frequencies = 0. SCF Energy (PCM/mPW1PW91/6-31+G**) = -1776.18264904.

Atom	X	Y	Z
C	4.749144	-2.642232	-0.521984
C	3.372852	-1.996248	-0.7718
O	3.441751	-0.70099	-0.1443
C	4.753667	-0.443187	0.393247
C	5.672612	-1.43212	-0.329588
C	0.630145	-2.370529	1.604864
C	-0.060463	-2.464928	0.239074
O	0.982592	-2.107097	-0.678365
C	2.172773	-2.805775	-0.25547
C	2.056852	-2.883205	1.299236
C	5.006222	1.65134	-1.14409
C	5.076851	1.060033	0.284407
O	4.046392	1.735782	1.030583
C	3.233776	2.592795	0.212225
C	3.608313	2.277326	-1.247316
C	-1.333051	-1.621053	-0.003173
C	1.736575	2.372956	0.558247
C	6.420284	1.341114	0.96802
C	2.192701	-4.198551	-0.911687
C	0.91201	3.478902	-0.151599
C	-0.56489	3.401247	0.113076
C	-1.464124	3.008135	-0.790556

C	-2.937311	2.746151	-0.561468
C	-3.418966	3.056855	0.861113
C	-3.795207	3.476893	-1.601377
C	1.531751	2.392788	2.080368
O	1.289359	1.113542	0.030344
C	-3.87003	-1.804732	0.619815
C	-2.446542	-2.207143	0.911995
C	-6.054517	-0.607518	1.200237
C	-4.597751	-0.995467	1.394878
C	-1.702192	-1.672993	-1.494229
O	-1.169702	-0.260446	0.42409
C	-6.879896	-1.636577	0.422506
C	-6.694108	-0.319918	2.565662
O	-3.129846	1.336199	-0.834826
O	-6.137525	0.690819	0.550658
O	-5.738163	0.566621	-0.836862
H	5.05755	-3.293751	-1.344101
H	4.728144	-3.246514	0.392824
H	3.211994	-1.829719	-1.845811
H	4.733307	-0.67682	1.468937
H	6.570802	-1.668666	0.247824
H	5.989004	-1.03139	-1.299729
H	0.126511	-2.955831	2.379859
H	0.645947	-1.32395	1.921771
H	-0.350976	-3.510895	0.047725
H	2.812487	-2.263574	1.789056
H	2.196407	-3.912417	1.645884
H	5.783795	2.415761	-1.253481
H	5.180972	0.901425	-1.920806
H	3.486732	3.633431	0.466497
H	2.89191	1.564099	-1.659631
H	3.599876	3.173401	-1.874801
H	6.585872	2.421446	1.020948
H	7.252178	0.891927	0.413009
H	6.431592	0.947248	1.990115
H	3.050336	-4.78997	-0.57391
H	1.287915	-4.758488	-0.657074
H	2.23602	-4.109686	-2.002884
H	1.303355	4.451366	0.180548
H	1.093871	3.403982	-1.229839
H	-0.889565	3.651283	1.121215
H	-1.128818	2.752382	-1.797179
H	-4.46654	2.759427	0.963173
H	-2.836895	2.494427	1.599509
H	-3.329838	4.124716	1.09122
H	-4.835517	3.144114	-1.522435
H	-3.440712	3.254869	-2.613538
H	-3.753844	4.560077	-1.446323
H	1.785387	3.371251	2.50715
H	2.17071	1.64262	2.552226
H	0.489945	2.163374	2.319272
H	2.001327	0.448146	0.138727
H	-4.329218	-2.262139	-0.254917
H	-2.390173	-3.303697	0.849961
H	-2.192542	-1.929349	1.940812
H	-4.114839	-0.532832	2.25775
H	-1.89324	-2.703776	-1.817977
H	-0.878834	-1.279702	-2.096335
H	-2.594029	-1.073421	-1.690417
H	-0.332637	0.132904	0.086629
H	-7.914258	-1.286563	0.34365
H	-6.493933	-1.765413	-0.59008
H	-6.873084	-2.604387	0.935336
H	-7.730053	0.008353	2.437933
H	-6.143529	0.468028	3.090823
H	-6.683261	-1.222433	3.186369

H	-2.500603	0.817271	-0.279825
H	-4.776357	0.806734	-0.782496

Cartesian coordinates for conformer 5-7 after optimization at the PCM/B3LYP/6-31G* level of theory. Number of imaginary frequencies = 0. SCF Energy (PCM/mPW1PW91/6-31+G**) = -1776.17612423.

Atom	X	Y	Z
C	4.825036	-2.716448	-0.371987
C	3.421202	-2.116848	-0.523779
O	3.460865	-0.875276	0.203405
C	4.81024	-0.47205	0.50526
C	5.709098	-1.466876	-0.250618
C	1.314278	-2.320905	2.137042
C	0.365288	-1.877191	1.010452
O	1.058359	-2.136061	-0.22035
C	2.224717	-2.966239	-0.043024
C	2.23596	-3.344061	1.459616
C	4.700274	1.387919	-1.318776
C	5.035171	1.008519	0.15389
O	4.10802	1.762864	0.953053
C	3.823949	2.996364	0.27167
C	3.71971	2.576916	-1.203074
C	-0.035379	-0.38089	1.053781
C	2.587008	3.67005	0.918556
C	6.471669	1.403757	0.540085
C	2.077419	-4.195093	-0.948282
C	2.467915	5.154807	0.490435
C	2.212868	5.415299	-0.969935
C	2.912395	6.273657	-1.719363
C	2.656944	6.663521	-3.162582
C	3.901371	6.387047	-4.016286
C	1.419516	5.996948	-3.777169
C	2.691707	3.599028	2.451114
O	1.372702	3.042923	0.483866
C	-1.52936	1.229211	2.42061
C	-0.881872	-0.129173	2.328652
C	-1.668566	3.618288	3.31355
C	-1.074708	2.229266	3.179624
C	-0.782802	0.014155	-0.227246
O	1.152699	0.417953	1.190532
C	-3.1079	3.747484	2.809974
C	-1.556911	4.089886	4.770659
O	2.500989	8.100137	-3.213704
O	-0.813086	4.573247	2.624057
O	-0.924653	4.382038	1.189703
H	5.100315	-3.351965	-1.218544
H	4.892575	-3.32244	0.538549
H	3.228638	-1.879759	-1.581107
H	4.947356	-0.573522	1.591138
H	6.649789	-1.658298	0.272881
H	5.953709	-1.079268	-1.246434
H	0.771589	-2.746735	2.98619
H	1.884658	-1.460723	2.493926
H	-0.558089	-2.476918	1.015279
H	3.241146	-3.339916	1.890161
H	1.841155	-4.359575	1.577107
H	5.609502	1.661117	-1.864356
H	4.237521	0.551743	-1.850586
H	4.675332	3.68358	0.410636
H	2.694023	2.257972	-1.404022
H	3.959116	3.396296	-1.883657
H	6.625852	2.476849	0.389914
H	7.213193	0.875699	-0.069813
H	6.662867	1.180832	1.595709
H	2.91847	-4.887284	-0.82281

H	1.155142	-4.728473	-0.698261
H	2.023111	-3.902191	-2.002368
H	1.618644	5.559278	1.058374
H	3.362524	5.695651	0.822828
H	1.366895	4.879686	-1.394988
H	3.75227	6.810373	-1.272616
H	3.754922	6.782754	-5.027297
H	4.778456	6.87838	-3.582536
H	4.097658	5.311786	-4.082425
H	1.27536	6.373264	-4.794661
H	0.518245	6.227474	-3.197348
H	1.521684	4.906899	-3.820886
H	3.607723	4.084815	2.808128
H	2.712164	2.557319	2.780693
H	1.827607	4.099964	2.897793
H	1.324951	2.112542	0.816532
H	-2.431968	1.374237	1.829834
H	-1.664847	-0.900142	2.37073
H	-0.227425	-0.282527	3.193601
H	-0.160698	2.073928	3.755602
H	-1.730276	-0.531711	-0.318316
H	-0.17243	-0.215897	-1.103756
H	-0.99814	1.086363	-0.219241
H	1.886429	0.011577	0.678929
H	-3.449239	4.778082	2.95002
H	-3.172124	3.514978	1.745528
H	-3.774742	3.078706	3.364805
H	-1.912581	5.120655	4.86335
H	-0.517183	4.050512	5.113067
H	-2.159513	3.447835	5.421955
H	1.78294	8.322792	-2.598863
H	-0.105838	3.856338	1.00033

Cartesian coordinates for conformer 5-8 after optimization at the PCM/B3LYP/6-31G* level of theory. Number of imaginary frequencies = 0. SCF Energy (PCM/mPW1PW91/6-31+G**) = -1776.17597425.

Atom	X	Y	Z
C	4.824296	-2.715394	-0.371664
C	3.428641	-2.107027	-0.545084
O	3.46174	-0.876329	0.203062
C	4.812342	-0.481228	0.51883
C	5.708964	-1.466876	-0.250598
C	1.195572	-2.201024	1.990335
C	0.305703	-1.811315	0.796645
O	1.055797	-2.139105	-0.38344
C	2.215238	-2.950453	-0.104601
C	2.143193	-3.261692	1.410246
C	4.68424	1.439446	-1.233612
C	5.047033	1.005562	0.203541
O	4.12428	1.741613	1.020757
C	3.976841	3.060886	0.4678
C	4.124334	2.873447	-1.063814
C	-0.089853	-0.313848	0.734066
C	2.650354	3.679311	0.979071
C	6.493197	1.377976	0.579033
C	2.129399	-4.213965	-0.968575
C	2.608649	5.207592	0.717748
C	2.553948	5.643418	-0.721282
C	3.432932	6.471417	-1.294473
C	3.437121	6.971464	-2.725675
C	2.285371	6.426246	-3.578898
C	3.447665	8.50501	-2.748077
C	2.504293	3.430101	2.489066
O	1.529545	3.122178	0.279624
C	-1.686568	1.363603	1.874166

C	-1.028181	0.00871	1.926396
C	-1.913787	3.806451	2.58368
C	-1.312202	2.415253	2.606344
C	-0.741128	0.011746	-0.617501
O	1.088272	0.492729	0.909732
C	-3.259921	3.90904	1.863545
C	-2.019937	4.34916	4.016442
O	4.698864	6.592863	-3.326367
O	-0.957509	4.725508	1.983734
O	-0.855232	4.459142	0.560324
H	5.106087	-3.358839	-1.209854
H	4.8791	-3.313464	0.54584
H	3.262729	-1.854679	-1.603292
H	4.944132	-0.606366	1.602823
H	6.654456	-1.659598	0.263943
H	5.943147	-1.071658	-1.246177
H	0.608503	-2.581047	2.831646
H	1.755544	-1.327326	2.330325
H	-0.62056	-2.406245	0.786807
H	3.125351	-3.244214	1.891196
H	1.732618	-4.268024	1.551495
H	5.543611	1.401198	-1.910484
H	3.911126	0.774218	-1.62878
H	4.792729	3.693037	0.851355
H	3.151943	2.974369	-1.548347
H	4.790481	3.630927	-1.485725
H	6.671654	2.44647	0.423932
H	7.220271	0.831726	-0.031341
H	6.684583	1.155596	1.634696
H	2.959624	-4.898542	-0.759248
H	1.191525	-4.738036	-0.760718
H	2.146537	-3.963254	-2.034811
H	1.702818	5.574617	1.218942
H	3.464724	5.673286	1.2215
H	1.70213	5.270714	-1.285895
H	4.276659	6.841616	-0.707787
H	2.390688	6.793346	-4.604508
H	2.297852	5.329912	-3.60729
H	1.309519	6.741057	-3.194098
H	3.560322	8.862931	-3.777285
H	4.287487	8.889044	-2.159695
H	2.517346	8.904666	-2.332232
H	3.355772	3.84381	3.042118
H	2.454608	2.358059	2.694291
H	1.58718	3.907967	2.846395
H	1.388244	2.183399	0.554378
H	-2.525218	1.462837	1.187338
H	-1.80874	-0.765299	1.959174
H	-0.43996	-0.082633	2.846091
H	-0.465035	2.305804	3.285379
H	-1.679598	-0.54142	-0.749933
H	-0.0686	-0.260116	-1.434211
H	-0.956681	1.082021	-0.679788
H	1.861282	0.049064	0.497664
H	-3.612388	4.944699	1.900658
H	-3.163455	3.624674	0.814205
H	-4.007394	3.267449	2.342604
H	-2.37221	5.385373	4.003204
H	-1.046132	4.321606	4.51701
H	-2.723802	3.743418	4.596854
H	4.748669	5.624762	-3.271413
H	-0.010665	3.942157	0.522913

Cartesian coordinates for conformer 5-9 after optimization at the PCM/B3LYP/6-31G* level of theory. Number of imaginary frequencies = 0. SCF Energy (PCM/mPW1PW91/6-31+G**) = -1776.17710433.

Atom	X	Y	Z
C	4.824601	-2.715926	-0.371816
C	3.421894	-2.114719	-0.527435
O	3.460482	-0.875623	0.203392
C	4.809997	-0.466518	0.497326
C	5.709917	-1.467051	-0.250776
C	1.312434	-2.323678	2.12826
C	0.364198	-1.876422	1.002395
O	1.057642	-2.13322	-0.228715
C	2.223249	-2.964576	-0.052345
C	2.231602	-3.347628	1.448887
C	4.691808	1.373485	-1.346801
C	5.032685	1.010469	0.129414
O	4.110008	1.774172	0.925627
C	3.804954	2.990228	0.221123
C	3.677597	2.533295	-1.239131
C	-0.035379	-0.379984	1.049522
C	2.576513	3.673684	0.873738
C	6.470433	1.410576	0.505728
C	2.076714	-4.189779	-0.962636
C	2.449541	5.150352	0.421916
C	2.202999	5.386404	-1.043838
C	2.860577	6.274825	-1.793971
C	2.561743	6.588872	-3.250385
C	2.400788	8.111059	-3.430925
C	3.681492	6.056531	-4.156564
C	2.701135	3.628679	2.405854
O	1.356193	3.039973	0.466768
C	-1.526377	1.227305	2.423327
C	-0.880521	-0.131552	2.325831
C	-1.656053	3.616238	3.318637
C	-1.063542	2.227451	3.177193
C	-0.783384	0.018872	-0.229792
O	1.153137	0.417757	1.18808
C	-3.099109	3.746806	2.825927
C	-1.533778	4.083579	4.776358
O	1.375484	5.925281	-3.701382
O	-0.805741	4.573348	2.626044
O	-0.92811	4.387625	1.191916
H	5.101119	-3.353005	-1.21677
H	4.889632	-3.320327	0.539944
H	3.233882	-1.875362	-1.585137
H	4.949013	-0.556417	1.583922
H	6.648008	-1.658076	0.277707
H	5.960003	-1.083585	-1.246739
H	0.769025	-2.749448	2.977099
H	1.884787	-1.465037	2.485607
H	-0.559587	-2.475528	1.005373
H	3.236176	-3.34826	1.880766
H	1.833216	-4.362199	1.562535
H	5.595493	1.673418	-1.88793
H	4.258608	0.523101	-1.880971
H	4.655028	3.685219	0.327924
H	2.658112	2.17786	-1.406764
H	3.873641	3.340971	-1.946831
H	6.620628	2.483145	0.347453
H	7.210145	0.880107	-0.10431
H	6.667501	1.195431	1.561919
H	2.91853	-4.881668	-0.840233
H	1.154904	-4.724969	-0.714826
H	2.021887	-3.892195	-2.015322
H	1.593465	5.555616	0.979301
H	3.336852	5.703635	0.753848

H	1.400195	4.804572	-1.491922
H	3.672234	6.857643	-1.352962
H	2.177194	8.341533	-4.477937
H	1.579554	8.488426	-2.809157
H	3.31108	8.649049	-3.141041
H	3.474654	6.312145	-5.201657
H	3.742069	4.967355	-4.071559
H	4.649757	6.485274	-3.876727
H	3.622078	4.119283	2.742989
H	2.725345	2.592622	2.752458
H	1.842886	4.137085	2.855582
H	1.317079	2.111479	0.805596
H	-2.434583	1.372789	1.841139
H	-1.664504	-0.90166	2.36615
H	-0.225464	-0.288541	3.189616
H	-0.143657	2.071874	3.743642
H	-1.731247	-0.526283	-0.321447
H	-0.173888	-0.209088	-1.10755
H	-0.99802	1.091137	-0.219383
H	1.886756	0.011696	0.675996
H	-3.439428	4.777012	2.971119
H	-3.170803	3.517043	1.761365
H	-3.761671	3.07642	3.383864
H	-1.888964	5.114061	4.874823
H	-0.491611	4.043395	5.11075
H	-2.131358	3.439476	5.43014
H	0.652476	6.20685	-3.116686
H	-0.113352	3.858094	0.994435
